# Triazenyl Furans as Diels–Alder Dienes

**DOI:** 10.1021/jacs.6c06794

**Published:** 2026-06-01

**Authors:** Jessica E. Budwitz, Skyler A. Hollers, Abigail R. Wester, Christopher G. Newton

**Affiliations:** Department of Chemistry, 1355 University of Georgia, Athens, Georgia 30602, United States

## Abstract

2-Triazenyl furans are introduced as bench-stable, electron-rich Diels–Alder dienes, enabling convergent and divergent access to highly substituted arenes under redox-neutral conditions. The triazene substituent activates the furan toward cycloaddition, promotes *in situ* aromatization, and serves as a versatile handle for downstream functionalization. Diels–Alder reactions are demonstrated with a broad range of dienophiles, including alkenes, alkynes, allenes, and benzyne, in both inter- and intramolecular settings. Chemoselective derivatizations highlight the synthetic versatility of this platform, culminating in concise annulative syntheses of pomalidomide, (*S*)-apremilast, and related analogues.

## Graphical abstract

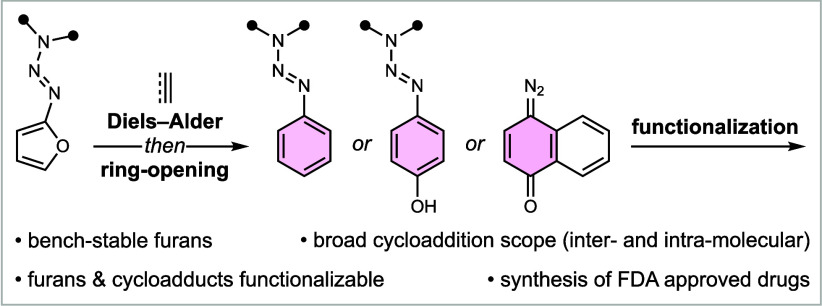

Highly substituted arenes feature prominently in a wide variety of pharmaceuticals,^1^ agrochemicals,^2^ and materials.^3^ Synthetic approaches to these motifs typically rely on iterative functionalization of preexisting aromatic frameworks (e.g., directed metalation,^4^ Friedel–Crafts reactions,^5^ cross-coupling,^6^ and C–H functionalization^7^). Although these “peripheral editing” strategies are highly effective in many contexts,^8^ they often come at the expense of step economy, a limiting factor in many target-oriented settings.^9^ Annulative approaches offer an attractive alternative by virtue of their inherent convergency; however, such strategies usually require *de novo* reaction design considerations that can limit generality.^10^

Diels–Alder/aromatization sequences of furans are well-established redox-neutral annulation processes^11^ that, if successfully paired with a subsequent cross-coupling protocol, could provide a modular and retrosynthetically simple approach^12^ to complex aromatic scaffolds. In practice, furans bearing conventional cross-coupling functionality (or direct precursors thereof) are poorly matched to this reactivity profile (Figure 1a). For example, while electron-donating substituents activate furans toward cycloaddition, this often comes at the expense of stability. Indeed, simple 2- and 3-aminofurans, which could serve as diazonium precursors, have been described as “*extremely unstable*”^13^ and “*unisolable*”,^14^ with their generation inferred only through *in situ* trapping experiments.^15^ While silyloxyfurans exhibit improved stability profiles, they remain moisture sensitive,^16^ and their functionalization post cycloaddition requires multiple steps. Notably, electron-withdrawing substituents can also be destabilizing. For example, McCarthy wrote of 2-bromofuran: “*on exposure to air at room temperature, it polymerized explosively*”.^17^ Similarly, the instability of furans bearing a sulfonate has been noted,^18^ which likely accounts for their limited representation in the literature. With respect to nucleophilic cross-coupling handles, a small number of silicon-, tin-, and boron-substituted furans have been shown to participate in Diels–Alder cycloadditions, but only with ultrareactive dienophiles (e.g., arynes).^11b,19^

**Figure 1:**
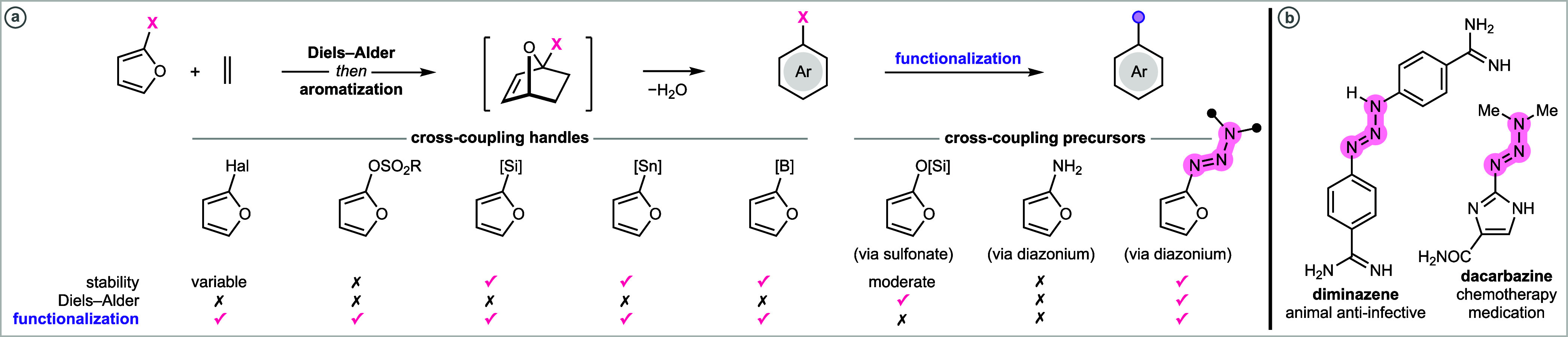
Diels–Alder reactions of triazenyl furans for the convergent and divergent synthesis of highly substituted aromatics.

To address this methodological gap, and advance fundamental understanding of structure and reactivity, we sought to develop a new class of cross-coupling compatible furyl diene. Building upon the elegant studies of Severin and co-workers on triazenyl-substituted alkynes, alkenes, and heteroaromatics,^20^ we hypothesized that the electron-donating and sterically undemanding nature of triazenyl substituents would (i) activate furans toward cycloaddition, (ii) facilitate cycloadduct aromatization, and (iii) serve as a functional handle for subsequent derivatization via mild diazonium generation.^21^ Moreover, (hetero)­aryl triazenes themselves are valuable biologically active targets (Figure 1b);^22^ thus, new approaches to this chemical space should facilitate their application in pharmaceutically relevant contexts.

To date, only two examples of triazenyl-substituted dienes participating in Diels–Alder reactions have been reported. In 2021, Severin and Cramer demonstrated that triazenyl pyrones undergo Diels–Alder/retro-Diels–Alder sequences; however, the forcing conditions led to cleavage of the triazene during aromatization.^23^ In a subsequent report, Cui and co-workers showed that highly arylated triazenyl butadienes participate in Diels–Alder reactions to furnish cyclohexenes,^24^ although four equivalents of the diene were required owing to a competing diene-transmissive pathway.^25^ With respect to the preparation of triazenyl furans, only two substrates have been reported in the literature (alongside two misassigned examples, see Supporting Information), neither of which were successfully applied in a subsequent transformation.^26^

Scouting experiments in our laboratory revealed that triazenyl furans bearing a free NH are significantly less stable than fully *N*-alkylated or *N*-arylated analogs, a trend we tentatively attribute to tautomerization-promoted decomposition (Figure 2a). Thus, to avoid the isolation of sensitive intermediates we adopted Severin’s one-pot triazenylation/intramolecular alkylation strategy,^27^ in this context proceeding via addition of a 2-lithiated furan to an azide bearing a tethered tosylate, followed by spontaneous cyclization to furnish the fully substituted triazene (Figure 2b). To date, this approach has enabled access to more than 40 2-triazenyl furans in our laboratory, including multiple examples prepared on >1 g scale.^28^

**Figure 2:**
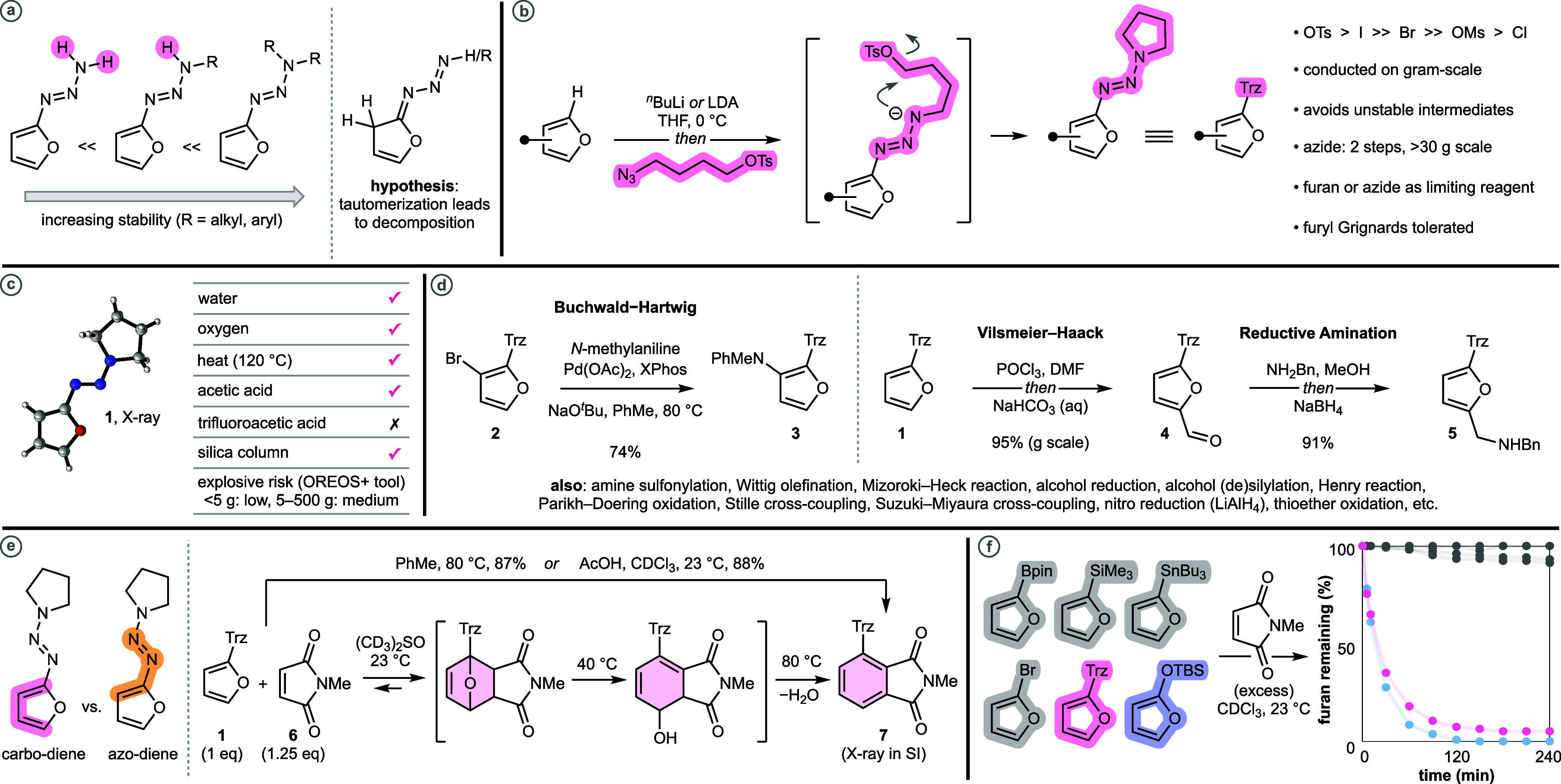
Synthesis of triazenyl furans and a proof-of-concept Diels–Alder/aromatization.

A detailed stability assessment revealed that triazenyl furan **1** is stable under standard laboratory conditions but, as expected, decomposes in the presence of strong acids (e.g., TFA, Figure 2c). Given its relatively high nitrogen-to-carbon ratio, potential explosive hazard was evaluated using Vertex Pharmaceuticals’ OREOS+ tool,^29^ which integrates multiple parameters to estimate handling risk. By this assessment, compound **1** is considered low risk on <5 g scale and medium risk on 5–500 g scale (full details in Supporting Information), a hazard profile analogous to commonly employed reagents such as sodium difluoromethanesulfinate and phenyl hydrazine. Furthermore, AstraZeneca and Merck’s shock sensitivity prediction model categorizes furan **1** as not impact sensitive,^30^ consistent with a qualitative hammer test performed in our laboratory.^31^ The excellent stability profile of triazenyl furans enables a range of peripheral functionalization reactions, provided strong acids are avoided (Figure 2d). Representative examples include Buchwald–Hartwig cross-coupling (**2** to **3**), Vilsmeier–Haack formylation (**1** to **4**), and reductive amination (**4** to **5**), alongside several additional classes of transformation detailed within the Supporting Information.

We next examined the Diels–Alder reactivity of triazenyl furan **1** using *N*-methylmaleimide (**6**) as dienophile (Figure 2e). Compound **1** contains both a carbo- and an azo-diene, raising the possibility of competing cycloaddition pathways. Gratifyingly, ^1^H NMR analysis revealed that a reversible Diels–Alder reaction occurs at ambient temperature—exclusively at the carbo-diene site—with the equilibrium favoring an inconsequential mixture of diastereomeric cycloadducts. Heating to approximately 40 °C induced irreversible cleavage of the bridging ether, and further heating to 80 °C resulted in aromatization through loss of water. This Diels–Alder/aromatization sequence could be conducted either thermally or at ambient temperature in the presence of a weak acid (AcOH), in either case providing triazenyl arene **7** in excellent yield.

To benchmark the activating effect of the triazenyl substituent we performed a competition experiment using several furan families discussed within the introduction (Figure 2f). Using a large excess of *N*-methylmaleimide as dienophile, we observed 2-triazenyl furan **1** reacts at a rate comparable to that of its *tert*-butyldimethylsilyloxy analogue, whereas boronic ester-, silane-, stannane-, and bromide-substituted furans all reacted significantly more slowly.

With proof-of-concept established the scope of the Diels–Alder reaction was investigated, with most reactions run using only a slight excess of dienophile and under an ambient atmosphere. With respect to furan scope (Figure 3a), *N*-phenylmaleimide was selected as a representative alkenic dienophile, and reactions were performed in toluene at 80 °C (**8**–**28**). Select highlights include furans directly bound to heteroatoms (**8**, **9**, **14**, **18**), heterocycles (**11**, **12**, **16**, **24**), and arenes (**10**, **15**), as well as various examples incorporating fluorine (**10**, **26**), sulfonamide (**21**), alcohol (**18**, **19**, **22**, **28**), silyloxy (**20**, **27**), and alkenyl (**23**) functionality. Notably, all seven possible furan substitution patterns are represented, including several tri- and tetrasubstituted derivatives (**25**–**28**).

**Figure 3:**
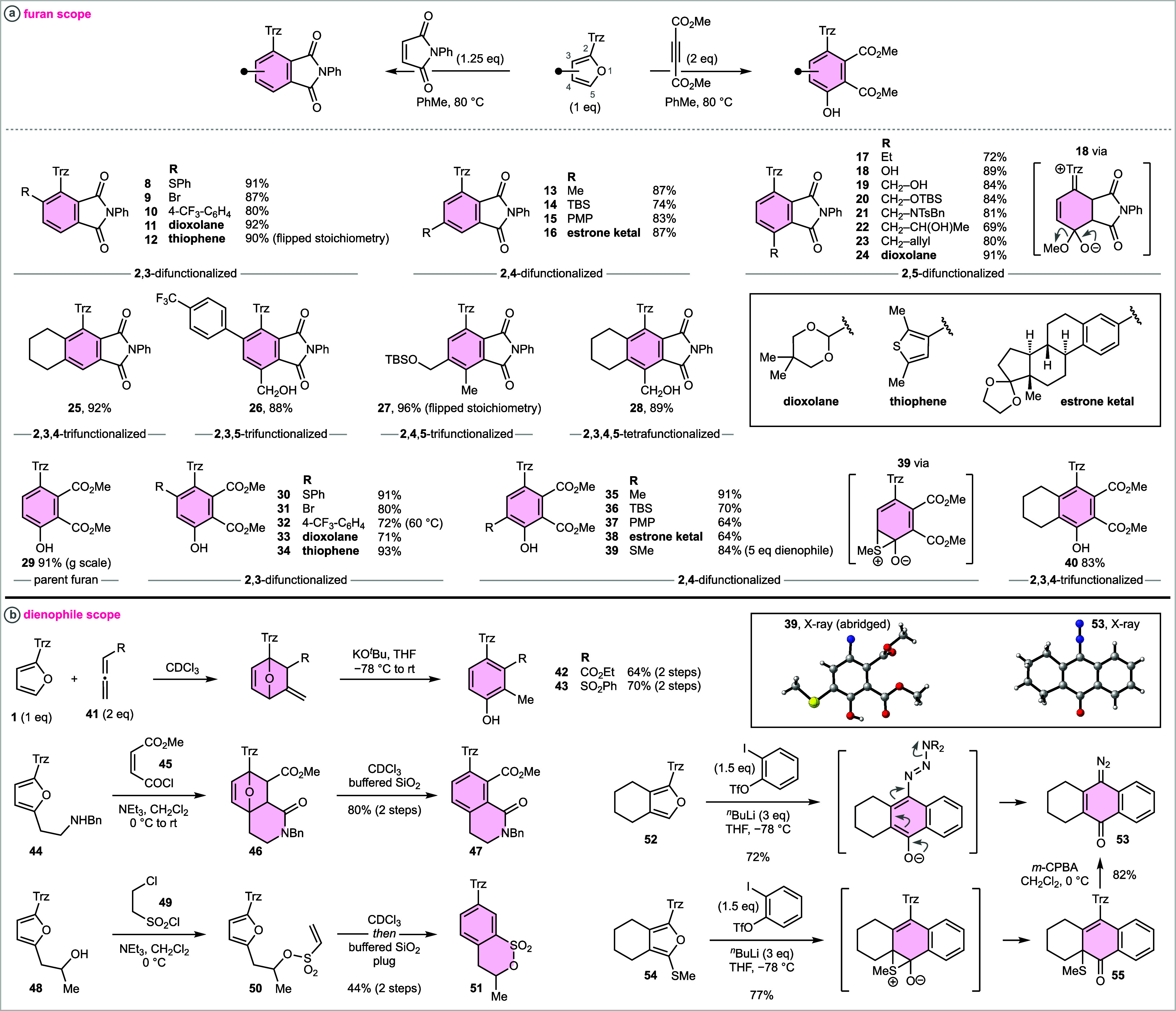
Diels–Alder scope.

Reactivity with alkynic dienophiles was evaluated using dimethyl acetylenedicarboxylate, and a similarly broad substrate scope was observed (**29**–**40**). In these cases, dehydration is not required for aromatization, leaving the phenolic functionality available for secondary derivatization through standard sulfation/cross-coupling methods. Introduction of a thioether at the 5-position of the furan generates an intermediate that cannot aromatize via the standard pathway. Instead, a 1,2-sulfur migration occurs, as observed for **39**, likely proceeding via a thiiranium intermediate.^32^

We next explored dienophile scope (Figure 3b). A major challenge in Diels–Alder reactions lies in achieving complete regiocontrol in intermolecular settings. Guided by our prior work on cumulenic dienophiles,^33^ we identified allenic dienophiles of general structure **41** as effective partners in this regard. Here, aromatization requires isomerization of the exocyclic olefin, which was achieved via treatment of the direct cycloadduct with potassium *tert*-butoxide, affording either **42** or **43** in 64–70% yield.

Intramolecular reactivity for the synthesis of polycyclic systems was also demonstrated. Coupling of amine **44** with acid chloride **45** installed a doubly activated dienophile that underwent an *in situ* intramolecular Diels–Alder reaction. The resulting cycloadduct **46** was treated with phosphate-buffered silica to promote aromatization,^34^ providing lactam **47** in 80% yield over the two steps. The same strategy was also applied to alcohol **48** and sulfonyl chloride **49**. In this case, cycloaddition of intermediate **50** required several hours at ambient temperature, ultimately yielding oxathiine **51**.

Finally, arynes were explored for the convergent synthesis of naphthalene derivatives.^11f,34,35^ Unexpectedly, *in situ* benzyne generation in the presence of furan **52** resulted in elimination of the pyrrolidine fragment to yield the moderately sensitive quinone diazide **53** (although this proved inconsequential for subsequent derivatization, see Figure 4c). In turn, thiol-substituted analog **54** gave the corresponding 1,2-sulfur migration product **55**. Interestingly, this compound serves as a new class of stable, protected quinone diazide, which can be unmasked in high yield upon treatment with one equivalent of anhydrous *m*-CPBA.

**Figure 4:**
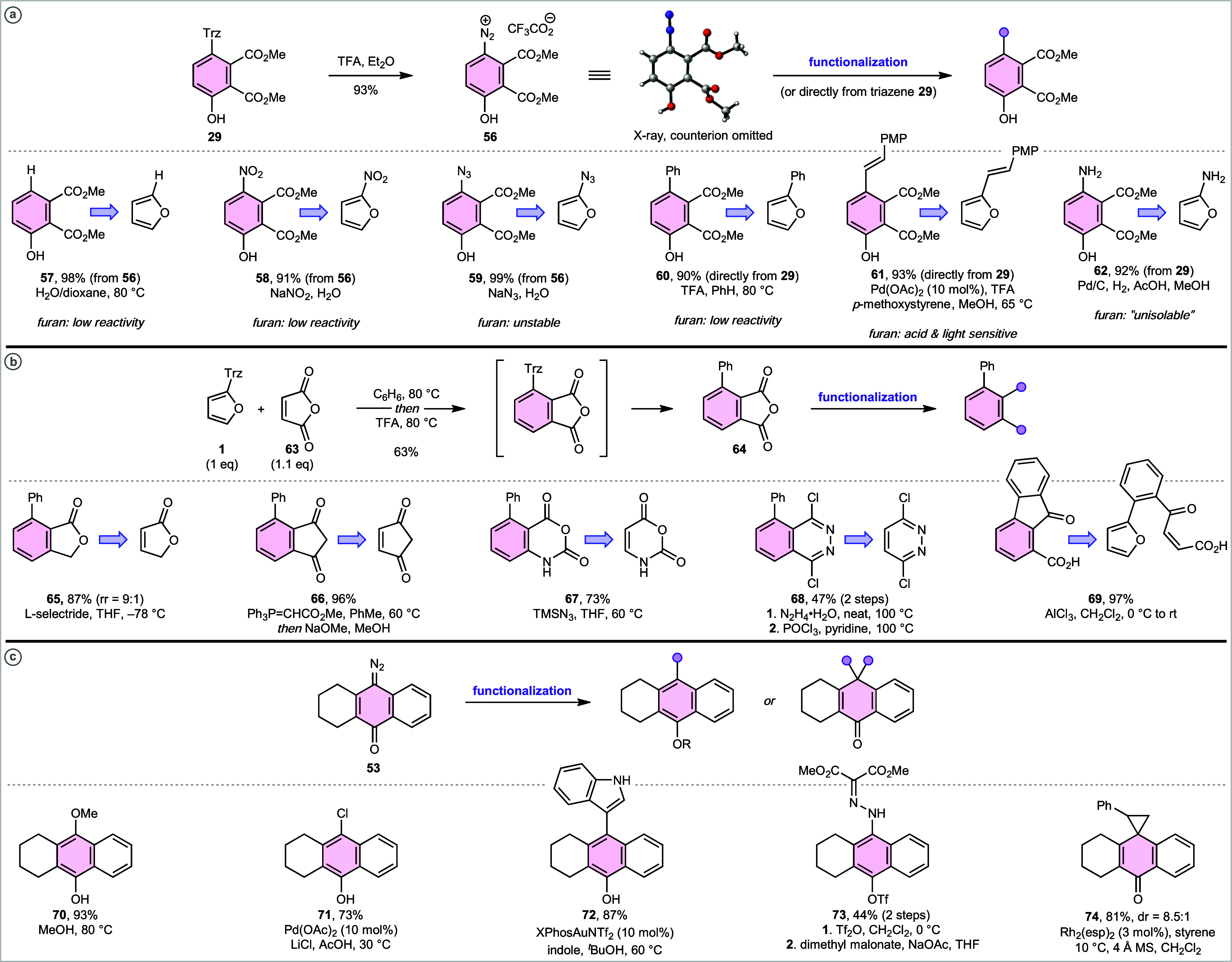
Cycloadduct derivatization.

A broad range of derivatization reactions were performed to demonstrate the versatility of this platform (Figure 4). With respect to functionalization of the triazene component, cycloadduct **29** was selected in order to demonstrate chemoselectivity in the presence of both ester and phenolic functionality. In terms of reaction design, we elected to prioritize substitution patterns that are otherwise challenging to access via conventional furan Diels–Alder disconnections (Figure 4a).^13,36^ Accordingly, conversion of **29** to diazonium salt **56** proceeded smoothly under standard conditions and in excellent yield. Warming this intermediate in aqueous 1,4-dioxane afforded the reductively cleaved product **57**, whereas treatment with sodium salts effected nitration (**58**) or azidation (**59**). Isolation of the diazonium intermediate is not always required. For example, direct treatment of **29** with TFA in benzene furnished biaryl **60**, while palladium-catalyzed interception of the *in situ* generated diazonium enabled a Mizoroki–Matsuda coupling to give **61**.^37^ Diazonium formation could also be bypassed entirely, as demonstrated by palladium-catalyzed hydrogenolysis of **29** to aniline **62**, proceeding here in 93% yield.

Derivatization of the dienophile-derived fragment was next examined, again focusing on substitution patterns not readily accessible via Diels–Alder approaches (Figure 4b). In this case, maleic anhydride (**63**) served as dienophile, and the resulting aromatized cycloadduct was converted *in situ* into phenyl-substituted phthalic anhydride **64**. Subsequent reduction with a sterically encumbered hydride reagent afforded phthalide **65** in good yield. Alternatively, a one-pot O-to-C atom exchange furnished **66**,^38^ which formally corresponds to the product of a Diels–Alder reaction with cyclopentenedione, a dienophile approximately 3 orders of magnitude more expensive than maleic anhydride. Additional derivatizations included selective *N*-insertion (**67**),^39^ an anhydride-to-pyridazine heterocycle exchange (**68**), and an intramolecular Friedel–Crafts acylation (**69**).

Quinone diazide **53** also proved to be a versatile intermediate (Figure 4c). Heating in methanol promoted substitution and aromatization, yielding unsymmetric naphthalene **70**. Conceptually related transition metal-catalyzed transformations incorporating chlorine (**71**)^40^ or indole (**72**)^41^ were also achieved. A two-step triflation/nucleophilic addition sequence enabled preservation of the diazonium functionality to furnish azo-derivative **73**,^42^ and finally, rhodium-catalyzed cyclopropanation with styrene afforded spirocycle **74** in an 8.5:1 diastereomeric ratio.^43^

To conclude the present study, this new disconnection was applied in convergent syntheses of several FDA-approved drugs, and related derivatives thereof (Figure 5). Pomalidomide,^44^ an anticancer agent typically prepared from nitro-substituted phthalic anhydrides or phthalimides,^45^ was first targeted. In our approach, a Diels–Alder reaction of the parent 2-triazenyl furan 1 with racemic maleimide **75** was conducted in DMF at 80 °C. Upon cooling to ambient temperature, *in situ* hydrogenolysis of the aryl triazene furnished **77** in 91% yield. This strategy also proved effective for (*S*)-apremilast, in this case employing enantiopure dienophile **(** ***S*** **)-76**. Subsequent acetylation delivered **78** in excellent yield with no detectable erosion of enantiopurity. Two previously unreported apremilast derivatives were also prepared via the same sequence (**79** and **80**). Notably, the corresponding nitro-substituted phthalic anhydrides or phthalimides required to access these analogues have not been described.

**Figure 5:**
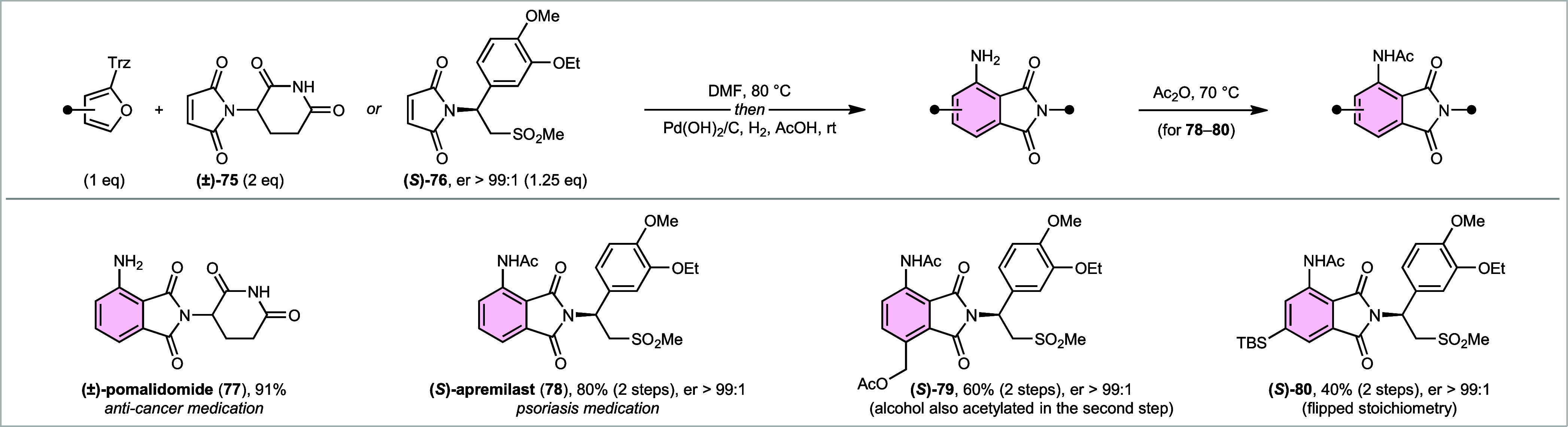
Convergent syntheses of (±)-pomalidomide, (*S*)-apremilast, and related derivatives.

In summary, we have established triazenyl furans as a new class of bench-stable Diels–Alder dienes enabling convergent and divergent access to densely substituted aromatic scaffolds. The reaction accommodates a diverse set of dienophiles, including alkenes, alkynes, allenes, and arynes, and the resulting products can be readily functionalized to provide substitution patterns inaccessible via conventional annulative disconnections. The utility of this platform is demonstrated by concise, convergent syntheses of pomalidomide, (*S*)-apremilast, and related derivatives. Overall, the triazene substituent provides a unique combination of stability, activation, and versatility, thereby addressing longstanding limitations associated with furan Diels–Alder strategies. This methodology is expected to streamline access to highly substituted arenes, while also providing a foundation for the broader application of triazenyl furans beyond Diels–Alder settings.

## Supplementary Material



## Notes

Experimental details, characterization data, and spectra (PDF)
