# Supplementary material for: Triazenyl Furans as Diels–Alder Dienes
Source: J Am Chem Soc. 2026 Jun 1;148(23):23474–80. doi: 10.1021/jacs.6c06794 (PMC13281527; doi:10.1021/jacs.6c06794)
Supplement: Supplementary file 1 [file ja6c06794_si_001.pdf]

Triazenyl Furans as Diels–Alder Dienes

Jessica E. Budwitz,[§] Skyler A. Hollers,[§] Abigail R. Wester, and Christopher G. Newton[*]

Department of Chemistry, University of Georgia, Athens, Georgia 30602, United States

[*] Email: chris.newton@uga.edu

[§] *J.E.B. and S.A.H. contributed equally to this work*

PRIOR WORK **3**

AMINOFURAN STABILITY TRENDS **4**

GENERAL EXPERIMENTAL **5**

EXPERIMENTAL PROCEDURES AND CHARACTERIZATION DATA **8**

SYNTHESIS OF AZIDES 8
SYNTHESIS OF 2-TRIAZENYL FURANS 14
Scouting Experiments *14*
One-Pot Furan Triazenylation/Intramolecular Alkylation *19*
Triazenylation Precursors 19
One-Pot Triazenylation/Intramolecular Alkylation 28
Derivatization of 2-Triazenyl Furans 40
SYNTHESIS OF A 3-TRIAZENYL FURAN 54
TRIAZENYL FURAN STABILITY AND HAZARD ASSESSMENT 55
DIELS–ALDER REACTIONS 62
Mechanism *62*
Benchmarking *65*
N-Phenylmaleimide as Dienophile *67*
Dimethyl Acetylenedicarboxylate as Dienophile *79*
Allenes as Dienophiles *86*
Tethered Dienophiles *88*
Benzyne as Dienophile *90*
DERIVATIZATION EXPERIMENTS 92
From Cycloadduct 29 .... *92*
From Phthalic Anhydride 64 .... *97*
From Quinone Diazide 53 .... *102*
SYNTHESIS OF POMALIDOMIDE, APREMILAST, AND RELATED DERIVATIVES 107

X-RAY CRYSTALLOGRAPHIC DATA **116**

NMR SPECTRA **122**

AZIDES 122
2-TRIAZENYL FURANS 130
Scouting Experiments *130*
One-Pot Furan Triazenylation/Intramolecular Alkylation *136*
Triazenylation Precursors 136
One-Pot Triazenylation/Intramolecular Alkylation 154
Derivatization of 2-Triazenyl Furans 194
SYNTHESIS OF A 3-TRIAZENYL FURAN 236
DIELS–ALDER REACTIONS 238
Mechanistic Experiment *238*
N-Phenylmaleimide as Dienophile *242*
Dimethyl Acetylenedicarboxylate as Dienophile *289*
Allenes as Dienophiles *315*
Tethered Dienophiles *319*
Benzyne as Dienophile *323*
DERIVATIZATION EXPERIMENTS 328
From Cycloadduct 29 .... *328*
From Phthalic Anhydride 64 .... *341*
From Quinone Diazide 53 .... *351*
SYNTHESIS OF POMALIDOMIDE, APREMILAST, AND RELATED DERIVATIVES 366

REFERENCES **378**

Prior Work

Four earlier publications describe the synthesis of a triazenyl furan.[1]

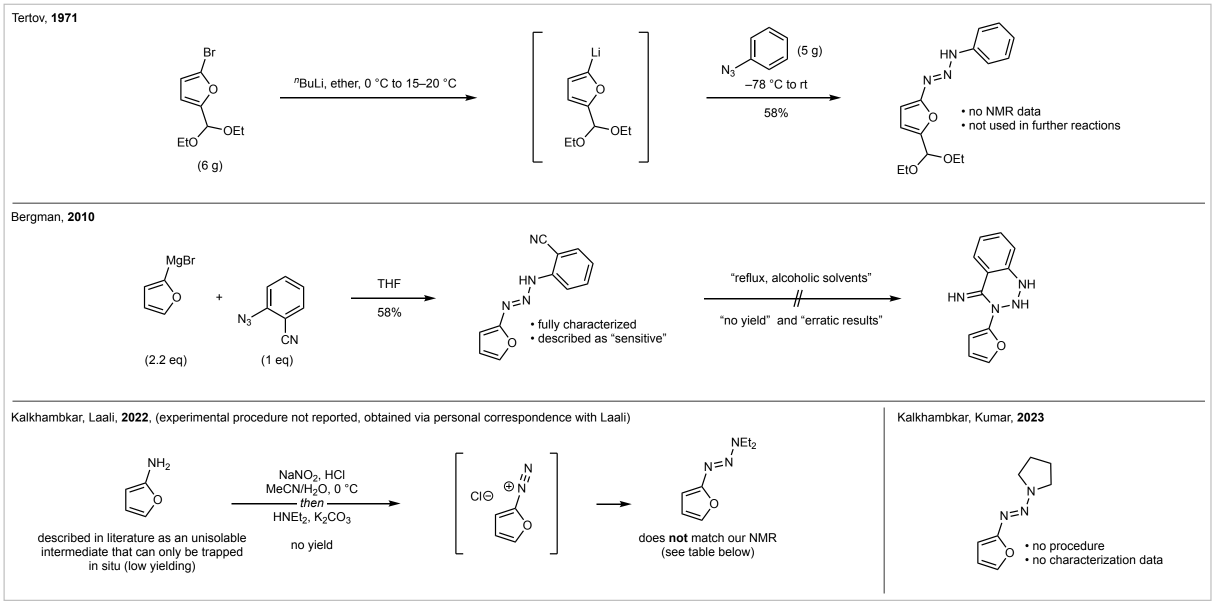

With respect to the 2022 work, we call into question the accuracy of this disclosure. First, their synthesis begins from 2-aminofuran, a highly unstable compound (see next page), which they then treat with a strong acid. Second, there are significant NMR inconsistencies relative to our own work (table below). By extension, a follow-up study from the same group in 2023 may also be problematic, although here, no experimental or characterization data were provided.

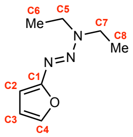

#	^{1}H NMR Kalkhambkar & Laali (400 MHz, $CDCl_3$)	^{1}H NMR This Work (400 MHz, $CDCl_3$)	^{13}C NMR* Kalkhambkar & Laali (100 MHz, $CDCl_3$)	^{13}C NMR This Work (100 MHz, $CDCl_3$)
**C1**	–	–	159.1	159.5
**C2**	7.27 – 7.41 (m, 3H)	7.19 (s, 1H)	125.4	137.7
**C3**		6.37 (s, 1H)	118.7	95.0
**C4**		6.04 (d, J = 3.4 Hz, 1H)	120.7	111.8
**C5 / C7**	3.09 – 3.12 (br m, 2H), 2.84 – 2.87 (br m, 2H)	3.72 (q, J = 7.2 Hz, 4H)	51.4 (br), 49.7 (br)	48.9 (br), 41.2 (br)
**C6 / C8**	1.84 – 2.03 (br m, 6H)	1.24 (s, 6H)	24.7, 24.6	14.2 (br), 11.6 (br)

*^{13}C NMR shifts not assigned by Kalkhambkar and Laali, rather assignments were made by us via extrapolation to the most similar resonances in our own work. No NMR screenshots provided.

Aminofuran Stability Trends

The following trends support our triazenyl furan stability hypothesis (i.e., that tautomerization leads to decomposition).

2-Aminofurans[2]

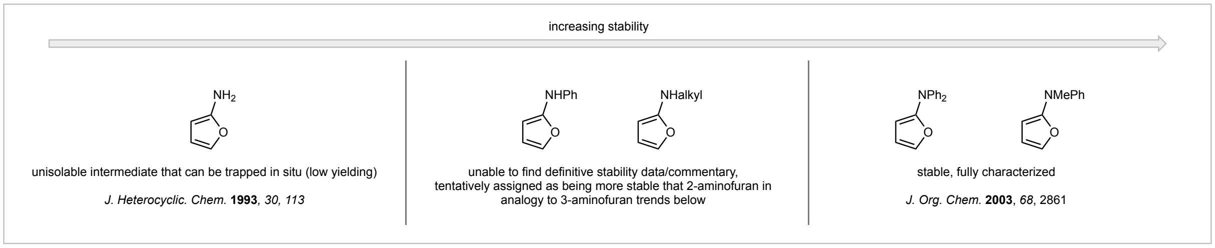

3-Aminofurans[2b, 3]

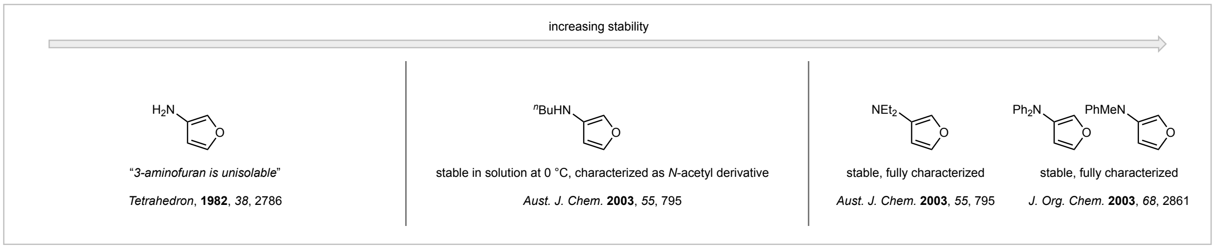

It should be noted that 2- and 3-aminofurans bearing an electron-withdrawing substituent are stable (but not well suited for Diels–Alder/cross-coupling applications).[3a, 4]

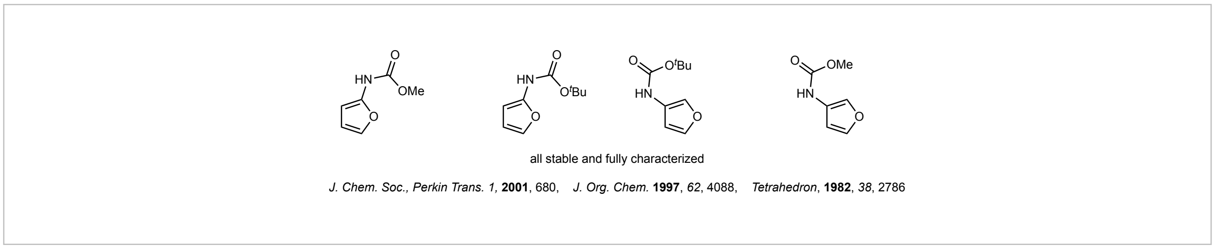

General Experimental

NMR Spectroscopy

^{1}H NMR spectra were recorded using either a Bruker 600 MHz Avance NEO console with a Magnex premium shielded 14.1 T magnet, Bruker 600 MHz Avance III-HD console with Oxford AS600 14.1 T magnet, Bruker 600 MHz Avance NEO console with Oxford AS600 14.1 T magnet, Bruker 900 MHz Avance NEO console with an Oxford 21.1 T pumped magnet, Bruker 400 MHz Avance III console with a Bruker Ascend 400 MHz magnet or a Bruker 600 MHz Avance NEO console with a Varian Premium Shielded 600 MHz magnet. Variable temperature ^{1}H NMR spectra were recorded using Bruker 400 MHz Avance III console with a Bruker Ascend 400 MHz magnet. ^{13}C NMR spectra were recorded using either a Bruker 600 MHz Avance NEO console with a Magnex premium shielded 14.1 T magnet at 150 MHz, Bruker 600 MHz Avance III-HD console with Oxford AS600 14.1 T magnet at 150 MHz, Bruker 600 MHz Avance NEO console with Oxford AS600 14.1 T magnet at 150 MHz, Bruker 900 MHz Avance NEO console with an Oxford 21.1 T pumped magnet at 225 MHz, Bruker Avance III console with a Bruker Ascend 400 MHz magnet at 100 MHz or a Bruker 600 MHz Avance NEO console with a Varian Premium Shielded 600 MHz magnet at 150 MHz. Residual solvent peaks were used as an internal reference for ^{1}H NMR spectra [$CDCl_3$ δ 7.26 ppm, $(CD_3)_2SO$ δ 2.50 ppm, CD_3OD δ 3.31 ppm, or CD_2Cl_2 δ 5.32 ppm] and ^{13}C NMR spectra [$CDCl_3$ δ 77.16 ppm, $(CD_3)_2SO$ δ 39.52 ppm, or CD_2Cl_2 δ 53.80 ppm]. ^{19}F NMR spectra were recorded using an Avance 400 MHz III HD console on a Bruker Ascend 400 MHz magnet at 377 MHz. ^{19}F NMR spectra were reported relative to the ^{19}F resonance of C_6F_6 [$CDCl_3$ δ −161.64 ppm, or CD_3OD δ −165.37 ppm].[5] Coupling constants (*J*) were quoted to the nearest 0.1 Hz. For ^{13}C NMR, coupling constants were included only in the case of coupling with ^{19}F nuclei. The following abbreviations (or combinations thereof) were used to describe ^{1}H NMR multiplicities: s = singlet, d = doublet, t = triplet, q = quartet, p = pentet, , h = heptet, m = multiplet, br = broad, app = apparent.

Infrared Spectroscopy

IR spectra were recorded neat on a Thermo Nicolet iS10 spectrometer or Thermo Nicolet 6700 spectrometer and are reported in wavenumbers (cm^{-1}).

Mass Spectrometry

High resolution mass spectrometry (HRMS) data were acquired via electrospray ionization (ESI) using either a Q-TOF mass spectrometer produced by Bruker model Impact II or Bruker Solarix XR 12 T FTICR MS.

Chromatography

Flash chromatography was performed with SiliaFlash® P60 silica, 0.040–0.063 mm grade. Analytical thin-layer chromatography was performed with commercial glass sheets coated with 0.25 mm silica gel (SiliaPlate™, silica gel 60, F254). Compounds were either visualized under UV light at 254 nm, or by dipping the plates in an aqueous potassium permanganate solution followed by heating, unless stated otherwise. All R_f values were measured to the nearest 0.1 cm.

Melting Points

Melting points were measured on a DigiMelt melting point apparatus, model SRS MPA161, and are uncorrected.

Optical Rotations

Optical rotations were recorded on an AUTOPOL III Automatic Polarimeter with a path length of 10 dm at the specified temperature.

Preparation of Phosphate Buffered Silica (pH = 7)

Prepared according to the method of Newton.[6] To a 5.00 L Erlenmeyer flask open to air and equipped with stir bar was added 3.80 L of deionized water. Sodium phosphate dibasic (114 g, 800 mmol) was slowly added with vigorous stirring, followed by additional deionized water to reach a total volume of 4000 mL (0.20 M). Once fully dissolved (ca. 10 minutes), 400 g of silica gel was slowly added. The pH of the mixture was measured (pH paper, range 1–13) to confirm the solution is neutral. If basic, the mixture was neutralized by either: (i) dropwise addition of phosphoric acid or (ii) addition of further silica gel (10.0 g portions). If acidic, additional phosphate buffer (0.2 M, prepared as above) was added. Once neutral, the mixture was filtered through a 2.00 L, 13.5 cm diameter sintered funnel (medium grit), then air was pulled through the funnel for 30 minutes. The phosphate buffered silica was transferred to a 2.00 L shallow glass dish and placed in an oven (105 °C) for three days, stirring every day to ensure even drying. The phosphate buffered silica was allowed to cool to ambient temperature and was sifted through a sieve into a container for long term storage. **Appearance**: White, free-flowing solid that partially adheres to glass when wet with solvent. **Note**: When using phosphate buffered silica, the addition of acidified sand to ensure a level silica gel line should be avoided. Instead, we recommend using additional phosphate buffered silica as a sand replacement.

Microwave

Reaction conditions invoking microwave irradiation were carried out in a CEM Discover 2.0 microwave synthesis system.

Experimental Procedures and Reagents

Commercially available chemicals were used as purchased or, where specified, purified by standard techniques. Solvent compositions are given in v/v. All reactions were carried out under an atmosphere of argon in flame-dried glassware unless otherwise indicated. All reactions underwent magnetic stirring employing an IKA plate, with heating facilitated by either an OptiTherm® heating mantle or a silicon oil bath when required. CH_2Cl_2, THF, Et_2O, DMF and toluene were purified by an Mbraun solvent purification system. All other solvents were used as purchased or, where specified, purified by standard techniques.

X-Ray Crystallography

X-ray data were measured at room temperature on a Bruker D8 Quest PHOTON 100 CMOS X-ray diffractometer system with Incoatec Microfocus Source (IµS) monochromated Mo K(alpha) radiation (lambda = 0.71073 Å, sealed tube) using phi and omega-scan technique. The data were integrated with the manufacturer's SAINT software and corrected for absorption effects using the Multi-Scan method (SADABS). The structure was solved and refined using the Bruker SHELXTL Software Package.[7] Non-hydrogen atoms were located from successive difference Fourier map calculations. In the final cycles of each refinement, all the non-hydrogen atoms were refined in anisotropic displacement parameters. All the hydrogen atom positions were calculated at geometrical positions and allowed to ride on the carbon to which they are bonded. Hydrogen atom temperature factors were fixed at n (n = 1.2 for PhH, CH, and CH_2 groups, n = 1.5 for CH_3 groups) times the isotropic temperature factors of the C–atoms to which they are bonded. Graphical representations of the X-ray structures were generated using CYLview20 (Legault, C. Y., Université de Sherbrooke, 2020, http://www.cylview.org).

General Comments on Compound Characterization

All new compounds were fully characterized. For compounds that have been previously prepared in the literature, if we modified their procedure we include a description of appearance, R_f, and ^1H NMR data, alongside our updated procedure.

Note: As a result of rotameric behavior about the triazene fragment, the pyrrolidinyl peaks tend to appear as very broad and (often) non-equivalent by both ^1H and ^{13}C NMR.

Experimental Procedures and Characterization Data

Synthesis of Azides

summary

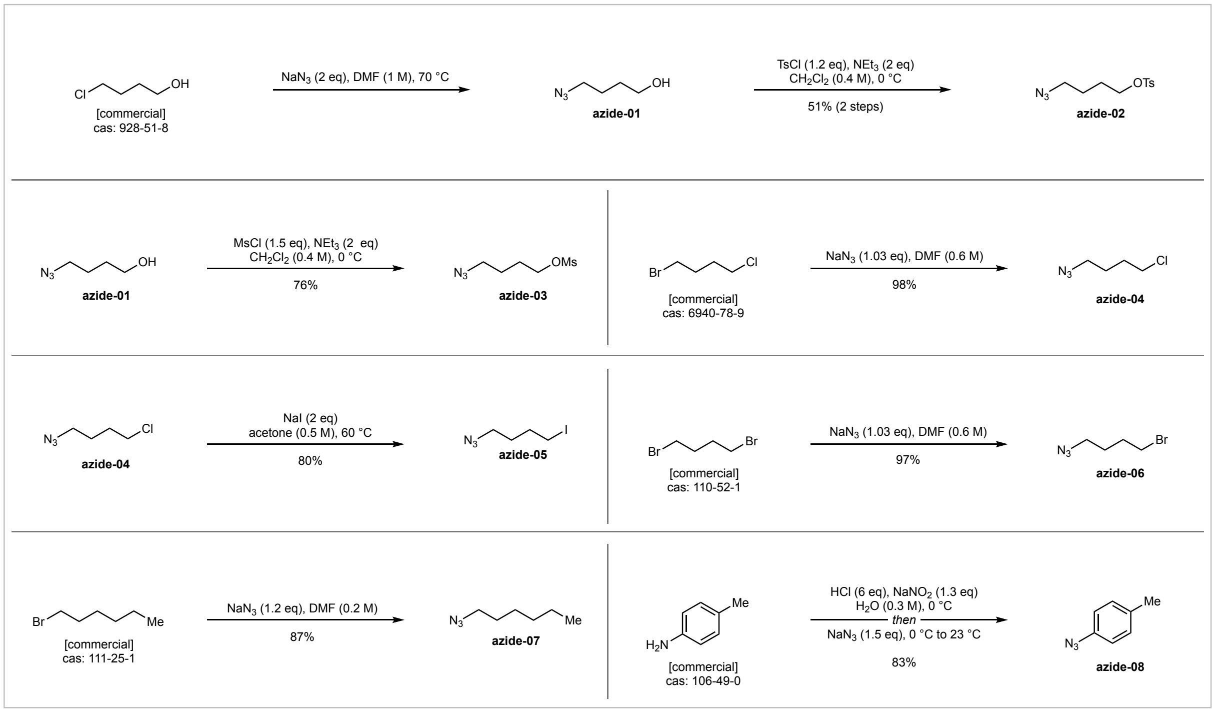

Caution: Organic azides are potentially explosive and toxic. Appropriate precautions should be taken during handling and storage (e.g., blast shield, avoid contact with metal spatulas, minimize exposure to heat, light, shock, and metal catalysts).

All NaN_3 waste was quenched via the following procedure: to an Erlenmeyer flask open to air and equipped with a stir bar was added NaN_3 waste (diluted with H_2O until less than 5% NaN_3 w/v), followed by an aqueous solution of $NaNO_2$ (×1.5 mass of NaN_3 dissolved in 30% w/v H_2O). Concentrated H_2SO_4 was slowly added (gas evolution) until a pH of ~2 was obtained (pH paper).

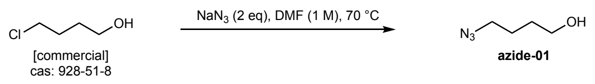

Prepared according to a modification of the procedure reported by Alcarranza and Alarcón-de-la-Lastra.[8] **Note:** 4-Chlorobutan-1-ol was employed as purchased (85% purity, containing approximately 10% THF and 5% HCl). To a flame-dried 1-neck 1000 mL round-bottom flask under argon and equipped with a stir bar was added 4-chlorobutan-1-ol (85% purity, 30.0 g, 235 mmol, 1.00 equiv), anhydrous DMF (235 mL, 1.0 M), and NaN_3 (30.5 g, 470 mmol, 2.00 equiv, added in one portion). Note: NaN_3 is poorly soluble in DMF and did not fully dissolve, even upon heating at 70 °C. The reaction was warmed on a heating mantle at 70 °C for 24 hours, by which time ^1H NMR analysis indicated the complete consumption of 4-chlorobutan-1-ol (accompanied by precipitation of NaCl). The reaction was cooled to ambient temperature, diluted with H_2O, and extracted with EtOAc (×3). The combined organic layers were washed with H_2O (×3), brine, dried over $MgSO_4$, filtered, and concentrated under reduced pressure (water bath at ambient temperature). The product was co-evaporated with hexane to remove trace water/EtOAc. This crude mixture was submitted to the next reaction without further purification. We recommend against putting **azide-01** under high vacuum (volatile).

Characterization data matched those reported by Alcarranza and Alarcón-de-la-Lastra.[8]

Appearance: pale-yellow oil;

R*f*: 0.17 (3:7 Et_2O:hexane);

^{1}H NMR (900 MHz, $CDCl_3$): δ 3.69 (t, *J* = 6.2 Hz, 2H), 3.33 (t, *J* = 6.7 Hz, 2H), 1.72 – 1.69 (m, 2H), 1.67 – 1.64 (m, 2H), 1.47 (br s, 1H) ppm.

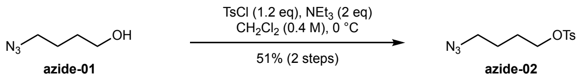

Prepared according to a modification of the procedure reported by Alcarranza and Alarcón-de-la-Lastra.[8] To a flame-dried 1-neck 1000 mL round-bottom flask under argon and equipped with a stir bar was added **azide-01** (27.0 g, 235 mmol, 1.00 equiv), anhydrous CH_2Cl_2 (588 mL, 0.40 M), and anhydrous NEt_3 (65.5 mL, 467 mmol, 2.00 equiv). The reaction flask was submerged in an ice/water bath and *p*-toluenesulfonyl chloride (53.7 g, 282 mmol, 1.20 equiv) was added portion-wise. The reaction was stirred for an additional 2 hours, by which time ^1H NMR analysis indicated the complete consumption of **azide-01**. The reaction was quenched with saturated aqueous sodium bicarbonate solution (approximately ×2 reaction volume). The ice/water bath was removed, and the reaction was sealed and stirred vigorously overnight to transfer remaining *p*-toluenesulfonyl chloride into the aqueous layer. The aqueous layer was extracted with CH_2Cl_2 (×2), and the combined organic layers were washed with H_2O, brine, dried over $MgSO_4$, filtered, and concentrated under reduced pressure. The crude residue was purified by flash column chromatography (ratio of silica to crude mass = 30:1, eluting with 1:4 EtOAc:hexane). Note: **azide-02** is a highly viscous oil. We found co-evaporation with hexane prior to placing under high vacuum was necessary for removal of trace EtOAc.

Characterization data matched those reported by Severin.[9]

Yield: 32.3 g, 120 mmol, 51% (2 steps);

Appearance: colorless oil;

R*f*: 0.10 (3:7 Et_2O:hexane);

^{1}H NMR (400 MHz, $CDCl_3$): δ 7.77 (d, *J* = 8.1 Hz, 2H), 7.34 (d, *J* = 8.2 Hz, 2H), 4.04 (t, *J* = 6.1 Hz, 2H), 3.24 (t, *J* = 6.6 Hz, 2H), 2.44 (s, 3H), 1.75 – 1.67 (m, 2H), 1.64 – 1.57 (m, 2H) ppm;

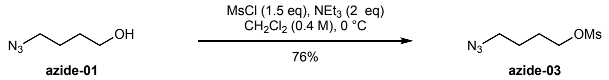

Prepared according to a modified procedure by Alcarranza and Alarcón-de-la-Lastra.[8] To a flame-dried 1-neck 1000 mL round-bottom flask under argon and equipped with a stir bar was added **azide-01** (0.540 g, 4.69 mmol, 1.00 equiv), anhydrous CH_2Cl_2 (15.6 mL, 0.30 M), and anhydrous NEt_3 (1.31 mL, 9.38 mmol, 2.00 equiv). The reaction flask was submerged in an ice/water bath, and methanesulfonyl chloride (0.806 g, 7.04 mmol, 1.50 equiv) was added dropwise. The reaction was stirred for an additional 2 hours, by which time ^{1}H NMR analysis indicated the complete consumption of **azide-01**. The reaction was quenched with H_2O (100 mL), the ice/water bath removed, and the aqueous layer was extracted with CH_2Cl_2 (×2). The combined organic layers were washed with H_2O, brine, dried over $MgSO_4$, filtered, and concentrated under reduced pressure. The crude residue was purified by flash column chromatography (ratio of silica to crude mass = 30:1, eluting with 1:4 EtOAc:hexane).

Characterization data matched those reported by Alcarranza and Alarcón-de-la-Lastra.[8]

Yield: 0.692 g, 3.58 mmol, 76%;

Appearance: colorless oil;

R*f*: 0.10 (3:7 Et_2O:hexane);

^{1}H NMR (400 MHz, $CDCl_3$): δ 4.27 (t, *J* = 6.2 Hz, 2H), 3.36 (t, *J* = 6.5 Hz, 2H), 3.02 (s, 3H), 1.89 – 1.82 (m, 2H), 1.76 – 1.69 (m, 2H) ppm.

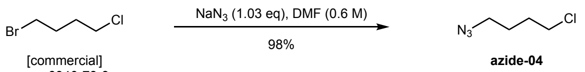

[commercial]
cas: 6940-78-9

Prepared according to a modified procedure by Severin.[9] To a 1-neck 100 mL round-bottom flask open to air and equipped with a stir bar was added 1-bromo-4-chlorobutane (5.00 g, 29.2 mmol, 1.00 equiv), DMF (48.6 mL, 0.60 M), and NaN_3 (1.99 g, 30.6 mmol, 1.03 equiv, added in one portion). Note: NaN_3 is poorly soluble in DMF and did not fully dissolve. The reaction was stirred for 15 hours, by which time ^{1}H NMR analysis indicated the complete consumption of 1-bromo-4-chlorobutane. The reaction was submerged in an ice/water bath, diluted with H_2O, and extracted with Et_2O (×3). The combined organic layers were washed with H_2O (×2), brine, dried over $MgSO_4$, filtered, and concentrated under reduced pressure. The crude product was submitted to the next reaction without further purification.

Characterization data matched those reported by Aubé.[10]

Yield: 3.82 g, 28.6 mmol, 98%;

Appearance: colorless oil;

R*f*: N/A (neither UV nor $KMnO_4$ active);

^{1}H NMR (400 MHz, $CDCl_3$): δ 3.57 (t, J = 6.4 Hz, 2H), 3.34 (t, J = 6.6 Hz, 2H), 1.91 – 1.84 (m, 2H), 1.80 – 1.73 (m, 2H) ppm.

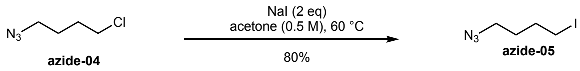

Prepared according to a modified procedure by Severin.[9] To a 1-neck 250 mL round-bottom flask equipped with a high-efficiency air condenser and stir bar was added **azide-04** (3.82 g, 28.6 mmol, 1.00 equiv), acetone (57.2 mL, 0.50 M), and NaI (8.57 g, 57.2 mmol, 2.00 equiv, added in one portion). The reaction was warmed on a heating mantle at 60 °C for 24 hours, by which time ^{1}H NMR analysis indicated the complete consumption of **azide-04** (accompanied by precipitation of NaCl). The reaction mixture was cooled to ambient temperature, filtered and concentrated under reduced pressure to remove the majority of acetone. The residue was diluted with H_2O and extracted with Et_2O (×3). The combined organic layers were washed with H_2O (×3), brine, dried over $MgSO_4$, filtered, and concentrated under reduced pressure. The crude residue was purified by flash column chromatography (ratio of silica to crude mass = 30:1, eluting with 1:20 Et_2O:hexane).

Characterization data matched those reported by Severin.[9]

Yield: 5.16 g, 22.9 mmol, 80%;

Appearance: pale red/brown oil;

R*f*: 0.32 (hexane);

^{1}H NMR (400 MHz, $CDCl_3$): δ 3.33 (t, J = 6.7 Hz, 2H), 3.21 (t, J = 6.8 Hz, 2H), 1.95 – 1.88 (m, J = 7.0 Hz, 2H), 1.75 – 1.68 (m, J = 6.8 Hz, 2H) ppm.

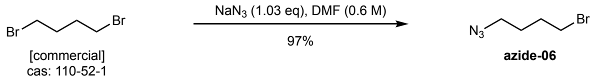

Prepared according to a modified procedure by Severin.[9] To a 1-neck 50 mL round-bottom flask open to air and equipped with a stir bar was added 1,4-dibromobutane (2.00 g, 9.26 mmol, 1.00 equiv), DMF (15.4 mL, 0.60 M), and NaN_3 (620 mg, 9.54 mmol, 1.03 equiv, added in one portion). Note: NaN_3 is poorly soluble in DMF and did not fully dissolve. The reaction was stirred for 16 hours, by which time ^{1}H NMR analysis indicated the complete consumption of 1,4-dibromobutane. The reaction was submerged in an ice/water bath and diluted with H_2O, warmed to ambient temperature and extracted with Et_2O (×3). The combined organic layers were washed with H_2O (×2), brine, dried over $MgSO_4$, filtered, and concentrated under reduced pressure. The crude product was submitted to the next reaction without further purification.

Characterization data matched those reported by Moellering.[11]

Yield: 1.59 g, 8.94 mmol, 97%;

Appearance: colorless oil;

R*f*: N/A (neither UV nor $KMnO_4$ active);

^{1}H NMR (400 MHz, $CDCl_3$): δ 3.44 (t, *J* = 6.4 Hz, 2H), 3.34 (t, *J* = 6.4 Hz, 2H), 2.04 (s, 1H) 1.99 – 1.92 (p, *J* = 6.8 Hz, 1H), 1.80 – 1.73 (p, *J* = 6.8 Hz, 1H), 1.68 (s, 1H) ppm.

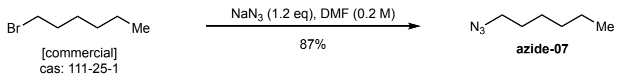

Prepared according to a modified procedure by Lautens.[12] To a 1-neck 100 mL round-bottom flask open to air and equipped with a stir bar was added 1-bromohexane (1.00 g, 6.05 mmol, 1.00 equiv), DMF (30.3 mL, 0.20 M), and NaN_3 (473 mg, 7.27 mmol, 1.20 equiv, added in one portion). Note: NaN_3 is poorly soluble in DMF and did not fully dissolve. The reaction was stirred for 16 hours, by which time ^{1}H NMR analysis indicated the complete consumption of 1-bromohexane. The reaction was submerged in an ice/water bath and diluted with H_2O, warmed to ambient temperature and extracted with Et_2O (×3). The combined organic layers were washed with H_2O (×2), brine, dried over $MgSO_4$, filtered, and concentrated under reduced pressure. The crude product was submitted to the next reaction without further purification.

Characterization data matched those reported by Qiu and Orita.[13]

Yield: 0.668 g, 5.25 mmol, 87%;

Appearance: colorless oil;

R*f*: N/A (neither UV nor $KMnO_4$ active);

^{1}H NMR (400 MHz, $CDCl_3$): δ 3.26 (t, *J* = 6.9 Hz, 2H), 1.63 – 1.56 (p, *J* = 7.1 Hz, 2H), 1.41 – 1.30 (m, 6H), 0.90 (t, *J* = 6.6 Hz, 3H) ppm.

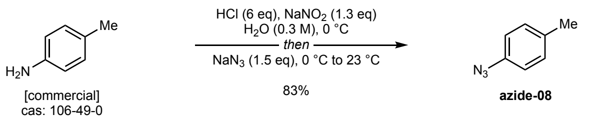

Prepared according to a modified procedure by Liang.[14] To a 3-neck 250 mL round-bottom flask open to air and equipped with a stir bar, thermometer, and two addition funnels was added *p*-toluidine (5.00 g, 46.7 mmol, 1.00 equiv), and aqueous HCl (6.0 M, 46.7 mL, 280 mmol, 6.00 equiv). The flask was submerged in an ice/water bath and a solution of $NaNO_2$ (4.19 g, 60.7 mmol, 1.30 equiv) in H_2O (75.8 mL, 0.80 M) was added dropwise via addition funnel, not allowing the internal reaction temperature to exceed 5 °C. The reaction was stirred for 30 minutes, then a solution of NaN_3 (4.55 g, 70.0 mmol, 1.50 equiv) in H_2O (70.0 mL, 1.0 M) was added dropwise via the second addition funnel, not allowing the internal reaction temperature to exceed 10 °C (white precipitate formed). The ice/water bath was removed, and the reaction was stirred for an additional 3 hours, by which time the precipitate had dissolved. The mixture was carefully neutralized with saturated aqueous sodium bicarbonate solution until pH of ~8 was obtained (pH paper). The reaction was extracted with Et_2O (×2), and the combined organic layers were

washed with H_2O, brine, dried over $MgSO_4$, filtered, and concentrated under reduced pressure. The crude product was submitted to the next reaction without further purification.

Characterization data matched those reported by Liang.[14]

Yield: 5.16 g, 38.7 mmol, 83%;

Appearance: brown oil;

R*f*: 0.44 (hexane);

^{1}H NMR (400 MHz, $CDCl_3$): δ 7.15 (d, *J* = 8.0 Hz, 2H), 6.92 (d, *J* = 8.3 Hz, 2H), 2.33 (s, 3H) ppm.

Synthesis of 2-Triazenyl Furans

Scouting Experiments

summary

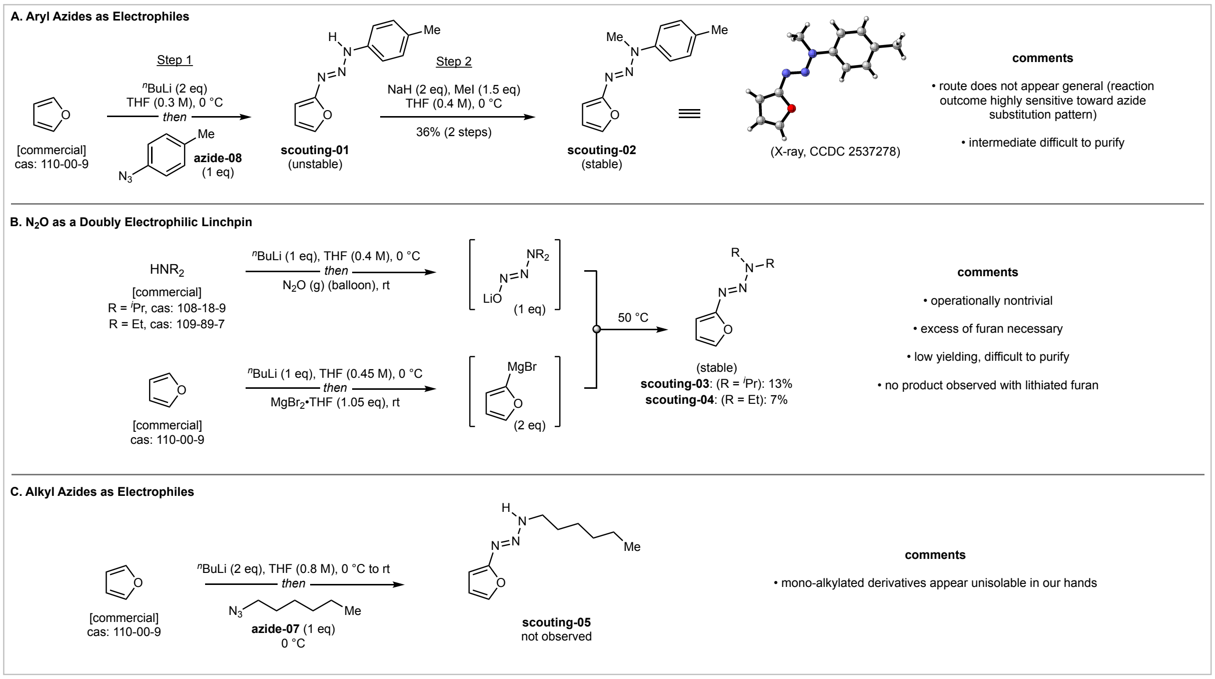

Note: As a result of rotameric behavior about the triazene moiety, significant broadening of peaks is often observed within their ^{1}H NMR spectra. With respect to their ^{13}C NMR spectra, often the two pyrrolidinyl methylene groups bound directly to nitrogen are non-equivalent and broad, whereas the other two methylene environments tend to appear as a single sharp resonance (however, there are several exceptions).

A. Aryl Azides as Electrophiles

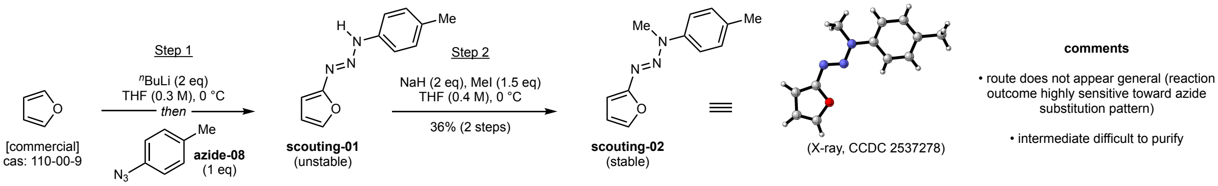

<u>Step 1</u>

Conducted according to a modification of a related procedure reported by Michejda.[15] To a flame-dried 100-mL one-neck round-bottom flask under argon and equipped with a stir bar was added furan (11.3 mmol, 0.820 mL, 3.00 equiv) and anhydrous THF (12.6 mL). The reaction flask was submerged in an ice/water bath, and freshly titrated[16] *n*-butyllithium (6.01 mL, 7.51 mmol, 1.25 M in hexane, 2.00 equiv) was added dropwise. The reaction was stirred in an ice/water bath for 30 minutes and then warmed to ambient temperature and stirred for an additional 30 minutes. The solution of 2-furyllithium (2.00 equiv, 0.500 M) was resubmerged in an ice/water bath and a solution of **azide-08** (500.0 mg, 3.76 mmol, 1.00 equiv) in anhydrous THF (3.76 mL) was added dropwise. The reaction was stirred

for an additional hour, quenched with a saturated aqueous solution of NH_4Cl and warmed to ambient temperature. The aqueous layer was extracted with Et_2O (×2), and the combined organic layers were washed with H_2O, brine, dried over $MgSO_4$, filtered, and concentrated under reduced pressure. **Note**: Due to the sensitive nature of **scouting-01** (decomposed during attempts at flash column chromatography), the crude product was subjected to the next step without further purification.

Appearance: Red-brown solid;

R*f*: N/A (decomposes on glass-backed silica TLC plates).

Step 2

Conducted according to a modification of a related procedure reported by Michejda.[15] A flame-dried 100-mL one-neck round-bottom flask containing crude **scouting-01** (729.0 mg) and equipped with a stir bar was evacuated and backfilled with argon (×3). Anhydrous THF (9.06 mL, 0.400 M) was added, the reaction flask was submerged in an ice/water bath, and NaH (290.0 mg, 7.25 mmol, 2.00 equiv) was added in two portions. The reaction mixture was stirred for one hour and then MeI (0.340 mL, 5.43 mmol, 1.50 equiv) was added. The reaction was warmed to ambient temperature and stirred for 16 hours, resubmerged in an ice/water bath, and quenched with a saturated aqueous solution of NH_4Cl. The aqueous layer was extracted with Et_2O (×2), and the combined organic layers were washed with H_2O (×2), brine, dried over $MgSO_4$, filtered, and concentrated under reduced pressure. The crude residue was purified by flash chromatography (neutral alumina, ratio alumina to crude mass = 50:1, eluting with 100% hexane to 1:25 Et_2O:hexane). A small quantity of pure **scouting-02** was crystallized via slow evaporation from hexane to yield yellow crystals suitable for X-ray analysis. **Note**: While **scouting-02** appears stable toward flash chromatography with silica as the stationary phase, other (unidentified) components in the crude mixture decomposed on silica, making purification challenging, hence the decision to use neutral alumina as the stationary phase.

Yield: 297 mg, 1.38 mmol, 36% (2 steps);

Appearance: Bright red solid;

R*f*: 0.38 (1:9 EtOAc:hexane);

M.p.: 51.3 – 53.9 °C;

^{1}H NMR (400 MHz, $CDCl_3$): δ 7.32 (d, *J* = 8.7 Hz, 3H), 7.19 (d, *J* = 6.8 Hz, 2H), 6.45 (s, 1H), 6.29 (s, 1H), 3.61 (s, 3H), 2.35 (s, 3H) ppm;

^{13}C NMR (100 MHz, $CDCl_3$): δ 159.1, 142.7, 139.1, 133.9, 129.9, 117.8, 112.2, 97.6, 33.3, 20.8 ppm;

IR: 2925, 2854, 2346, 1513, 1432, 1320, 1245, 733 cm^{-1};

HRMS (ESI): calculated for $[C_{12}H_{13}N_3O+H]^+$: 216.1131, found: 216.1132.

B. N_2O as a Doubly Electrophilic Linchpin

General Method

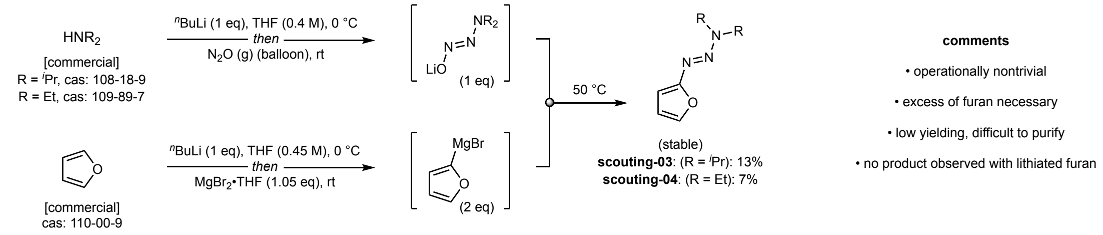

Lithium Dialkylamide Preparation

To a flame-dried 1-neck round-bottom flask under argon and equipped with a stir bar was added the appropriate dialkylamine (1.10 equiv) and anhydrous THF (0.450 M). The solution was cooled in a dry ice/acetone bath, and freshly titrated[16] *n*-butyllithium (1.00 equiv, solution in hexane) was added dropwise. The solution was submerged in an ice/water bath for 5 minutes, then returned to a dry ice/acetone bath for an additional 30 minutes. Prior to use, the lithium dialkylamide solution was titrated using menthol and 1,10-phenanthroline.[17]

Furyl Grignard Preparation

Prepared according to the method of Yamamoto.[18] To a flame-dried 2-neck round-bottom flask under argon and equipped with a stir bar was added furan (1.20 equiv) and anhydrous THF (1.50 M). The solution was cooled in an ice/water bath, and freshly titrated[16] *n*-butyllithium (1.00 equiv, solution in hexane) was added dropwise. The reaction was allowed to stir in an ice/water for 30 minutes, then warmed to ambient temperature and stirred for an additional 30 minutes. In a separate 2-neck round-bottom flask under argon equipped with a high-efficiency condenser and stir bar was added magnesium turnings (1.50 equiv). The apparatus was flame-dried under vacuum and backfilled with argon (×3) and then allowed to cool to ambient temperature. Anhydrous THF (1.00 M) was added, the flask was warmed on a heating mantle at 50 °C, and portions of dibromoethane (1.00 equiv) were added, maintaining a gentle reflux. Upon complete addition of dibromoethane, the solution was refluxed for 1 hour, in which magnesium dibromide crashed out as a white, crystalline solid. The suspension of magnesium dibromide (1.05 equiv relative to 2-furyllithium) was cooled in an ice/water bath, and the solution of 2-lithiofuran (1.00 equiv) was added dropwise. The solution was warmed to ambient temperature and allowed to stir for one hour. Prior to use, the furyl Grignard reagent was titrated with salicylaldehyde phenylhydrazone.[19]

Triazenyl Furan Synthesis

Conducted according to a modification of a related procedure reported by Severin.[20] To a flame-dried 1-neck round-bottom flask under argon and equipped with a stir bar was added a freshly prepared solution of lithium dialkylamide (ca. 0.450 M, 1.00 equiv). The argon inlet was replaced with a nitrous oxide balloon and N_2O was bubbled through the lithium dialkylamide solution at ambient temperature for 5 minutes. The reaction was sealed (N_2O balloon still

attached) and the reaction was stirred for four hours. The balloon was removed and the reaction mixture was placed under an argon atmosphere, and a solution of 2-furyl magnesium bromide (0.450 M, 2.00 equiv) was added in one portion. The reaction flask was equipped with a flame-dried high-efficiency condenser, and the reaction was warmed on a heating mantle at 50 °C for 16 hours. The reaction mixture was cooled to ambient temperature and passed through a pad of silica, eluting with diethyl ether. The filtrate was washed with H_2O, brine, dried over $MgSO_4$, filtered, and concentrated under reduced pressure. The crude product was purified by flash chromatography. Further purification details and characterization data are provided for each product below.

Scope

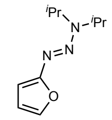

scouting-03

Synthesized from diisopropylamine. The crude product was purified by flash chromatography using phosphate buffered silica (pH = 7, ratio of buffered silica to crude mass = 50:1, eluting with 100% hexane to 1:20 Et_2O:hexane). **Note**: At the time of conducting this experiment, we did not appreciate that fully alkylated triazenyl furans were stable. Presumably this purification could have been conducted with regular silica.

Yield: 43 mg, 0.220 mmol, 13%;

Appearance: Yellow oil;

R*f*: 0.58 (1:9 Et_2O:hexane);

^{1}H NMR (400 MHz, $CDCl_3$): δ 7.21 (s, 1H), 6.38 (t, J = 2.5 Hz, 1H), 5.99 (d, J = 3.3 Hz, 1H), 5.18 (br s, 1H), 3.99 (br s, 1H), 1.30 (br s, 12H) ppm;

^{13}C NMR (100 MHz, $CDCl_3$): δ 160.9, 137.5, 111.7, 92.6, 49.2, 46.3, 23.7, 19.5 ppm;

IR: 2973, 2932, 1560, 1381, 1326, 1243, 1215, 1151, 713 cm^{-1};

HRMS (ESI): calculated for $[C_{10}H_{17}N_3O+H]^+$: 196.1444, found: 196.1441.

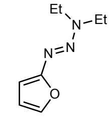

scouting-04

Synthesized from diethylamine. The crude product was purified via flash column chromatography (ratio of silica to crude mass = 50:1, eluting from 100% hexane to 1:4 Et_2O:hexane). **Note**: Characterization data did not match those previously reported by Kalkhambkar and Laali[1c] (see the **Prior Work** section earlier in the document for further details).

Yield: 54.0 mg, 0.323 mmol, 7%;

Appearance: Orange oil;

R*f*: 0.52 (1:4 Et_2O:hexane);

^{1}H NMR (400 MHz, $CDCl_3$): δ 7.19 (s, 1H), 6.37 (s, 1H), 6.03 (d, *J* = 3.4 Hz, 1H), 3.72 (q, *J* = 7.2 Hz, 4H), 1.24 (s, 6H) ppm;

^{13}C NMR (100 MHz, $CDCl_3$): δ 159.5, 137.7, 111.8, 95.0, 48.9, 41.2, 14.2, 11.6 ppm;

IR: 3116, 2976, 2935, 2365, 1572, 1409, 1370, 1350, 1248 cm^{-1};

HRMS (ESI): calculated for $[C_8H_{13}N_3O+H]^+$: 168.1131, found: 168.1129.

C. Alkyl Azides as Electrophiles

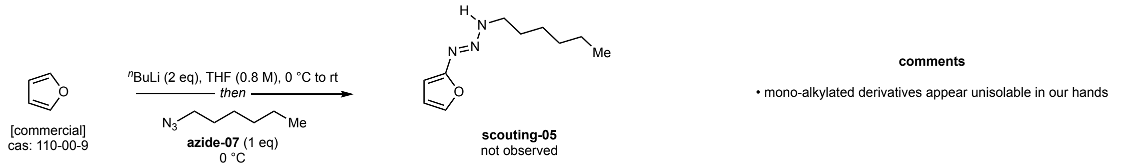

To a flame-dried 1-neck round-bottom flask under argon and equipped with a stir bar was added furan (0.860 mL, 11.8 mmol, 2.20 equiv) and anhydrous THF (7.13 mL). The reaction flask was submerged in an ice/water bath, and freshly titrated[16] *n*-butyllithium (1.96 M in hexane, 5.46 mL, 2.00 equiv) was added dropwise. The reaction was stirred for 30 minutes, then warmed to ambient temperature and stirred for an additional 30 minutes, before being resubmerged in an ice/water bath. A solution of **azide-07** (680.0 mg, 5.35 mmol, 1.00 equiv) in anhydrous THF (17.8 mL) was added dropwise, the reaction was stirred for one hour and then quenched with a saturated aqueous solution of NH_4Cl. The reaction mixture was warmed to ambient temperature and extracted with Et_2O (×2). The combined organic layers were washed with H_2O, brine, dried over $MgSO_4$, filtered, and concentrated under reduced pressure. **Note**: While full consumption of azide was observed, no product was isolated. We speculate that the high instability of the desired product precludes its isolation. Attempts to observe it by ^{1}H NMR aliquots of the reaction mixture were also unsuccessful.

One-Pot Furan Triazenylation/Intramolecular Alkylation

Triazenylation Precursors

summary

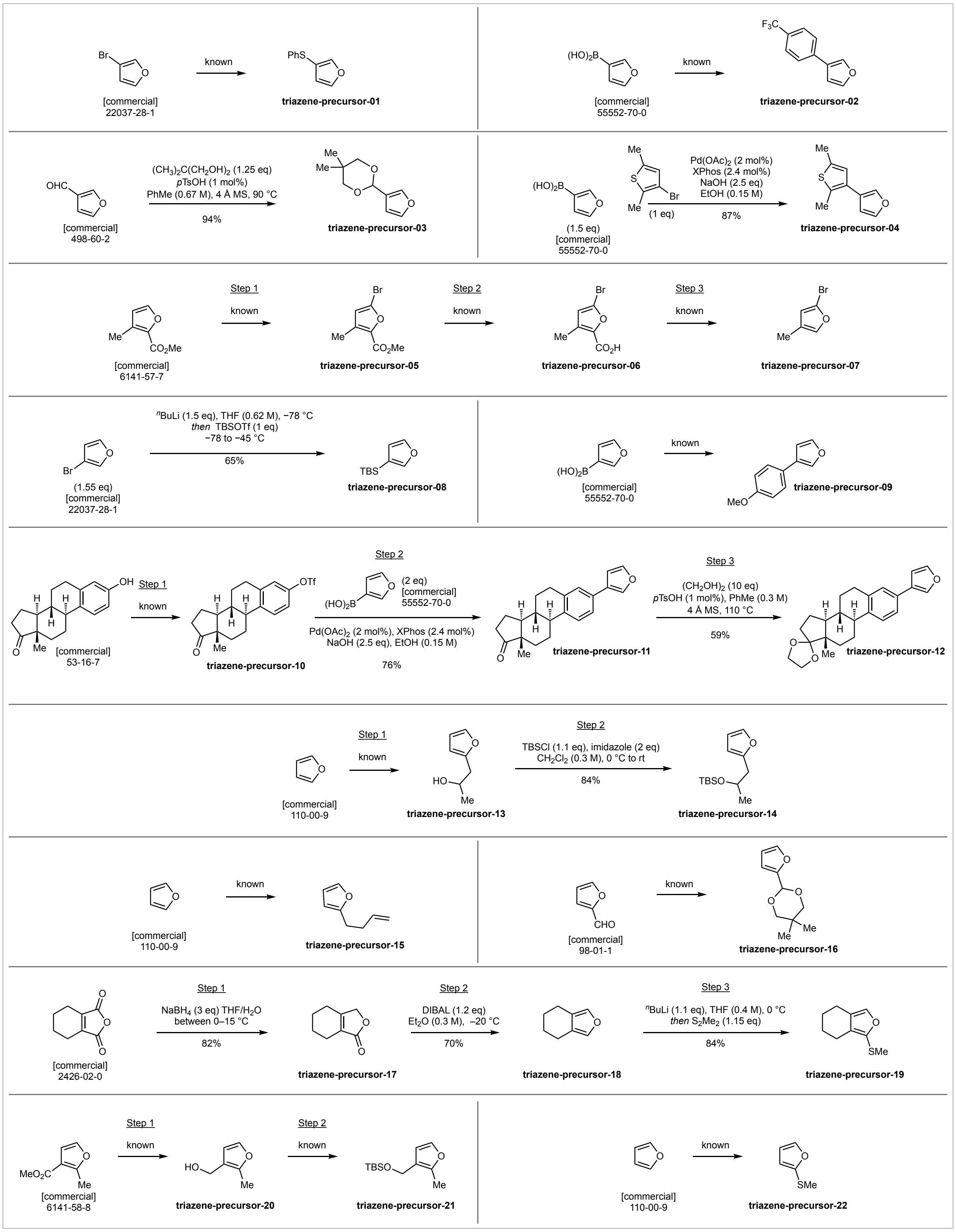

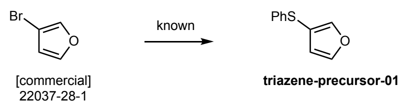

Note: 3-Bromofuran was distilled immediately prior to use using a flame-dried short path distillation apparatus under argon (flask containing 3-bromofuran sitting in a heating mantle set to 120 °C).

Reaction conducted according to the method of Tanino.[21]

Characterization data matched those reported by Saeys and Hierso.[22]

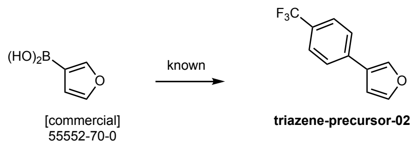

Reaction conducted according to the method of Glorius.[23]

Characterization data matched those reported by Glorius.[23]

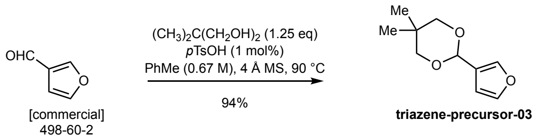

Reaction conducted according to a modification of the procedure reported by Lee.[24] To a flame-dried 1-neck 25 mL round-bottom flask under argon and equipped with a stir bar and an addition funnel charged with flame-dried 4 Å molecular sieves was added 3-furaldehyde (1.00 g, 10.4 mmol, 1.00 equiv), 2,2-dimethylpropane-1,3-diol (1.36 g, 13.0 mmol, 1.25 equiv), *p*-toluenesulfonic acid (19.8 mg, 0.104 mmol, 0.01 equiv), and anhydrous PhMe (15.1 mL, 0.670 M). The biphasic mixture was warmed to 90 °C on a heating mantle for three hours, then cooled to ambient temperature and concentrated under reduced pressure. The crude residue was purified by flash column chromatography on phosphate buffered silica (pH = 7, ratio of silica to crude mass = 30:1, eluting with 1:9 EtOAc:hexane).

Yield: 1.72 g, 9.41 mmol, 94%;

Appearance: Colorless oil;

$\mathbf{R_f}$: 0.50 (1:2 EtOAc:hexane; **note**: not UV active, stained with $KMnO_4$);

^{1}H NMR (400 MHz, $CDCl_3$): δ 7.53 (s, 1H), 7.39 (s, 1H), 6.49 (s, 1H), 5.42 (s, 1H), 3.71 (d, J = 10.9 Hz, 2H), 3.59 (d, J = 10.9 Hz, 2H), 1.26 (s, 3H), 0.78 (s, 3H) ppm;

^{13}C NMR (100 MHz, $CDCl_3$): δ 143.2, 140.4, 124.6, 108.4, 97.0, 77.5, 30.4, 23.1, 22.0 ppm;

IR: 2954, 2827, 1504, 1394, 1215, 1105, 919, 837 cm^{-1};

HRMS (ESI): calculated for $[C_{10}H_{14}O_3+H]^+$: 183.1016, found: 183.1012.

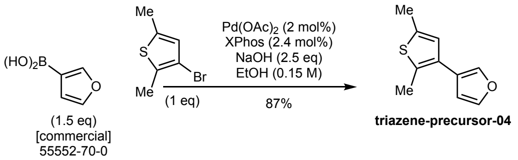

To a 3-neck 100 mL round-bottom flask open to air and equipped with a stir bar was added 3-bromo-2,5-dimethylthiophene[25] (900 mg, 4.71 mmol, 1.00 equiv), 3-furanylboronic acid (791 mg, 7.07 mmol, 1.50 equiv), and XPhos (53.9 mg, 0.113 mmol, 0.024 equiv). The reaction flask was evacuated and backfilled with argon (×3) and then EtOH (31.4 mL, 0.150 M) was added. Argon was bubbled through the stirring mixture for approximately 10 minutes, then $Pd(OAc)_2$ (21.2 mg, 0.0942 mmol, 0.02 equiv) and NaOH (471 mg, 11.8 mmol, 2.50 equiv) were added. The reaction was stirred at ambient temperature for 16 hours, then filtered over a plug of silica (eluting with EtOH). The filtrate was concentrated under reduced pressure, and the crude residue was purified by flash column chromatography on phosphate buffered silica (pH = 7, ratio of silica to crude mass = 30:1, eluting with hexane).

Yield: 730 mg, 4.10 mmol, 87%;

Appearance: Colorless to yellow oil;

R*f*: 0.22 (hexane);

^{1}H NMR (400 MHz, $CDCl_3$): δ 7.53 (s, 1H), 7.45 (s, 1H), 6.70 (s, 1H), 6.56 (s, 1H), 2.43 (d, J = 5.1 Hz, 6H) ppm;

^{13}C NMR (100 MHz, $CDCl_3$): δ 142.9, 139.2, 135.8, 131.4, 128.8, 126.4, 121.6, 110.5, 15.2, 14.6 ppm;

IR: 2918, 2858, 1767, 1446, 1151, 1037, 873 cm^{-1};

HRMS (ESI): calculated for $[C_{10}H_{10}OS+H]^+$: 179.0525, found: 179.0518.

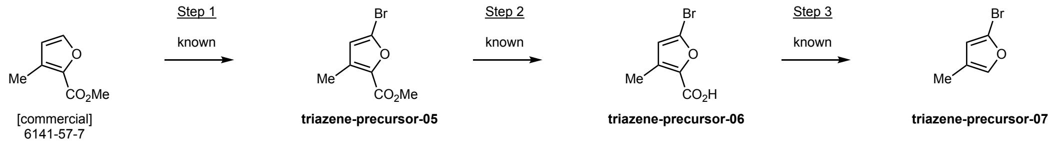

Steps 1–3

Reaction conducted according to the method of Li.[26]

Characterization data matched those reported by Li.[26]

Note: In our hands, **triazene-precursor-07** spontaneously and violently decomposed if left for several hours at ambient temperature without protection from light. If stored under a blanket of argon in the freezer (–20 °C) with K_2CO_3 as stabilizer, no decomposition was observed over several months.

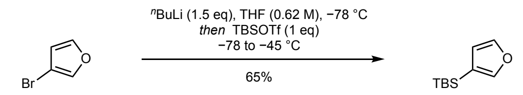

(1.55 eq)
[commercial]
22037-28-1

triazene-precursor-08

Note: 3-Bromofuran was distilled immediately prior to use using a flame-dried short path distillation apparatus under argon (flask containing 3-bromofuran sitting in a heating mantle set to 120 °C).

To a flame-dried 3-neck 100 mL round-bottom flask under argon and equipped with a stir bar was added 3-bromofuran (2.50 g, 17.0 mmol, 1.55 equiv) and anhydrous THF (27.4 mL, 0.620 M). The reaction flask was submerged in a dry ice/acetone bath, and freshly titrated[16] *n*-butyllithium (7.04 mL, 16.5 mmol, 2.34 M in hexane, 1.50 equiv) was added dropwise over approximately 20 minutes. The reaction was stirred for one hour and then TBSOTf (2.36 mL, 11.0 mmol, 1.00 equiv) was added dropwise over 20 minutes. The reaction was stirred for two hours, then transferred to an immersion chiller cooled bath (–45 °C) and stirred for 12 hours. The reaction was quenched with a saturated aqueous solution of NH_4Cl and warmed to ambient temperature. The mixture was extracted with Et_2O (×2), and the combined organic layers were washed with H_2O, brine, dried over $MgSO_4$, filtered, and concentrated under reduced pressure (water bath kept at ambient temperature as **triazene-precursor-08** is slightly volatile). The crude residue was purified by flash column chromatography on silica (ratio of silica to crude mass = 30:1, eluting with hexane), yielding an inseparable mixture of **triazene-precursor-08** and its 2-substituted analog (ca. 7:1) that were submitted to the subsequent reaction.

Yield: 1.30 g, 7.11 mmol, 65%;

Appearance: Colorless oil;

R*f*: 0.56 (hexane, **Note**: not UV active, stained with $KMnO_4$);

^{1}H NMR (400 MHz, $CDCl_3$, only major isomer indicated): δ 7.50 (s, 1H), 7.35 (s, 1H), 6.38 (s, 1H), 0.89 (s, 9H), 0.18 (s, 6H) ppm;

^{13}C NMR (150 MHz, $CDCl_3$, only major isomer indicated): δ 147.9, 142.8, 115.9, 114.0, 26.5, 16.7, − 5.4 ppm;

IR: 2928, 2856, 1746, 1471, 1251, 1057, 773 cm^{-1};

HRMS (ESI): calculated for $[C_{10}H_{18}OSi+H]^+$: 183.1200, found: 183.1195.

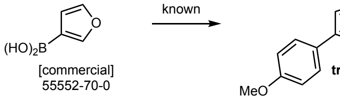

[commercial]
55552-70-0

triazene-precursor-09

Reaction conducted according to the method of Glorius.[23]

Characterization data matched those reported by Glorius.[23]

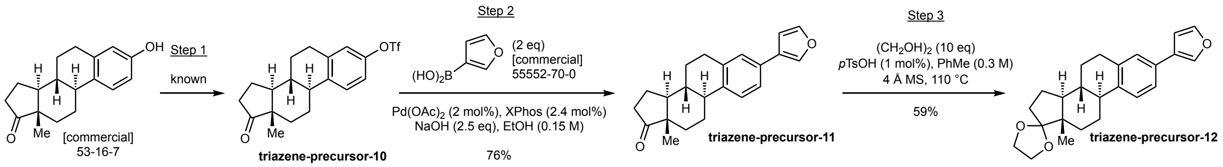

Step 1

Reaction conducted according to the method of Chattopadhyay.[27]

Characterization data matched those reported by Noël.[28]

Step 2

To a 3-neck 25 mL round-bottom flask open to air and equipped with a stir bar was added **triazene-precursor-10** (570 mg, 1.42 mmol, 1.00 equiv), 3-furanylboronic acid (317 mg, 2.83 mmol, 2.00 equiv), and XPhos (16.2 mg, 0.0340 mmol, 0.024 equiv). The reaction flask was evacuated and backfilled with argon (×3) and then EtOH (9.44 mL, 0.150 M) was added. Argon was bubbled through the stirring mixture for approximately 10 minutes, the balloon was removed, then $Pd(OAc)_2$ (6.36 mg, 0.0283 mmol, 0.02 equiv) and NaOH (142 mg, 3.54 mmol, 2.50 equiv) were added. The reaction was stirred at ambient temperature for 16 hours, then filtered over a plug of silica (eluting with EtOH). The filtrate was concentrated under reduced pressure, and the crude residue was purified by flash column chromatography on silica (ratio of silica to crude mass = 30:1, eluting with 1:5 EtOAc:hexane).

Yield: 345 mg, 1.08 mmol, 76%;

Appearance: Yellow solid;

R*f*: 0.38 (1:5 EtOAc:hexane);

^{1}H NMR (400 MHz, $CDCl_3$): δ 7.70 (s, 1H), 7.46 (s, 1H), 7.32 – 7.27 (m, 2H), 7.23 (s, 1H), 6.68 (s, 1H), 2.95 (dd, *J* = 8.9, 4.2 Hz, 2H), 2.52 (dd, *J* = 18.6, 8.6 Hz, 1H), 2.47 – 2.41 (m, 1H), 2.33 (t, *J* = 10.7 Hz, 1H), 2.20 – 1.95 (m, 4H), 1.71 – 1.54 (m, 4H), 1.52 – 1.40 (m, 2H), 0.92 (s, 3H) ppm;

^{13}C NMR (100 MHz, $CDCl_3$): δ 221.1, 143.7, 138.8, 138.4, 137.0, 130.1, 126.6, 126.4, 126.0, 123.6, 109.0, 50.6, 48.1, 44.5, 38.3, 36.0, 31.7, 29.6, 26.6, 25.9, 21.7, 14.0 ppm;

IR: 2929, 2858, 1736, 1513, 1261, 1050, 874 cm^{-1};

HRMS (ESI): calculated for $[C_{22}H_{24}O_2+H]^+$: 321.1849, found: 321.1843.

Step 3

Reaction conducted according to a modification of a procedure reported by Stoltz.[29] To a flame-dried 1-neck 10 mL round-bottom flask under argon and equipped with a stir bar and an addition funnel containing flame-dried 4 Å molecular sieves was added **triazene-precursor-11** (300 mg, 0.936 mmol, 1.00 equiv), ethylene glycol (0.524 mL, 9.36 mmol, 10.0 equiv), *p*-toluenesulfonic acid (1.78 mg, 0.00936 mmol, 0.01 equiv), and anhydrous PhMe (3.12 mL, 0.300 M). The biphasic mixture was warmed to 110 °C on a heating mantle for 16 hours, then cooled to ambient

temperature and concentrated under reduced pressure. The crude residue was purified by flash column chromatography (ratio of silica to crude mass = 50:1, eluting with 5:95 EtOAc:hexane).

Yield: 202 mg, 0.554 mmol, 59%;

Appearance: White solid;

R*f*: 0.35 (1:5 EtOAc:hexane);

^{1}H NMR (400 MHz, $CDCl_3$): δ 7.68 (s, 1H), 7.44 (s, 1H), 7.30 (app d, *J* = 10.1 Hz, 1H), 7.19 (s, 1H), 6.66 (s, 1H), 4.02 – 3.81 (m, 5H), 2.88 (s, 2H), 2.40 – 2.24 (m, 2H), 2.03 (t, *J* = 12.8 Hz, 1H), 1.96 – 1.89 (m, 1H), 1.87 – 1.73 (m, 3H), 1.70 – 1.55 (m, 2H), 1.50 – 1.32 (m, 4H), 0.88 (s, 3H) ppm;

^{13}C NMR (100 MHz, $CDCl_3$): δ 143.6, 139.5, 138.4, 137.3, 129.8, 126.6, 126.5, 126.0, 123.4, 119.6, 109.1, 65.4, 64.7, 49.6, 46.3, 44.2, 39.0, 34.4, 30.9, 29.7, 27.1, 26.1, 22.5, 14.5 ppm;

IR: 2934, 2868, 1737, 1455, 1308, 1160, 1105, 874 cm^{-1};

HRMS (ESI): calculated for $[C_{24}H_{28}O_3+H]^+$: 365.2111, found: 365.2106.

[α]D^{20}: +30.7 (c = 0.38, $CHCl_3$).

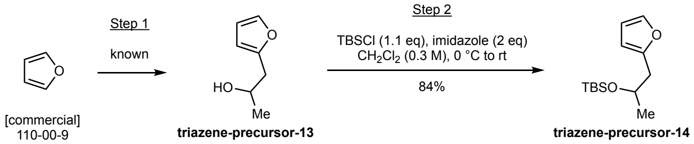

Step 1

Reaction conducted according to the method of Markó.[30]

Characterization data matched those reported by Markó.[30]

Step 2

To a flame-dried 1-neck 100 mL round-bottom flask under argon and equipped with a stir bar was added **triazene-precursor-13** (1.15 g, 9.12 mmol, 1.00 equiv) anhydrous CH_2Cl_2 (30.4 mL, 0.300 M), imidazole (1.24 g, 18.3 mmol, 2.00 equiv), and the reaction flask was submerged in an ice/water bath. TBSCl (1.51 g, 10.0 mmol, 1.10 equiv) was added in one portion and the reaction was warmed to ambient temperature and stirred for 16 hours, by which time TLC analysis indicated the complete consumption of **triazene-precursor-13.** The reaction was quenched with H_2O, and the aqueous layer was extracted with CH_2Cl_2. The combined organic layers were washed with water (×2), brine, dried over $MgSO_4$, filtered, and concentrated under reduced pressure. The crude residue was purified by flash column chromatography (ratio of silica to crude mass = 30:1, eluting with hexane).

Yield: 1.83 g, 7.62 mmol, 84%;

Appearance: Colorless oil;

R*f*: 0.20 (hexane);

^{1}H NMR (900 MHz, $CDCl_3$): δ 7.30 (s, 1H), 6.27 (s, 1H), 6.03 (d, J = 3.1 Hz, 1H), 4.08 (h, J = 6.1 Hz, 1H), 2.76 (dd, J = 14.6, 6.9 Hz, 1H), 2.68 (dd, J = 14.7, 5.8 Hz, 1H), 1.16 (d, J = 6.1 Hz, 3H), 0.85 (s, 9H), −0.02 (s, 3H), −0.08 (s, 3H) ppm;

^{13}C NMR (100 MHz, $CDCl_3$): δ 153.7, 141.0, 110.4, 106.9, 67.8, 38.7, 26.0, 23.9, 18.2, −4.8, −5.0 ppm;

IR: 2955, 2857, 2360, 1256, 1087, 999, 836, 775 cm^{-1};

HRMS (ESI): calculated for $[C_{13}H_{24}O_2Si+Na]^+$: 263.1438, found: 263.1429.

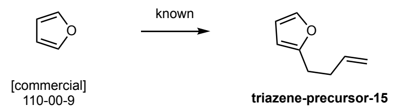

Reaction conducted according to the method of Vassilikogiannakis.[31]

Characterization data matched those reported by Vassilikogiannakis.[31]

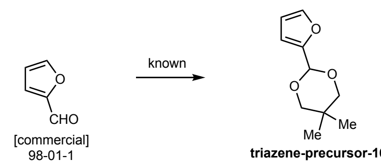

Reaction conducted according to the method of Lee.[24]

Characterization data matched those reported by Lee.[24]

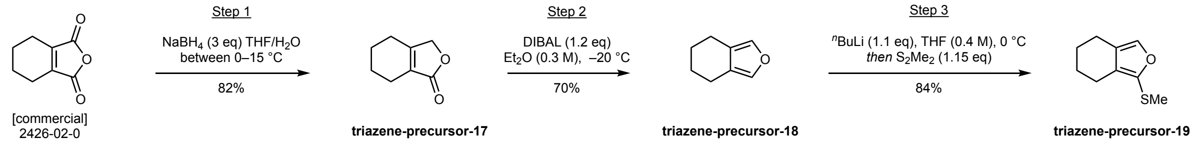

Step 1

Reaction conducted according to a modification of the procedure reported by Shenvi.[32] To a 500 mL 3-neck round-bottom flask open to air and equipped with a stir bar and thermometer was added 4,5,6,7-tetrahydroisobenzofuran-1,3-dione (10.0 g, 65.7 mmol, 1.00 equiv) and THF (316 mL, 0.208 M). The reaction flask was submerged in an ice/water bath and H_2O (13.1 mL, 5.00 M) was added. $NaBH_4$ (7.46 g, 197 mmol, 3.00 equiv) was then added in portions to prevent internal reaction temperature exceeding 15 °C (ca. 1 g every 5 minutes). The reaction was stirred for two hours, quenched with 2 M aqueous HCl solution, and then warmed to ambient temperature. The reaction was extracted with EtOAc (×3), and the combined organic layers were washed with water (×2), brine, dried over $MgSO_4$, filtered, and concentrated under reduced pressure.

Characterization data matched those reported by Hiemstra.[33]

Yield: 7.46 g, 54.0 mmol, 82%;

Appearance: White/grey solid;

R*f*: 0.30 (2:5 EtOAc:hexane);

^{1}H NMR (400 MHz, $CDCl_3$): δ 4.72 – 4.64 (m, 2H), 2.34 – 2.28 (m, 2H), 2.25 – 2.19 (m, 2H), 1.82 – 1.69 (m, 4H) ppm.

Step 2

Reaction conducted according to a modification of the procedure reported by Chui.[34] To a flame-dried 3-neck 1000 mL round-bottom flask under argon and equipped with a stir bar and thermometer was added **triazene-precursor-17** (11.5 g, 83.3 mmol, 1.00 equiv) and anhydrous Et_2O (278 mL, 0.300 M). The reaction flask was submerged in a dry ice/aqueous $CaCl_2$ bath (–20 °C), and DIBAL (100 mL, 1.00 M in hexane, 1.20 equiv) was added dropwise over approximately 30 minutes. The reaction was stirred for two hours and then quenched with 2 M aqueous HCl solution (ca. ×1 reaction volume). The reaction was warmed to ambient temperature and stirred vigorously overnight. The aqueous layer was extracted with Et_2O (×2), and the combined organic layers were washed with water (×2), brine, dried over $MgSO_4$, filtered, and concentrated under reduced pressure (water bath kept at ambient temperature as **triazene-precursor-18** is slightly volatile). The crude residue was passed through a plug of silica (ratio of silica to crude mass = 10:1, eluting with hexane), and concentrated under reduced pressure at ambient temperature.

Characterization data matched those reported by Takadoi.[35]

Yield: 7.10 g, 58.1 mmol, 70%;

Appearance: Colorless oil;

R*f*: 0.43 (hexane; **Note**: not UV active, stained with vanillin);

^{1}H NMR (400 MHz, $CDCl_3$): δ 7.14 (s, 2H), 2.55 (s, 4H), 1.70 (s, 4H) ppm.

Step 3

To a flame-dried 1-neck 25 mL round-bottom flask under argon and equipped with a stir bar was added **triazene-precursor-18** (400 mg, 3.27 mmol, 1.00 equiv) and anhydrous THF (8.19 mL, 0.400 M). The reaction flask was submerged in an ice/water bath, and freshly titrated[16] *n*-butyllithium (1.65 mL, 2.19 M in hexane, 1.10 equiv) was added dropwise. The reaction was stirred for two hours and then dimethyl disulfide (0.337 mL, 3.76 mmol, 1.15 equiv) was added dropwise, immediately forming a white precipitate. The reaction was stirred for one hour, by which time TLC analysis indicated the complete consumption of **triazene-precursor-18**. The reaction was quenched by addition of a saturated aqueous solution of NH_4Cl and then warmed to ambient temperature. The reaction mixture was extracted with Et_2O (×2), and the combined organic layers were washed with water, brine, dried over $MgSO_4$, filtered, and concentrated under reduced pressure. The crude residue was purified by flash column chromatography on silica (ratio of silica to crude mass = 20:1, eluting with hexane).

Yield: 463 mg, 2.75 mmol, 84%;

Appearance: Pale yellow oil;

R*f*: 0.32 (hexane);

^{1}H NMR (400 MHz, $CDCl_3$): δ 7.20 (s, 1H), 2.51 (q, *J* = 5.6 Hz, 4H), 2.30 (s, 3H), 1.74 – 1.64 (m, 4H) ppm;

^{13}C NMR (100 MHz, $CDCl_3$): δ 140.5, 139.6, 126.9, 123.3, 23.2, 23.1, 21.2, 20.3, 18.7 ppm;

IR: 2923, 2854, 1760, 1437, 1311, 1108, 984, 847 cm^{-1};

HRMS (ESI): calculated for $[C_9H_{12}OS+H]^+$: 169.0682, found: 169.0678.

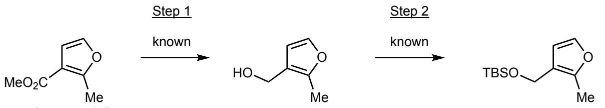

[commercial] 6141-58-8 **triazene-precursor-20** **triazene-precursor-21**

Step 1

Reaction conducted according to the method of Greaney.[36]

Characterization data matched those reported by Greaney.[36]

Step 2

Reaction conducted according to the method of Sutton.[37]

Characterization data matched those reported by Jung.[38]

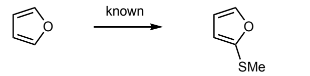

[commercial] 110-00-9 **triazene-precursor-22**

Reaction conducted according to the method of Holland.[39]

Characterization data matched those reported by Holland.[39]

One-Pot Triazenylation/Intramolecular Alkylation

Select Optimization

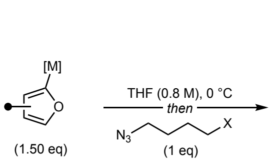

deviation from optimized conditions	yield	conversion (NMR)
none	64%	100%
azide leaving group		
OMs	7% (NMR)	72%
I	48%	100%
Br	33% (NMR)	100%
Cl	traces (NMR)	100%
nucleophile		
2-furyllithium (1.25 eq, from *n*-butyllithium)	60%	90%
2-furyllithium (1.50 eq, from lithium diisopropylamide)	64% (NMR)	100%
2-furylmagnesium bromide (1.50 equiv)	17%	100%

General Method

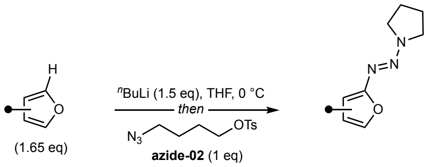

Note: Concentrations, stoichiometry and yields, are calculated relative to **azide-02**.

Reaction conducted according to a modification of the procedure reported by Severin.[9] To a flame-dried 3-neck round-bottom flask under argon and equipped with a stir bar was added the appropriate furan (1.65 equiv) and anhydrous THF (0.8 M). The solution was submerged in an ice/water bath, and freshly titrated[16] *n*-butyllithium (solution in hexane, 1.50 equiv) was added dropwise, and the reaction was allowed to stir in an ice/water bath for 2 hours. A solution of **azide-02** in anhydrous THF (0.8 M, 1.00 equiv) was added dropwise, causing the reaction to turn dark brown. The reaction was allowed to stir for an additional hour before being quenched with a saturated aqueous solution of NH_4Cl. The mixture was warmed to ambient temperature and extracted with Et_2O (×2). The combined organic layers were washed with water, brine, dried over $MgSO_4$, filtered, and concentrated under reduced pressure. Further purification details and characterization data are provided for each product below.

Scope

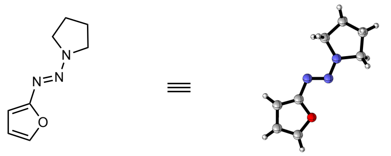

2-triazenyl-furan-01 (or **1**), 58% (g scale) (X-ray, CCDC 2537282)

Prepared from furan (cas 110-00-9) according to the general procedure. Purified by flash column chromatography (ratio of silica to crude mass= 30:1, eluting with 1:9 EtOAc:hexane). A small quantity of pure **1** was crystallized via slow evaporation from hexane to yield yellow crystals suitable for X-ray analysis.

Yield: 1.77 g, 10.7 mmol, 58%;

Appearance: Pale-orange solid;

R*f*: 0.22 (1:9 EtOAc:hexane);

M.p.: 57.8 – 61.2 °C;

^{1}H NMR (400 MHz, $CDCl_3$): δ 7.19 (s, 1H), 6.38 (s, 1H), 6.06 (s, 1H), 3.78 (s, 4H), 2.03 (s, 4H) ppm;

^{13}C NMR (100 MHz, $CDCl_3$): δ 159.5, 137.8, 111.9, 95.7, 51.3, 46.6, 23.9 ppm;

IR: 2973, 2932, 1560, 1362, 1243, 1214, 713 cm^{-1};

HRMS (ESI): calculated for $[C_8H_{11}N_3O+H]^+$: 166.0975, found: 166.0971.

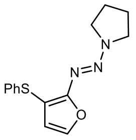

2-triazenyl-furan-02, 25%

Prepared from **triazene-precursor-01** according to the general procedure. Purified by flash column chromatography using phosphate buffered silica (pH = 7, ratio of silica to crude mass = 50:1, eluting with hexane to 5:95 Et_2O:hexane).

Yield: 449 mg, 1.64 mmol, 25%;

Appearance: Yellow solid;

R*f*: 0.33 (1:5 EtOAc:hexane);

^{1}H NMR (600 MHz, $CDCl_3$): δ 7.25 – 7.21 (m, 5H), 7.12 (t, J = 6.6 Hz, 1H), 6.38 (d, J = 2.1 Hz, 1H), 3.92 (br s, 2H), 3.63 (br s, 2H), 2.01 (br s, 4H) ppm;

^{13}C NMR (100 MHz, $CDCl_3$): δ 158.7, 137.9, 137.7, 128.8, 127.7, 125.5, 116.4, 100.2, 51.5, 46.9, 24.0, 23.5 ppm;

IR: 3143, 3055, 2973, 2872, 1555, 1477, 1398, 1307, 1209, 1127, 1055, 1024, 1006, 877, 689 cm^{-1};

HRMS (ESI): calculated for $[C_{14}H_{15}N_3OS+H]^+$: 274.1009, found: 274.1009.

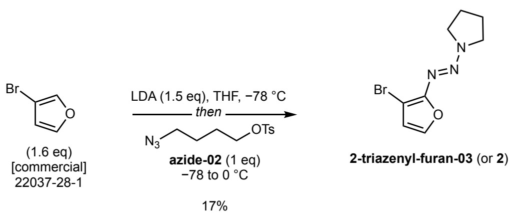

Note: 3-Bromofuran was distilled immediately prior to use using a flame-dried short path distillation apparatus under argon (flask containing 3-bromofuran sitting in a heating mantle set to 120 °C).

To a flame-dried 1-neck 50 mL round bottom flask under argon and equipped with a stir bar was added diisopropylamine (0.960 mL, 6.80 mmol, 1.60 equiv) and anhydrous THF (16.0 mL, 0.400 M). The reaction flask was submerged in a dry ice/acetone bath, and freshly titrated[16] *n*-butyllithium (2.73 mL, 6.38 mmol, 2.34 M in hexane, 1.50 equiv) was added dropwise over approximately 15 minutes. The dry ice/acetone bath was replaced with an ice/water bath, and the reaction was allowed to stir for 5 minutes before being re-submerged in the ice/acetone bath. 3-Bromofuran (1.00 g, 6.80 mmol, 1.60 equiv) was added dropwise over approximately 5 minutes and the reaction was stirred for one hour. A solution of azide (1.15 g, 4.25 mmol, 1.00 equiv) in anhydrous THF (4.25 mL, 1.00 M) was added in one portion and the dry ice/acetone bath was replaced with an ice/water bath. The reaction was stirred for one hour and then quenched with a saturated aqueous solution of NH_4Cl. The reaction was warmed to ambient temperature and extracted with Et_2O (×2). The combined organic layers were washed with water, brine, dried over $MgSO_4$, filtered, and concentrated under reduced pressure. The crude residue was promptly purified (product appears to slowly degrade when held crude neat) by flash column chromatography using phosphate buffered silica (pH = 7, ratio of silica to crude mass = 100:1, eluting with hexane to 5:95 Et_2O:hexane).

Yield: 179 mg, 0.733 mmol, 17%;

Appearance: Yellow to dark orange solid;

R*f*: 0.18 (1:9 EtOAc:hexane);

^{1}H NMR (400 MHz, $CDCl_3$): δ 7.13 (s, 1H), 6.44 (s, 1H), 3.93 (s, 2H), 3.67 (s, 2H), 2.04 (s, 4H) ppm;

^{13}C NMR (100 MHz, $CDCl_3$): δ 154.0, 137.6, 115.4, 88.0, 51.5, 46.8, 24.1, 23.6 ppm;

IR: 3150, 3121, 2975, 2873, 2705, 2363, 2342, 2097, 1598, 1500, 1409, 1352, 1236, 1017 cm^{-1};

HRMS (ESI): calculated for $[C_8H_{10}BrN_3O+Na]^+$: 265.9899, found: 265.9899.

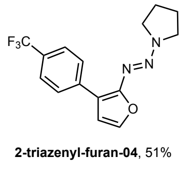

2-triazenyl-furan-04, 51%

Prepared from **triazene-precursor-02** according to the general procedure. Purified by flash column chromatography using silica (ratio of silica to crude mass = 50:1, eluting with hexane to 5:95 Et_2O:hexane).

Yield: 572 mg, 1.85 mmol, 51%;

Appearance: Yellow solid;

R*f*: 0.33 (1:5 EtOAc:hexane);

^{1}H NMR (400 MHz, $CDCl_3$): δ 7.93 (d, *J* = 8.4 Hz, 2H), 7.60 (d, *J* = 8.4 Hz, 2H), 7.21 (d, *J* = 2.2 Hz, 1H), 6.74 (d, *J* = 2.2 Hz, 1H), 4.00 (br s, 2H), 3.72 (br s, 2H), 2.08 (br s, 4H) ppm;

^{13}C NMR (100 MHz, $CDCl_3$): δ 154.0, 138.0, 137.1, 127.6 (q, *J* = 32.5 Hz), 127.8, 125.3 (q, *J* = 4.1 Hz), 125.5 (q, *J* = 271.3 Hz), 111.4, 110.9, 51.5, 47.2, 24.2, 23.6 ppm;

^{19}F NMR (377 MHz, $CDCl_3$): δ −63.0 ppm;

IR: 2974, 2874, 1715, 1502, 1325, 1110, 835 cm^{-1};

HRMS (ESI): calculated for $[C_{15}H_{14}F_3N_3O+H]^+$: 310.1162, found: 310.1160.

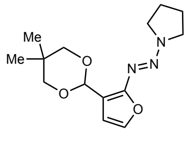

2-triazenyl-furan-05, 34%

Prepared from **triazene-precursor-03** according to the general procedure. Purified by flash column chromatography using phosphate buffered silica (pH = 7, ratio of silica to crude mass = 50:1, eluting with 5:95 EtOAc:hexane to 1:5 EtOAc:hexane).

Yield: 175 mg, 0.627 mmol, 34%;

Appearance: Light orange solid;

R*f*: 0.29 (1:5 EtOAc:hexane);

^{1}H NMR (400 MHz, $CDCl_3$): δ 7.12 (d, *J* = 2.1 Hz, 1H), 6.59 (d, *J* = 2.1 Hz, 1H), 5.67 (s, 1H), 3.99 – 3.67 (m, 6H), 3.61 (d, *J* = 10.4 Hz, 2H), 2.02 (t, *J* = 1.6 Hz, 4H), 1.31 (s, 3H), 0.77 (s, 3H) ppm;

^{13}C NMR (100 MHz, $CDCl_3$): δ 154.8, 137.6, 110.3, 110.3, 96.4, 77.9, 51.3, 46.8, 30.3, 23.9 (2 coincident peaks), 23.1, 22.1, 18.4 ppm;

IR: 2952, 2868, 2089, 1616, 1505, 1468, 1411, 1340, 1276, 1231, 1165, 1102, 1070, 1047, 1013, 957, 924, 883, 737, 695, 651, 533, 511 cm^{-1};

HRMS (ESI): calculated for $[C_{14}H_{21}N_3O_3+H]^+$: 280.1656, found: 280.1652.

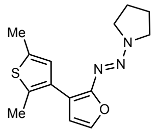

2-triazenyl-furan-06, 52%

Prepared from **triazene-precursor-04** according to the general procedure. Purified by flash column chromatography using phosphate buffered silica (pH = 7, ratio of silica to crude mass = 50:1, eluting with hexane to 5:95 Et_2O:hexane).

Yield: 354 mg, 1.29 mmol, 52%;

Appearance: Yellow solid;

R*f*: 0.44 (1:5 EtOAc:hexane);

^{1}H NMR (400 MHz, $CDCl_3$): δ 7.18 (s, 1H), 6.92 (s, 1H), 6.54 (s, 1H), 3.78 (br s, 4H), 2.44 (s, 3H), 2.42 (s, 3H), 2.02 (br s, 4H) ppm;

^{13}C NMR (100 MHz, $CDCl_3$): δ 152.6, 137.2, 134.7, 132.2, 129.2, 127.7, 113.0, 109.6, 51.1, 46.8, 23.8 (2 coincident peaks), 15.2, 14.9 ppm;

IR: 3853, 3218, 2971, 2869, 2361, 2339, 1685, cm^{-1};

HRMS (ESI): calculated for $[C_{14}H_{17}N_3OS+H]^+$: 276.1165, found: 276.1158.

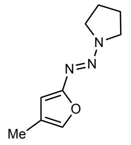

2-triazenyl-furan-07, 50%

Prepared from **triazene-precursor-07** according to the general procedure. Purified by flash column chromatography using phosphate buffered silica (pH = 7, ratio of silica to crude mass = 50:1, eluting with hexane to 5:95 Et_2O:hexane).

Yield: 334 mg, 1.86 mmol, 50%;

Appearance: Light orange solid;

R*f*: 0.33 (1:5 EtOAc:hexane);

^{1}H NMR (400 MHz, $CDCl_3$): δ 6.97 (s, 1H), 5.94 (s, 1H), 3.74 (br s, 4H), 2.01 (br s, 7H). ppm;

^{13}C NMR (100 MHz, $CDCl_3$): δ 159.3, 134.6, 122.2, 98.5, 51.3, 46.8, 23.9 (2 coincident peaks), 10.3 ppm;

IR: 2956, 2926, 2872, 2098, 1763, 1625, 1451, 1409, 1354, 1314, 1277, 1210, 1188, 1175, 1122, 1033, 1009, 933, 816, 683, 569, 556, 421 cm^{-1};

HRMS (ESI): calculated for $[C_9H_{13}N_3O+Na]^+$: 202.0951, found: 202.0951.

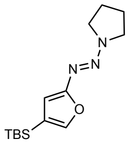

2-triazenyl-furan-08, 46%

Prepared from **triazene-precursor-08** according to the general procedure. Purified by flash column chromatography using phosphate buffered silica (pH = 7, ratio of silica to crude mass = 50:1, eluting with hexane to 5:95 Et_2O:hexane), followed by crystallization of the isolated solid (–20 °C freezer) from hexane.

Yield: 526 mg, 1.88 mmol, 46% yield;

Appearance: Orange solid;

R*f*: 0.46 (1:5 EtOAc:hexane);

^{1}H NMR (400 MHz, $CDCl_3$): δ 7.09 (s, 1H), 6.07 (s, 1H), 3.75 (br s, 4H), 2.01 (t, *J* = 6.4 Hz, 4H), 0.91 (s, 9H), 0.17 (s, 6H) ppm;

^{13}C NMR (150 MHz, $CDCl_3$): δ 160.0, 143.2, 118.7, 100.3, 51.3, 46.4, 26.5, 23.9 (2 coincident peaks), 16.7, −5.6 ppm;

IR: 2924, 2854, 1491, 1407, 1249, 942, 833 cm^{-1};

HRMS (ESI): calculated for $[C_{14}H_{25}N_3OSi+Na]^+$: 302.1659, found: 302.1659.

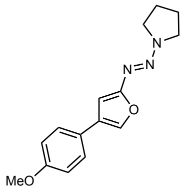

2-triazenyl-furan-09, 49%

Prepared from **triazene-precursor-09** according to the general procedure, except the reaction was extracted with EtOAc. Purified by diluting the crude residue with Et_2O (ca. 4 mL) and filtering, washing the precipitate with a small amount of cold Et_2O.

Yield: 298 mg, 1.10 mmol, 49%;

Appearance: Yellow solid;

R*f*: 0.30 (1:5 EtOAc:hexane);

^{1}H NMR (400 MHz, $CDCl_3$): δ 7.77 (d, *J* = 8.4 Hz, 2H), 7.17 (s, 1H), 6.92 (d, *J* = 8.6 Hz, 2H), 6.67 (s, 1H), 4.01 – 3.65 (m, 7H), 2.06 (br s, 4H) ppm;

^{13}C NMR (100 MHz, $CDCl_3$): δ 158.1, 152.3, 137.6, 128.5, 126.9, 126.0, 114.3, 114.0, 112.7, 111.1, 55.4, 51.3, 46.9, 23.9 (2 coincident peaks) ppm;

IR: 2966, 2877, 2836, 2048, 1585, 1518, 1405, 1352, 1317, 1245, 1184, 1124, 1031, 961, 879, 831, 738, 611, 569 524 cm^{-1};

HRMS (ESI): calculated for $[C_{15}H_{17}N_3O_2+H]^+$: 272.1394, found: 272.1392.

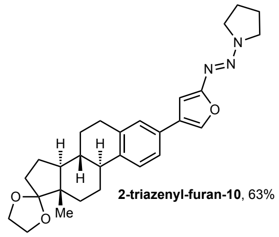

2-triazenyl-furan-10, 63%

Prepared from **triazene-precursor-12** according to the general procedure. Purified by flash column chromatography using phosphate buffered silica (pH = 7, ratio of silica to crude mass = 50:1, eluting with hexane to 1:9 Et_2O:hexane).

Yield: 353 mg, 0.765 mmol, 63%;

Appearance: Yellow solid;

R*f*: 0.26 (1:5 EtOAc:hexane);

^{1}H NMR (400 MHz, $CDCl_3$): δ 7.61 (d, J = 8.3 Hz, 1H), 7.54 (s, 1H), 7.30 (d, J = 8.2 Hz, 1H), 7.18 (s, 1H), 6.69 (s, 1H), 3.99 – 3.88 (m, 6H), 3.72 (br s, 2H), 2.92 – 2.86 (m, 2H), 2.42 – 2.28 (m, 2H), 2.06 – 2.02 (m, 4H), 1.94 – 1.75 (m, 5H), 1.70 – 1.62 (m, 1H), 1.54 – 1.33 (m, 5H), 0.89 (s, 3H) ppm;

^{13}C NMR (100 MHz, $CDCl_3$): δ 152.8, 138.4, 137.6, 136.6, 130.6, 128.0, 125.4, 124.8, 119.6, 113.1, 111.3, 65.4, 64.7, 51.4, 49.6, 46.9, 46.3, 44.2, 39.1, 34.4, 30.9, 29.8, 27.2, 26.1, 24.2, 23.9, 22.5, 14.5 ppm;

IR: 3051, 2939, 2868, 1611, 1586, 1513, 1440, 1323, 1253 cm^{-1};

HRMS (ESI): calculated for $[C_{28}H_{35}N_3O_3+H]^+$: 462.2751, found: 462.2751.

[α]D^{20}: +30.7 (c = 0.52, $CHCl_3$).

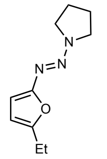

2-triazenyl-furan-11, 58%

Prepared from 2-ethylfuran (cas: 3208-16-0) according to the general procedure. Purified by flash column chromatography using phosphate buffered silica (pH = 7, ratio of silica to crude mass = 50:1, eluting with hexane to 5:95 Et_2O:hexane).

Yield: 988 mg, 5.11 mmol, 58%;

Appearance: Yellow oil;

R*f*: 0.50 (1:5 EtOAc:hexane);

^{1}H NMR (400 MHz, $CDCl_3$): δ 5.96 (s, 2H), 3.74 (br s, 4H), 2.65 (q, J = 7.6 Hz, 2H), 1.99 (s, 4H), 1.22 (t, J = 7.8 Hz, 3H) ppm;

^{13}C NMR (100 MHz, $CDCl_3$): δ 158.1, 153.4, 106.1, 96.1, 49.1 (2 coincident peaks), 23.9 (2 coincident peaks), 21.6, 12.2 ppm;

IR: 2972, 2874, 1415, 1321, 1010, 770;

HRMS (ESI): calculated for $[C_{10}H_{15}N_3O+Na]^+$: 216.1107, found: 216.1107.

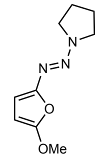

2-triazenyl-furan-12, 45%

Prepared from 2-methoxyfuran (cas: 25414-22-6) according to the general procedure, except the furan lithiation step was run in a dry ice/aqueous $CaCl_2$ bath (–20 °C). Purified by flash column chromatography using phosphate buffered silica (pH = 7, ratio of silica to crude mass = 50:1, eluting with hexane to 1:9 Et_2O:hexane), followed by crystallization of the isolated solid (–20 °C freezer) from a 5:95 Et_2O:hexane mixture (to remove unreacted azide).

Yield: 142 mg, 0.727 mmol, 45%;

Appearance: Yellow solid;

$\mathbf{R}_{\boldsymbol{f}}$: 0.31 (1:5 EtOAc:hexane);

^{1}H NMR (400 MHz, $CDCl_3$): δ 5.94 (d, J = 3.3 Hz, 1H), 5.17 (d, J = 3.3 Hz, 1H), 3.84 (s, 3H), 3.67 (br s, 4H), 1.99 (p, J = 5.7 Hz, 4H) ppm;

^{13}C NMR (100 MHz, $CDCl_3$): δ 158.3, 150.1, 97.8, 82.0, 57.9, 48.8 (2 coincident peaks), 23.9 (2 coincident peaks) ppm;

IR: 3133, 3096, 3055, 2979, 2885, 2359, 2101, 1604, 1564, 1414, 1344, 1268 cm^{-1};

HRMS (ESI): calculated for $[C_9H_{13}N_3O_2+H]^+$: 196.1081, found: 196.1080.

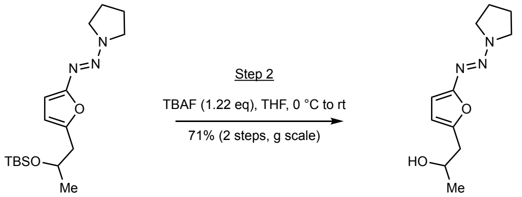

2-triazenyl-furan-13 **2-triazenyl-furan-14**

<u>Step 1</u>

Prepared from **triazene-precursor-14** according to the general procedure. Submitted crude to the next reaction.

<u>Step 2</u>

To a 1-neck 500 mL round-bottom flask open to air, equipped with a stir bar, and containing the crude triazenyl furan (12.8 g, 37.9 mmol, 1.00 equiv) was added THF (75.8 mL, 0.500 M). The reaction flask was submerged in an ice/water bath and TBAF (45.5 mL, 1.00 M in THF, 45.5 mmol, 1.20 equiv) was added dropwise over approximately 3 minutes. The reaction was warmed to ambient temperature and stirred for 16 hours, by which time TLC analysis

indicated complete consumption of **2-triazenyl-furan-13**. The reaction was diluted with H_2O (ca. ×2 reaction volume) and the aqueous layer extracted with EtOAc (×3). The combined organic layers were washed with H_2O, brine, dried over $MgSO_4$, filtered, and concentrated under reduced pressure. The crude residue was purified by flash column chromatography (ratio of silica to crude mass = 50:1, eluting with 2:5 EtOAc:hexane to 1:1 EtOAc:hexane).

Yield: 4.01 g, 18.0 mmol, 71% (2 steps);

Appearance: Yellow oil;

R*f*: 0.14 (2:5 EtOAc:hexane);

^{1}H NMR (400 MHz, $CDCl_3$): δ 6.08 (s, 1H), 5.95 (s, 1H), 4.09 (h, *J* = 6.2 Hz, 1H), 3.72 (br s, 4H), 2.82 – 2.65 (m, 2H), 1.98 (br s, 5H), 1.21 (d, *J* = 6.0 Hz, 3H) ppm;

^{13}C NMR (100 MHz, $CDCl_3$): δ 158.9, 148.1, 109.3, 95.6, 66.7, 50.5, 47.2, 38.3, 23.8 (2 coincident peaks), 22.7 ppm;

IR: 3428, 2971, 2875, 2097, 1597, 1528, 1412, 1332, 1209, 1162, 998 cm^{-1};

HRMS (ESI): calculated for $[C_{11}H_{17}N_3O_2+Na]^+$: 246.1213, found: 246.1212.

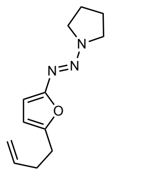

2-triazenyl-furan-15, 51%

Prepared from **triazene-precursor-15** according to the general procedure. Purified by flash column chromatography using phosphate buffered silica (pH = 7, ratio of silica to crude mass = 50:1, eluting with hexane to 5:95 Et_2O:hexane).

Yield: 230 mg, 1.05 mmol, 51%;

Appearance: Orange oil;

R*f*: 0.46 (15:85 EtOAc:hexane);

^{1}H NMR (400 MHz, $CDCl_3$): δ 5.99 (d, *J* = 3.2 Hz, 1H), 5.94 (d, *J* = 3.2 Hz, 1H), 5.89 – 5.78 (m, 1H), 5.05 (dd, *J* = 17.1, 1.7 Hz, 1H), 4.97 (dd, *J* = 10.2, 1.6 Hz, 1H), 3.73 (br s, 4H), 2.71 (t, *J* = 6.7 Hz, 2H), 2.40 (q, *J* = 7.5, 1.6 Hz,, 2H), 1.98 (t, *J* = 7.0 Hz, 4H) ppm;

^{13}C NMR (100 MHz, $CDCl_3$): δ 158.2, 151.0, 137.7, 115.2, 107.2, 95.8, 48.9 (2 coincident peaks), 32.2, 27.8, 23.8 (2 coincident peaks) ppm;

IR: 3077, 2975, 2869, 2107, 1640, 1597, 1525, 1414, 1328, 1228 cm^{-1};

HRMS (ESI): calculated for $[C_{12}H_{17}N_3O+Na]^+$: 242.1264, found: 242.1264.

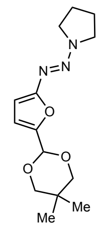

2-triazenyl-furan-16, 42%

Prepared from **triazene-precursor-16** according to the general procedure. Purified by flash column chromatography using phosphate buffered silica (pH = 7, ratio of silica to crude mass = 50:1, eluting with 5:95 EtOAc:hexane to 1:5 EtOAc:hexane).

Yield: 194 mg, 0.694 mmol, 42%;

Appearance: Light orange solid;

$\mathbf{R}_f$: 0.33 (1:2 EtOAc:hexane);

^{1}H NMR (400 MHz, $CDCl_3$): δ 6.46 (d, J = 3.4 Hz, 1H), 6.02 (d, J = 3.4 Hz, 1H), 5.46 (s, 1H), 3.89 (br s, 2H), 3.74 (d, J = 10.9 Hz, 2H), 3.67 – 3.49 (m, 4H), 2.00 (s, 4H), 1.26 (s, 3H), 0.77 (s, 3H) ppm;

^{13}C NMR (100 MHz, $CDCl_3$): δ 159.4, 146.1, 109.4, 96.5, 95.4, 77.6, 51.5, 46.4, 30.5, 23.9 (2 coincident peaks), 23.2, 22.0 ppm;

IR: 2952, 2868, 2429, 2355, 2335, 1527, 1412, 1310, 1214 cm^{-1};

HRMS (ESI): calculated for $[C_{14}H_{21}N_3O_3+H]^+$: 280.1656, found: 280.1655.

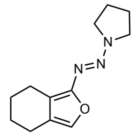

2-triazenyl-furan-17 (or **52**), 42%

Prepared from **triazene-precursor-18** according to the general procedure. Purified by flash column chromatography using phosphate buffered silica (pH = 7, ratio of silica to crude mass = 50:1, eluting with hexane to 3:97 Et_2O:hexane). In some cases, a second column under the same conditions was necessary. This product decomposes if exposed to untreated silica.

Yield: 757 mg, 3.45 mmol, 42%;

Appearance: Yellow to light green solid;

$\mathbf{R}_f$: 0.51 (1:5 EtOAc:hexane);

^{1}H NMR (400 MHz, $CDCl_3$): δ 6.92 (s, 1H), 3.73 (s, 4H), 2.61 (s, 2H), 2.51 (s, 2H), 2.00 (s, 4H), 1.70 (s, 4H) ppm;

^{13}C NMR (100 MHz, $CDCl_3$): δ 152.5, 132.1, 124.0, 109.3, 48.9 (2 coincident peaks), 23.9, 23.4 (3 coincident peaks), 21.2, 20.6 ppm;

IR: 2928, 2857, 1664, 1413, 1282, 860, 661 cm^{-1};

HRMS (ESI): calculated for $[C_{12}H_{17}N_3O+H]^+$: 220.1444, found: 220.14437.

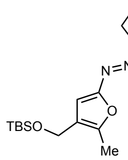

2-triazenyl-furan-18, 25%

Prepared from **triazene-precursor-14** according to the general procedure. Purified by flash column chromatography using phosphate buffered silica (pH = 7, ratio of silica to crude mass = 50:1, eluting with hexane to 5:95 Et_2O:hexane).

Yield: 60.0 mg, 0.186 mmol, 25%;

Appearance: Yellow solid;

R*f*: 0.45 (1:5 EtOAc:hexane);

^{1}H NMR (400 MHz, $CDCl_3$): δ 6.01 (s, 1H), 4.48 (s, 2H), 3.74 (br s, 4H), 2.27 (s, 3H), 1.99 (t, J = 6.5 Hz, 4H), 0.90 (s, 9H), 0.07 (s, 6H) ppm;

^{13}C NMR (100 MHz, $CDCl_3$): δ 157.2, 143.5, 121.4, 97.4, 57.6, 26.1, 23.9 (2 coincident peaks), 18.5, 12.0, −5.03 ppm (two of the pyrrolidinyl peaks are too broad to be visible within this spectra);

IR: 2951, 2926, 2856, 1626, 1536, 1471, 1413, 1317, 1251, 1141, 1063, 943, 835, 814, 775, 667, 572 cm^{-1};

HRMS (ESI): calculated for $[C_{16}H_{29}N_3O_2Si+H]^+$: 324.2102, found: 324.2101.

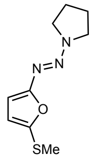

2-triazenyl-furan-19, 60%

Prepared from **triazene-precursor-21** according to the general procedure. Purified by flash column chromatography using phosphate buffered silica (pH = 7, ratio of silica to crude mass = 50:1, eluting with hexane to 1:9 Et_2O:hexane).

Yield: 253 mg, 1.20 mmol, 60%;

Appearance: Yellow solid;

R*f*: 0.38 (1:5 EtOAc:hexane);

^{1}H NMR (400 MHz, $CDCl_3$): δ 6.44 (d, J = 3.3 Hz, 1H), 5.98 (d, J = 3.3 Hz, 1H), 3.87 (br s, 2H), 3.63 (br s, 2H), 2.38 (s, 3H), 2.00 (s, 4H) ppm;

^{13}C NMR (100 MHz, $CDCl_3$): δ 161.5, 142.1, 118.0, 96.4, 51.3, 46.7, 23.8 (2 coincident peaks), 19.8 ppm;

IR: 2975, 2925, 2873, 1572, 1409, 1310, 1226, 1207, 1161, 987 cm^{-1};

HRMS(ESI): calculated for $[C_9H_{13}N_3OS+H]^{+:}$ 212.0852, found: 212.08519.

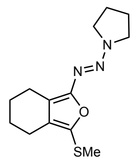

2-triazenyl-furan-20, 49%

Prepared from **triazene-precursor-19** according to the general procedure. Purified by flash column chromatography using phosphate buffered silica (pH = 7, ratio of silica to crude mass = 50:1, eluting with hexane to 5:95 Et_2O:hexane). In some cases, the columned product was contaminated with a non-polar oily impurity which could be removed by dissolving the isolated solid in warm hexane and then allowing to crystallize in the freezer (–20 °C) overnight.

Yield: 215 mg, 1.28 mmol, 49%;

Appearance: Yellow solid;

$\mathbf{R}_{\boldsymbol{f}}$: 0.45 (1:5 EtOAc:hexane);

^{1}H NMR (400 MHz, $CDCl_3$): δ 3.73 (br s, 4H), 2.54 (d, *J* = 19.1 Hz 4H), 2.29 (s, 3H), 1.99 (s, 4H), 1.69 (s, 4H) ppm;

^{13}C NMR (100 MHz, $CDCl_3$): δ 154.4, 135.3, 130.3, 110.0, 48.6 (2 coincident peaks), 23.9 (2 coincident peaks), 23.2, 23.2, 21.6, 21.5, 19.6 ppm;

IR: 2933, 2858, 1766, 1607, 1591, 1524, 1402, 1349, 1235, 1017 cm^{-1};

HRMS (ESI): calculated for $[C_{13}H_{19}N_3O_2S+H]^+$: 266.1322, found: 266.1322.

Derivatization of 2-Triazenyl Furans

summary

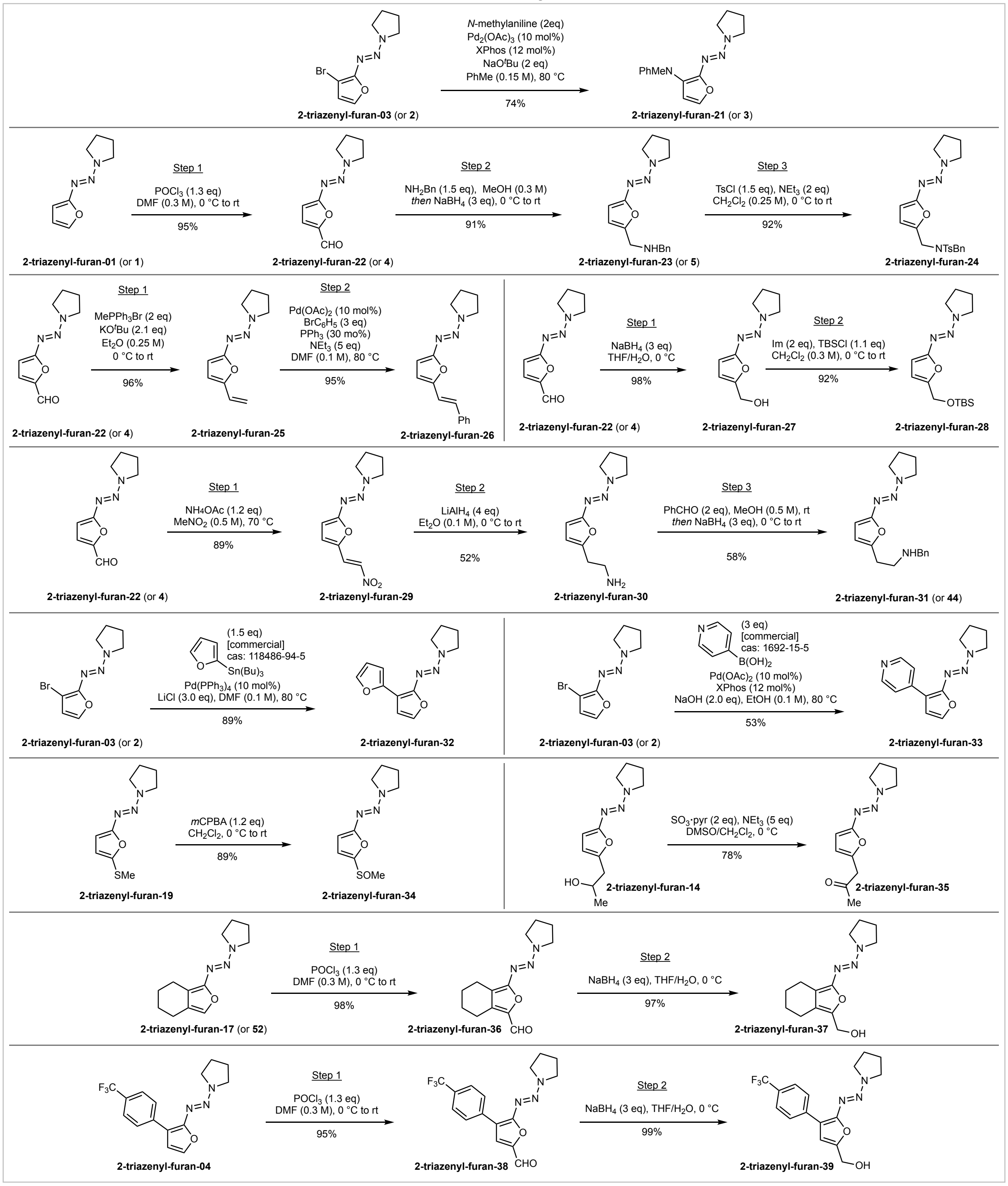

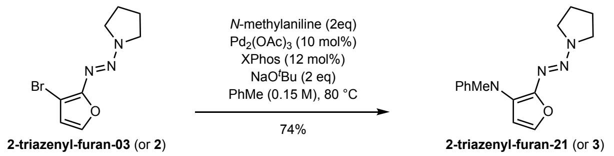

Reaction conducted according to a modification of a related procedure reported by Hartwig.[2b] To a 2-dram vial open to air and equipped with a stir bar was added **2-triazenyl-furan-03** (or **2**) (45.0 mg, 0.184 mmol, 1.00 equiv) and *N*-methyl aniline (39.5 mg, 0.369 mmol, 2.00 equiv). The vial was fitted with a septum, evacuated and backfilled with argon in triplicate, and anhydrous PhMe (1.23 mL, 0.150 M) was added. Argon was bubbled through the solution for 10 minutes, then XPhos (10.5 mg, 0.022 mmol, 0.120 equiv), NaO^{t}Bu (35.4 mg, 0.369 mmol, 2.00 equiv), and $Pd(OAc)_2$ (4.14 mg, 0.018 mmol, 0.10 equiv) were added, causing the reaction to turn dark brown. The vial was sealed with a screw cap, wrapped with electrical tape, and warmed on a heating block at 80 °C for 16 hours. The reaction mixture was concentrated under reduced pressure, and the crude residue was purified by flash column chromatography on phosphate buffered silica (pH = 7, ratio of silica to crude mass = 50:1, eluting with 5:95 EtOAc:hexane to 1:9 EtOAc:hexane).

Yield: 37.0 mg, 0.137 mmol, 74%;

Appearance: Yellow oil;

R*f*: 0.23 (1:5 EtOAc:hexane);

^{1}H NMR (400 MHz, $CDCl_3$): δ 7.20 (t, *J* = 7.9 Hz, 2H), 7.12 (d, *J* = 2.3 Hz, 1H), 6.86 (d, *J* = 8.8 Hz, 2H), 6.75 (t, *J* = 7.8 Hz, 1H), 6.37 (d, *J* = 3.1 Hz, 1H), 3.68 (br s, 4H), 3.30 (s, 3H), 1.97 (p, *J* = 3.5 Hz, 4H) ppm;

^{13}C NMR (100 MHz, $CDCl_3$): δ 149.6, 148.8, 136.6, 128.9, 121.2, 117.8, 114.6, 111.3, 50.0, 46.4, 39.5, 23.9 (2 coincident peaks) ppm;

IR: 2925, 2873, 1595, 1498, 1404, 1326, 751 cm^{-1};

HRMS (ESI): calculated for $[C_{15}H_{18}N_4O+Na]^+$: 293.1373, found: 293.1365.

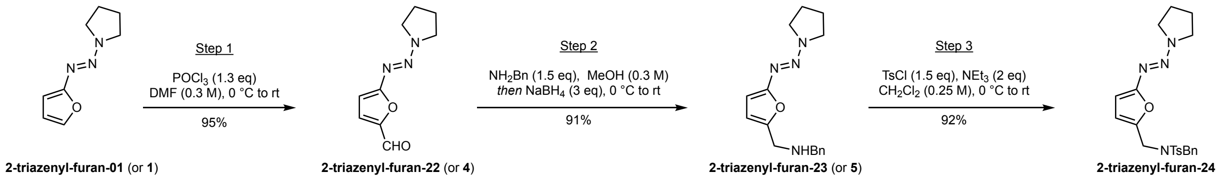

Step 1

Reaction conducted according to a modification of a related procedure reported by Joullie.[40] To a flame-dried 3-neck 250 mL round-bottom flask under argon and equipped with a stir bar was added anhydrous DMF (30.3 mL, 0.300 M). The reaction flask was submerged in an ice/water bath, and $POCl_3$ (1.10 mL, 11.8 mmol, 1.30 equiv) was added dropwise. The reaction was stirred for 10 minutes, then **2-triazenyl-furan-01** (or **1**) (1.50 g, 9.08 mmol, 1.00 equiv) was added in portions, causing the reaction to turn dark yellow and eventually brown. The reaction was stirred for one hour, then warmed to ambient temperature and stirred for 16 hours, by which time TLC analysis indicated the complete consumption of **2-triazenyl-furan-01** (or **1**). A saturated aqueous solution of $NaHCO_3$ was

slowly added (ca. ×3 reaction volume), and the mixture was stirred for three hours, by which time a yellow precipitate had formed. The reaction was extracted with EtOAc (×3), and the combined organic layers were washed with H_2O (×3), brine, dried over $MgSO_4$, filtered, and concentrated under reduced pressure.

Yield: 1.67 g, 8.66 mmol, 95%;

Appearance: Yellow solid;

R*f*: 0.17 (2:5 EtOAc:hexane);

^{1}H NMR (400 MHz, $CDCl_3$): δ 9.49 (s, 1H), 6.22 (s, 1H), 3.98 (t, *J* = 6.1 Hz, 2H), 3.69 (t, *J* = 6.1 Hz, 2H), 2.08 (p, *J* = 6.8 Hz, 5H) ppm;

^{13}C NMR (100 MHz, $CDCl_3$): δ 176.3, 164.7, 147.9, 125.1, 97.3, 52.0, 47.4, 23.9, 23.4 ppm;

IR: 3127, 2969, 2879, 1641, 1558, 1501, 1427, 1371, 1344, 1304, 1249, 1016 cm^{-1};

HRMS (ESI): calculated for $[C_9H_{11}N_3O_2+H]^+$: 194.0924, found: 194.0924.

Step 2

Reaction conducted according to a modification of a related procedure reported by Scheeren.[41] To a flame-dried 1-neck 10 mL round-bottom flask under argon and equipped with a stir bar was added **2-triazenyl-furan-22** (or **4**) (230 mg, 1.19 mmol, 1.00 equiv) and anhydrous MeOH (3.45 mL, 0.300 M). Benzylamine (0.195 mL, 1.79 mmol, 1.50 equiv) was added in one portion and the reaction was stirred for 3 hours, by which time a bright yellow precipitate had formed, and TLC analysis indicated consumption of **2-triazenyl-furan-22** (or **4**). The reaction flask was submerged in an ice/water bath and $NaBH_4$ (135 mg, 3.57 mmol, 3.00 equiv) was added in portions. The reaction was warmed to ambient temperature and stirred for 12 hours, by which time TLC analysis indicated complete consumption of the imine intermediate. The reaction was quenched with H_2O and extracted with EtOAc (×3). The combined organic layers were washed with H_2O, brine, dried over $MgSO_4$, filtered, and concentrated under reduced pressure. Once the crude residue had solidified, warm hexane (ca. 5 mL) was added, and the suspension was placed in the freezer (–20 °C) overnight. The mixture was filtered and the precipitate was washed with hexane (ca. 5 mL).

Yield: 292 mg, 1.08 mmol, 91%;

Appearance: Yellow to tan solid;

R*f*: 0.31 (1:19 MeOH:CH_2Cl_2);

^{1}H NMR (400 MHz, $CDCl_3$): δ 7.27 – 7.21 (m, 4H), 7.20 – 7.14 (m, 1H), 6.13 (d, *J* = 3.3 Hz, 1H), 5.93 (d, *J* = 3.3 Hz, 1H), 3.83 – 3.56 (m, 8H), 1.94 (t, *J* = 2.7 Hz, 4H), 1.72 (s, 1H) ppm;

^{13}C NMR (100 MHz, $CDCl_3$): δ 159.1, 149.1, 140.2, 128.5, 128.4, 127.0, 109.5, 95.5, 52.7, 50.9, 46.6, 45.7, 23.9 (2 coincident peaks) ppm;

IR: 3311, 3026, 2972, 2868, 1407, 1312, 1226, 1207, 1161, 1106, 1009, 963, 906, 773, 737, 698, 560, 470 cm^{-1};

HRMS (ESI): calculated for $[C_{16}H_{20}N_4O+H]^+$: 285.1710, found: 285.1693.

Step 3

To a flame-dried 1-neck 5 mL round-bottom flask under argon and equipped with a stir bar was added **2-triazenyl-furan-23** (or **5**) (15.0 mg, 0.053 mmol, 1.00 equiv), anhydrous CH_2Cl_2 (0.211 mL, 0.250 M), and anhydrous NEt_3 (0.015 mL, 0.106 mmol, 2.00 equiv). The reaction flask was submerged in an ice/water bath and TsCl (15.1 mg, 0.079 mmol, 1.50 equiv) was added in one portion. The reaction was warmed to ambient temperature and stirred for 1.5 hours, by which time TLC analysis indicated complete consumption of **2-triazenyl-furan-23**. The reaction was diluted with Et_2O (ca. ×2 reaction volume) and passed through a phosphate buffered silica plug (pH = 7), eluting with Et_2O. The filtrate was concentrated under reduced pressure, and the crude residue was purified by flash column chromatography on phosphate buffered silica (pH = 7, ratio of silica to crude mass = 30:1, eluting with hexane to 1:3 Et_2O:hexane).

Yield: 21.3 mg, 0.0486 mmol, 92%;

Appearance: Yellow solid;

R_f: 0.19 (1:5 EtOAc:hexane);

^{1}H NMR (400 MHz, $CDCl_3$): δ 7.73 (d, J = 8.3 Hz, 2H), 7.34 – 7.29 (m, 5H), 7.25 – 7.22 (m, 2H), 5.96 (d, J = 3.4 Hz, 1H), 5.84 (d, J = 3.3 Hz, 1H), 4.34 (d, J = 12.8 Hz, 4H), 3.89 (br s, 2H), 3.64 (br s, 2H), 2.39 (s, 3H), 2.04 (br s, 4H) ppm;

^{13}C NMR (100 MHz, $CDCl_3$): δ 159.5, 144.3, 143.0, 137.5, 136.0, 129.6, 128.7, 128.6, 127.8, 127.5, 112.0, 95.4, 51.4, 50.4, 46.5, 43.0, 23.9 (2 coincident peaks), 21.6 ppm;

IR: 3111, 2971, 2868, 1407, 1313, 1207, 772, 698 cm^{-1};

HRMS (ESI): calculated for $[C_{23}H_{26}N_4O_3S+Na]^+$: 461.1618, found: 461.1612.

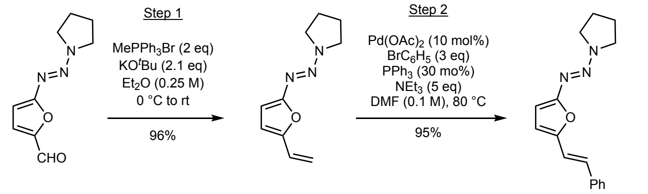

2-triazenyl-furan-22 (or **4**) **2-triazenyl-furan-25** **2-triazenyl-furan-26**

Step 1

Reaction conducted according to a modification of a related procedure reported by Gleason.[42] To a flame-dried 3-neck 25 mL round-bottom flask under argon and equipped with a stir bar was added $MePPh_3Br$ (1.15 g, 3.22 mmol, 2.00 equiv), anhydrous Et_2O (6.44 mL, 0.250 M), and KO^tBu (379 mg, 3.38 mmol, 2.10 equiv), and the reaction was stirred at ambient temperature for two hours. The reaction flask was submerged in an ice/water bath and **2-triazenyl-furan-22** (or **4**) (311 mg, 1.61 mmol, 1.00 equiv) was added in two portions. The reaction warmed to ambient temperature and stirred for 16 hours, by which time TLC analysis indicated the complete consumption of **2-triazenyl-furan-22** (or **4**). The reaction mixture was filtered, concentrated under reduced pressure, and the crude residue was purified by flash column chromatography on phosphate buffered silica (pH = 7, ratio of silica to crude mass = 30:1, eluting with 5:95 Et_2O:hexane).

Yield: 294 mg, 1.54 mmol, 96%

Appearance: Yellow solid;

R*f*: 0.38 (1:5 EtOAc:hexane);

^{1}H NMR (400 MHz, $CDCl_3$): δ 6.43 (dd, *J* = 17.5, 11.3 Hz, 1H), 6.26 (d, *J* = 3.4 Hz, 1H), 6.04 (d, *J* = 3.4 Hz, 1H), 5.65 (d, *J* = 17.5 Hz, 1H), 5.06 (d, *J* = 11.3 Hz, 1H), 3.89 (br s, 2H), 3.66 (br s, 2H), 2.02 (br s, 4H)ppm;

^{13}C NMR (100 MHz, $CDCl_3$): δ 159.4, 148.7, 125.0, 110.8 (2 coincident peaks), 96.4, 51.4, 46.7, 23.9 (2 coincident peaks) ppm;

IR: 2975, 2871, 2429, 2357, 1629, 1367, 1314, 1231 cm^{-1};

HRMS (ESI): calculated for $[C_{10}H_{13}N_3O+H]^+$:192.1131, found: 192.1128.

Step 2

Reaction conducted according to a modification of a related procedure reported by Santi.[43] To a flame-dried 2-dram vial under argon and equipped with a stir bar was added **2-triazenyl-furan-25** (51.2 mg, 0.268 mmol, 1.00 equiv), bromobenzene (0.085 mL, 0.803 mmol, 3.00 equiv), PPh_3 (21.1 mg, 0.080 mmol, 0.30 equiv), NEt_3 (0.187 mL, 1.34 mmol, 5.00 equiv), and anhydrous DMF (2.68 mL, 0.100 M). Argon was bubbled through the solution for approximately 10 minutes and then $Pd(OAc)_2$ (6.01 mg, 0.0268 mmol, 0.10 equiv) was added. The vial was sealed with a screw cap, wrapped with electrical tape, and warmed on a heating block at 80 °C for 36 hours, by which time TLC analysis indicated the consumption of **2-triazenyl-furan-25**. The reaction was cooled to ambient temperature and diluted with H_2O (ca. ×3 reaction volume). The reaction was extracted with EtOAc (×3), and the combined organic layers were washed with H_2O (×3), brine, dried over $MgSO_4$, filtered, and concentrated under reduced pressure. The crude residue was purified by flash column chromatography on phosphate buffered silica (pH = 7, ratio of silica to crude mass = 30:1, eluting with hexane to 5:95 Et_2O:hexane).

Yield: 69.0 mg, 0.258 mmol, 95%

Appearance: Yellow solid;

R*f*: 0.25 (1:5 EtOAc:hexane);

^{1}H NMR (400 MHz, $CDCl_3$): δ 7.44 (d, *J* = 7.9 Hz, 2H), 7.32 (t, *J* = 7.7 Hz, 2H), 7.20 (t, *J* = 7.9 Hz, 1H), 7.05 (d, *J* = 16.1 Hz, 1H), 6.84 (d, *J* = 16.1 Hz, 1H), 6.38 (d, *J* = 3.8 Hz, 1H), 6.12 (d, *J* = 3.7 Hz, 1H), 3.93 (br s, 2H), 3.69 (br s, 2H), 2.04 (br s, 4H) ppm;

^{13}C NMR (100 MHz, $CDCl_3$): δ 159.5, 148.9, 137.6, 128.8, 127.2, 126.2, 125.6, 116.5, 111.8, 97.3, 51.5, 46.9, 23.9 (2 coincident peaks) ppm;

IR: 3034, 2973, 2872, 1630, 1595, 1557, 1446, 1403, 1339, 1310, 1209, 1013, 955, 774, 693 cm^{-1};

HRMS (ESI): calculated for $[C_{16}H_{17}N_3O+H]^+$: 268.1444, found: 268.1445.

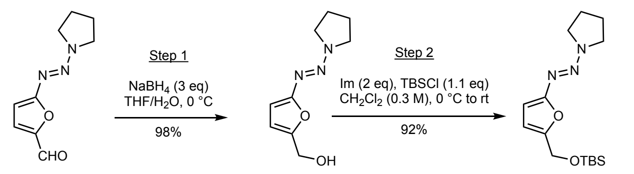

2-triazenyl-furan-22 (or **4**) **2-triazenyl-furan-27** **2-triazenyl-furan-28**

Step 1

Reaction conducted according to a modification of a related procedure reported by Shenvi.[32] To a 1-neck 25 mL round-bottom flask open to air and equipped with a stir bar was added **2-triazenyl-furan-22** (or **4**) (202 mg, 1.05 mmol, 1.00 equiv) THF (5.04 mL, 0.208 M), and H_2O (0.203 mL, 5.00 M). The reaction flask was submerged in an ice/water bath and $NaBH_4$ (119 mg, 3.14 mmol, 3.00 equiv) was added in portions. The reaction was stirred for one hour, by which time TLC analysis indicated the complete consumption of **2-triazenyl-furan-22** (or **4**). The reaction was quenched with H_2O, warmed to ambient temperature, extracted with EtOAc (×3), and the combined organic layers were washed with H_2O, brine, dried over $MgSO_4$, filtered, and concentrated under reduced pressure. Note: **2-triazenyl-furan-27** was found to be slightly light sensitive and should be stored in the dark.

Yield: 200 mg, 1.02 mmol, 98%;

Appearance: Orange solid;

$\mathbf{R}_f$: 0.20 (1:1 EtOAc:hexane);

^{1}H NMR (400 MHz, $CDCl_3$): δ 6.30 (d, J = 3.3 Hz, 1H), 6.00 (d, J = 3.3 Hz, 1H), 4.59 (s, 2H), 3.88 (br s, 2H), 3.66 (br s, 2H), 2.03 (s, 4H), 1.68 (br s, 1H) ppm;

^{13}C NMR (100 MHz, $CDCl_3$): δ 159.4, 149.3, 110.2, 95.8, 57.8, 51.4, 46.6, 23.8 (2 coincident peaks) ppm;

IR: 3350, 2973, 2870, 1408, 1333, 1007, 776 cm^{-1};

HRMS (ESI): calculated for $[C_9H_{13}N_3O_2+Na]^+$: 218.0900, found: 218.0900.

Step 2

To a flame-dried 1-neck 10 mL round-bottom flask under argon and equipped with a stir bar was added **triazenyl-furan-27** (100 mg, 0.512 mmol, 1.00 equiv), anhydrous CH_2Cl_2 (1.71 mL, 0.300 M), and imidazole (69.8 mg, 1.02 mmol, 2.00 equiv). The reaction flask was submerged in an ice/water bath and TBSCl (84.9 mg, 0.564 mmol, 1.10 equiv) was added in one portion. The reaction was warmed to ambient temperature and stirred for 16 hours, by which time TLC analysis indicated the complete consumption of **triazenyl-furan-27**. The reaction was washed with H_2O, brine, dried over $MgSO_4$, filtered, and concentrated under reduced pressure. The crude residue was purified by flash column chromatography on phosphate buffered silica (pH = 7, ratio of silica to crude mass = 30:1, eluting with hexane to 5:95 Et_2O:hexane).

Yield: 145 mg, 0.469 mmol, 92%;

Appearance: Yellow solid;

$\mathbf{R}_f$: 0.50 (1:5 EtOAc:hexane);

^{1}H NMR (400 MHz, $CDCl_3$): δ 6.23 (d, *J* = 3.2 Hz, 1H), 5.99 (d, *J* = 3.1 Hz, 1H), 4.63 (s, 2H), 3.75 (br s, 4H), 2.01 (br s, 4H), 0.90 (s, 9H), 0.07 (s, 6H) ppm;

^{13}C NMR (100 MHz, $CDCl_3$): δ 159.1, 149.7, 109.4, 95.7, 58.6, 51.3, 46.2, 26.1, 23.9 (2 coincident peaks), 18.5, −5.1 ppm;

IR: 3106, 2952, 2926, 2883, 2856, 1732, 1529, 1470, 1415, 1352, 1249, 1109, 1048, 961, 934, 830, 812, 776, 723, 655, 560, 459 cm^{-1};

HRMS (ESI): calculated for $[C_{15}H_{27}N_3O_2Si+H]^+$: 310.1945, found: 310.1944.

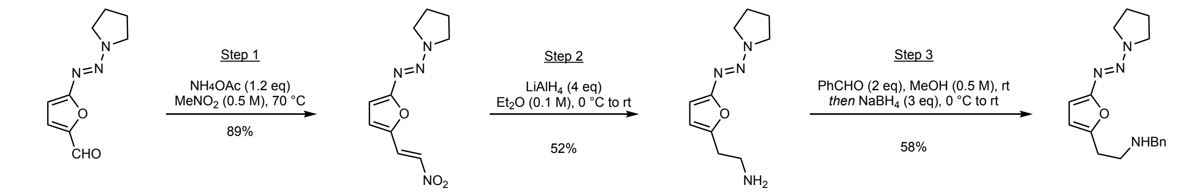

2-triazenyl-furan-22 (or **4**) **2-triazenyl-furan-29** **2-triazenyl-furan-30** **2-triazenyl-furan-31** (or **44**)

Step 1

Reaction conducted according to a modification of a related procedure reported by Suresh.[44] To a 2-dram vial open to air and equipped with a stir bar was added **2-triazenyl-furan-22** (or **4**) (899 mg, 4.65 mmol, 1.00 equiv), $MeNO_2$ (9.31 mL, 0.500 M), and NH_4OAc (430 mg, 5.58 mmol, 1.20 equiv). The vial was sealed with a screw cap, wrapped with electrical tape, and warmed on a heating block at 70 °C for one hour, producing a dark red solution. The reaction was cooled to ambient temperature and passed through a silica plug (ratio of silica to theoretical yield mass = 20:1, eluting with 1:2 EtOAc:hexane), and the filtrate was concentrated under reduced pressure.

Yield: 973 mg, 4.12 mmol, 89%;

Appearance: Violet solid;

R*f*: 0.18 (1:5 EtOAc:hexane);

^{1}H NMR (400 MHz, $CDCl_3$): δ 7.70 (d, *J* = 12.8 Hz, 1H), 7.55 (d, *J* = 13.0 Hz, 1H), 6.92 (d, *J* = 3.7 Hz, 1H), 6.23 (d, *J* = 3.8 Hz, 1H), 3.99 (t, *J* = 6.7 Hz, 2H), 3.70 (t, *J* = 7.0 Hz, 2H), 2.15 – 2.04 (m, 4H) ppm;

^{13}C NMR (100 MHz, $CDCl_3$): δ 164.3, 141.9, 131.9, 125.6, 124.9, 98.6, 52.2, 47.5, 24.0, 23.5 ppm;

IR: 3112, 2977, 2877, 1621, 1493, 1409, 1405, 1381, 1155, 1286, 1254, 1209, 1018, 973, 784, 625, 583, 535 cm^{-1};

HRMS (ESI): calculated for $[C_{10}H_{12}N_4O_3+H]^+$: 237.0982 found: 237.0978.

Step 2

Reaction conducted according to a modification of a related procedure reported by Liu.[45] To a flame-dried 2-neck 50 mL round-bottom flask under argon and equipped with a stir bar and high efficiency air condenser was added **2-triazenyl-furan-29** (600 mg, 2.54 mmol, 1.00 equiv) and anhydrous Et_2O (25.4 mL, 0.100 M). The reaction flask was submerged in an ice/water bath and $LiAlH_4$ (386 mg, 10.2 mmol, 4.00 equiv) was added in portions. The reaction was stirred for one hour, then warmed to ambient temperature and stirred for three hours. The reaction was

worked up employing the "Fieser and Fieser Method": the reaction flask was submerged in an ice/water bath, and H_2O (0.386 mL) was added dropwise, then a 15% aqueous NaOH solution (0.386 mL), and finally H_2O (1.16 mL). The mixture was warmed to ambient temperature, stirred for 15 minutes, then $MgSO_4$ was added and the mixture stirred for 15 minutes. The mixture was filtered and the precipitate washed with Et_2O. The filtrate was concentrated under reduced pressure, and the crude residue was purified by flash column chromatography on phosphate buffered silica (pH = 7, ratio of silica to crude mass = 30:1, eluting with 5:95 MeOH:CH_2Cl_2 to 1:9 MeOH:CH_2Cl_2).

Yield: 273 mg, 1.31 mmol, 52%;

Appearance: Orange to red oil;

R*f*: 0.43 (2:5 MeOH:CH_2Cl_2);

^{1}H NMR (400 MHz, $CDCl_3$): δ 6.06 (d, *J* = 3.2 Hz, 1H), 5.96 (d, *J* = 2.7 Hz, 1H), 3.74 (br s, 4H), 2.97 (t, *J* = 6.4 Hz, 2H), 2.76 (t, *J* = 6.5 Hz, 2H), 2.00 (br s, 4H), 1.59 (br s, 2H) ppm;

^{13}C NMR (100 MHz, $CDCl_3$): δ 158.7, 149.3, 108.5, 95.8, 50.2, 45.5, 40.9, 32.8, 23.9 (2 coincident peaks) ppm;

IR: 3361, 2949, 2870, 1595, 1409, 1329, 1209, 1011, 774 cm^{-1};

HRMS (ESI): calculated for $[C_{10}H_{16}N_4O+H]^+$: 209.1397, found: 209.1393.

<u>Step 3</u>

Conducted according to a modification of a related procedure reported by Marsden and Nelson.[46] To a 1-neck 10 mL round-bottom flask open to air and equipped with a stir bar was added **2-triazenyl-furan-30** (95.0 mg, 0.456 mmol, 1.00 equiv). The flask was evacuated and backfilled with argon in triplicate, then anhydrous MeOH (0.912 mL, 0.500 M) and benzaldehyde (51.8 mg, 1.37 mmol, 2.00 equiv) were added. The reaction was stirred at ambient temperature overnight, by which time TLC analysis indicated complete consumption of **2-triazenyl-furan-30**. The reaction flask was submerged in an ice/water bath and $NaBH_4$ (51.8 mg, 1.37 mmol, 3.00 equiv) was added in portions. The reaction was warmed to ambient temperature and stirred for two hours, by which time TLC analysis indicated complete consumption of the imine intermediate. The reaction was quenched with H_2O and extracted with EtOAc (×3). The combined organic layers were washed with saturated aqueous $NaHCO_3$ solution, brine, dried over $MgSO_4$, filtered, and concentrated under reduced pressure. The crude residue was purified by flash column chromatography on phosphate buffered silica (pH = 7, ratio of silica to crude mass = 30:1, eluting with 1:2 EtOAc:hexane to 2:5 EtOAc:hexane).

Yield: 79.0 mg, 0.265 mmol, 58%;

Appearance: Orange solid;

R*f*: 0.34 (1:5 MeOH:CH_2Cl_2);

^{1}H NMR (400 MHz, $CDCl_3$): δ 7.31 (d, *J* = 4.2 Hz, 4H), 7.26 – 7.21 (m, 1H), 6.05 (d, *J* = 3.1 Hz, 1H), 5.96 (d, *J* = 3.2 Hz, 1H), 3.93 – 3.61 (m, 6H), 2.94 (t, *J* = 11.2 Hz, 2H), 2.86 (t, *J* = 6.6 Hz, 2H), 2.00 (t, *J* = 6.6 Hz, 4H), 1.79 (br s, 1H) ppm;

^{13}C NMR (100 MHz, $CDCl_3$): δ 158.6, 149.4, 140.4, 128.5, 128.2, 127.0, 108.2, 95.8, 53.8, 50.8 (2 coincident peaks), 47.6, 29.0, 23.9 (2 coincident peaks) ppm;

IR: 2948, 2870, 1410, 1325, 1208, 1009, 772 cm^{-1};

HRMS (ESI): calculated for $[C_{17}H_{22}N_4O+H]^+$: 299.1866, found: 299.1862.

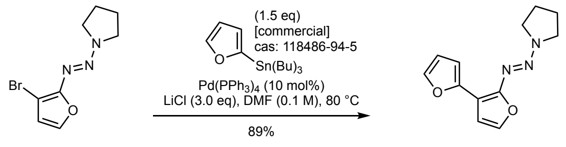

2-triazenyl-furan-03 (or **2**) **2-triazenyl-furan-32**

Conducted according to a modification of a related procedure reported by Chemler.[47] To an oven-dried 2-dram vial under argon and equipped with a stir bar was added **2-triazenyl-furan-03** (or **2**) (30.0 mg, 0.123 mmol, 1.00 equiv), tributyl(furan-2-yl)stannane (65.8 mg, 0.184 mmol, 1.50 equiv), LiCl (15.6 mg, 0.369 mmol, 3.00 equiv), and anhydrous DMF (1.23 mL, 0.100 M). Argon was bubbled through the solution for approximately 10 minutes, then $Pd(PPh_3)_4$ (14.2 mg, 0.0123 mmol, 0.10 equiv) was added. The vial was sealed with a screw cap, wrapped with electrical tape, and warmed on a heating block at 80 °C for four hours. The reaction was cooled to ambient temperature and diluted with H_2O (ca. ×3 reaction volume). The mixture was extracted with EtOAc (×3), and the combined organic layers were washed with H_2O (×3), brine, dried over $MgSO_4$, filtered, and concentrated under reduced pressure. The crude residue was purified by flash column chromatography on phosphate buffered silica (pH = 7, ratio of silica to crude mass = 50:1, eluting with 1:2 CH_2Cl_2/hexane to 2:5 CH_2Cl_2/hexane).

Yield: 25.3 mg, 0.109 mmol, 89%;

Appearance: Yellow solid;

R*f*: 0.33 (1:5 EtOAc:hexane);

^{1}H NMR (400 MHz, $CDCl_3$): δ 7.41 (s, 1H), 7.14 (s, 1H), 6.73 (s, 1H), 6.61 (s, 1H), 6.46 (s, 1H), 3.95 (br s, 2H), 3.72 (br s, 2H), 2.06 (br s, 4H) ppm;

^{13}C NMR (100 MHz, $CDCl_3$): δ 152.0, 148.6, 140.9, 137.8, 111.8, 109.7, 106.7, 105.8, 51.4, 47.0, 24.2, 23.5 ppm;

IR: 3119, 2973, 2872, 1498, 1401, 1334, 1131, 977, 886 cm^{-1};

HRMS (ESI): calculated for $[C_{12}H_{13}N_3O_2+H]^+$: 232.1081, found: 232.1077.

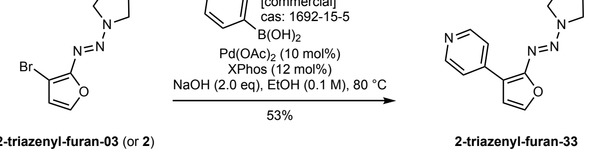

2-triazenyl-furan-03 (or **2**) **2-triazenyl-furan-33**

Conducted according to a modification of a related procedure reported by Zhou.[48] To an oven-dried 2-dram vial under argon and equipped with a stir bar was added **2-triazenyl-furan-03** (or **2**) (70.0 mg, 0.287 mmol, 1.00 equiv), pyridin-4-ylboronic acid (106 mg, 0.860 mmol, 3.00 equiv), XPhos (16.4 mg, 0.0344 mmol, 0.12 equiv), and

anhydrous EtOH (2.87 mL, 0.100 M). Argon was bubbled through the solution for approximately 10 minutes, then $Pd(OAc)_2$ (6.44 mg, 0.0287 mmol, 0.10 equiv) was added and the reaction was stirred at ambient temperature for 5 minutes. NaOH (22.9 mg, 0.574 mmol, 2.00 equiv) was added and the vial was sealed with a screw cap, wrapped with electrical tape, and warmed on a heating block at 80 °C for 16 hours. The reaction was cooled to ambient temperature, passed through a pad of phosphate buffered silica (pH = 7, ratio of silica to crude mass = 10:1, eluting with EtOAc), and the filtrate was concentrated under reduced pressure. The crude residue was purified by flash column chromatography on phosphate buffered silica (pH = 7, ratio of silica to crude mass = 50:1, eluting with 1:1 EtOAc:hexane).

Yield: 37.0 mg, 0.153 mmol, 53%;

Appearance: Yellow solid;

R*f*: 0.15 (1:1 EtOAc:hexane);

^{1}H NMR (400 MHz, $CDCl_3$): δ 8.54 (d, *J* = 6.4 Hz, 2H), 7.72 (d, *J* = 6.5 Hz, 2H), 7.22 (s, 1H), 6.76 (d, *J* = 2.3 Hz, 1H), 4.02 (br s, 2H), 3.76 (br s, 2H), 2.16 – 2.05 (m, 4H) ppm;

^{13}C NMR (100 MHz, $CDCl_3$): δ 155.0, 149.9, 141.0, 138.1, 121.51, 110.4, 109.9, 51.6, 47.4, 24.2, 23.6 ppm;

IR: 3367, 2923, 2853, 2359, 2341, 2048, 1599, 1456, 1406 cm^{-1};

HRMS (ESI): calculated for $[C_{13}H_{14}N_4O+H]^+$: 243.1240, found: 243.1240.

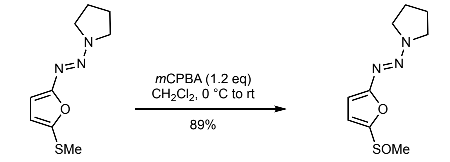

2-triazenyl-furan-19 **2-triazenyl-furan-34**

Conducted according to a modification of a related procedure reported by Procter.[49] To a 3-neck 25 mL round bottom flask open to air and equipped with a stir bar and addition funnel was added **2-triazenyl-furan-19** (150 mg, 0.710 mmol, 1.00 equiv) and CH_2Cl_2 (3.55 mL, 0.200 M). The reaction flask was submerged in an ice/water bath, and a solution of *m*-CPBA (75 wt%, 196 mg, 0.852 mmol, 1.20 equiv) in CH_2Cl_2 (8.52 mL, 0.100 M) was added dropwise via addition funnel over approximately 30 minutes. The reaction warmed to ambient temperature and stirred overnight, by which time TLC analysis indicated the complete consumption of **2-triazenyl-furan-19**. The reaction mixture was washed with saturated aqueous $NaHCO_3$ solution, brine, dried over $MgSO_4$, filtered, and concentrated under reduced pressure.

Yield: 144 mg, 0.634 mmol, 89%;

Appearance: Red solid;

R*f*: 0.45 (1:5 MeOH:CH_2Cl_2);

^{1}H NMR (400 MHz, $CDCl_3$): δ 6.87 (d, *J* = 3.5 Hz, 1H), 6.02 (d, *J* = 3.7 Hz, 1H), 3.88 (t, *J* = 6.7 Hz, 2H), 3.58 (t, *J* = 6.9 Hz, 2H), 2.92 (s, 3H), 2.04 – 1.93 (m, 4H) ppm;

^{13}C NMR (100 MHz, $CDCl_3$): δ 163.4, 146.3, 118.6, 94.7, 51.6, 46.9, 38.4, 23.8, 23.3 ppm;

IR: 3308, 2976 2877, 2359, 2092, 1572, 1555, 1469, 1402, 1306, 1228, 1162 cm^{-1};

HRMS (ESI): calculated for $[C_9H_{13}N_3O_2S+H]^+$: 228.0801, found: 228.0794.

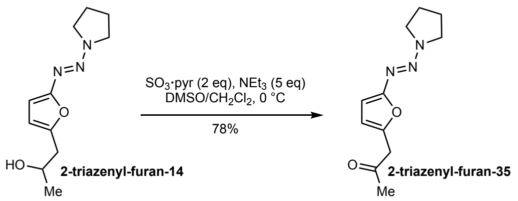

Conducted according to a modification of a related procedure reported by Nicolaou.[50] To a flame-dried 1-neck 10 mL round-bottom flask under argon and equipped with a stir bar was added **2-triazenyl-furan-14** (400 mg, 1.79 mmol, 1.00 equiv), anhydrous DMSO (4.71 mL, 0.400 M), anhydrous CH_2Cl_2 (4.71 mL, 0.400 M), and anhydrous NEt_3 (1.25 mL, 8.96 mmol, 5.00 equiv). The reaction flask was submerged in an ice/water bath, and SO_3•pyridine (570 mg, 3.58 mmol, 2.00 equiv) was added in portions. The reaction was stirred for two hours, by which time TLC analysis indicated the complete consumption of **2-triazenyl-furan-14** (note: longer reaction times were found to be detrimental). The reaction was diluted with water, and the aqueous layer was extracted with CH_2Cl_2 (×2). The combined organic layers were washed with H_2O (×3), brine, dried over $MgSO_4$, filtered, and concentrated under reduced pressure. The crude residue was purified by flash column chromatography on phosphate buffered silica (pH = 7, ratio of silica to crude mass = 50:1, eluting with 15:85 EtOAc/hexane). The product was found to be slightly sensitive when held neat in the freezer (ca. 10% decomposition over the span of several weeks)

Yield: 309 mg, 1.40 mmol, 78%;

Appearance: Yellow to red oil;

R_f: 0.20 (1:2 EtOAc:hexane);

^{1}H NMR (400 MHz, $CDCl_3$): δ 6.18 (d, J = 3.7 Hz, 1H), 5.99 (d, J = 3.7 Hz, 1H), 3.90 – 3.57 (m, 6H), 2.16 (s, 3H), 2.00 (br s, 4H) ppm;

^{13}C NMR (100 MHz, $CDCl_3$): δ 204.6, 159.4, 143.5, 110.6, 95.8, 51.3, 46.5, 43.5, 29.3, 23.8 (2 coincident peaks) ppm;

IR: 2973, 2873, 1713, 1406, 1207, 1023, 774 cm^{-1};

HRMS (ESI): calculated for $[C_{11}H_{15}N_3O_2+H]^+$: 222.1237, found: 222.1230.

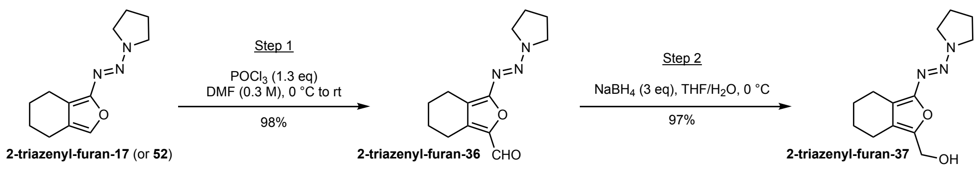

Step 1

Conducted according to a modification of a related procedure reported by Joullie.[40] To a flame-dried 3-neck 25 mL round-bottom flask under argon and equipped with a stir bar was added anhydrous DMF (1.52 mL, 0.300 M). The reaction flask was submerged in an ice/water bath and $POCl_3$ (0.553 mL, 0.593 mmol, 1.30 equiv) was added dropwise. The reaction was stirred for 10 minutes, then **2-triazenyl-furan-17** (or **52**) (100 mg, 0.456 mmol, 1.00 equiv) was added in portions, causing the reaction to turn yellow then eventually dark red. The reaction was stirred for one hour, then warmed to ambient temperature and stirred for 16 hours, by which time TLC analysis indicated the complete consumption of **2-triazenyl-furan-17** (or **52**). A saturated aqueous solution of $NaHCO_3$ was slowly added (ca. ×3 reaction volume), and the mixture was stirred for three hours, by which time a yellow precipitate formed. The reaction was extracted with EtOAc (×3), and the combined organic layers were washed with H_2O (×3), brine, dried over $MgSO_4$, filtered, and concentrated under reduced pressure.

Yield: 111 mg, 0.449 mmol, 98%;

Appearance: Yellow solid;

R_f: 0.21 (2:5 EtOAc:hexane);

^{1}H NMR (400 MHz, $CDCl_3$): δ 9.57 (br s, 1H), 3.94 (t, J = 6.5 Hz, 2H), 3.65 (t, J = 6.8 Hz, 2H), 2.85 (t, J = 6.7 Hz, 2H), 2.58 (t, J = 1.4 Hz, 2H), 2.10 – 1.99 (m, 4H), 1.76 – 1.70 (m, 4H) ppm;

^{13}C NMR (100 MHz, $CDCl_3$): δ 178.2, 157.7, 142.6, 111.8 (2 coincident peaks), 51.8, 47.0, 23.9, 23.4, 22.6, 22.2, 21.2 (2 coincident peaks) ppm;

IR: 2934, 2874, 2348, 1675, 1517, 1388, 1357, 1304, 1210, 1169, 1103, 1042, 970, 614, 557, 457 cm^{-1};

HRMS (ESI): calculated for $[C_{13}H_{17}N_3O_2+H]^+$: 248.1394, found: 248.1390.

Step 2

Conducted according to a modification of the procedure reported by Shenvi.[32] To a 1-neck 5 mL round-bottom flask open to air and equipped with a stir bar was added **2-triazenyl-furan-36** (41.0 mg, 0.166 mmol, 1.00 equiv), THF (0.797 mL, 0.208 M), and H_2O (0.0332 mL, 5.00 M). The reaction flask was submerged in an ice/water bath, and $NaBH_4$ (18.8 mg, 0.497 mmol, 3.00 equiv) was added in one portion. The reaction was stirred for one hour, by which time TLC analysis indicated the complete consumption of **2-triazenyl-furan-36**. The reaction was quenched with H_2O and warmed to ambient temperature. The mixture was extracted with EtOAc (×3), and the combined organic layers were washed with H_2O, brine, dried over $MgSO_4$, filtered, and concentrated under reduced pressure. The product was found to be slightly light sensitive and slowly decomposed over several days when exposed to constant ambient light at room temperature.

Yield: 40.2 mg, 0.161 mmol, 97%;

Appearance: Yellow or brown solid;

R*f*: 0.45 (EtOAc);

^{1}H NMR (400 MHz, $CDCl_3$): δ 4.50 (s, 2H), 3.70 (br s, 4H), 2.56 (s, 2H), 2.49 (s, 2H), 2.25 (br s, 1H), 1.97 (s, 4H), 1.67 (s, 4H) ppm;

^{13}C NMR (100 MHz, $CDCl_3$): δ 152.0, 142.4, 122.0, 109.4, 55.8, 48.9 (2 coincident peaks), 23.9 (2 coincident peaks), 23.2, 23.2, 21.3, 20.4 ppm;

IR: 3354, 2925, 2856, 2244, 1725, 1632, 1568, 1402, 1316, 1210, 1161,1085, 1024, 990, 906, 848, 815, 729, 605, 575, 438 cm^{-1};

HRMS (ESI): calculated for $[C_{13}H_{19}N_3O_2+H]^+$: 250.1550, found: 250.1548.

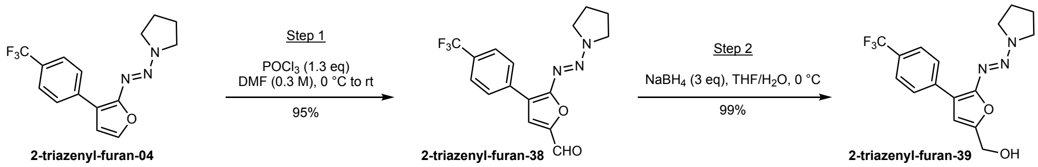

2-triazenyl-furan-04 2-triazenyl-furan-38 2-triazenyl-furan-39

Step 1

Conducted according to a modification of a related procedure reported by Joullie.[40] To a flame-dried 3-neck 25 mL round-bottom flask under argon and equipped with a stir bar was added anhydrous DMF (0.560 mL, 0.300 M). The reaction flask was submerged in an ice/water bath, and $POCl_3$ (0.0204 mL, 0.219 mmol, 1.30 equiv) was added dropwise. The reaction was stirred for 10 minutes, then **2-triazenyl-furan-04** (52.0 mg, 0.168 mmol, 1.00 equiv) was added in portions, causing the reaction to turn orange, and eventually an orange precipitate formed. The reaction was stirred for one hour, then warmed to ambient temperature and stirred for two hours, by which time TLC analysis indicated the complete consumption of **2-triazenyl-furan-04**. A saturated aqueous solution of $NaHCO_3$ solution slowly added (ca. ×3 reaction volume), and the mixture was stirred for three hours, by which time a yellow precipitate formed. The reaction was extracted with EtOAc (×3), and the combined organic layers were washed with H_2O (×3), brine, dried over $MgSO_4$, filtered, and concentrated under reduced pressure. The crude residue was purified by flash column chromatography on silica (ratio of silica to crude mass = 50:1, eluting with 1:3 EtOAc:hexane).

Yield: 53.7 mg, 0.159 mmol, 95%;

Appearance: Yellow solid;

R*f*: 0.20 (2:5 EtOAc:hexane);

^{1}H NMR (400 MHz, $CDCl_3$): δ 9.57 (s, 1H), 7.93 (d, J = 8.1 Hz, 2H), 7.64 (d, J = 8.1 Hz, 2H), 7.56 (s, 1H), 4.08 (t, J = 6.7 Hz, 2H), 3.78 (t, J = 6.9 Hz, 2H), 2.17 – 2.09 (m, 4H) ppm;

^{13}C NMR (100 MHz, $CDCl_3$): δ 176.9, 158.0, 147.1, 135.6, 135.6, 128.6 (q, J = 32.6 Hz), 127.4, 125.5 (q, J = 3.7 Hz), 124.4 (q, J = 274.2 Hz), 113.7, 52.3, 48.1, 24.1, 23.4 ppm;

^{19}**F NMR** (377 MHz, $CDCl_3$): δ −63.00 ppm;

IR: 2979, 2879, 1663, 1386, 1324, 1117, 1068, 840 cm^{-1}

HRMS (ESI): calculated for $[C_{16}H_{14}N_3O_2F_3+H]^+$: 338.1111, found: 338.1106.

Step 2

Conducted according to a modification of the procedure reported by Shenvi.[32] To a 2-dram vial open to air and equipped with a stir bar was added the **2-triazenyl-furan-38** (32.0 mg, 0.0949 mmol, 1.00 equiv), THF (0.456 mL, 0.208 M), and H_2O (0.019 mL, 5.00 M). The reaction flask was submerged in an ice/water bath, and $NaBH_4$ (10.8 mg, 0.285 mmol, 3.00 equiv) was added in one portion. The reaction was stirred for one hour, by which time TLC analysis indicated the complete consumption of **2-triazenyl-furan-38**. The reaction was quenched with H_2O and warmed to ambient temperature. The mixture was extracted with EtOAc (×3), and the combined organic layers were washed with H_2O, brine, dried over $MgSO_4$, filtered, and concentrated under reduced pressure.

Yield: 31.8 mg, 0.0937 mmol, 99%

Appearance: Yellow solid;

R*f*: 0.24 (1:1 EtOAc:hexane);

^{1}**H NMR** (400 MHz, $CDCl_3$): δ 7.88 (d, *J* = 8.0 Hz, 2H), 7.58 (d, *J* = 8.1 Hz, 2H), 6.64 (s, 1H), 4.63 (s, 2H), 3.97 (br s, 2H), 3.69 (br s, 2H), 2.39 – 1.99 (m, 5H) ppm;

^{13}**C NMR** (100 MHz, $CDCl_3$): δ 153.6, 149.3, 136.9, 127.6 (q, *J* = 31.7 Hz), 127.2, 125.3 (q, *J* = 3.8 Hz), 124.6 (q, *J* = 271.7 Hz), 112.0, 108.9, 57.7, 51.5, 47.3, 24.2, 23.5 ppm;

^{19}**F NMR** (377 MHz, $CDCl_3$): δ –63.07 ppm;

IR: 3355, 2971, 2876, 1615, 1554, 1316, 1160, 1105, 999, 838 cm^{-1};

HRMS (ESI): calculated for $[C_{16}H_{16}N_3O_2F_3+Na]^+$: 362.1081, found: 362.1077.

Synthesis of a 3-Triazenyl Furan

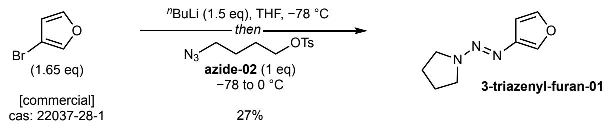

Note: 3-Bromofuran was distilled immediately prior to use using a flame-dried short path distillation apparatus under argon (flask containing 3-bromofuran sitting in a heating mantle set to 120 °C).

Conducted according to a modification of the procedure reported by Severin.[9] To a flame-dried 50 mL 1-neck round-bottom flask under argon and equipped with a stir bar was added 3-bromofuran (6.13 mmol, 0.551 mL, 1.65 equiv) and anhydrous THF (13.9 mL, 0.27 M). The flask was submerged in a dry ice/acetone bath and freshly titrated[16] *n*-butyllithium (2.53 mL, 5.57 mmol, 2.20 M in hexane, 1.50 equiv) was added dropwise. The reaction was stirred for one hour and a solution of **azide-02** (3.71 mmol, 1.00 g, 1.00 equiv) in anhydrous THF (3.71 mL, 1.00 M) was added dropwise. The reaction was stirred for one hour and then transferred to an ice/water bath and stirred for an additional 30 minutes. The reaction was quenched with a saturated aqueous solution of NH_4Cl, warmed to ambient temperature, and extracted with EtOAc (×3). The combined organic layers were washed with H_2O, brine, dried over $MgSO_4$, filtered, and concentrated under reduced pressure. The crude residue was purified by flash column chromatography using phosphate buffered silica (pH = 7, ratio of buffered silica to crude mass = 50:1, eluting with 1:9 Et_2O:hexane).

Yield: 157 mg, 0.95 mmol, 27%;

Appearance: Light orange solid;

R*f*: 0.22 (1:9 EtOAc:hexane);

^{1}H NMR (400 MHz, $CDCl_3$): δ 7.62 (s, 1H), 7.29 (s, 1H), 6.63 (s, 1H), 3.70 (br s, 4H), 1.98 (br s, 4H) ppm;

^{13}C NMR (100 MHz, $CDCl_3$): δ 143.0, 141.4, 135.3, 103.3, 48.6 (2 coincident peaks), 23.9 (2 coincident peaks) ppm;

IR: 2972, 2930, 2871, 1425, 1246, 1126, 974, 864 cm^{-1};

HRMS (ESI): calculated for $[C_8H_{11}N_3O+H]^+$: 166.0975, found: 166.0970.

Triazenyl Furan Stability and Hazard Assessment

Stability Studies

Stability studies adapted from those originally disclosed by Sherburn[51] and Newton.[6, 52]

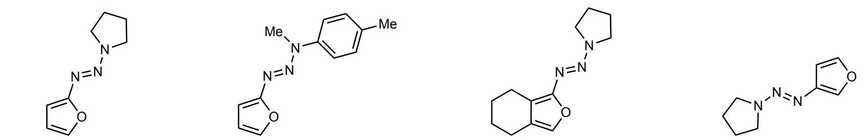

	**2-triazenyl-furan-01** (or **1**)	**scouting-02**	**2-triazenyl-furan-17** (or **52**)	**3-triazenyl-furan-01**
**oxygen**[a]	≥95%	≥95%	≥95%	≥95%
**heat** (120 °C)[b]	≥95%	≥95%	0% (100 °C), ≥95% (80 °C)	≥95%
**AcOH**[c]	≥95%	≥95%	≥95%	≥95%
**TFA**[d]	≥0%	≥0%	≥0%	≥0%
**silica** (column)[e]	≥95%	≥95%	79%	≥95%
**water**[f]	91%	90%	≥95%	≥95%

[a] O_2 bubbled through a $CDCl_3$ solution (0.025 M) for 5 min, vial sealed, stirred for 30 min

[b] C_6D_6 solution (0.025 M) heated at 120 °C in a microwave reactor for 2 h

[c] Aqueous AcOH (0.1 M in $CDCl_3$) stirred for 1 h

[d] TFA (0.1 M in $CDCl_3$) stirred for 1 h

[e] 50 mg of furan subjected to flash chromatography with 5 g silica as stationary phase

[f] $(CD)_3CO/D_2O$ (9:1, 0.025 M) solution stirred for 1 h

Oxygen

To a 2-dram vial equipped with a stir bar was added a 0.025 M $CDCl_3$ solution of the appropriate furan ($CDCl_3$ stored over K_2CO_3 to neutralize any trace acid present) followed by an internal standard (durene). An initial 1H NMR spectrum was obtained. The vial was fitted with a rubber septum, and anhydrous O_2 gas (balloon) was bubbled through the solution for five minutes. The vial was sealed with a screw cap, wrapped with electrical tape, and stirred under an O_2 atmosphere for 30 minutes. An ^{1}H NMR spectrum was obtained to determine whether any decomposition had occurred.

Heat

To a flame-dried 10 mL microwave vessel equipped with a stir bar under argon was the appropriate furan, an internal standard (durene), C_6D_6 (0.025 M), and BHT (ca. 2 mg/mL) to inhibit the autoxidative effects of any oxygen present in solution. An initial ^{1}H NMR spectrum was obtained. The vial was sealed, and the sample was heated to 120 °C for 2 hours in a microwave reactor. An ^{1}H NMR spectrum was obtained to determine whether any decomposition had occurred.

AcOH

To a 2-dram vial equipped with a stir bar was added a 0.025 M $CDCl_3$ solution of the appropriate furan, an internal standard (durene), and BHT (ca. 2 mg/mL) to inhibit the autoxidative effects of any oxygen present in solution. An initial ^{1}H NMR spectrum was obtained. An equivalent volume of 0.20 M AcOH in $CDCl_3$ was added, the vial was

sealed with a screw cap, wrapped with electrical tape, and the sample was stirred for 1 hour. An ^{1}H NMR spectrum was obtained to determine whether any decomposition had occurred.

TFA

To a 2-dram vial equipped with a stir bar was added a 0.025 M $CDCl_3$ solution of the appropriate furan, an internal standard (durene), and BHT (ca. 2 mg/mL) to inhibit the autoxidative effects of any oxygen present in solution. An initial ^{1}H NMR spectrum was obtained. An equivalent volume of 0.20 M TFA in $CDCl_3$ was added, the vial was sealed with a screw cap, wrapped with electrical tape, and the sample was stirred for 1 hour. An ^{1}H NMR spectrum was obtained to determine whether any decomposition had occurred.

Silica Column

A flash chromatography column was charged with 5.00 g of silica. The silica was pre-wet with hexane and 50.0 mg of the appropriate furan was loaded onto the silica using a minimum volume of CH_2Cl_2 (ca. 1 mL). The column was eluted with 50 mL of hexane, then 50 mL of Et_2O, then 50 mL of EtOAc, directly into a round bottom flask, and the solvent was removed under reduced pressure. An internal standard (durene) was added to each sample and an ^{1}H NMR spectrum was obtained to determine the percent recovery of substrate.

Water

To a 2-dram vial equipped with a stir bar was added 0.025 M $(CD_3)_2CO/D_2O$ (9:1) solutions of the appropriate furan, an internal standard (durene), and BHT (ca. 2 mg/mL) to inhibit the autoxidative effects of any oxygen present in solution. An initial ^{1}H NMR spectrum was obtained. The vial was sealed with a screw cap, wrapped with electrical tape, and the sample was stirred for 1 hour. An ^{1}H NMR spectrum was obtained to determine whether any decomposition had occurred.

Hazard Assessment

Hazard assessment adapted from those originally disclosed by Boyn and Roberts.[53] **2-Triazenyl-furan-01** (or **1**) and **3-triazenyl-furan-01** were submitted for thermogravimetric analysis (TGA) and differential scanning calorimetry (DSC). With respect to the former, decomposition appears to occur above their boiling point, as such, we have elected to omit TGA data. The DSC data were then input into the following hazard assessment calculators:

- Vertex Pharmaceutical's OREOS+ Scale.[54]
- AstraZeneca and Merck's shock sensitivity predictive model.[55]

Differential Scanning Calorimetry

2-Triazenyl-furan-01, ramp rate at 5.0 °C/min. A decomposition event is observed to reach maximum heat flow at 267 °C, with a calculated enthalpy of 1474.8 J/g.

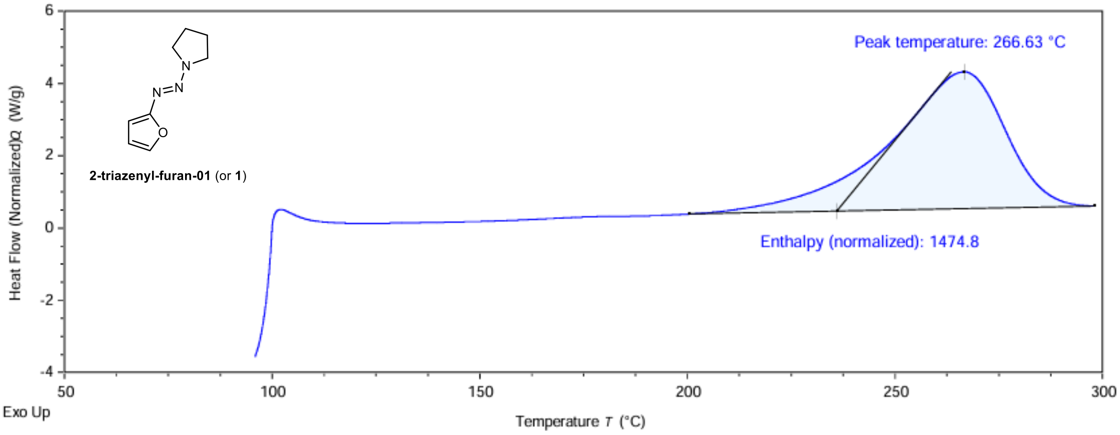

3-Triazenyl-furan-01, ramp rate at 5.0 °C/min. A decomposition event is observed to reach maximum heat flow at 227 °C, with a calculated enthalpy of 1770.8 J/g.

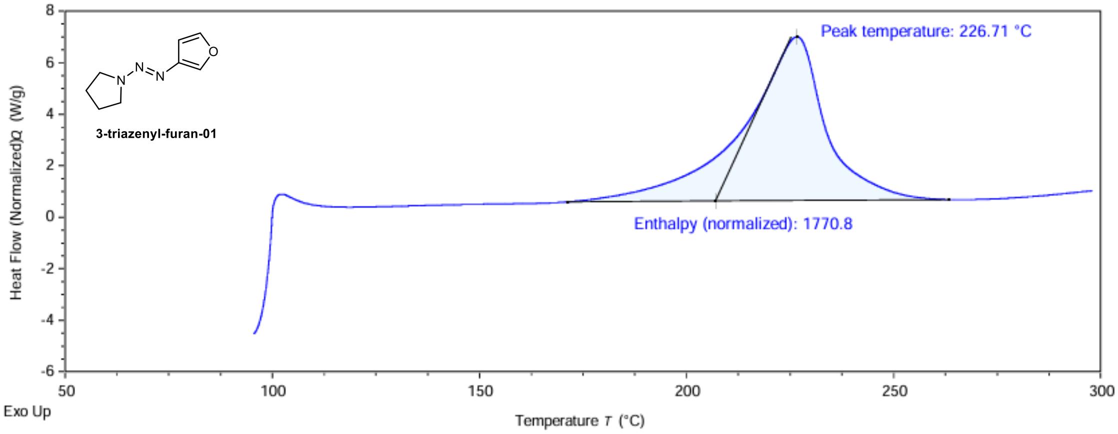

Vertex Pharmaceutical's OREOS+ Scale

The OREOS+ calculator evaluates several parameters to estimate possible explosion risk in handling a compound of interest.[54]

The parameters are as listed:

- Explosive Functional Group (ExFG)
- Oxygen Balance Equation
- Enthalpy of Decomposition
- Onset Temperature of Decomposition
- Rule of 6
- Scale

Criterion	**Score Points**					
	**-40**	**0**	**1**	**2**	**4**	**8**
ExFG			No			Yes
OB hazard				LOW	MEDIUM	HIGH
total[a] (exo) ΔH_D	< 500 J/g	≥ 500 J/g				
$T_{LL\text{-}onset}$			> 300°C	200-300°C	125-200°C	< 125°C
Rule of 6				Pass		Fail
Scale			< 5 g	5-100 g	100-500 g	> 500g
**OREOS+ final explosion hazard ranking**						
HIGH explosion hazard	Score: 28 to 40			LOW explosion hazard	Score: 7 to 17	
MEDIUM explosion hazard	Score: 18 to 27			NO explosion hazard	Score ≤0	

3-triazenyl-furan-01 (or **1**)

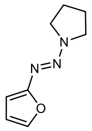

2-triazenyl-furan-01 (or **1**)

Chemical Formula: $C_8H_{11}N_3O$
Explosive FG: Yes
Molecular Weight: 165.20
OB hazard: medium
ΔH_D = 1474.8 J/g
Onset Temperature: ~210 °C
Rule of 6: Pass

Scale:	OREOS+ (Score)
<5g	Low Risk (17)
5-100g	Medium Risk (18)
100-500g	Medium Risk (20)
>500g	Medium Risk (24)

Criterion	Score Points					
	-40	0	1	2	4	8
ExFG			No			Yes
OB hazard				LOW	MEDIUM	HIGH
total[a] (exo) ΔH_D	< 500 J/g	≥ 500 J/g				
$T_{LL\text{-}onset}$			> 300°C	200-300°C	125-200°C	< 125°C
Rule of 6				Pass		Fail
Scale			< 5 g	5-100 g	100-500 g	> 500g
**OREOS+ final explosion hazard ranking**						
HIGH explosion hazard	Score: 28 to 40			LOW explosion hazard	Score: 7 to 17	
MEDIUM explosion hazard	Score: 18 to 27			NO explosion hazard	Score ≤0	

3-triazenyl-furan-01

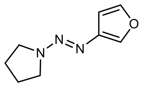

3-triazenyl-furan-01

Chemical Formula: $C_8H_{11}N_3O$
Explosive FG: Yes
Molecular Weight: 165.20
OB hazard: medium
ΔH_D = 1770.8 J/g
Onset Temperature: ~175 °C
Rule of 6: Pass

Scale:	OREOS+ (Score)
<5g	Medium Risk (19)
5-100g	Medium Risk (20)
100-500g	Medium Risk (22)
>500g	Medium Risk (26)

Criterion	Score Points					
	-40	0	1	2	4	8
ExFG			No			Yes
OB hazard				LOW	MEDIUM	HIGH
total[a] (exo) ΔH_D	< 500 J/g	≥ 500 J/g				
$T_{LL\text{-}onset}$			> 300°C	200-300°C	125-200°C	< 125°C
Rule of 6				Pass		Fail
Scale			< 5 g	5-100 g	100-500 g	> 500g
**OREOS+ final explosion hazard ranking**						
HIGH explosion hazard	Score: 28 to 40			LOW explosion hazard	Score: 7 to 17	
MEDIUM explosion hazard	Score: 18 to 27			NO explosion hazard	Score ≤0	

Overall, **2-triazenyl-furan-01** (or **1**) and **3-triazenyl-furan-01** each display a low to medium explosion hazard risk according to thermal decomposition pathways.

AstraZeneca and Merck's Shock Sensitivity Predictive Model.[55]

This predictive model builds off the Yoshida correlation (e.g., DSC initiation temperature and enthalpy), while also accounting for the oxygen balance and the number of energetic nitrogen groups in a molecule.

Material-specific variables	Value	Range within Dataset			Description
		Low	High	Out of Range?	
MW (g/mol)	0				Molecular Weight (used in OB_{100} calc.)
n_O	0				Number of Oxygen atoms (used in OB_{100} calc.)
n_H	0				Number of Hydrogen atoms (used in OB_{100} calc.)
n_C	0				Number of Carbon atoms (used in OB_{100} calc.)
n_{COO}	0				Number of Carboxyl groups (used in OB_{100} calc.)
ENG	0	0	2	NO	Number of "Energetic Nitrogen Groups" in the molecule, specifically diazo, diazonium, azide, triazole (with 3 adjacent N atoms), and tetrazole. Note: Do not include nitro and nitrate groups
Calculated OB_{100}	#DIV/0!	-22.6	5.1	#DIV/0!	Oxygen Balance (adjusted for carboxyls)
DSC exotherm magnitude (ΔH), Units J/g	0				From DSC integration. Select appropriate units, a positive number corresponds to an exothermic event
Calculated Log(ΔH), using cal/g	#NUM!	1.5	3.1	#NUM!	
Tinit (°C)	0	40	364	YES	DSC exotherm initiation temp (left-limit of integration, where peak just begins to deviate from baseline)
Calculated Logit					
Calculated Probability					
Theshold Value	0.1				Probability threshold above which the material is predicted to be shock-sensitive; default is 0.1
Predicted to be Shock Sensitive?	N/A			#DIV/0!	

2-triazenyl-furan-01 (or **1**)

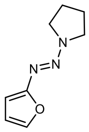

2-triazenyl-furan-01 (or 1)

Material-specific variables		Value	Range within Dataset		
			Low	High	Out of Range?
MW (g/mol)		165.2			
n_O		1			
n_H		11			
n_C		8			
n_{COO}		0			
ENG		1	0	2	NO
Calculated OB_{100}		-15.1	-22.6	5.1	NO
DSC exotherm magnitude (ΔH), Units	J/g	1474.8			
Calculated Log(ΔH), using cal/g		2.55	1.5	3.1	NO
Tinit (°C)		210	40	364	NO
Calculated Logit		-4.579			
Calculated Probability		**0.01**			
Theshold Value		0.1			
Predicted to be Shock Sensitive?		**NO**			

3-triazenyl-furan-01

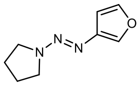

3-triazenyl-furan-01

Material-specific variables		Value	Range within Dataset		
			Low	High	Out of Range?
MW (g/mol)		165.2			
n_O		1			
n_H		11			
n_C		8			
n_{COO}		0			
ENG		1	0	2	NO
Calculated OB_{100}		-15.1	-22.6	5.1	NO
DSC exotherm magnitude (ΔH), Units	J/g	1770.8			
Calculated Log(ΔH), using cal/g		2.63	1.5	3.1	NO
Tinit (°C)		175	40	364	NO
Calculated Logit		-2.670			
Calculated Probability		**0.06**			
Theshold Value		0.1			
Predicted to be Shock Sensitive?		**NO**			

Finally, a rudimentary empirical measurement was carried out by striking 3–5 mgs of **2-triazenyl-furan-01** (or **1**) and **3-triazenyl-furan-01** with a hammer and either a "go" or "no-go" event was recorded (a description of these events has been provided by Williams.[56] **2-Triazenyl-furan-01** (or **1**) and **3-triazenyl-furan-01** each exhibited "no-go" events and thus appear insensitive to impact decomposition.

Diels–Alder Reactions

Mechanism

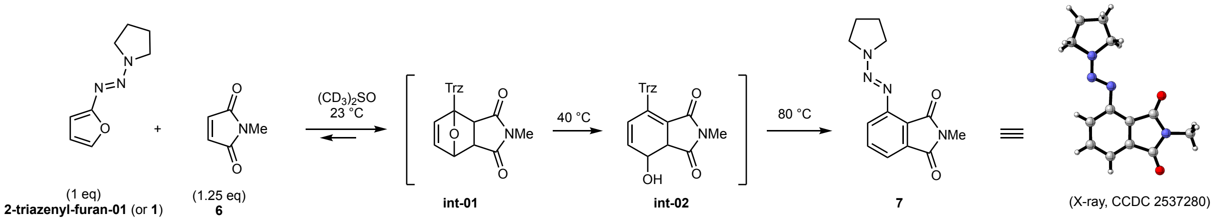

To a 2-dram vial open to air and equipped with a stir bar was added **2-triazenyl-furan-01** (or **1**) (100.0 mg, 0.605 mmol, 1.00 equiv), $(CD_3)_2SO$ (1.21 mL, 0.500 M), and *N*-methylmaleimide (**6**) (84.1 mg, 0.757 mmol, 1.25 equiv). ^{1}H NMR aliquots were collected every 30 minutes. Within 5 hours, the reaction had reached equilibrium (ca. 3:7 **2-triazenyl-furan-01**: a diastereomeric mixture of Diels–Alder adducts). The diastereomeric ratio was observed to change over the course of this time period, indicating a reversible process. During this period, one diastereoisomer, ***exo*-int-01**, partially crashed out of solution as a white solid. This appears to be the thermodynamic product, and NOE experiments indicate it is the *exo*-diastereoisomer. Upon warming the reaction mixture to 40 °C, **int-01** irreversibly converted into **int-02**. Upon warming the reaction mixture to 80 °C, aromatization to **7** occurred.

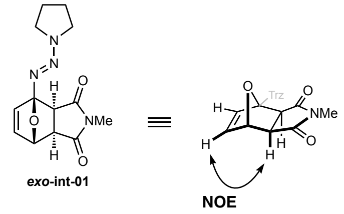

An authentic sample was isolated via the following procedure:

To a 2-dram vial open to air and equipped with a stir bar was added **2-triazenyl-furan-01** (or **1**) (100 mg, 0.605 mmol, 1.00 equiv), *N*-methylmaleimide (**6**) (135 mg, 1.21 mmol, 2.00 equiv), and PhMe (0.605 mL, 1.00 M). The reaction was stirred at ambient temperature for 16 hours, during which time a white precipitate formed. The mixture was filtered over a cotton plug, and the solid was washed with cold Et_2O (ca. 3 mL) and dried under vacuum to give a pure sample of the major diastereomer of ***exo*-int-01**.

Appearance: Powdery white solid;

^{1}H NMR (400 MHz, CD_2Cl_2): δ 6.65 (d, *J* = 5.7 Hz, 1H), 6.57 (dd, *J* = 5.7, 1.9 Hz, 1H), 5.17 (d, *J* = 1.9 Hz, 1H), 3.84 (br s, 2H), 3.49 (br s, 2H), 3.04 (d, *J* = 6.4 Hz, 1H), 2.93 (d, *J* = 6.4 Hz, 1H), 2.91 (s, 3H), 1.99 (br s, 4H) ppm;

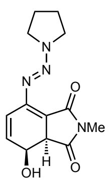

***exo*-int-02**

An authentic sample was isolated via the following procedure:

Approximately 10 mg of ***exo*-int-01** was dissolved in 0.500 mL of (non-basified) $CDCl_3$ in an NMR tube. After 5 minutes, the sample went from colorless to bright yellow and ^{1}H NMR analysis showed clean conversion to ***exo*-int-02**.

Appearance: Yellow solid;

^{1}H NMR (400 MHz, $CDCl_3$): δ 6.99 (d, *J* = 9.9 Hz, 1H), 6.49 (dd, *J* = 9.8, 5.7 Hz, 1H), 4.62 (br s, 1H), 3.93 (t, *J* = 6.5 Hz, 2H), 3.90 – 3.80 (m, 1H), 3.79 – 3.69 (m, 1H), 3.61 (d, *J* = 5.5 Hz, 1H), 3.03 (s, 3H), 2.16 (s, 1H), 2.06 (dq, *J* = 12.8, 6.9 Hz, 4H) ppm.

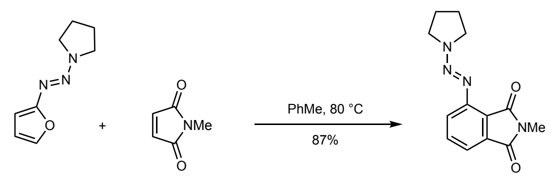

To a 2-dram vial open to air and equipped with a stir bar was added **2-triazenyl-furan-01** (or **1**) (50.0 mg, 0.303 mmol, 1.00 equiv), PhMe (0.605 mL, 0.500 M), and *N*-methylmaleimide (**6**) (42.0 mg, 0.378 mmol, 1.25 equiv). The vial was sealed with a screw cap, wrapped with electrical tape, and warmed on a heating block at 80 °C for 16 hours, by which time ^{1}H NMR analysis indicated the complete consumption of the triazenyl furan and any non-aromatized cycloadducts. *N*-methylmaleimide proved to be challenging to remove from **7**, therefore we elected to converted excess dienophile to a more easily separable species: Furan (0.440 mL, 6.05 mmol, 20.0 equiv) was added, the vial was sealed with a screw cap, wrapped with electrical tape, and warmed on a heating block at 60 °C for 5 hours, then cooled to ambient temperature and concentrated under reduced pressure. The crude residue was purified by flash column chromatography (ratio of silica to crude mass= 100:1, eluting with 5:95 EtOAc:hexane to 2:5 EtOAc:hexane).

Yield: 68.0 mg, 0.263 mmol, 87%;

Appearance: Yellow solid;

R*f*: 0.16 (1:2 EtOAc:hexane);

M.p.: 177.0 – 181.4 °C;

^{1}H NMR (400 MHz, $CDCl_3$): δ 7.69 (t, *J* = 3.3 Hz, 1H), 7.56 (d, *J* = 4.6 Hz, 2H), 3.99 (t, *J* = 6.8 Hz, 2H), 3.84 (t, *J* = 6.9 Hz, 2H), 3.15 (s, 3H), 2.08 (h, *J* = 6.8 Hz, 4H) ppm;

^{13}C NMR (150 MHz, $CDCl_3$): δ 168.8, 168.0, 149.2, 134.3, 133.7, 123.7, 121.8, 119.2, 51.6, 47.3, 24.1, 23.8, 23.6 ppm;

IR: 2971, 2879, 1708, 1429, 1308, 1026, 867 cm^{-1};

HRMS (ESI): calculated for $[C_{13}H_{14}N_4O_2+Na]^+$: 281.1109, found: 281.1102.

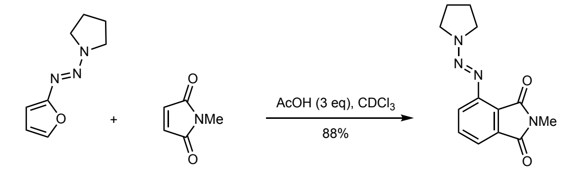

(1 eq) **2-triazenyl-furan-01** (or **1**)   (1.25 eq) **6**   **7**

To a 2-dram vial open to air and equipped with a stir bar was added **2-triazenyl-furan-01** (or **1**) (50.0 mg, 0.303 mmol, 1.00 equiv), $CDCl_3$ (0.605 mL, 0.500 M), AcOH (0.052 mL, 0.908 mmol, 3.00 equiv) and *N*-methylmaleimide (**6**) (42.0 mg, 0.378 mmol, 1.25 equiv). The vial was sealed with a screw cap and stirred at ambient temperature for 30 hours, by which time NMR analysis indicated the complete consumption of the triazenyl furan and any non-aromatized cycloadducts. *N*-methylmaleimide proved to be challenging to remove from **7**, therefore we elected to converted excess dienophile to a more easily separable species: Furan (0.440 mL, 6.05 mmol, 20.0 equiv) was added, the vial was sealed with a screw cap, wrapped with electrical tape, and warmed on a heating block at 60 °C for 5 hours, then cooled to ambient temperature and concentrated under reduced pressure. The crude residue was purified by flash column chromatography (ratio of silica to crude mass= 100:1, eluting with 5:95 EtOAc:hexane to 2:5 EtOAc:hexane).

Yield: 69.0 mg, 0.267 mmol, 88%;

Characterization data matched those reported earlier within this document.

Benchmarking

- 2-Bpin-furan: commercial (cas: 374790-93-9)
- 2-TMS-furan: prepared in 1 step according to the method of Helten.[57]
- 2-$SnBu_3$-furan: commercial (cas: 118486-94-5)
- 2-Br-furan: prepared in 1 step according to the method of Tam.[58]
- 2-OTBS-furan: prepared in 1 step according to the method of Casiraghi.[59]

Note: In our hands, **2-Br-furan** spontaneously and violently decomposed if left for several hours at ambient temperature without protection from light. If stored under a blanket of argon in the freezer (–20 °C) with K_2CO_3 as stabilizer, no decomposition was observed over several months.

Note: To simplify NMR analysis, the furan benchmarking assessment was run as two separate experiments and then combined into a single graph.

<u>Experiment 1</u>

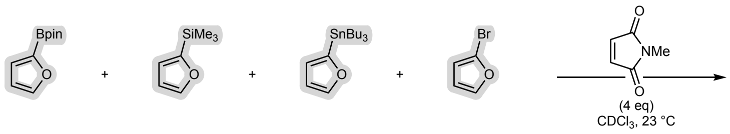

To a 2-dram vial open to air and equipped with a stir bar was added 2-Bpin-furan (69.2 mg, 0.356 mmol, 1.00 equiv), 2-TMS-furan (50.0 mg, 0.356 mmol, 1.00 equiv), 2-$SnBu_3$-furan (127 mg, 0.356 mmol, 1.00 equiv), 2-Br-furan (52.4 mg, 0.356 mmol, 1.00 equiv), 1,2,4,5-tetramethylbenzene (23.9 mg, 0.178 mmol, 0.50 equiv) as ^{1}H NMR internal standard, and $CDCl_3$ (treated with K_2CO_3, 1.19 mL, 0.300 M). An ^{1}H NMR spectrum was obtained to determine confirm the initial amount of each furan relative to the standard, and then *N*-methylmaleimide (158 mg, 1.43 mmol, 4.00 equiv) was added in one portion. The reaction was stirred at ambient temperature, and ^{1}H NMR aliquots were taken to determine the percentage of furan remaining.

<u>Experiment 2</u>

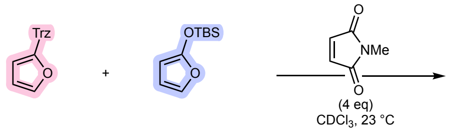

To a 2-dram vial open to air and equipped with a stir bar was added 2-triazenyl-furan (50.0 mg, 0.303 mmol, 1.00 equiv), 2-OTBS-furan (60.0 mg, 0.303 mmol, 1.00 equiv), 1,2,4,5-tetramethylbenzene (23.9 mg, 0.151 mmol, 0.50 equiv) as ^{1}H NMR internal standard, and $CDCl_3$ (treated with K_2CO_3, 1.01 mL, 0.300 M). An ^{1}H NMR spectrum was obtained to determine confirm the initial amount of each furan relative to the standard, and then *N*-

methylmaleimide (134 mg, 1.21 mmol, 4.00 equiv) was added in one portion. The reaction was stirred at ambient temperature, and ^1H NMR aliquots were taken to determine the percentage of furan remaining.

Time (minutes)	Furan Remaining (%)					
	Bpin	TMS	Sn	Br	Trz	OTBS
0	100.00	100.00	100.00	100.00	100.00	100.00
5	100.00	100.00	100.00	100.00	75.53	78.10
10	100.00	100.00	100.00	100.00	64.89	60.95
30	100.00	100.00	99.04	99.03	35.11	27.62
60	100.00	100.00	98.08	98.06	18.09	8.57
90	100.00	98.85	95.19	97.09	10.64	3.81
120	100.00	100.00	93.27	96.12	7.45	0.95
150	100.00	97.70	93.27	95.15	6.38	0.00
180	100.00	100.00	92.31	94.17	5.32	0.00
210	100.00	100.00	92.31	94.17	5.32	0.00
240	100.00	100.00	91.35	93.20	5.32	0.00

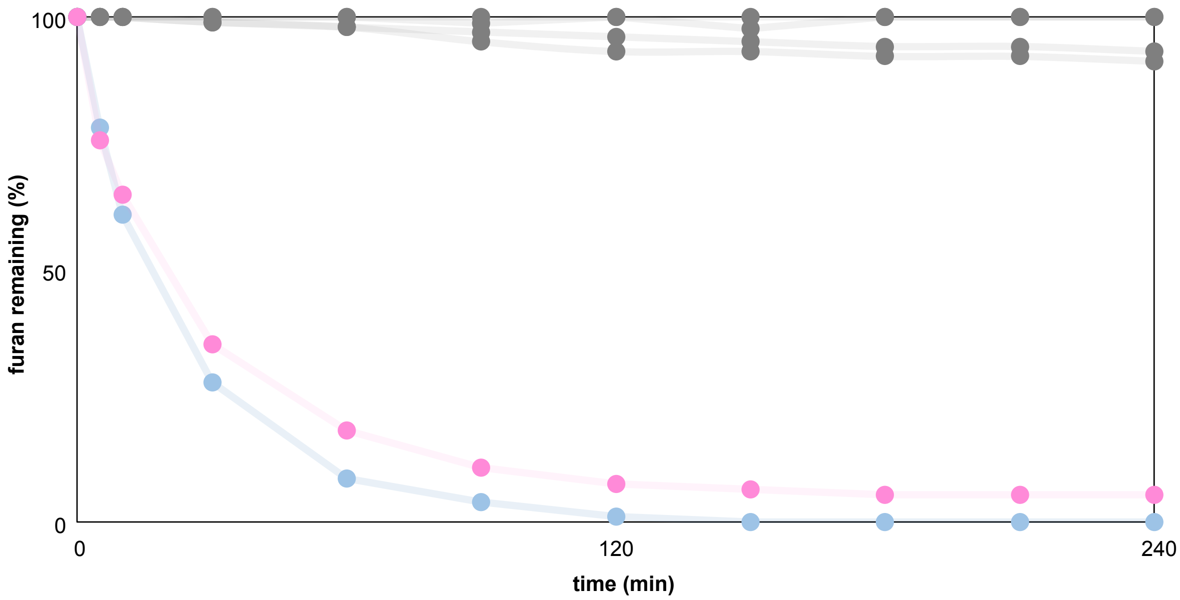

N-Phenylmaleimide as Dienophile

General Method

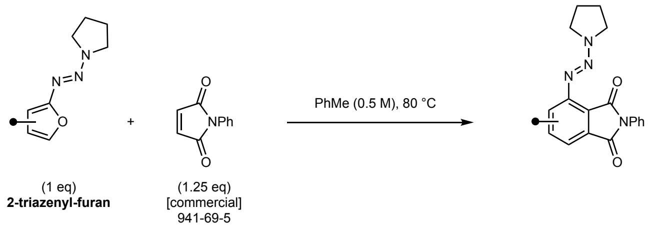

To a 2-dram vial open to air and equipped with a stir bar was added the appropriate triazenyl furan (1.00 equiv), PhMe (0.500 M), and *N*-phenylmaleimide (1.25 equiv). The vial was sealed with a screw cap, wrapped with electrical tape, and warmed on a heating block at 80 °C for 16 hours, by which time TLC indicated the complete consumption of the triazenyl furan. The reaction was cooled to ambient temperature and concentrated under reduced pressure. Further purification details and characterization data are provided for each product below.

Scope

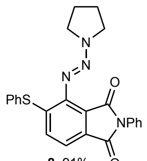

8, 91%

Prepared from **2-triazenyl-furan-02** according to the general procedure. Purified by flash column chromatography (ratio of silica to crude mass= 100:1, eluting with 1:1 CH_2Cl_2:hexane then 1:5 EtOAc:hexane).

Yield: 71.5 mg, 0.167 mmol, 91%;

Appearance: Yellow solid;

R*f*: 0.31 (1:2 EtOAc:hexane);

^{1}H NMR (400 MHz, $CDCl_3$): δ 7.59 – 7.53 (m, 2H), 7.49 – 7.34 (m, 9H), 7.03 (d, *J* = 7.8 Hz, 1H), 4.01 (t, *J* = 6.9 Hz, 2H), 3.82 (t, *J* = 7.1 Hz, 2H), 2.12 – 2.04 (m, 4H) ppm;

^{13}C NMR (100 MHz, $CDCl_3$): δ 167.2, 166.3, 147.3, 143.0, 135.3, 132.2, 134.0, 131.3, 130.1, 129.9, 129.4, 129.1, 123.0, 127.0, 120.6, 119.5, 51.5, 47.2, 24.1, 23.7 ppm;

IR: 3062, 2923, 2874, 1709, 1596, 1369, 1304, 1107, 909, 745 cm^{-1};

HRMS (ESI): calculated for $[C_{24}H_{20}N_4O_2S+H]^+$: 429.1380, found: 429.1379.

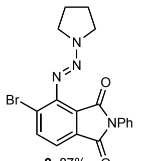

9, 87%

Prepared from **2-triazenyl-furan-03** according to the general procedure. Purified by flash column chromatography (ratio of silica to crude mass= 100:1, eluting with 1:1 CH_2Cl_2:hexane then 1:9 EtOAc:hexane).

Yield: 42.9 mg, 0.107 mmol, 87%;

Appearance: Yellow solid;

R*f*: 0.36 (1:2 EtOAc:hexane);

^{1}H NMR (400 MHz, $CDCl_3$): δ 7.95 (d, *J* = 10.5 Hz, 1H), 7.54 (d, *J* = 7.8 Hz, 1H), 7.48 (t, *J* = 7.9 Hz, 2H), 7.38 (d, *J* = 8.2 Hz, 3H), 4.00 (t, *J* = 6.8 Hz, 2H), 3.80 (t, *J* = 7.0 Hz, 2H), 2.13 – 2.05 (m, 4H) ppm;

^{13}C NMR (100 MHz, $CDCl_3$): δ 166.7, 165.7, 149.8, 138.5, 132.7, 131.9, 129.2, 128.2, 127.0, 126.0, 122.0, 120.2, 51.5, 47.3, 24.1, 23.7 ppm;

IR: 3069, 2976, 2876, 1709, 1597, 1501, 1372, 1168, 828 cm^{-1};

HRMS (ESI): calculated for $[C_{18}H_{15}N_4O_2Br+H]^+$: 399.0451, found: 399.0443.

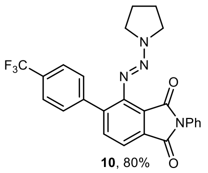

10, 80%

Prepared from **2-triazenyl-furan-04** according to the general procedure. Purified by flash column chromatography (ratio of silica to crude mass= 100:1, eluting with 1:1 CH_2Cl_2:hexane then 1:5 EtOAc:hexane).

Yield: 48.0 mg, 0.103 mmol, 80%;

Appearance: Yellow solid;

R*f*: 0.38 (1:2 EtOAc:hexane);

^{1}H NMR (400 MHz, $CDCl_3$): δ 7.78 (d, *J* = 7.5 Hz, 1H), 7.72 – 7.58 (m, 5H), 7.49 (app t, *J* = 7.6 Hz, 2H), 7.44 – 7.35 (m, 3H), 3.87 (t, *J* = 6.6 Hz, 2H), 3.55 (t, *J* = 6.8 Hz, 2H), 2.01 (p, *J* = 7.8 Hz, 4H) ppm;

^{13}C NMR (100 MHz, $CDCl_3$) δ 167.0, 166.5, 149.6, 142.2, 141.4, 136.0, 133.3, 132.1, 130.6, 129.7 (q, *J* = 32.4 Hz) 129.2, 128.2, 127.0, 124.9 (q, *J* = 3.7 Hz), 124.3 (q, *J* = 272.4 Hz), 121.8, 119.8, 51.2, 47.1, 24.0, 23.6 ppm;

^{19}F NMR (377 MHz, $CDCl_3$) δ −63.23 ppm;

IR: 2976, 2876, 1769, 1709, 1370, 1162, 835, 622 cm^{-1};

HRMS (ESI): calculated for $[C_{25}H_{19}N_4O_2F_3+H]^+$: 465.1533, found: 465.1533.

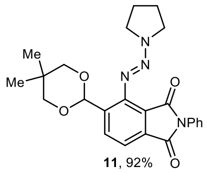

11, 92%

Prepared from **2-triazenyl-furan-05** according to the general procedure. Purified by flash column chromatography (ratio of silica to crude mass= 100:1, eluting with 1:9 EtOAc:hexane to 1:3 EtOAc:hexane).

Yield: 57.1 mg, 0.131 mmol, 92%;

Appearance: Yellow solid;

R*f*: 0.27 (2:5 EtOAc:hexane);

^{1}H NMR (400 MHz, $CDCl_3$): δ 8.11 (d, *J* = 7.4 Hz, 1H), 7.72 (d, *J* = 7.6 Hz, 1H), 7.50 – 7.43 (m, 2H), 7.42 – 7.32 (m, 3H), 5.90 (s, 1H), 3.99 (br s, 2H), 3.83 – 3.72 (m, 4H), 3.63 (d, *J* = 10.8 Hz, 2H), 2.06 (br s, 4H), 1.34 (s, 3H), 0.80 (s, 3H) ppm;

^{13}C NMR (100 MHz, $CDCl_3$): δ 167.2, 166.4, 149.3, 138.7, 134.0, 133.0, 132.2, 129.1, 128.0, 127.1, 120.8, 119.6, 97.2, 78.0, 51.3, 46.9, 30.4, 24.1, 23.7, 23.3, 22.0 ppm;

IR: 2953, 2870, 1711, 1501, 1367, 1097, 915 cm^{-1};

HRMS (ESI): calculated for $[C_{24}H_{26}N_4O_4+H]^+$: 435.2027, found: 435.2020.

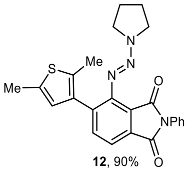

12, 90%

Prepared from **2-triazenyl-furan-06** according to the general procedure, but with flipped stoichiometry (to aid purification). Purified by flash column chromatography (ratio of silica to crude mass= 100:1, eluting with 5:95 EtOAc:hexane to 1:2 EtOAc:hexane).

Yield: 60.0 mg, 0.139 mmol, 90%;

Appearance: Yellow solid;

R*f*: 0.40 (1:2 EtOAc:hexane);

^{1}H NMR (900 MHz, $CDCl_3$): δ 7.70 (d, *J* = 7.4 Hz, 1H), 7.58 (d, *J* = 7.4 Hz, 1H), 7.48 (t, *J* = 7.9 Hz, 2H), 7.41 (d, *J* = 1.1 Hz, 2H), 7.37 (t, *J* = 7.4 Hz, 1H), 6.60 (s, 1H), 3.86 (s, 2H), 3.58 (s, 2H), 2.43 (s, 3H), 2.26 (s, 3H), 2.00 (app d, *J* = 23.7 Hz, 4H) ppm;

^{13}C NMR (225 MHz, $CDCl_3$): δ 167.3, 166.7, 145.0, 138.5, 136.5, 135.0, 134.8, 134.2, 132.3, 132.3, 129.2, 128.3, 128.0, 127.1, 121.6, 119.4, 51.1, 46.8, 24.1, 23.7, 15.3, 14.5 ppm;

IR: 2919, 2873, 1713, 1373, 1112, 912, 760 cm^{-1};

HRMS (ESI): calculated for $[C_{24}H_{22}N_4O_2S+H]^+$: 431.1536, found: 431.1539.

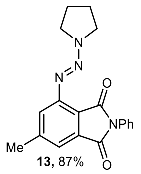

13, 87%

Prepared from **2-triazenyl-furan-07** according to the general procedure, but rather than concentrating the reaction under reduced pressure, the reaction mixture was filtered and the solid washed with a small amount of PhMe (ca. 1 mL).

Yield: 66.3 mg, 0.198 mmol, 87%;

Appearance: Yellow solid;

R*f*: 0.35 (2:5 EtOAc:hexane);

^{1}H NMR (400 MHz, $CDCl_3$): δ 7.56 (s, 1H), 7.52 – 7.40 (m, 5H), 7.36 (t, *J* = 7.9 Hz, 1H), 3.99 (t, *J* = 6.7 Hz, 2H), 3.82 (t, *J* = 6.8 Hz, 2H), 2.49 (s, 3H), 2.06 (s, 4H) ppm;

^{13}C NMR (100 MHz, $CDCl_3$): δ 167.8, 166.7, 149.5, 146.1, 133.5, 132.3, 129.0, 127.8, 126.9, 124.1, 120.7, 119.0, 51.6, 47.3, 24.1, 23.6, 22.2 ppm;

IR: 2925, 1712, 1363, 1305, 1221, 1097, 757 cm^{-1};

HRMS (ESI): calculated for $[C_{19}H_{18}N_4O_2+H]^+$: 335.1503, found: 335.1496

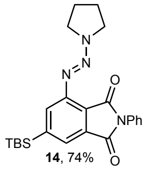

14, 74%

Prepared from **2-triazenyl-furan-08** according to the general procedure. Purified by flash column chromatography (ratio of silica to crude mass= 100:1, eluting with 2:5 CH_2Cl_2:hexane then 1:5 EtOAc:hexane).

Yield: 29.0 mg, 0.0667 mmol, 74%;

Appearance: Yellow solid;

R*f*: 0.36 (1:2 EtOAc:hexane);

^{1}H NMR (400 MHz, $CDCl_3$): δ 7.86 (s, 1H), 7.82 (s, 1H), 7.51 – 7.41 (m, 4H), 7.37 (t, *J* = 7.9 Hz, 1H), 4.02 (t, *J* = 6.8 Hz, 2H), 3.83 (t, *J* = 6.8 Hz, 2H), 2.07 (s, 4H), 0.91 (s, 9H), 0.35 (s, 6H) ppm;

^{13}C NMR (100 MHz, $CDCl_3$): δ 168.2, 166.9, 148.3, 147.8, 132.3, 131.8, 130.7, 129.1, 127.9, 127.0, 125.2, 121.5, 51.6, 47.3, 26.6, 24.1, 23.6, 17.1, −6.0 ppm;

IR: 2951, 2854, 1704, 1500, 1336, 913, 753 cm^{-1};

HRMS (ESI): calculated for $[C_{24}H_{30}N_4O_2Si+H]^+$: 435.2211, found: 435.2210.

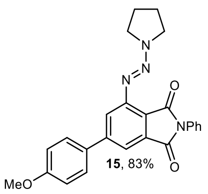

15, 83%

Prepared from **2-triazenyl-furan-09** according to the general procedure. Purified by flash column chromatography (ratio of silica to crude mass= 100:1, eluting with 1:5 EtOAc/hexane).

Yield: 65.3 mg, 0.153 mmol, 83%;

Appearance: Yellow solid;

R*f*: 0.15 (1:2 EtOAc:hexane);

^{1}H NMR (400 MHz, $CDCl_3$): δ 7.74 (d, *J* = 9.0 Hz, 1H), 7.69 (d, *J* = 7.5 Hz, 1H), 7.51 – 7.33 (m, 7H), 6.94 (d, *J* = 7.8 Hz, 2H), 3.90 (s, 2H), 3.86 (s, 3H), 3.59 (s, 2H), 2.01 (s, 4H) ppm;

^{13}C NMR (100 MHz, $CDCl_3$): δ 167.2, 166.7, 159.4, 149.5, 142.6, 135.7, 132.2, 132.0, 131.5, 130.7, 129.1, 128.0, 127.0, 121.6, 119.8, 113.4, 55.4, 51.0, 46.9, 24.1, 23.6 ppm;

IR: 3061, 2953, 2873, 1708, 1515, 1365, 1027, 828, 732 cm^{-1};

HRMS (ESI): calculated for $[C_{25}H_{22}N_4O_3+H]^+$: 427.1765, found: 427.1757.

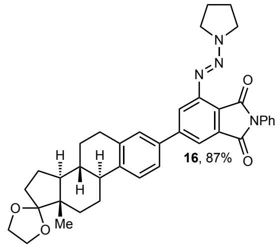

16, 87%

Prepared from **2-triazenyl-furan-10** according to the general procedure. Purified by flash column chromatography (ratio of silica to crude mass= 100:1, eluting with 5:95 EtOAc:hexane to 1:3 EtOAc:hexane).

Yield: 58.0 mg, 0.0940 mmol, 87%;

Appearance: Yellow solid;

R*f*: 0.22 (1:2 EtOAc:hexane);

^{1}H NMR (600 MHz, $CDCl_3$): δ 7.73 (q, *J* = 25.8 Hz, 2H), 7.48 (t, *J* = 7.7 Hz, 2H), 7.42 – 7.39 (m, 2H), 7.37 (t, *J* = 20.3 Hz, 1H), 7.34 – 7.31 (m, 2H), 7.29 (s, 1H), 4.00 – 3.87 (m, 6H), 3.61 (br s, 2H), 2.93 – 2.87 (m, 2H), 2.42 – 2.31 (m, 2H), 2.08 – 1.93 (m, 6H), 1.90 – 1.77 (m, 3H), 1.70 – 1.64 (m, 1H), 1.58 – 1.44 (m, 3H), 1.44 – 1.36 (m, 2H), 0.91 (s, 3H) ppm;

^{13}C NMR (150 MHz, $CDCl_3$): δ 167.3, 166.8, 149.7, 142.8, 140.2, 136.4, 135.9, 135.4, 132.3, 132.2, 130.8, 129.1, 128.0, 127.7, 127.1, 124.9, 121.7, 119.9, 119.5, 65.4, 64.7, 51.1, 49.6, 47.0, 46.3, 44.2, 39.0, 34.4, 30.9, 29.8, 27.1, 26.1, 24.1, 23.7, 22.5, 14.5 ppm;

IR: 2934, 2870, 1710, 1367, 1306, 1103, 907, 726 cm^{-1};

HRMS (ESI): calculated for $[C_{38}H_{40}N_4O_4+H]^+$: 617.3122, found: 617.3123.

[α]D[20]: +15.9 (c = 0.44, $CHCl_3$).

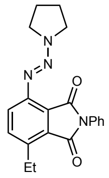

17, 72%

Prepared from **2-triazenyl-furan-11** according to the general procedure. Purified by flash column chromatography (ratio of silica to crude mass= 100:1, eluting with 5% EtOAc:hexane to 1:2 EtOAc:hexane).

Yield: 39.0 mg, 0.112 mmol, 72%;

Appearance: Yellow solid;

R*f*: 0.35 (2:5 EtOAc:hexane);

^{1}H NMR (400 MHz, $CDCl_3$): δ 7.68 (d, *J* = 8.6 Hz, 1H), 7.50 – 7.40 (m, 5H), 7.36 (t, *J* = 7.8 Hz, 1H), 3.98 (t, *J* = 6.5 Hz, 2H), 3.82 (t, *J* = 6.8 Hz, 2H), 3.15 (q, *J* = 7.5 Hz, 2H), 2.06 (s, 4H), 1.30 (t, *J* = 7.5 Hz, 3H);

^{13}C NMR (100 MHz, $CDCl_3$): δ 168.1, 166.7, 148.0, 141.1, 136.1, 132.2, 129.0, 128.4, 127.8, 127.1, 124.2, 121.6, 51.6, 47.3, 24.5, 24.1, 23.6, 15.2 ppm;

IR: 2970, 2872, 1710, 1501, 1393, 1112, 881 cm^{-1};

HRMS (ESI): calculated for $[C_{20}H_{20}N_4O_2+Na]^+$: 371.1478, found: 371.1474.

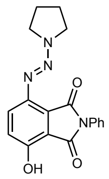

18, 89%

Prepared from **2-triazenyl-furan-12** according to the general procedure, but rather than concentrating the reaction under reduced pressure, the reaction mixture was filtered and the solid washed with a small amount of Et_2O (ca. 1 mL).

Yield: 61.0 mg, 0.181 mmol, 89%;

Appearance: Yellow solid;

R*f*: 0.61 (EtOAc);

^{1}H NMR (900 MHz, $CDCl_3$): δ 7.93 (s, 1H), 7.76 (d, *J* = 9.0 Hz, 1H), 7.49 (t, *J* = 7.9 Hz, 2H), 7.44 (d, *J* = 7.1 Hz, 2H), 7.38 (t, *J* = 7.4 Hz, 1H), 3.97 (t, *J* = 6.9 Hz, 2H), 3.79 (t, *J* = 7.2 Hz, 2H), 2.06 (dq, *J* = 24.9, 6.7 Hz, 4H);

^{13}C NMR (225 MHz, $CDCl_3$): δ 169.9, 165.9, 152.8, 143.8, 131.7, 129.2, 128.1, 127.5, 126.7, 124.2, 119.6, 113.7, 51.5, 47.2, 24.1, 23.6 ppm;

IR: 3390, 2972, 1704, 1372, 1103, 883 cm^{-1};

HRMS (ESI): calculated for $[C_{18}H_{16}N_4O_3+H]^+$: 337.1295, found: 337.1291.

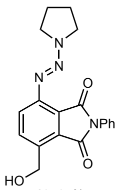

19, 84%

Prepared from **2-triazenyl-furan-27** according to the general procedure. Purified by flash column chromatography (ratio of silica to crude mass= 100:1, eluting with 1:5 EtOAc:hexane to 2:5 EtOAc:hexane).

Yield: 45.0 mg, 0.128 mmol, 84%;

Appearance: Yellow solid;

R*f*: 0.17 (2:5 EtOAc:hexane);

^{1}H NMR (400 MHz, $CDCl_3$): δ 7.75 (d, *J* = 8.4 Hz, 1H), 7.55 (d, *J* = 8.3 Hz, 1H), 7.50 (t, *J* = 7.7 Hz, 2H), 7.45 – 7.37 (m, 3H), 4.94 (s, 2H), 4.09 (br s, 1H), 4.00 (t, *J* = 6.5 Hz, 2H), 3.83 (t, *J* = 6.7 Hz, 2H), 2.07 (q, *J* = 6.8 Hz, 4H) ppm;

^{13}C NMR (100 MHz, $CDCl_3$): δ 169.4, 166.5, 149.4, 137.1, 134.8, 131.8, 129.8, 129.2, 128.3, 127.0, 124.2, 121.6, 62.6, 51.7, 47.5, 24.1, 23.6 ppm;

IR: 3440, 3062, 2931, 2869, 1702, 1372, 1209, 909, 695 cm^{-1};

HRMS (ESI): calculated for $[C_{19}H_{18}N_4O_3+H]^+$: 351.1452, found: 351.1445.

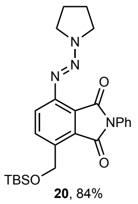

20, 84%

Prepared from **2-triazenyl-furan-28** according to the general procedure. Purified by flash column chromatography (ratio of silica to crude mass= 100:1, eluting with 5:95 EtOAc:hexane).

Yield: 182 mg, 0.392 mmol, 84%;

Appearance: Yellow solid;

R*f*: 0.39 (2:5 EtOAc:hexane);

^{1}H NMR (900 MHz, $CDCl_3$): δ 7.89 (d, *J* = 8.7 Hz, 1H), 7.78 (d, *J* = 8.6 Hz, 1H), 7.47 (t, *J* = 7.8 Hz, 2H), 7.43 (d, *J* = 8.3 Hz, 2H), 7.36 (t, *J* = 8.0 Hz, 1H), 5.26 (s, 2H), 3.97 (s, 2H), 3.81 (s, 2H), 2.04 (dt, *J* = 21.7, 6.5 Hz, 4H), 0.97 (s, 9H), 0.15 (s, 6H) ppm;

^{13}**C NMR** (225MHz, $CDCl_3$): δ 167.9, 166. 7, 148.4, 138.0, 132.8, 132.1, 129.0, 127.2, 127.0, 126.9, 124.2, 120.8, 60.8, 51.5, 47.3, 26.1, 24.0, 23.5, 18.7, −5.12 ppm;

IR: 2953, 2855, 1704, 1501, 1365, 1097, 833, 726 cm^{-1};

HRMS (ESI): calculated for $[C_{25}H_{32}N_4O_3Si+H]^+$: 465.2316, found: 465.2316.

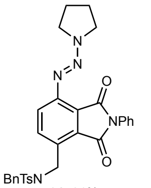

21, 81%

Prepared from **2-triazenyl-furan-24** according to the general procedure. Purified by flash column chromatography (ratio of silica to crude mass= 100:1, eluting with 5:95 EtOAc:hexane to 2:5 EtOAc:hexane).

Yield: 53.0 mg, 0.0893 mmol, 81%;

Appearance: Yellow solid;

R*f*: 0.23 (2:5 EtOAc:hexane);

^{1}**H NMR** (900 MHz, $CDCl_3$): δ 7.79 (d, *J* = 8.6 Hz, 1H), 7.75 (d, *J* = 8.3 Hz, 2H), 7.63 (d, *J* = 8.6 Hz, 1H), 7.46 (t, *J* = 7.9 Hz, 2H), 7.37 – 7.33 (m, 5H), 7.08 – 7.03 (m, 5H), 4.86 (s, 2H), 4.37 (s, 2H), 3.99 (t, *J* = 6.7 Hz, 2H), 3.80 (t, *J* = 6.9 Hz, 2H), 2.45 (s, 3H), 2.09 – 2.03 (m, 4H) ppm;

^{13}**C NMR** (225 MHz, $CDCl_3$): δ 167.8, 166.1, 148.7, 143.8, 136.3, 136.2, 135.4, 133.3, 132.0, 130.0, 129.0, 129.0, 128.5, 128.2, 127.9, 127.8, 127.4, 126.8, 123.9, 120.3, 54.3, 51.6, 47.4, 46.8, 24.1, 23.6, 21.7 ppm;

IR: 3031, 2923, 2874, 1706, 1375, 1159, 913, 732 cm^{-1};

HRMS (ESI): calculated for $[C_{33}H_{31}N_5O_4S+Na]^+$: 616.1989, found: 616.1985.

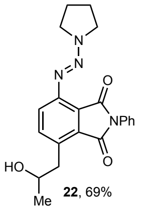

22, 69%

Prepared from **2-triazenyl-furan-14** according to the general procedure. Purified by flash column chromatography (ratio of silica to crude mass= 100:1, eluting with 1:1 EtOAc:hexane).

Yield: 70.0 mg, 0.185 mmol, 69%;

Appearance: Yellow solid;

R*f*: 0.12 (2:5 EtOAc:hexane);

^{1}H NMR (400 MHz, $CDCl_3$): δ 7.70 (d, *J* = 8.4 Hz, 1H), 7.43 (m, 6H), 4.15 (q, *J* = 5.9 Hz, 1H), 3.99 (t, *J* = 6.6 Hz, 2H), 3.82 (t, *J* = 6.7 Hz, 2H), 3.35 (dd, *J* = 13.2, 4.5 Hz, 1H), 3.13 (dd, *J* = 13.3, 7.6 Hz, 1H), 2.06 (s, 4H), 1.28 (d, *J* = 6.2 Hz, 3H) ppm;

^{13}C NMR (100 MHz, $CDCl_3$): δ 169.1, 166.4, 148.6, 138.0, 135.1, 132.0, 129.8, 129.1, 128.0, 127.0, 124.0, 121.4, 69.0, 51.6, 47.4, 40.8, 24.1, 23.6, 23.3 ppm;

IR: 3506, 2970, 2875, 1707, 1502, 1379, 1113, 883 cm^{-1};

HRMS (ESI): calculated for $[C_{21}H_{22}N_4O_3+H]^+$: 379.1765, found: 379.1759.

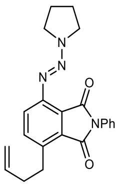

23, 80%

Prepared from **2-triazenyl-furan-15** according to the general procedure. Purified by flash column chromatography (ratio of silica to crude mass= 100:1, eluting with 1:1 CH_2Cl_2:hexane then 1:5 EtOAc:hexane).

Yield: 58.8 mg, 0.157 mmol, 80%;

Appearance: Yellow solid;

R*f*: 0.43 (2:5 EtOAc:hexane);

^{1}H NMR (400 MHz, $CDCl_3$): δ 7.67 (d, *J* = 8.4 Hz, 1H), 7.50 – 7.34 (m, 6H), 5.94 – 5.82 (m, 1H), 4.99 (dd, *J* = 21.0, 13.6 Hz, 2H), 3.97 (t, *J* = 6.7 Hz, 2H), 3.82 (t, *J* = 6.8 Hz, 2H), 3.20 (t, *J* = 7.7 Hz, 2H), 2.43 (q, *J* = 7.3 Hz, 2H), 2.05 (s, 4H) ppm;

^{13}C NMR (100 MHz, $CDCl_3$): δ 168.0, 166.6, 148.1, 138.5, 137.7, 136.8, 132.2, 129.0, 128.8, 127.8, 127.0, 123.9, 121.5, 115.5, 51.50, 47.3, 35.0, 30.7, 24.1, 23.6 ppm;

IR: 3071, 2973, 2874, 1704, 1500, 1359, 1103, 881, 691 cm^{-1};

HRMS (ESI): calculated for $[C_{22}H_{22}N_4O_2+H]^+$: 375.1816, found: 375.1809.

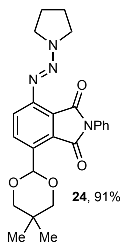

24, 91%

Prepared from **2-triazenyl-furan-16** according to the general procedure. Purified by flash column chromatography (ratio of silica to crude mass= 100:1, eluting with 1:9 EtOAc:hexane to 1:3 EtOAc:hexane).

Yield: 170 mg, 0.391 mmol, 91%;

Appearance: Off-white solid;

R*f*: 0.38 (2:5 EtOAc:hexane);

^{1}H NMR (400 MHz, $CDCl_3$): δ 8.02 (d, *J* = 8.6 Hz, 1H), 7.82 (d, *J* = 8.6 Hz, 1H), 7.51 – 7.34 (m, 5H), 6.43 (s, 1H), 4.01 (s, 2H), 3.84 (s, 2H), 3.77 (s, 4H), 2.07 (s, 4H), 1.34 (s, 3H), 0.81 (s, 3H) ppm;

^{13}C NMR (100 MHz, $CDCl_3$): δ 167.1, 166.4, 150.3, 133.1, 133.0, 132.1, 129.0, 128.9, 128.0, 127.1, 124.2, 120.9, 96.5, 78.1, 51.7, 47.5, 30.5, 24.1, 23.6, 23.4, 22.0 ppm;

IR: 2954, 2870, 1716, 1377, 1101, 985 cm^{-1};

HRMS (ESI): calculated for $[C_{24}H_{26}N_4O_4+H]^+$: 435.2027, found: 435.2028.

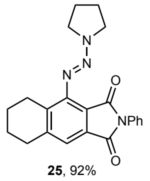

25, 92%

Prepared from **2-triazenyl-furan-17** according to the general procedure. Purified by flash column chromatography (ratio of silica to crude mass= 100:1, eluting with 5:95 EtOAc hexane to 1:9 EtOAc:hexane).

Yield: 23.8 mg, 0.0636 mmol, 92%;

Appearance: Yellow solid;

R*f*: 0.39 (1:2 EtOAc:hexane);

^{1}H NMR (400 MHz, $CDCl_3$): δ 7.48 – 7.44 (m, 3H), 7.39 – 7.32 (m, 3H), 3.96 (s, 2H), 3.73 (s, 2H), 2.92 (s, 2H), 2.82 (s, 2H), 2.05 (s, 4H), 1.83 (s, 4H) ppm;

^{13}C NMR (100 MHz, $CDCl_3$): δ 167.8, 167.0, 150.0, 144.9, 138.9, 132.4, 130.3, 129.1, 127.8, 127.1, 120.9, 118.0, 51.2, 46.7, 30.8, 25.8, 24.1, 23.8, 22.7, 22.4 ppm;

IR: 2930, 2871, 1707, 1501, 1375, 1137, 910 cm^{-1};

HRMS (ESI): calculated for $[C_{22}H_{22}N_4O_2+H]^+$: 375.1816, found: 375.1810.

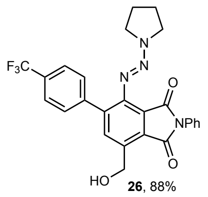

26, 88%

Prepared from **2-triazenyl-furan-39** according to the general procedure. Purified by flash column chromatography (ratio of silica to crude mass= 100:1, eluting with 1:9 EtOAc:hexane to 1:5 EtOAc:hexane).

Yield: 54.1 mg, 0.109 mmol, 88%;

Appearance: Yellow solid;

R*f*: 0.24 (2:5 EtOAc:hexane);

^{1}H NMR (400 MHz, $CDCl_3$): δ 7.66 (d, *J* = 8.1 Hz, 2H), 7.61 (d, *J* = 8.9 Hz, 3H), 7.51 (t, *J* = 7.2 Hz, 2H), 7.40 (t, *J* = 7.2 Hz, 3H), 5.00 (s, 2H), 3.99 (s, 1H), 3.85 (s, 2H), 3.55 (s, 2H), 2.00 (s, 4H) ppm;

^{13}C NMR (100 MHz, $CDCl_3$): *δ* 168.6, 166.3, 149.1, 142.0, 141.5, 137.1, 135.7, 131.7, 130.5, 129.8 (q, *J* = 30.7 Hz), 129.7, 129.2, 128.4, 127.1, 124.9 (q, *J* = 3.9 Hz), 124.2 (q, *J* = 274.6 Hz), 122.3, 62.3, 51.2, 47.1, 24.0, 23.6 ppm;

^{19}F NMR (377 MHz, $CDCl_3$): δ −63.26 ppm;

IR: 3457, 2976, 2877, 1707, 1502, 1324, 1113, 909, 843 cm^{-1};

HRMS (ESI): calculated for $[C_{26}H_{21}N_4O_3F_3+H]^+$: 495.1639, found: 495.1635.

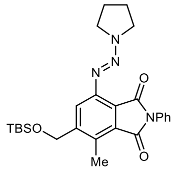

27, 96%

Prepared from **2-triazenyl-furan-18** according to the general procedure, but with flipped stoichiometry (to aid purification). Purified by flash column chromatography (ratio of silica to crude mass= 100:1, eluting with 5:95 EtOAc:hexane).

Yield:115 mg, 0.240 mmol, 96%;

Appearance: Yellow solid;

R*f*: 0.50 (2:5 EtOAc:hexane);

^{1}H NMR (400 MHz, $CDCl_3$): δ 7.95 (s, 1H), 7.50 – 7.33 (m, 5H), 4.80 (s, 2H), 3.90 (d, *J* = 33.1 Hz, 4H), 2.64 (s, 3H), 2.06 (s, 4H), 0.97 (s, 9H), 0.14 (s, 6H) ppm;

^{13}C NMR (100 MHz, $CDCl_3$): δ 168.6, 166.5, 148.2, 147.7, 132.3, 132.0, 129.1, 129.0, 127.8, 127.1, 121.5, 120.0, 62.9, 51.5, 47.1, 26.0, 24.0, 23.6, 18.5, 12.6, −5.19 ppm;

IR: 2952, 2856, 1704, 1502, 1360, 1113, 1061, 942 cm^{-1};

HRMS (ESI): calculated for $[C_{26}H_{34}N_4O_3Si+H]^+$: 479.2473, found: 479.2472.

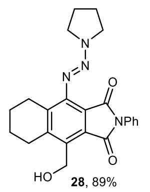

28, 89%

Prepared from **2-triazenyl-furan-37** according to the general procedure. Purified by flash column chromatography (ratio of silica to crude mass= 100:1, eluting with 1:5 EtOAc:hexane to 2:5 EtOAc:hexane).

Yield: 94.9 mg, 0.235 mmol, 89%;

Appearance: Yellow solid;

R*f*: 0.15 (1:2 EtOAc:hexane);

^{1}H NMR (400 MHz, $CDCl_3$): δ 7.47 (t, *J* = 7.4 Hz, 2H), 7.40 – 7.32 (m, 3H), 4.99 (d, *J* = 5.4 Hz, 2H), 4.04 (d, *J* = 7.4 Hz, 1H), 3.94 (s, 2H), 3.73 (s, 2H), 2.95 (t, *J* = 5.9 Hz, 2H), 2.83 (t, *J* = 6.4 Hz, 2H), 2.05 (s, 4H), 1.80 (dq, *J* = 24.8, 6.8 Hz, 4H) ppm;

^{13}C NMR (100 MHz, $CDCl_3$): δ 169.7, 166.6, 149.6, 143.1, 139.9, 136.0, 132.0, 129.1, 128.1, 127.6, 127.2, 118.2, 57.3, 51.1, 46.7, 27.3, 26.5, 24.1, 23.7, 22.5, 21.9 ppm;

IR: 3440, 2931, 2869, 1702, 1501, 1372, 1209, 1026, 960 cm^{-1};

HRMS (ESI): calculated for $[C_{23}H_{24}N_4O_3+Na]^+$: 427.1741, found: 427.1736.

Dimethyl Acetylenedicarboxylate as Dienophile

General Method

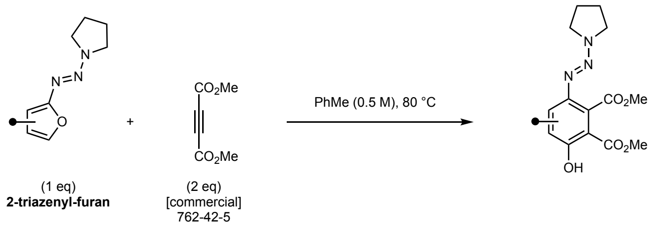

To a 2-dram vial open to air and equipped with a stir bar was added the appropriate triazenyl furan (1.00 equiv), PhMe (0.500 M), and dimethyl acetylenedicarboxylate (2.00 equiv). The reaction flask was sealed with a screw cap, wrapped with electrical tape, and warmed on a heating block at 80 °C for 2 hours, by which time TLC indicated the complete consumption of the triazenyl furan. The reaction was cooled to ambient temperature and concentrated under reduced pressure. Further purification details and characterization data are provided for each product below.

Scope

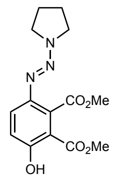

29, 91% (g scale)

Prepared from **2-triazenyl-furan-01** (or **1**) according to the general procedure. Purified by flash column chromatography (ratio of silica to crude mass= 50:1, eluting with 1:5 EtOAc:hexane to 2:5 EtOAc:hexane).

Yield: 1.69 g, 5.48 mmol, 91%;

Appearance: Yellow solid;

R*f*: 0.25 (2:5 EtOAc:hexane);

^{1}H NMR (400 MHz, $CDCl_3$) δ 10.93 (s, 1H), 7.73 (d, *J* = 10.4 Hz, 1H), 6.99 (d, *J* = 9.2 Hz, 1H), 3.98 – 3.79 (m, 8H), 3.57 (br s, 2H), 1.99 (br s, 4H) ppm;

^{13}C NMR (100 MHz, $CDCl_3$): δ 169.7, 169.1, 159.6, 141.2, 130.0, 124.5, 119.6, 108.5, 53.0, 52.4, 51.0, 46.4, 23.9, 23.7 ppm;

IR: 3150, 2951, 2873, 1732, 1672, 1410, 1314, 1207, 1016, 911 cm^{-1};

HRMS (ESI): calculated for $[C_{14}H_{17}N_3O_5+H]^+$: 308.1241, found: 308.1229.

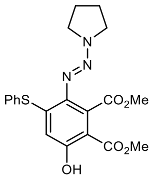

30, 91%

Prepared from **2-triazenyl-furan-02** according to the general procedure. Purified by flash column chromatography (ratio of silica to crude mass= 50:1, eluting with 1:5 EtOAc:hexane).

Yield: 55.3 mg, 0.133 mmol, 91%;

Appearance: Orange solid;

R*f*: 0.35 (2:5 EtOAc:hexane);

^{1}H NMR (400 MHz, $CDCl_3$): δ 10.86 (s, 1H), 7.54 (s, 2H), 7.43 (s, 3H), 6.33 (s, 1H), 3.93 (s, 2H), 3.88 (s, 3H), 3.84 (s, 3H), 3.69 – 3.61 (m, 2H), 2.03 (s, 4H) ppm;

^{13}C NMR (100 MHz, $CDCl_3$): δ 169.7, 168.8, 158.8, 145.4, 136.8, 135.9, 131.5, 130.0, 129.7, 126.0, 115.5, 106.1, 52.9, 52.0, 51.1, 46.6, 24.0, 23.7 ppm;

IR: 2949, 2873, 1734, 1667, 1581, 1314, 1223, 937, 731 cm^{-1};

HRMS (ESI): calculated for $[C_{20}H_{21}N_3O_5S+H]^+$: 416.1275, found: 416.1258.

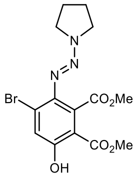

31, 80%

Prepared from **2-triazenyl-furan-03** according to the general procedure. Purified by flash column chromatography (ratio of silica to crude mass= 50:1, eluting with 2:5 EtOAc:hexane).

Yield: 222 mg, 0.575 mmol, 80%;

Appearance: Yellow solid;

R*f*: 0.20 (2:5 EtOAc:hexane);

^{1}H NMR (900 MHz, $CDCl_3$): δ 10.78 (s, 1H), 7.36 (s, 1H), 3.91 (s, 3H),), 3.88 (br s, 2H), 3.80 (s, 3H), 3.64 (br s, 2H), 2.05 (br s, 2H), 2.02 (br s, 2H) ppm;

^{13}C NMR (225 MHz, $CDCl_3$): δ 169.4, 167.9, 158.7, 139.3, 127.2, 126.7, 123.6, 109.4, 53.2, 52.0, 51.2, 46.7, 24.1, 23.6 ppm;

IR: 2950, 2875, 1735, 1675, 1579, 1314, 1213, 1043, 930 cm^{-1};

HRMS (ESI): calculated for $[C_{14}H_{16}N_3O_5Br+H]^+$: 386.0346, found: 386.0331.

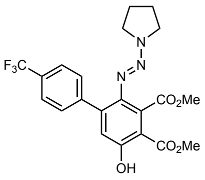

32, 72%

Prepared from **2-triazenyl-furan-04** according to the general procedure, but at 60 °C. Purified by flash column chromatography (ratio of silica to crude mass= 50:1, eluting with 1:5 EtOAc:hexane to 2:5 EtOAc:hexane).

Yield: 42.0 g, 0.0930 mmol, 72%;

Appearance: Yellow solid;

R*f*: 0.21 (2:5 EtOAc:hexane);

^{1}H NMR (900 MHz, $CDCl_3$): δ 10.81 (s, 1H), 7.58 (d, *J* = 8.1 Hz, 2H), 7.48 (d, *J* = 8.0 Hz, 2H), 7.02 (s, 1H), 3.95 (s, 3H), 3.83 (s, 3H), 3.60 (br s, 2H), 3.35 (br s, 2H), 1.92 (br s, 2H), 1.89 (br s, 2H) ppm;

^{13}C NMR (225 MHz, $CDCl_3$): δ 169.5, 168.8, 158.8, 143.0, 142.6, 139.3, 130.2, 129.4 (q, *J* = 32.3 Hz), 127.5, 125.0, 124.6 (d, *J* = 4.3 Hz), 123.8, 120.8, 109.6, 53.2, 52.0, 50.9, 46.4, 24.0, 23.6 ppm;

^{19}F NMR (377 MHz, $CDCl_3$): δ −63.18 ppm;

IR: 2952, 1736, 1677, 1418, 1323, 1231, 1121, 1067, 847 cm^{-1};

HRMS (ESI): calculated for $[C_{21}H_{20}N_3O_5F_3+H]^+$: 452.1428, found: 452.1415.

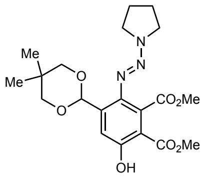

33, 71%

Prepared from **2-triazenyl-furan-05** according to the general procedure. Purified by flash column chromatography (ratio of silica to crude mass= 50:1, eluting with 1:3 EtOAc:hexane).

Yield: 43.0 mg, 0.102 mmol, 71%;

Appearance: Yellow solid;

R*f*: 0.24 (2:5 EtOAc:hexane);

^{1}H NMR (900 MHz, $CDCl_3$): δ 10.69 (d, *J* = 2.3 Hz, 1H), 7.46 (d, *J* = 3.5 Hz, 1H), 5.83 (s, 1H), 3.89 (d, *J* = 3.4 Hz, 3H), 3.85 (s, 2H), 3.78 (d, *J* = 2.9 Hz, 3H), 3.72 (d, *J* = 12.9 Hz, 2H), 3.58 (s, 2H), 3.55 (d, *J* = 11.2 Hz, 2H), 2.01 (s, 4H), 1.28 (d, *J* = 2.7 Hz, 3H), 0.76 (d, *J* = 3.0 Hz, 3H) ppm;

^{13}C NMR (225 MHz, $CDCl_3$): δ 169.5, 168.9, 159.0, 139.8, 138.3, 126.4, 117.5, 110.2, 97.4, 77.9, 53.0, 51.8, 51.0, 46.3, 30.4, 24.0, 23.7, 23.2, 22.0 ppm;

IR: 3100, 2952, 2869, 1731, 1673, 1313, 1185, 1103, 987 cm^{-1};

HRMS (ESI): calculated for $[C_{20}H_{27}N_3O_7+H]^+$: 422.1922, found: 422.1910.

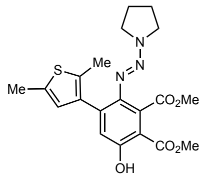

34, 93%

Prepared from **2-triazenyl-furan-06** according to the general procedure. Purified by flash column chromatography (ratio of silica to crude mass= 50:1, eluting with 1:9 EtOAc:hexane to 1:4 EtOAc:hexane).

Yield: 87.0 mg, 0.208 mmol, 93%;

Appearance: Orange oil;

R*f*: 0.41 (2:5 EtOAc:hexane);

^{1}H NMR (400 MHz, $CDCl_3$): δ 10.77 (s, 1H), 6.92 (s, 1H), 6.50 (s, 1H), 3.92 (s, 3H), 3.82 (s, 3H), 3.50 (br s, 4H), 2.37 (s, 3H), 2.20 (s, 3H), 1.91 (s, 4H) ppm;

^{13}C NMR (100 MHz, $CDCl_3$): δ 169.6, 168.9, 158.4, 140.2, 139.4, 135.2, 134.3, 133.4, 128.1, 127.4, 121.0, 108.6, 53.0, 51.9, 50.8, 46.2, 23.8 (2 coincident peaks), 15.2, 14.2 ppm;

IR: 3120, 2949, 2871, 1735, 1673, 1433, 1315, 1206, 1023, 807 cm^{-1};

HRMS (ESI): calculated for $[C_{20}H_{23}N_3O_5S+H]^+$: 418.1413, found: 418.1425.

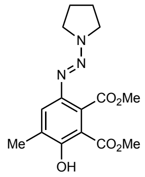

35, 91%

Prepared from **2-triazenyl-furan-07** according to the general procedure. Purified by flash column chromatography (ratio of silica to crude mass= 50:1, eluting with 1:9 EtOAc:hexane).

Yield: 49.0 mg, 0.152 mmol, 91%;

Appearance: Yellow solid;

R*f*: 0.23 (1:2 EtOAc:hexane);

^{1}H NMR (400 MHz, $CDCl_3$): δ 11.23 (s, 1H), 7.60 (s, 1H), 3.91 (s, 4H), 3.88 (s, 4H), 3.57 (br s, 2H), 2.27 (br s, 3H), 1.99 (br s, 4H) ppm;

^{13}C NMR (100 MHz, $CDCl_3$): δ 170.2, 169.3, 158.4, 140.2, 129.0, 127.7, 124.9, 107.8, 52.9, 52.3, 50.8, 46.1, 24.0, 23.9, 16.3 ppm;

IR: 3120, 2952, 2873, 1724, 1670, 1407, 1208, 1023, 894 cm^{-1};

HRMS (ESI): calculated for $[C_{15}H_{19}N_3O_5+H]^+$: 322.1397, found: 322.1388.

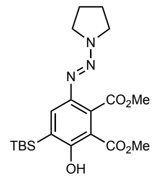

36, 70%

Prepared from **2-triazenyl-furan-08** according to the general procedure. by flash column chromatography (ratio of silica to crude mass= 100:1, eluting with 1:5 Et_2O:hexane).

Yield: 27.9 mg, 0.0662 mmol, 70%;

Appearance: Yellow solid;

R*f*: 0.25 (1:2 EtOAc:hexane);

^{1}H NMR (400 MHz, $CDCl_3$): δ 11.25 (s, 1H), 7.79 (s, 1H), 3.96 – 3.81 (m, 8H), 3.57 (s, 2H), 1.99 (s, 4H), 0.90 (s, 9H), 0.31 (s, 6H) ppm;

^{13}C NMR (100 MHz, $CDCl_3$): δ 170.2, 169.4, 164.5, 140.1, 131.7, 130.8, 128.8, 107.3, 52.9, 52.3, 30.5, 29.9, 27.3, 23.9, 23.8, 17.6, −4.6 ppm;

IR: 2952, 2856, 1740, 1396, 1209, 1035, 837 cm^{-1};

HRMS (ESI): calculated for $[C_{20}H_{31}N_3O_5Si+H]^+$: 422.2106, found: 422.2097.

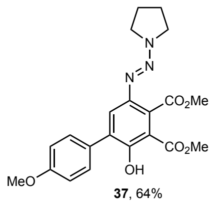

37, 64%

Prepared from **2-triazenyl-furan-09** according to the general procedure. Purified by flash column chromatography (ratio of silica to crude mass= 50:1, eluting with 1:1 Et_2O:hexane).

Yield: 24.0 mg, 0.0590 mmol, 64%;

Appearance: Yellow solid;

R*f*: 0.18 (2:5 EtOAc:hexane);

^{1}H NMR (400 MHz, $CDCl_3$): δ 10.78 (s, 1H), 7.35 (d, J = 7.2 Hz, 2H), 7.03 (s, 1H), 6.86 (d, J = 6.6 Hz, 2H), 3.93 (s, 3H), 3.82 (app d, J = 5.0 Hz, 6H), 3.72 – 3.37 (m, 4H), 1.92 (s, 4H) ppm;

^{13}C NMR (100 MHz, $CDCl_3$): δ 169.7, 169.0, 159.2, 158.9, 144.1, 139.5, 131.3, 131.3, 126.8, 120.5, 113.1, 108.6, 55.4, 53.0, 51.9, 50.6, 46.3, 23.9 (2 coincident peaks) ppm;

IR: 2954, 1734, 1435, 1247, 1034, 835 cm^{-1};

HRMS (ESI): calculated for $[C_{21}H_{23}N_3O_6+H]^+$: 414.1660, found: 414.1653.

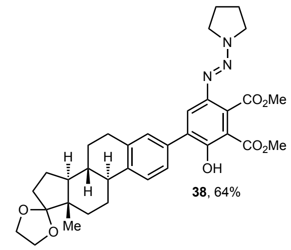

38, 64%

Prepared from **2-triazenyl-furan-10** according to the general procedure. Purified by flash column chromatography (ratio of silica to crude mass= 50:1, eluting with 1:9 EtOAc:hexane to 1:2 EtOAc:hexane).

Yield: 42.0 mg, 0.0696 mmol, 64%;

Appearance: Orange amorphous solid;

R*f*: 0.28 (2:5 EtOAc:hexane);

^{1}H NMR (900 MHz, $CDCl_3$): δ 10.75 (s, 1H), 7.25 (d, *J* = 7.5 Hz, 1H), 7.20 (dd, *J* = 8.2, 2.0 Hz, 1H), 7.17 (s, 1H), 7.07 (s, 1H), 3.98 – 3.88 (m, 7H), 3.81 (s, 3H), 3.66 (s, 2H), 3.43 (s, 2H), 2.88 – 2.81 (m, 2H), 2.38 – 2.35 (m, 1H), 2.30 (td, *J* = 11.0, 4.2 Hz, 1H), 2.03 (ddd, *J* = 14.3, 11.6, 3.1 Hz, 1H), 1.95 – 1.90 (m, 4H), 1.87 – 1.83 (m, 1H), 1.81 – 1.76 (m, 2H), 1.67 – 1.63 (m, 1H), 1.59 – 1.54 (m, 2H), 1.50 (qd, *J* = 13.3, 4.0 Hz, 1H), 1.44 (qd, *J* = 11.1, 2.6 Hz, 1H), 1.41 – 1.32 (m, 2H), 0.90 (s, 3H) ppm;

^{13}C NMR (225 MHz, $CDCl_3$): δ 169.7, 169.0, 158.9, 144.3, 139.9, 139.6, 136.0, 135.9, 130.7, 127.5, 126.8, 124.6, 120.6, 119.6, 108.7, 65.4, 64.7, 53.0, 51.7, 50.8, 49.6, 46.5, 46.3, 44.2, 39.0, 34.4, 30.9, 29.7, 27.2, 26.1, 24.1, 23.9, 22.5, 14.5 ppm;

IR: 3150, 2944, 2871, 1733, 1672, 1433, 1229, 1039, 910 cm^{-1};

HRMS (ESI): calculated for $[C_{34}H_{41}N_3O_7+H]^+$: 604.3017, found: 604.3009.

$[\alpha]D^{20}$: +19.8 (c = 0.40, $CHCl_3$).

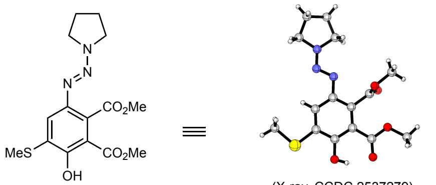

39, 84%

(X-ray, CCDC 2537279)

Prepared from **2-triazenyl-furan-21** according to the general procedure, but with five equivalents of dimethyl acetylenedicarboxylate and for 24 hours. Purified by flash column chromatography (ratio of silica to crude mass= 50:1, eluting with 1:2 EtOAc:hexane). A small quantity of pure **39** was crystallized via slow evaporation from Et_2O to yield yellow crystals suitable for X-ray analysis.

Yield: 42.0 mg, 0.119 mmol, 84%;

Appearance: Yellow solid;

R*f*: 0.24 (2:5 EtOAc:hexane);

M.p.: 119.7 – 123.9 °C;

^{1}H NMR (900 MHz, $CDCl_3$): δ 11.54 (s, 1H), 7.60 (s, 1H), 3.93 (s, 3H), 3.92 – 3.86 (m, 5H), 3.57 (br s, 2H), 2.47 (s, 3H), 2.00 (s, 4H) ppm;

^{13}C NMR (225 MHz, $CDCl_3$): δ 170.0, 169.0, 156.5, 140.9, 130.4, 126.4, 119.7, 107.8, 53.2, 52.4, 51.1, 46.5, 24.1, 23.6, 14.8 ppm;

IR: 3150, 2951, 2873, 1733, 1670, 1404, 1206, 1034, 917 cm^{-1};

HRMS (ESI): calculated for $[C_{15}H_{19}N_3O_5S+Na]^+$: 376.0938, found: 376.0927.

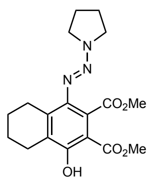

40, 83%

Prepared from **2-triazenyl-furan-17** according to the general procedure. Purified by flash column chromatography (ratio of silica to crude mass= 50:1, eluting with 1:9 EtOAc:hexane to 1:2 EtOAc:hexane).

Yield: 68.0 mg, 0.188 mmol, 83%;

Appearance: Yellow solid;

R*f*: 0.15 (1:2 EtOAc:hexane);

^{1}H NMR (400 MHz, $CDCl_3$): δ 11.15 (s, 1H), 3.88 (s, 3H), 3.78 (s, 3H), 3.76 – 3.55 (m, 4H), 2.73 (dt, *J* = 19.2, 6.3 Hz, 4H), 1.99 (t, *J* = 6.8 Hz, 4H), 1.74 (tt, *J* = 11.3, 5.2 Hz, 4H) ppm;

^{13}C NMR (225 MHz, $CDCl_3$): δ 170.4, 169.4, 157.5, 140.7, 139.1, 128.2, 122.6, 105.9, 52.8, 51.7, 50.8, 46.3, 26.7, 23.9, 23.9, 23.5, 22.4, 22.0 ppm;

IR: 3120, 2938, 2864, 1731, 1665, 1424, 1225, 1006, 980 cm^{-1};

HRMS (ESI): calculated for $[C_{18}H_{23}N_3O_5+H]^+$: 362.1710, found: 362.1700.

Allenes as Dienophiles

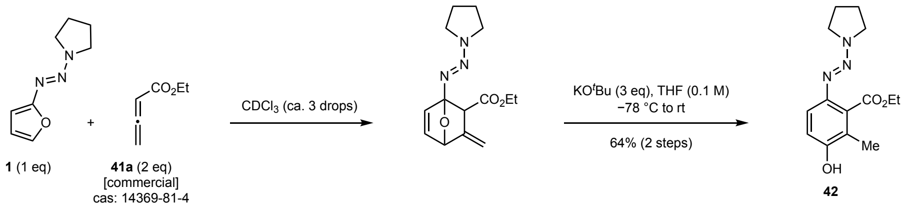

To a 1-dram vial open to air and equipped with a stir bar was added triazenyl furan **1** (70.0 mg, 0.424 mmol, 1.00 equiv) and ethyl 2,3-butadienoate (**41a**) (95.0 mg, 0.847 mmol, 2.00 equiv). A few drops of $CDCl_3$ were added to solubilize the furan, then the reaction was stirred at room temperature for 48 hours, by which time ^{1}H NMR analysis indicated the complete consumption of furan. The reaction mixture was transferred to a 1-neck 25 mL round-bottom flask with CH_2Cl_2 and concentrated under reduced pressure. A stir bar was added, and the flask was evacuated and backfilled with argon. Anhydrous THF (4.24 mL, 0.100 M) was added, the reaction flask was submerged in a dry ice/acetone bath, and a solution of KO^{t}Bu (143 mg, 1.27 mmol, 3.00 equiv) in anhydrous THF (4.24 mL, 0.300 M) was added dropwise. The reaction was allowed to stir for 30 minutes, then the dry ice/acetone bath was removed, and the reaction was allowed to stir for an additional 30 minutes. The reaction was quenched with a saturated aqueous solution of NH_4Cl and extracted with EtOAc (×3). The combined organic layers were washed with H_2O, brine, dried over $MgSO_4$, filtered, and concentrated under reduced pressure. The crude residue was purified by flash column chromatography (ratio of silica to crude mass = 30:1, eluting with 1:9 EtOAc:hexane).

Yield: 74.6 mg, 0.269 mmol, 64% (2 steps);

Appearance: Yellow solid;

R*f*: 0.17 (1:3 EtOAc:hexane);

^{1}H NMR (400 MHz, $CDCl_3$): δ 7.20 (d, *J* = 8.7 Hz, 1H), 6.72 (d, *J* = 8.7 Hz, 1H), 5.24 (br s, 1H), 4.37 (q, *J* = 7.1 Hz, 2H), 3.69 (br s, 4H), 2.18 (s, 3H), 1.97 (t, *J* = 6.6 Hz, 4H), 1.37 (t, *J* = 7.1 Hz, 3H) ppm;

^{13}C NMR (100 MHz, $CDCl_3$): δ 169.9, 151.5, 141.9, 130.9, 120.8, 116.5, 116.0, 61.1, 23.9 (2 coincident peaks), 14.5, 12.6 ppm (two of the pyrrolidinyl peaks are too broad to be visible within this spectra);

IR: 3190, 2977, 2873, 2114, 1714, 1573, 1289, 1158, 1046, 906 cm^{-1};

HRMS (ESI): calculated for $[C_{14}H_{19}N_3O_3+H]^+$: 278.1499, found: 278.1491.

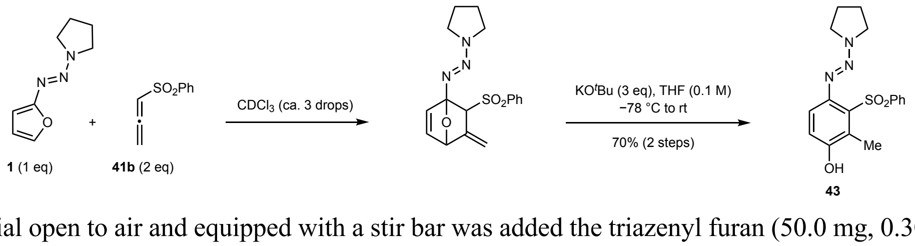

To a 1-dram vial open to air and equipped with a stir bar was added the triazenyl furan (50.0 mg, 0.303 mmol, 1.00 equiv) and (1,2-propadien-1-ylsulfonyl)benzene (**41b**)[60] (109 mg, 0.605 mmol, 2.00 equiv). A few drops of $CDCl_3$ were added to solubilize the furan, then the reaction was stirred at room temperature for 48 hours, by which time an

orange precipitate had formed and ^{1}H NMR analysis indicated the complete consumption of furan. The reaction was diluted with a small amount of cold Et_2O (ca. 0.500 mL), filtered, and the precipitate washed with a small amount of cold Et_2O (ca. 0.500 mL). The precipitate was transferred to a 10 mL round bottom flask with CH_2Cl_2 and concentrated under reduced pressure. A stir bar was added, and the flask was evacuated and backfilled with argon. Anhydrous THF (3.03 mL, 0.100 M) was added, the reaction flask was submerged in a dry ice/acetone bath, and a solution of KO^{t}Bu (102 mg, 0.908 mmol, 3.00 equiv) in anhydrous THF (3.03 mL, 0.300 M) was added dropwise. The reaction was allowed to stir for 30 minutes, then the dry ice/acetone bath was removed, and the reaction was allowed to stir for an additional 30 minutes. The reaction was quenched with a saturated aqueous solution of NH_4Cl and extracted with EtOAc (×3). The combined organic layers were washed with H_2O, brine, dried over $MgSO_4$, filtered, and concentrated under reduced pressure. The crude residue was purified by flash column chromatography (ratio of silica to crude mass = 30:1, eluting with 1:1 EtOAc:hexane).

Yield: 73.0 mg, 0.211 mmol, 70% (2 steps);

Appearance: Pale orange solid;

R*f*: 0.17 (2:5 EtOAc:hexane);

^{1}H NMR (400 MHz, $CDCl_3$): δ 7.84 (d, *J* = 0.9 Hz, 2H), 7.50 – 7.37 (m, 3H), 7.11 (d, *J* = 8.6 Hz, 1H), 6.96 (d, *J* = 8.5 Hz, 1H), 3.75 (br s, 2H), 3.28 (br s, 2H), 2.73 (s, 3H), 1.95 – 1.85 (m, 4H) ppm;

^{13}C NMR (100 MHz, $CDCl_3$): δ 152.0, 145.6, 144.6, 131.8, 131.8, 128.4, 126.3, 126.0, 121.0, 117.1, 50.8, 47.2, 23.8, 23.8, 12.9 ppm;

IR: 3388, 2975, 2874, 2359, 1404, 1267, 1150, 1023, 909 cm^{-1};

HRMS (ESI): calculated for $[C_{17}H_{19}N_3O_3S+H]^+$: 346.1220, found: 346.1211.

Tethered Dienophiles

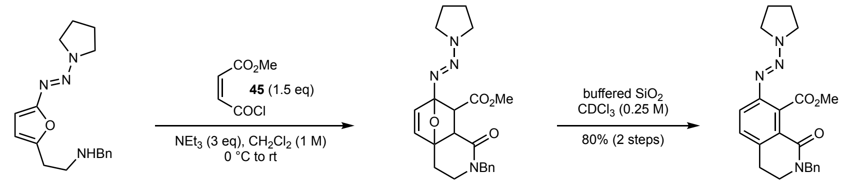

To a flame dried 10 mL round bottom flask under argon and equipped with a stir bar was added furan **44** (35.5 mg, 0.119 mmol, 1.00 equiv), anhydrous CH_2Cl_2 (0.595 mL, 0.200 M), and NEt_3 (0.497 mL, 0.357 mmol, 3.00 equiv). The reaction flask was submerged in an ice/water bath, and a solution of acid chloride **45**[61] (26.5 mg, 0.178 mmol, 1.50 equiv) in anhydrous CH_2Cl_2 (0.178 mL, 1.00 M) was added dropwise. The reaction flask was removed from the ice/water bath and stirred at ambient temperature for one hour, by which time TLC analysis indicated the complete consumption of **44**. The reaction was diluted with Et_2O (×3 reaction volume) and filtered to remove solids. The filtrate was concentrated under reduced pressure, the crude residue transferred to a 2-dram vial, and a stir bar, CH_2Cl_2, and phosphate buffered silica (pH= 7, ×10 theoretical mass of **46**) were added. The reaction was sealed and stirred at ambient temperature for 12 hours, by which time TLC analysis indicated the complete consumption of **46**. The reaction was filtered (washing silica with acetone) and then concentrated under reduced pressure. The crude residue was purified by flash column chromatography (ratio of silica to crude mass = 40:1, eluting with 1:1 EtOAc:hexane). **Note**: The Diels–Alder reaction did not always go to completion during the first step. However, we found this did not impact the overall two-step yield (i.e., the second step could be conducted with a mixture of the cycloaddition precursor and cycloadduct **46**).

Yield: 37.4 mg, 0.0953 mmol, 80% (2 steps);

Appearance: Yellow solid;

R*f*: 0.15 (1:1 EtOAc:hexane);

^{1}H NMR (400 MHz, $CDCl_3$): δ 7.59 (d, *J* = 8.2 Hz, 1H), 7.35 – 7.27 (m, 5H), 7.11 (d, *J* = 8.2 Hz, 1H), 4.86 (br s, 1H), 4.6 (br s, 1H), 3.99 (s, 3H), 3.95 – 3.85 (m, 2H), 3.69 – 3.55 (m, 2H), 3.48 – 3.37 (m, 2H), 2.94 – 2.81 (m, 2H), 2.05 – 1.97 (m, 4H) ppm;

^{13}C NMR (100 MHz, $CDCl_3$): δ 170.4, 163.5, 147.8, 137.5, 135.1, 130.3, 128.7, 128.4, 128.2, 127.6, 126.8, 119.9, 52.4, 51.1, 50.2, 46.6, 44.9, 28.3, 24.1, 23.6 ppm;

IR: 2946, 2873, 1732, 1650, 1403, 1252, 1124, 1018, 912 cm^{-1};

HRMS (ESI): calculated for $[C_{22}H_{24}N_4O_3+Na]^+$: 415,1741, found: 415.1729.

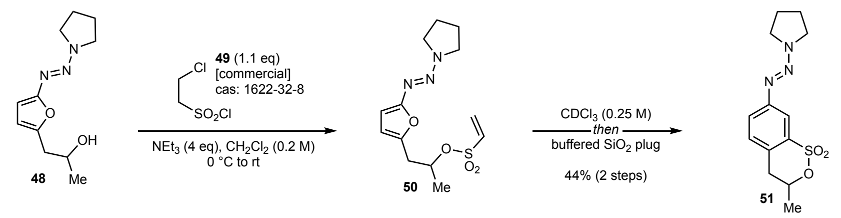

To a flame dried 10 mL round bottom flask under argon and equipped with a stir bar was added furan **48** (200 mg, 0.896 mmol, 1.00 equiv), anhydrous CH_2Cl_2 (4.48 mL, 0.200 M), and NEt_3 (0.499 mL, 3.58 mmol, 4.00 equiv). The reaction flask was submerged in an ice/water bath, 2-chloroethanesulfonyl chloride (**49**) (0.103 mL, 0.986 mmol, 1.10 equiv) was added dropwise, and the reaction was stirred for one hour, by which time TLC analysis indicated the complete consumption of **48**. The reaction was diluted with Et_2O and passed through a silica plug (×10 mass theoretical yield of **50**), eluting with Et_2O, to remove solids. The filtrate was concentrated under reduced pressure, the crude residue transferred to a 2-dram vial, and a stir bar and $CDCl_3$ (3.58 mL, 0.25 M) were added. The reaction was sealed and stirred at ambient temperature for 48 hours, by which time ^{1}H NMR analysis indicated the complete consumption of **50**. The reaction mixture was passed through a phosphate buffered silica plug (pH= 7, ×30 mass theoretical yield of **51**), eluting with EtOAc, and the filtrate was concentrated under reduced pressure. The crude residue was purified by flash column chromatography (ratio of silica to crude mass = 30:1, eluting with 2:5 EtOAc:hexane).

Yield: 117 mg, 0.396 mmol, 44% (2 steps);

Appearance: Yellow solid;

R*f*: 0.29 (1:1 EtOAc:hexane);

^{1}H NMR (600 MHz, $CDCl_3$): δ 7.90 (d, *J* = 2.2 Hz, 1H), 7.51 (dd, *J* = 8.3, 2.1 Hz, 1H), 7.17 (d, *J* = 8.4 Hz, 1H), 5.26 – 5.20 (m, 1H), 3.93 (t, *J* = 7.0 Hz, 2H), 3.64 (t, *J* = 7.3 Hz, 2H), 3.14 (dd, *J* = 17.0, 11.6 Hz, 1H), 2.95 (dd, *J* = 17.1, 3.0 Hz, 1H), 2.07 – 2.00 (m, 4H), 1.59 (d, *J* = 6.4 Hz, 3H) ppm;

^{13}C NMR (100 MHz, $CDCl_3$): δ 151.4, 135.2, 129.6, 129.5, 125.4, 115.8, 79.6, 51.4, 46.6, 35.5, 23.9, 23.8, 21.3 ppm;

IR: 2977, 2874, 1485, 1396, 1308, 1189, 1029, 882 cm^{-1};

HRMS (ESI): calculated for $[C_{13}H_{17}N_3O_3S+H]^+$: 296.1063, found: 296.1061.

Benzyne as Dienophile

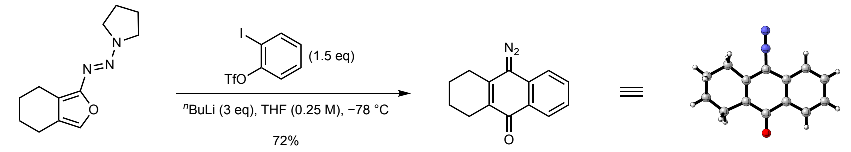

2-triazenyl-furan-17 (or **52**) **53**

To a flame-dried round-bottom flask under argon and equipped with a stir bar was added 2**-triazenyl-furan-17** (or **52**) (250 mg, 1.14 mmol, 1.00 equiv), 2-iodophenyl trifluoromethanesulfonate[6] (602 mg,1.71 mmol, 1.50 equiv), and anhydrous THF (4.56 ml, 0.25 M). The reaction flask was submerged in a dry ice/acetone bath, and freshly titrated[16] *n*-butyllithium (1.52 ml, 2.25 M in hexane, 3.00 equiv) was added dropwise over one hour via syringe pump. The reaction was stirred for an additional 1 hour, then quenched with a saturated aqueous solution of NH_4Cl. The reaction was warmed to ambient temperature and extracted with EtOAc (×3). The combined organic layers were washed with H_2O, brine, dried over $MgSO_4$, filtered, and concentrated under reduced pressure. The crude residue was purified by flash column chromatography using phosphate buffered silica (pH = 7, ratio of silica to crude mass = 40:1, eluting with 5:95 EtOAc:hexane to 2:8 EtOAc:hexane). A small quantity of pure **53** was crystallized via slow evaporation from $CDCl_3$ to yield red crystals suitable for X-ray analysis.

Yield: 184 mg, 1.14 mmol, 72%;

Appearance: Red solid;

R*f*: 0.22 (1:5 EtOAc:hexane);

M.p.: 93.2 – 99.1 °C;

^{1}H NMR (600 MHz, $CDCl_3$): δ 8.40 (d, *J* = 8.0 Hz, 1H), 7.57 (td, *J* = 7.7, 1.4 Hz, 1H), 7.37 (td, *J* = 7.6, 1.1 Hz, 1H), 7.18 (d, *J* = 8.6 Hz, 1H), 2.62 (t, *J* = 6.3 Hz, 2H), 2.55 (t, *J* = 6.2 Hz, 2H), 1.88 – 1.83 (m, 2H), 1.81 – 1.76 (m, 2H) ppm;

^{13}C NMR (150 MHz, $CDCl_3$) δ 179.9, 137.0, 131.2, 130.6, 129.4, 129.4, 128.1, 125.7, 120.0, 70.0, 26.3, 23.8, 22.1, 21.8 ppm;

IR: 2937, 2862, 2060, 1583, 1482, 1288, 1173, 756 cm^{-1};

HRMS (ESI): calculated for $[C_{14}H_{12}N_2O+Na]^+$: 247.0842, found: 247.0836.

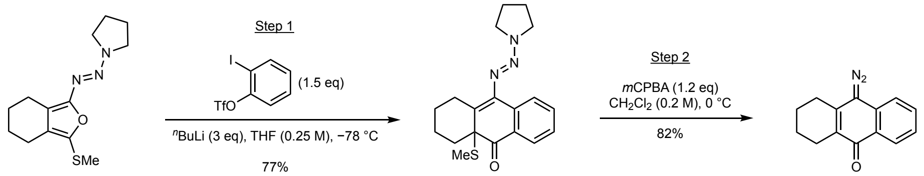

2-triazenyl-furan-20 (or **54**) **55** **53**

Step 1

To a flame-dried 1-neck 10 mL round-bottom flask under argon and equipped with a stir bar was added **2-triazenyl-furan-20** (or **54**) (71.0 mg, 0.269 mmol, 1.00 equiv), 2-iodophenyl trifluoromethanesulfonate[6] (142 mg, 0.403 mmol, 1.50 equiv), and anhydrous THF (1.07 mL, 0.25 M). The reaction flask was submerged in a dry ice/acetone

bath and freshly titrated[16] *n*-butyllithium (0.344 mL, 2.34 M in hexane, 3.00 equiv) was added dropwise over one hour via syringe pump. The reaction was stirred for an additional 1 hour and then quenched with a saturated aqueous solution of NH_4Cl. The reaction was warmed to ambient temperature and extracted with EtOAc (×2). The combined organic layers were washed with H_2O, brine, dried over $MgSO_4$, filtered, and concentrated under reduced pressure. The crude residue was purified by flash column chromatography using phosphate buffered silica (pH = 7, ratio of silica to crude mass = 40:1, eluting with 3:97 Et_2O:hexane to 1:9 Et_2O:hexane).

Yield: 70.3 mg, 0.206 mmol, 77%;

Appearance: Orange viscous oil;

R_f: 0.20 (1:5 EtOAc:hexane);

^{1}H NMR (400 MHz, $CDCl_3$): δ 8.09 (d, *J* = 7.7 Hz, 1H), 7.50 (t, *J* = 7.0 Hz, 1H), 7.38 – 7.31 (m, 2H), 3.80 (br s, 4H), 2.76 (d, *J* = 17.9 Hz, 1H), 2.41 (d, *J* = 14.8 Hz, 1H), 2.28 (td, *J* = 14.2, 4.5 Hz, 1H), 2.07 (br s, 4H), 1.93 (dt, *J* = 12.8, 3.5 Hz, 1H), 1.87 (s, 3H), 1.82 (br s, 1H), 1.75 (br d, *J* = 16.0 Hz, 1H), 1.60 (td, *J* = 14.1, 3.8 Hz, 1H), 1.37 – 1.25 (m, 1H) ppm;

^{13}C NMR (100 MHz, $CDCl_3$): δ 193.8, 140.6, 136.5, 133.6, 129.4, 127.6, 127.5, 127.4, 124.4, 54.3, 29.4, 26.0, 24.9, 24.0 (2 coincident peaks), 21.2, 11.3 ppm (two of the pyrrolidinyl peaks are too broad to be visible within this spectra);

IR: 2928, 2857, 2057, 1662, 1418, 1287, 1145, 899 cm^{-1};

HRMS (ESI): calculated for $[C_{19}H_{23}N_3OS+H]^+$: 342.1635, found: 342.1626.

<u>Step 2</u>

Note: Dry *m*-CPBA is necessary for high yields. This was obtained by evaporation of *m*-CPBA with anhydrous PhMe (in triplicate) and then placing under high vacuum for several hours. Caution should be taken when handling dry *m*-CPBA (e.g., blast shield).

A 2-dram vial containing **55** (70.0 mg, 0.205 mmol, 1.00 equiv) was equipped with a stir bar and evacuated/backfilled with argon in triplicate. Anhydrous CH_2Cl_2 (1.02 mL, 0.200 M) was added, and the vial was submerged in an ice/water bath. A solution of dry *m*-CPBA (42.4 mg, 0.246 mmol, 1.20 equiv) in anhydrous CH_2Cl_2 (0.615 mL, 0.400 M) was added dropwise, and the reaction was stirred for 30 minutes. The reaction was quenched with saturated aqueous $NaHCO_3$ solution and warmed to ambient temperature. The aqueous layer was extracted with CH_2Cl_2, and the combined organic layers were washed with H_2O, brine, dried over $MgSO_4$, filtered, and concentrated under reduced pressure. The crude residue was purified by flash column chromatography using phosphate buffered silica (pH = 7, ratio of silica to crude mass = 40:1, eluting with 1:5 Et_2O:hexane).

Yield: 37.7 mg, 0.168 mmol, 82%;

Characterization data matched those reported earlier within this document.

Derivatization Experiments

From Cycloadduct 29

summary

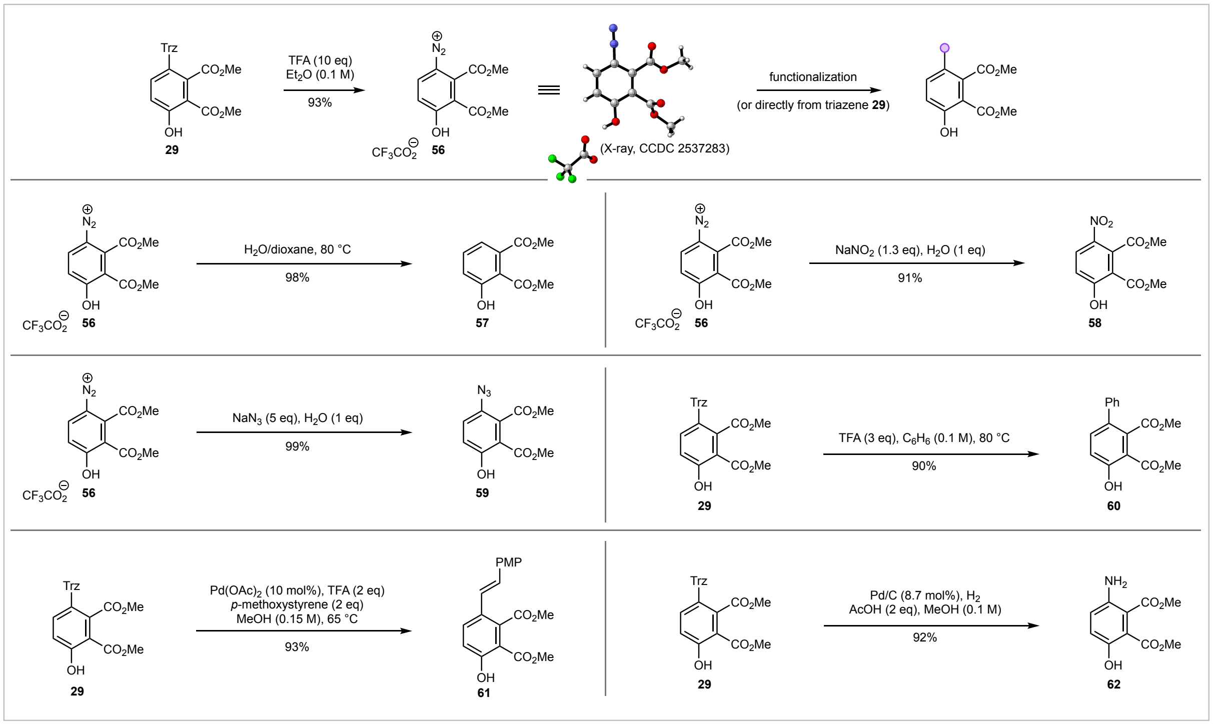

Caution Diazonium salts are potentially explosive. Appropriate precautions should be taken during handling and storage (e.g., blast shield, minimize exposure to heat, light, and shock).

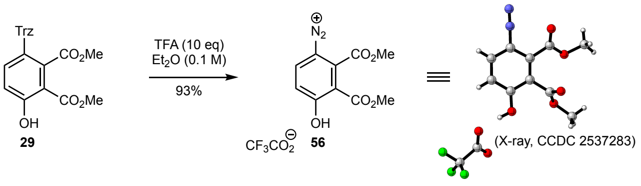

To a 50 mL 1-neck round-bottom flask open to air and equipped with a stir bar was added triazene **29** (200 mg, 0.651 mmol, 1.00 equiv) and Et_2O (6.51 mL, 0.100 M). The flask was submerged in an ice/water bath and trifluoroacetic acid (742 mg, 0.501 mL, 6.51 mmol, 10.0 equiv) was added slowly (approximately 30 seconds). The ice/water bath was removed, and the reaction was stirred for 3 hours (precipitate forms). The precipitate was collected by vacuum filtration (washing with a minimal amount of Et_2O). A small quantity of pure **56** was crystallized via slow evaporation from $CDCl_3$ to yield orange crystals suitable for X-ray analysis.

Yield: 211 mg, 0.651 mmol, 93%;

Appearance: White to orange-brown solid;

R*f*: 0.45 (1:9 MeOH:CH_2Cl_2);

M.p.: 114.3 – 121.0 °C;

^{1}H NMR (400 MHz, $(CD_3)_2SO$): δ 8.00 (d, *J* = 9.8 Hz, 1H), 6.49 (d, *J* = 9.8 Hz, 1H), 5.32 (br s, 5H), 3.84 (s, 3H), 3.74 (s, 3H) ppm;

^{13}C NMR (100 MHz, $(CD_3)_2SO$): δ 176.3, 166.2, 162.6, 158.8 (q, *J* = 37.2 Hz), 135.0, 132.2, 130.0, 126.0, 115.8 (q, *J* = 288.3 Hz), 78.06, 54.05, 52.77 ppm;

^{19}F NMR (377 MHz, $CDCl_3$): δ −76.5 ppm;

IR: 3750, 2960, 2154, 1754, 1587, 1307 cm^{-1};

HRMS (ESI): calculated for $[C_{12}H_9F_3N_2O_5{-}C_2F_3O_2]^+$: 237.0506, found: 237.0497.

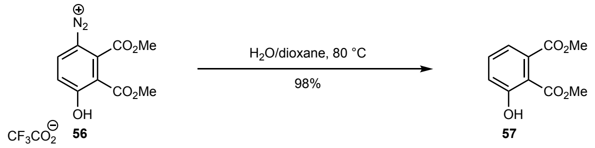

To a 50 mL 1-neck round-bottom flask open to air and equipped with a stir bar was added diazonium **56** (50.4 mg, 0.144 mmol, 1.00 equiv), H_2O (0.720 mL, 0.200 M), and 1,4-dioxane (0.720 mL, 0.200 M). The vial was sealed with a screw cap, wrapped with electrical tape, and warmed on a heating block at 80 °C for 16 hours. The heating mantle was removed, and the reaction was allowed to cool to ambient temperature. The reaction was extracted with EtOAc (×2), and the combined organic layers were washed with H_2O, brine, dried over $MgSO_4$, filtered, and concentrated under reduced pressure.

Characterization data matched those reported by Bahlis and Derksen.[62]

Yield: 29.0 mg, 0.144 mmol, 98%;

Appearance: Yellow oil;

R*f*: 0.44 (3:7 EtOAc:hexane);

^{1}H NMR (400 MHz, $CDCl_3$): δ 10.56 (br s, 1H), 7.44 (t, *J* = 7.8 Hz, 1H), 7.07 (d, *J* = 8.6 Hz, 1H), 6.95 (d, *J* = 7.5 Hz, 1H), 3.91 (s, 3H), 3.88 (s, 3H) ppm.

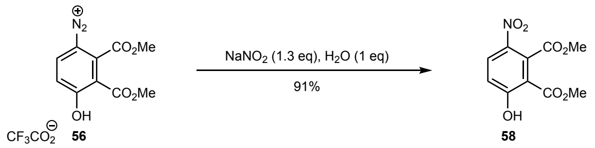

Conducted according to a modification of a related procedure reported by Tour.[63] To a 2-dram vial open to air and equipped with a stir bar was added diazonium **56** (50.0 mg, 0.143 mmol, 1.00 equiv) and H_2O (2.86 mL, 0.0500 M). The salt was allowed to dissolve (approximately 5 min), a solution of $NaNO_2$ (98.5 mg, 60.7 mmol, 1.30 equiv) in H_2O (1.43 mL, 1.00 M) was added dropwise, and the reaction was stirred for 16 hours. The reaction was extracted with CH_2Cl_2 (×5), and the combined organic layers were washed with H_2O, brine, dried over $MgSO_4$, filtered, and concentrated under reduced pressure. **Note**: The product appears to be slightly sensitive and decomposes upon standing over the course of several days at ambient temperature.

Yield: 33.0 mg, 0.143 mmol, 91%;

Appearance: Orange solid;

R*f*: 0.46 (1:10 MeOH:CH_2Cl_2);

^{1}H NMR (400 MHz, $CDCl_3$): δ 7.49 (d, *J* = 9.7 Hz, 1H), 6.66 (d, *J* = 9.7 Hz, 1H), 3.92 (s, 3H), 3.90 (s, 3H), 2.51 (br s, 1H) ppm;

^{13}C NMR (100 MHz, $CDCl_3$): δ 168.9, 167.0, 159.0, 128.9, 126.7, 125.6, 120.7, 110.1, 53.4, 53.0 ppm;

IR: 2140, 1754, 1738, 1591, 1440, 1307, 1210, 726 cm^{-1};

HRMS (ESI): calculated for $[C_{10}H_9NO_7{-}H]^-$: 254.0306, found: 254.0308.

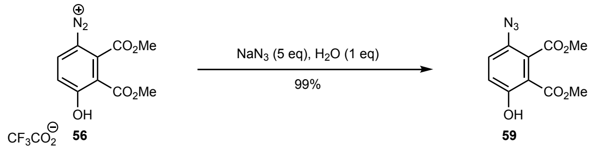

56 59

To a 2-dram vial open to air and equipped with a stir bar was added diazonium **56** (30.0 mg, 0.0857 mmol, 1.00 equiv) and H_2O (0.857 mL, 0.100 M). The salt was allowed to dissolve (approximately 5 min), and NaN_3 (27.8 mg, 0.428 mmol, 5.00 equiv) was added in one portion (white foam forms). The reaction was stirred for 2 hours at room temperature, by which time the foam has dissipated to leave a clear solution and a white precipitate. The reaction was extracted with Et_2O (×3), and the combined organic layers were washed with H_2O, brine, dried over $MgSO_4$, filtered, and concentrated under reduced pressure.

Yield: 21.4 mg, 0.0854 mmol, 99%;

Appearance: Off-white solid;

R*f*: 0.34 (1:4 EtOAc:hexane);

^{1}H NMR (400 MHz, $CDCl_3$): δ 10.85 (s, 1H), 7.32 (d, *J* = 8.9 Hz, 1H), 7.12 (d, *J* = 9.0 Hz, 1H), 3.92 (s, 3H), 3.92 (s, 3H) ppm;

^{13}C NMR (100 MHz, $CDCl_3$): δ 165.9, 167.0, 159.1, 128.9, 126.6, 120.7, 125.6, 120.7, 53.4, 53.0 ppm;

IR: 2355, 2126, 1743, 1688, 1679, 1596, 1462, 1451, 1307, 1251, 1017 cm^{-1};

HRMS (ESI): calculated for $[C_{10}H_9N_3O_5{+}Na]^+$: 274.0434, found: 274.0430.

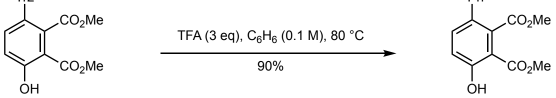

29 60

To a flame-dried 2-dram vial under argon and equipped with a stir bar was added triazene **29** (50.0 mg, 0.163 mmol, 1.00 equiv), anhydrous benzene (1.63 mL, 0.100 M), followed by the dropwise addition of trifluoroacetic acid (0.038 mL, 0.488 mmol, 3.00 equiv). The vial was sealed with a screw cap, wrapped with electrical tape, and warmed on a heating block at 80 °C for 18 hours. The reaction was cooled to ambient temperature, diluted with

H_2O and extracted with EtOAc (×2). The combined organic layers were washed with H_2O, brine, dried over $MgSO_4$, filtered, and concentrated under reduced pressure. The crude residue was purified by flash column chromatography (ratio of silica to crude mass = 30:1, eluting with 1:9 EtOAc:hexane).

Characterization data matched those reported by Sonoda.[64]

Yield: 42.0 mg, 0.147 mmol, 90%;

Appearance: Yellow solid;

R*f*: 0.35 (1:4 EtOAc:hexane);

^{1}H NMR (400 MHz, $CDCl_3$): δ 10.96 (s, 1H), 7.44 (d, *J* = 8.6 Hz, 1H), 7.40 – 7.29 (m, 5H), 7.11 (d, *J* = 8.7 Hz, 1H), 3.92 (s, 3H), 3.61 (s, 3H) ppm.

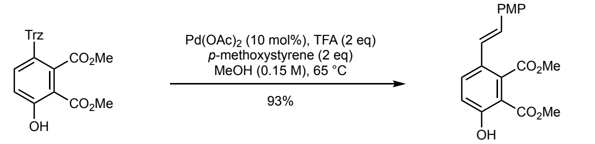

29 **61**

Conducted according to a modification of a related procedure reported by Mao, Zhang, and Li.[65] To a 2-dram vial under argon and equipped with a stir bar was added triazene **29** (100 mg, 0.324 mmol, 1.00 equiv), 4-methoxystyrene (87.3 mg, 0.651 mmol, 2.00 equiv.), TFA (0.0501 mL, 0.651 mmol, 2.00 equiv), $Pd(OAc)_2$ (7.31 mg, 0.0325 mmol, 0.100 equiv.), and MeOH (2.17 mL, 0.150 M). The vial was sealed with a screw cap, wrapped with electrical tape, and warmed on a heating block at 65 °C for 40 minutes. The crude residue was concentrated under reduced pressure and purified by flash column chromatography (ratio of silica to crude mass = 30:1, eluting with 1:10 EtOAc: hexane).

Yield: 33.0 mg, 0.143 mmol, 93%;

Appearance: Off-white solid;

R*f*: 0.29 (1:4 EtOAc:hexane);

^{1}H NMR (400 MHz, $CDCl_3$): δ 10.99 (s, 1H), 7.80 (d, *J* = 8.9 Hz, 1H), 7.38 (d, *J* = 7.6 Hz, 2H), 7.07 (d, *J* = 9.6 Hz, 1H), 6.97 – 6.77 (m, 5H), 3.94 (s, 6H), 3.82 (s, 3H) ppm;

^{13}C NMR (100 MHz, $CDCl_3$): δ 169.5, 169.3, 161.1, 159.7, 133.6, 132.4, 130.3, 129.8, 128.0, 127.1, 121.6, 119.7, 114.3, 109.2, 55.4, 53.2, 52.6 ppm;

IR: 2927, 2656, 1763, 1697, 1608, 1519, 1301, 1260, 1135, 1091, 1028, 965, 807, 773, 754, 683cm^{-1};

HRMS (ESI): calculated for $[C_{19}H_{18}O_6+Na]^+$: 365.0996, found: 365.0995.

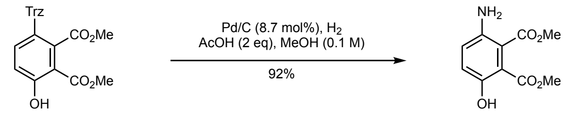

29 → **62**

Conducted according to a modification of a related procedure reported by Huang.[66] To a 2-dram vial open to air and equipped with a stir bar was added triazene **29** (100 mg, 0.326 mmol, 1.00 equiv), MeOH (3.26 mL, 0.100 M), AcOH (39.0 mg, 0.652 mmol, 2.00 equiv), and Pd/C (30.1 mg, 8.7 mol%, 0.0283 mmol). The vial was fit with a septum and H_2 (balloon) was bubbled through the solution for 5 minutes. The reaction was then vigorously stirred at ambient temperature for 24 h (H_2 balloon still attached), by which time TLC indicated the complete consumption of starting material. The reaction was filtered through a pad of celite (eluting with MeOH), and the filtrate was concentrated under reduced pressure.

Yield: 67.0 mg, 326 mmol, 92%;

Appearance: Orange solid;

R*f*: 0.30 (3:7 EtOAc:hexane);

^{1}H NMR (400 MHz, $CDCl_3$): δ 9.52 (br s, 1H), 6.92 (d, *J* = 8.9 Hz, 1H), 6.85 (d, *J* = 9.0 Hz, 1H), 4.23 (br s, 2H), 3.88 (s, 3H), 3.84 (s, 3H) ppm;

^{13}C NMR (100 MHz, $CDCl_3$): δ 169.8, 168.9, 153.1, 139.1, 124.7, 121.7, 115.2, 111.3, 52.8, 52.4 ppm;

IR: 3375, 2955, 2351, 1737, 1726, 1710, 1692, 1679, 1598, 1479, 1468, 1442, 1433, 1304 cm^{-1};

HRMS (ESI): calculated for $[C_{10}H_{11}NO_5+Na]^+$: 248.0529, found: 248.0527.

From Phthalic Anhydride 64

summary

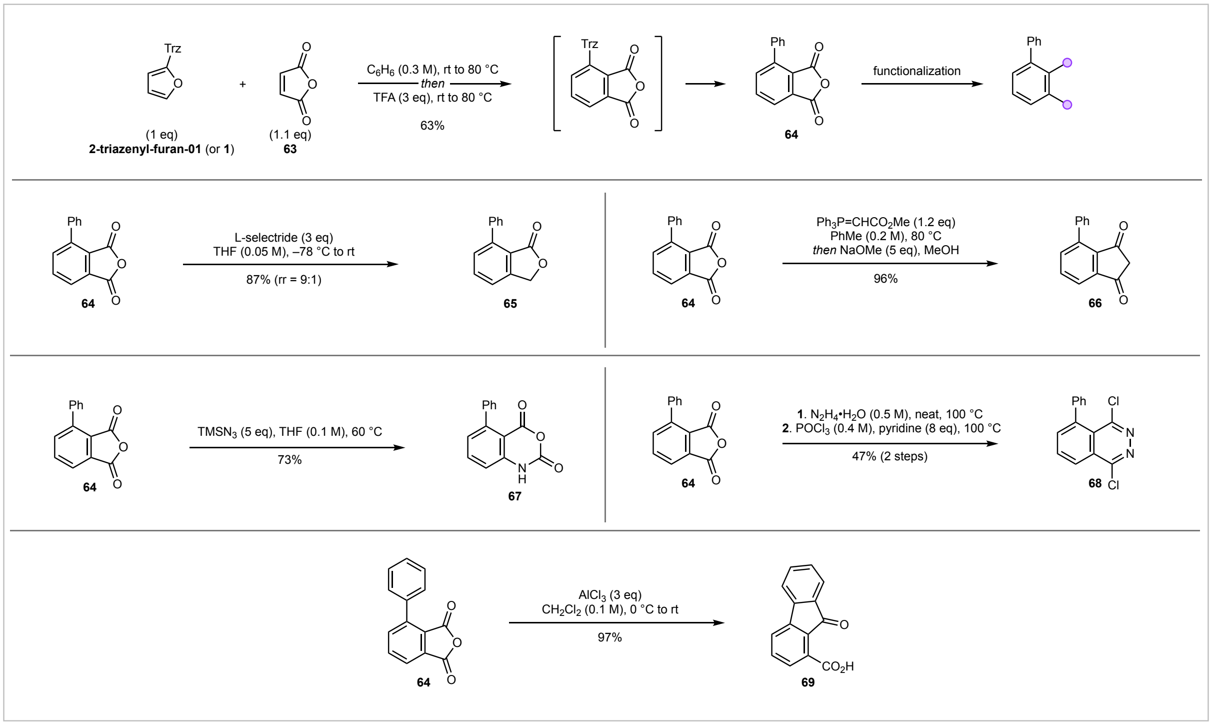

Caution Diazonium salts are potentially explosive. Appropriate precautions should be taken during handling and storage (e.g., blast shield, minimize exposure to heat, light, and shock).

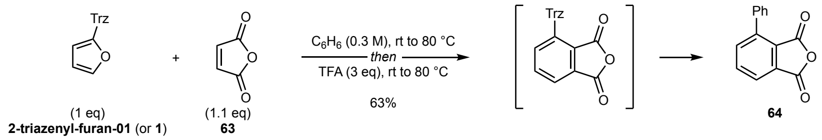

To a flame-dried 1-neck 25 mL round-bottom flask under argon and equipped with a stir bar was **2-triazenyl-furan-01** (or **1**) (300 mg, 1.82 mmol, 1.00 equiv) and anhydrous PhH (6.05 mL, 0.300 M). The furan was allowed to fully dissolve, then maleic anhydride (**63**) (196 mg, 2.00 mmol, 1.10 equiv) was added in one portion. The reaction was stirred at ambient temperature for 20 minutes, during which a yellow precipitate forms. The reaction flask was warmed to 80 °C on a heating mantle for 2 hours, then cooled to ambient temperature. Trifluoroacetic acid (0.420 mL, 5.45 mmol, 3.00 equiv) was slowly added, then the reaction was warmed to 80 °C on a heating mantle during which gas evolution was observed. The reaction was cooled to ambient temperature and concentrated under reduced pressure. The crude residue was purified by flash column chromatography (ratio of silica to crude mass = 30:1, eluting with 5:95 EtOAc:hexane to 1:9 EtOAc:hexane).

Yield: 255 mg, 1.14 mmol, 63%;

Appearance: White to pale yellow solid;

R*f*: 0.42 (1:4 EtOAc:hexane);

^{1}H NMR (400 MHz, $CDCl_3$): δ 8.01 (d, *J* = 7.1 Hz, 1H), 7.93 (t, *J* = 7.7 Hz, 1H), 7.85 (d, *J* = 7.5 Hz, 1H), 7.63 – 7.45 (m, 5H) ppm;

^{13}C NMR (100 MHz, $CDCl_3$): δ 162.9, 162.1, 143.5, 137.7, 136.0, 135.1, 132.6, 129.6, 129.4, 128.7, 127.1, 124.5 ppm;

IR: 3063, 1848, 1778, 1472, 1262, 1212, 907 cm^{-1};

HRMS (ESI): calculated for $[C_{14}H_8O_3+H]^+$: 225.0546, found: 225.0542.

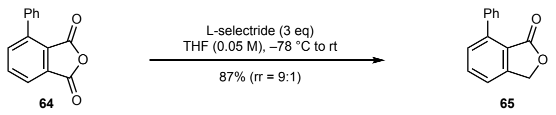

Conducted according to a modification of a related procedure reported by Kayser.[67] To a flame-dried 1-neck 10 mL round-bottom flask under argon and equipped with a stir bar was added 4-phenylisobenzofuran-1,3-dione (**64**) (50.0 mg, 0.223 mmol, 1.00 equiv) and anhydrous THF (4.46 mL, 0.0500 M). The solution was submerged in a dry ice/acetone bath, and L-Selectride (0.669 mL, 1.00 M in THF, 0.669 mmol, 3.00 equiv) was added dropwise over approximately 10 minutes. The reaction was stirred for two hours, warmed to ambient temperature, and quenched with a 2 M aqueous HCl solution. The reaction was extracted with EtOAc (×2), and the combined organic layers were washed with H_2O, brine, dried over $MgSO_4$, filtered, and concentrated under reduced pressure. The crude residue was purified by flash column chromatography (ratio of silica to crude mass = 30:1, eluting with 1:5 EtOAc:hexane).

Minor regioisomer has been fully characterized by Padwa.[68]

Yield: 41.0 mg, 0.194 mmol, 87%;

Appearance: Pale yellow solid;

R*f*: 0.30 (major) and 0.43 (minor) (1:5 EtOAc:hexane);

^{1}H NMR (400 MHz, $CDCl_3$, only major isomer indicated): δ 7.71 (t, *J* = 6.5 Hz, 1H), 7.54 (app d, *J* = 7.3 Hz, 2H), 7.50 – 7.40 (m, 5H), 5.32 (s, 2H) ppm;

^{13}C NMR (100 MHz, $CDCl_3$, only major isomer indicated): δ 169.9, 148.0, 143.0, 136.5, 13.40, 131.0, 129.7, 128.5, 128.1, 121.9, 120.9, 68.6 ppm;

IR: 2927, 1754, 1599, 1472, 1358, 1202, 1039, 799 cm^{-1};

HRMS (ESI): calculated for $[C_{14}H_{10}O_2+H]^+$: 211.0754, found: 211.0747.

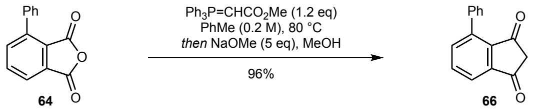

Conducted according to a modification of a related procedure reported by Tang.[69] To a flame-dried 1-neck 10 mL round-bottom flask under argon and equipped with a stir bar was added 4-phenylisobenzofuran-1,3-dione (**64**) (50.2 mg, 0.224 mmol, 1.00 equiv), methyl 2-(triphenyl-l5-phosphaneylidene)acetate (82.3 mg, 0.246 mmol, 1.20 equiv),

and anhydrous PhMe (1.12 mL, 0.200 M). The reaction was warmed to 80 °C on a heating mantle for 1 hour, then cooled to ambient temperature. NaOMe (60.5 mg, 1.12 mmol, 5.00 equiv) and MeOH (1.12 mL, 0.200 M) were added, then the reaction was warmed to 80 °C on a heating mantle for 1 hour, during which time a yellow precipitate formed. The reaction was cooled to ambient temperature and an aqueous 2 M HCl solution (ca. ×1 reaction volume) was added and the reaction was warmed to 80 °C on a heating mantle for 1 hour. The reaction was cooled to ambient temperature and extracted with EtOAc (×2). The combined organic layers were washed with H_2O, brine, dried over $MgSO_4$, filtered, and concentrated under reduced pressure. The crude residue was purified by flash column chromatography (ratio of silica to crude mass = 50:1, eluting with 1:9 EtOAc:hexane).

Yield: 48.0 mg, 0.216 mmol, 96%;

Appearance: Green solid;

R*f*: 0.42 (1:5 EtOAc:hexane);

^{1}H NMR (400 MHz, $CDCl_3$): δ 7.98 (d, *J* = 7.5 Hz, 1H), 7.84 (t, *J* = 7.5 Hz, 1H), 7.73 (d, *J* = 7.5 Hz, 1H), 7.51 – 7.43 (m, 5H), 3.25 (s, 2H) ppm;

^{13}C NMR (100 MHz, $CDCl_3$): δ 197.6, 196.5, 144.6, 141.6, 139.2, 137.8, 136.9, 135.2, 129.4, 128.7, 128.2, 122.2, 45.7 ppm;

IR: 3061, 2922, 2853, 1707, 1469, 1330, 1212, 909 cm^{-1};

HRMS (ESI): calculated for $[C_{15}H_{10}O_2+Na]^+$: 245.0573, found: 245.0569.

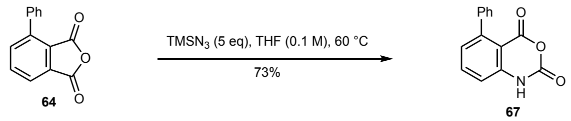

Conducted according to a modification of a related procedure reported by Sarmiento.[70] To a flame-dried 2-dram vial under argon and equipped with a stir bar was added 4-phenylisobenzofuran-1,3-dione (**64**) (50.0 mg, 0.223 mmol, 1.00 equiv), $TMSN_3$ (0.147 mL, 1.12 mmol, 5.00 equiv), and anhydrous THF (2.23 mL, 0.100 M). The vial was sealed with a screw cap, wrapped with electrical tape, and warmed on a heating block at 60 °C for 24 hours. The reaction was cooled to ambient temperature and concentrated under reduced pressure. The crude residue was purified by flash column chromatography (ratio of silica to crude mass = 50:1, eluting with 1:9 EtOAc:hexane to 1:5 EtOAc:hexane).

Yield: 39.0 mg, 0.163 mmol, 73%;

Appearance: White solid;

R*f*: 0.29 (1:4 EtOAc:hexane);

^{1}H NMR (400 MHz, $CDCl_3$): δ 8.13 (d, *J* = 7.9 Hz, 1H), 7.71 (br s, 1H), 7.63 (d, *J* = 7.5 Hz, 1H), 7.60 – 7.49 (m, 3H), 7.40 – 7.31 (m, 3H) ppm;

^{13}C NMR (150 MHz, $CDCl_3$): δ 158.9, 146.6, 137.8, 137.2, 134.4, 130.1, 129.9, 129.6, 129.0, 129.0, 124.5, 110.7 ppm;

IR: 3274, 1783, 1602, 1506, 1354, 1020, 753 cm^{-1};

HRMS (ESI): calculated for $[C_{14}H_9NO_3+H]^+$: 240.0655, found: 240.0648.

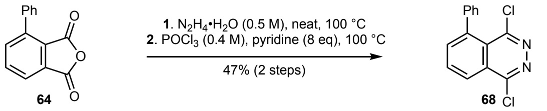

Step 1

To a 2-dram vial open to air and equipped with a stir bar was added 4-phenylisobenzofuran-1,3-dione (**64**) (40.0 mg, 0.178 mmol, 1.00 equiv) and hydrazine hydrate (0.357 mL, 0.500 M). The vial was sealed with a screw cap, wrapped with electrical tape, and warmed on a heating block at 100 °C for 16 hours, by which time a white precipitate formed. The reaction mixture was concentrated under reduced pressure, co-evaporating with PhMe in triplicate. Approximately 1 mL of Et_2O was added, the mixture was decanted, and the residue was dried under high vacuum to give a white/pale yellow solid. The phthalhydrazide was subjected to the next reaction without further purification.

Step 2

Stoichiometry based on theoretical yield of previous step. Conducted according to a modification of related procedures reported by Frederich[71] and Magauer.[72] A 2-dram vial containing the crude phthalhydrazide (42.5 mg, 0.178 mmol, 1.00 equiv) and a stir bar was evacuated and backfilled with argon in triplicate. $POCl_3$ (0.446 mL, 0.400 M) was added, and the vial was submerged in an ice/water bath. Pyridine (0.115 mL, 8.00 equiv) was added dropwise, then the vial was sealed with a screw cap, wrapped with electrical tape, and warmed on a heating block at 100 °C for 16 hours. The reaction was cooled to ambient temperature and then transferred into a stirring Erlenmeyer flask of ice/water using CH_2Cl_2. The mixture was stirred for 10 minutes, then basified to ~pH = 10 (pH paper) by dropwise addition of 1 M aqueous KOH. The aqueous layer was extracted with CH_2Cl_2 (×2), and the combined organic layers were washed with H_2O, brine, dried over $MgSO_4$, filtered, and concentrated under reduced pressure. The crude residue was purified by flash column chromatography (ratio of silica to crude mass = 30:1, eluting with 1:9 EtOAc:hexane to 1:5 EtOAc:hexane).

Characterization data matched those reported by Finlay.[73]

Yield: 23.0 mg, 0.0840 mmol, 47% (2 steps);

Appearance: Yellow solid;

^{1}H NMR [400 MHz, $(CD_3)_2SO$]: δ 8.46 (d, J = 8.1 Hz, 1H), 8.25 (t, J = 7.2 Hz, 1H), 8.05 (d, J = 7.3 Hz, 1H), 7.48 (s, 3H), 7.41 (s, 2H) ppm.

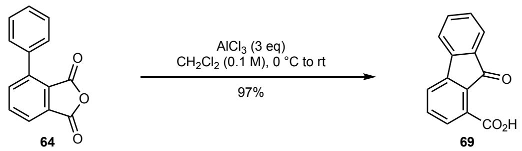

Conducted according to a modification of a related procedure reported by Schofield.[74] To a flame-dried 1-neck 10 mL round-bottom flask under argon and equipped with a stir bar was added $AlCl_3$ (53.5 mg, 0.401 mmol, 3.00 equiv) and anhydrous CH_2Cl_2 (1.34 mL, 0.100 M). The reaction flask was submerged in an ice/water bath, and 4-phenylisobenzofuran-1,3-dione (**64**) (30.0 mg, 0.134 mmol, 1.00 equiv) was added in one portion. The ice/water bath was removed, and the reaction was stirred at ambient temperature for 16 hours, by which time TLC analysis indicated complete consumption of anhydride **64**. The reaction was quenched with a 2 M aqueous solution of HCl, and the aqueous layer was extracted with CH_2Cl_2 (×2). The combined organic layers were washed with H_2O, brine, dried over $MgSO_4$, filtered, and concentrated under reduced pressure.

Characterization data matched those reported by Scott.[75]

Yield: 29.0 mg, 0.129 mmol, 97%;

Appearance: Pale orange solid;

R*f*: 0.12 (2:5 EtOAc:hexane);

^{1}H NMR (400 MHz, $CDCl_3$): δ 13.81 (s, 1H), 8.20 (dd, *J* = 7.8, 1.1 Hz, 1H), 7.75 (d, *J* = 7.5 Hz, 1H), 7.72 (dd, *J* = 7.5, 1.1 Hz, 1H), 7.66 (t, *J* = 7.6 Hz, 1H), 7.60 (td, *J* = 7.4, 1.0 Hz, 1H), 7.55 (d, *J* = 7.4 Hz, 1H), 7.37 (td, *J* = 7.4, 1.0 Hz, 1H) ppm.

From Quinone Diazide 53

summary

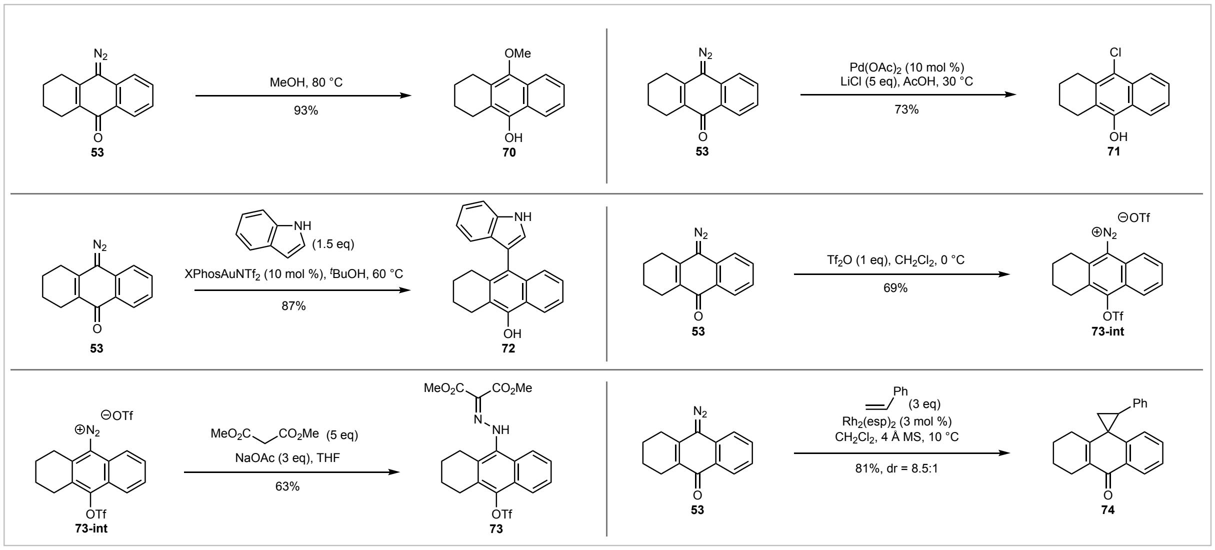

Caution: Quinone diazides are potentially explosive. Appropriate precautions should be taken during handling and storage (e.g., blast shield, avoid contact with metal spatulas, minimize exposure to heat, light, shock, and metal catalysts).

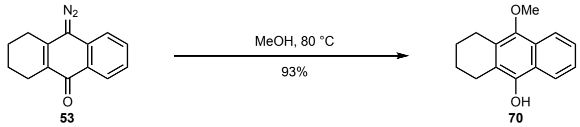

To a flame-dried 1-dram vial under argon and equipped with a stir bar was added quinone diazide **53** (20.0 mg, 0.087 mmol, 1.00 equiv) and anhydrous MeOH (0.865 ml, 0.100 M). The vial was sealed, and the reaction mixture was warmed on a heating mantle at 80 °C for 18 hours. The reaction mixture was cooled to ambient temperature and concentrated under reduced pressure.

Yield: 0.080 mmol, 93%;

Appearance: Red oil;

R*f*: N/A (decomposes on glass-backed silica TLC plates);

^{1}H NMR (400 MHz, CD_3OD): δ 8.12 (d, *J* = 8.3 Hz, 1H), 7.92 (d, *J* = 8.1 Hz, 1H), 7.36 (app p, *J* = 8.1, 2H), 3.81 (s, 3H), 2.92 (t, *J* = 6.2 Hz, 2H), 2.85 (t, *J* = 6.4 Hz, 2H), 1.89 – 1.78 (m, 4H) ppm;

^{13}C NMR (100 MHz, CD_3OD): δ 147.3, 146.7, 128.4, 127.6, 125.9, 125.9, 125.1, 122.9, 122.2, 120.8, 61.1, 25.1, 24.9, 23.9, 23.7 ppm;

IR: 3452, 3070, 2938, 2860, 1726, 1658, 1643 1596, 1452, 1379, 1291 cm^{-1};

HRMS (ESI): calculated for $[C_{15}H_{16}O_2+H]^+$: 229.1223, found: 229.1228.

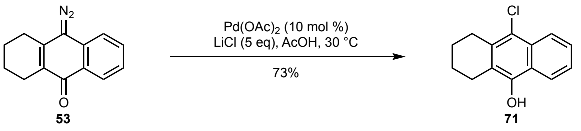

Conducted according to a modification of a related procedure reported by Kitamura.[76] To a 1-dram vial open to air and equipped with a stir bar was added quinone diazide **53** (15.0 mg, 0.067 mmol, 1.00 equiv), concentrated AcOH (0.133 ml, 0.500 M), LiCl (14.2 mg, 0.334 mmol, 5.00 equiv), and $Pd(OAc)_2$ (4.2 mg, 0.007 mmol, 0.10 equiv). The reaction was warmed on a heating mantle at 30 °C for 1 hour, cooled to 0 °C, and quenched with a saturated aqueous sodium bicarbonate solution. The reaction was extracted with CH_2Cl_2 (×3), and the combined organic layers were washed with H_2O, brine, dried over $MgSO_4$, filtered, and concentrated under reduced pressure. The crude residue was purified by flash column chromatography (ratio of silica to crude mass = 30:1, eluting with 1:9 EtOAc:hexane).

Yield: 11.4 mg, 0.049 mmol, 73%;

Appearance: Tan solid;

R*f*: 0.28 (1:9 EtOAc:hexane);

^{1}H NMR (400 MHz, $CDCl_3$): δ 8.22 (d, *J* = 8.2 Hz, 1H), 8.12 (d, *J* = 8.5 Hz, 1H), 7.52 (ddd, *J* = 8.4, 6.8, 1.5 Hz, 1H), 7.46 (ddd, *J* = 8.2, 6.8, 1.3 Hz, 1H), 5.07 (s, 1H), 3.01 (t, *J* = 6.1 Hz, 2H), 2.79 (t, *J* = 6.4 Hz, 2H), 1.94 – 1.84 (m, 4H) ppm;

^{13}C NMR (100 MHz, $CDCl_3$): δ 147.2, 134.0, 130.0, 126.7, 125.2, 124.1, 123.4, 122.7, 121.3, 118.2, 28.8, 23.7, 22.8, 22.4 ppm;

IR: 3337, 2937, 2855, 1589, 1496, 1442, 1348, 1313, 1280, 1176, 1062, 947, 906, 750, 657 cm^{-1};

HRMS (ESI): calculated for $[C_{14}H_{13}ClO-H]^-$: 231.0582, found: 231.0584.

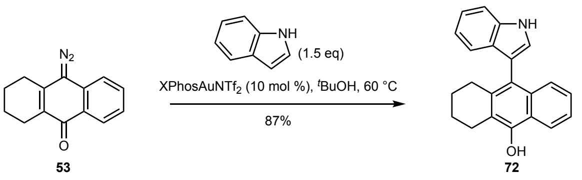

Conducted according to a modification of a related procedure reported by Zhong and Chan.[77] To a 2-dram vial open to air and equipped with a stir bar was added quinone diazide **53** (30.0 mg, 0.134 mmol, 1.00 equiv), indole (23.5 mg, 0.200 mmol, 1.50 equiv), $XPhosAuNTf_2$ (12.8 mg, 0.013 mmol, 0.100 equiv), and *tert*-butyl alcohol (1.34 ml, 0.10 M). The reaction was warmed on a heating mantle at 60 °C for 2 hours. The heating mantle was removed, and the reaction was allowed to cool to ambient temperature. The reaction was concentrated under reduced pressure, and the crude residue was purified by flash column chromatography (ratio of silica to crude mass = 50:1, eluting with 1:9 EtOAc:hexane).

Yield: 12.0 mg, 0.0380 mmol, 87%;

Appearance: Pale brown solid;

R$_f$: 0.35 (1:4 EtOAc:hexane);

^{1}H NMR (400 MHz, CD_3OD): δ 8.18 (d, J = 7.5 Hz, 1H), 7.46 (d, J = 8.3 Hz, 1H), 7.39 (d, J = 8.6 Hz, 1H), 7.27 (t, J = 7.6 Hz, 1H), 7.14 – 7.07 (m, 3H), 6.96 (d, J = 7.9 Hz, 1H), 6.90 (t, J = 7.1 Hz 1H), 2.94 (td, J = 6.2, 1.3 Hz, 2H), 2.71 – 2.62 (m, 1H), 2.55 (dt, J = 17.2, 6.2 Hz, 1H), 1.89 – 1.79 (m, 2H), 1.69 – 1.58 (m, 2H) ppm;

^{13}C NMR (100 MHz, CD_3OD): δ 149.6, 137.9, 137.6, 134.7, 129.6, 127.3, 125.5, 125.2, 125.0, 124.5, 124.2, 122.3, 122.2, 120.4, 120.3, 119.9, 114.7, 112.3, 30.2, 25.2, 24.4, 24.0 ppm;

IR: 3054, 2933, 2055, 1614, 1576, 1547, 1282, 1172, 1121, 736, 694 cm^{-1};

HRMS (ESI): calculated for $[C_{22}H_{19}NO+Na]^+$: 336.1359, found: 336.1354.

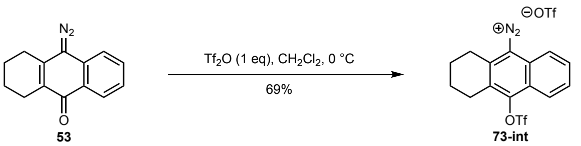

Conducted according to a modification of a related procedure reported by Maas.[78] To a flame-dried 1-neck 50 mL round-bottom flask under argon and equipped with a stir bar was added quinone diazide **53** (250.0 mg, 1.12 mmol, 1.00 equiv) and anhydrous CH_2Cl_2 (22.3 mL, 0.0500 M). The reaction flask was submerged in an ice/water bath and a solution of trifluoromethanesulfonic anhydride (1.12 mmol, 0.187 ml, 1.00 equiv) in anhydrous CH_2Cl_2 (2.23 mL) was added dropwise via syringe. The reaction mixture was stirred for 15 minutes and anhydrous Et_2O (ca. 15 ml) was added. The flask was sealed, transferred to a −20 °C freezer for 3 hours, filtered, and the resulting solid was washed with cold Et_2O.

Yield: 396.9 mg, 0.764 mmol, 69%;

Appearance: Pale yellow solid;

R$_f$: N/A (decomposes on glass-backed silica TLC plates);

^{1}H NMR (400 MHz, CD_3OD): δ 8.46 (d, J = 8.4 Hz, 1H), 8.39 (d, J = 8.7 Hz, 1H), 8.17 (t, J = 7.8 Hz, 1H), 8.07 (t, J = 7.9 Hz, 1H), 3.50 (t, J = 6.4 Hz, 2H), 3.16 (t, J = 6.3 Hz, 2H), 2.14 – 2.08 (m, 2H), 2.04 – 1.98 (m, 2H) ppm;

^{13}C NMR (100 MHz, CD_3OD): δ 153.5, 152.7, 134.5, 133.9, 132.2, 129.7, 127.1, 124.7, 122.6, 121.8 (q, J = 317.2 Hz), 120.0 (q, J = 319.3 Hz), 111.3, 28.9, 25.8, 21.8, 21.4 ppm;

^{19}F NMR (377 MHz, CD_3OD): δ −73.9, −80.2 ppm;

IR: 2964, 2248, 1563, 1430, 1243, 1214, 1148, 1026, 844, 751, 674 cm^{-1};

HRMS (ESI): calculated for $[C_{16}H_{12}F_6N_2O_6S_2{-}CF_3O_3S]^+$: 357.0515, found: 357.0514.

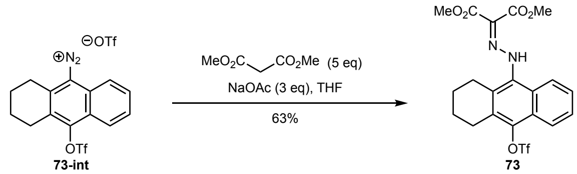

Conducted according to a modification of a related procedure reported by Scammells.[79] To a flame-dried 2-dram vial under argon and equipped with a stir bar was added triflate salt **73-int** (15.0 mg, 0.0310 mmol, 1.00 equiv), dimethyl malonate (0.157 mmol, 0.018 ml, 5.00 equiv), sodium acetate (7.70 mg, 0.0940 mmol, 3.00 equiv) and anhydrous THF (0.209 mL, 0.150 M). The vial was sealed, and the reaction mixture was stirred at ambient temperature for 1 hour before being diluted with H_2O. The crude reaction mixture was extracted with EtOAc (×3), and the combined organic layers were washed with H_2O, brine, dried over $MgSO_4$, filtered, and concentrated under reduced pressure. The crude residue was purified by flash column chromatography (ratio of silica to crude mass = 30:1, eluting with 1:9 EtOAc:hexane).

Yield: 9.6 mg, 0.020 mmol, 63%;

Appearance: Yellow solid;

R*f*: 0.40 (1:4 EtOAc:hexane);

^{1}H NMR (400 MHz, $CDCl_3$): δ 13.04 (br s, 1H), 8.24 (dd, J = 7.7, 2.2 Hz, 1H), 8.06 (dd, J = 7.6, 1.9 Hz, 1H), 7.58 (pd, J = 7.0, 1.6 Hz, 2H), 3.98 (s, 3H), 3.85 (s, 3H), 3.05 (t, J = 6.2 Hz, 2H), 2.98 (t, J = 6.4 Hz, 2H), 1.95 – 1.80 (m, 4H) ppm;

^{13}C NMR (100 MHz, $CDCl_3$): δ 164.2, 163.6, 141.4, 134.4, 130.2, 129.7, 127.5, 127.4, 127.2, 126.2, 123.0, 121.3, 120.3, 118.9 (q, J = 320.1 Hz), 52.6, 52.5, 26.0, 25.1, 22.2, 21.7 ppm;

^{19}F NMR (377 MHz, $CDCl_3$): δ −72.6 ppm;

IR: 2938, 2058, 1727, 1582, 1534, 1407, 1208, 1006, 847, 754 cm^{-1};

HRMS (ESI): calculated for $[C_{20}H_{19}N_2O_7S+Na]^+$: 511.0757, found: 511.0755.

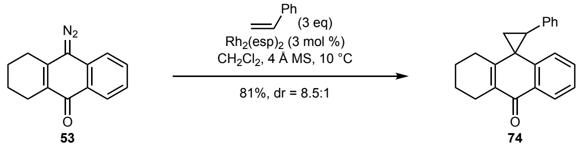

Conducted according to a modification of a related procedure reported by Baran.[80] To a flame-dried 2-dram vial under argon and equipped with a stir was added 4 Å molecular sieves (activated powder, 200 mg), styrene (57.8 mg, 0.555 mmol, 3.00 equiv), anhydrous CH_2Cl_2 (6.93 ml, 0.0800 M), and $Rh_2(esp)_2$ (4.30 mg, 6.00 μmol, 3.00 mol %). Argon was bubbled through the solution for five minutes and the flask was sealed and submerged in an ethanol bath maintained at 10 °C by an immersion cooler (Julabo FT902). A solution of quinone diazide **53** (50.0 mg, 0.185 mmol, 1.00 equiv) in anhydrous CH_2Cl_2 (1.85 ml, 0.100 M) was added dropwise via syringe pump over 2 hours, and the reaction was stirred for an additional 1 hour. The cooling bath was removed, and the reaction mixture was filtered over Celite (eluting with CH_2Cl_2) then concentrated under reduced pressure. The crude residue was purified

by flash column chromatography (ratio of silica to crude mass = 30:1, eluting with 1:9 Et_2O:hexane) to yield cyclopropane **74** as 8.5:1 mixture of diastereoisomers. **Note**: Upon standing for 24 hours, an ^{1}H NMR sample of **74** in $CDCl_3$ was found to convert from an 8.5:1 to a 1:0.9 mixture of diastereoisomers. For simplicity, we elected to characterize **74** as this 1:0.9 mixture of diastereoisomers.

Yield: 45.0 mg, 0.150 mmol, 81%;

Appearance: Yellow oil;

R*f*: 0.26 (1:4 Et_2O:hexane);

^{1}H NMR (400 MHz, CD_2Cl_2, both diastereoisomers indicated): δ 8.21 (dd, *J* = 7.8, 1.6 Hz, 0.9H), 8.12 (dd, *J* = 7.8, 1.6 Hz, 1H), 7.56 (td, *J* = 7.7, 1.6 Hz, 0.9H), 7.42 – 7.37 (td, *J* = 7.5, 0.7 Hz, 1H), 7.32 – 7.07 (m, 10.7H), 7.04 – 6.95 (m, 3.5H), 6.42 (d, *J* = 8.2 Hz, 1H), 3.22 (t, *J* = 8.8 Hz, 1H), 2.96 (t, *J* = 8.8 Hz, 1H), 2.67 – 2.43 (m, 5.2H), 2.41 – 2.33 (m, 3.3H), 2.27 – 2.23 (m, 2H), 1.87 – 1.66 (m, 5.9H), 1.18 – 1.02 (m, 2.6H) ppm;

^{13}C NMR (100 MHz, CD_2Cl_2, both diastereoisomers indicated): δ 184.8, 184.7, 154.7, 153.5, 147.0, 141.5, 137.4, 136.7, 136.1, 133.7, 133.6, 132.5, 132.4, 130.6, 130.0, 129.9, 128.8, 128.4, 127.6, 127.2, 126.8, 126.7, 126.1, 125.8, 124.6, 121.5, 43.1, 38.1, 32.5, 32.1, 29.1, 26.2, 24.1, 23.9, 22.9, 22.4, 22.2, 22.1, 21.4, 20.8 ppm;

IR: 3026, 2929, 2858, 1628, 1599, 1460, 1307, 1174, 957, 762, 699 cm^{-1};

HRMS (ESI): calculated for $[C_{22}H_{20}O_4+H]^+$: 301.1587, found: 301.1585.

Synthesis of Pomalidomide, Apremilast, and Related Derivatives

Dienophile Synthesis

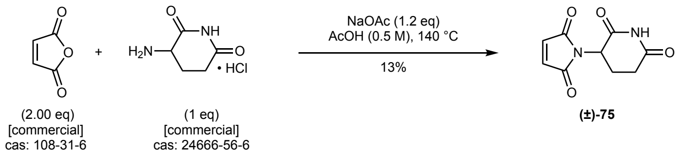

Conducted according to a modification of a related procedure reported by Park.[81] To a 1-neck 100 mL round-bottom flask open to air and equipped with a stir bar and high-efficiency air condenser was added 3-aminopiperidine-2,6-dione hydrochloride (3.36 g, 20.4 mmol, 1.00 equiv), maleic anhydride (4.00 g, 40.8 mmol, 2.00 equiv), NaOAc (2.01 g, 24.5 mmol, 1.20 equiv), and AcOH (40.8 mL, 0.500 M). The reaction flask was warmed on a heating mantle to 140 °C for 24 hours, turning dark brown. The reaction was cooled to ambient temperature, concentrated under reduced pressure, then diluted with H_2O and extracted with EtOAc (×3). The combined organic layers were washed with saturated aqueous $NaHCO_3$ solution, brine, dried over $MgSO_4$, filtered, and concentrated under reduced pressure. The crude residue was purified by flash column chromatography (ratio of silica to crude mass = 50:1, eluting with 1:1 EtOAc:hexane).

Characterization data matched those reported by Waser.[82]

Yield: 555 mg, 2.67 mmol, 13%;

Appearance: White solid;

R*f*: 0.33 (3:1 EtOAc:hexane);

^{1}H NMR [400 MHz, $(CD_3)_2SO$]: δ 11.07 (s, 1H), 7.13 (s, 2H), 4.96 (dd, *J* = 13.2, 5.4 Hz, 1H), 2.91 – 2.75 (m, 1H), 2.56 (d, *J* = 18.6 Hz, 1H), 2.42 (qd, *J* = 13.1, 4.0 Hz, 1H), 2.03 – 1.91 (m, 1H) ppm.

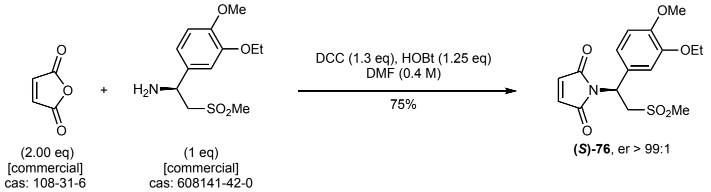

Conducted according to a modification of a related procedure reported by Imperiali.[83] To a flame-dried 3-neck 100 mL round-bottom flask under argon and equipped with a stir bar was added (*S*)-1-(3-ethoxy-4-methoxyphenyl)-2-(methylsulfonyl)ethan-1-amine (1.50 g, 5.49 mmol, 1.00 equiv), anhydrous DMF (18.3 mL, 0.400 M), and maleic anhydride (646 mg, 6.59 mmol, 1.20 equiv). The reaction was stirred at ambient temperature for two hours and then DCC (1.47 g, 7.13 mmol, 1.30 equiv) and HOBt (1.05 g, 6.86 mmol, 1.25 equiv) were added. The reaction was stirred for a further 24 hours and then diluted with EtOAc (ca. ×2 reaction volume) and filtered. The filtrate was diluted with H_2O, the layers were separated, and the aqueous layer was extracted with EtOAc (×2). The combined organic layers were washed with a saturated aqueous solution of $NaHCO_3$, brine, dried over $MgSO_4$, filtered, and concentrated under reduced pressure. The crude residue was purified by flash column chromatography (ratio of silica to crude mass = 50:1, eluting with 2:3 EtOAc:hexane).

Yield: 1.46 g, 4.12 mmol, 75%;

Appearance: White solid;

R$_f$: 0.33 (3:1 EtOAc:hexane);

^{1}H NMR (400 MHz, $CDCl_3$): δ 7.02 (app d, J = 11.8 Hz, 2H), 6.82 (d, J = 8.2 Hz, 1H), 6.68 (s, 2H), 5.69 (dd, J = 10.7, 4.2 Hz, 1H), 4.40 (dd, J = 14.4, 10.5 Hz, 1H), 4.08 (q, J = 6.9 Hz, 2H), 3.84 (s, 3H), 3.63 (dd, J = 14.4, 4.6 Hz, 1H), 2.82 (s, 3H), 1.45 (t, J = 6.9 Hz, 3H) ppm;

^{13}C NMR (100 MHz, $CDCl_3$): δ 170.3, 149.8, 148.8, 134.4, 129.6, 120.4, 112.4, 111.6, 64.6, 56.1, 54.7, 48.8, 41.7, 14.8 ppm;

HRMS (ESI): calculated for $[C_{16}H_{19}NO_6S+Na]^+$: 376.0825, found: 376.0815;

Chiral HPLC [Chiralpak IA, 4.6 x 250 mm; 12% iPrOH/hexane, 1.0 mL/min, 210 nm]: t_R (minor) = 50.2 min, t_R (major) = 13.76 min, er > 99:1;

[α]D^{20}: +20.2 (c = 1.63, $CHCl_3$).

HPLC trace (racemate):

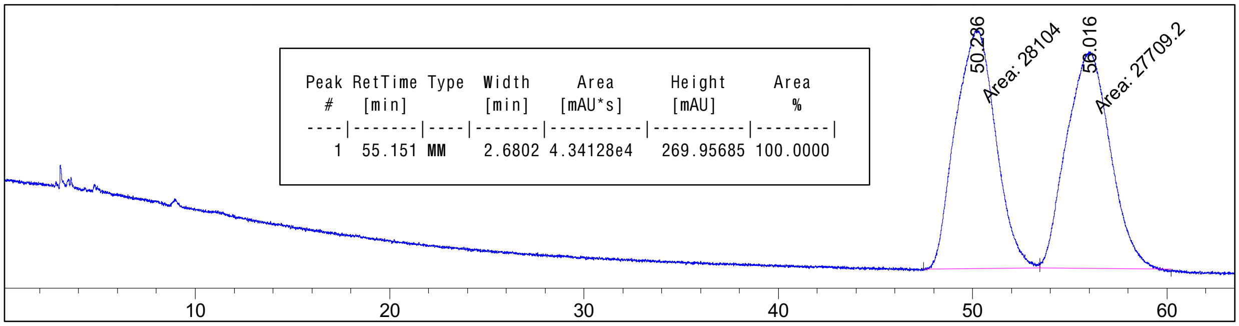

HPLC trace (enantioenriched):

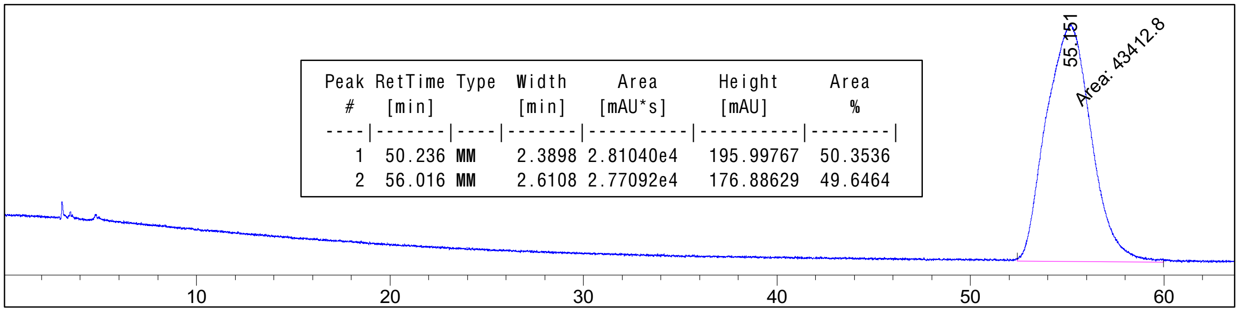

Diels–Alder/Acylation Sequences

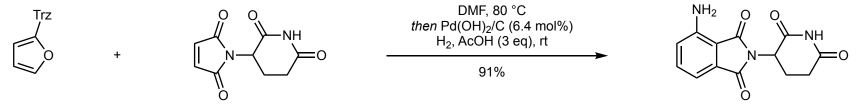

(1 eq)
2-triazenyl-furan-01 (or **1**)

(2 eq)
(±)-75

(±)-pomalidomide (77)

To a 2-dram vial open to air and equipped with a stir bar was added **2-triazenyl-furan-01** (or **1**) (30.0 mg, 0.182 mmol, 1.00 equiv), **(±)-75** (75.6 mg, 0.363 mmol, 2.00 equiv), and DMF (0.363 mL, 0.500 M). The vial was sealed with a screw cap, wrapped with electrical tape, and warmed on a heating block at 80 °C for 24 hours, by which time TLC analysis indicated complete consumption of the triazenyl furan. The reaction was cooled to ambient temperature, and DMF (0.545 mL, 0.200 M total concentration), AcOH (0.0321 mL, 0.545 mmol, 3.00 equiv), and 10% $Pd(OH)_2/C$ (16.3 mg, 6.4 mol% based on theoretical yield of the direct Diels–Alder adduct, 0.0116 mmol) were added. The vial was fitted with a septum, and H_2 (balloon) was bubbled through the solution for 10 minutes. The reaction was stirred under an atmosphere of H_2 (balloon still attached) for 24 hours, then filtered over a pad of celite, eluting with EtOAc. The filtrate was washed with H_2O (×3), brine, dried over $MgSO_4$, filtered, and concentrated under reduced pressure. The crude residue was purified by flash column chromatography (ratio of silica to crude mass = 100:1, eluting with 1:1 EtOAc:hexane to 3:1 EtOAc:hexane).

Characterization data matched those reported by Gütschow and Krönke.[84]

Yield: 45.0 mg, 0.165 mmol, 91%;

Appearance: Yellow solid;

R*f*: 0.32 (3:1 EtOAc:hexane);

^{1}H NMR [400 MHz, $(CD_3)_2SO$]: δ 11.08 (s, 1H), 7.47 (dd, *J* = 8.5, 7.0 Hz, 1H), 7.01 (t, *J* = 7.7 Hz, 2H), 6.51 (br s, 2H), 5.04 (dd, *J* = 12.8, 5.3 Hz, 1H), 2.94 – 2.82 (m, 1H), 2.64 – 2.52 (m, 2H), 2.06 – 1.98 (m, 1H) ppm.

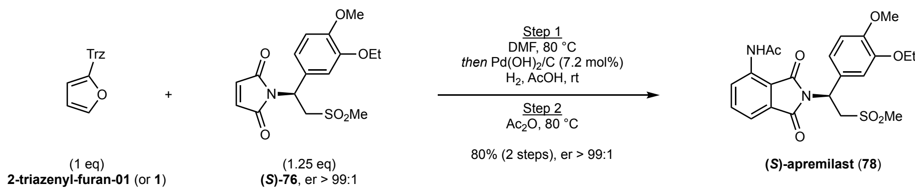

Step 1

To a 2-dram vial open to air and equipped with a stir bar was added **2-triazenyl-furan-01** (or **1**) (30.0 mg, 0.182 mmol, 1.00 equiv), ***(S)*-76** (80.2 mg, 0.227 mmol, 1.25 equiv), and DMF (0.363 mL, 0.500 M). The vial was sealed with a screw cap, wrapped with electrical tape, and warmed on a heating block at 80 °C for 24 hours, by which time TLC analysis indicated complete consumption of **2-triazenyl-furan-01** (or **1**). The reaction was cooled to ambient temperature, and DMF (1.82 mL total volume, 0.200 M total concentration), AcOH (0.0312 mL, 0.545 mmol, 3.00 equiv) and 10% $Pd(OH)_2/C$ (18.2 mg, 7.2 mol% based on theoretical yield of the direct Diels–Alder adduct, 0.0131 mmol) were added. The vial was fitted with a septum, and H_2 (balloon) was bubbled through the solution for 10 minutes. The reaction was stirred under an atmosphere of H_2 (balloon still attached) for 24 hours, then filtered over a pad of celite, eluting with EtOAc. The filtrate was washed with H_2O (×3), brine, dried over $MgSO_4$, filtered, and concentrated under reduced pressure. The crude residue was subjected to the next reaction without purification.

Step 2

Conducted according to a modification of a related procedure reported by Wu.[85] Stoichiometry is based on the theoretical yield of Step 1. To a 2-dram vial open to air and charged with the crude aniline (76.0 mg, 0.182 mmol, 1.00 equiv) and a stir bar was added Ac_2O (0.446 mL, 0.407 M). The vial was sealed with a screw cap, wrapped with electrical tape, warmed on a heating block at 70 °C for 3 hours, and then concentrated under reduced pressure (co-evaporating with PhMe in triplicate). The crude residue was purified by flash column chromatography (ratio of silica to crude mass = 100:1, eluting with 1:1 EtOAc:hexane to 2:1 EtOAc:hexane).

Characterization data matched those reported by Wu.[85]

Yield: 67.3 mg, 0.146 mmol, 80% (2 steps);

Appearance: Yellow solid;

$\mathbf{R_f}$: 0.14 (2:1 EtOAc:hexane);

^{1}H NMR (400 MHz, $CDCl_3$): δ 9.46 (s, 1H), 8.76 (d, J = 8.7 Hz, 1H), 7.65 (t, J = 7.5 Hz, 1H), 7.49 (d, J = 6.6 Hz, 1H), 7.10 (app d, J = 8.3 Hz, 2H), 6.84 (d, J = 8.1 Hz, 1H), 5.87 (dd, J = 10.6, 3.5 Hz, 1H), 4.56 (dd, J = 13.8, 11.2 Hz, 1H), 4.11 (q, J = 6.7 Hz, 2H), 3.85 (s, 3H), 3.72 (dd, J = 14.5, 4.0 Hz, 1H), 2.88 (app d, J = 6.9 Hz, 3H), 2.26 (s, 3H), 1.47 (t, J = 6.9 Hz, 3H) ppm;

Chiral HPLC [Chiralpak IA, 4.6 x 250 mm; 50% (0.01% acetic acid in MeOH) / hexane, 1.0 mL/min, 230 nm]: t_R (minor) = 8.68 min, t_R (major) = 13.76 min, er > 99:1;

$[\alpha]D^{20}$: +21.5 (c = 0.55, $CHCl_3$), literature: +30.6 (c = 2.00, $CHCl_3$).[85]

HPLC trace (racemate):

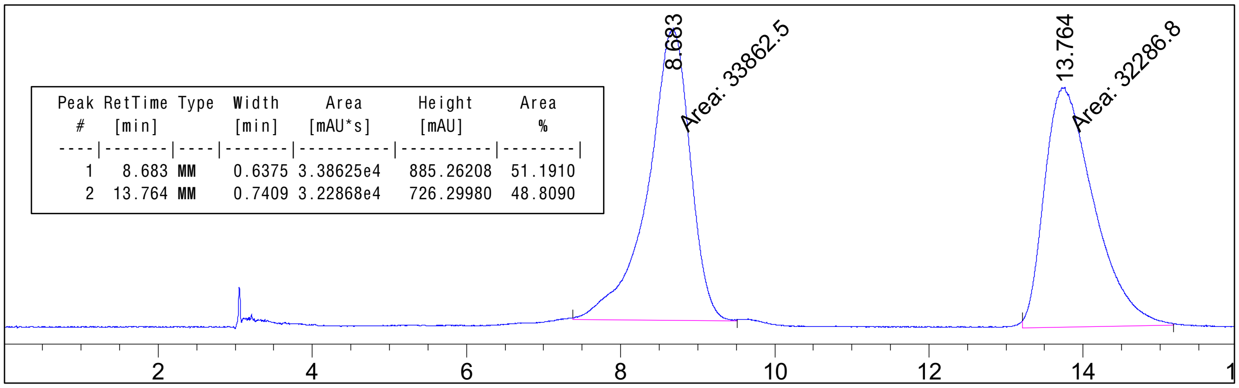

Peak #	RetTime [min]	Type	Width [min]	Area [mAU*s]	Height [mAU]	Area %
1	8.683	MM	0.6375	3.38625e4	885.26208	51.1910
2	13.764	MM	0.7409	3.22868e4	726.29980	48.8090

HPLC trace (enantioenriched):

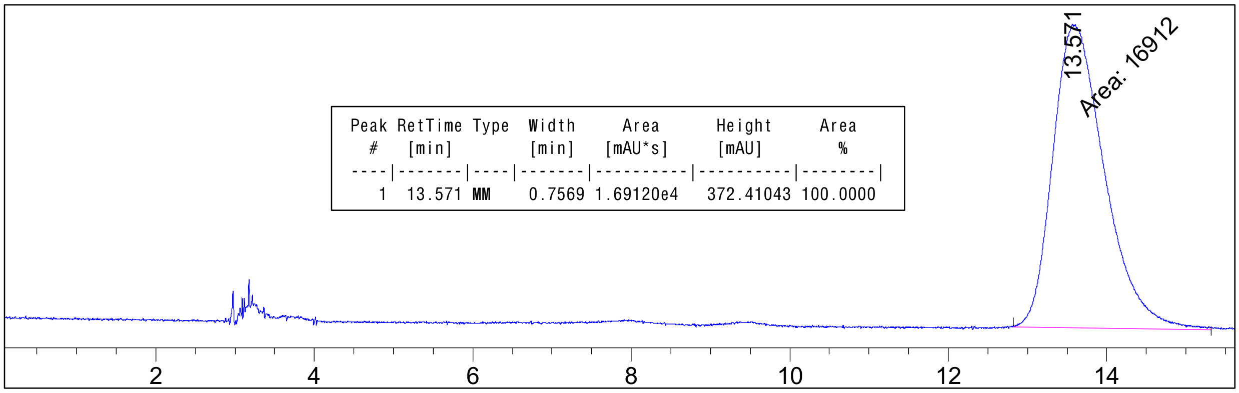

Peak #	RetTime [min]	Type	Width [min]	Area [mAU*s]	Height [mAU]	Area %
1	13.571	MM	0.7569	1.69120e4	372.41043	100.0000

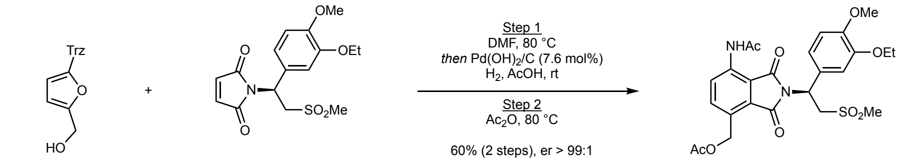

(1 eq)
2-triazenyl-furan-27

(1.25 eq)
(*S*)-76, er > 99:1

(*S*)-79

Step 1

To a 2-dram vial open to air and equipped with a stir bar was added **2-triazenyl-furan-27** (30.0 mg, 0.154 mmol, 1.00 equiv), **(*S*)-76** (67.9 mg, 0.192 mmol, 1.25 equiv), and DMF (0.307 mL, 0.500 M). The vial was sealed with a screw cap, wrapped with electrical tape, and warmed on a heating block at 80 °C for 24 hours, by which time TLC analysis indicated complete consumption of **2-triazenyl-furan-27**. The reaction was cooled to ambient temperature, and DMF (1.54 mL total volume, 0.200 M total concentration), AcOH (0.0264 mL, 0.461 mmol, 3.00 equiv) and 10 $Pd(OH)_2/C$ (16.3 mg, 7.6 mol% based on theoretical yield of the direct Diels–Alder adduct, 0.0117 mmol) were added. The vial was fitted with a septum, and H_2 (balloon) was bubbled through the solution for 10 minutes. The reaction was stirred under an atmosphere of H_2 (balloon still attached) for 24 hours, then filtered over a pad of celite, eluting with EtOAc. The filtrate was washed with H_2O (×3), brine, dried over $MgSO_4$, filtered, and concentrated under reduced pressure. The crude residue was subjected to the next reaction without purification.

Step 2

Conducted according to a modification of a related procedure reported by Wu.[85] Stoichiometry is based on the theoretical yield of Step 1. To a 2-dram vial open to air and charged with the crude aniline (68.9 mg, 0.154 mmol, 1.00 equiv) and a stir bar was added Ac_2O (0.377 mL, 0.407 M). The vial was sealed with a screw cap, wrapped with electrical tape, warmed on a heating block at 70 °C for 3 hours, and then concentrated under reduced pressure (co-evaporating with PhMe in triplicate). The crude residue was purified by flash column chromatography (ratio of silica to crude mass = 100:1, eluting with 2:5 EtOAc:hexane to 2:1 EtOAc:hexane).

Yield: 48.7 g, 0.0914 mmol, 60%;

Appearance: Yellow solid;

R*f*: 0.29 (3:1 EtOAc:hexane);

^{1}H NMR (400 MHz, $CDCl_3$): δ 9.53 (s, 1H), 8.74 (d, J = 8.7 Hz, 1H), 7.64 (d, J = 8.7 Hz, 1H), 7.09 (app d, J = 9.0 Hz, 2H), 6.84 (d, J = 8.2 Hz, 1H), 5.85 (dd, J = 10.4, 1.1 Hz, 1H), 5.48 (s, 2H), 4.54 (t, J = 1.9 Hz, 1H), 4.11 (q, J = 7.0 Hz, 2H), 3.85 (s, 3H), 3.71 (dd, J = 13.9, 3.8 Hz, 1H), 2.87 (s, 3H), 2.26 (s, 3H), 2.11 (s, 3H), 1.47 (t, J = 6.9 Hz, 3H) ppm;

^{13}C NMR (100 MHz, $CDCl_3$): δ 170.7, 169.4, 169.3, 167.3, 150.0, 148.8, 137.5, 136.2, 130.0, 129.3, 127.5, 125.2, 120.5, 115.3, 112.6, 111.6, 64.7, 60.8, 56.1, 54.5, 48.7, 41.8, 25.1, 20.9, 14.8 ppm;

IR: 3357, 2932, 1698, 1518, 1304, 1242, 1139, 1027, 763 cm^{-1};

HRMS (ESI): calculated for $[C_{25}H_{28}N_2O_9S+Na]^+$: 555.1408, found: 555.1398;

Chiral HPLC (Chiralpak IA, 4.6 x 250 mm; 30% *i*PrOH/hexane, 1.0 mL/min, 230 nm): t_R (major) = 33.9 min, t_R (minor) = 45.8 min, er > 99:1;

[α]D[20]: +18.5 (c = 0.48, $CHCl_3$).

HPLC trace (racemate):

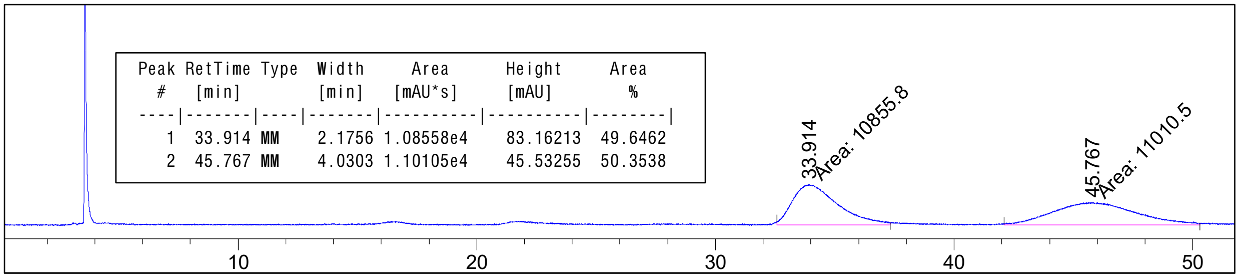

HPLC trace (enantioenriched):

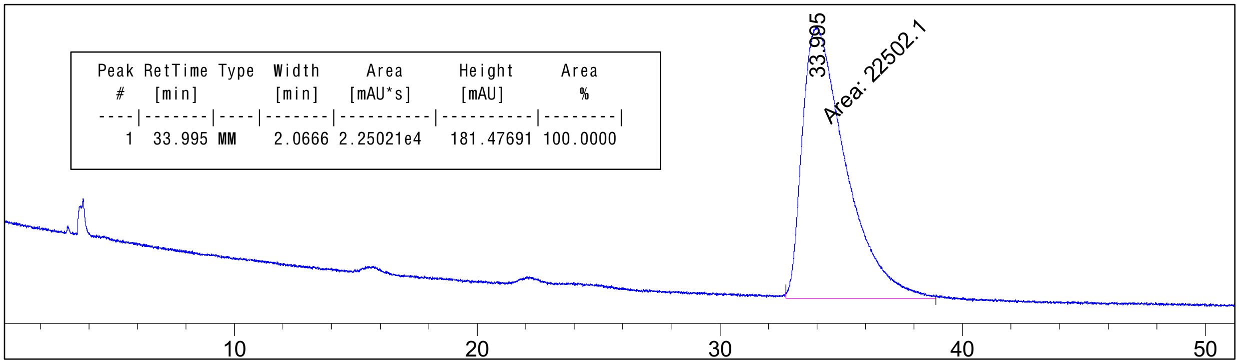

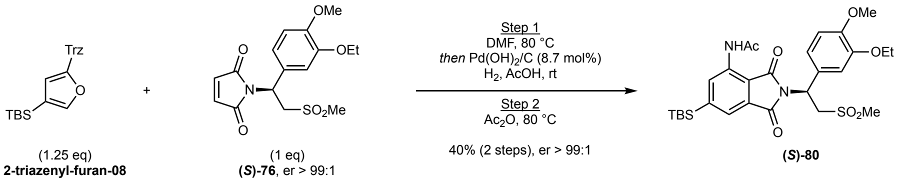

Note: Stoichiometry flipped to aid purification.

Step 1

To a 2-dram vial open to air and equipped with a stir bar was added **2-triazenyl-furan-08** (36.2 mg, 0.130 mmol, 1.25 equiv), **(*S*)-76** (36.6 mg, 0.104 mmol, 1.00 equiv), and DMF (0.207 mL, 0.500 M). The vial was sealed with a screw cap, wrapped with electrical tape, and warmed on a heating block at 80 °C for 24 hours, by which time TLC analysis indicated complete consumption of **(*S*)-76**. The reaction was cooled to ambient temperature, and DMF (1.04 mL total volume, 0.100 M total concentration), AcOH (0.0178 mL, 0.311 mmol, 3.00 equiv) and 10% $Pd(OH)_2/C$ (12.7 mg, 8.7 mol% based on theoretical yield of direct Diels–Alder adduct, 0.00902 mmol) were added. The vial was fitted with a septum, and H_2 (balloon) was bubbled through the solution for 10 minutes. The reaction was stirred under an atmosphere of H_2 (balloon still attached) for 24 hours, then filtered over a pad of celite, eluting with EtOAc. The filtrate was washed with H_2O (×3), brine, dried over $MgSO_4$, filtered, and concentrated under reduced pressure. The crude residue was subjected to the next reaction without purification.

Step 2

Conducted according to a modification of a related procedure reported by Wu.[85] Stoichiometry is based on the theoretical yield of Step 1. To a 2-dram vial open to air and charged with the crude aniline (0.104 mmol, 1.00 equiv) and a stir bar was added Ac_2O (0.255 mL, 0.407 M). The vial was sealed with a screw cap, wrapped with electrical tape, warmed on a heating block at 70 °C for 3 hours, and then concentrated under reduced pressure (co-evaporating with PhMe in triplicate). The crude residue was purified by flash column chromatography (ratio of silica to crude mass = 100:1, eluting with 1:2 EtOAc:hexane to 2:5 EtOAc:hexane).

Yield: 23.9 mg, 0.0416 mmol, 40%;

Appearance: Yellow solid;

R*f*: 0.18 (1:1 EtOAc:hexane);

^{1}H NMR (400 MHz, $CDCl_3$): δ 9.38 (s, 1H), 8.96 (s, 1H), 7.61 (s, 1H), 7.10 (d, *J* = 7.1 Hz, 2H), 6.84 (d, *J* = 8.2 Hz, 1H), 5.87 (dd, *J* = 9.4, 1.3 Hz, 1H), 4.56 (dd, *J* = 14.4, 10.4 Hz, 1H), 4.11 (q, *J* = 6.6 Hz, 2H), 3.85 (s, 3H), 3.73 (dd, *J* = 14.8, 4.0 Hz, 1H), 2.87 (s, 3H), 2.26 (s, 3H), 1.47 (t, *J* = 6.6 Hz, 3H), 0.88 (s, 9H), 0.30 (s, 6H) ppm;

^{13}C NMR (100 MHz, $CDCl_3$): δ 169.8, 169.3, 168.3, 150.3, 149.9, 148.8, 136.7, 131.0, 129.8, 129.5, 123.7, 120.5, 115.5, 112.6, 111.6, 64.7, 56.1, 54.8, 48.8, 41.8, 26.6, 25.1, 17.1, 14.9, −6.0 ppm;

IR: 2925, 2873, 1595, 1498, 1326, 875, 751 cm^{-1};

HRMS (ESI): calculated for $[C_{28}H_{38}N_2O_7SSi+Na]^+$: 597.2061, found: 597.2044;

Chiral HPLC (Chiralpak IA, 4.6 x 250 mm; 30% *i*PrOH/hexane, 1.0 mL/min, 230 nm): t_R (minor) = 11.2 min, t_R (major) = 17.1 min, er > 99:1;

[α]D[20]: +21.4 (c = 0.49, $CHCl_3$).

<u>HPLC trace (racemate):</u>

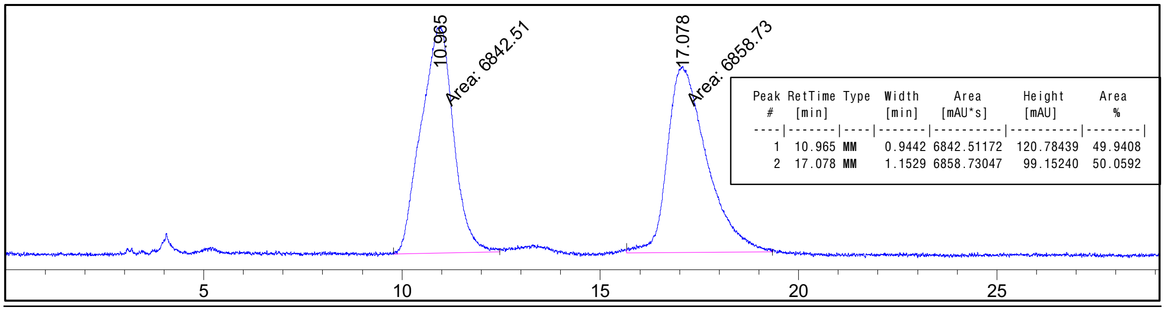

<u>HPLC trace (enantioenriched):</u>

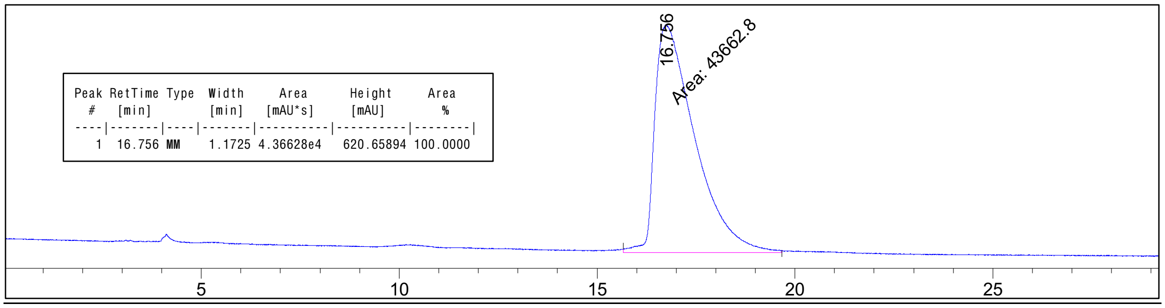

X-Ray Crystallographic Data

Crystallographic data for **scouting-02** can be obtained free of charge from the Cambridge Crystallographic Data Centre (CCDC) via www.ccdc.cam.ac.uk/data_request/cif under CCDC deposition number CCDC 2537278.

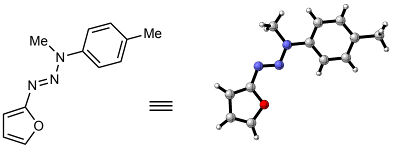

scouting-02 (X-ray, CCDC 2537278)

Crystal data and structure refinement for **scouting-02**:

Identification code	j1_a
Empirical formula	$C_{12}H_{13}N_3O$
Formula weight	215.25
Temperature	299(2) K
Wavelength	0.71073 A
Crystal system, space group	Monoclinic, P2 (1)
Unit cell dimensions	
a	5.1568(4) A
b	9.4428(8) A
c	11.9726(9) A
α	90 °
β	92.119(3) °
γ	90 °
Volume	582.60(8) A^3
Z, Calculated density	2, 1.227 Mg/m^3
Absorption coefficient	0.082 mm^{-1}
F(000)	228
Crystal size	0.400 × 0.360 × 0.100 mm
Θ range for data collection	2.748 to 25.679 °
Limiting indices	$-6 \leq h \leq 6, -11 \leq k \leq 5, -14 \leq l \leq 14$
Reflections collected / unique	11569 / 2195 [R_{int} = 0.0893]
Completeness to θ	25.242 99.3%
Absorption correction	Semi-empirical from equivalents
Max. and min. transmission	0.7453 and 0.4549
Refinement method	Full-matrix least-squares on F^2
Data / restraints / parameters	2195 / 1 / 145
Goodness-of-fit on F^2	1.036
Final R indices [I>2σ(I)]	R_1 = 0.0527, wR_2 = 0.1320
R indices (all data)	R_1 = 0.0713, wR_2 = 0.1487
Extinction coefficient	N/A
Largest diff. peak and hole	0.147 and −0.135 $e.Å^{-3}$

Crystallographic data for **1** can be obtained free of charge from the Cambridge Crystallographic Data Centre (CCDC) via www.ccdc.cam.ac.uk/data_request/cif under CCDC deposition number CCDC 2537282.

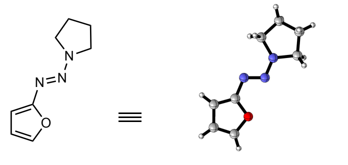

Crystal data and structure refinement for **1**.

Identification code	j1_a
Empirical formula	$C_8H_{11}N_3O$
Formula weight	215.25
Temperature	299(2) K
Wavelength	0.71073 A
Crystal system, space group	Monoclinic, P2(1)/n
Unit cell dimensions<br>a<br>b<br>c<br>α<br>β<br>γ	 5.9269(2) A<br>13.5830(5) A<br>10.7855(4) A<br>90 °<br>101.7820(10) °<br>90 °
Volume	849.99(5) A^3
Z, Calculated density	4, 1.291 Mg/m^3
Absorption coefficient	0.089 mm^{-1}
F(000)	352
Crystal size	0.440 × 0.350 × 0.140 mm
Θ range for data collection	2.999 to 34.748 °
Limiting indices	$-9 \leq h \leq 9$, $-21 \leq k \leq 21$, $-17 \leq l \leq 17$
Reflections collected / unique	36055 / 3674 [R_{int} = 0.1198]
Completeness to θ	25.242 99.9%
Absorption correction	Semi-empirical from equivalents
Max. and min. transmission	0.7481 and 0.3868
Refinement method	Full-matrix least-squares on F^2
Data / restraints / parameters	3674 / 114 / 146
Goodness-of-fit on F^2	1.019
Final R indices [I>2σ(I)]	R_1 = 0.0656, wR_2 = 0.1588
R indices (all data)	R_1 = 0.1188, wR_2 = 0.1894
Extinction coefficient	N/A
Largest diff. peak and hole	0.217 and −0.321 e.$Å^{-3}$

Crystallographic data for **7** can be obtained free of charge from the Cambridge Crystallographic Data Centre (CCDC) via www.ccdc.cam.ac.uk/data_request/cif under CCDC deposition number CCDC 2537280.

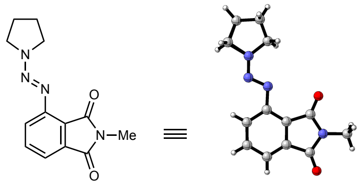

7 (X-ray, CCDC 2537280)

Crystal data and structure refinement for **7**.

Identification code	j1_a
Empirical formula	$C_{13}H_{14}N_4O_4$
Formula weight	258.28
Temperature	299(2) K
Wavelength	0.71073 A
Crystal system, space group	Monoclinic, P2 (1)/n
Unit cell dimensions	
a	5.8503(3) A
b	16.7923(8) A
c	12.6841(7) A
α	90 °
β	90.376(2) °
γ	90 °
Volume	1246.06(11) A^3
Z, Calculated density	4, 1.377 Mg/m^3
Absorption coefficient	0.097 mm^{-1}
F(000)	544
Crystal size	0.400 × 0.240 × 0.080 mm
Θ range for data collection	2.012 to 26.014 °
Limiting indices	$-7 \le h \le 7$, $-20 \le k \le 20$, $-15 \le l \le 15$
Reflections collected / unique	27389 / 2447 [R_{int} = 0.0669]
Completeness to θ	25.242 99.9%
Absorption correction	Semi-empirical from equivalents
Max. and min. transmission	0.7454 and 0.4981
Refinement method	Full-matrix least-squares on F^2
Data / restraints / parameters	2447 / 114 / 210
Goodness-of-fit on F^2	1.052
Final R indices [I>2σ(I)]	R_1 = 0.0437, wR_2 = 0.1094
R indices (all data)	R_1 = 0.0556, wR_2 = 0.1181
Extinction coefficient	N/A
Largest diff. peak and hole	0.246 and −0.188 e.$Å^{-3}$

Crystallographic data for **39** can be obtained free of charge from the Cambridge Crystallographic Data Centre (CCDC) via www.ccdc.cam.ac.uk/data_request/cif under CCDC deposition number CCDC 2537279.

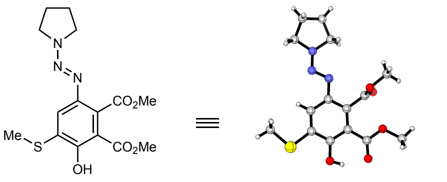

Crystal data and structure refinement for **39**.

Identification code	j1_a
Empirical formula	$C_{15}H_{19}N_3O_5S$
Formula weight	353.39
Temperature	300(2) K
Wavelength	0.71073 A
Crystal system, space group	Triclinic, P–1
Unit cell dimensions<br>a<br>b<br>c<br>α<br>β<br>γ	 7.7887(5) A<br>10.6013(6) A<br>10.9124(7) A<br>76.821(2) °<br>76.366(2) °<br>89.739(2) °
Volume	851.44(9) A^3
Z, Calculated density	2, 1.378 Mg/m^3
Absorption coefficient	0.220 mm^{-1}
F(000)	372
Crystal size	0.430 × 0.340 × 0.290 mm
Θ range for data collection	2.695 to 37.778 °
Limiting indices	$-13 \leq h \leq 13$, $-18 \leq k \leq 18$, $-18 \leq l \leq 18$
Reflections collected / unique	62800 / 9138 [R_{int} = 0.0842]
Completeness to θ	25.242 99.8%
Absorption correction	Semi-empirical from equivalents
Max. and min. transmission	0.7487 and 0.4247
Refinement method	Full-matrix least-squares on F^2
Data / restraints / parameters	9138 / 1 / 221
Goodness-of-fit on F^2	1.011
Final R indices [I>2σ(I)]	R_1 = 0.0640, wR_2 = 0.1673
R indices (all data)	R_1 = 0.1302, wR_2 = 0.2067
Extinction coefficient	N/A
Largest diff. peak and hole	0.367 and −0.340 e.$Å^{-3}$

Crystallographic data for **56** can be obtained free of charge from the Cambridge Crystallographic Data Centre (CCDC) via www.ccdc.cam.ac.uk/data_request/cif under CCDC deposition number CCDC 2537283.

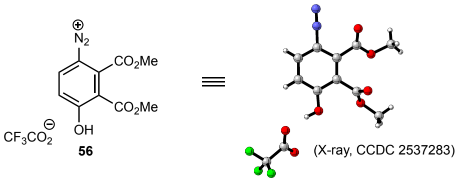

Crystal data and structure refinement for **56**.

Identification code	j1_a
Empirical formula	$C_{12}H_9F_3N_2O_7$
Formula weight	350.21
Temperature	298(2) K
Wavelength	0.71073 A
Crystal system, space group	Triclinic, P–1
Unit cell dimensions<br>a<br>b<br>c<br>α<br>β<br>γ	 8.5251(4) A<br>9.1919(4) A<br>11.0018(5) A<br>73.319(2) °<br>85.412(2) °<br>62.824°
Volume	733.35(6) A^3
Z, Calculated density	2, 1.586 Mg/m^3
Absorption coefficient	0.154 mm^{-1}
F(000)	404
Crystal size	0.450 × 0.340 × 0.090 mm
Θ range for data collection	2.643 to 29.996 °
Limiting indices	$-13 \le h \le 13$, $-14 \le k \le 14$, $-17 \le l \le 17$
Reflections collected / unique	32257 / 6149 [R_{int} = 0.0823]
Completeness to θ	25.242 99.8%
Absorption correction	Semi-empirical from equivalents
Max. and min. transmission	0.7470 and 0.4647
Refinement method	Full-matrix least-squares on F^2
Data / restraints / parameters	6149 / 42 / 249
Goodness-of-fit on F^2	1.017
Final R indices [I>2σ(I)]	R_1 = 0.0684, wR_2 = 0.1753
R indices (all data)	R_1 = 0.1194, wR_2 = 0.2067
Extinction coefficient	N/A
Largest diff. peak and hole	0.376 and −0.258 e.$Å^{-3}$

Crystallographic data for **53** can be obtained free of charge from the Cambridge Crystallographic Data Centre (CCDC) via www.ccdc.cam.ac.uk/data_request/cif under CCDC deposition number CCDC 2537281.

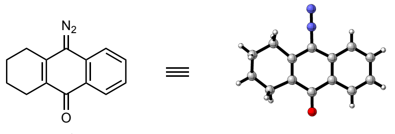

Crystal data and structure refinement for **53**.

Identification code	j2_a
Empirical formula	$C_{14}H_{12}N_2O$
Formula weight	224.26
Temperature	299(2) K
Wavelength	0.71073 A
Crystal system, space group	Monoclinic, P2(1)/c
Unit cell dimensions<br>a<br>b<br>c<br>α<br>β<br>γ	 9.0123(6) A<br>17.7345(11) A<br>7.1065(5) A<br>90 °<br>101.741(3) °<br>90 °
Volume	1112.06(13) A^3
Z, Calculated density	4, 1.339 Mg/m^3
Absorption coefficient	0.086 mm^{-1}
F(000)	472
Crystal size	0.350 × 0.250 × 0.140 mm
Θ range for data collection	3.145 to 25.349 °
Limiting indices	$-10 \le h \le 10$, $-21 \le k \le 21$, $-8 \le l \le 8$
Reflections collected / unique	20556 / 2032 [R_{int} = 0.0929]
Completeness to θ	25.242 99.6%
Absorption correction	Semi-empirical from equivalents
Max. and min. transmission	0.7454 and 0.4404
Refinement method	Full-matrix least-squares on F^2
Data / restraints / parameters	2032 / 123 / 191
Goodness-of-fit on F^2	1.073
Final R indices [I>2σ(I)]	R_1 = 0.1031, wR_2 = 0.2807
R indices (all data)	R_1 = 0.1543, wR_2 = 0.3238
Extinction coefficient	N/A
Largest diff. peak and hole	0.232 and −0.173 e.$Å^{-3}$

NMR Spectra

Azides

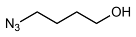

azide-01
^{1}H NMR
$CDCl_3$, 900 MHz

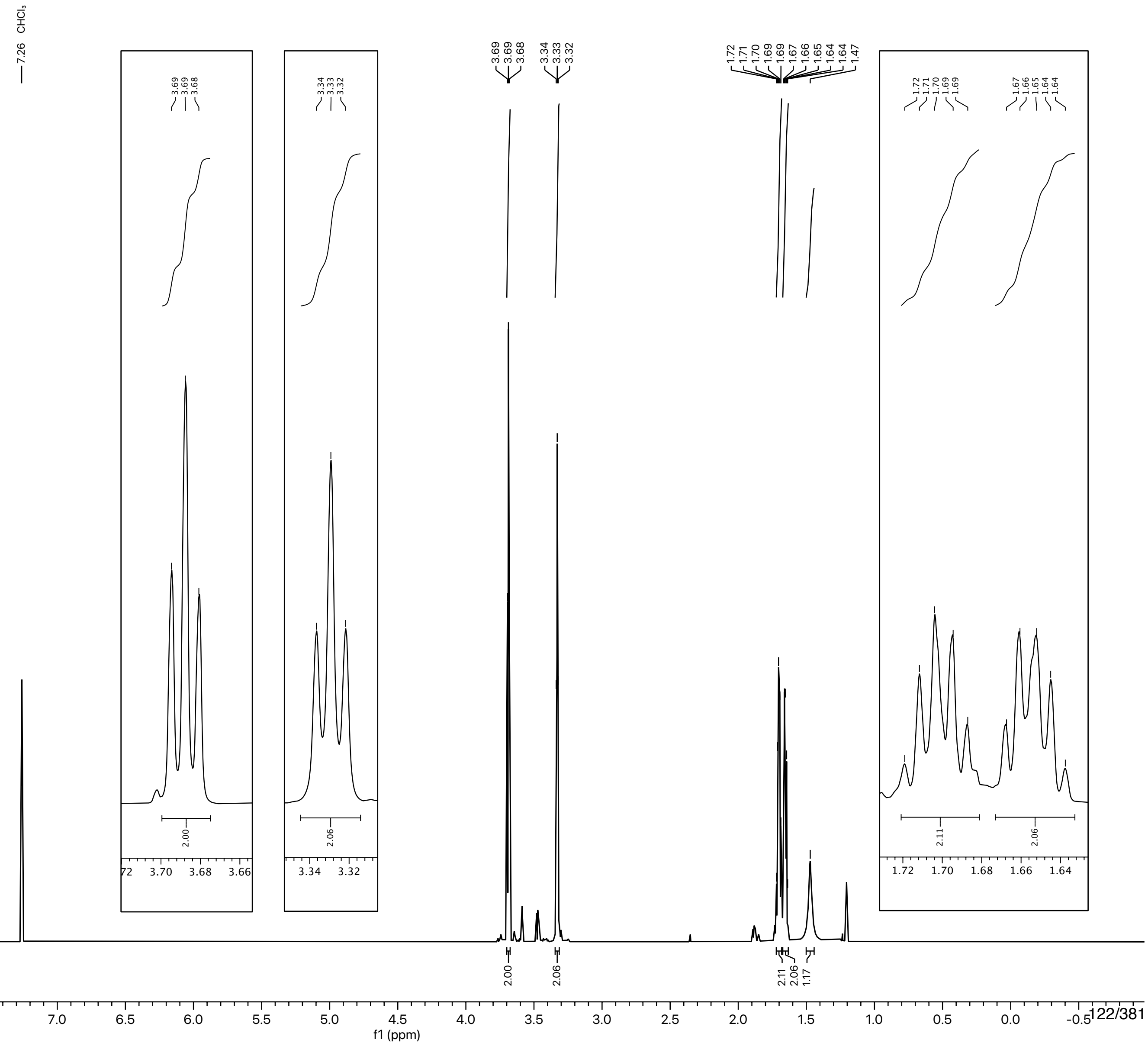

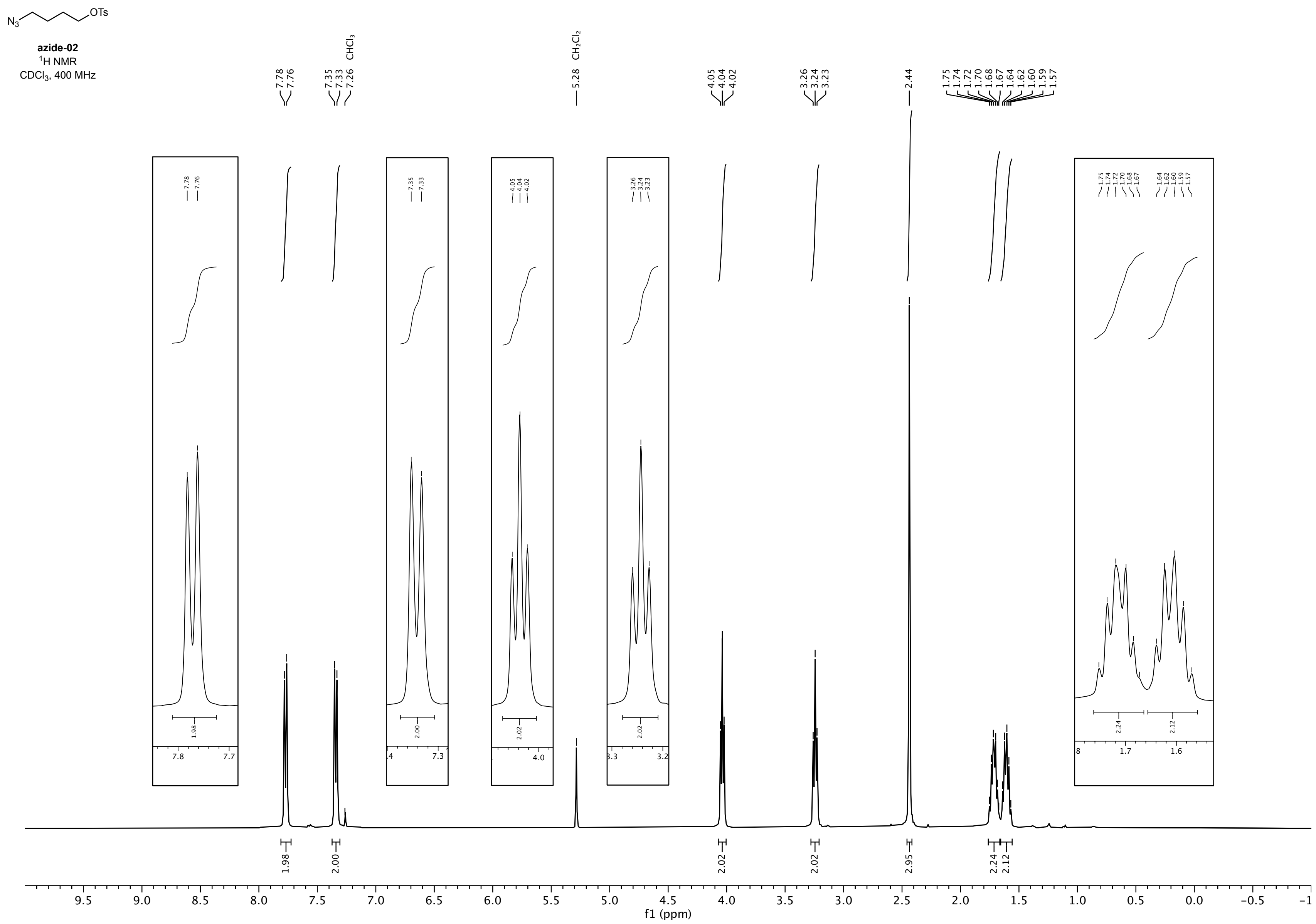
N3
OTs
azide-02
1H NMR
CDCl3, 400 MHz
7.78
7.76
7.35
7.33
7.26
CHCl3
5.28
CH2Cl2
4.05
4.04
4.02
3.26
3.24
3.23
2.44
1.75
1.74
1.72
1.70
1.68
1.67
1.64
1.62
1.60
1.59
1.57
1.98
2.00
2.02
2.02
2.95
2.24
2.12
f1 (ppm)

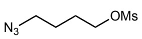

azide-03
^{1}H NMR
$CDCl_3$, 400 MHz

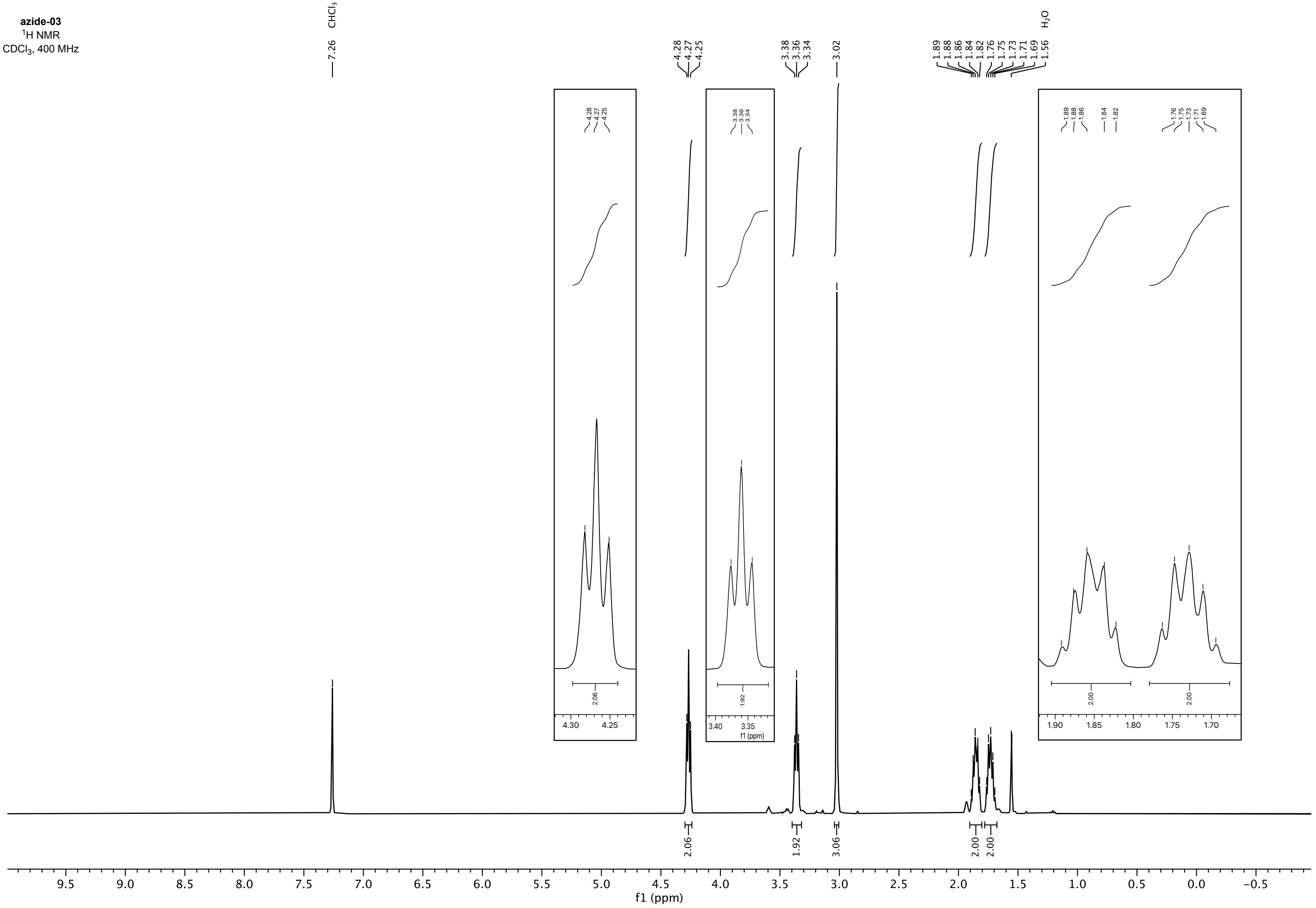

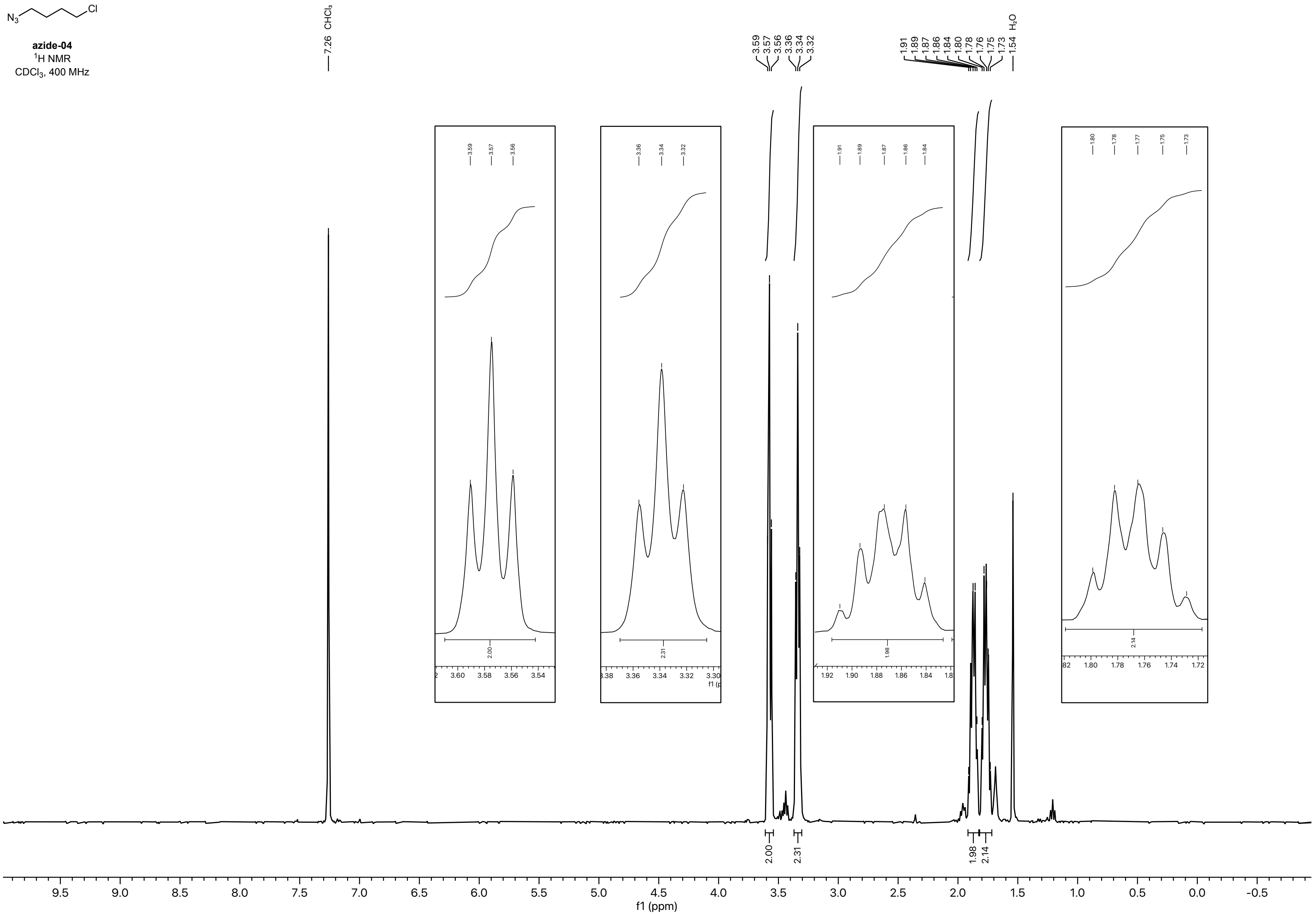

azide-04
^1H NMR
$CDCl_3$, 400 MHz
7.26 $CHCl_3$
3.59 3.57 3.56 3.36 3.34 3.32
1.91 1.89 1.87 1.86 1.84 1.80 1.78 1.76 1.75 1.73
1.54 H_2O
2.00 2.31 1.98 2.14
f1 (ppm)

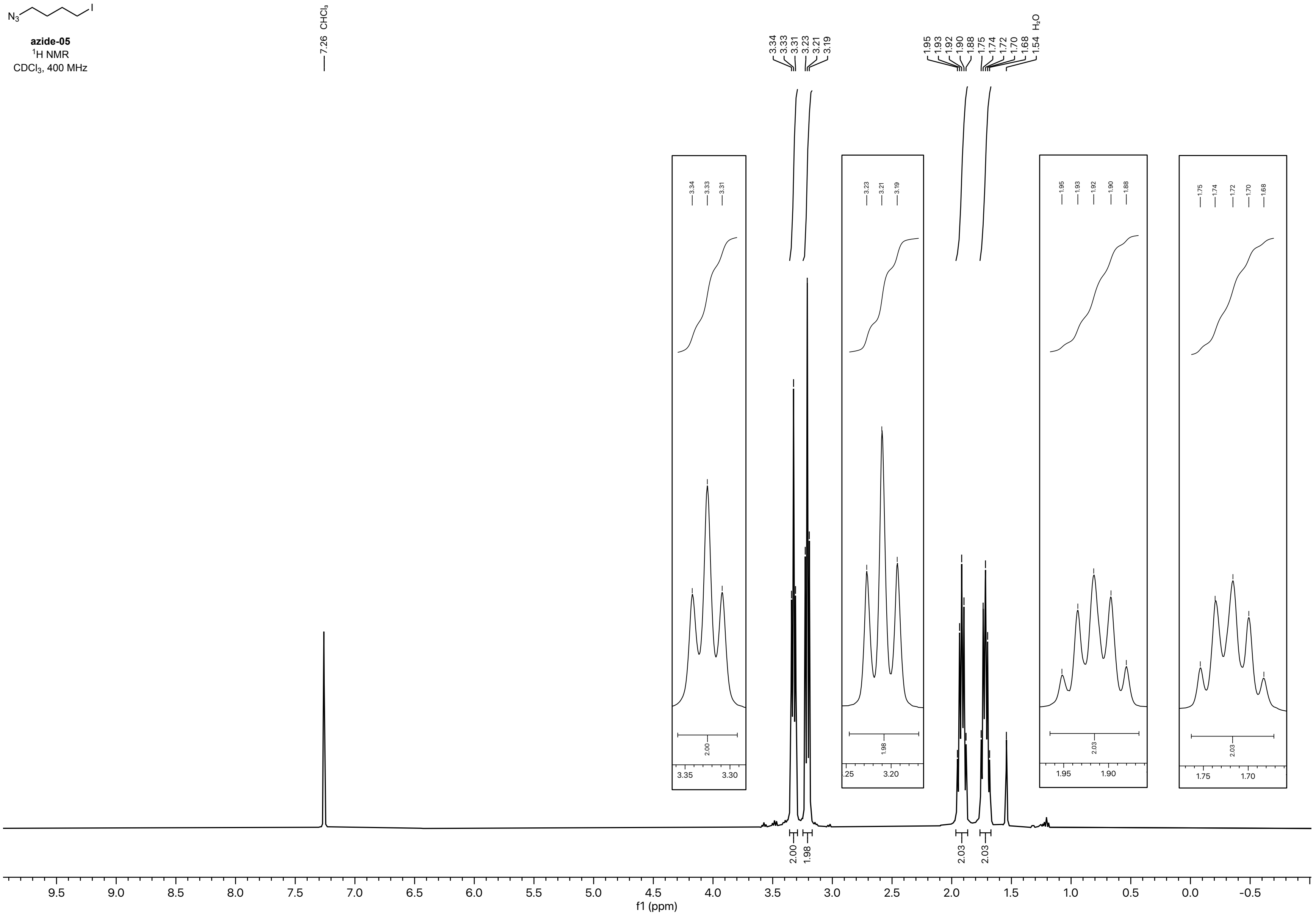
azide-05
^{1}H NMR
$CDCl_3$, 400 MHz
7.26 $CHCl_3$
3.34
3.33
3.31
3.23
3.21
3.19
1.95
1.93
1.92
1.90
1.88
1.75
1.74
1.72
1.70
1.68
1.54 H_2O
2.00
1.98
2.03
2.03
f1 (ppm)

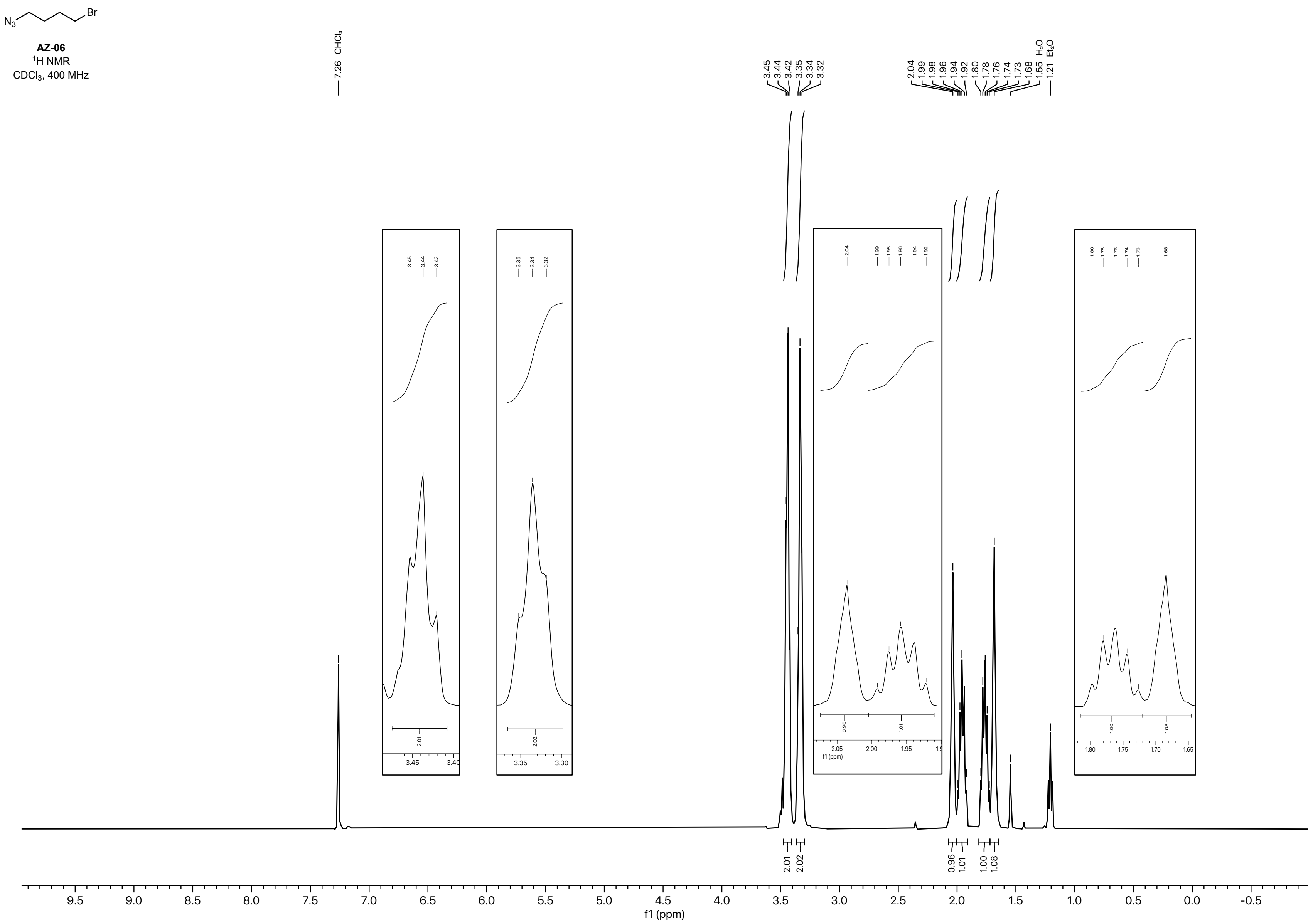

N_3
Br
AZ-06
^{1}H NMR
$CDCl_3$, 400 MHz
7.26 $CHCl_3$
3.45
3.44
3.42
3.35
3.34
3.32
2.04
1.99
1.98
1.96
1.94
1.92
1.80
1.78
1.76
1.74
1.73
1.68
1.55 H_2O
1.21 Et_2O
2.01
2.02
0.96
1.01
1.00
1.08
9.5 9.0 8.5 8.0 7.5 7.0 6.5 6.0 5.5 5.0 4.5 4.0 3.5 3.0 2.5 2.0 1.5 1.0 0.5 0.0 -0.5
f1 (ppm)

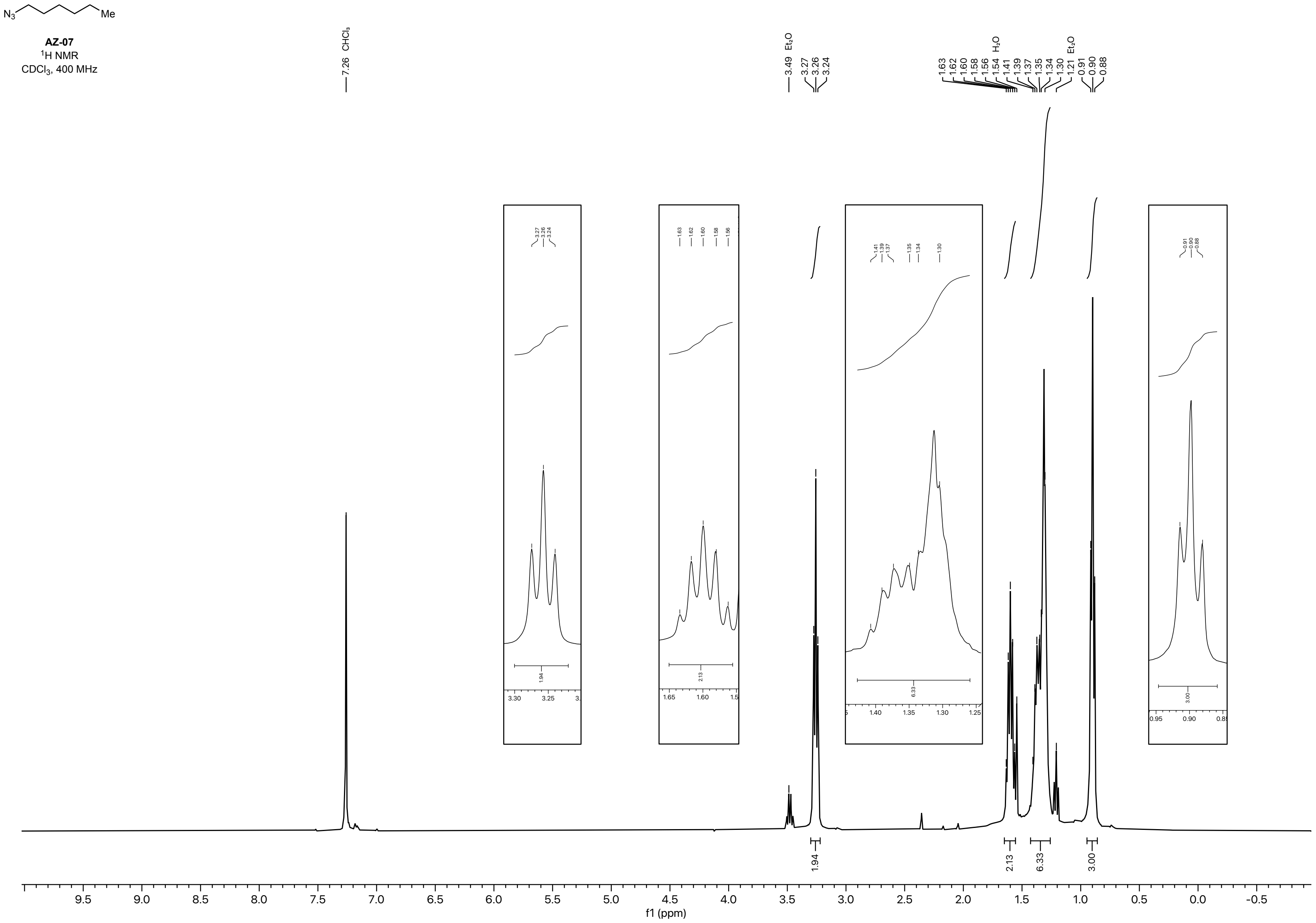

N3
Me
AZ-07
1H NMR
CDCl3, 400 MHz
7.26 CHCl3
3.49 Et2O
3.27
3.26
3.24
1.63
1.62
1.60
1.58
1.56
1.54 H2O
1.41
1.39
1.37
1.35
1.34
1.30
1.21 Et2O
0.91
0.90
0.88
1.94
2.13
6.33
3.00
f1 (ppm)

azide-08
^1H NMR
$CDCl_3$, 400 MHz

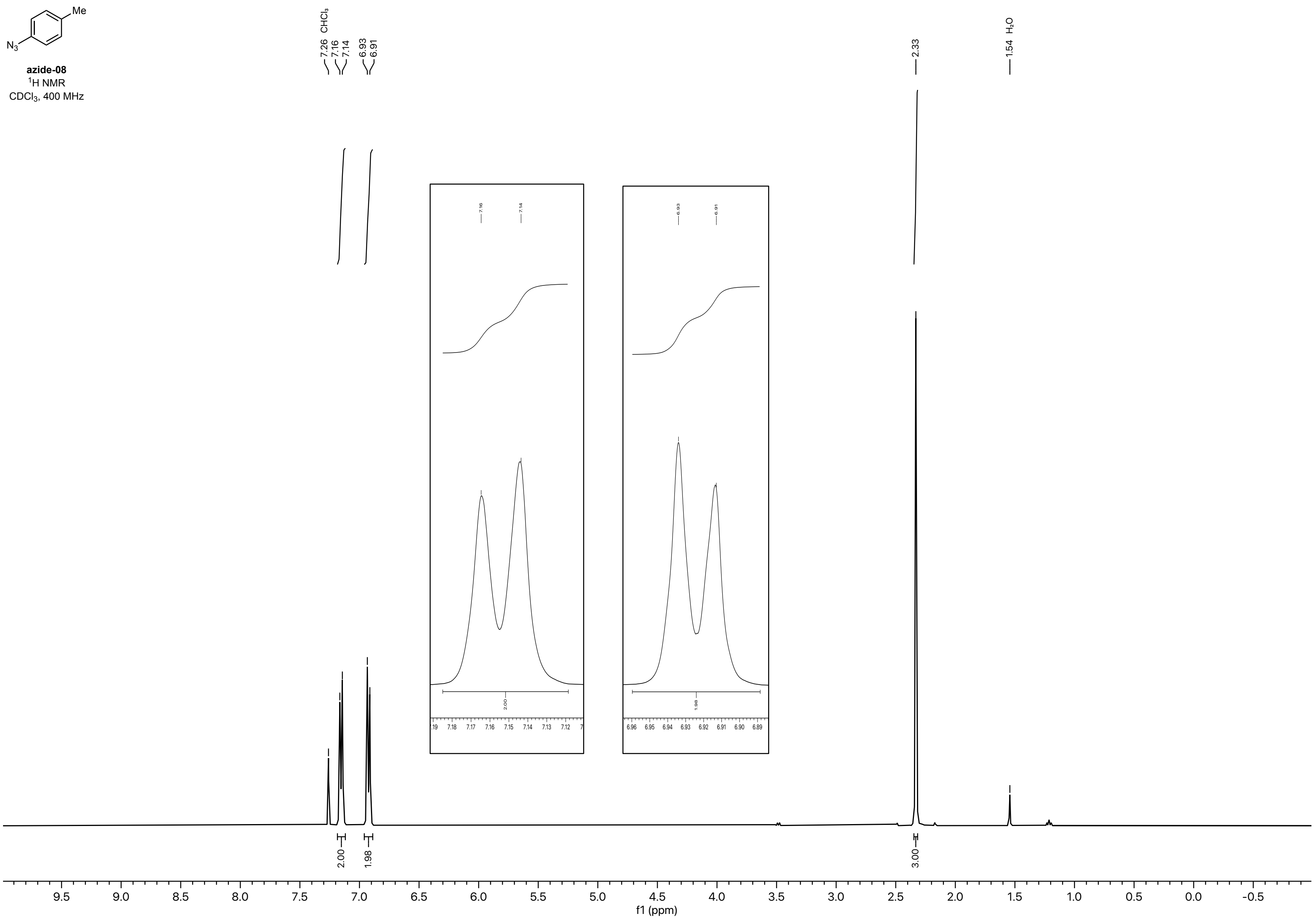

2-Triazenyl Furans

Scouting Experiments

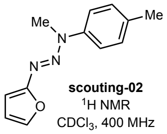

scouting-02
^{1}H NMR
$CDCl_3$, 400 MHz

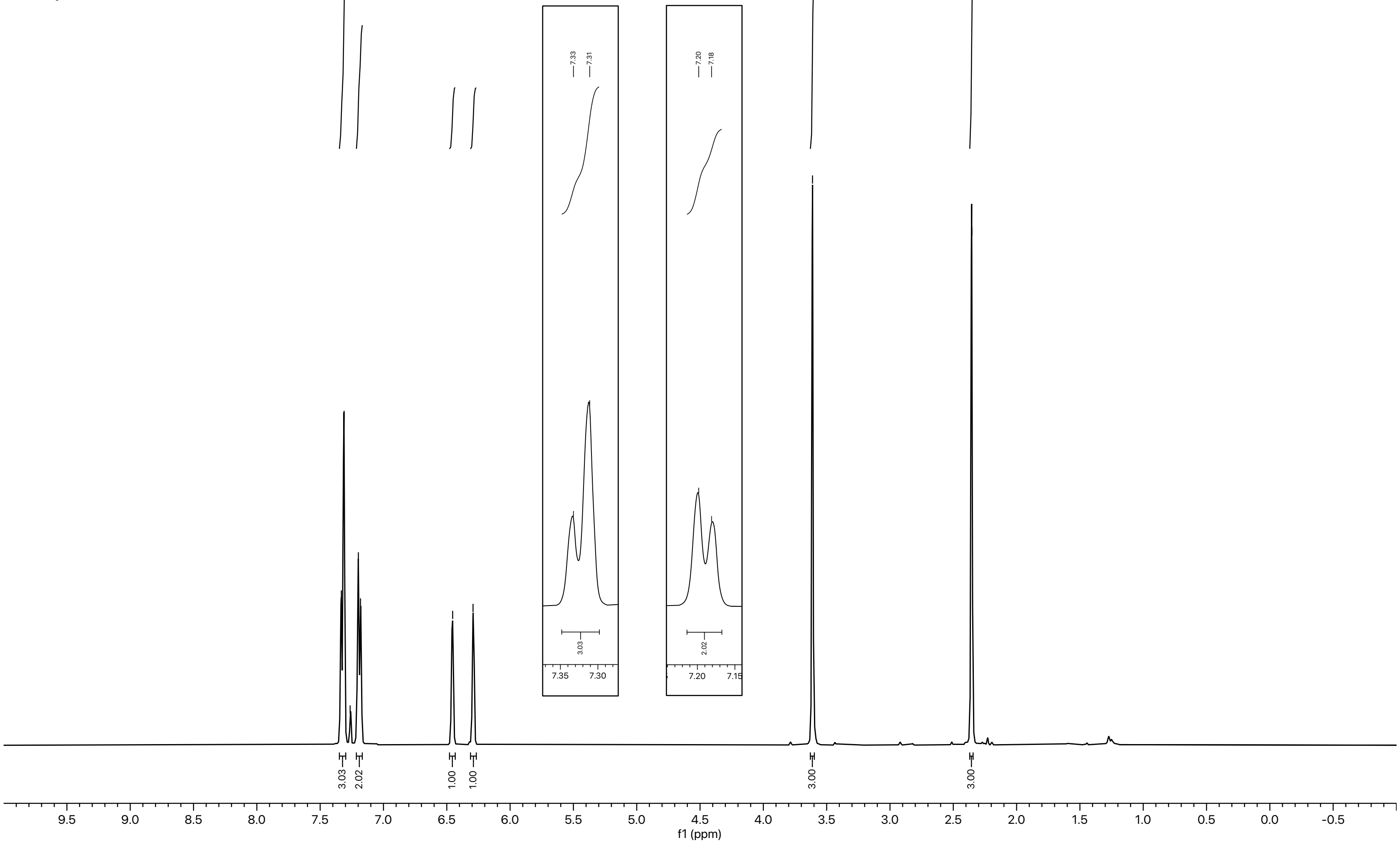

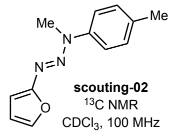

scouting-02
^{13}C NMR
$CDCl_3$, 100 MHz

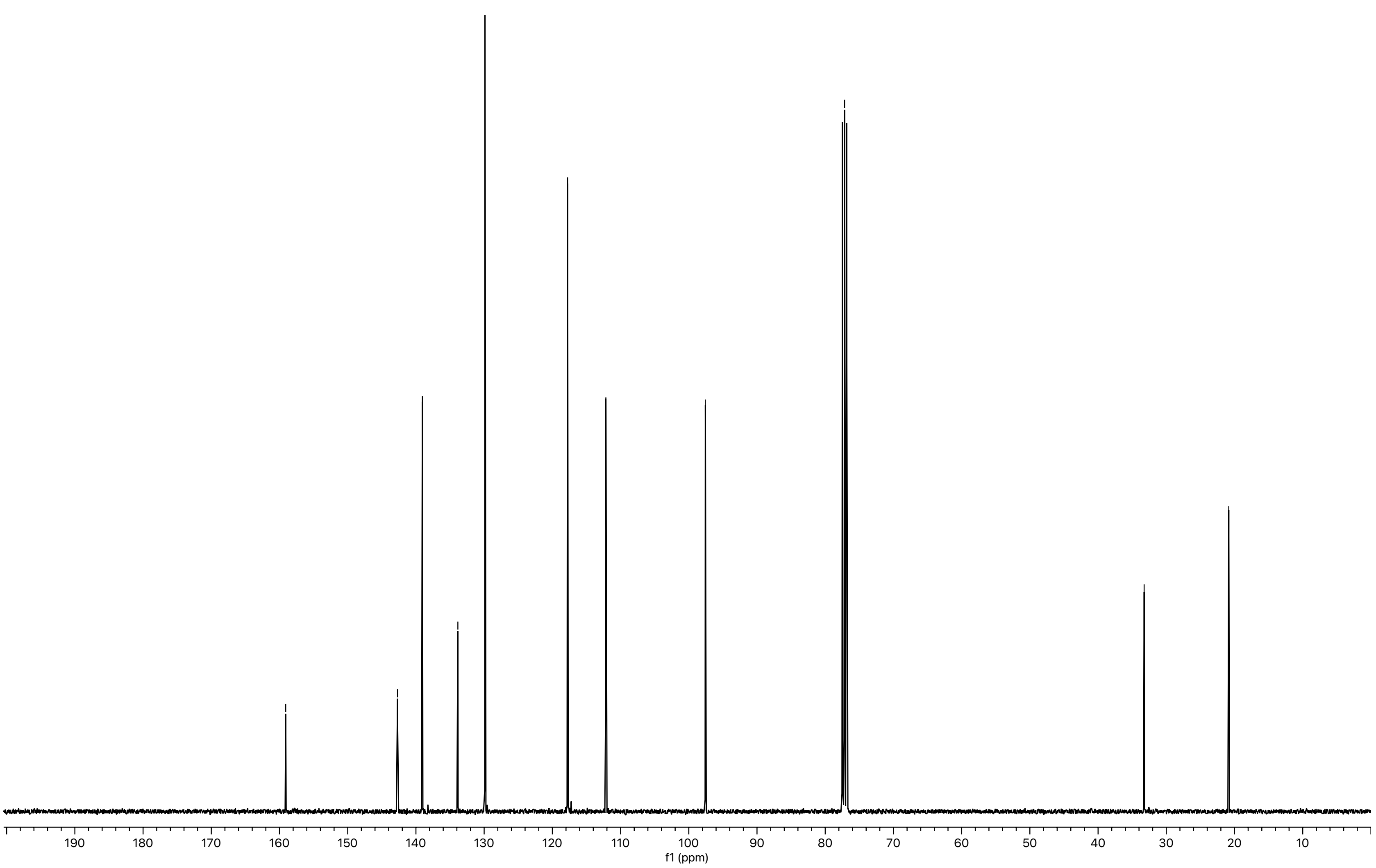

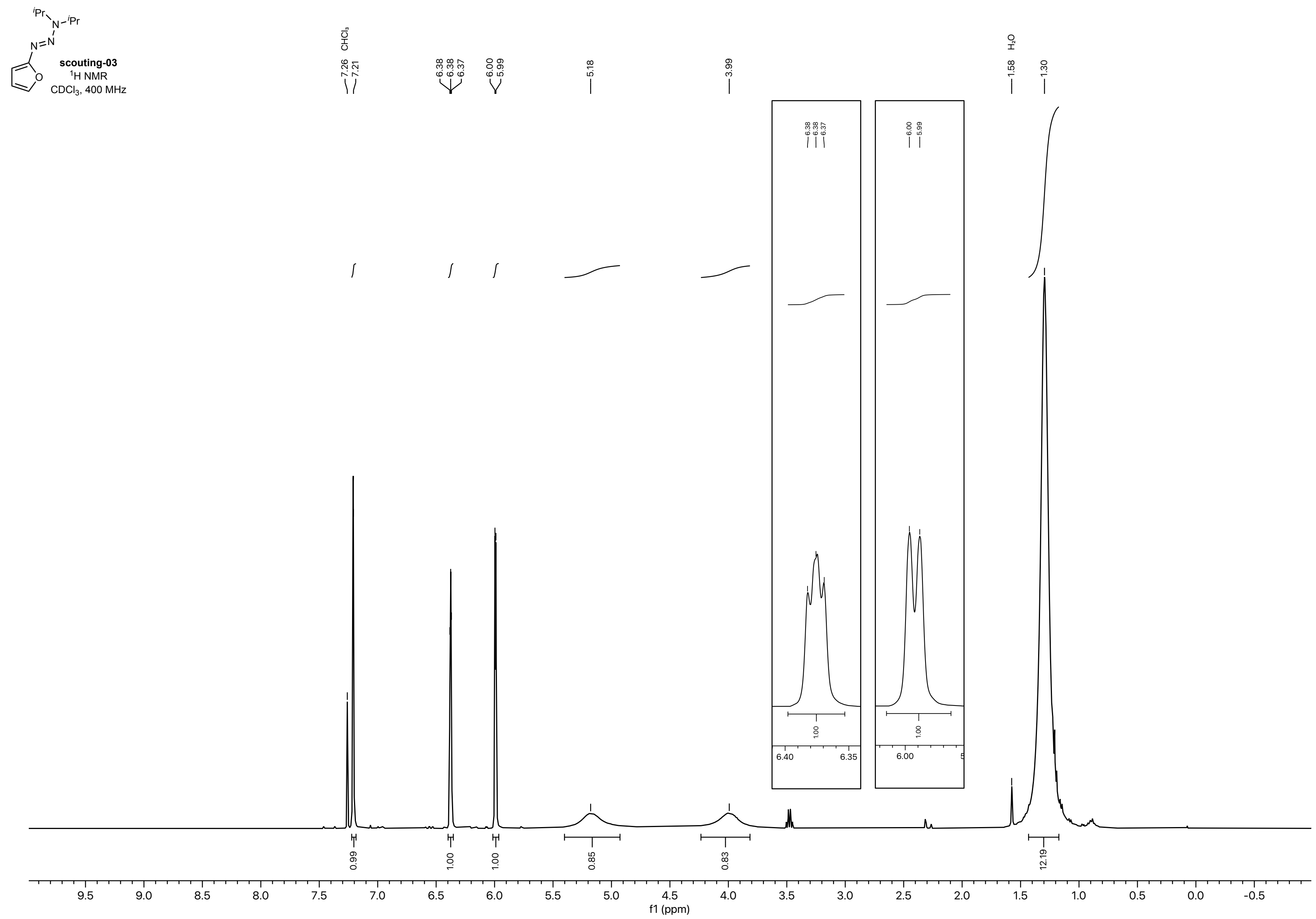
scouting-03
1H NMR
CDCl3, 400 MHz
7.26 CHCl3
7.21
6.38
6.38
6.37
6.00
5.99
5.18
3.99
1.58 H2O
1.30
0.99
1.00
1.00
0.85
0.83
12.19
f1 (ppm)

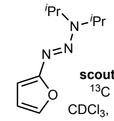

scouting-03
^{13}C NMR
$CDCl_3$, 100 MHz

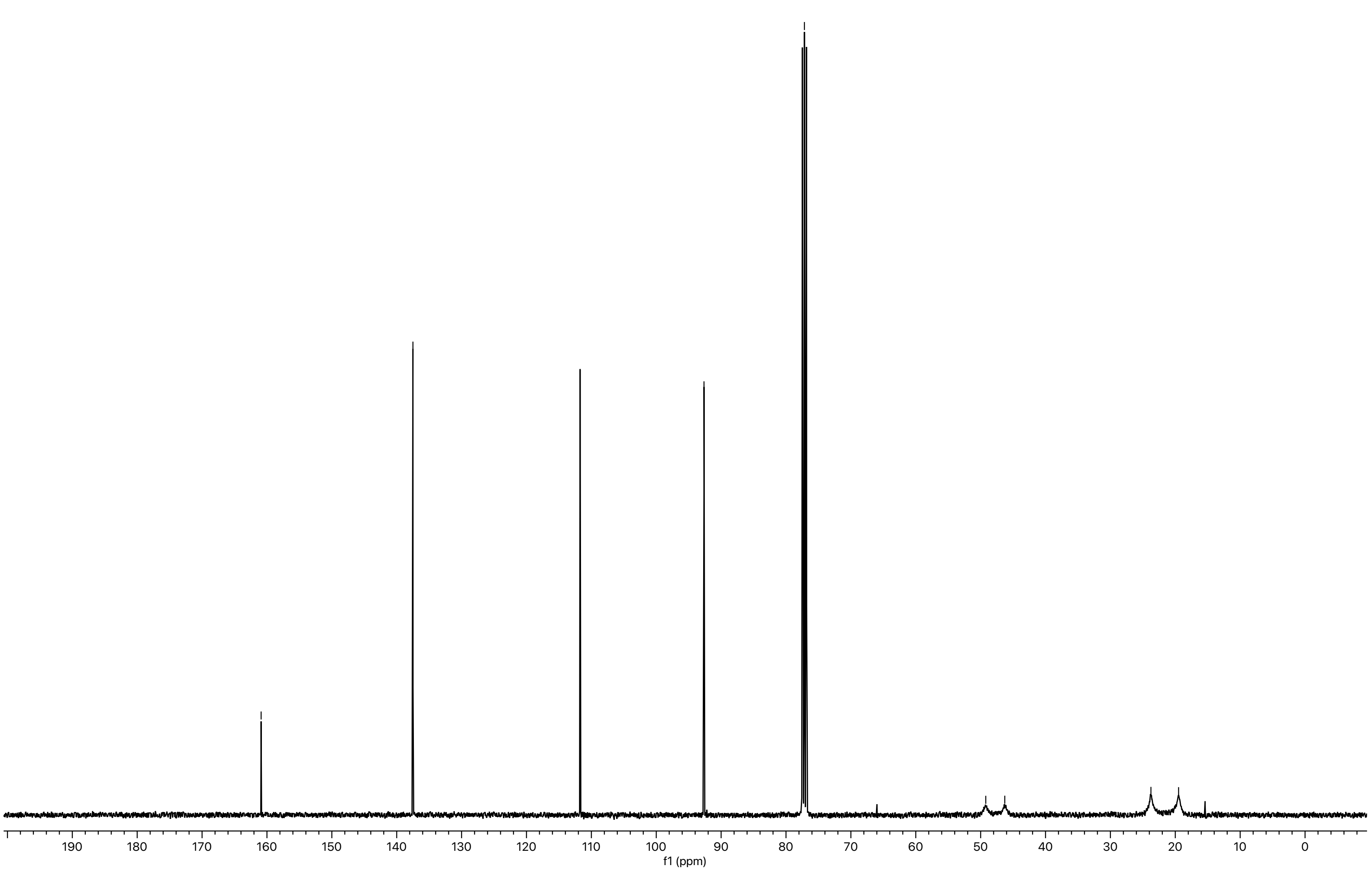

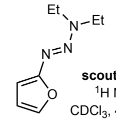

scouting-04
^{1}H NMR
$CDCl_3$, 400 MHz

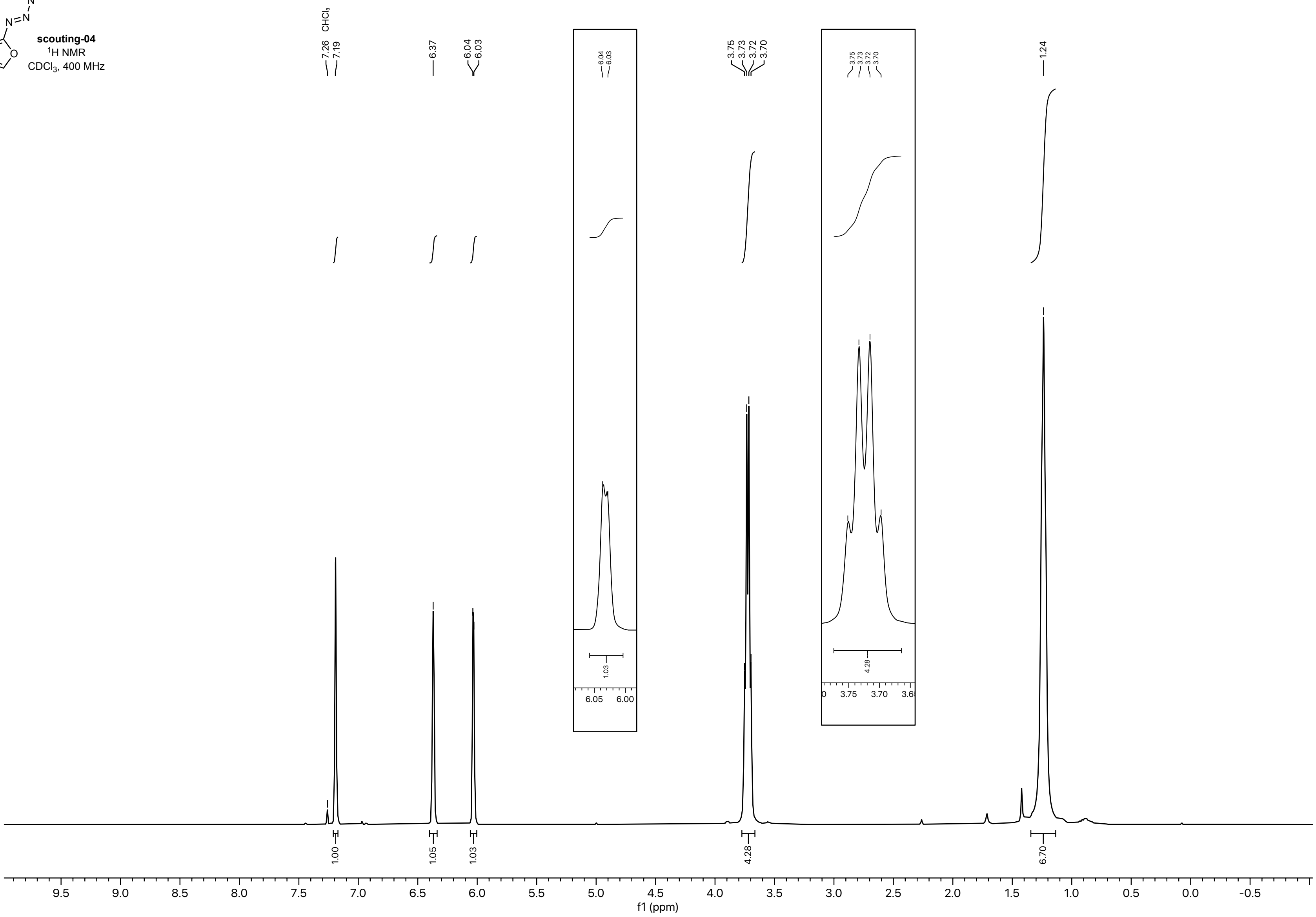

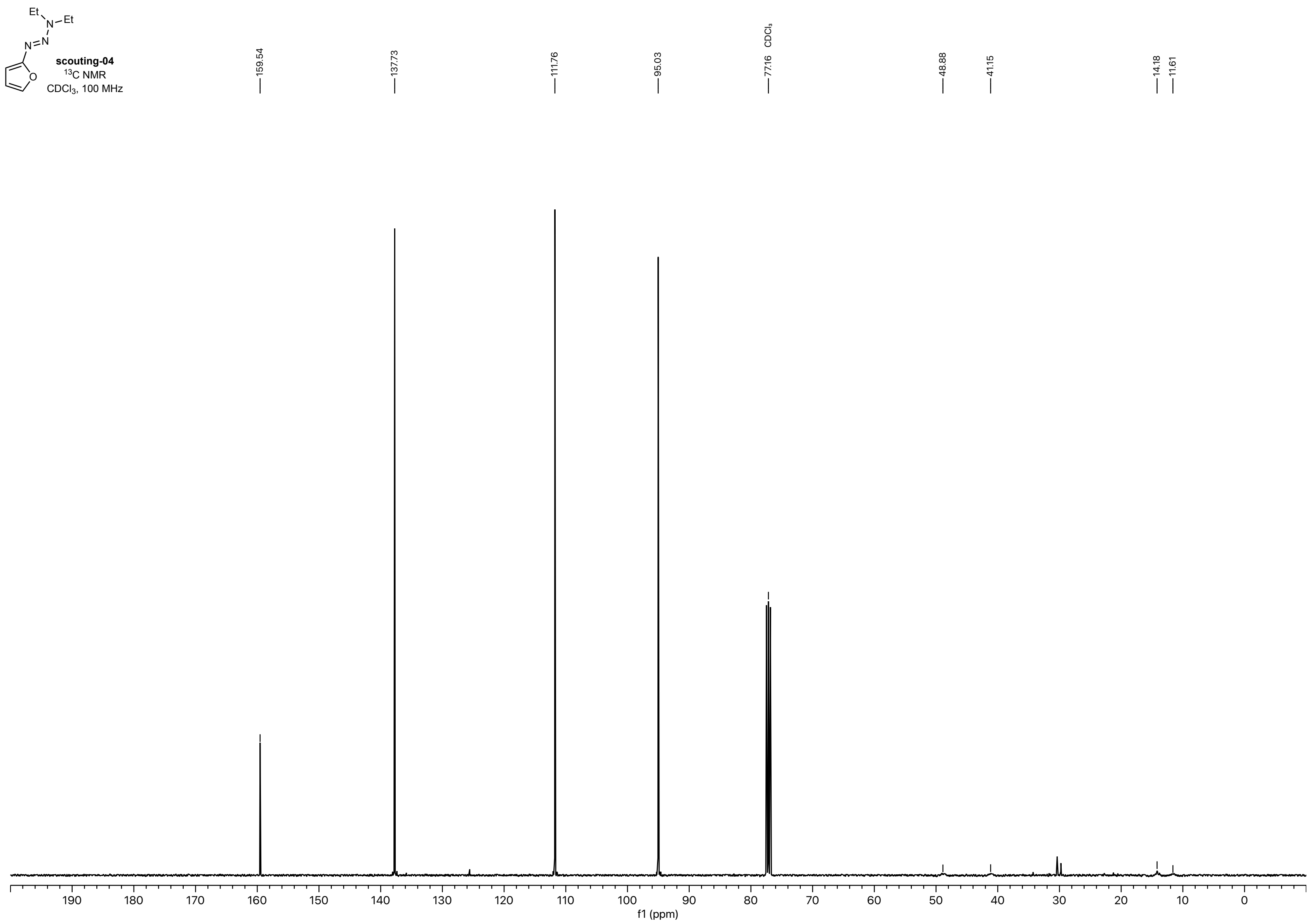
Et
N
Et
N
N
O
scouting-04
13C NMR
CDCl3, 100 MHz
159.54
137.73
111.76
95.03
77.16 CDCl3
48.88
41.15
14.18
11.61
190
180
170
160
150
140
130
120
110
100
90
80
70
60
50
40
30
20
10
0
f1 (ppm)

One-Pot Furan Triazenylation/Intramolecular Alkylation

Triazenylation Precursors

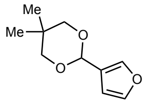

triazene-precursor-03
^1H NMR
$CDCl_3$, 400 MHz

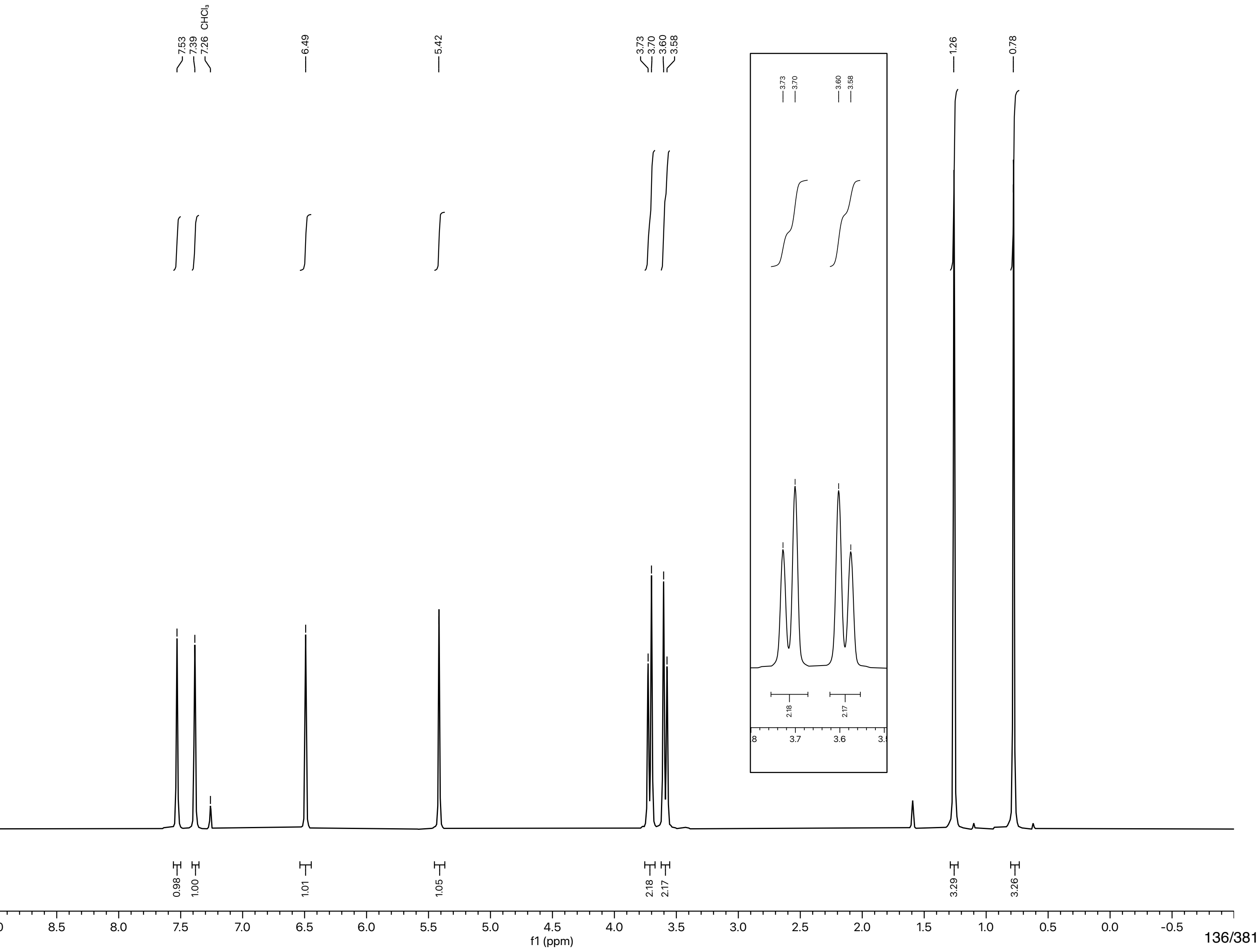

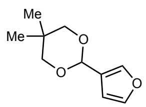

triazene-precursor-03
^{13}C NMR
$CDCl_3$, 100 MHz

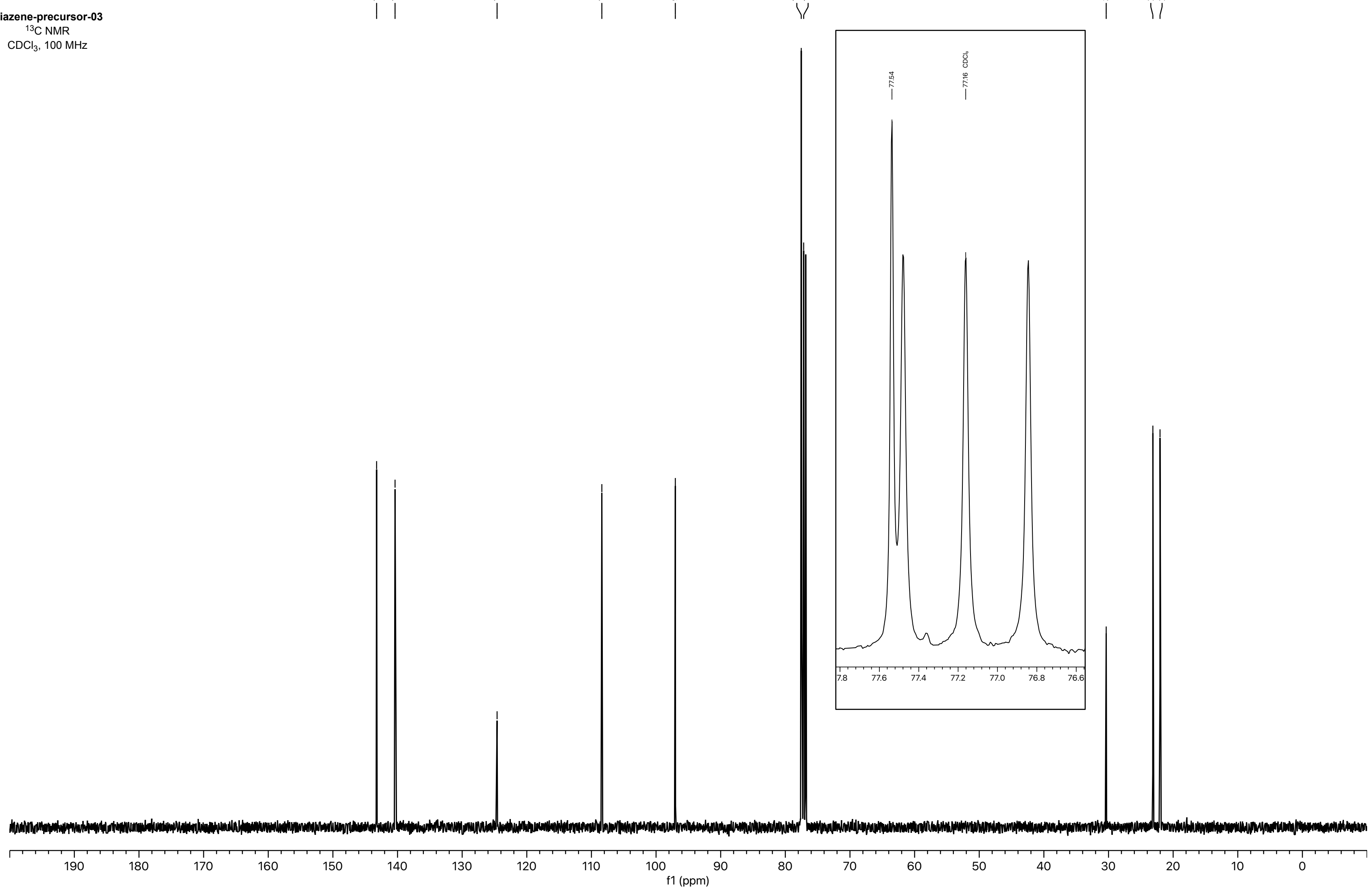

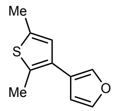

triazene-precursor-04
^1H NMR
$CDCl_3$, 400 MHz

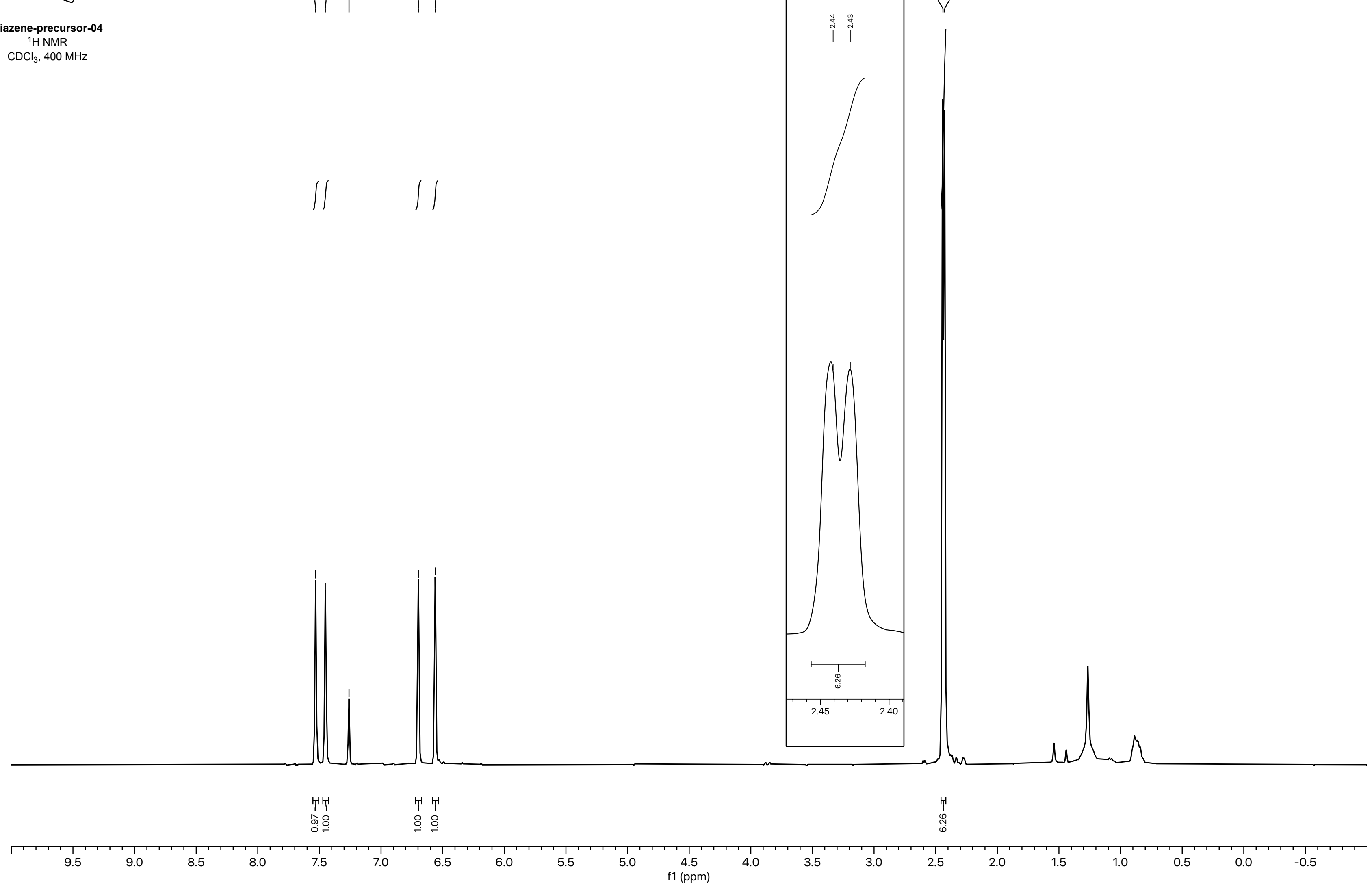

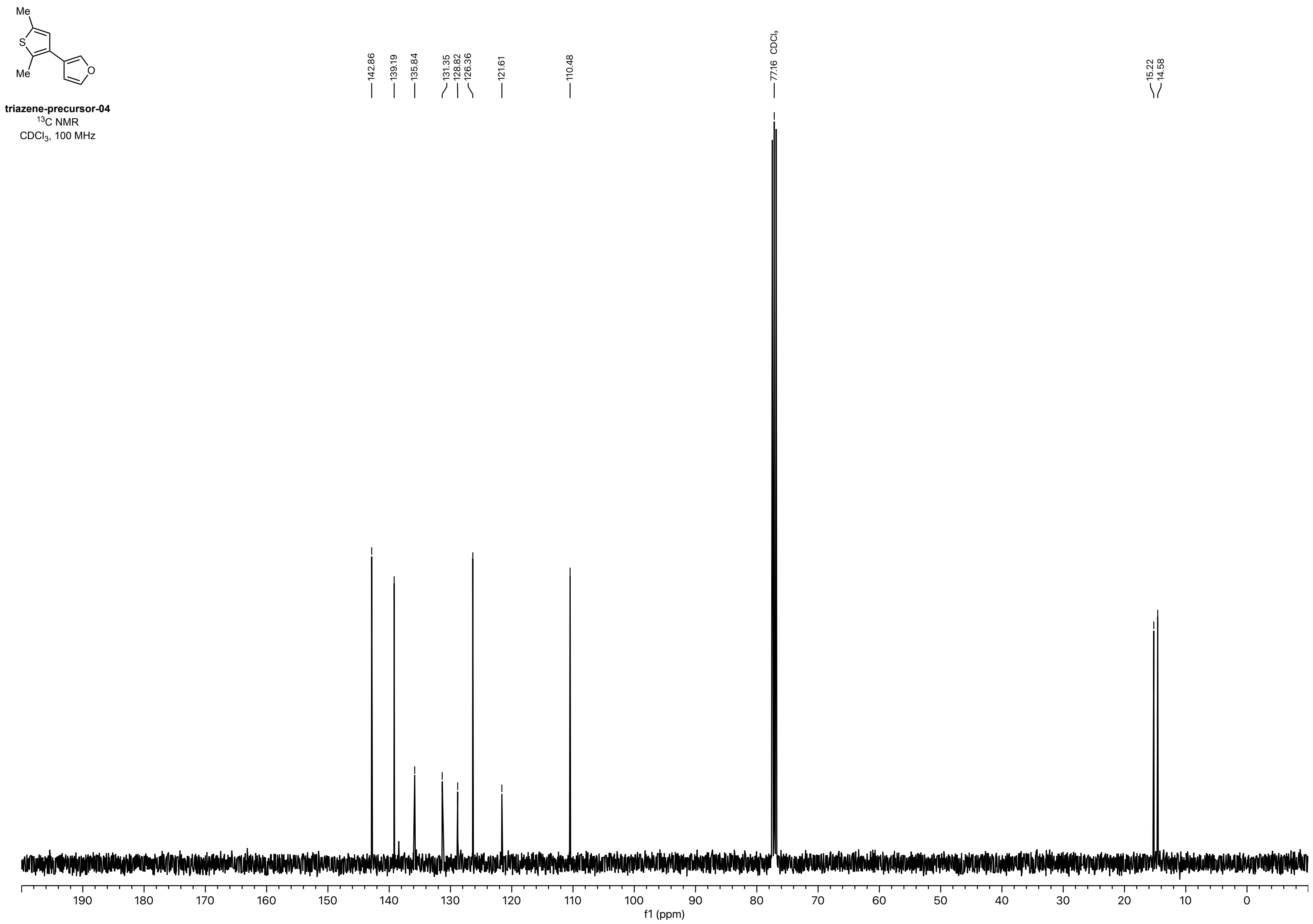

Me
S
Me
O
triazene-precursor-04
13C NMR
CDCl3, 100 MHz
142.86
139.19
135.84
131.35
128.82
126.36
121.61
110.48
77.16 CDCl3
15.22
14.58
190
180
170
160
150
140
130
120
110
100
90
80
70
60
50
40
30
20
10
0
f1 (ppm)

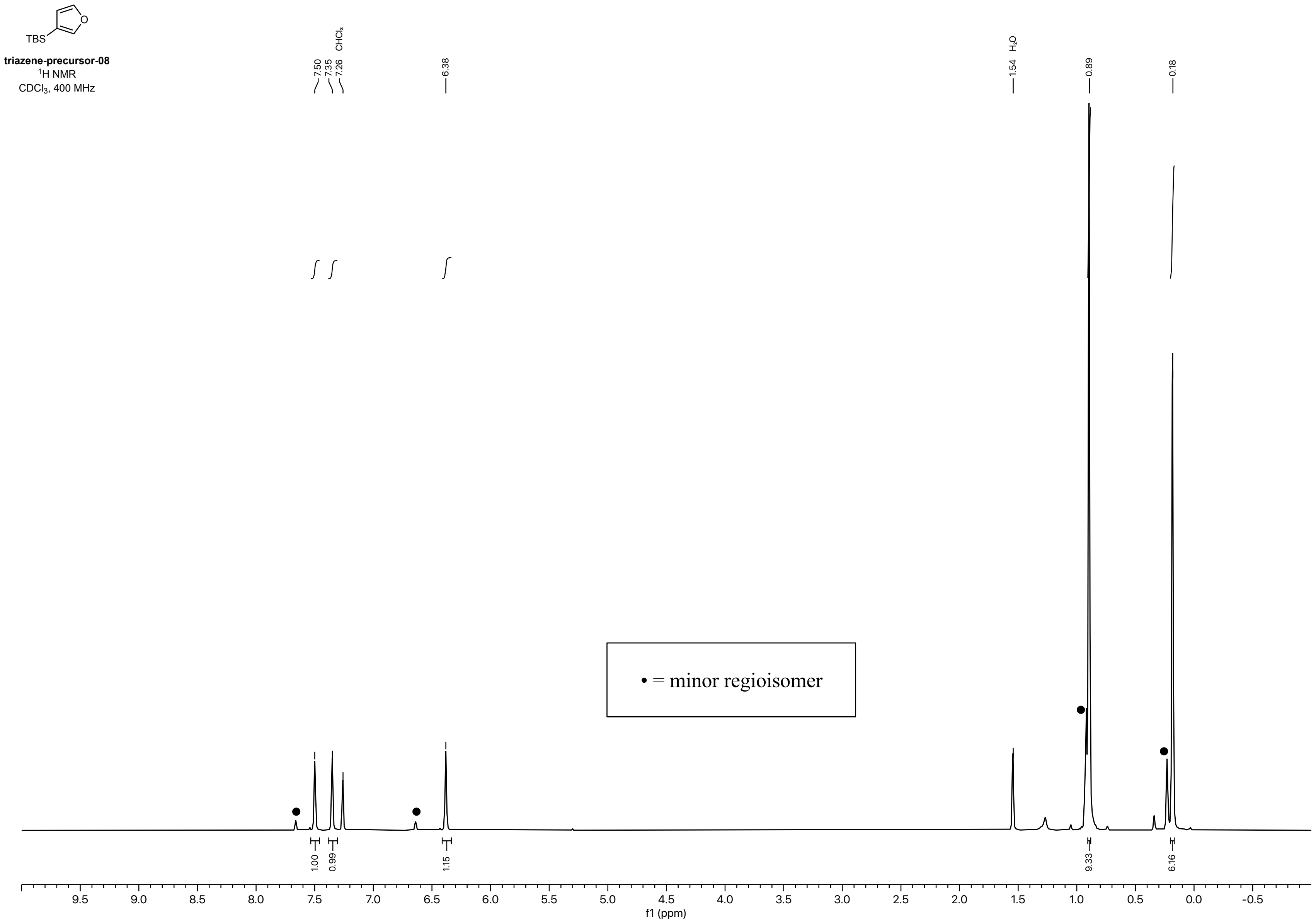

TBS
O
triazene-precursor-08
^{1}H NMR
$CDCl_3$, 400 MHz
7.50
7.35
7.26
$CHCl_3$
6.38
1.54
H_2O
0.89
0.18
• = minor regioisomer
1.00
0.99
1.15
9.33
6.16
9.5
9.0
8.5
8.0
7.5
7.0
6.5
6.0
5.5
5.0
4.5
4.0
3.5
3.0
2.5
2.0
1.5
1.0
0.5
0.0
-0.5
f1 (ppm)

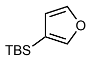

triazene-precursor-08
^{13}C NMR
$CDCl_3$, 150 MHz

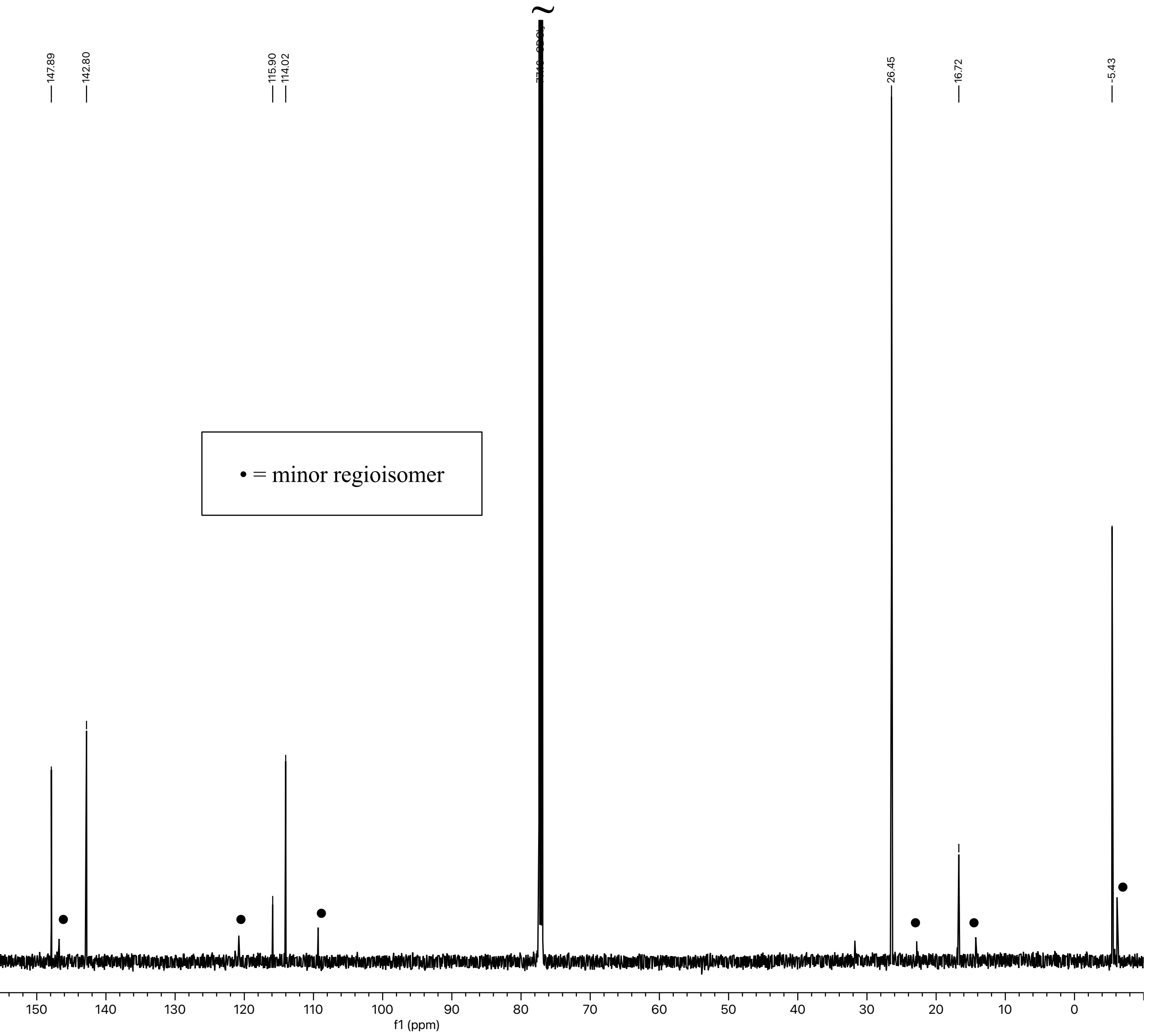

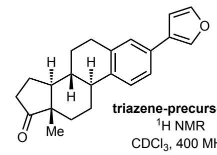

triazene-precursor-11
^1H NMR
$CDCl_3$, 400 MHz

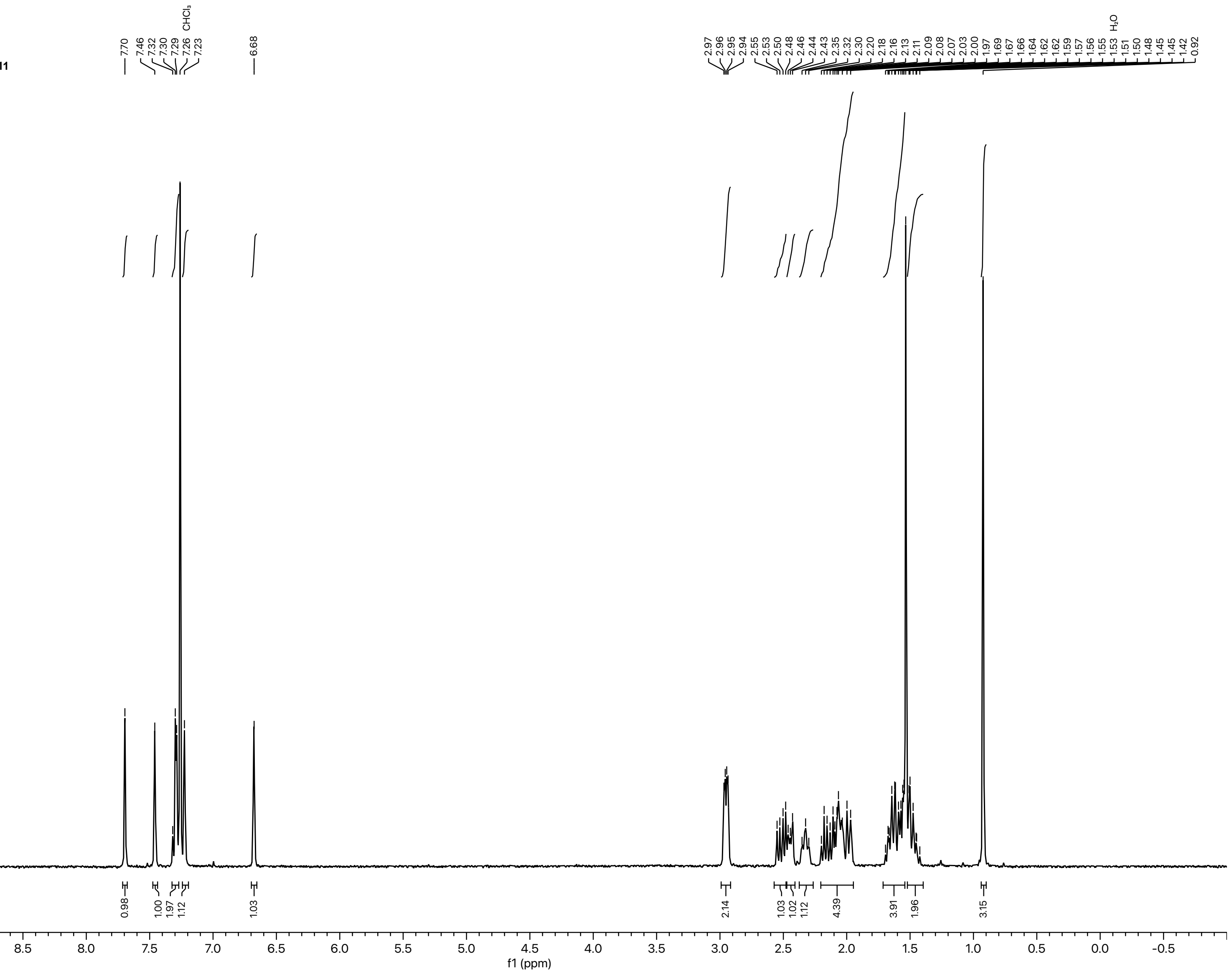

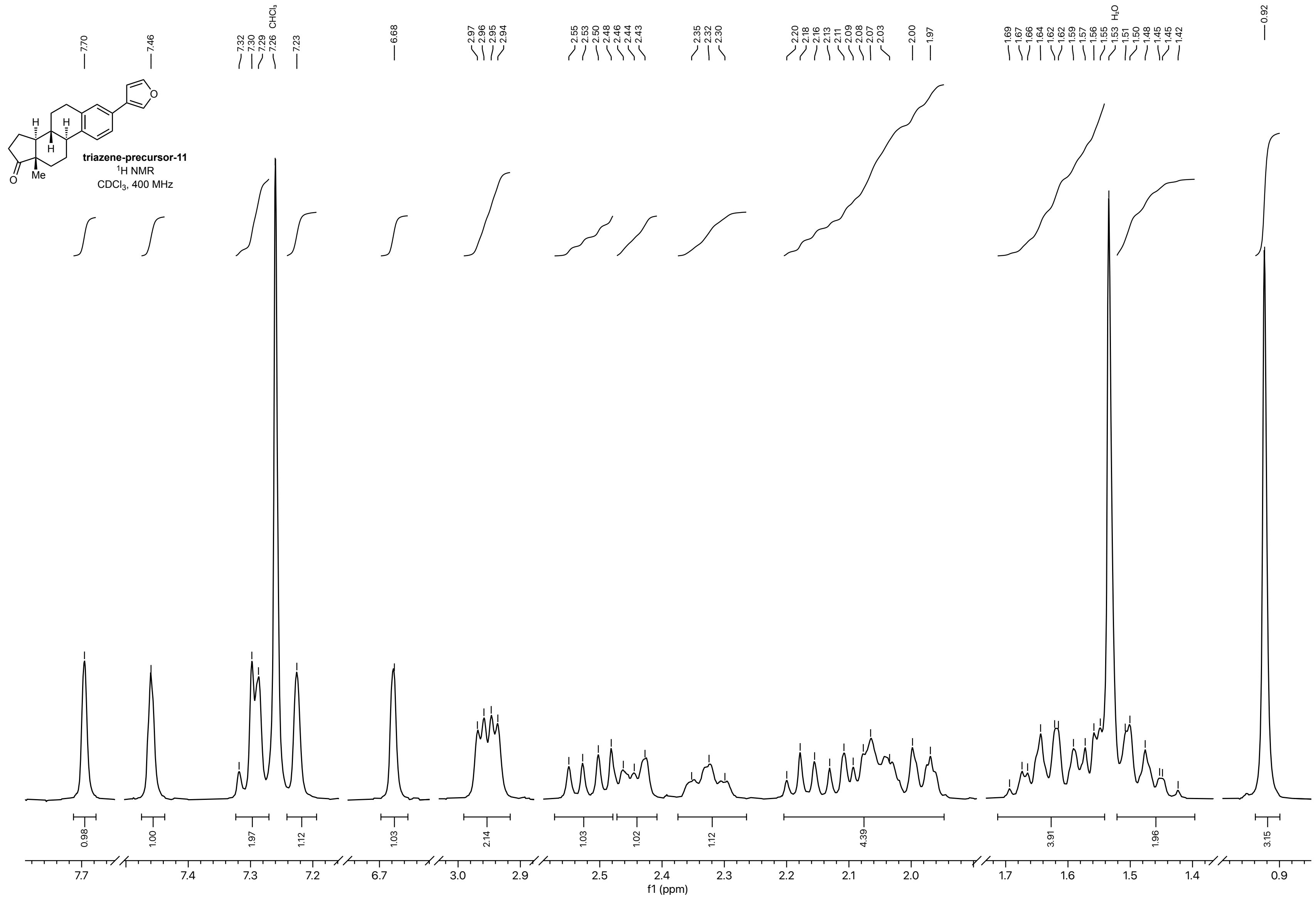
triazene-precursor-11
^{1}H NMR
$CDCl_3$, 400 MHz
f1 (ppm)

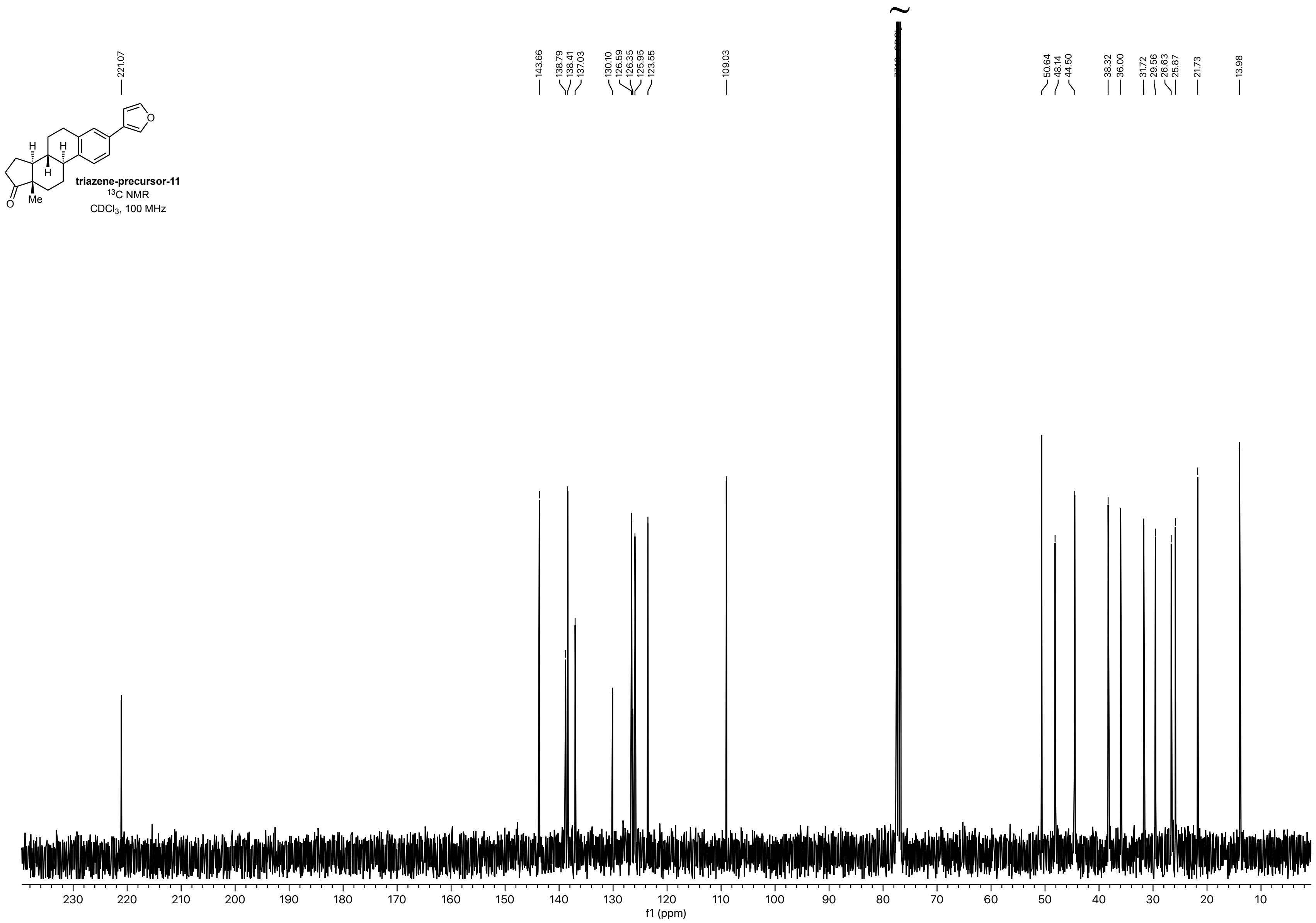

221.07
143.66
138.79
138.41
137.03
130.10
126.59
126.35
125.95
123.55
109.03
50.64
48.14
44.50
38.32
36.00
31.72
29.56
26.63
25.87
21.73
13.98
O
H
H
H
Me
O
triazene-precursor-11
^{13}C NMR
$CDCl_3$, 100 MHz
230 220 210 200 190 180 170 160 150 140 130 120 110 100 90 80 70 60 50 40 30 20 10
f1 (ppm)

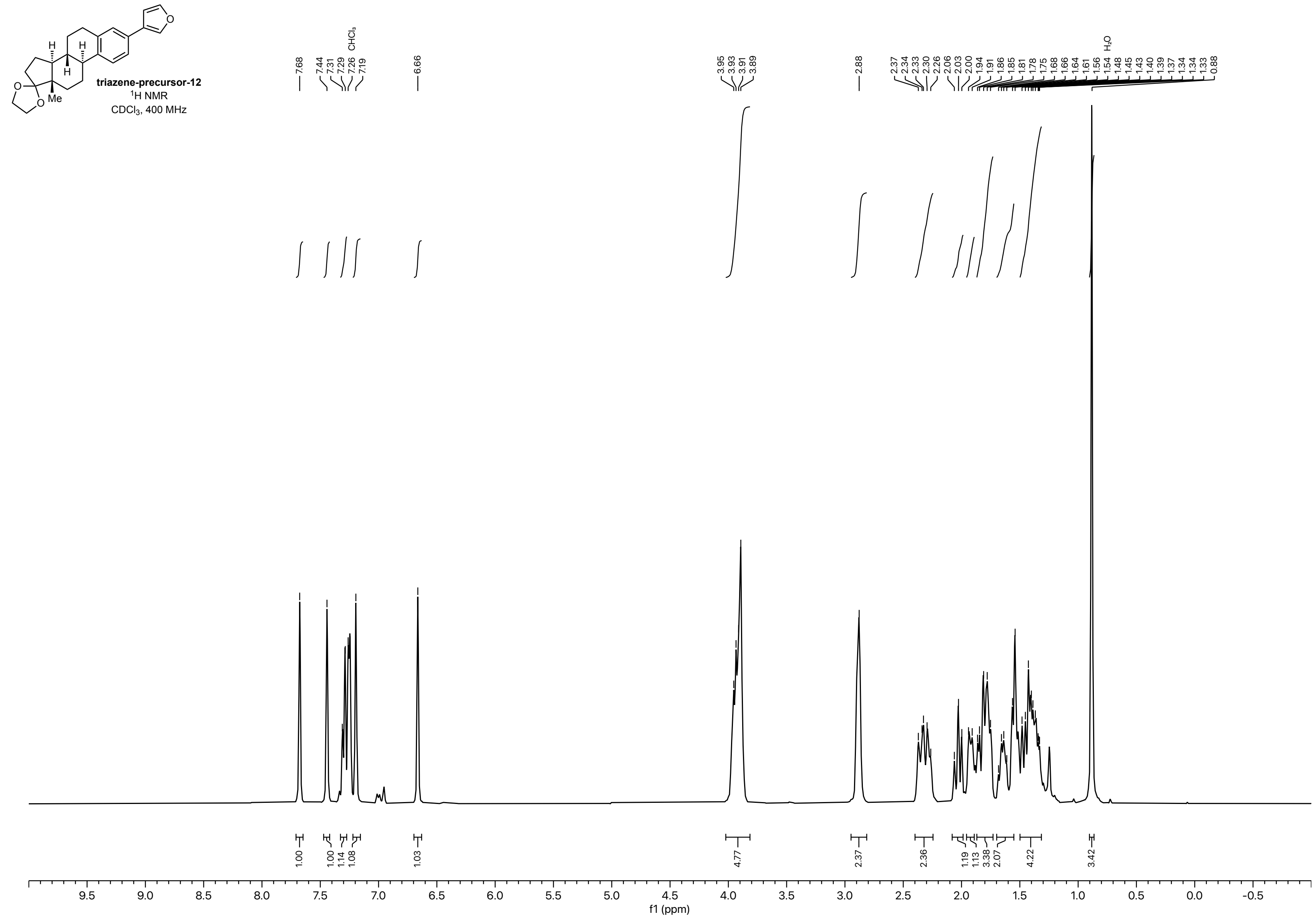
triazene-precursor-12
^{1}H NMR
CDCl$_3$, 400 MHz
7.68
7.44
7.31
7.29
7.26 CHCl$_3$
7.19
6.66
3.95
3.93
3.91
3.89
2.88
2.37
2.34
2.33
2.30
2.26
2.06
2.03
2.00
1.94
1.91
1.86
1.85
1.81
1.78
1.75
1.68
1.66
1.64
1.61
1.56
1.54 H$_2$O
1.48
1.45
1.43
1.40
1.39
1.37
1.34
1.34
1.33
0.88
1.00
1.00
1.14
1.08
1.03
4.77
2.37
2.36
1.19
1.13
3.38
2.07
4.22
3.42
9.5 9.0 8.5 8.0 7.5 7.0 6.5 6.0 5.5 5.0 4.5 4.0 3.5 3.0 2.5 2.0 1.5 1.0 0.5 0.0 -0.5
f1 (ppm)

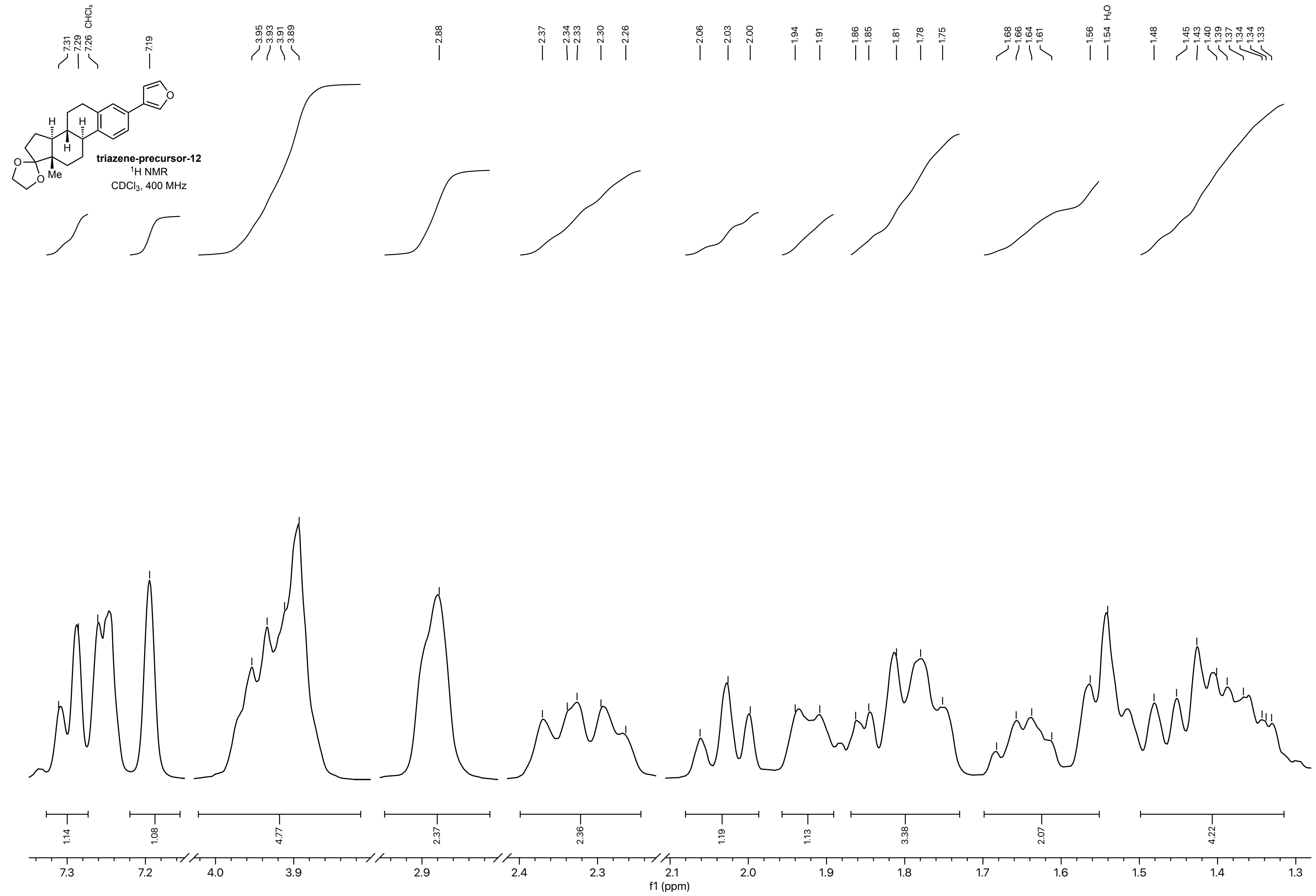
7.31
7.29
7.26 CHCl3
7.19
3.95
3.93
3.91
3.89
2.88
2.37
2.34
2.33
2.30
2.26
2.06
2.03
2.00
1.94
1.91
1.86
1.85
1.81
1.78
1.75
1.68
1.66
1.64
1.61
1.56
1.54 H2O
1.48
1.45
1.43
1.40
1.39
1.37
1.34
1.34
1.33
H
H
H
O
O
O
Me
triazene-precursor-12
1H NMR
CDCl3, 400 MHz
1.14
1.08
4.77
2.37
2.36
1.19
1.13
3.38
2.07
4.22
7.3
7.2
4.0
3.9
2.9
2.4
2.3
2.1
2.0
1.9
1.8
1.7
1.6
1.5
1.4
1.3
f1 (ppm)

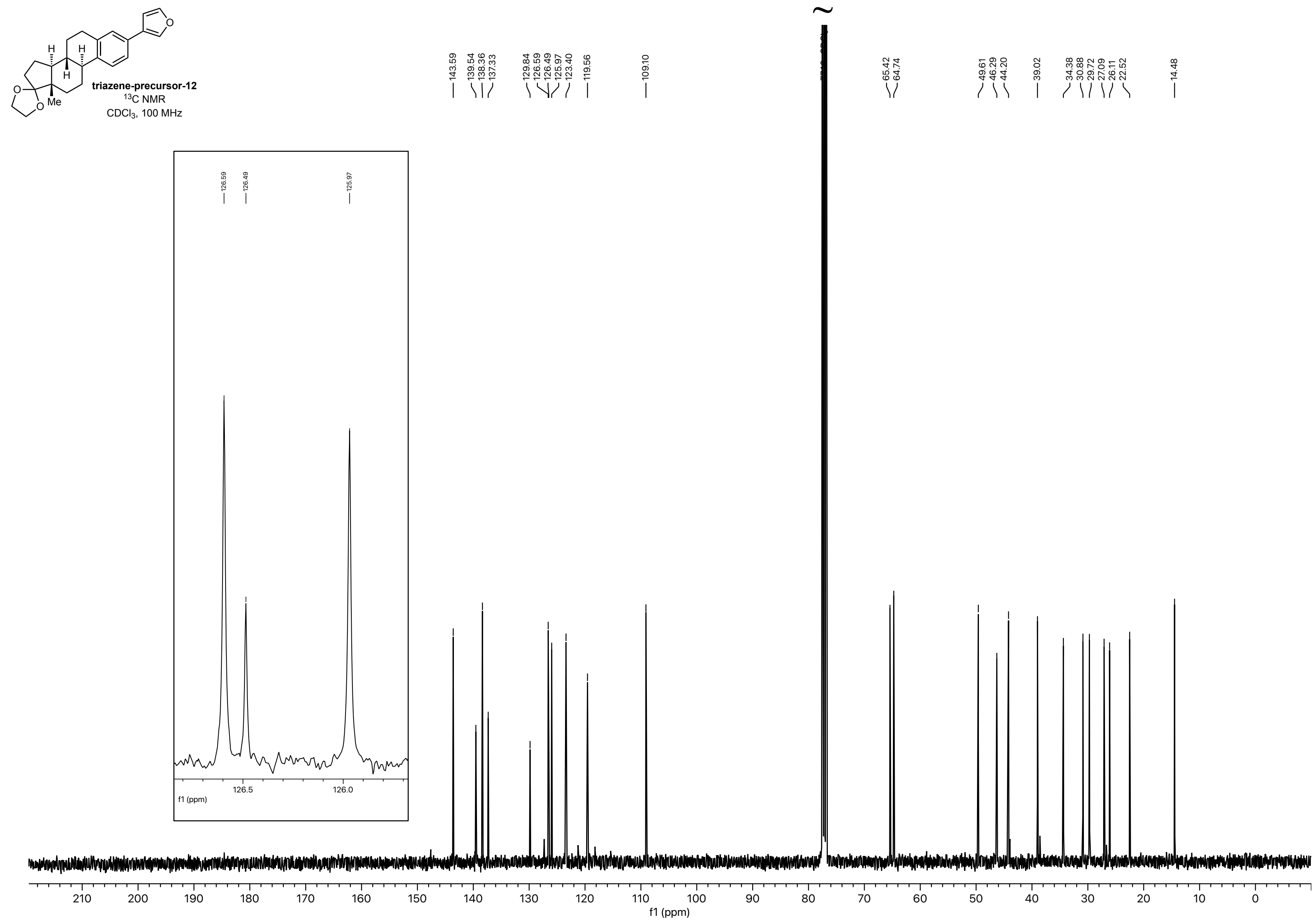

H
H
H
O
O
Me
O
triazene-precursor-12
^{13}C NMR
$CDCl_3$, 100 MHz
143.59
139.54
138.36
137.33
129.84
126.59
126.49
125.97
123.40
119.56
109.10
65.42
64.74
49.61
46.29
44.20
39.02
34.38
30.88
29.72
27.09
26.11
22.52
14.48
126.5
126.0
f1 (ppm)
210
200
190
180
170
160
150
140
130
120
110
100
90
80
70
60
50
40
30
20
10
0
f1 (ppm)

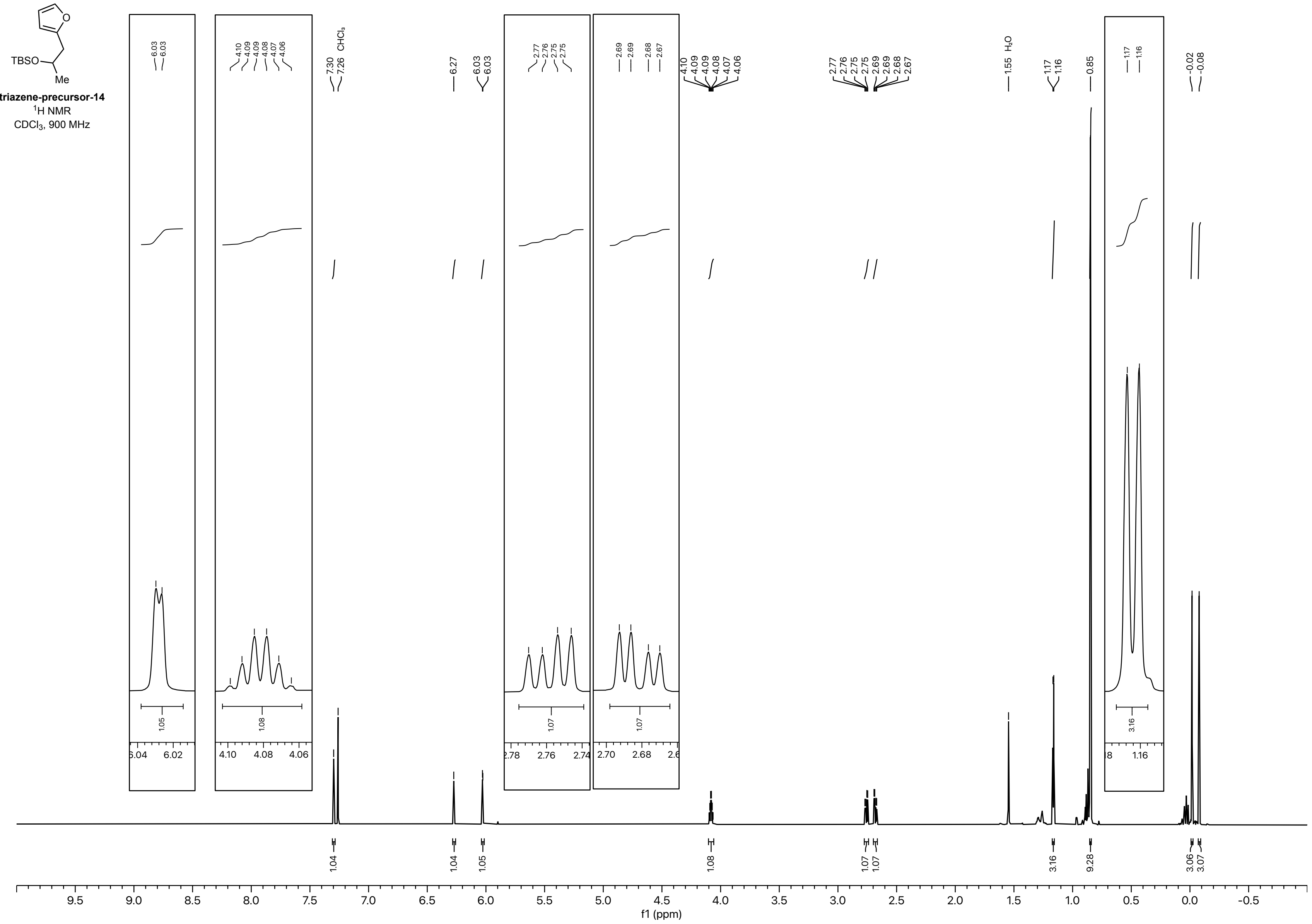

triazene-precursor-14
^{1}H NMR
$CDCl_3$, 900 MHz

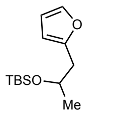

triazene-precursor-14
^{13}C NMR
$CDCl_3$, 100 MHz

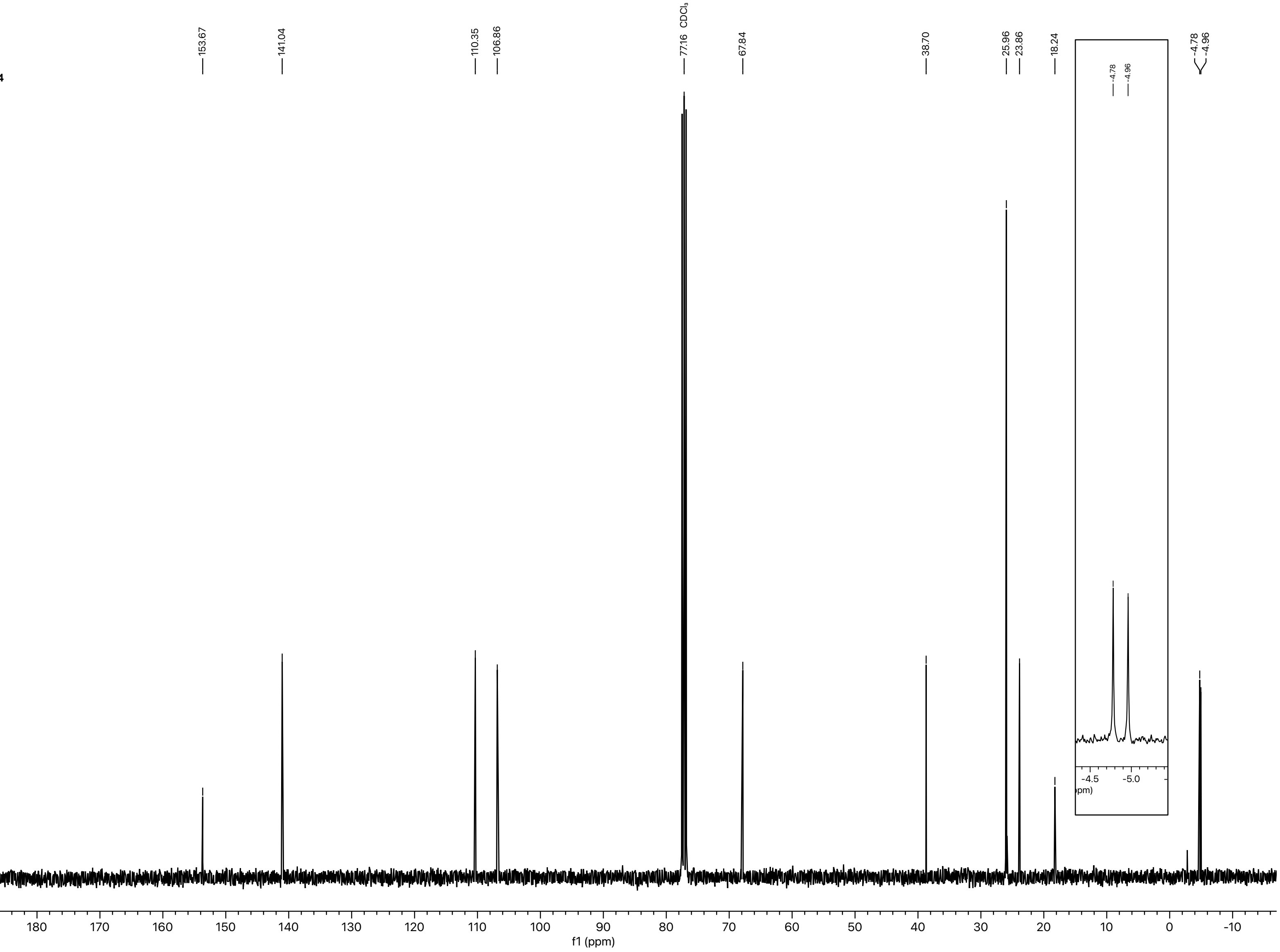

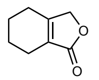

triazene-precursor-17
^1H NMR
$CDCl_3$, 400 MHz

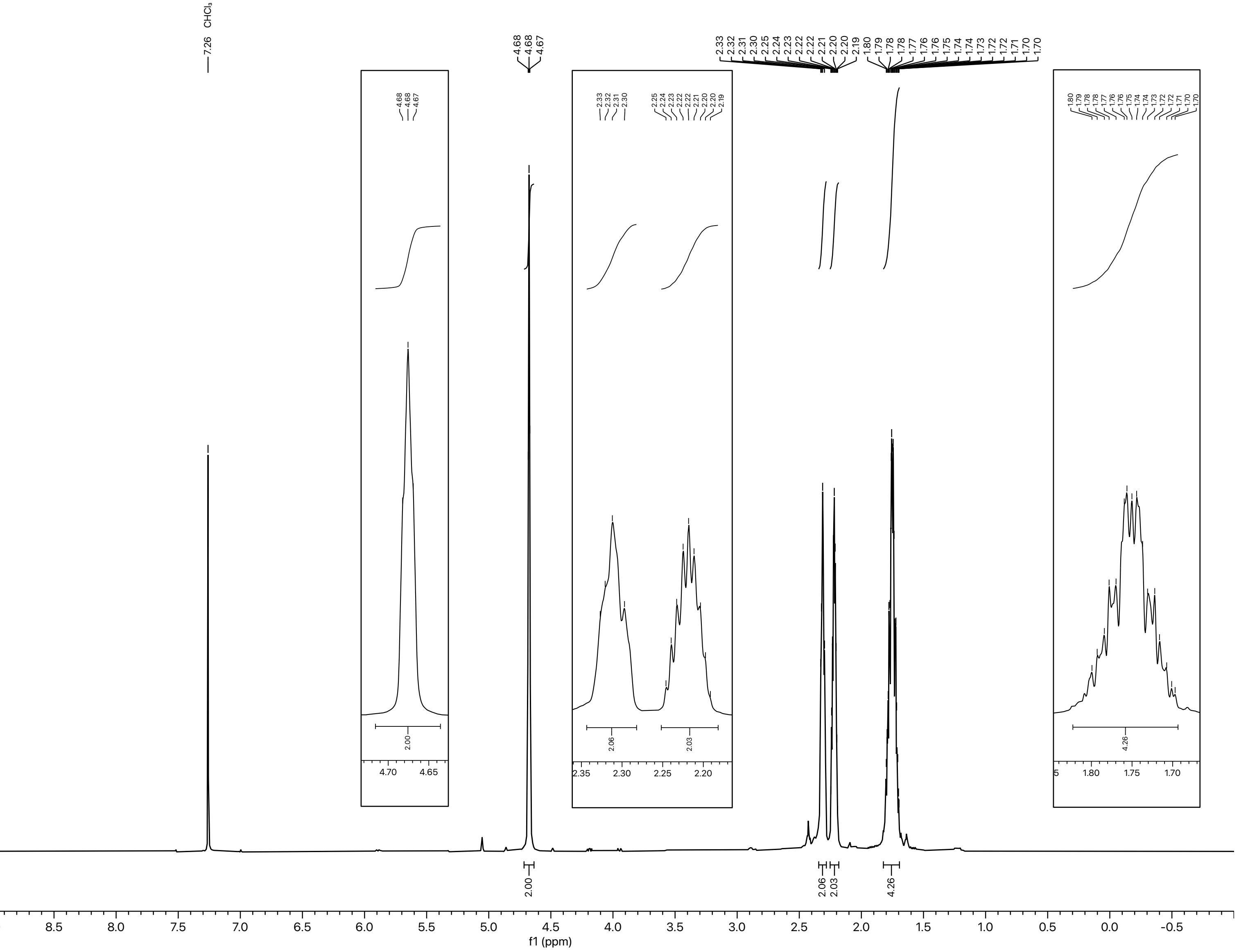

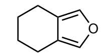

triazene-precursor-18
^1H NMR
$CDCl_3$, 400 MHz

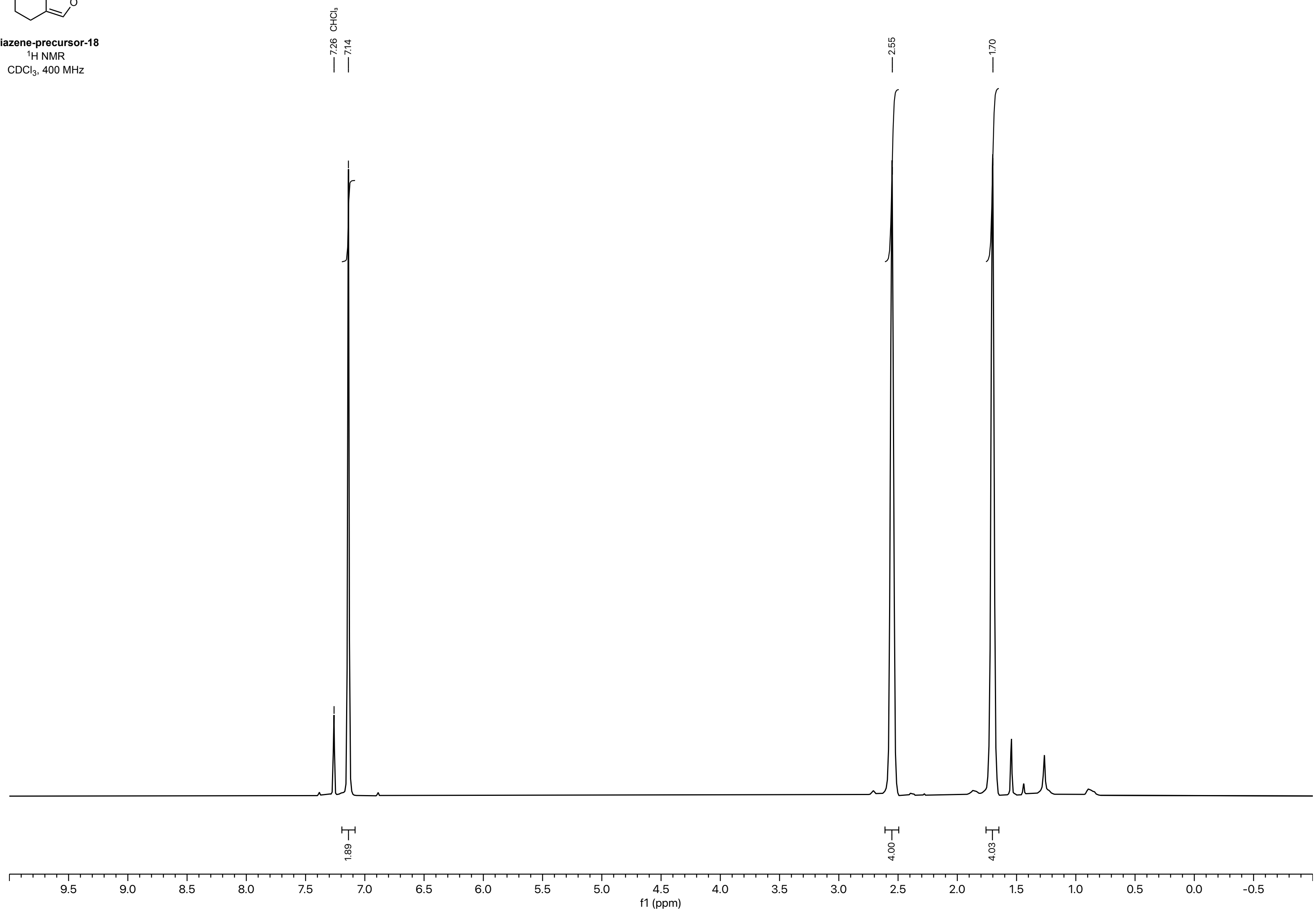

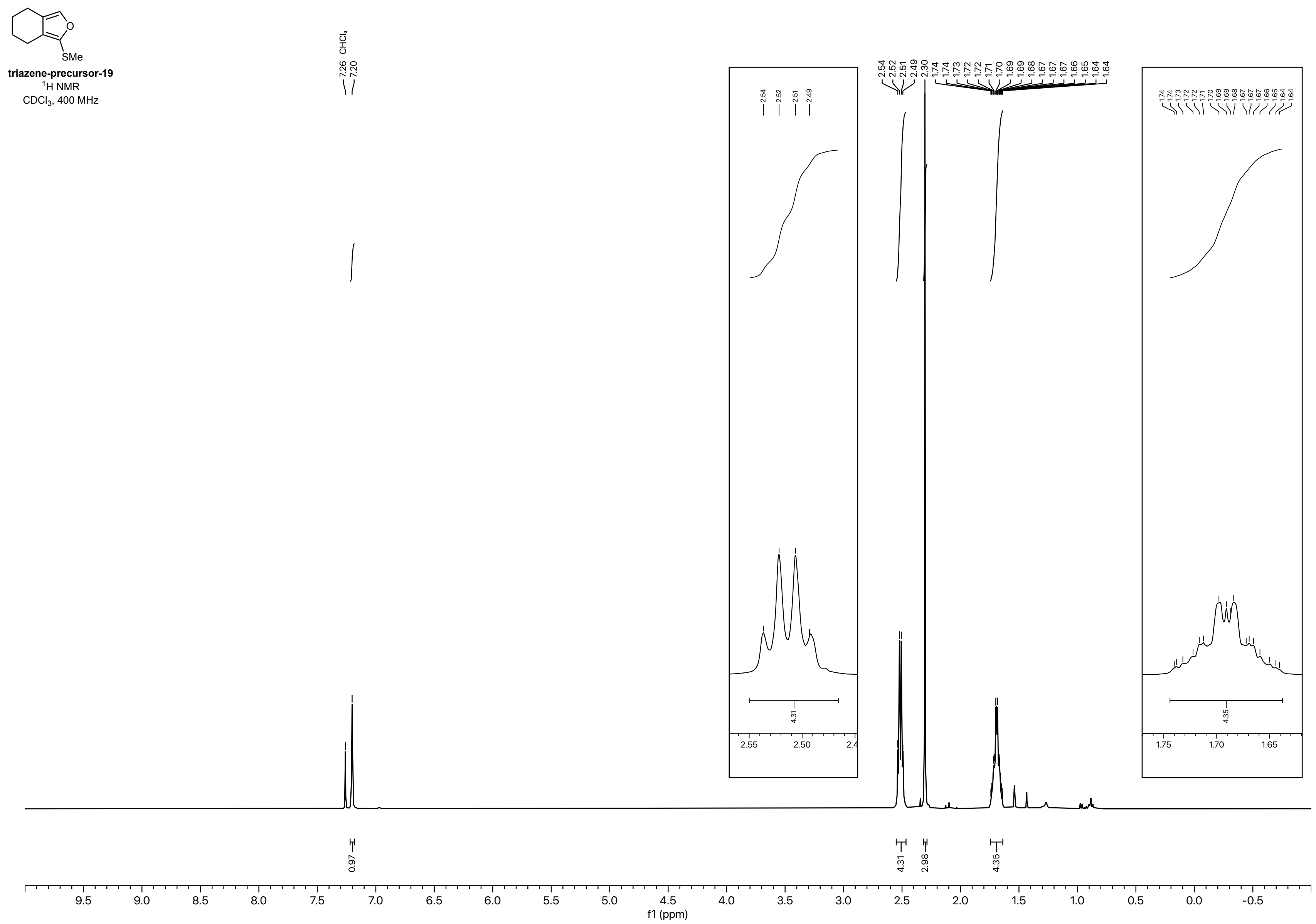
SMe
triazene-precursor-19
1H NMR
CDCl3, 400 MHz
7.26 CHCl3
7.20
2.54
2.52
2.51
2.49
2.30
1.74
1.74
1.73
1.72
1.72
1.71
1.70
1.69
1.69
1.68
1.67
1.67
1.67
1.66
1.65
1.64
1.64
0.97
4.31
2.98
4.35
f1 (ppm)

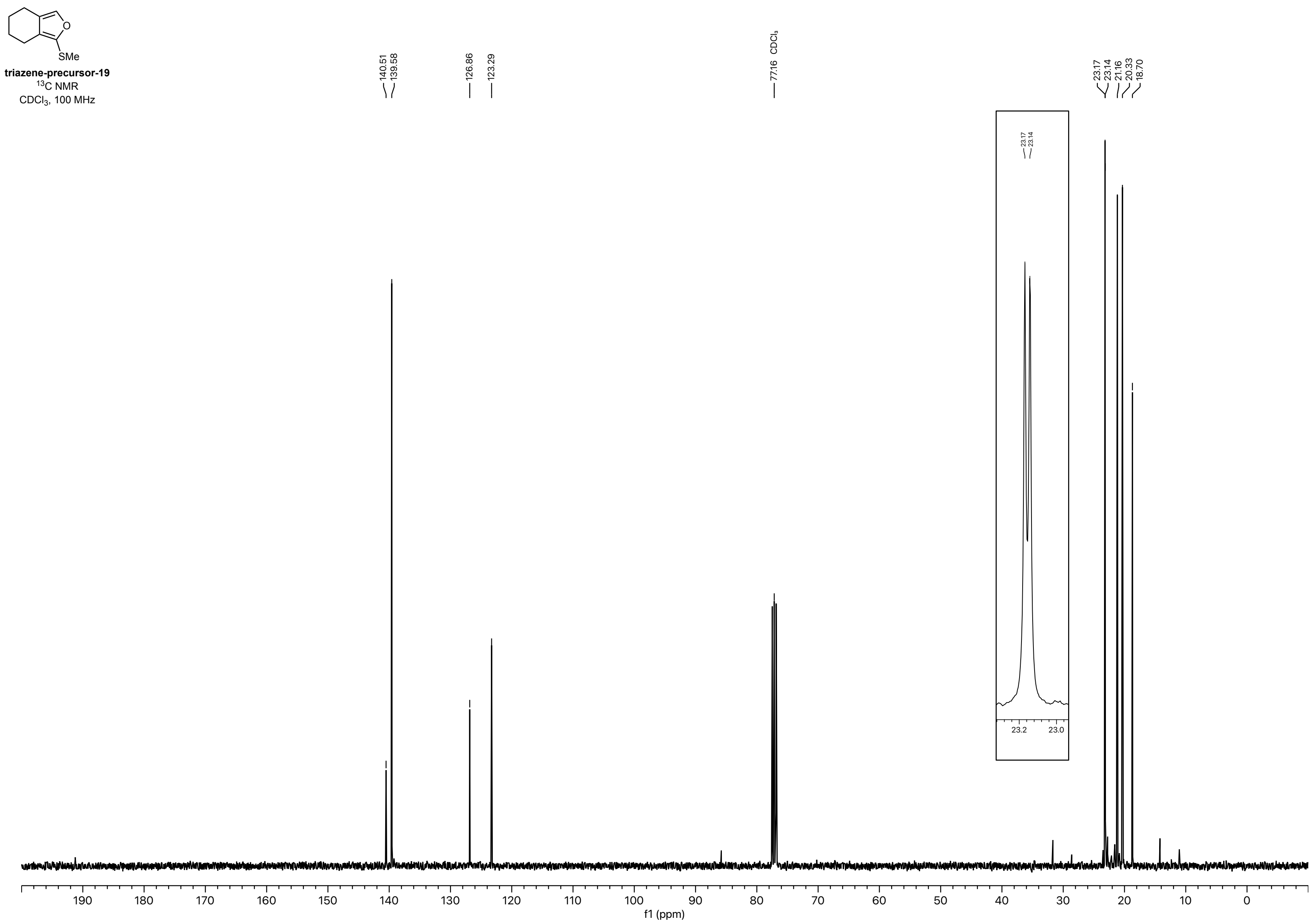
SMe
O
triazene-precursor-19
13C NMR
CDCl3, 100 MHz
140.51
139.58
126.86
123.29
77.16 CDCl3
23.17
23.14
21.16
20.33
18.70
23.2
23.0
190
180
170
160
150
140
130
120
110
100
90
80
70
60
50
40
30
20
10
0
f1 (ppm)

One-Pot Triazenylation/Intramolecular Alkylation

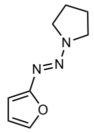

2-triazenyl-furan-01 (or **1**)
^{1}H NMR
$CDCl_3$, 400 MHz

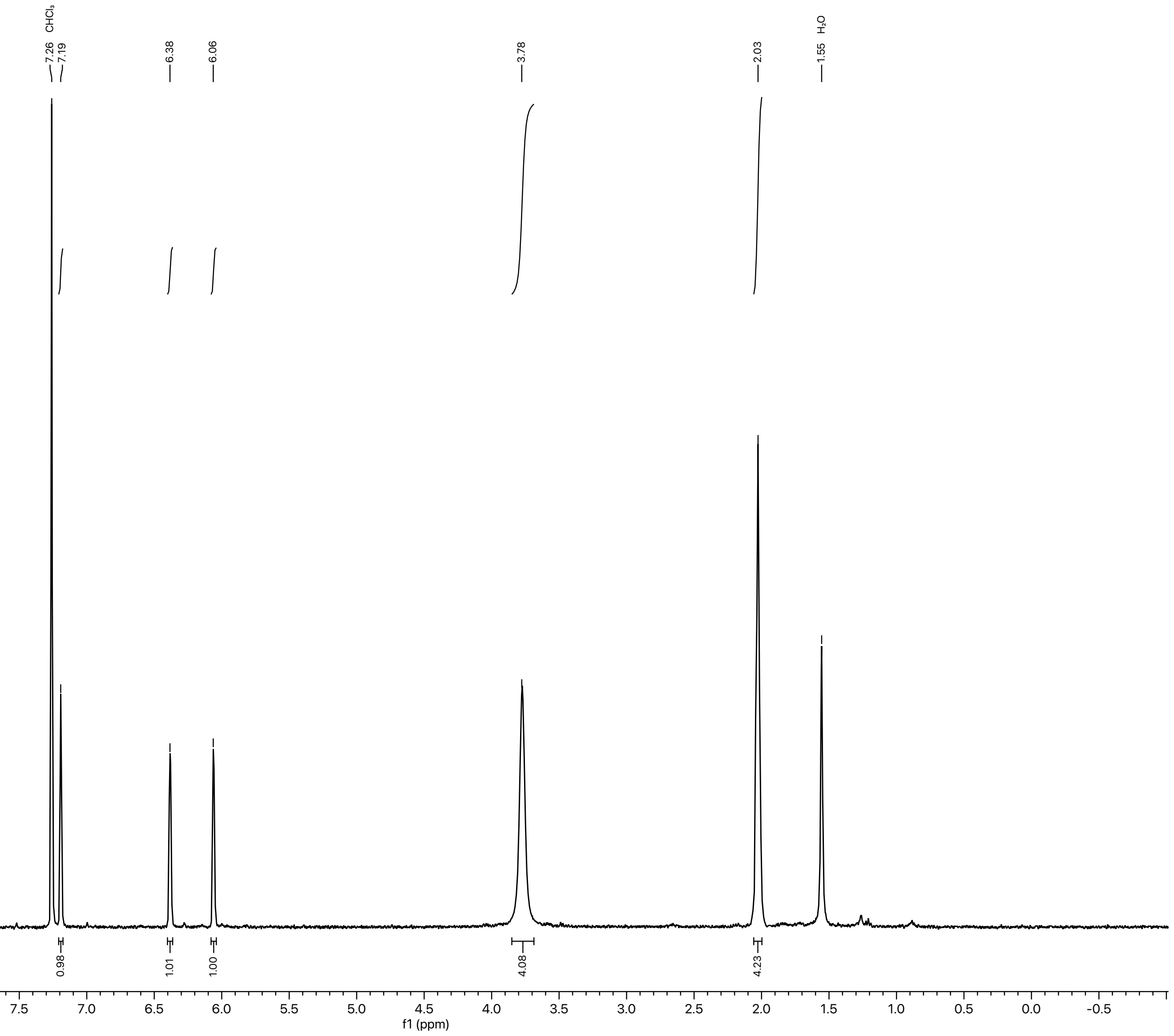

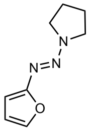

2-triazenyl-furan-01 (or **1**)
^{13}C NMR
$CDCl_3$, 100 MHz

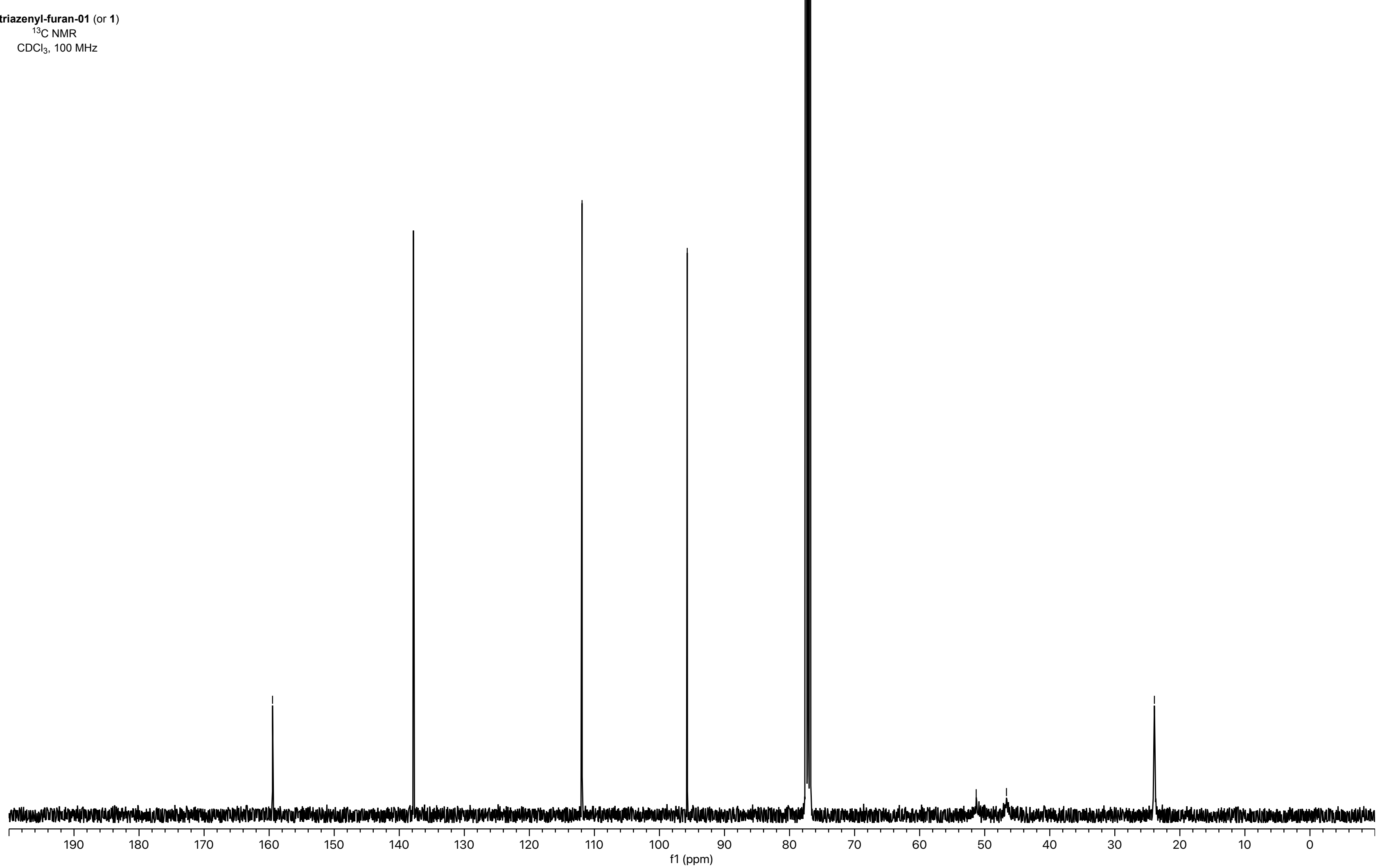

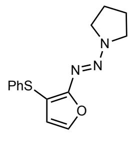

2-triazenyl-furan-02
^{1}H NMR
$CDCl_3$, 600 MHz

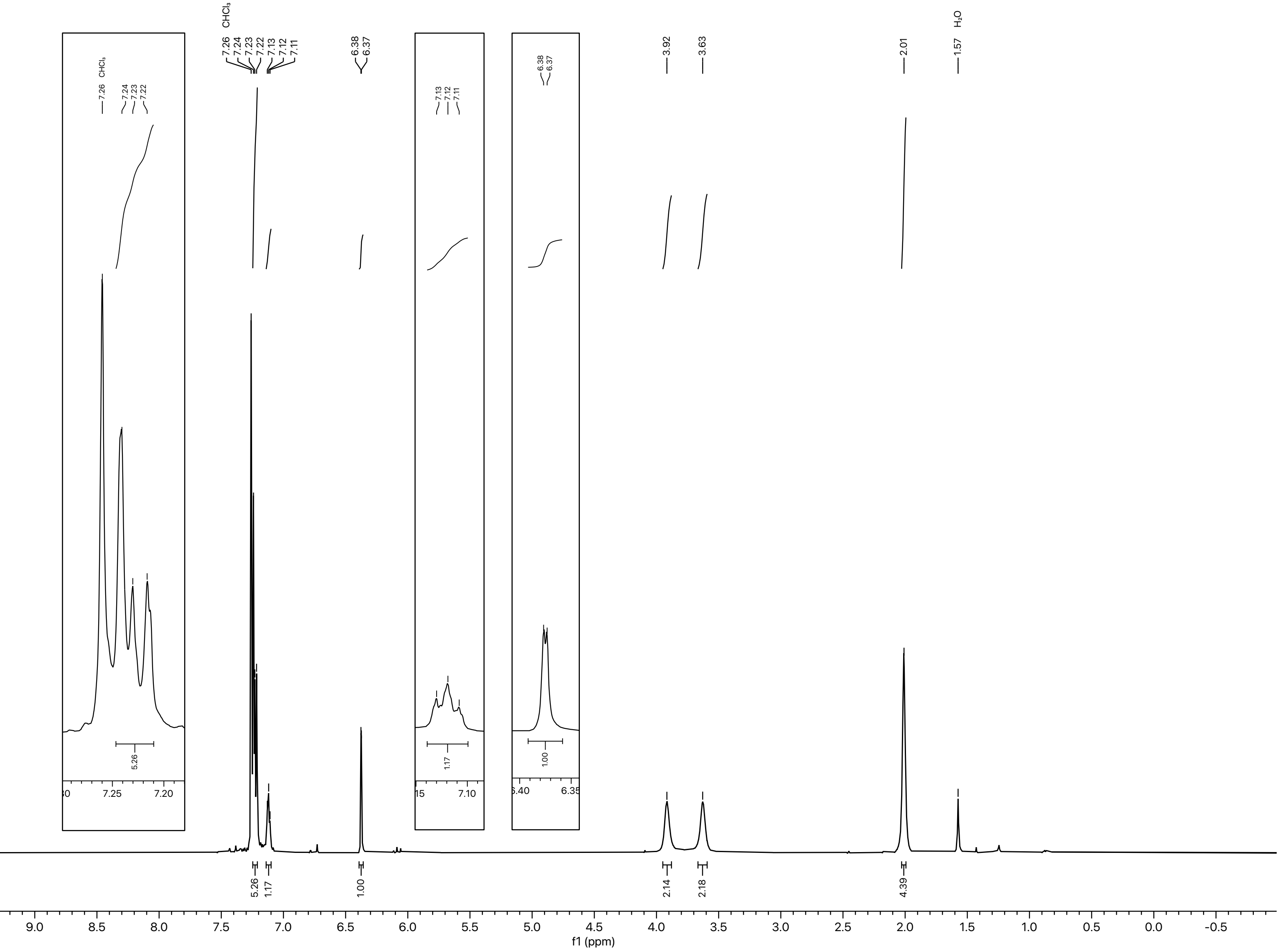

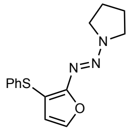

2-triazenyl-furan-02

^{13}C NMR

$CDCl_3$, 100 MHz

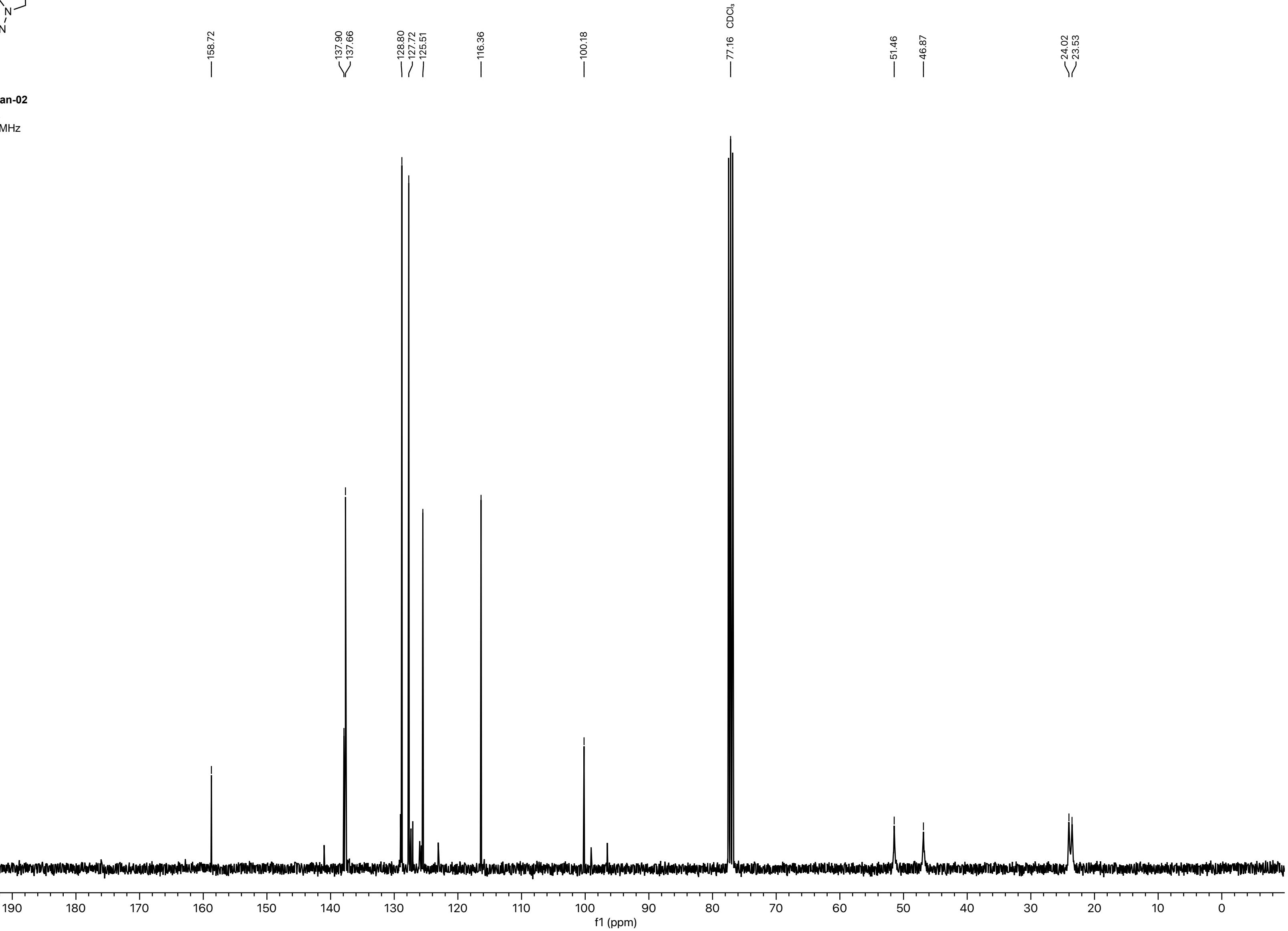

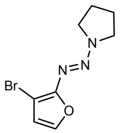

2-triazenyl-furan-03 (or **2**)
^{1}H NMR
$CDCl_3$, 400 MHz

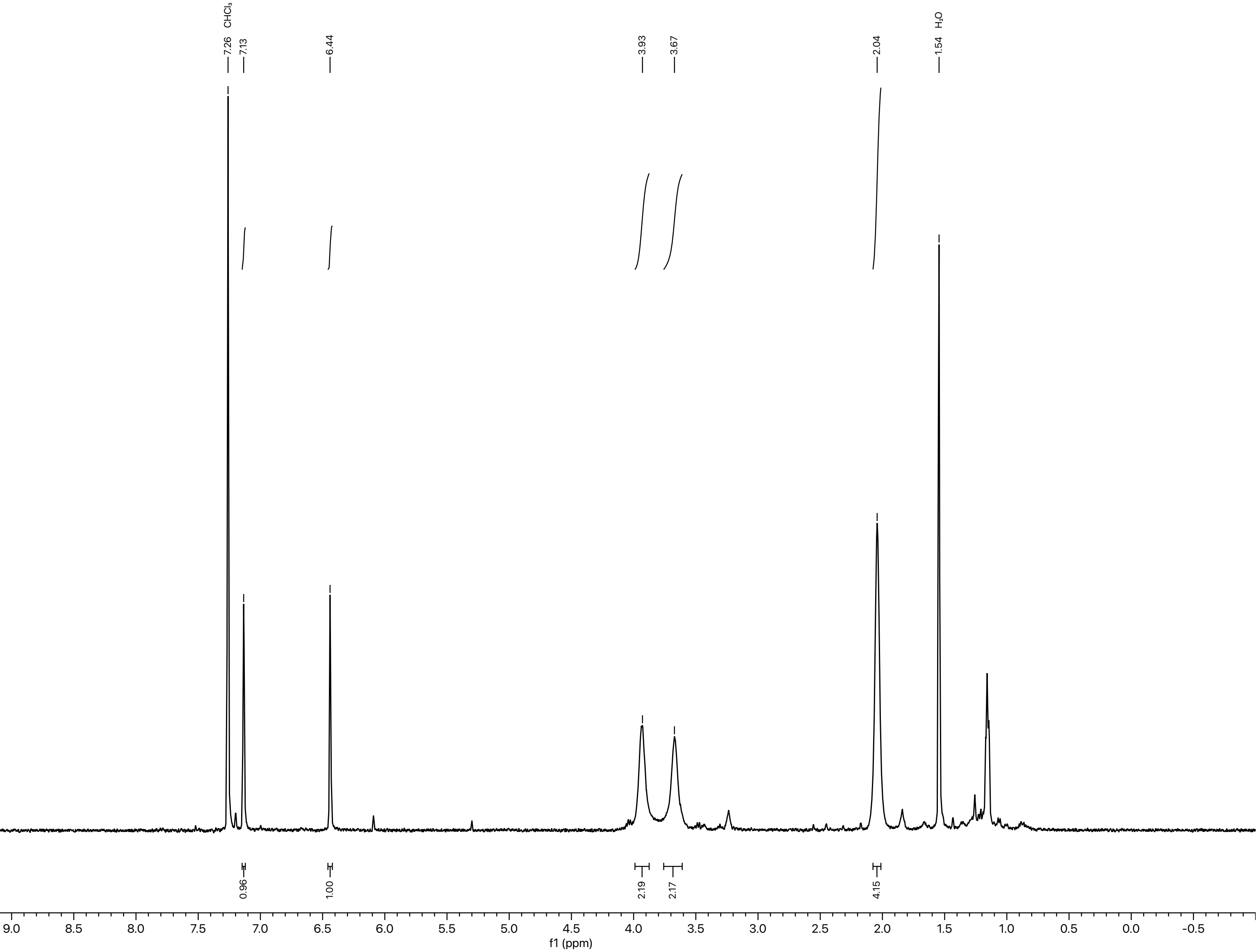

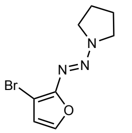

2-triazenyl-furan-03 (or **2**)
^{13}C NMR
$CDCl_3$, 100 MHz

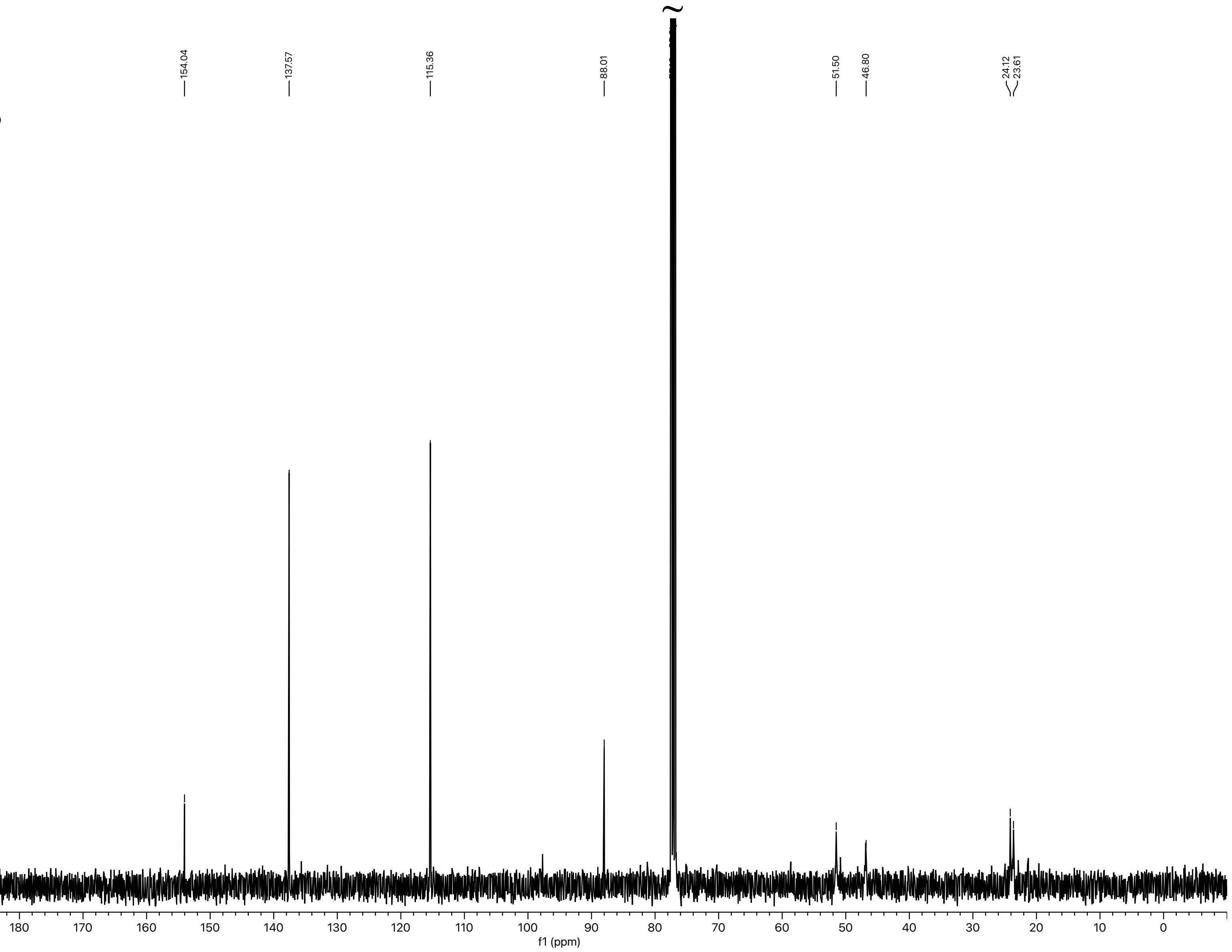

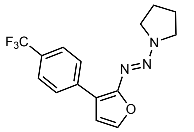

2-triazenyl-furan-04
^{1}H NMR
$CDCl_3$, 400 MHz

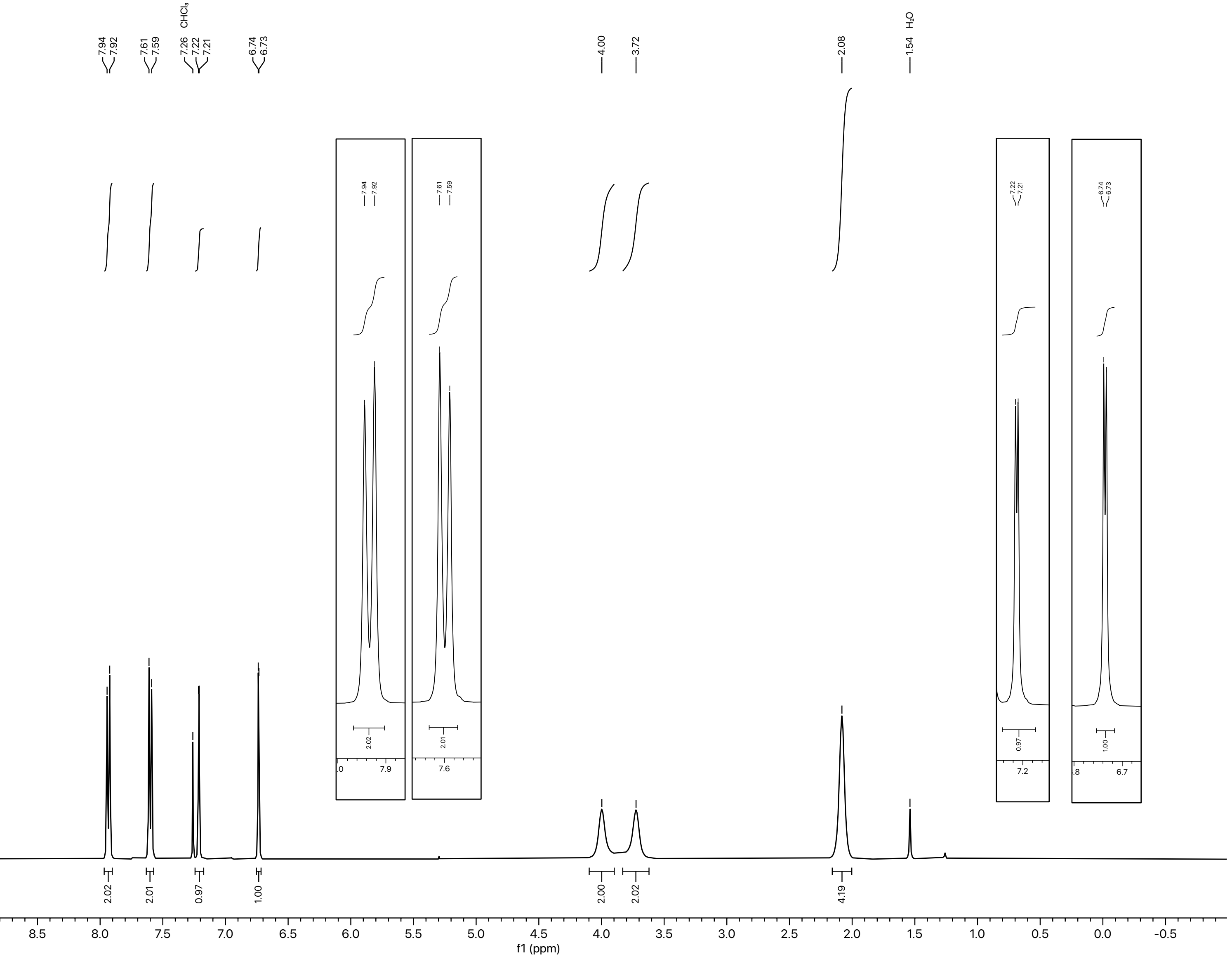

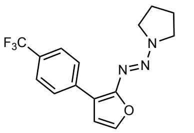

2-triazenyl-furan-04
^{13}C NMR
$CDCl_3$, 100 MHz

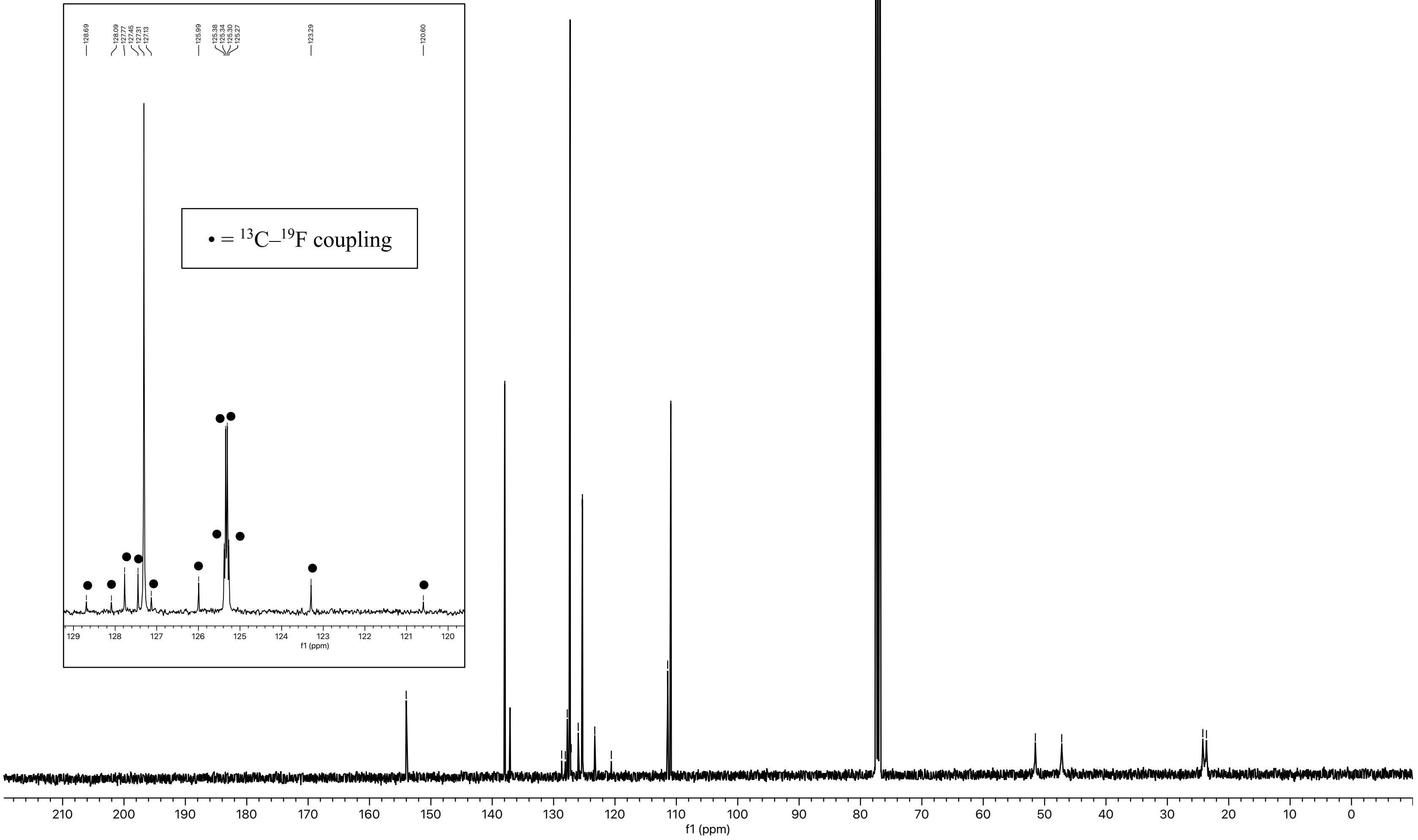

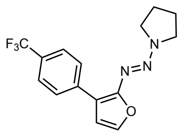

2-triazenyl-furan-04
^{19}F NMR
$CDCl_3$, 377 MHz

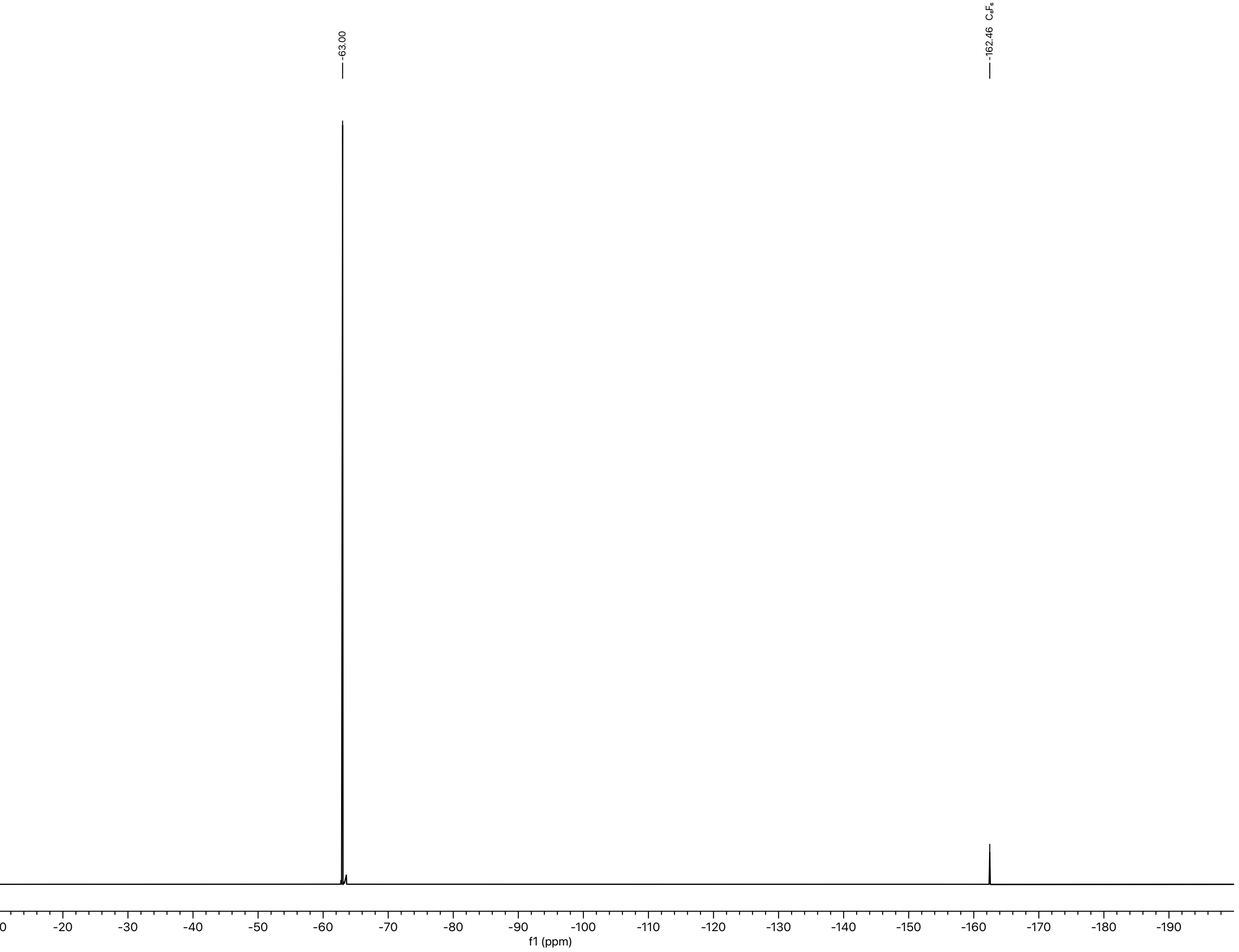

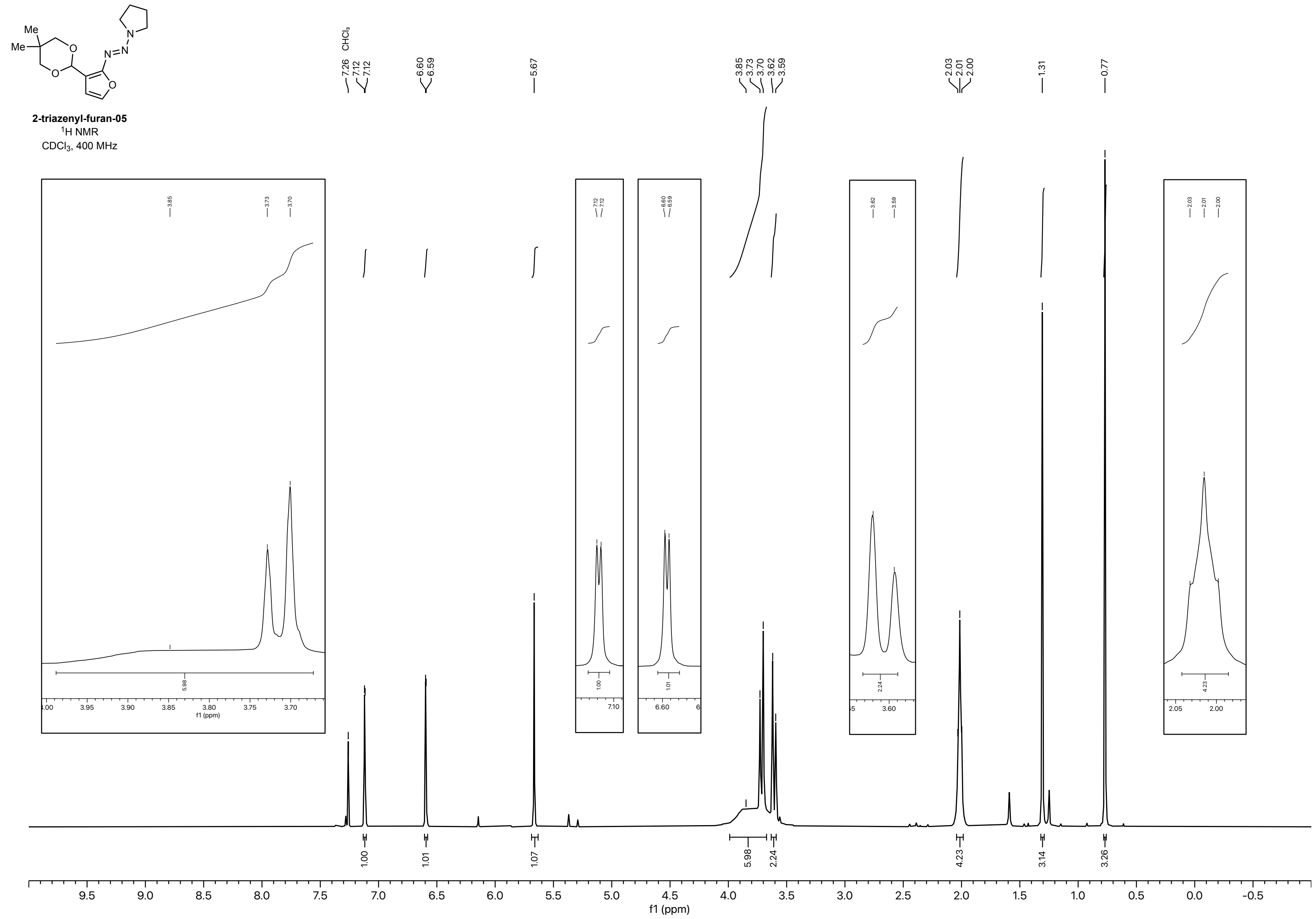

2-triazenyl-furan-05
^{1}H NMR
$CDCl_3$, 400 MHz

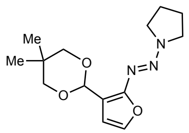

2-triazenyl-furan-05
^{13}C NMR
$CDCl_3$, 100 MHz

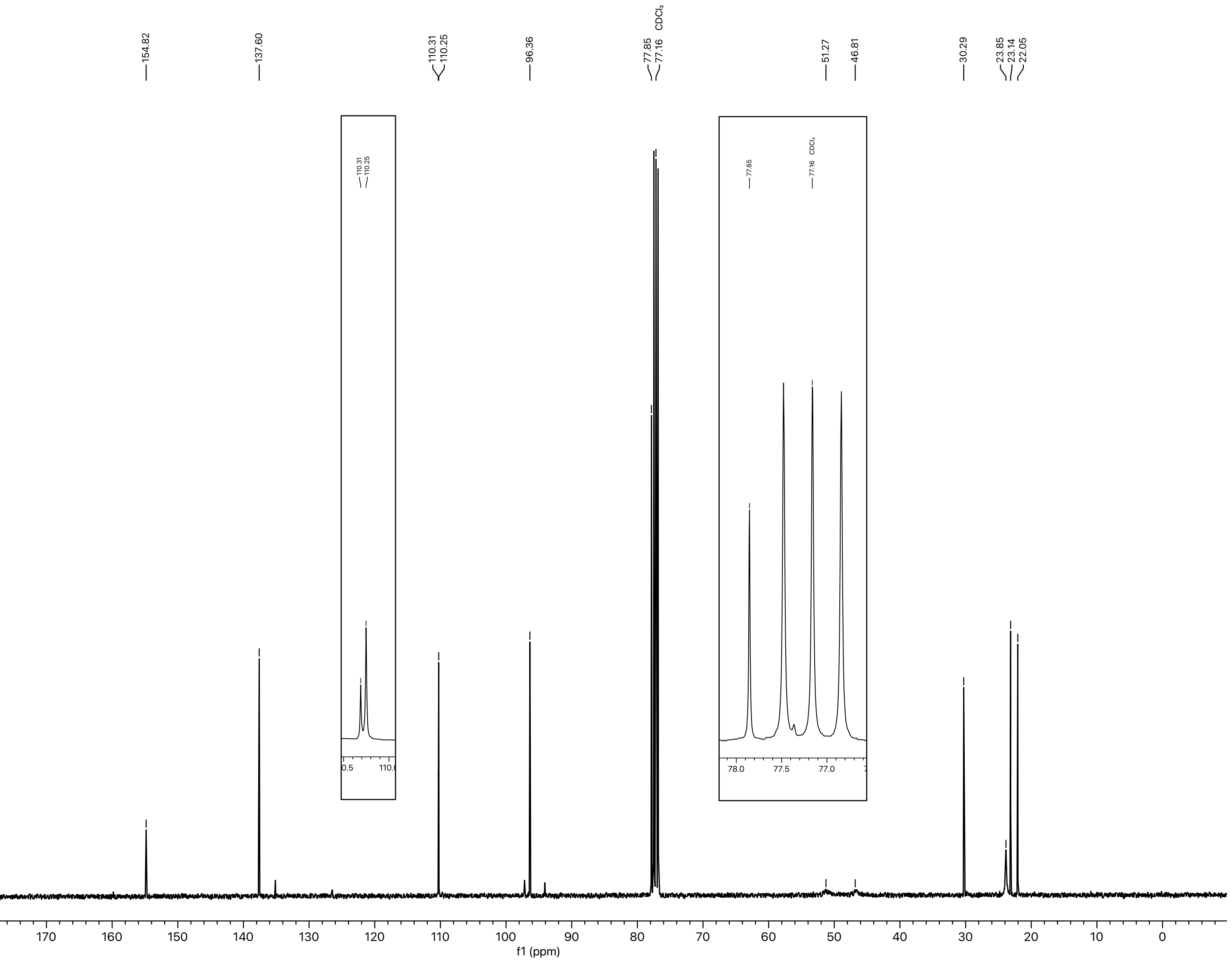

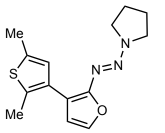

2-triazenyl-furan-06
^1H NMR
$CDCl_3$, 400 MHz

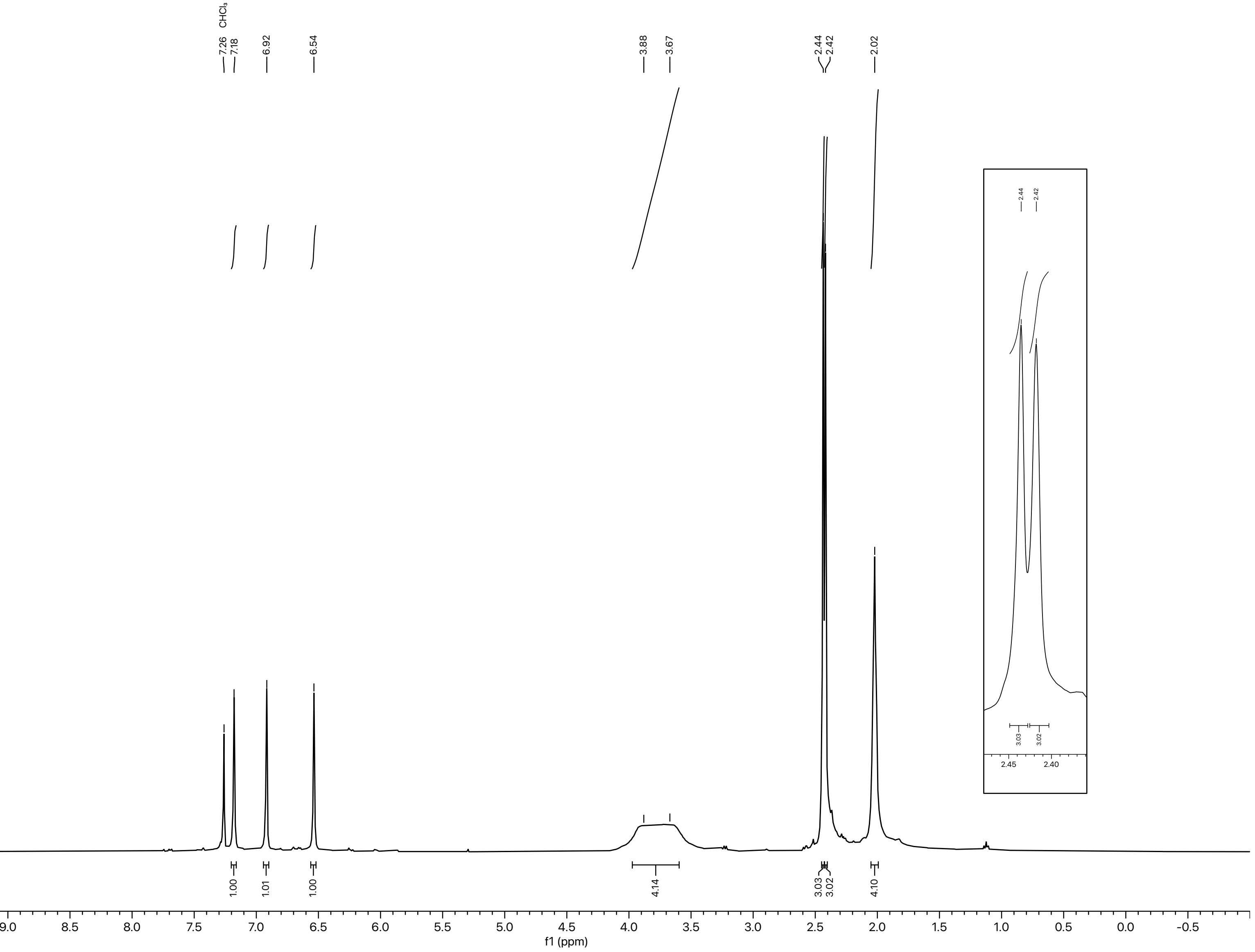

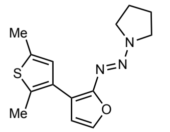

2-triazenyl-furan-06
^{13}C NMR
$CDCl_3$, 100 MHz

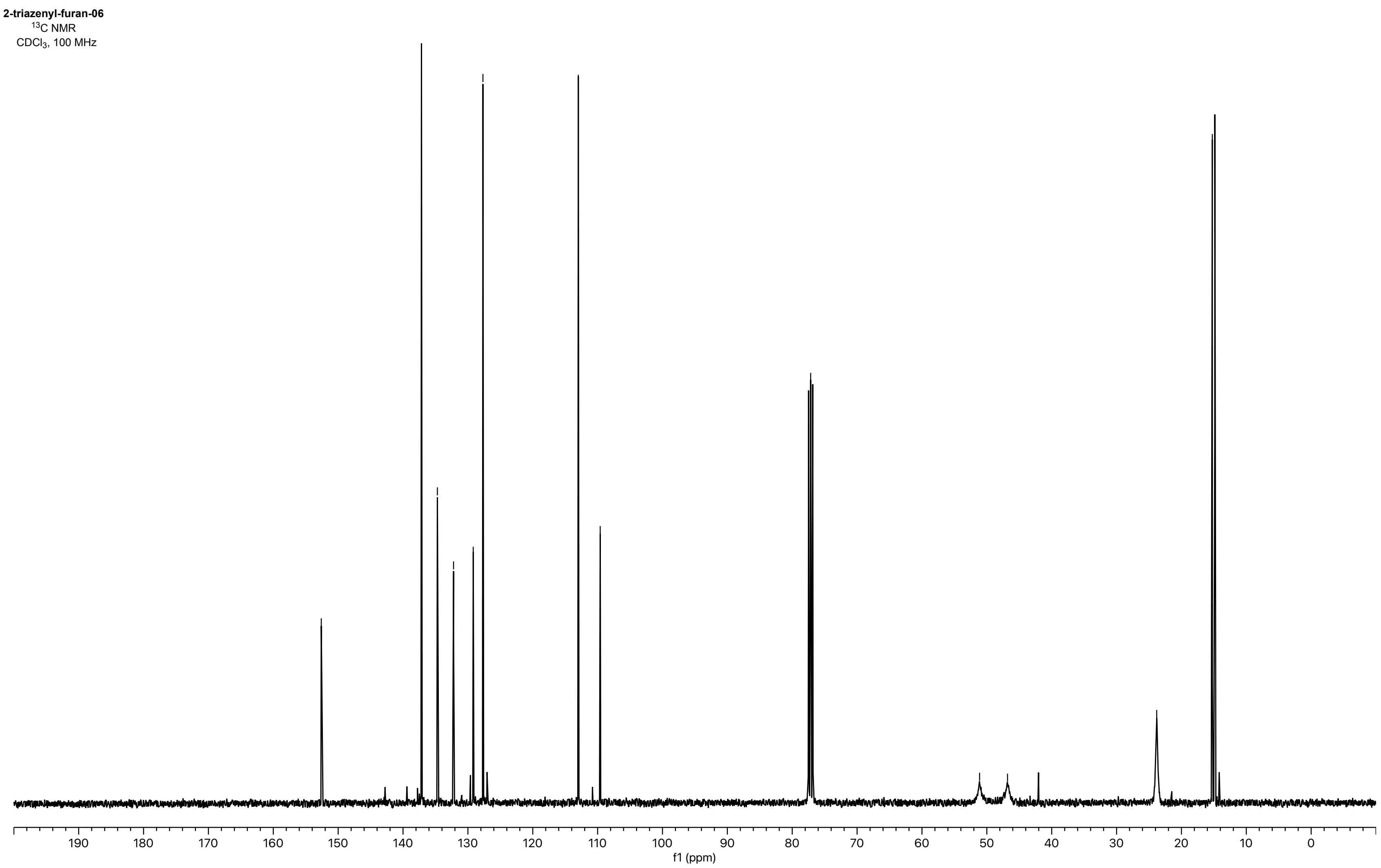

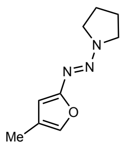

2-triazenyl-furan-07
^{1}H NMR
$CDCl_3$, 400 MHz

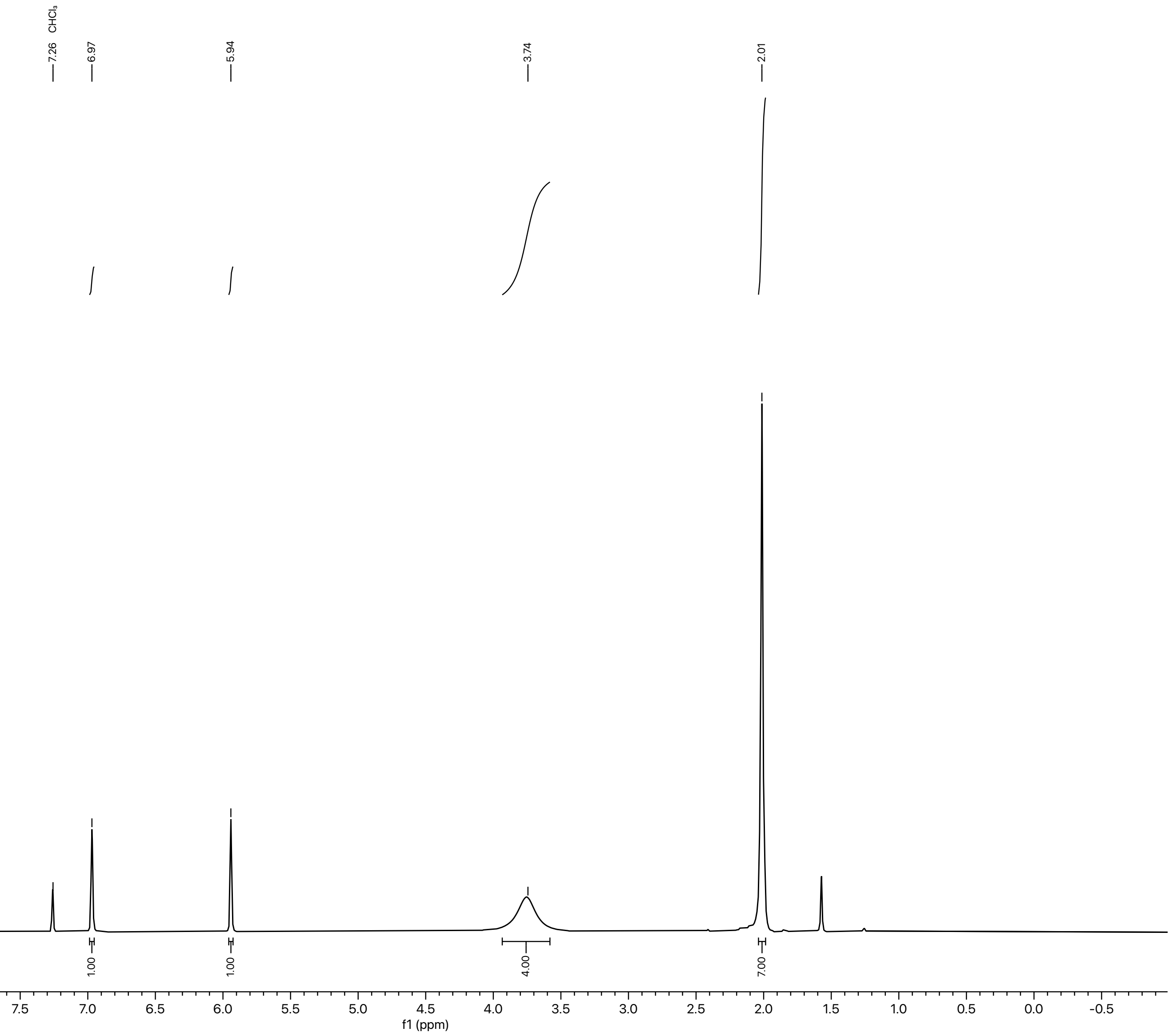

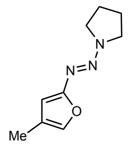

2-triazenyl-furan-07
^{13}C NMR
$CDCl_3$, 100 MHz

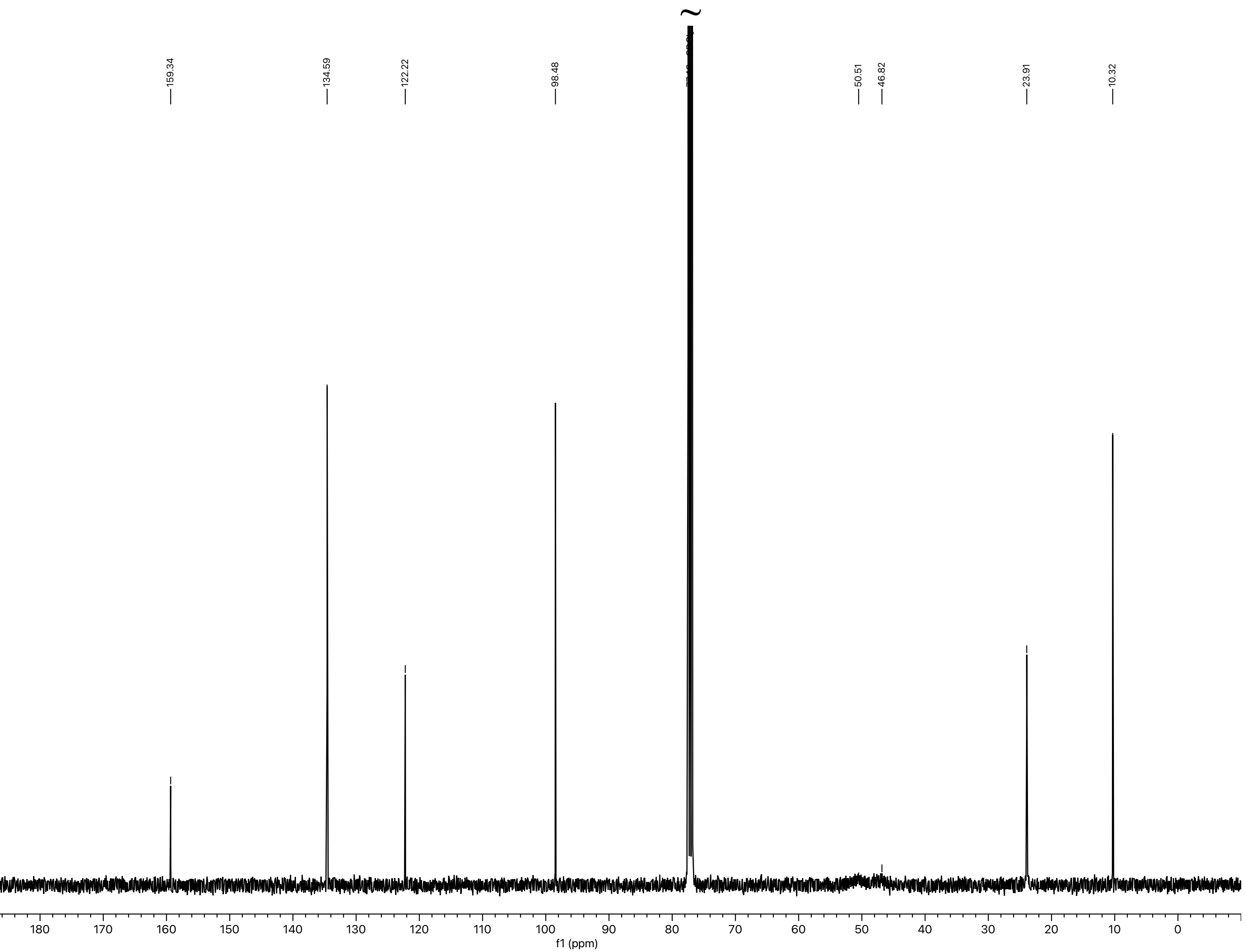

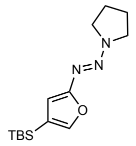

2-triazenyl-furan-08
^1H NMR
$CDCl_3$, 400 MHz

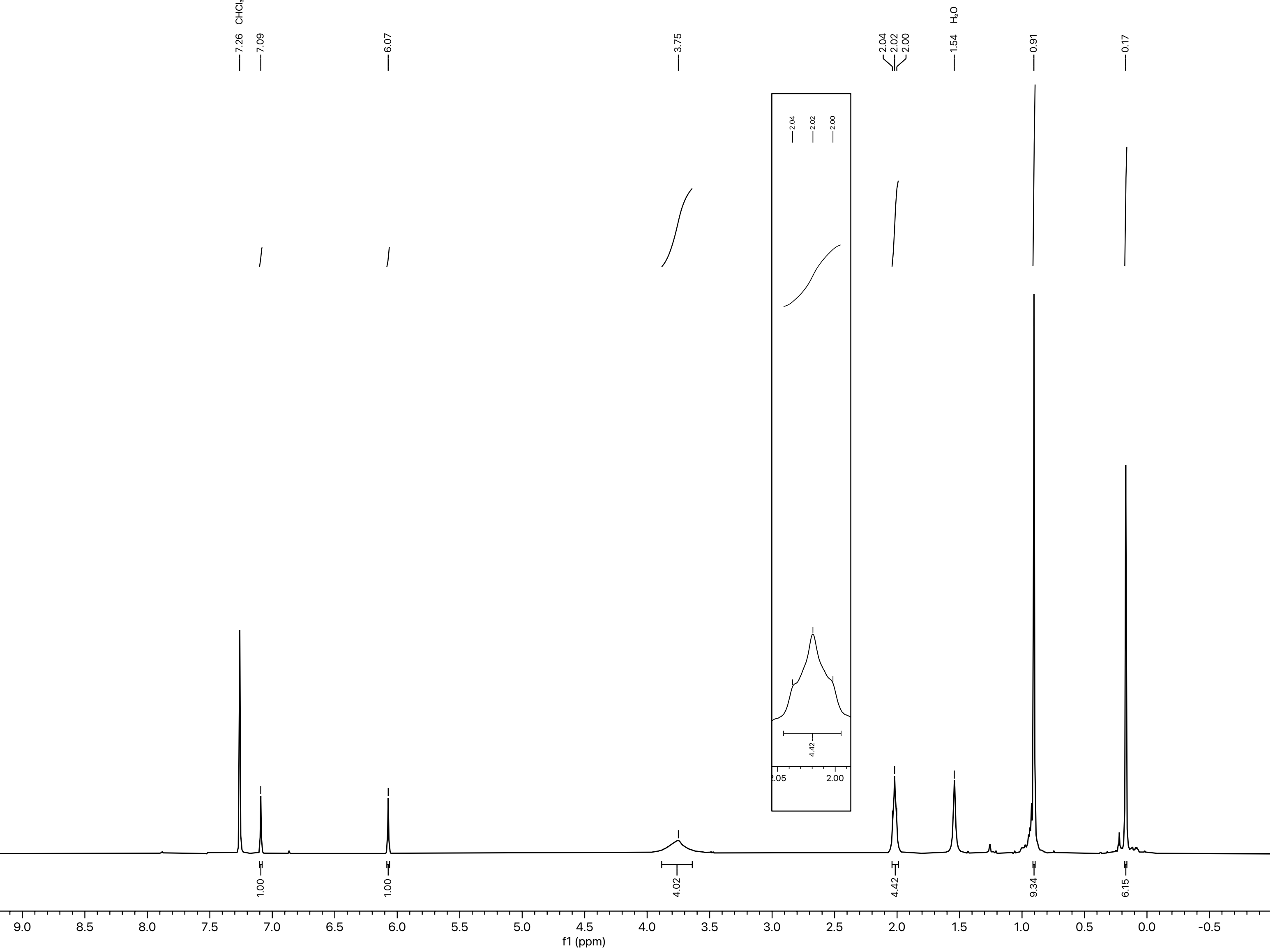

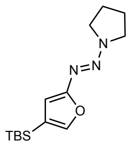

2-triazenyl-furan-08
^{13}C NMR
$CDCl_3$, 150 MHz

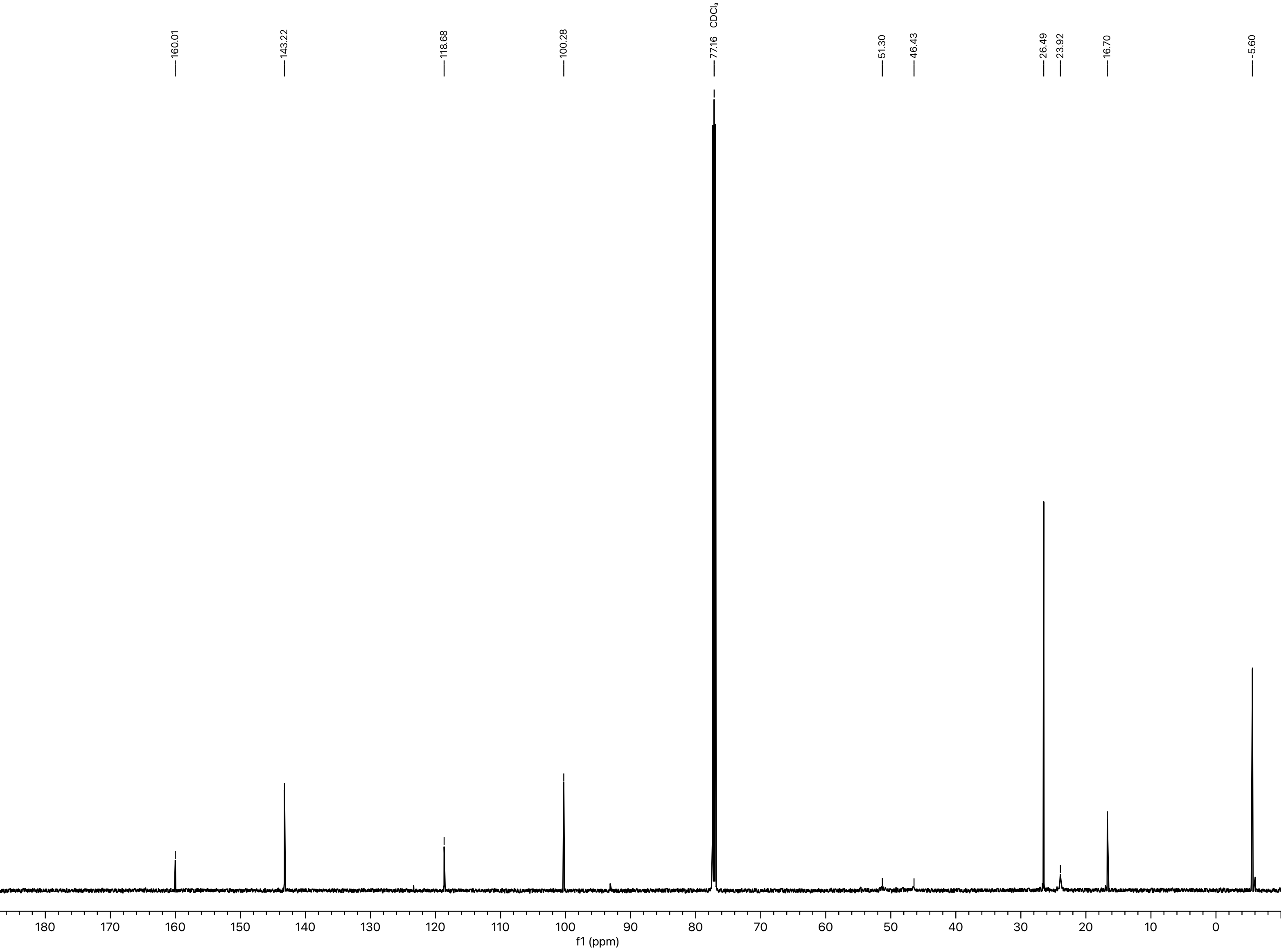

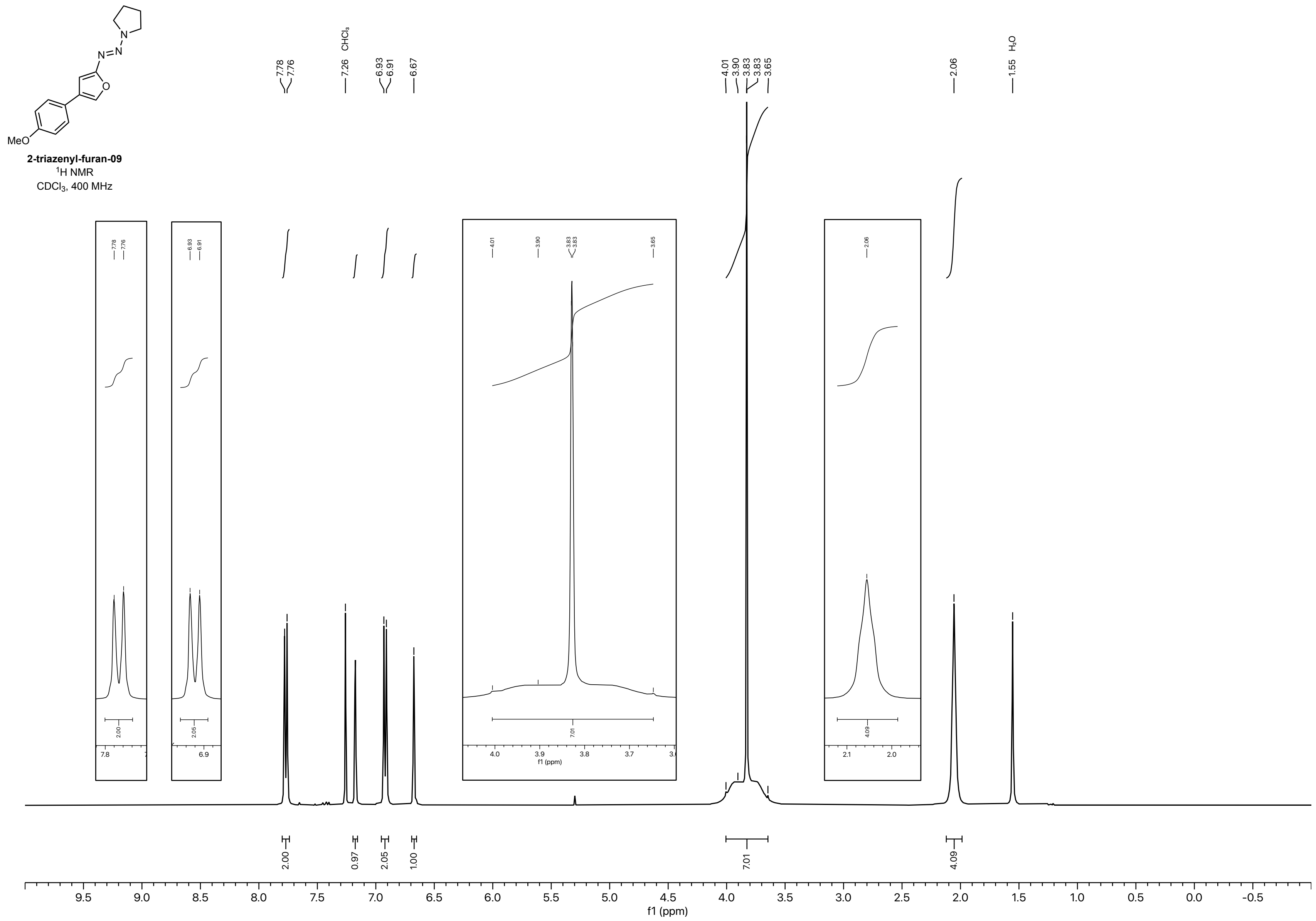

MeO
N=N
N
O
2-triazenyl-furan-09
^{1}H NMR
$CDCl_3$, 400 MHz
7.78
7.76
7.26 $CHCl_3$
6.93
6.91
6.67
4.01
3.90
3.83
3.83
3.65
2.06
1.55 H_2O
2.00
0.97
2.05
1.00
7.01
4.09
f1 (ppm)

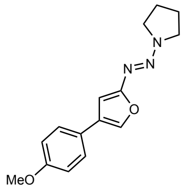

2-triazenyl-furan-09
^{13}C NMR
$CDCl_3$, 100 MHz

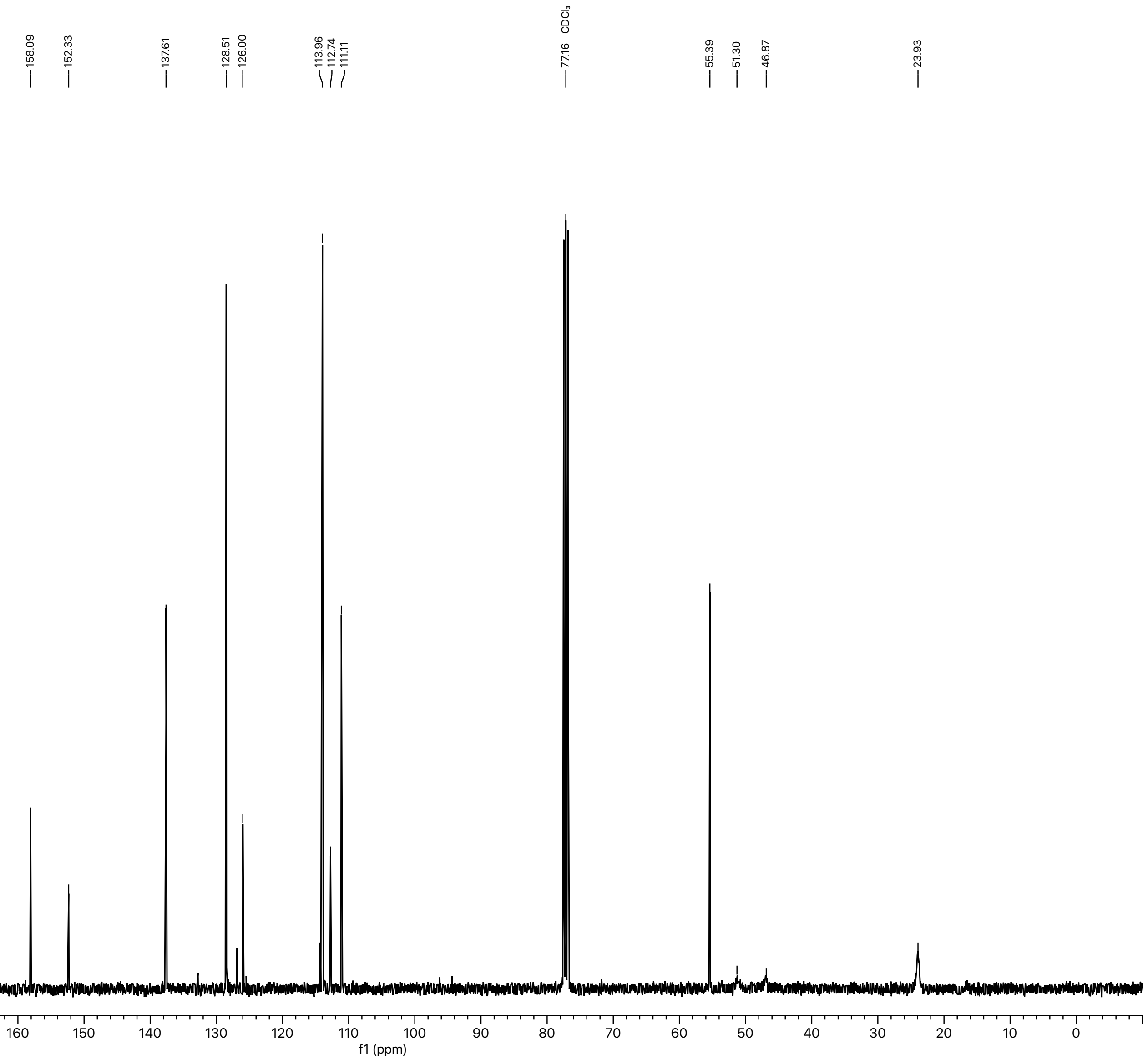

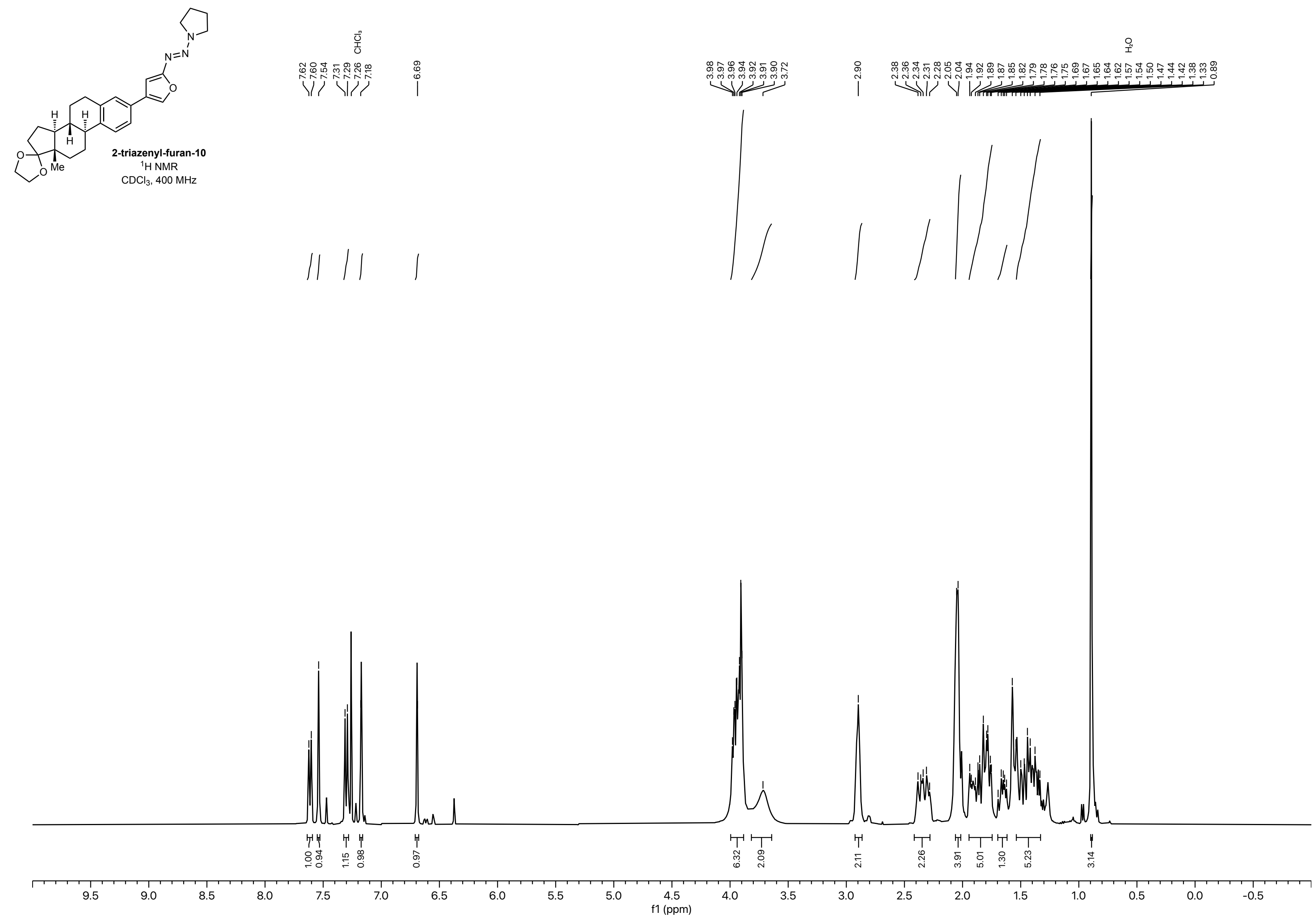
2-triazenyl-furan-10
¹H NMR
CDCl₃, 400 MHz
7.62 7.60 7.54 7.31 7.29 7.26 7.18 CHCl₃
6.69
3.98 3.97 3.96 3.94 3.92 3.91 3.90 3.72
2.90
2.38 2.36 2.34 2.31 2.28 2.05 2.04 1.94 1.92 1.89 1.87 1.85 1.82 1.79 1.78 1.76 1.75 1.69 1.67 1.65 1.64 1.62 1.57 1.54 1.50 1.47 1.44 1.42 1.38 1.33 0.89
H₂O
1.00 0.94 1.15 0.98 0.97 6.32 2.09 2.11 2.26 3.91 5.01 1.30 5.23 3.14
f1 (ppm)

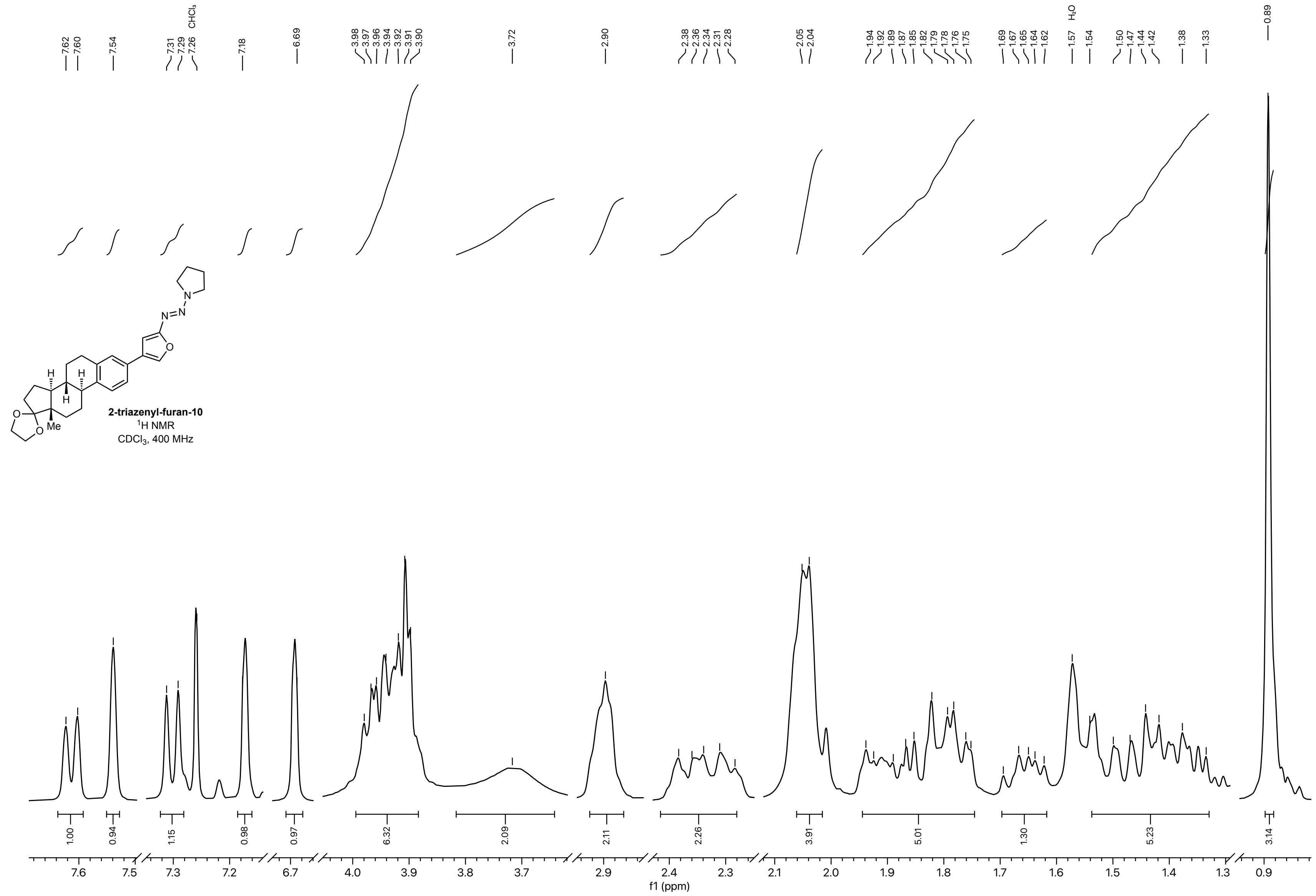
2-triazenyl-furan-10
^{1}H NMR
$CDCl_3$, 400 MHz
f1 (ppm)

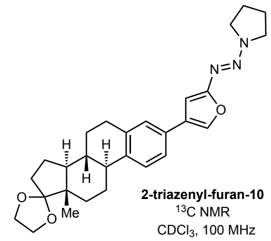

2-triazenyl-furan-10
^{13}C NMR
$CDCl_3$, 100 MHz

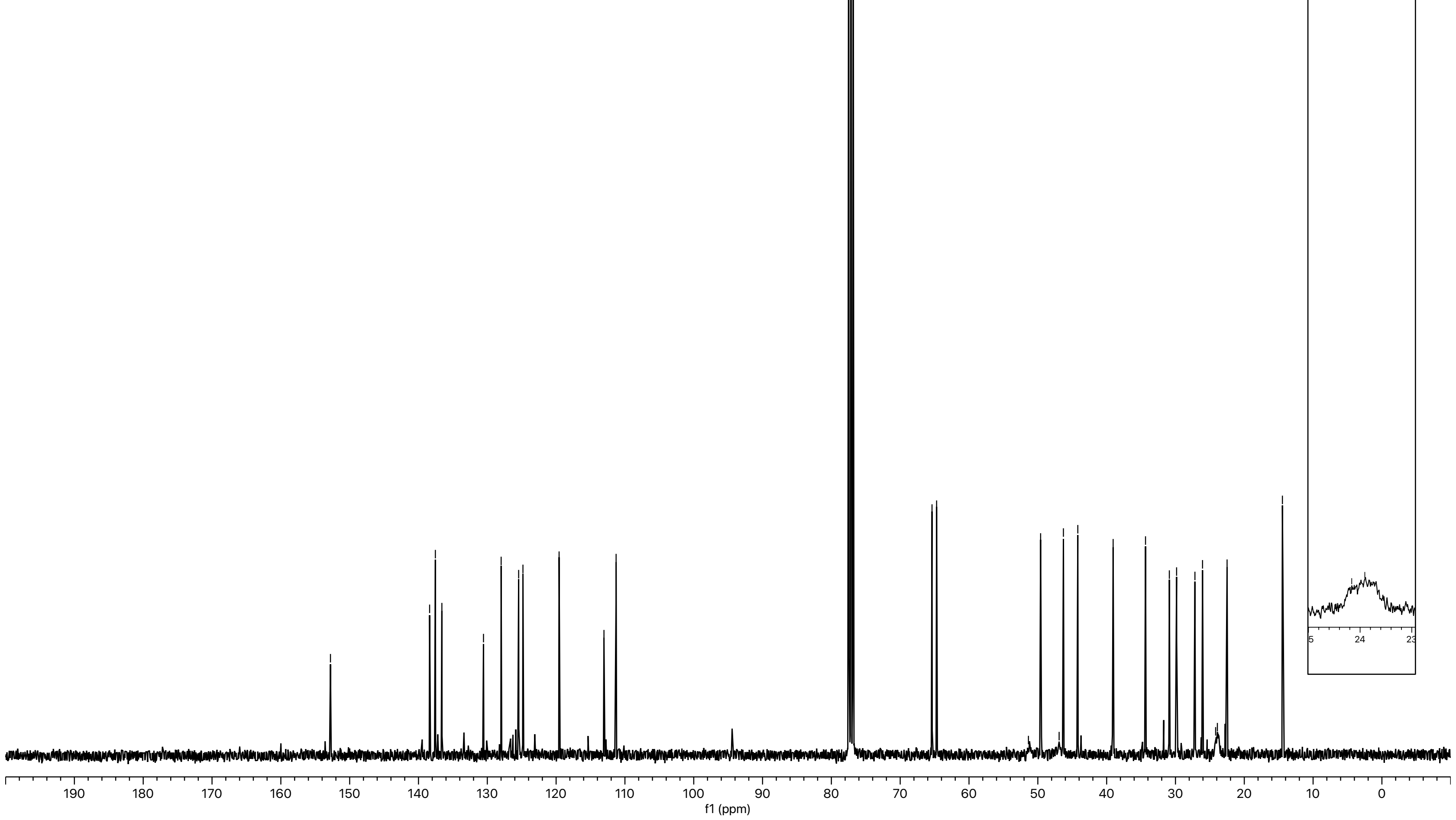

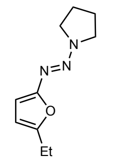

2-triazenyl-furan-11

^{1}H NMR

$CDCl_3$, 400 MHz

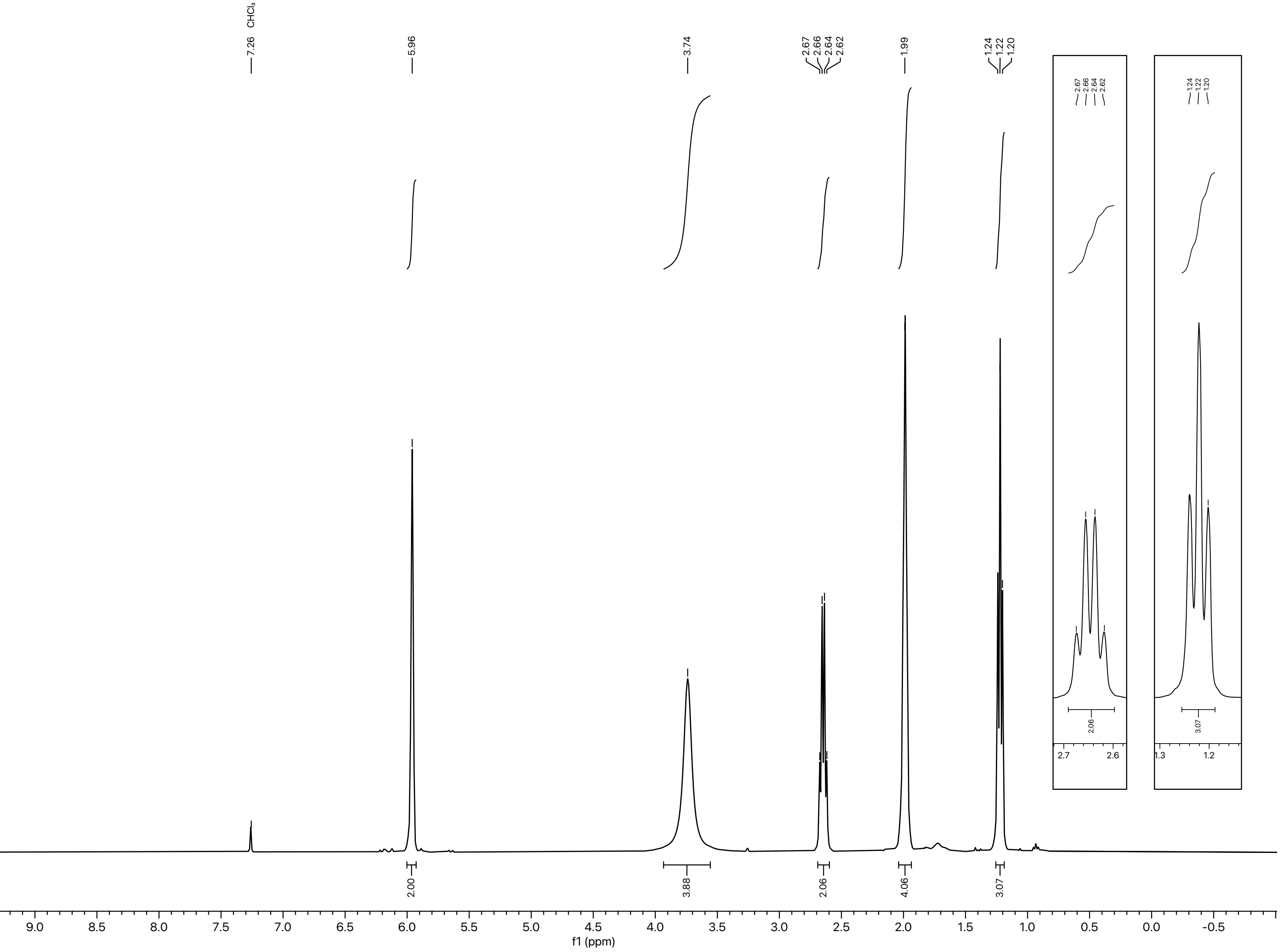

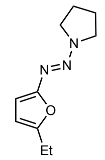

2-triazenyl-furan-11
^{13}C NMR
$CDCl_3$, 100 MHz

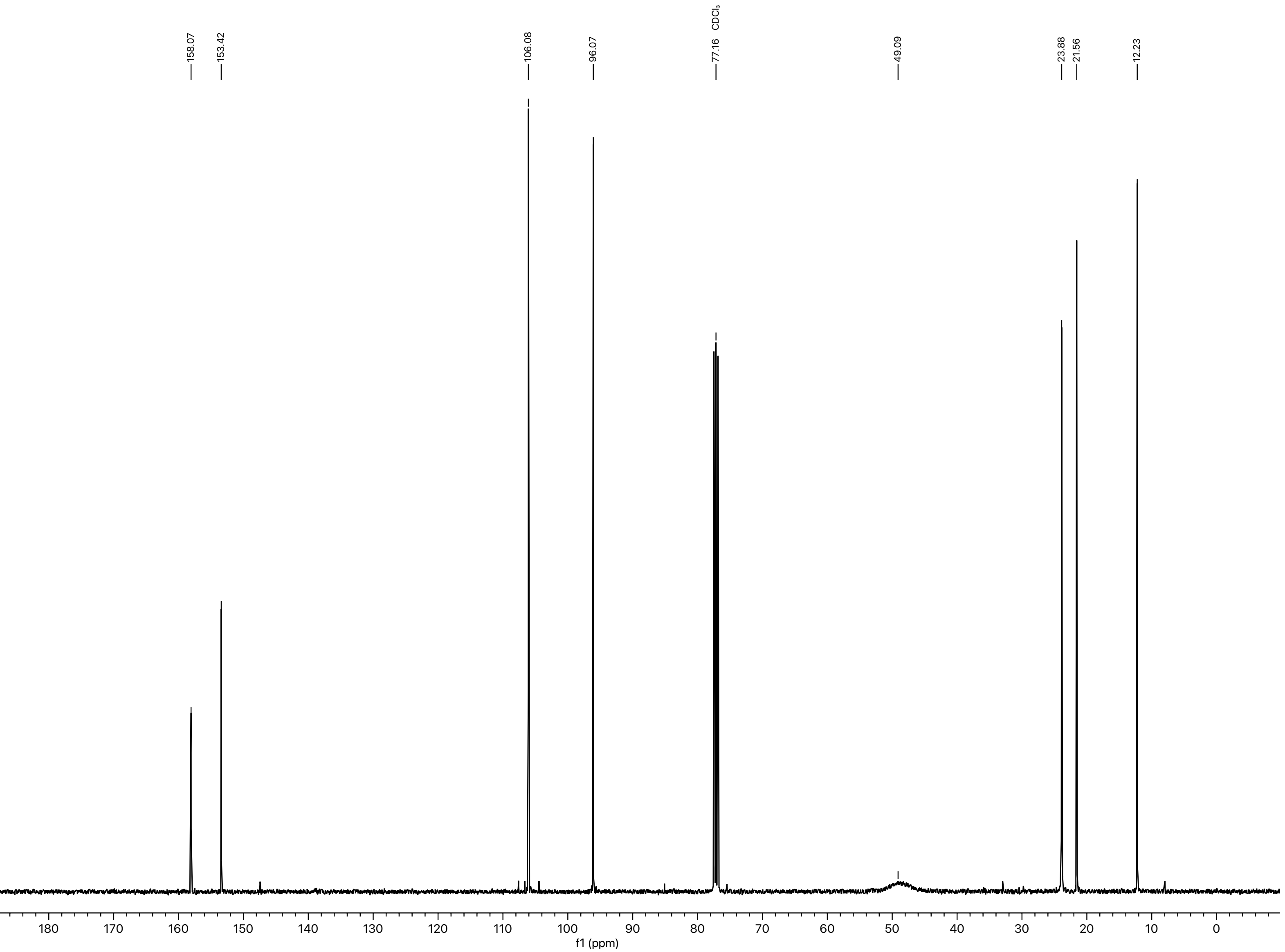

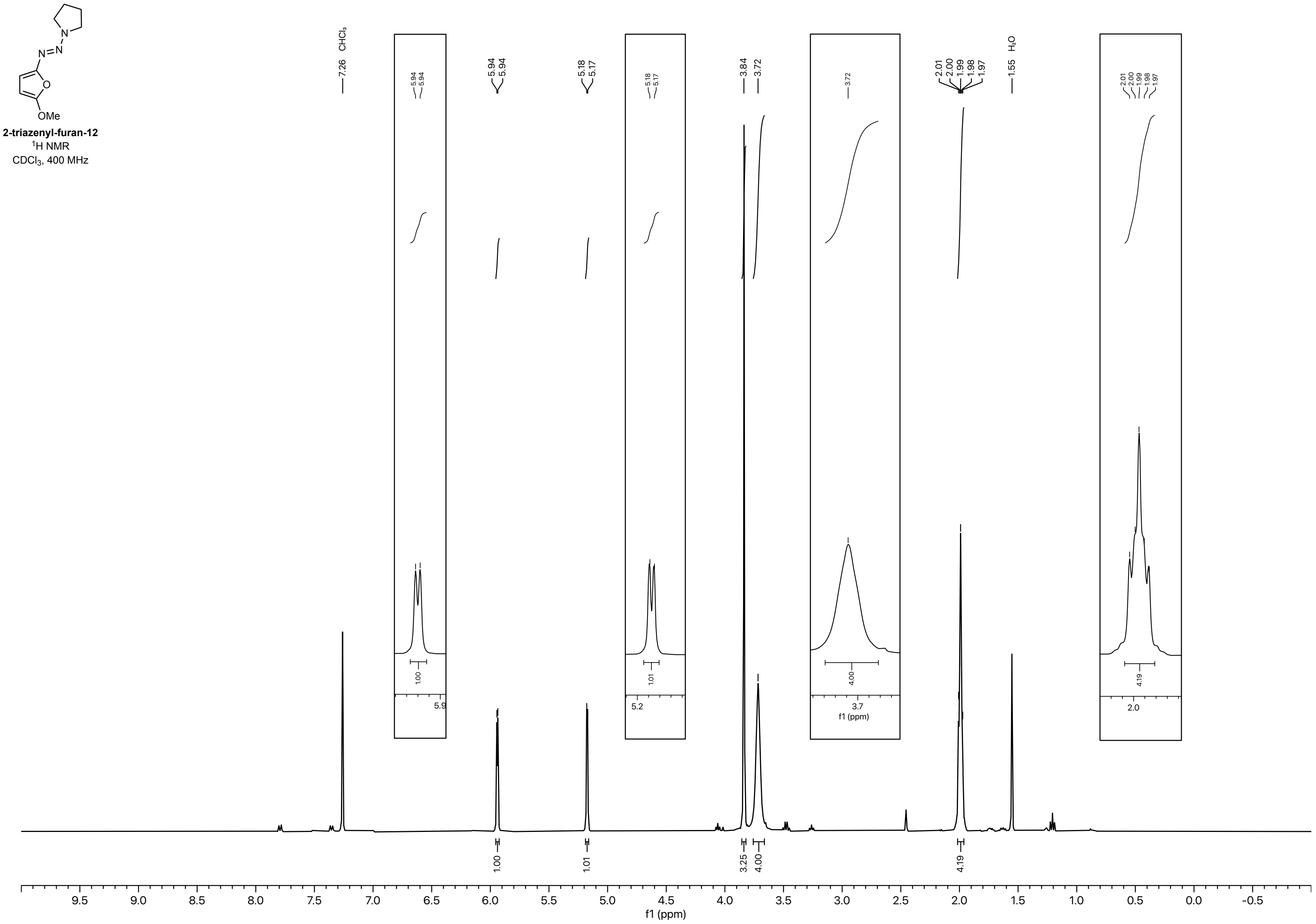

2-triazenyl-furan-12

^{1}H NMR
$CDCl_3$, 400 MHz

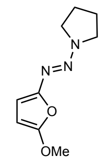

2-triazenyl-furan-12
^{13}C NMR
$CDCl_3$, 100 MHz

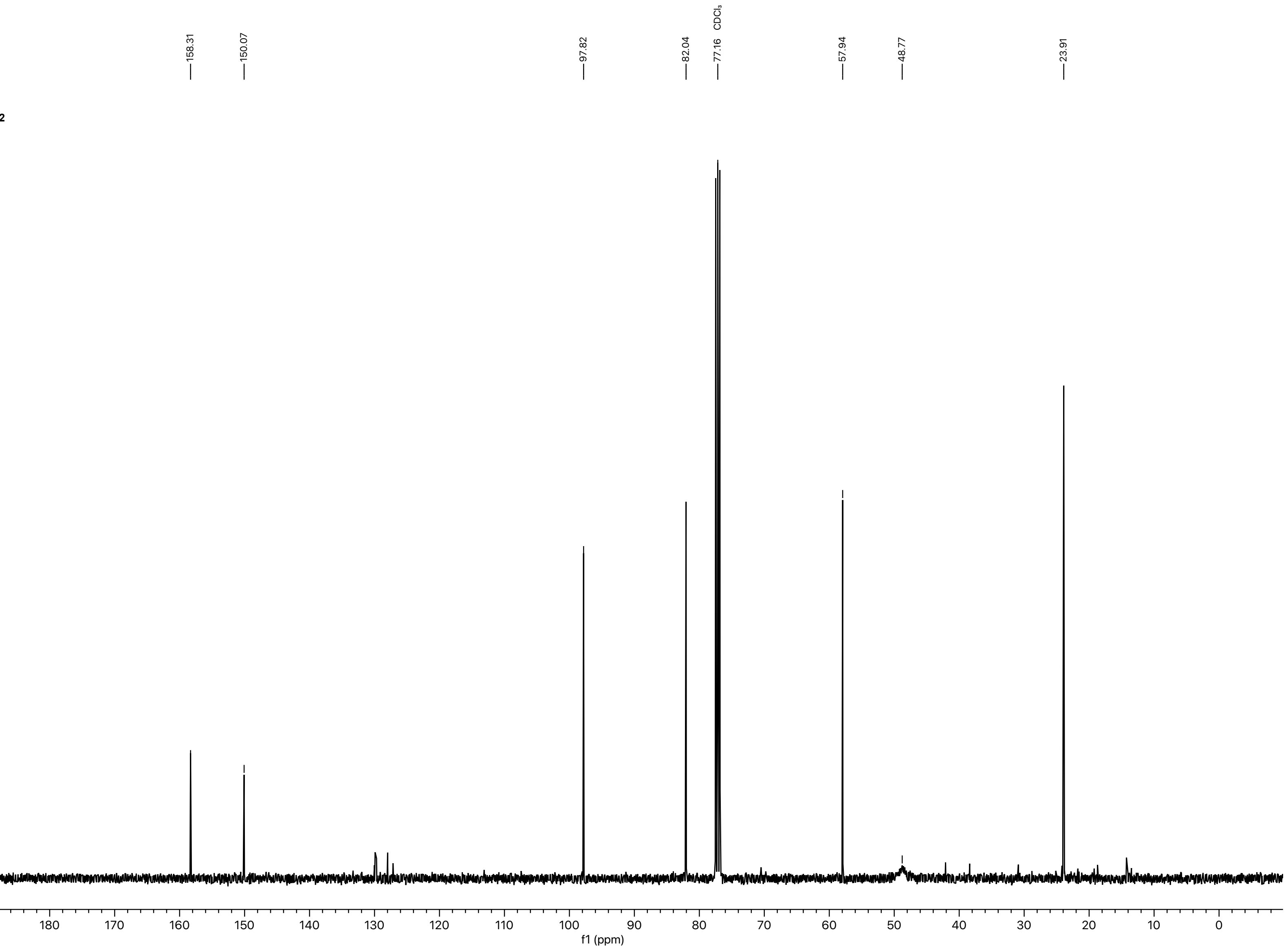

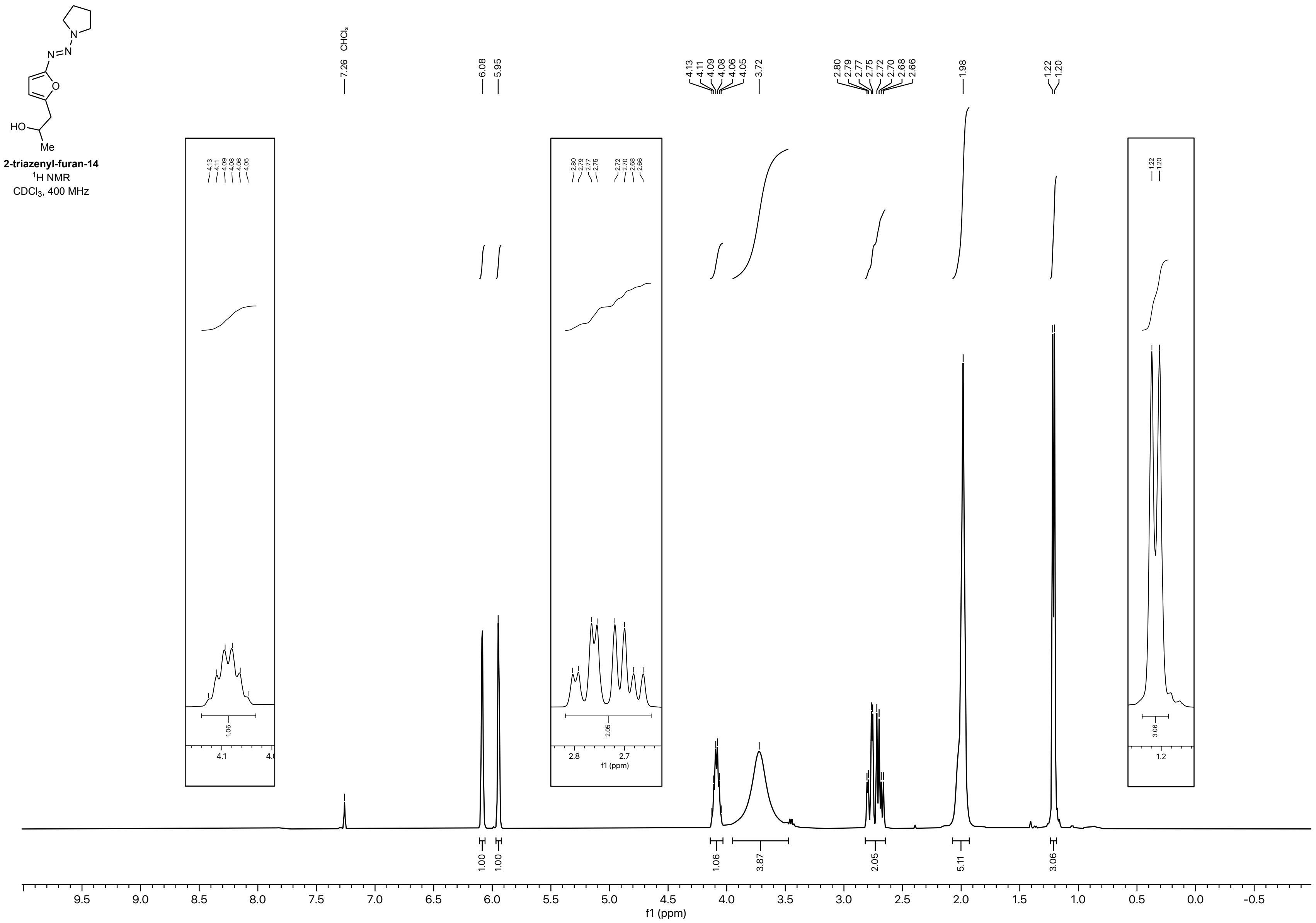

2-triazenyl-furan-14
^{1}H NMR
$CDCl_3$, 400 MHz

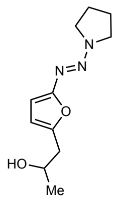

2-triazenyl-furan-14
^{13}C NMR
$CDCl_3$, 100 MHz

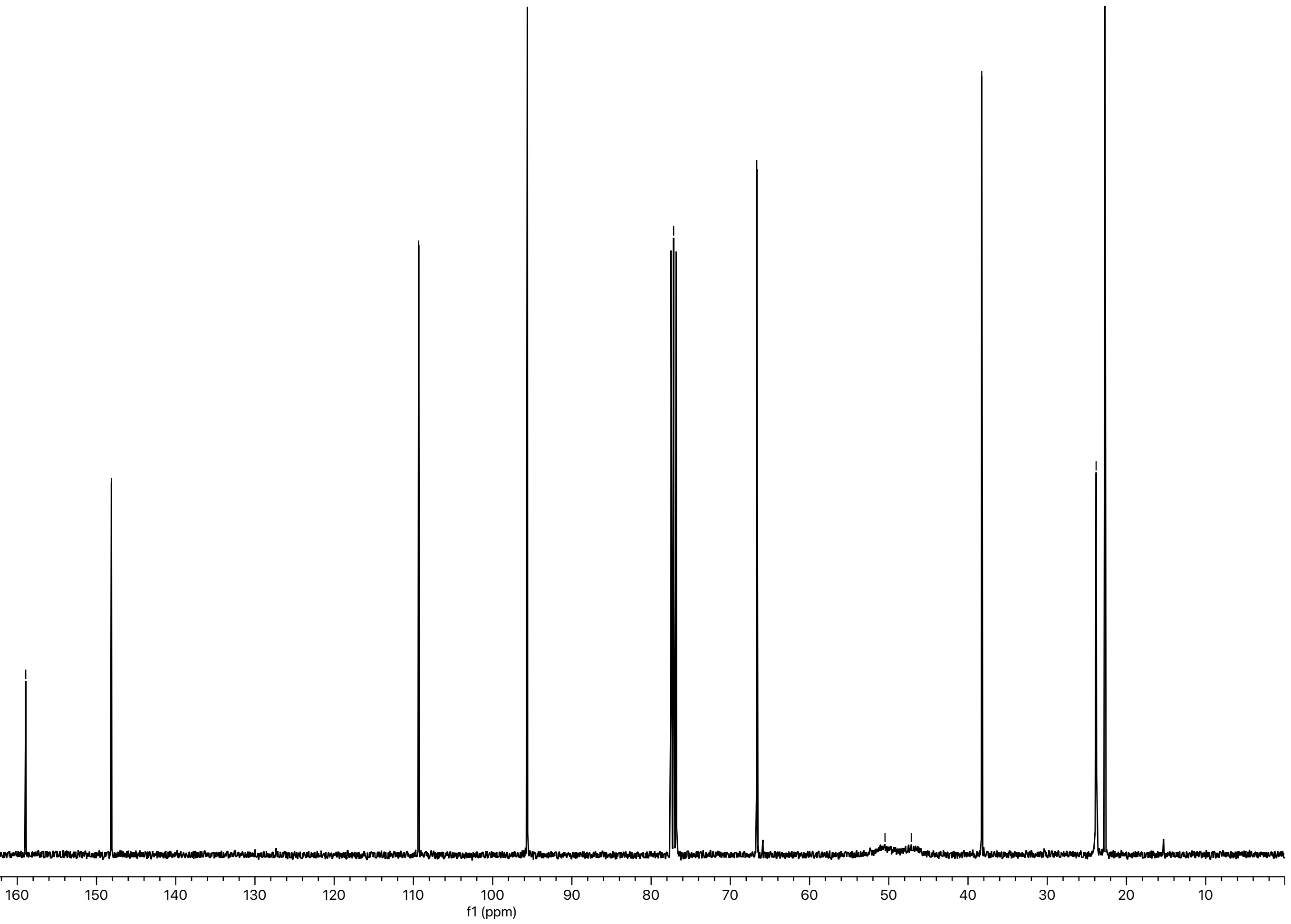

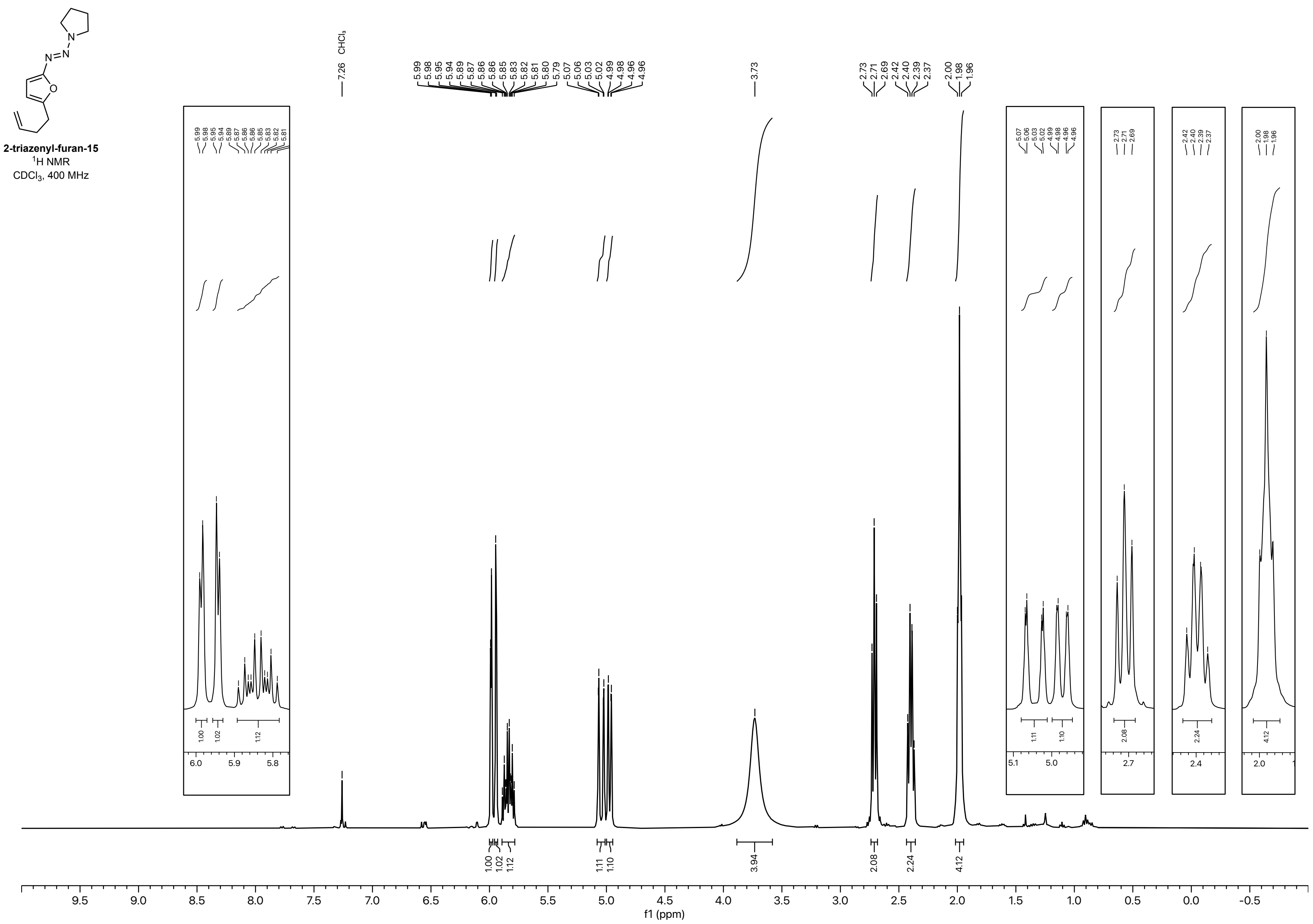

2-triazenyl-furan-15
1H NMR
CDCl3, 400 MHz
7.26 CHCl3
5.99 5.98 5.95 5.94 5.89 5.87 5.86 5.86 5.85 5.83 5.82 5.81 5.80 5.79 5.07 5.06 5.03 5.02 4.99 4.98 4.96 4.96
3.73
2.73 2.71 2.69 2.42 2.40 2.39 2.37 2.00 1.98 1.96
1.00 1.02 1.12 1.11 1.10 3.94 2.08 2.24 4.12
9.5 9.0 8.5 8.0 7.5 7.0 6.5 6.0 5.5 5.0 4.5 4.0 3.5 3.0 2.5 2.0 1.5 1.0 0.5 0.0 -0.5
f1 (ppm)

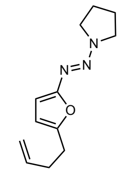

2-triazenyl-furan-15
^{13}C NMR
$CDCl_3$, 100 MHz

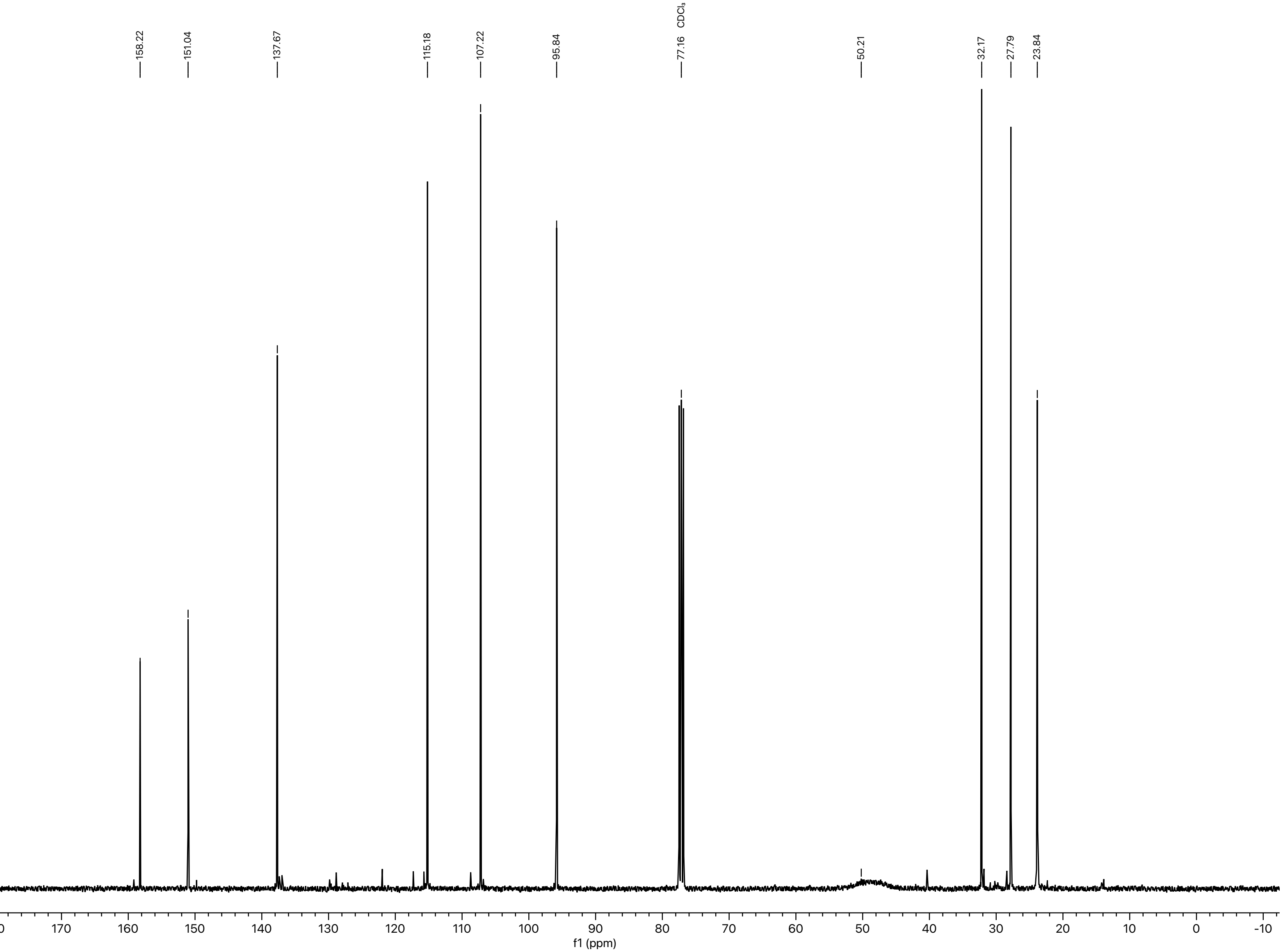

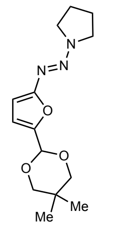

2-triazenyl-furan-16
^1H NMR
$CDCl_3$, 400 MHz

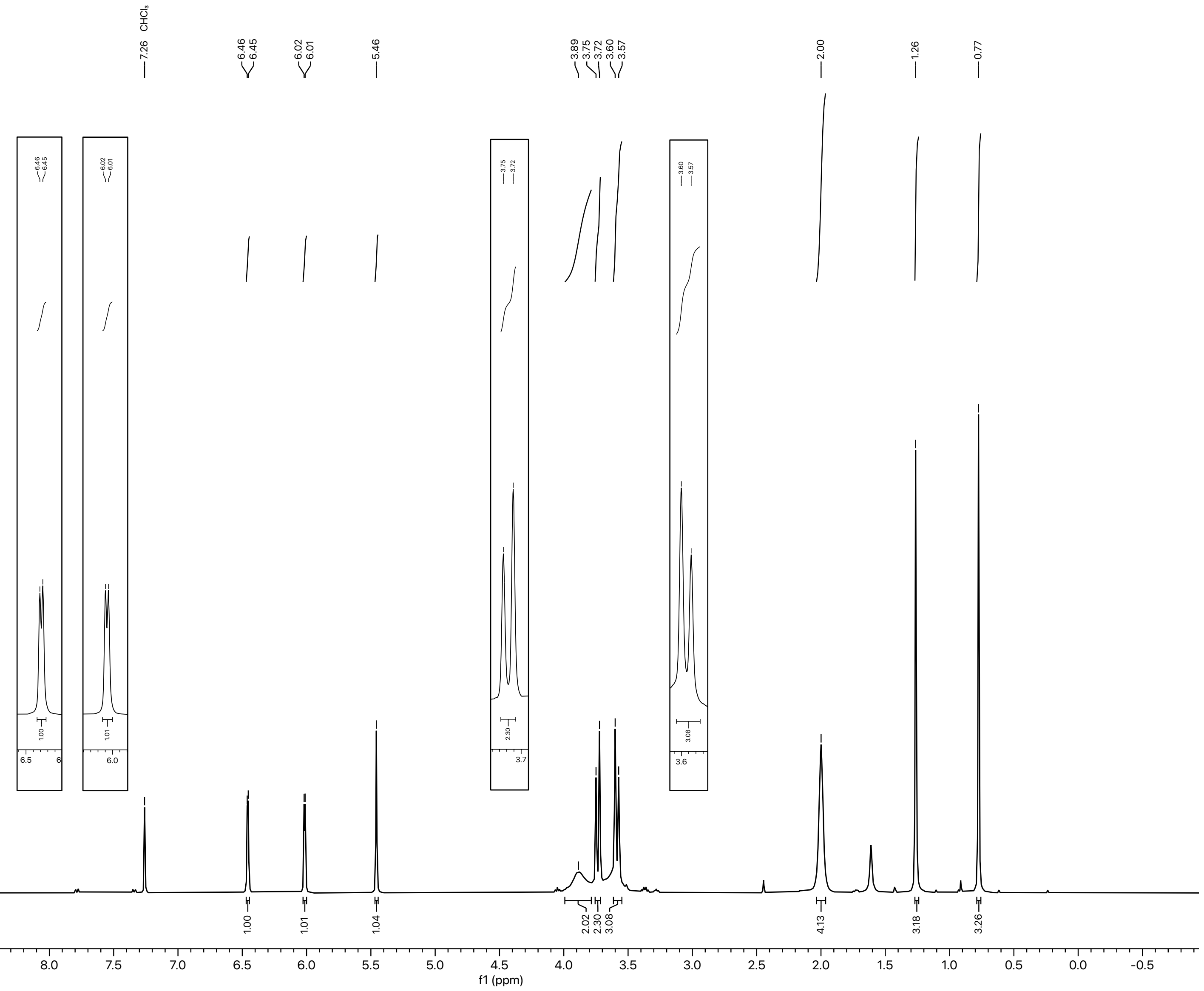

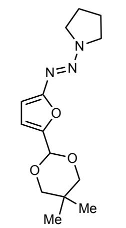

2-triazenyl-furan-16
^{13}C NMR
$CDCl_3$, 100 MHz

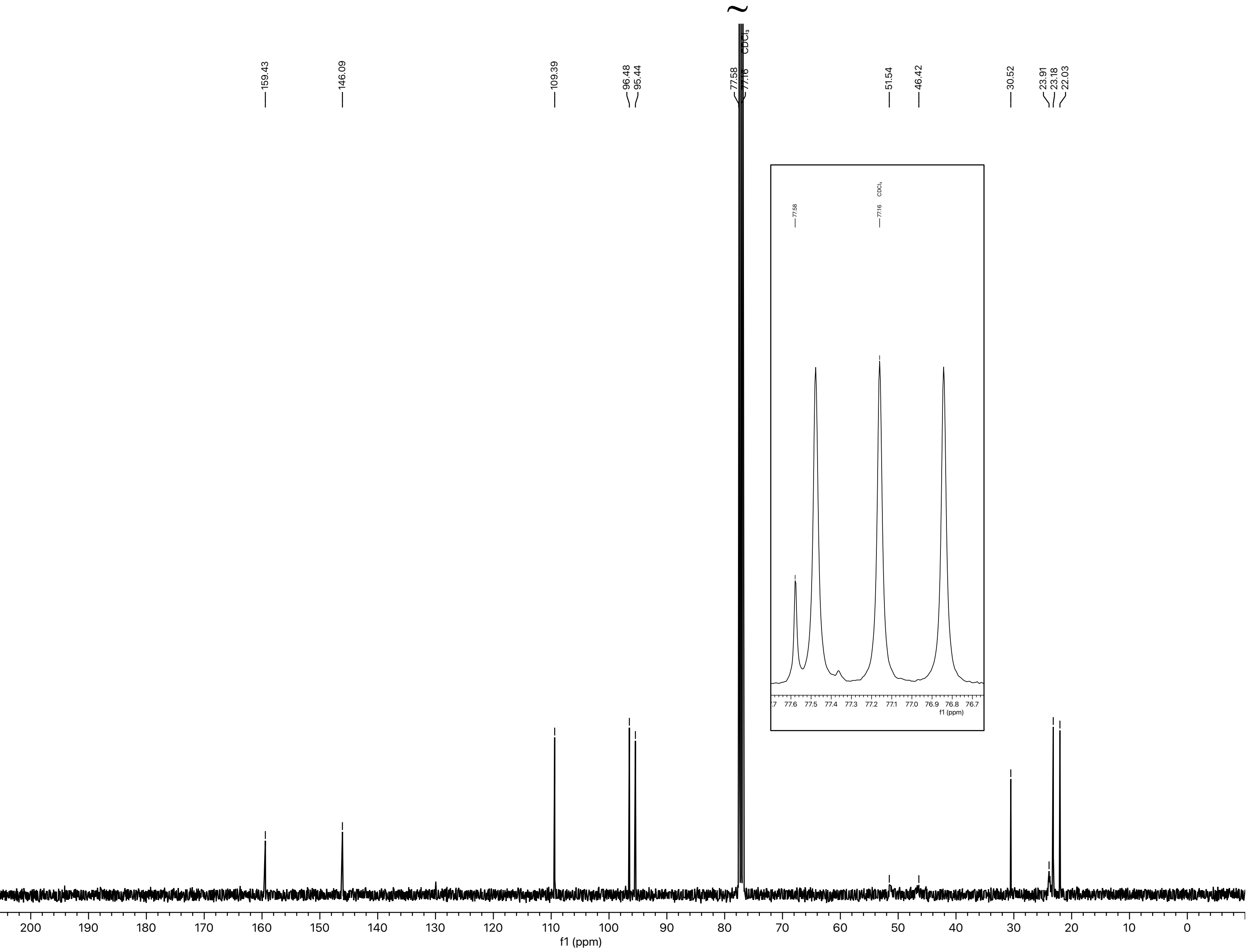

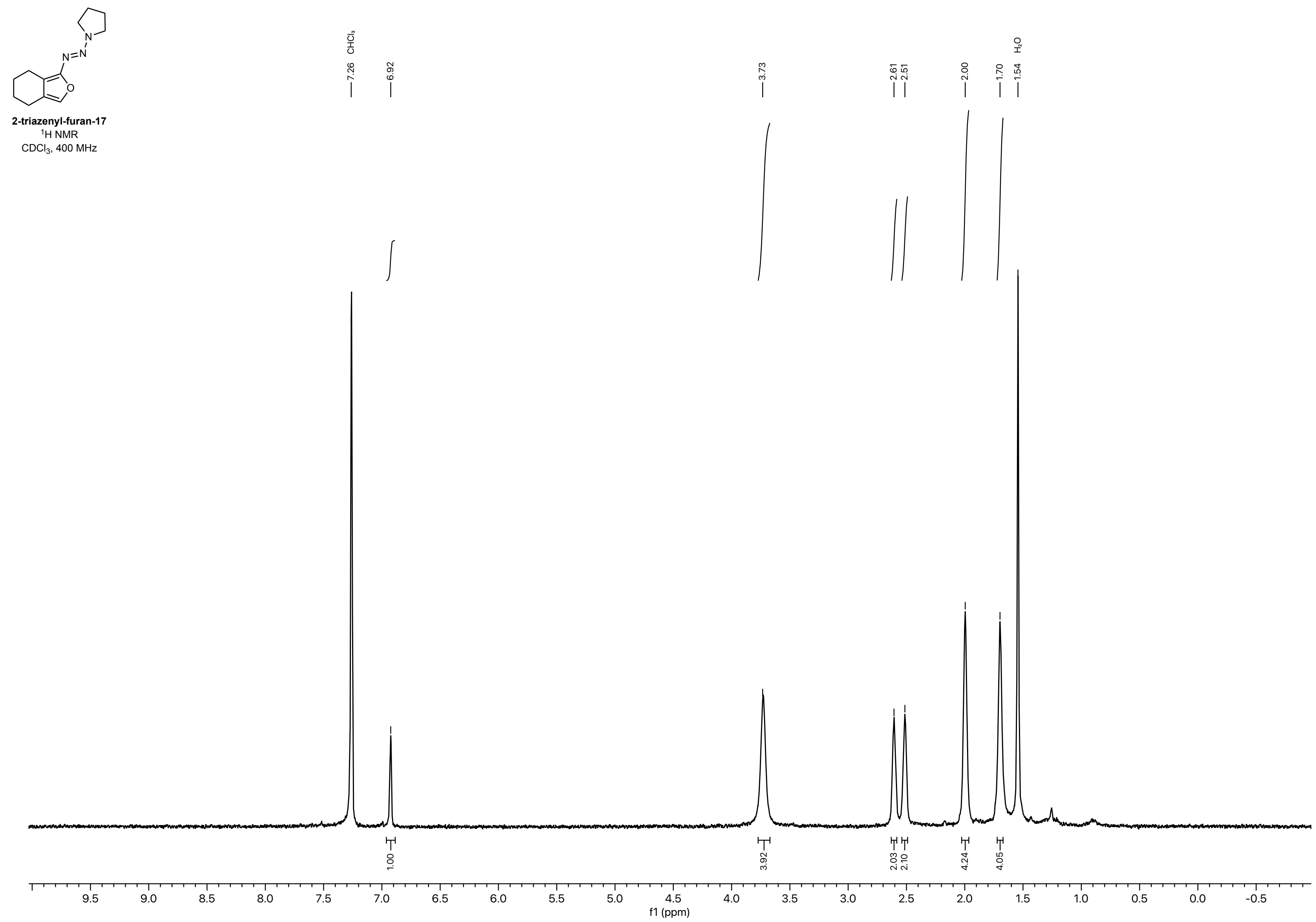

2-triazenyl-furan-17
¹H NMR
CDCl3, 400 MHz
7.26 CHCl3
6.92
3.73
2.61
2.51
2.00
1.70
1.54 H2O
1.00
3.92
2.03
2.10
4.24
4.05
9.5
9.0
8.5
8.0
7.5
7.0
6.5
6.0
5.5
5.0
4.5
4.0
3.5
3.0
2.5
2.0
1.5
1.0
0.5
0.0
-0.5
f1 (ppm)

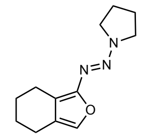

2-triazenyl-furan-17
^{13}C NMR
$CDCl_3$, 100 MHz

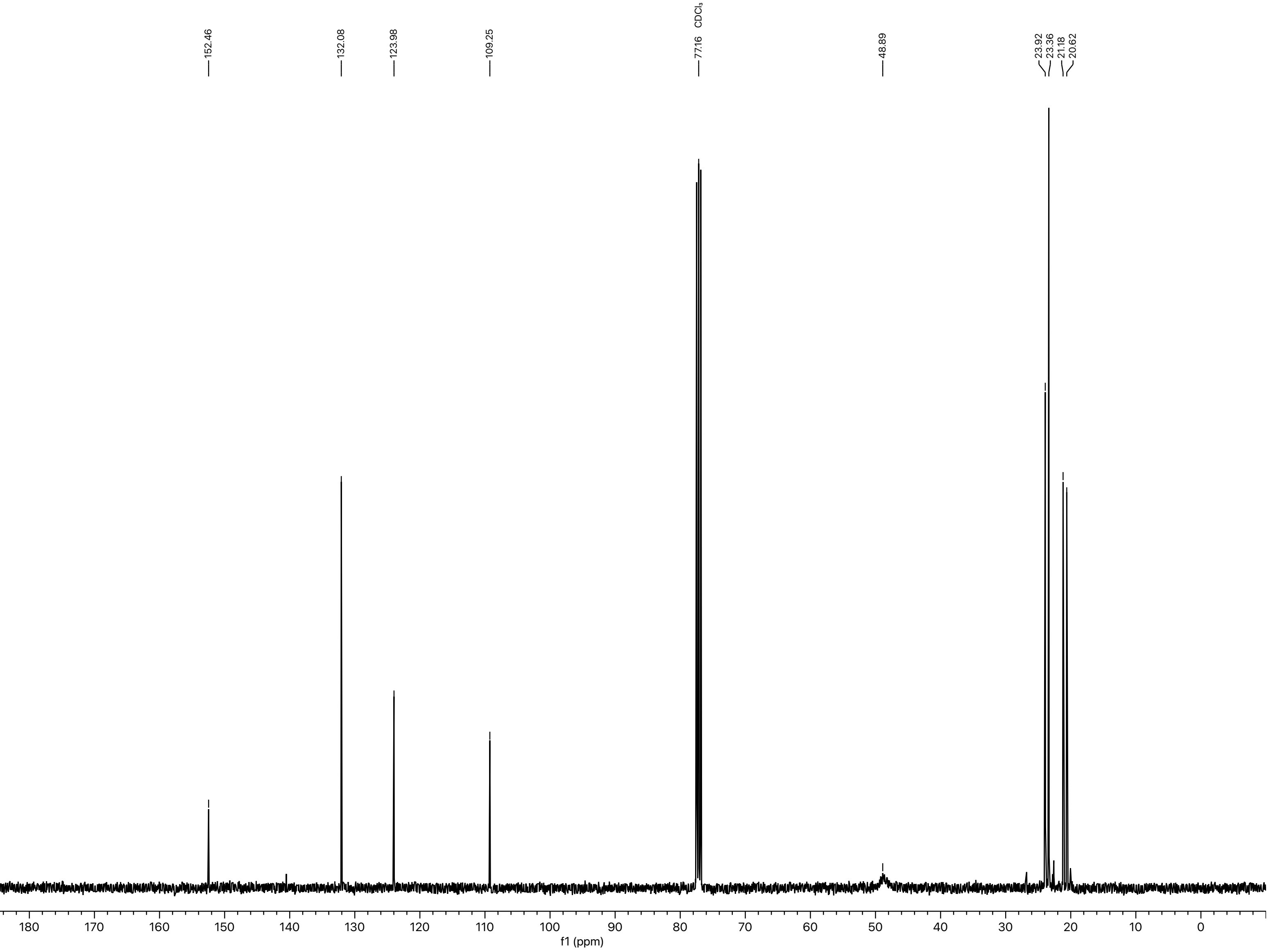

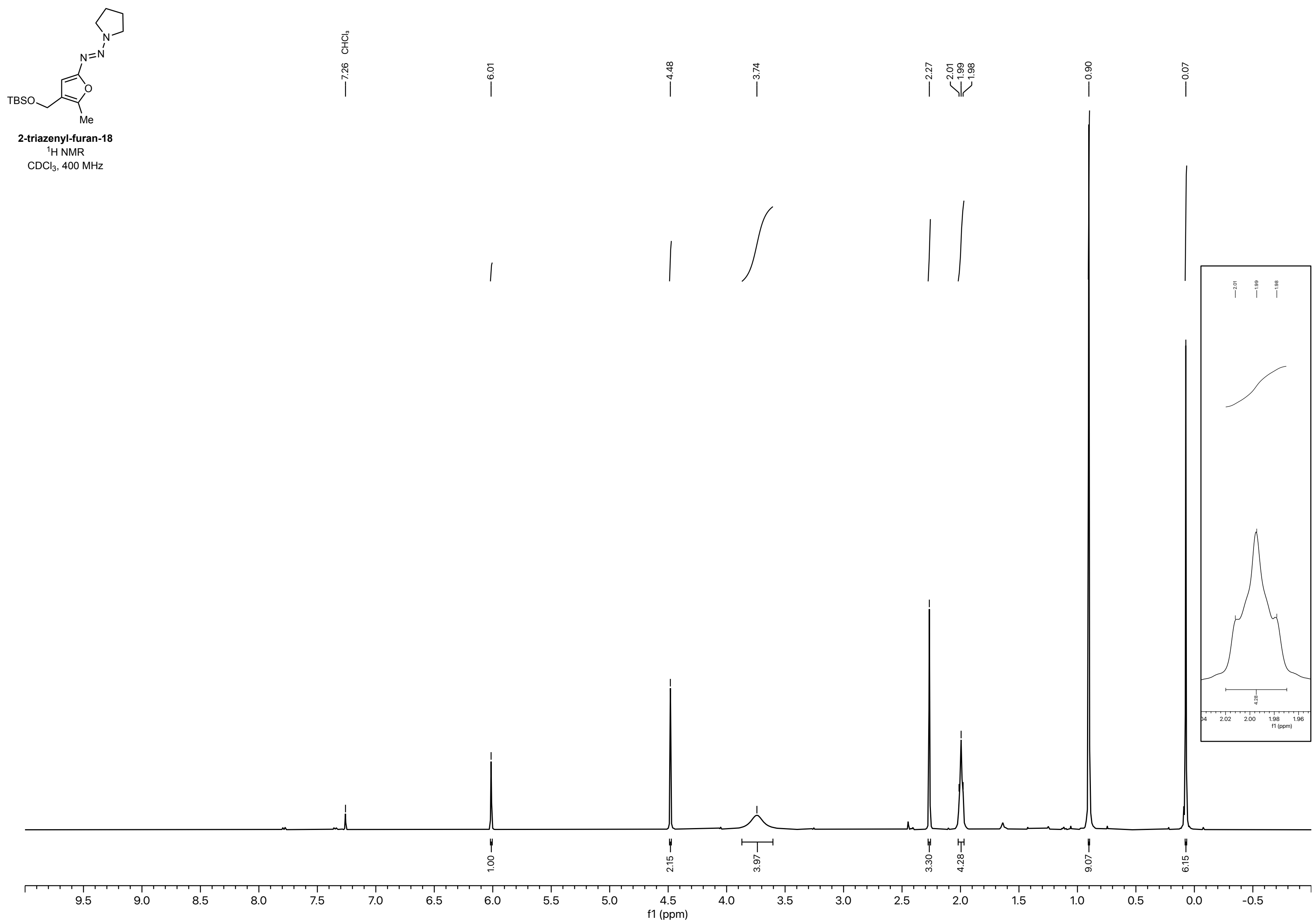

TBSO
N=N
N
O
Me
2-triazenyl-furan-18
^{1}H NMR
$CDCl_3$, 400 MHz
7.26 $CHCl_3$
6.01
4.48
3.74
2.27
2.01
1.99
1.98
0.90
0.07
1.00
2.15
3.97
3.30
4.28
9.07
6.15
f1 (ppm)

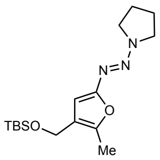

2-triazenyl-furan-18
^{13}C NMR
$CDCl_3$, 100 MHz

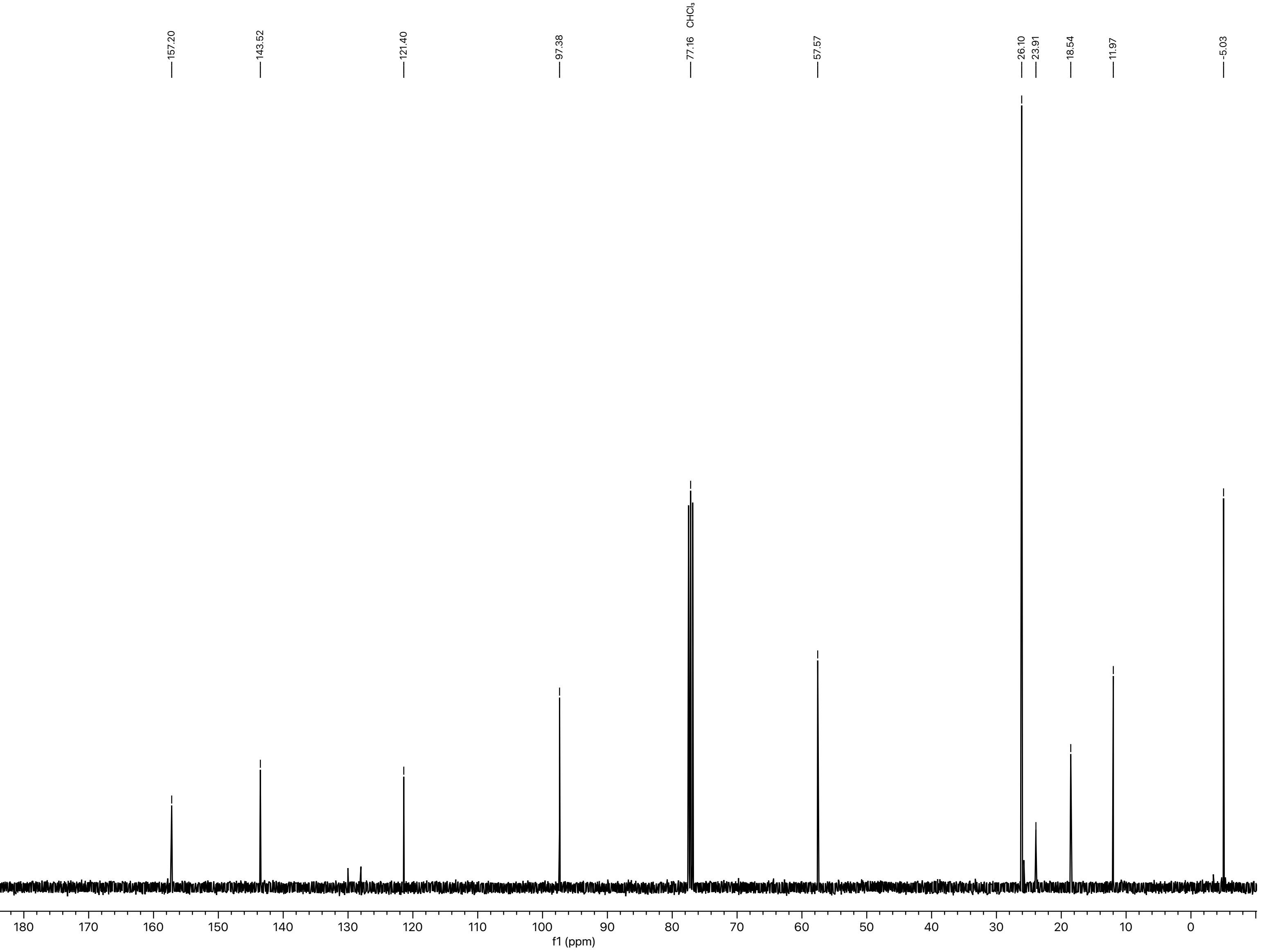

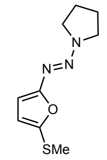

2-triazenyl-furan-19
^1H NMR
$CDCl_3$, 400 MHz

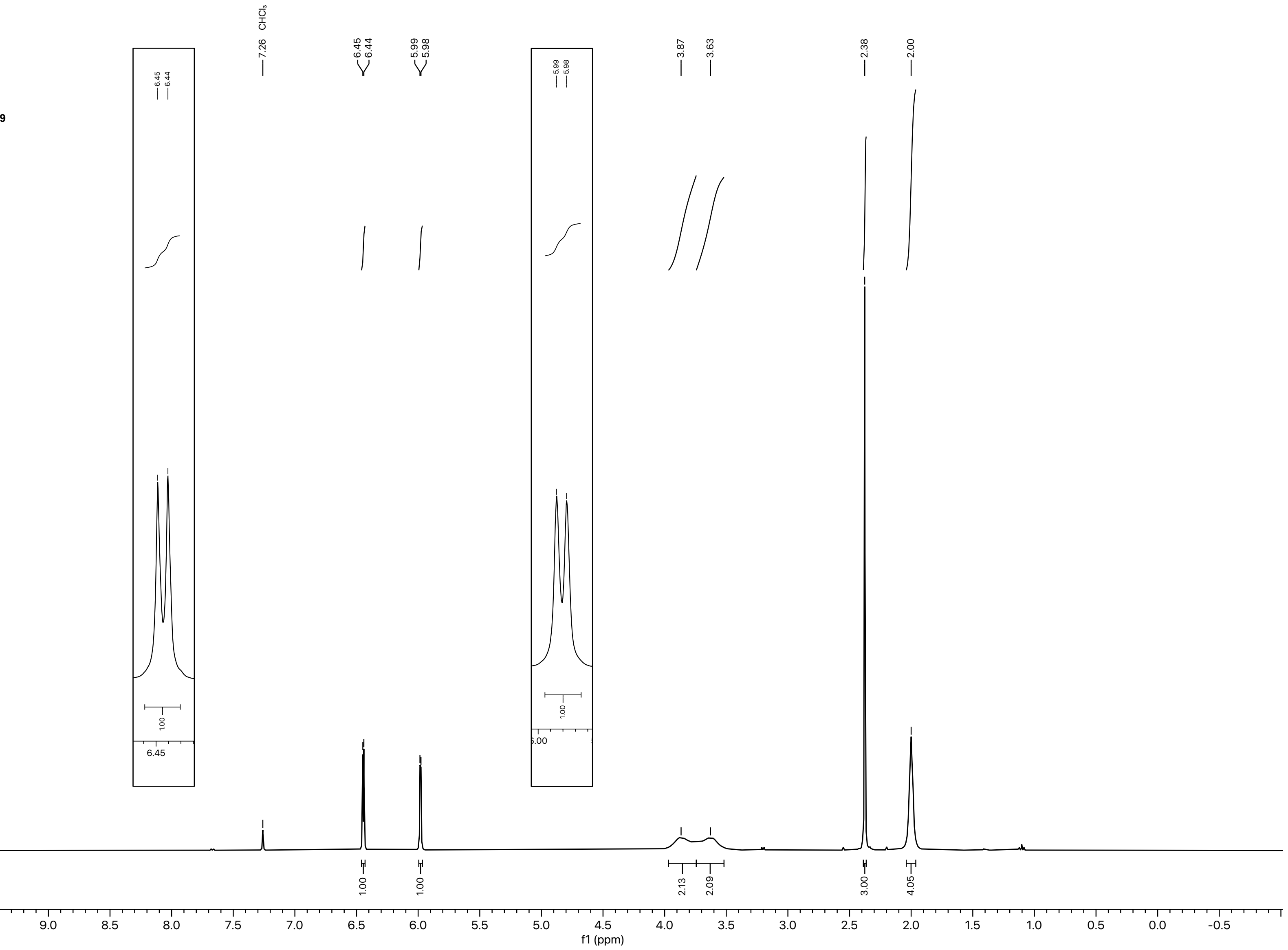

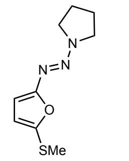

2-triazenyl-furan-19
^{13}C NMR
$CDCl_3$, 100 MHz

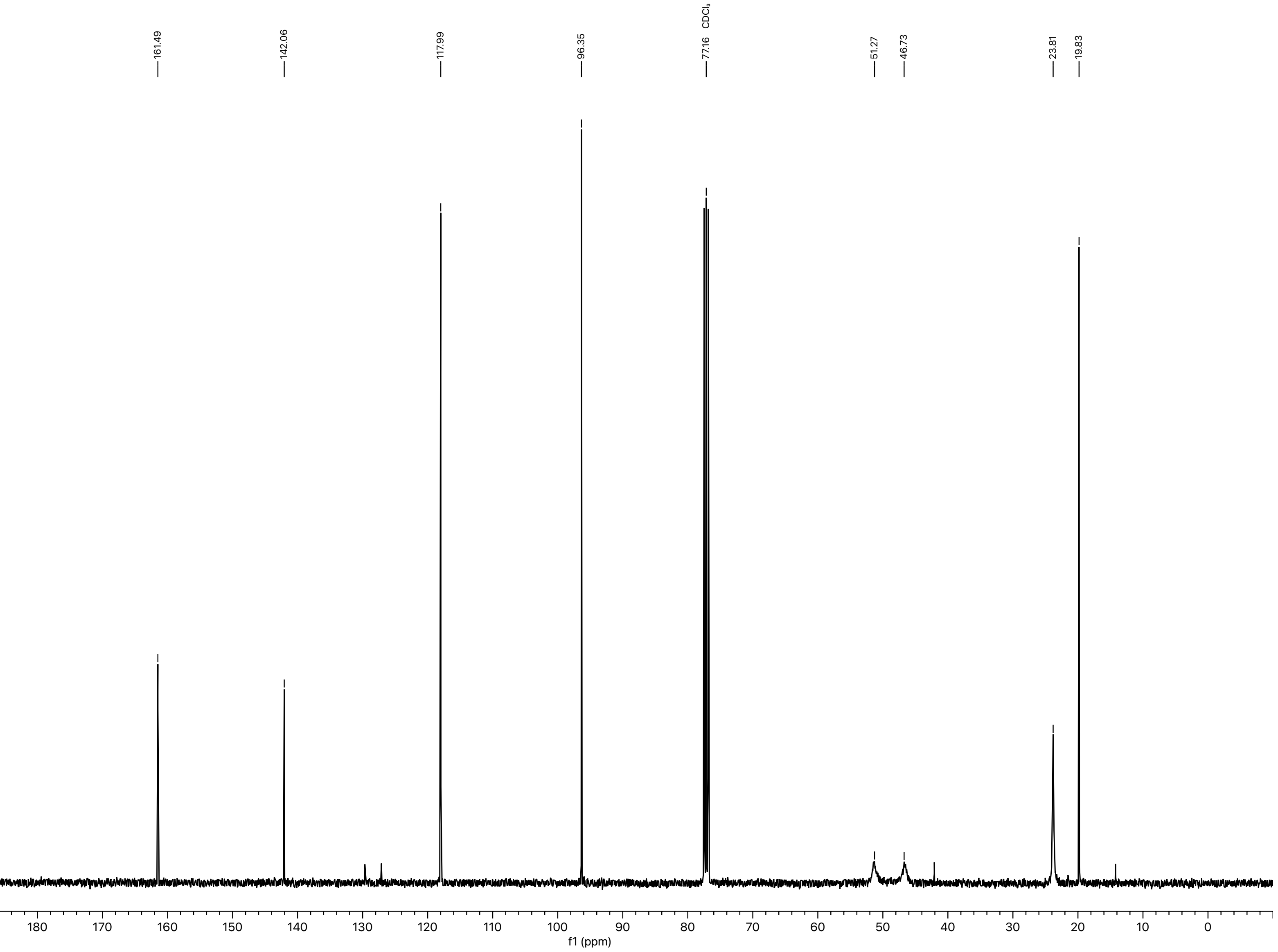

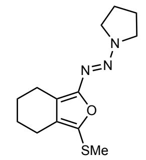

2-triazenyl-furan-20
^{1}H NMR
$CDCl_3$, 400 MHz

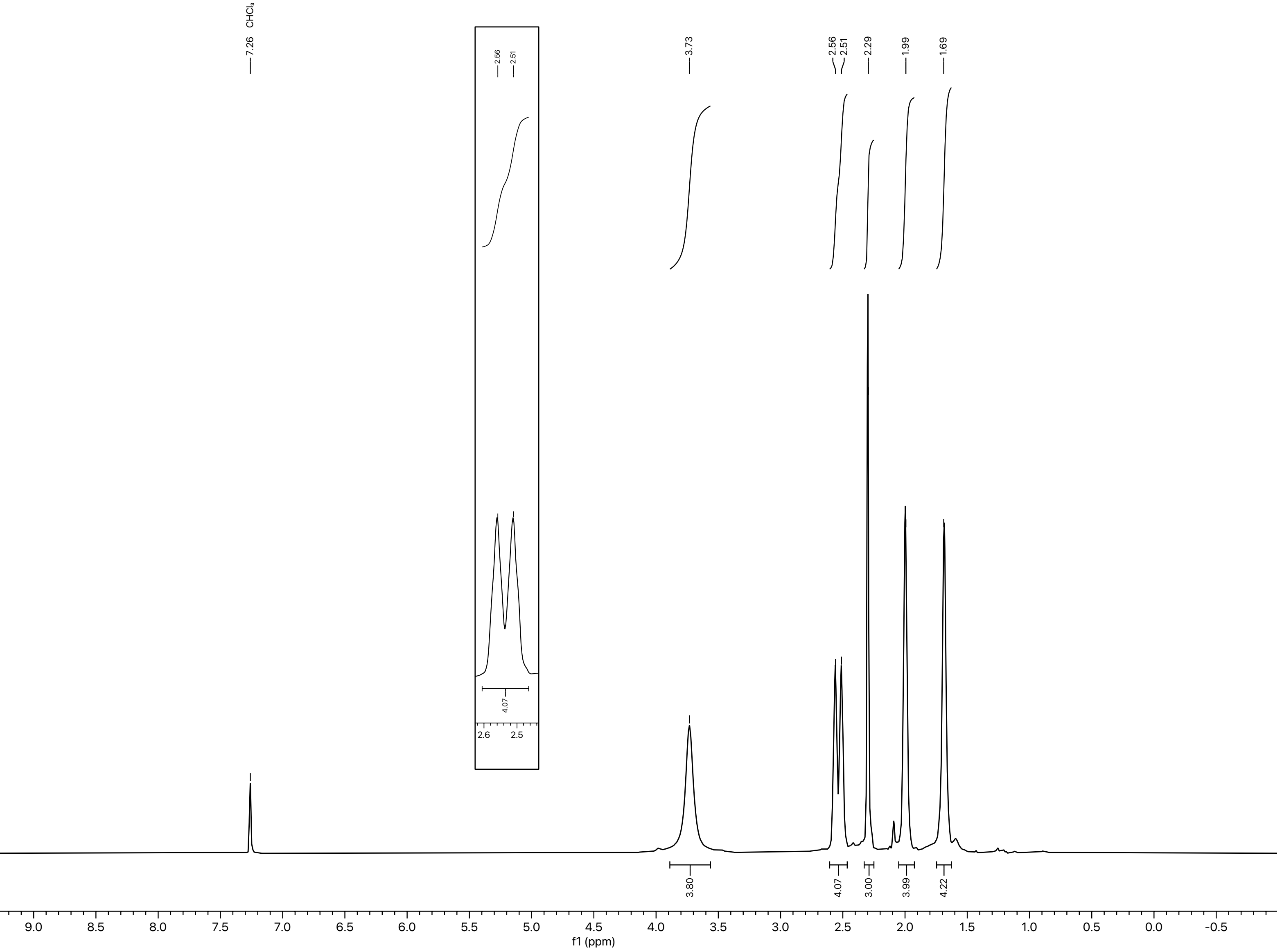

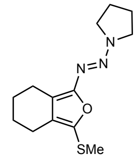

2-triazenyl-furan-20
^{13}C NMR
$CDCl_3$, 100 MHz

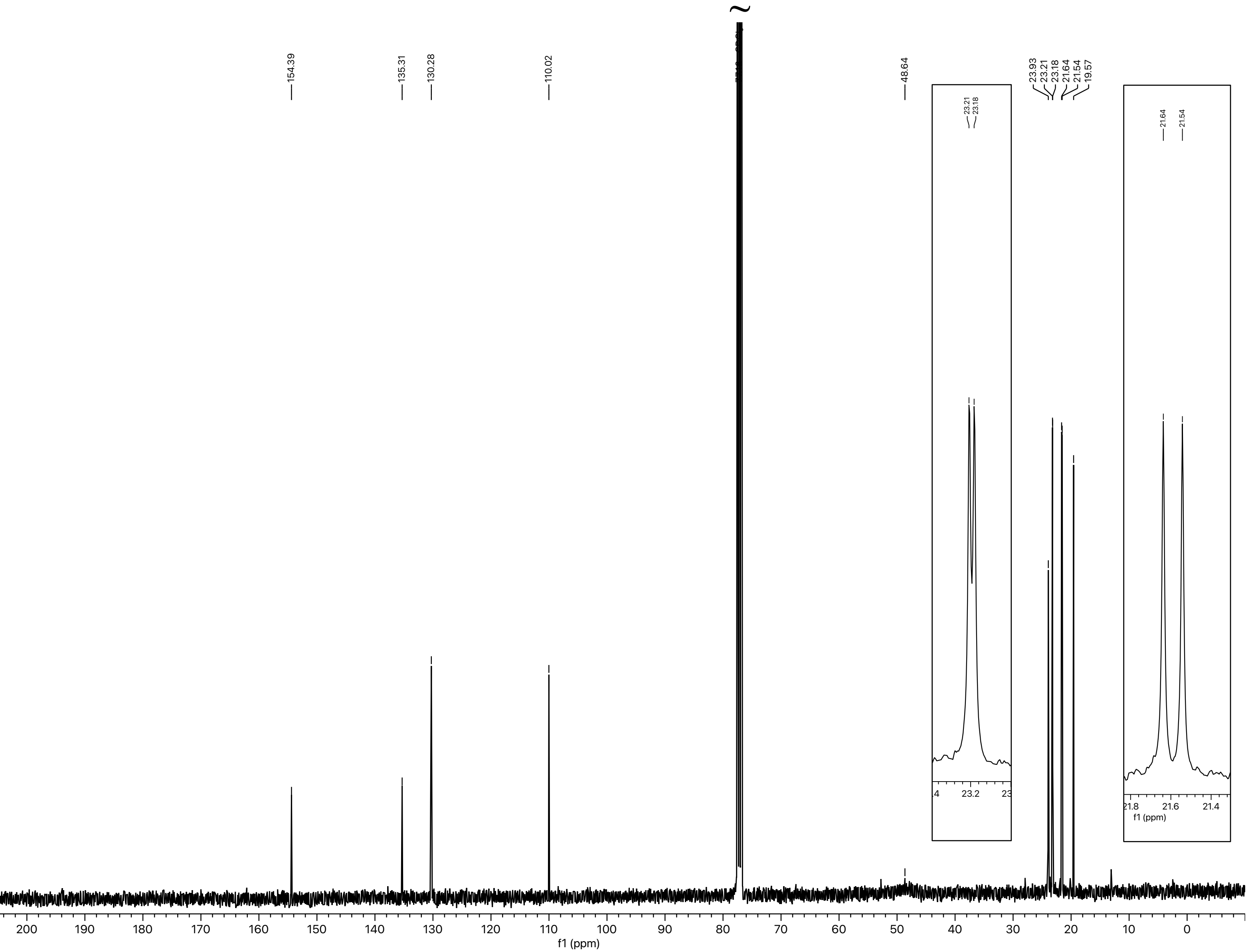

Derivatization of 2-Triazenyl Furans

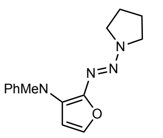

2-triazenyl-furan-21 (or **3**)
^{1}H NMR
$CDCl_3$, 400 MHz

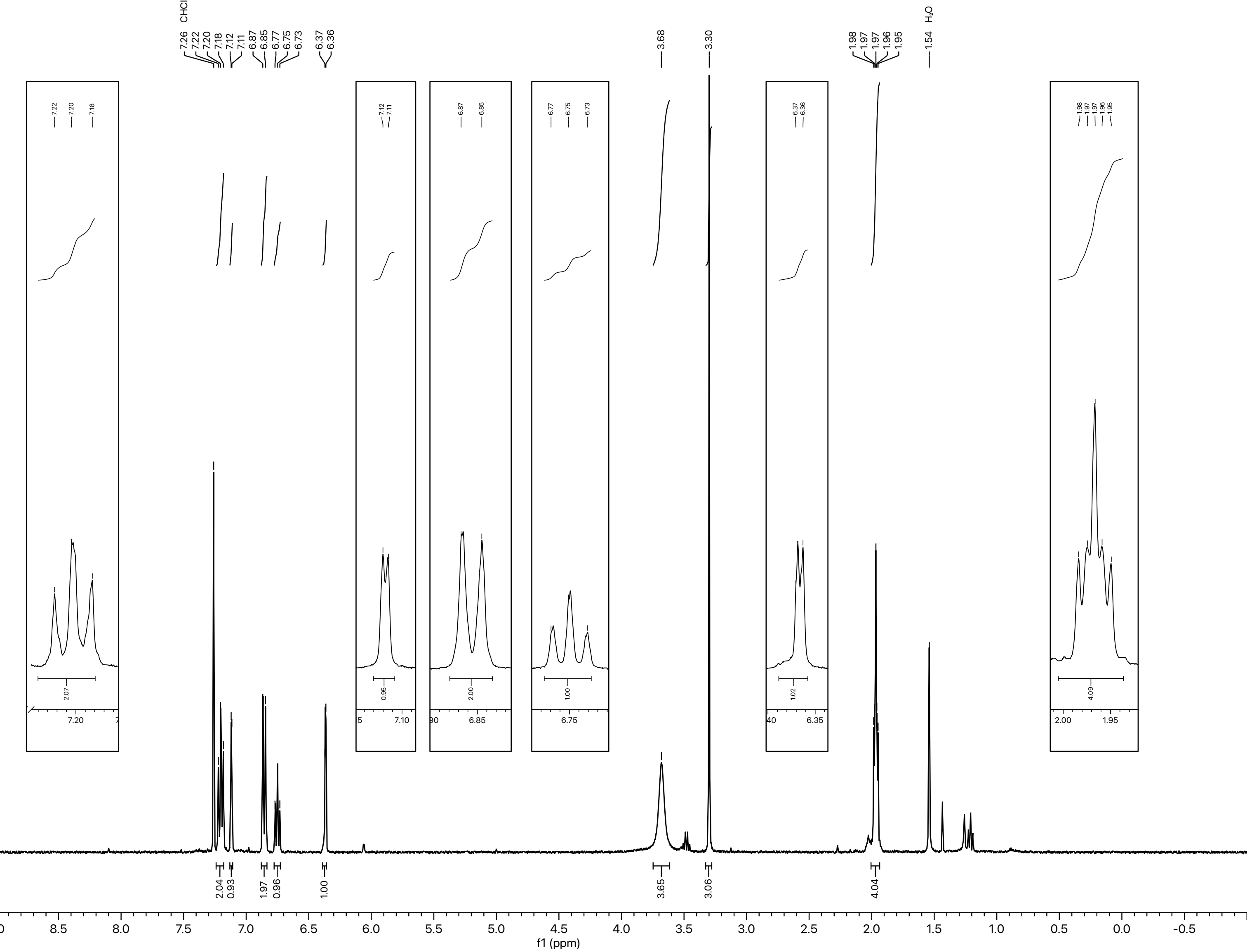

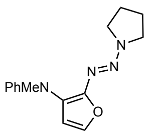

2-triazenyl-furan-21 (or **3**)
^{13}C NMR
$CDCl_3$, 100 MHz

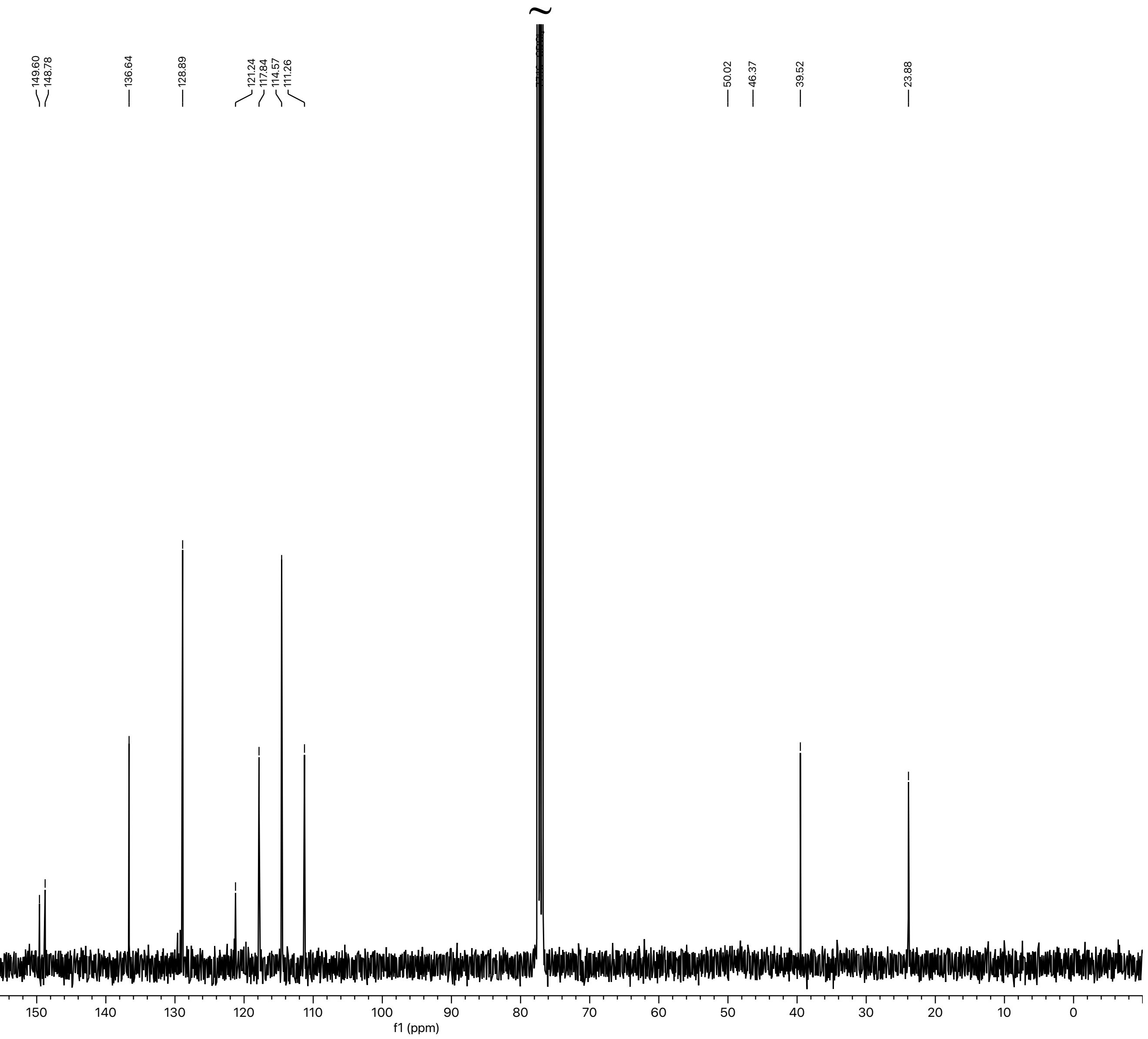

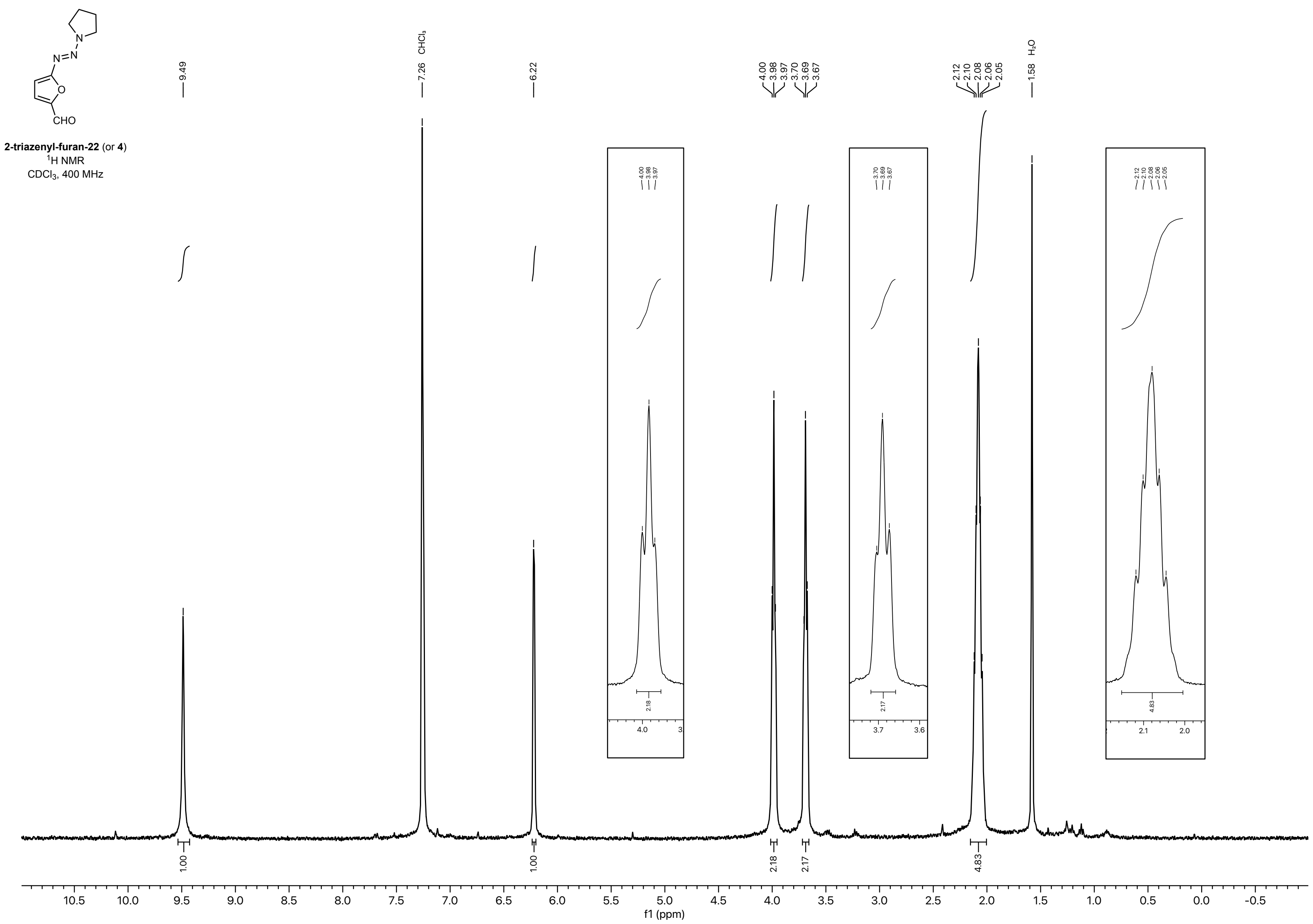

2-triazenyl-furan-22 (or **4**)
^{1}H NMR
$CDCl_3$, 400 MHz

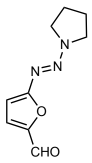

2-triazenyl-furan-22 (or **4**)
^{13}C NMR
$CDCl_3$, 100 MHz

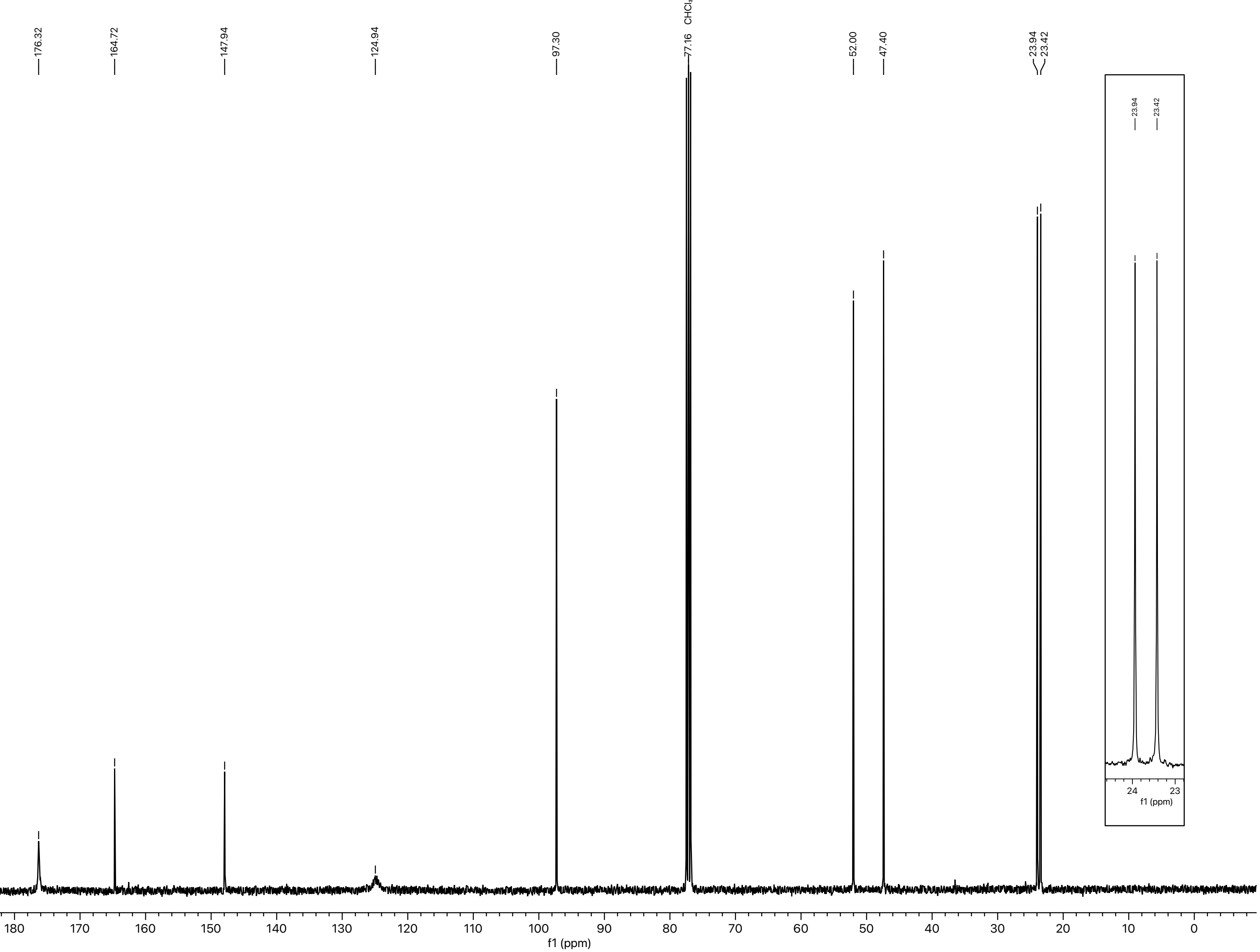

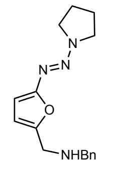

2-triazenyl-furan-23 (or **5**)
^{1}H NMR
$CDCl_3$, 400 MHz

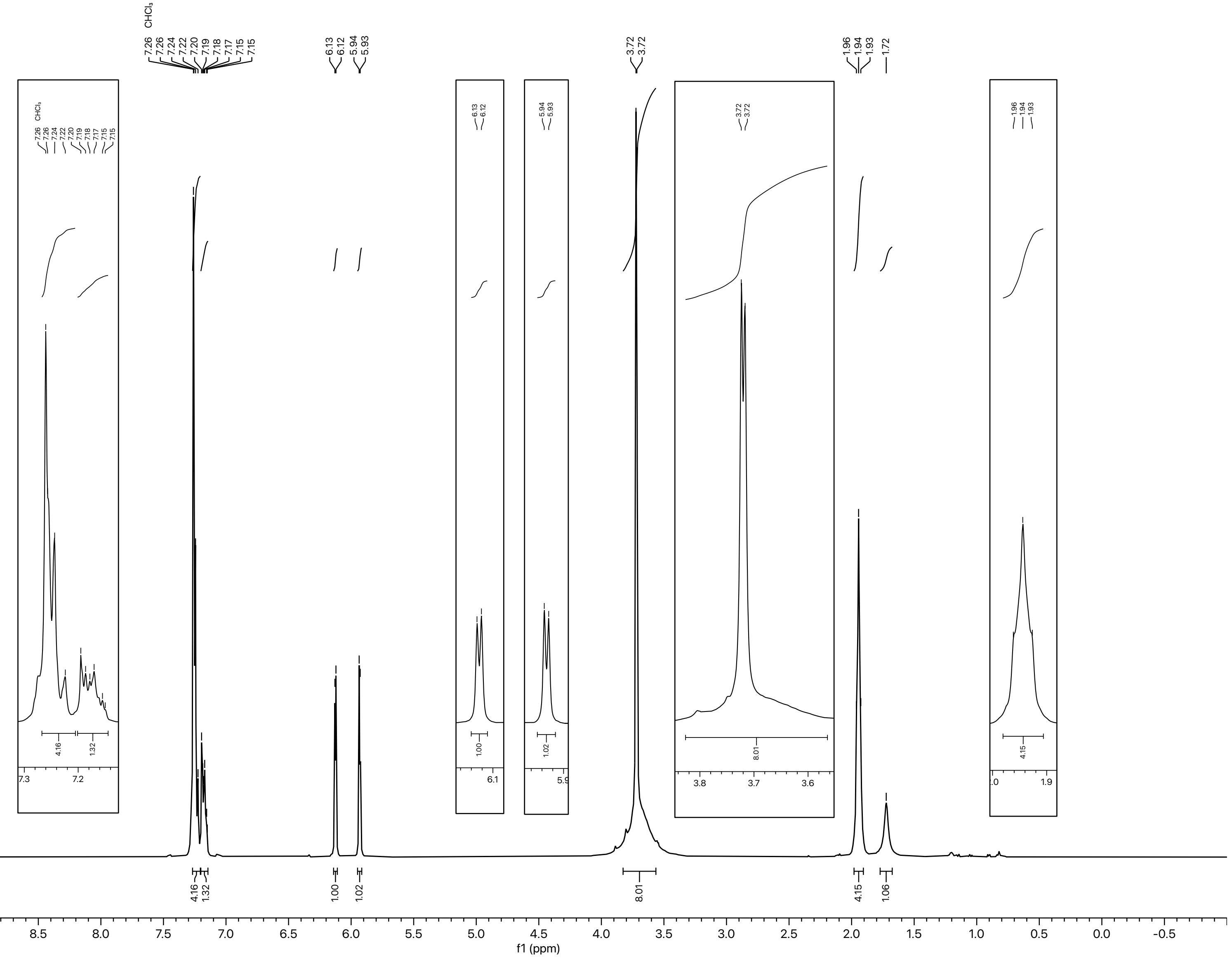

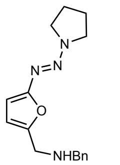

2-triazenyl-furan-23 (or **5**)
^{13}C NMR
$CDCl_3$, 100 MHz

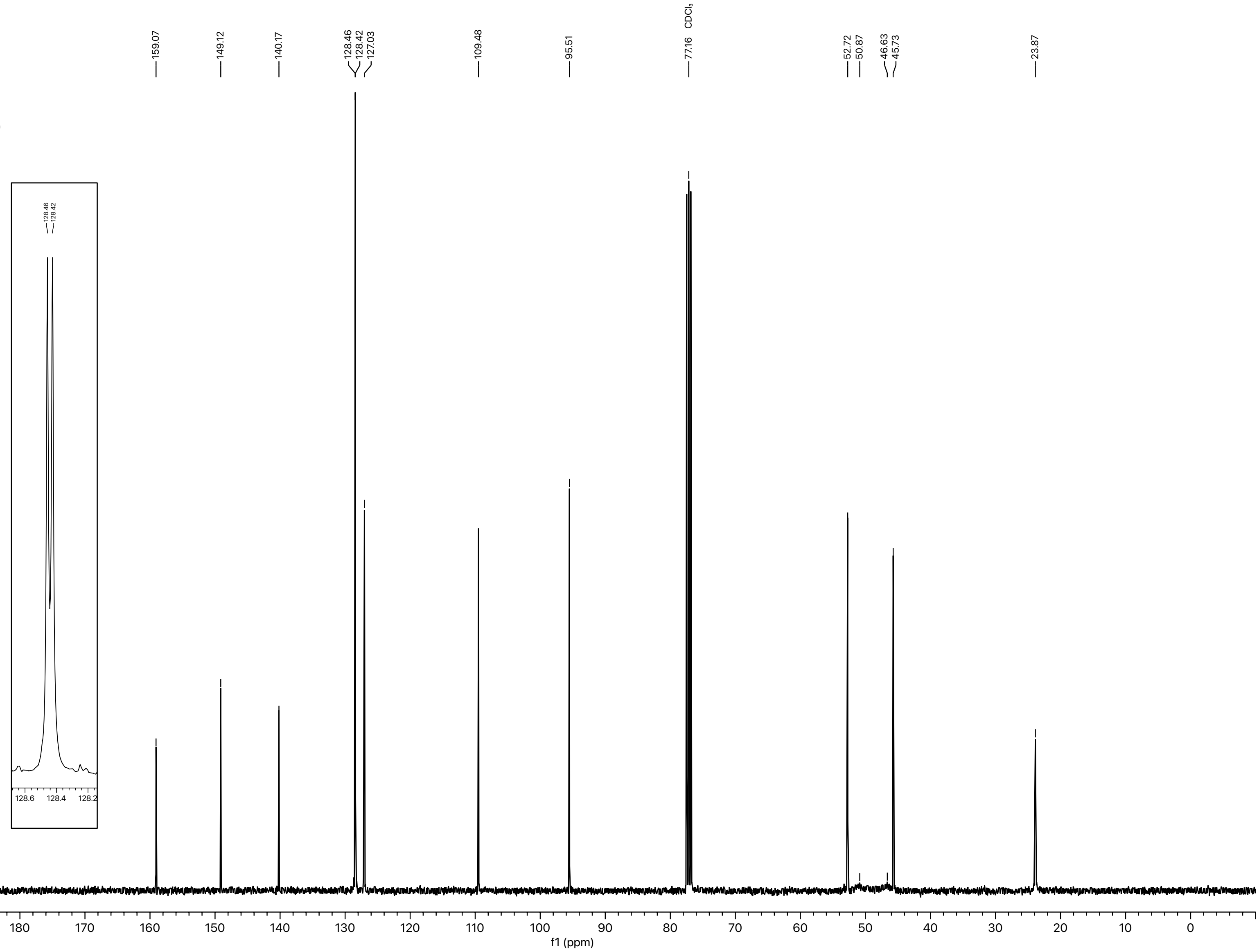

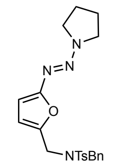

2-triazenyl-furan-24
^{1}H NMR
$CDCl_3$, 400 MHz

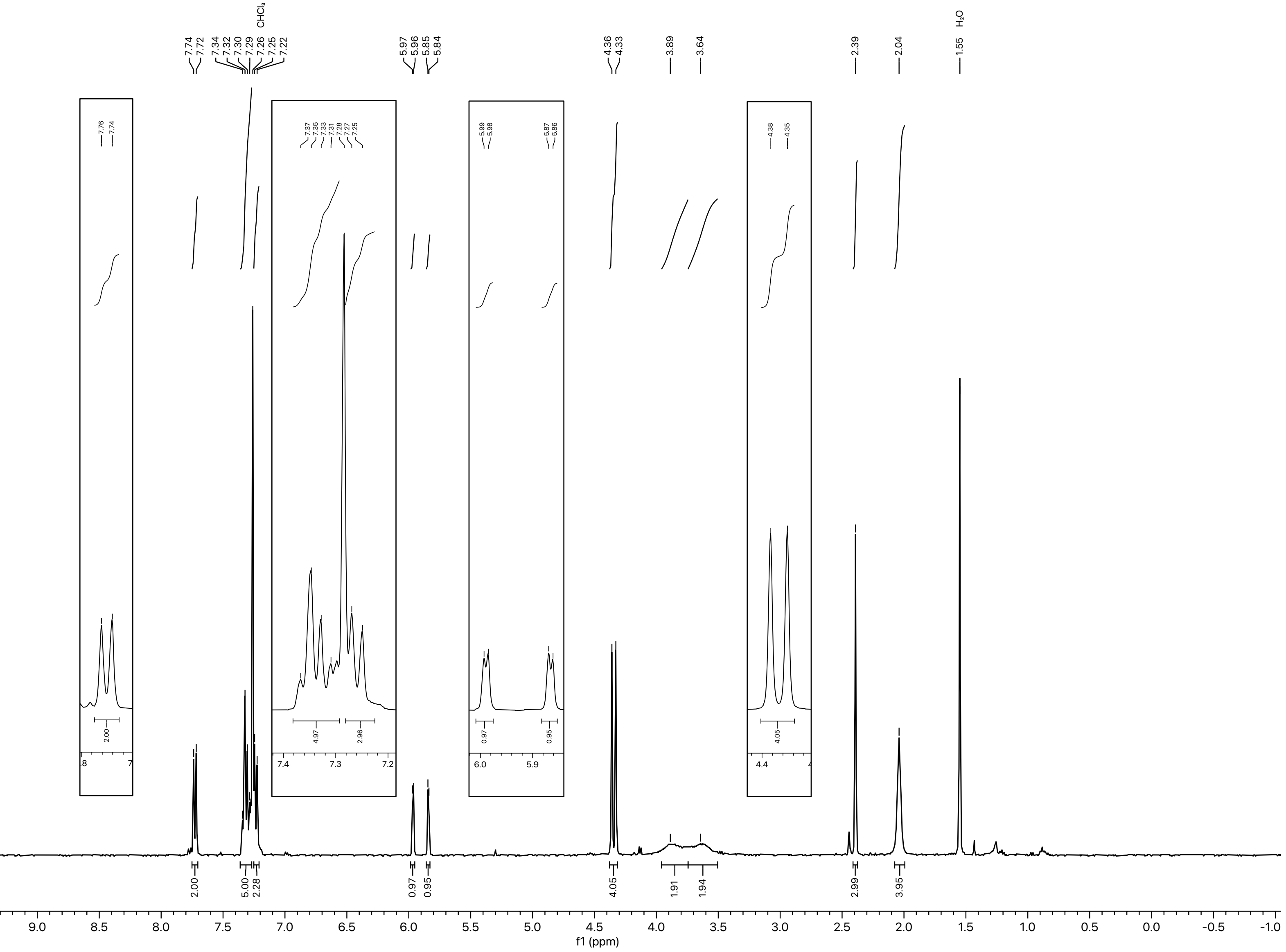

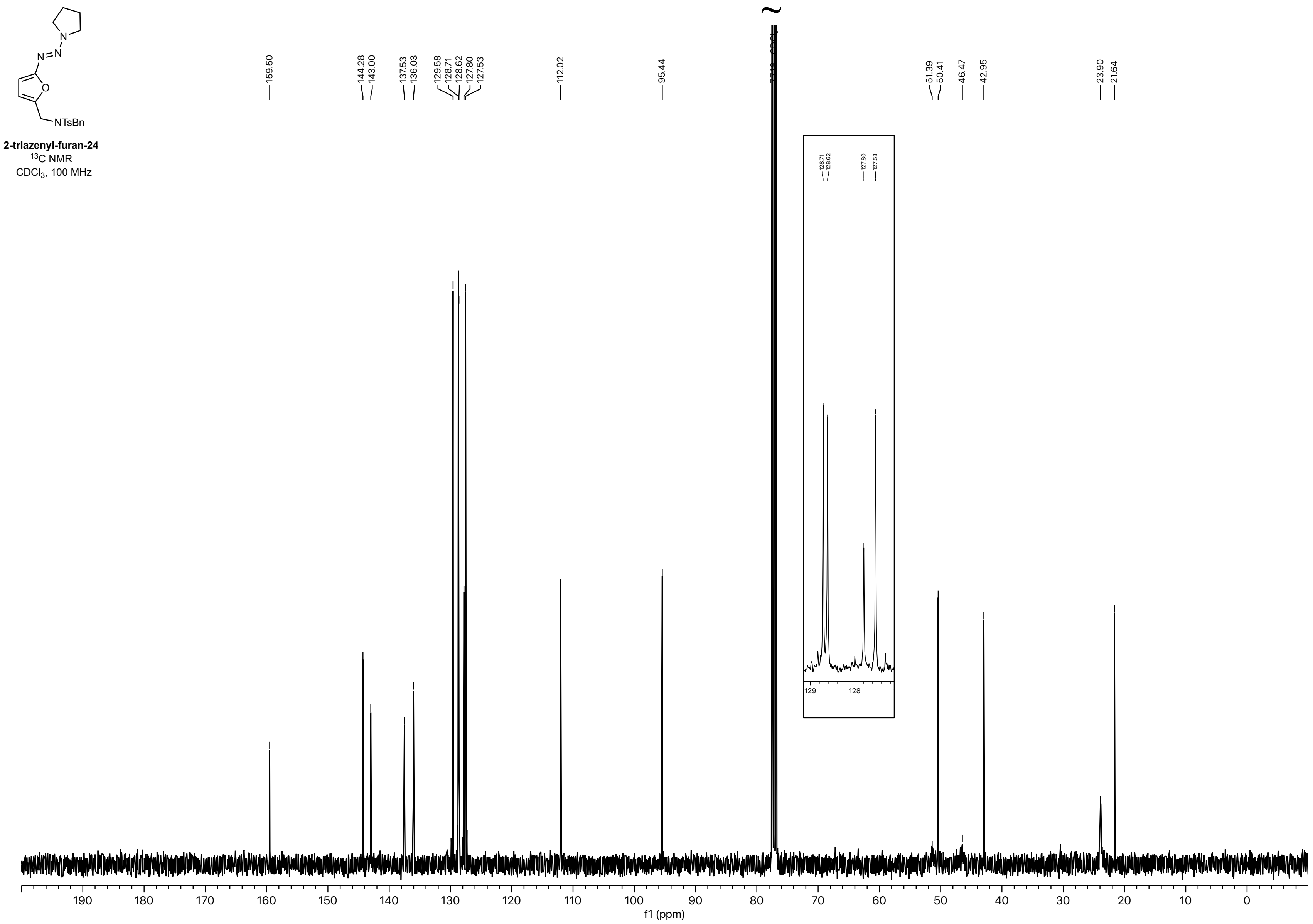

2-triazenyl-furan-24
^{13}C NMR
$CDCl_3$, 100 MHz

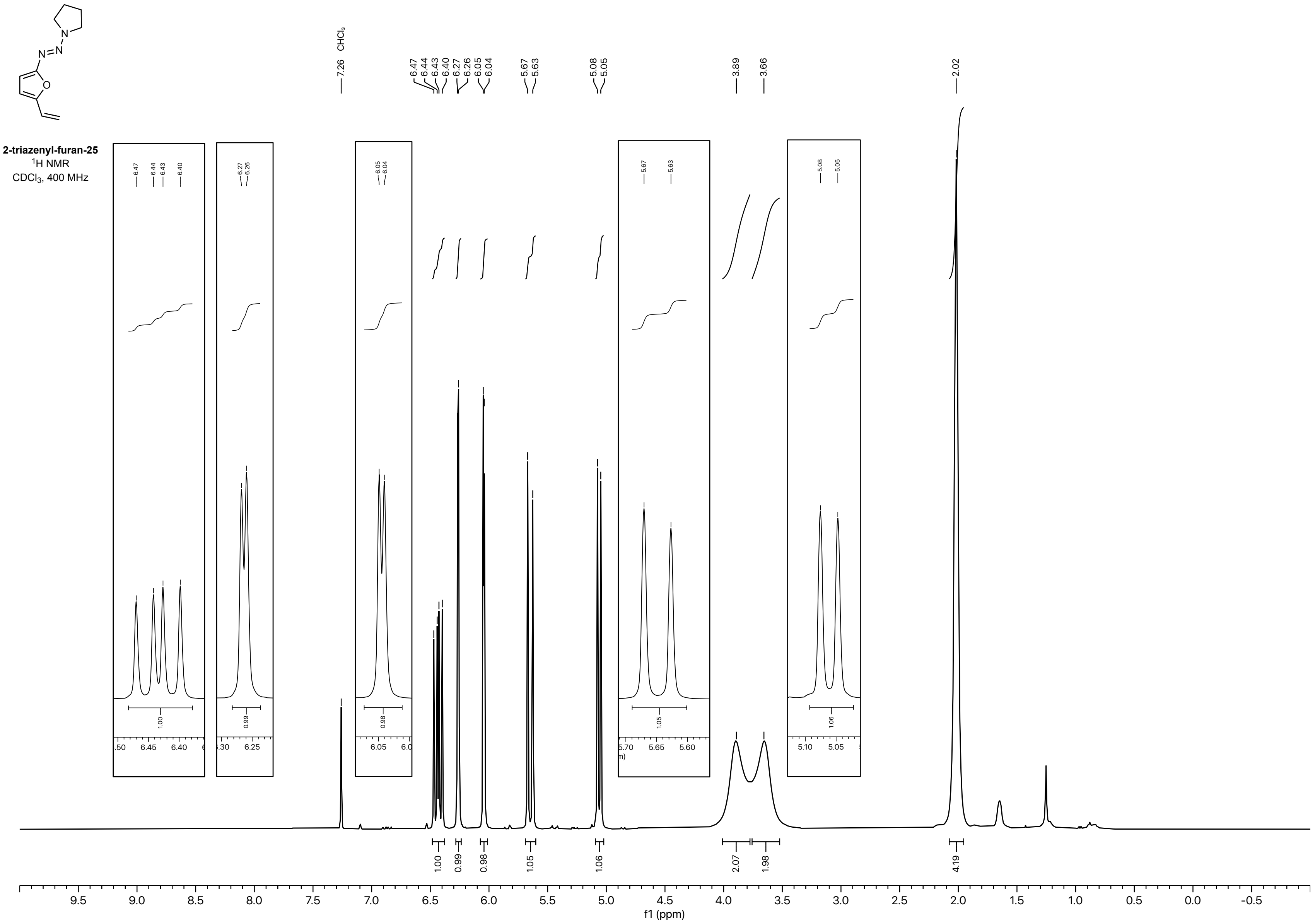

2-triazenyl-furan-25
^{1}H NMR
$CDCl_3$, 400 MHz

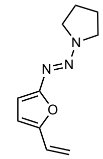

2-triazenyl-furan-25
^{13}C NMR
$CDCl_3$, 100 MHz

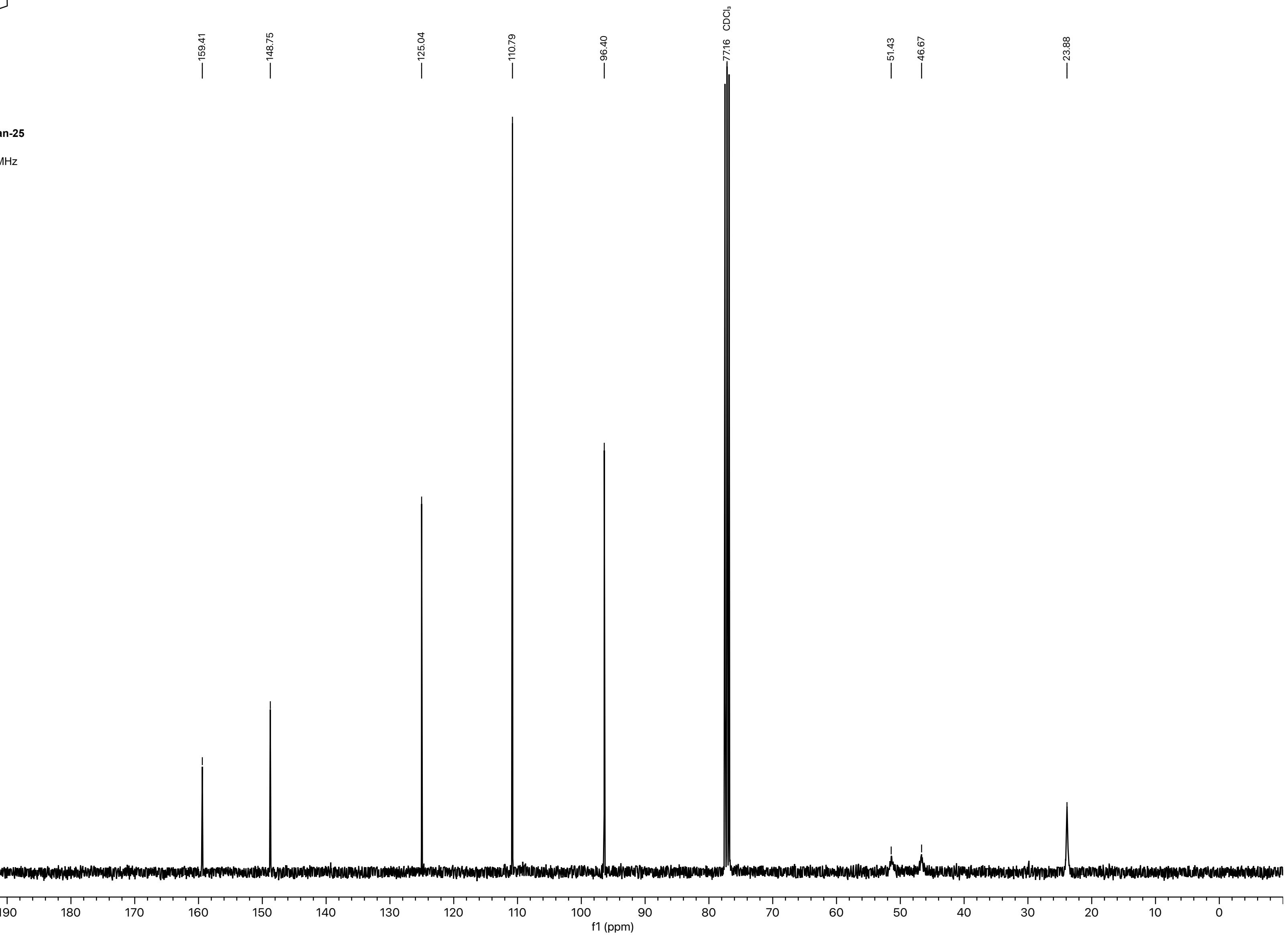

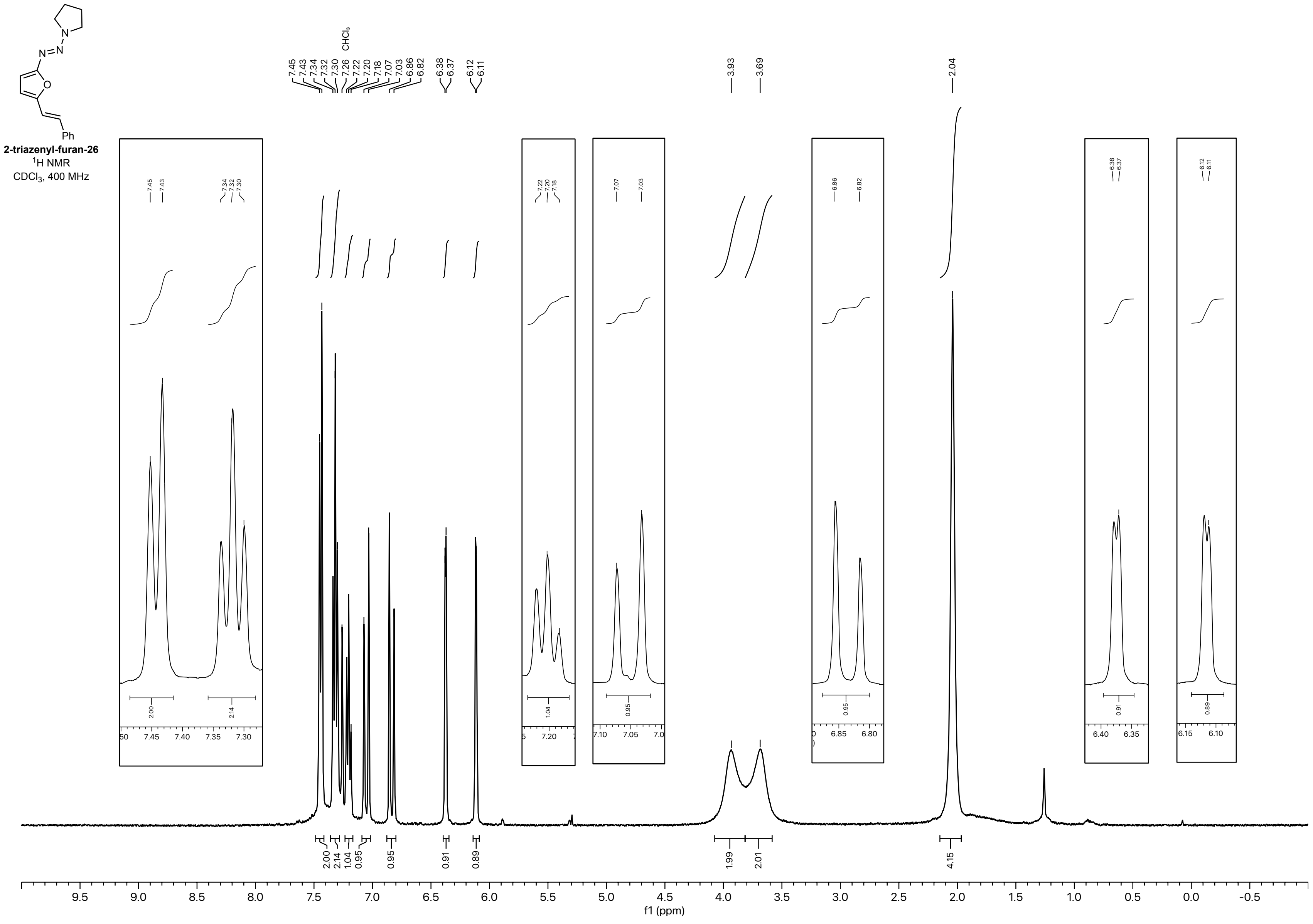
N=N
O
Ph
2-triazenyl-furan-26
^{1}H NMR
CDCl$_3$, 400 MHz
CHCl$_3$
7.45
7.43
7.34
7.32
7.30
7.26
7.22
7.20
7.18
7.07
7.03
6.86
6.82
6.38
6.37
6.12
6.11
3.93
3.69
2.04
2.00
2.14
1.04
0.95
0.95
0.91
0.89
1.99
2.01
4.15
f1 (ppm)

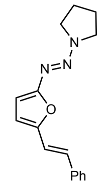

2-triazenyl-furan-26

^{13}C NMR

$CDCl_3$, 100 MHz

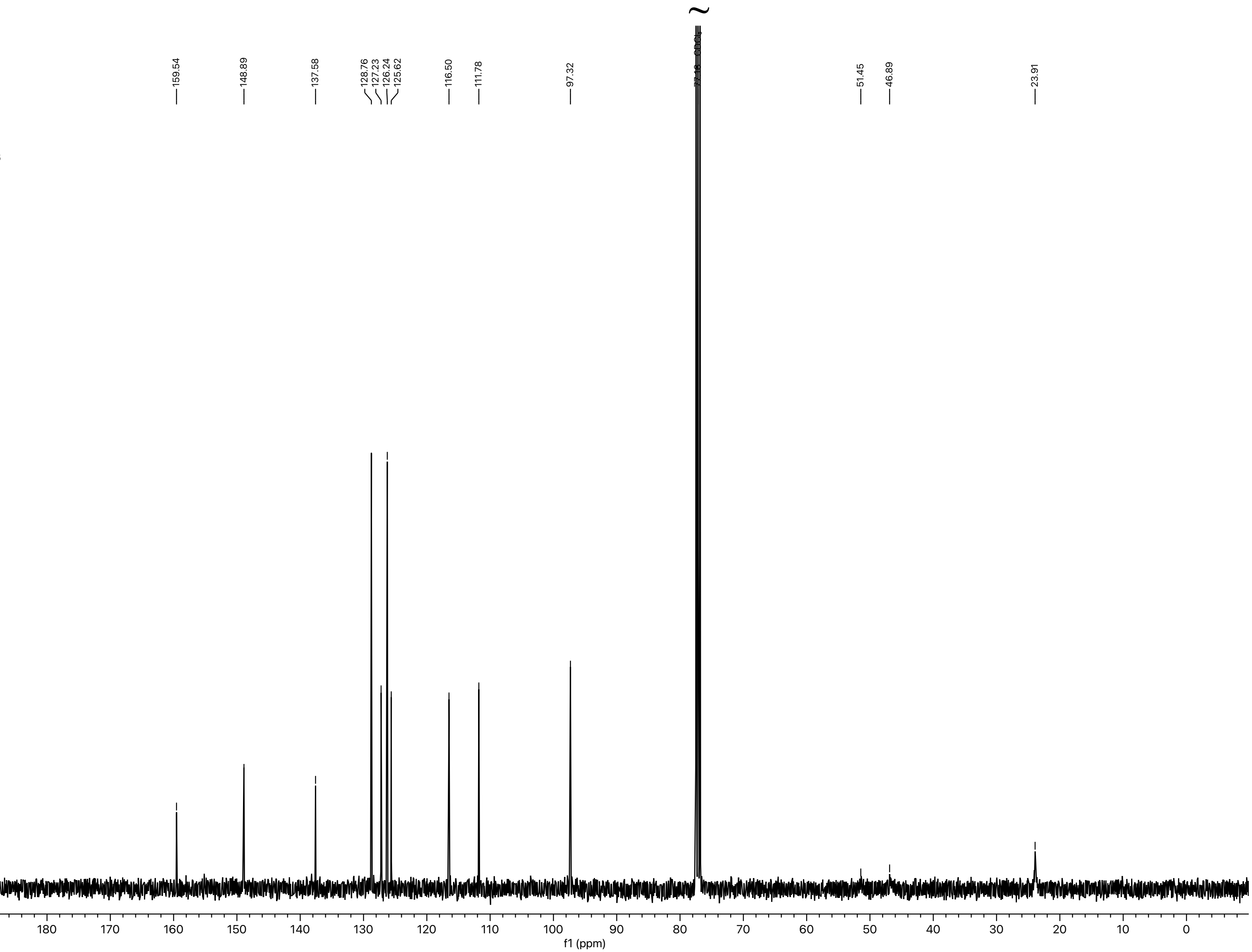

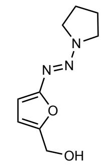

2-triazenyl-furan-27
^{1}H NMR
$CDCl_3$, 400 MHz

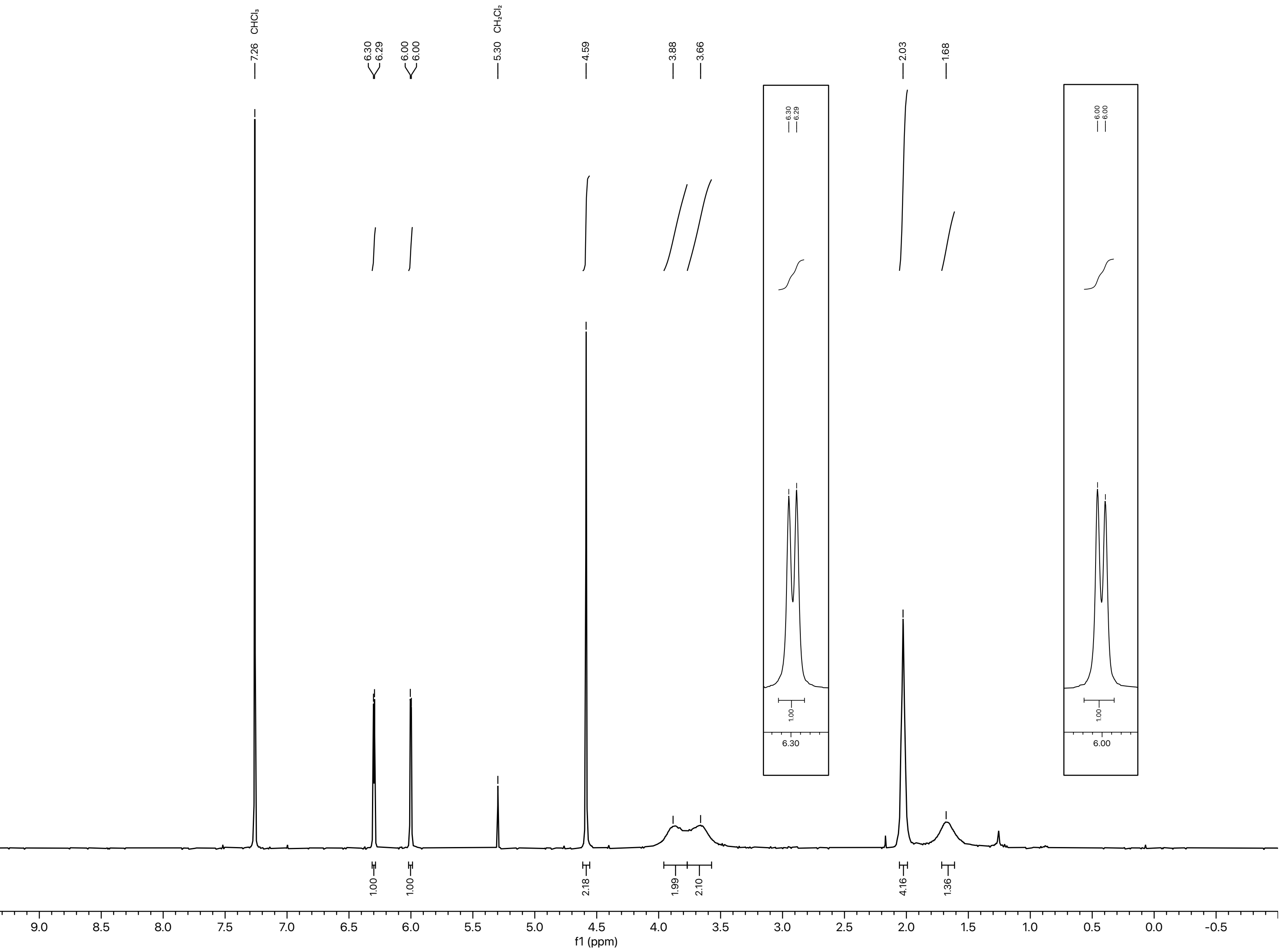

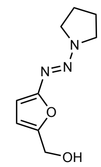

2-triazenyl-furan-27
^{13}C NMR
$CDCl_3$, 100 MHz

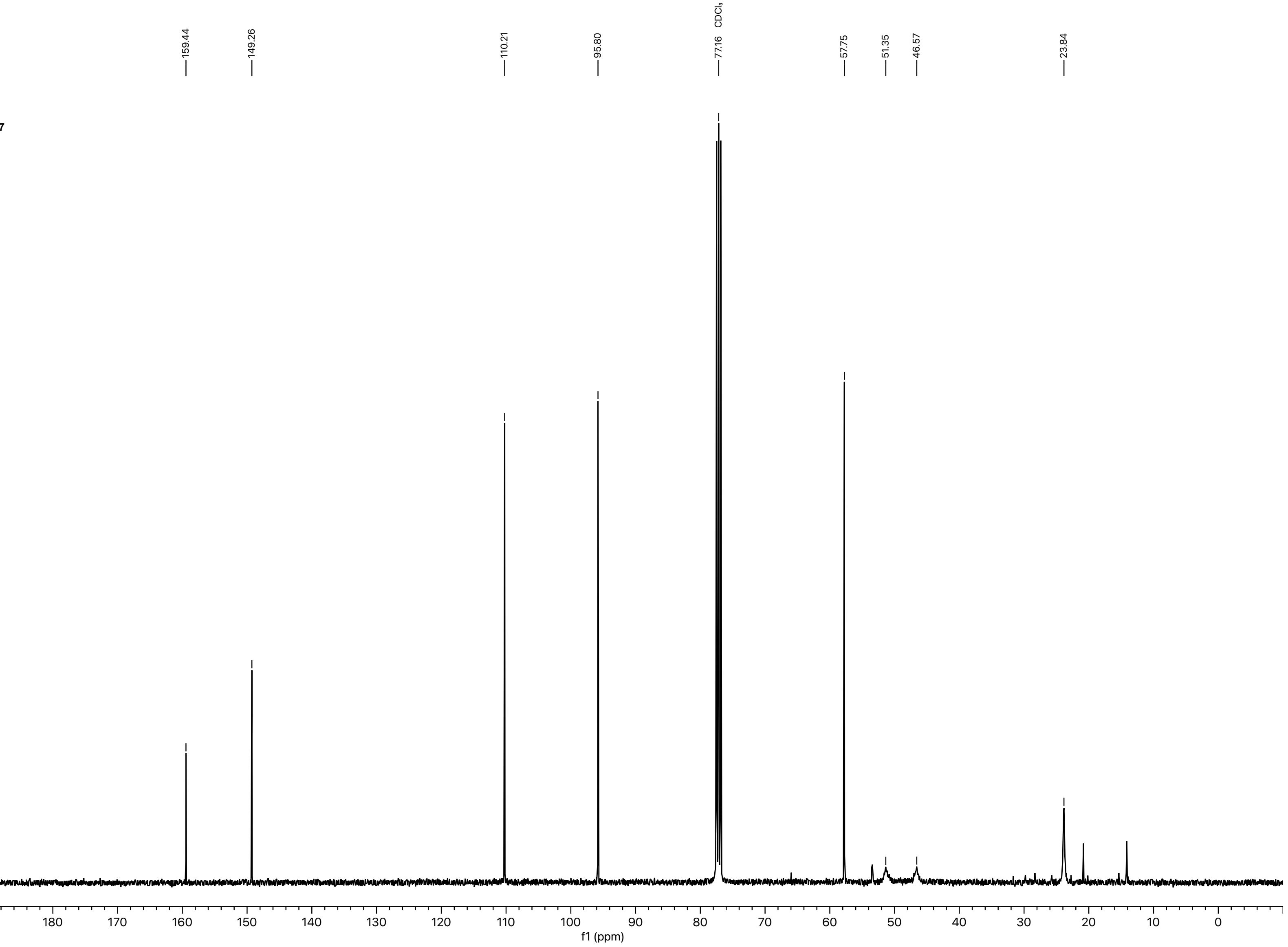

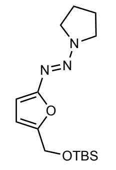

2-triazenyl-furan-28
^{1}H NMR
$CDCl_3$, 400 MHz

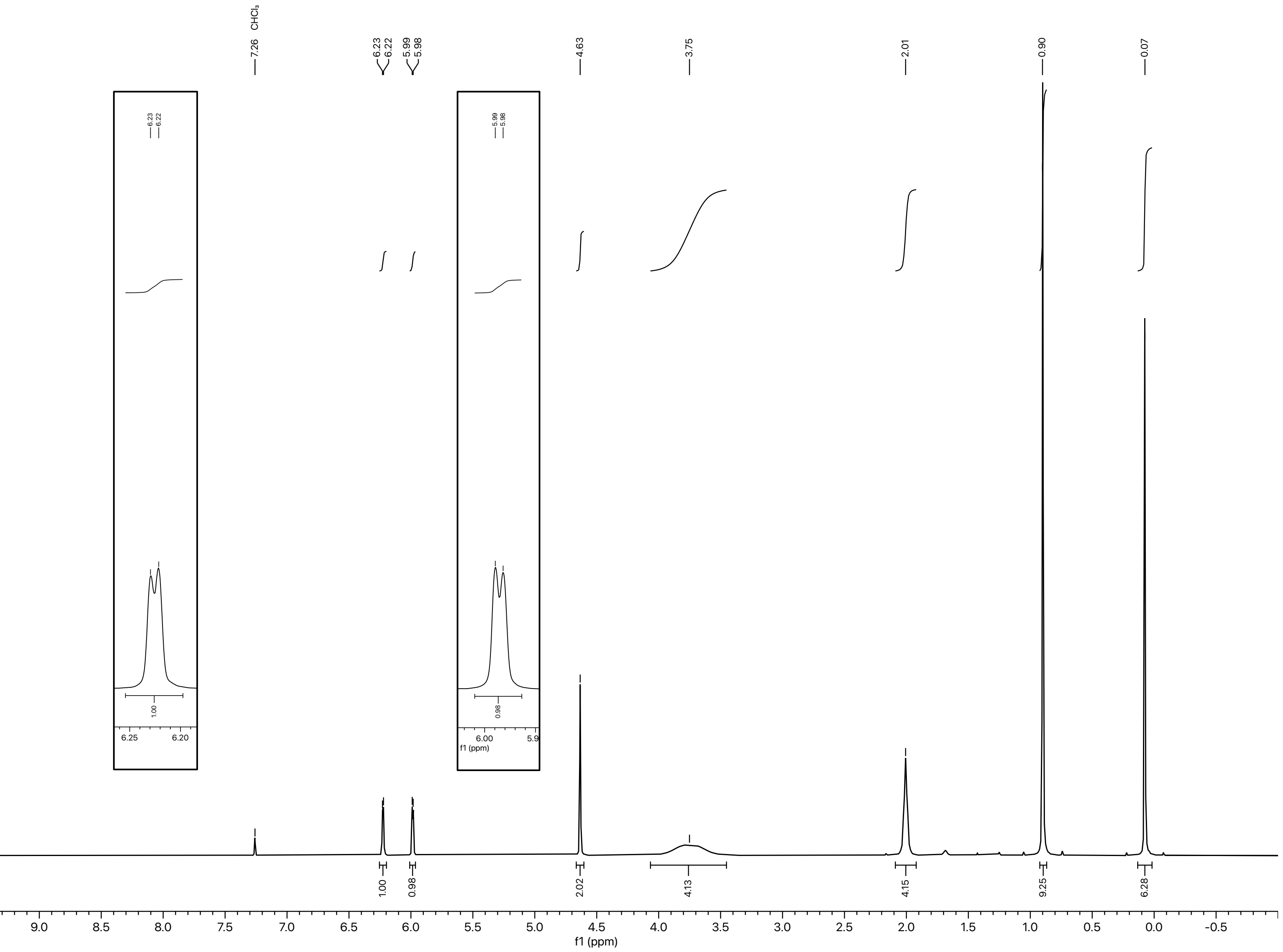

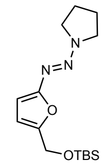

2-triazenyl-furan-28
^{13}C NMR
$CDCl_3$, 100 MHz

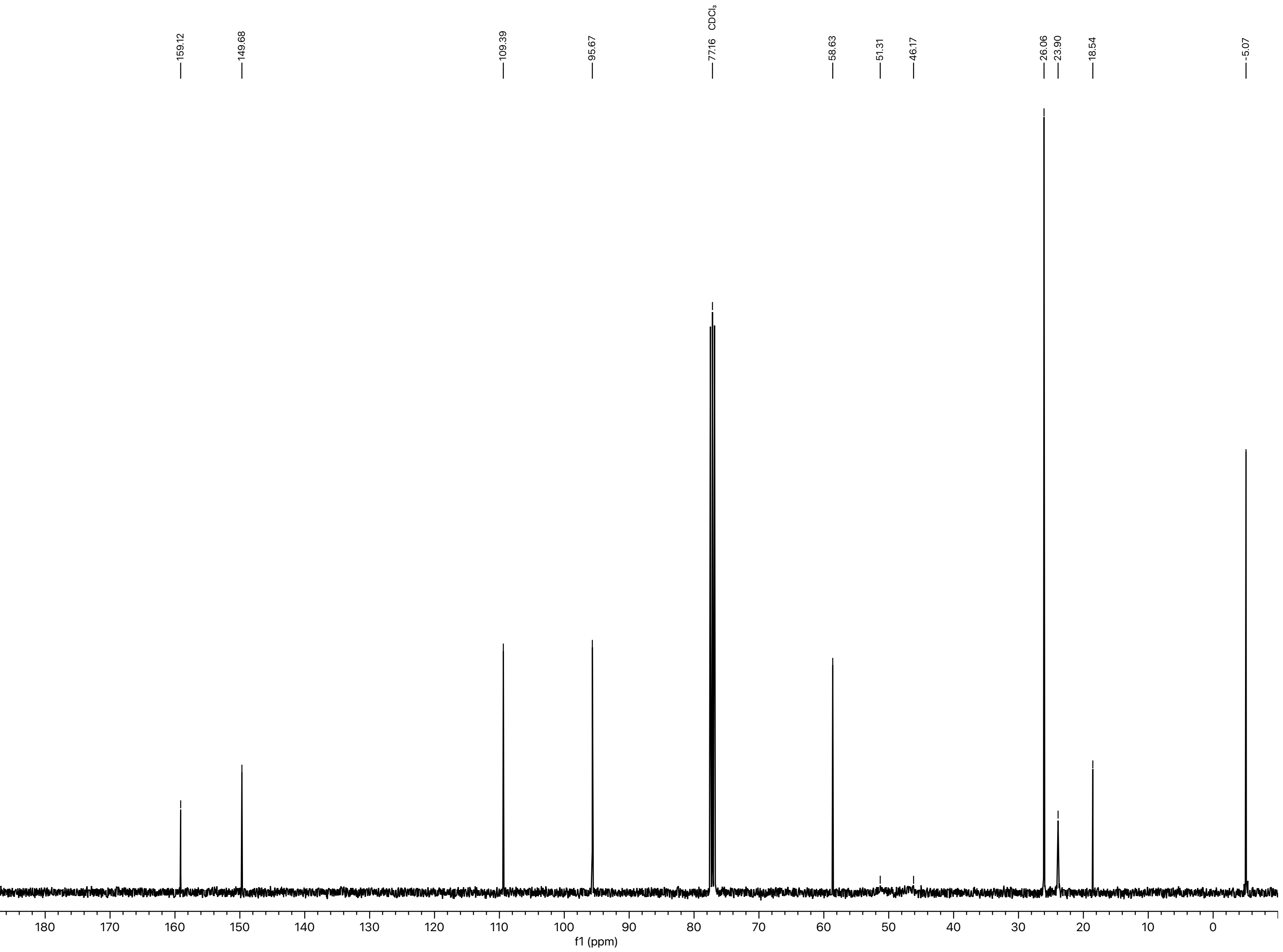

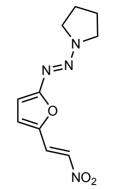

2-triazenyl-furan-29
^{1}H NMR
$CDCl_3$, 400 MHz

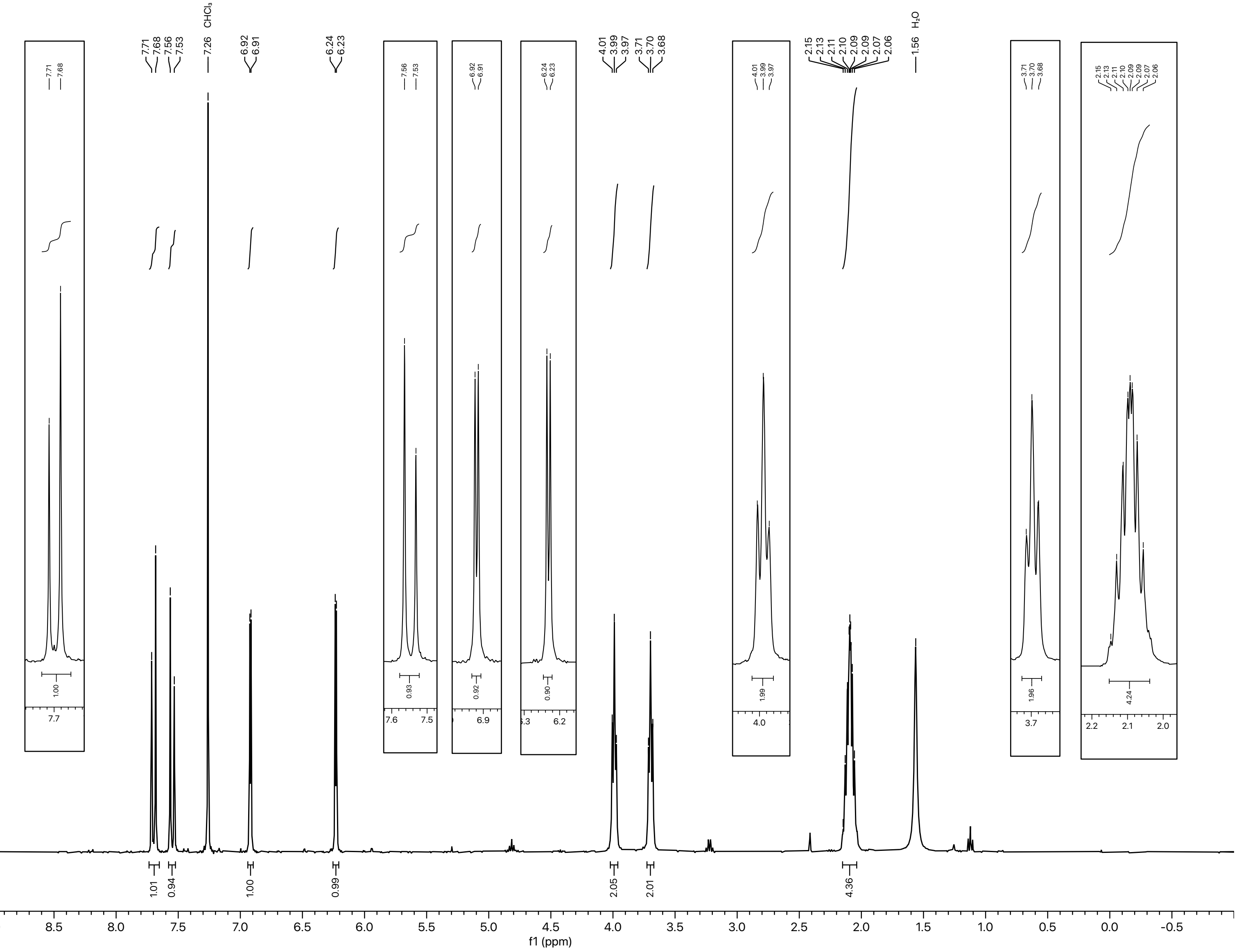

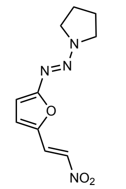

2-triazenyl-furan-29
^{13}C NMR
$CDCl_3$, 100 MHz

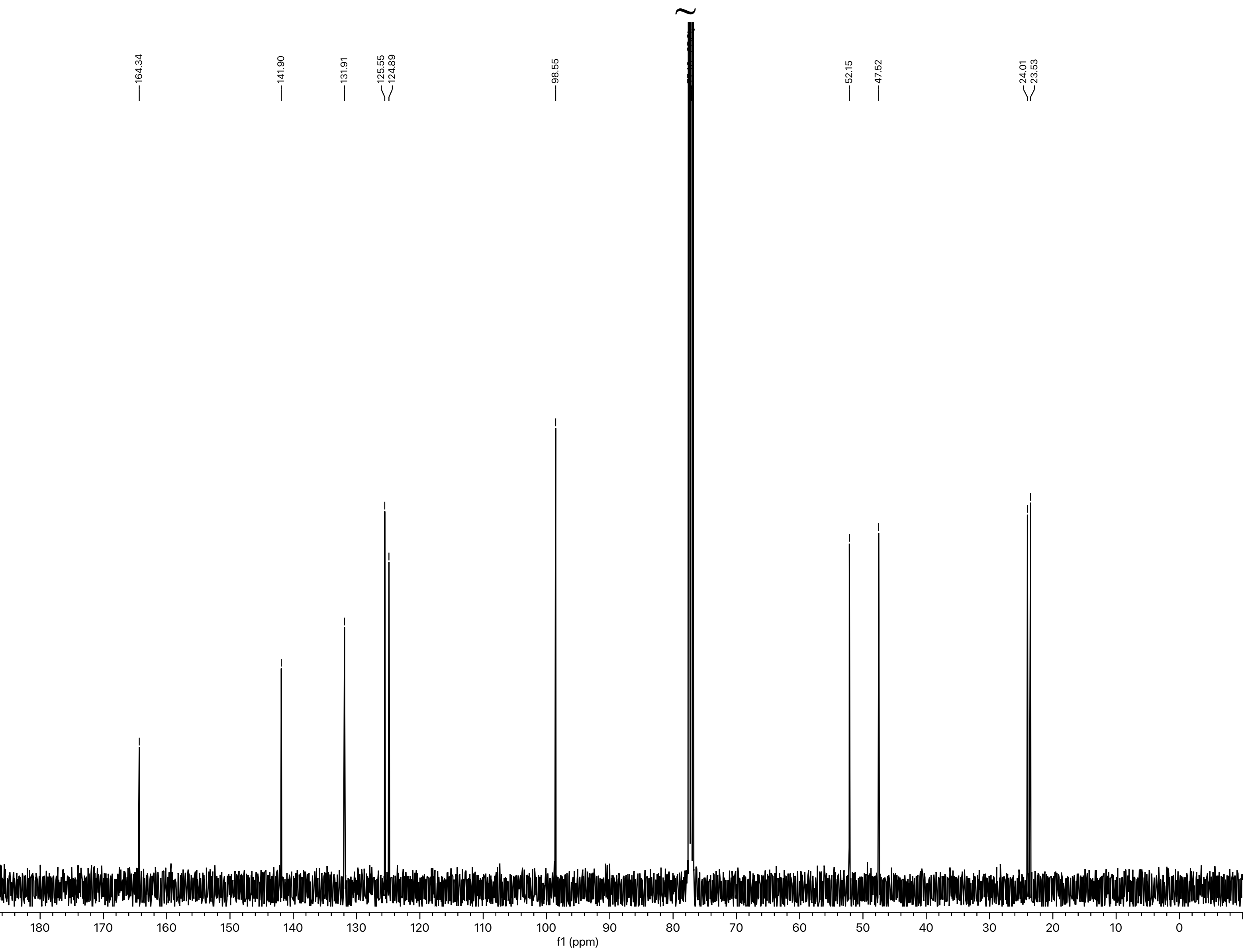

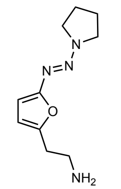

2-triazenyl-furan-30
^1H NMR
$CDCl_3$, 400 MHz

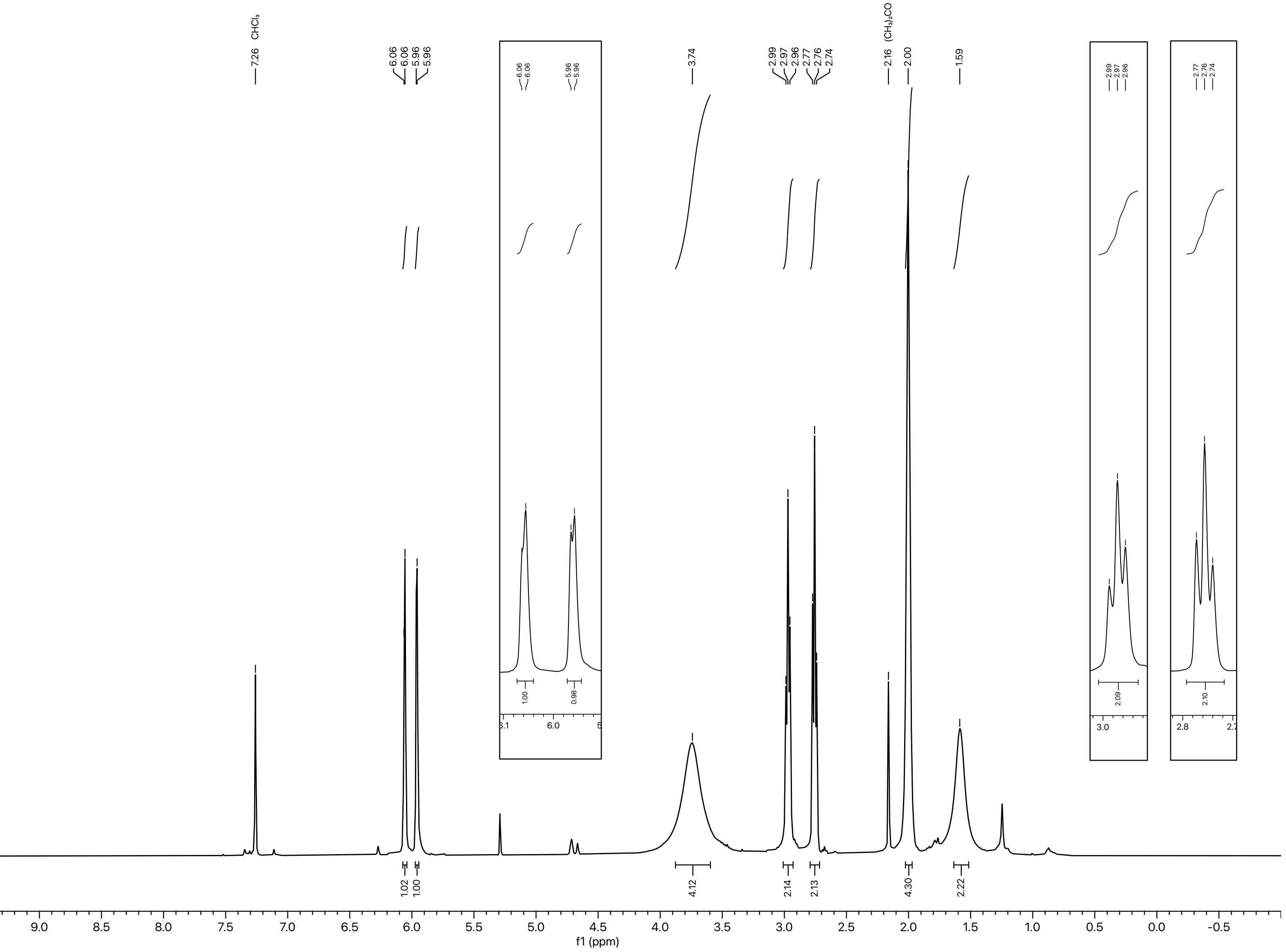

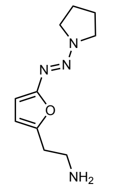

2-triazenyl-furan-30
^{13}C NMR
$CDCl_3$, 100 MHz

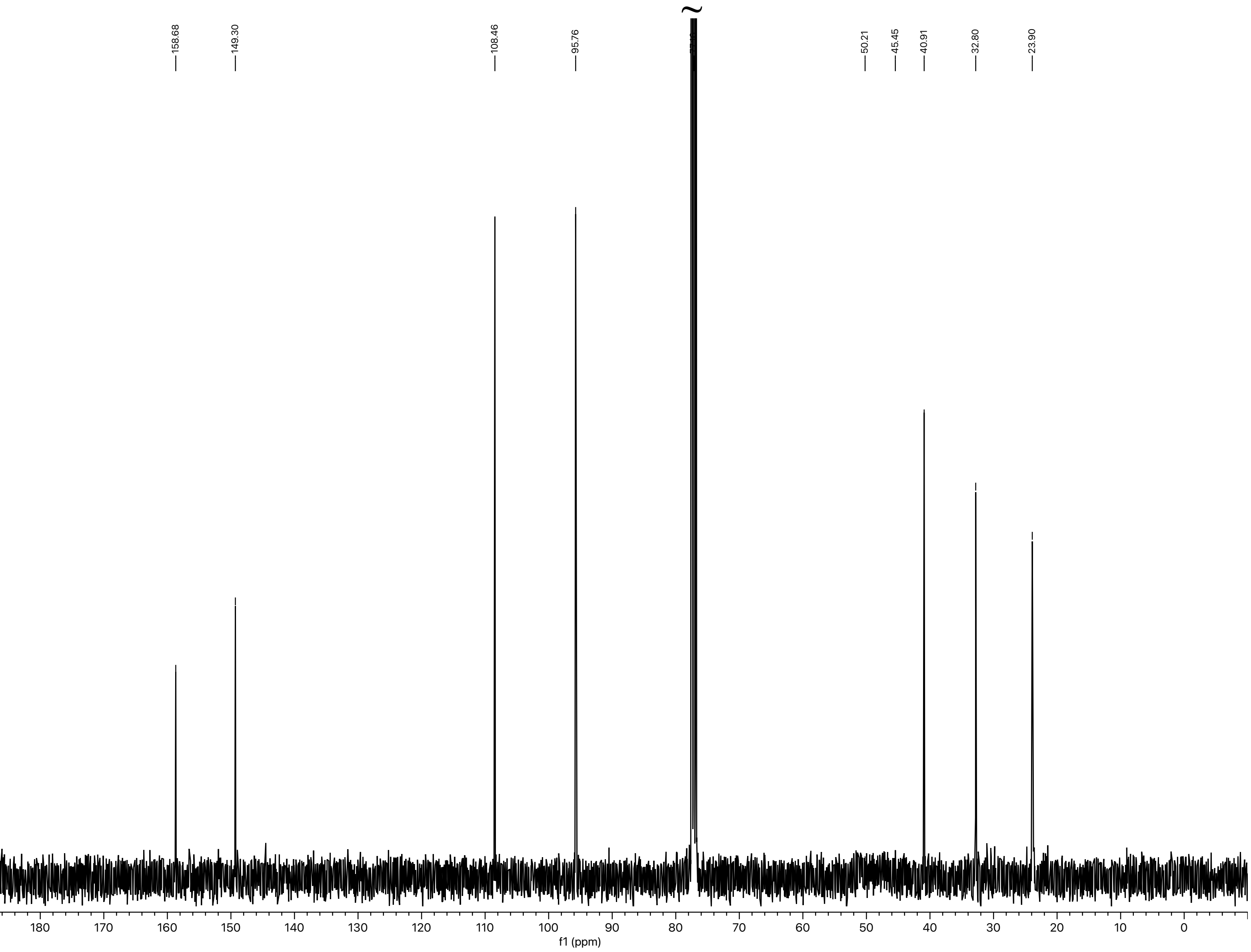

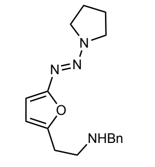

2-triazenyl-furan-31 (or **44**)
^{1}H NMR
$CDCl_3$, 400 MHz

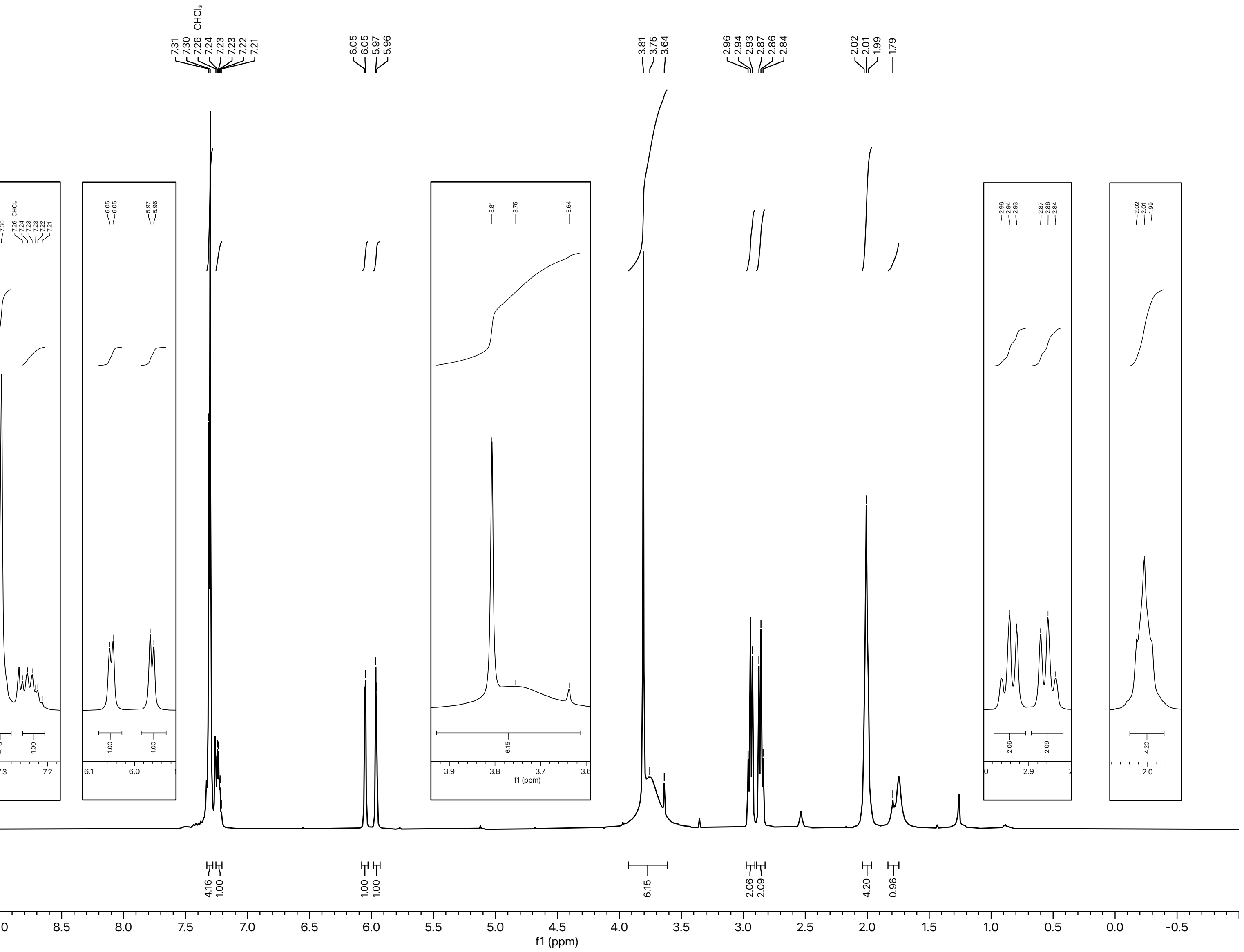

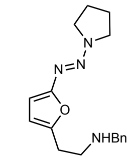

2-triazenyl-furan-31 (or **44**)
^{13}C NMR
$CDCl_3$, 100 MHz

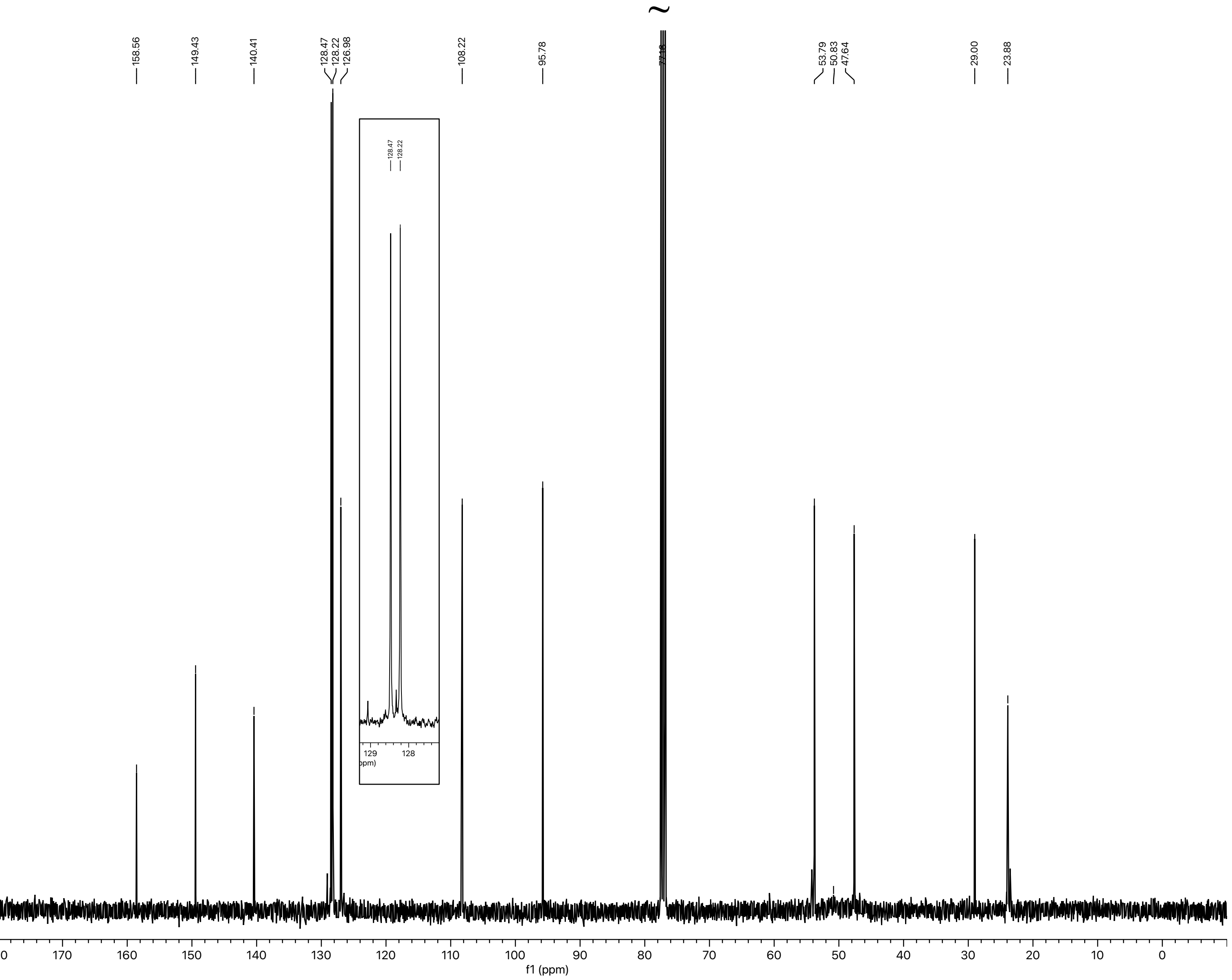

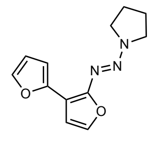

2-triazenyl-furan-32
^{1}H NMR
$CDCl_3$, 400 MHz

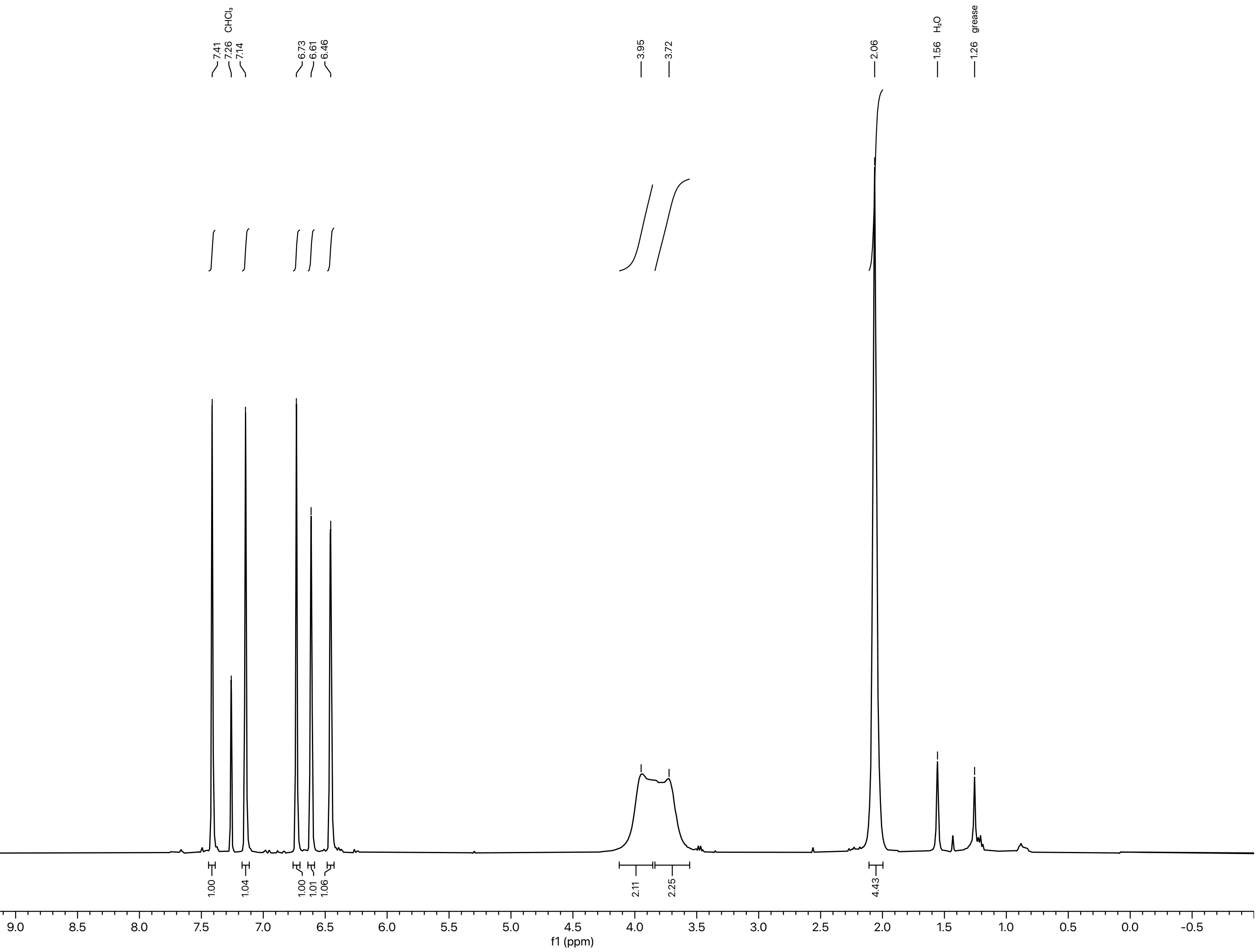

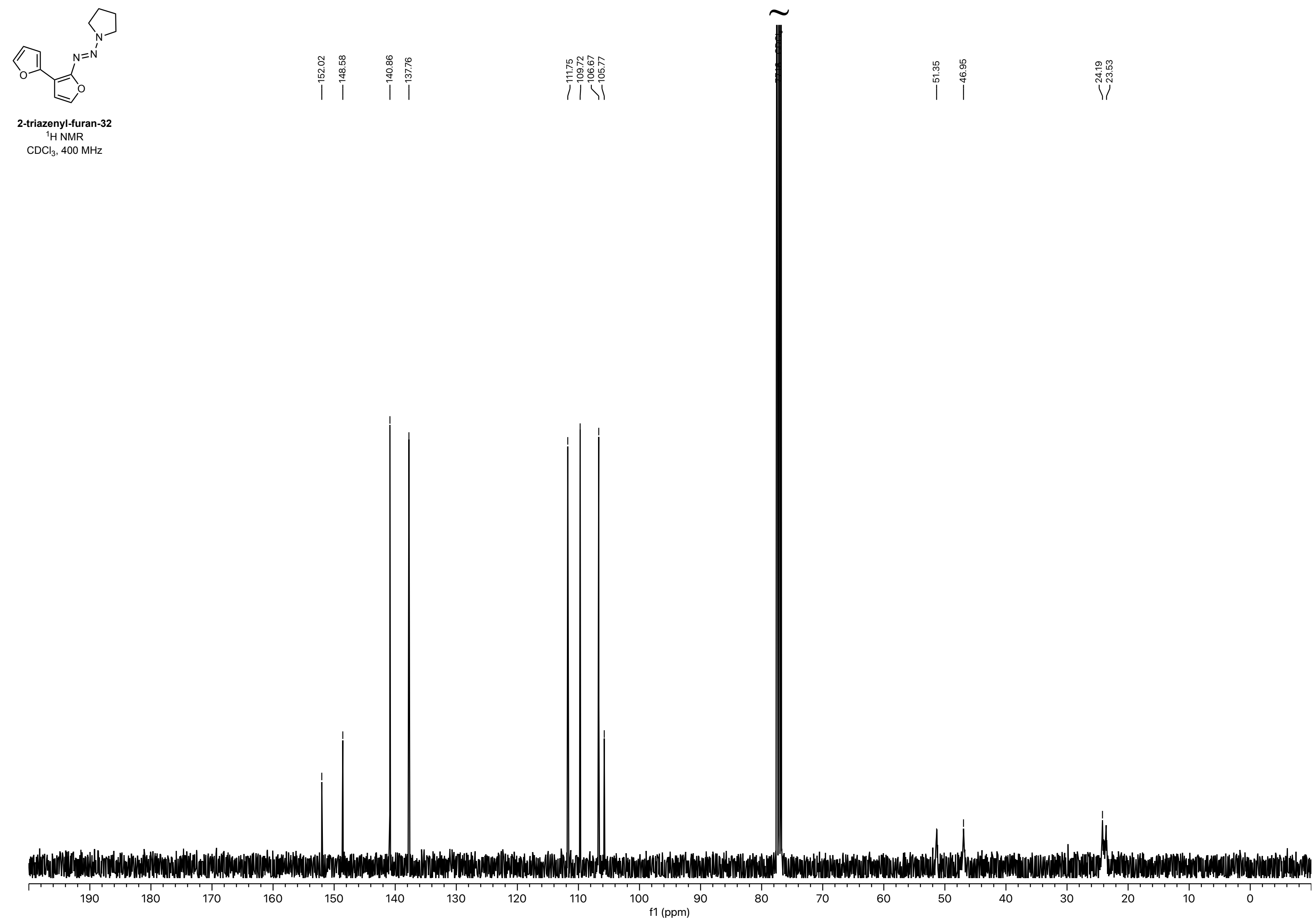

2-triazenyl-furan-32
^{1}H NMR
$CDCl_3$, 400 MHz

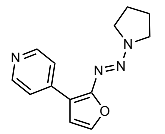

2-triazenyl-furan-33
^{1}H NMR
$CDCl_3$, 400 MHz

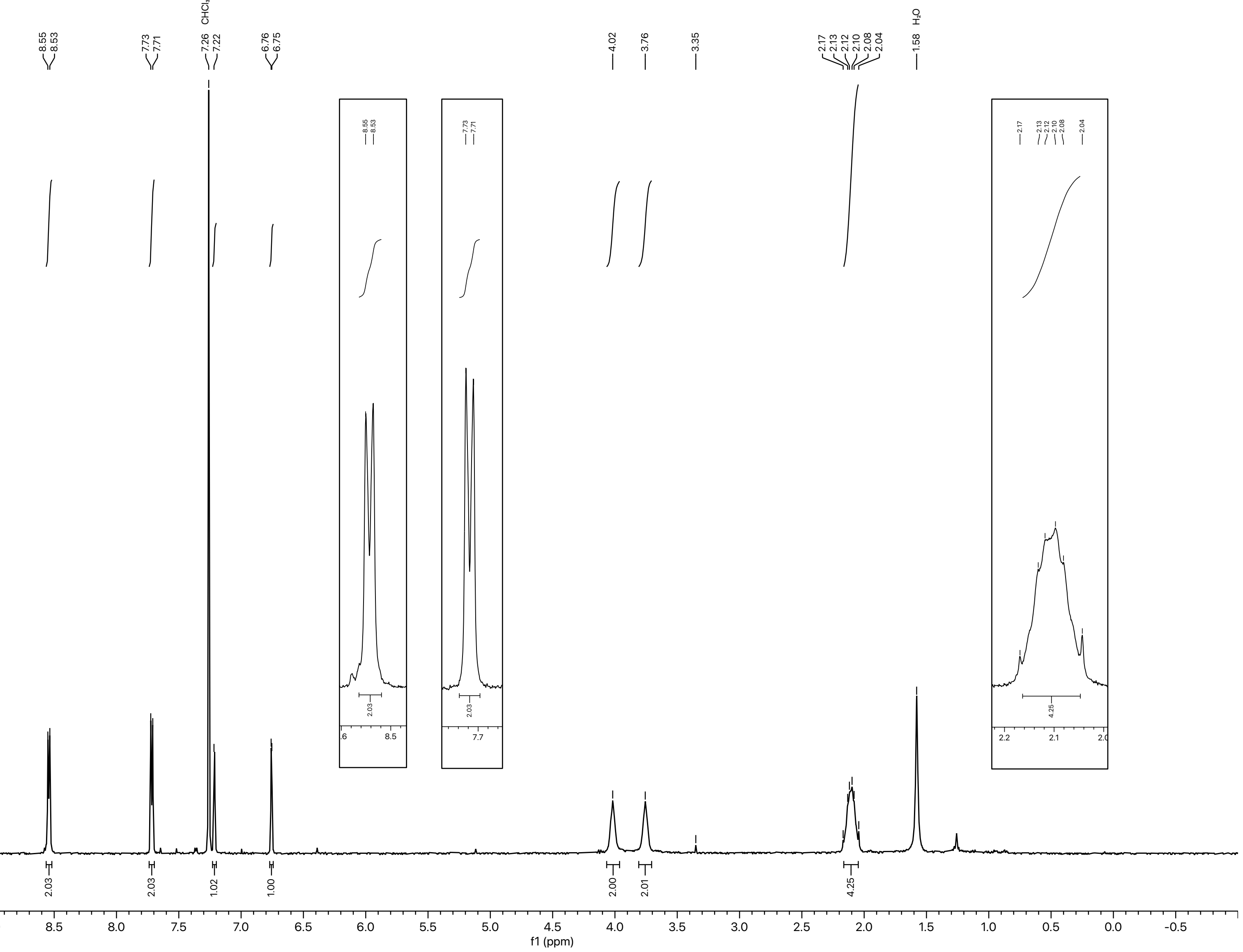

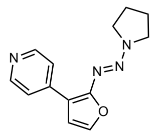

2-triazenyl-furan-33
^{13}C NMR
$CDCl_3$, 100 MHz

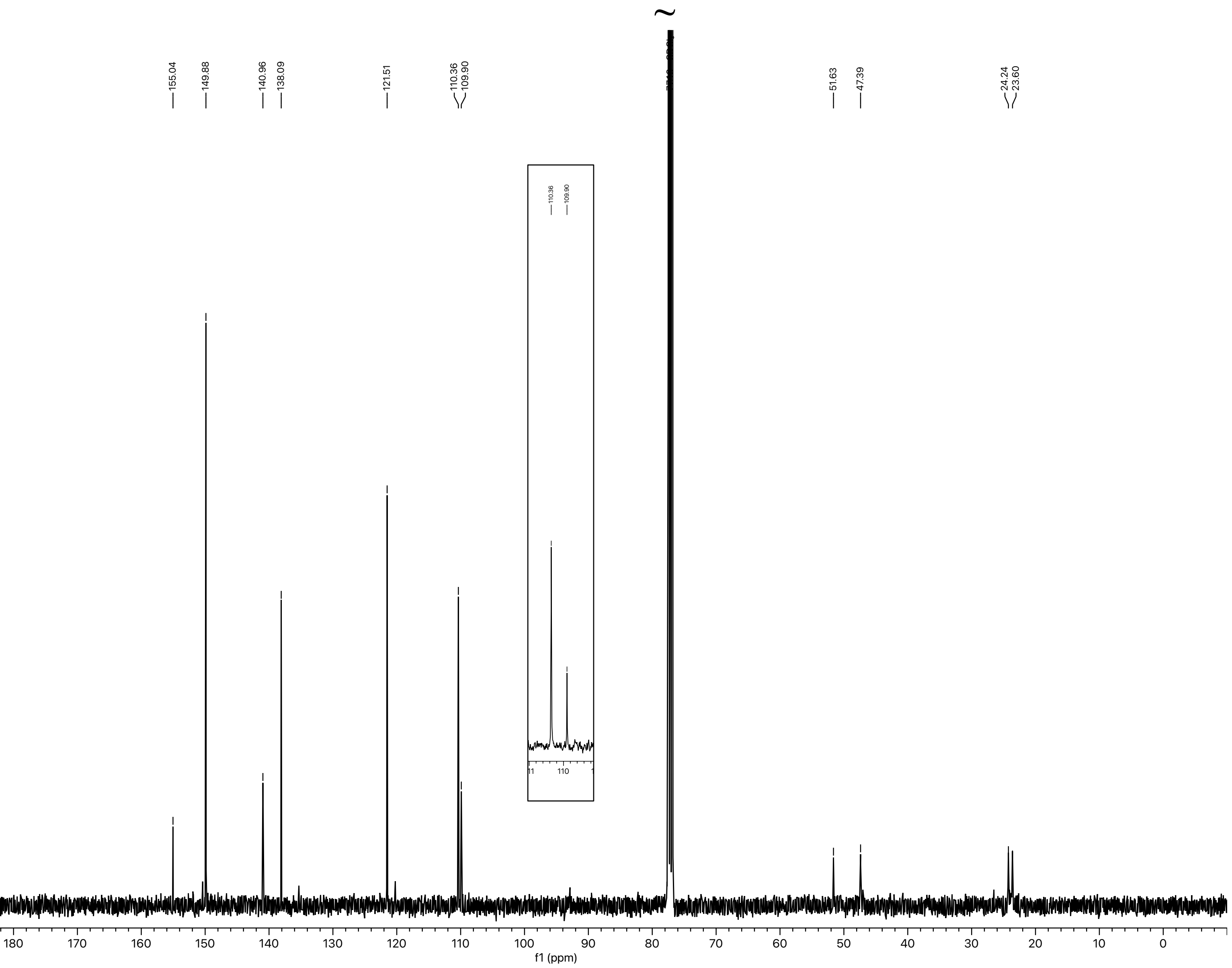

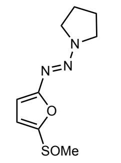

2-triazenyl-furan-34
^{1}H NMR
$CDCl_3$, 400 MHz

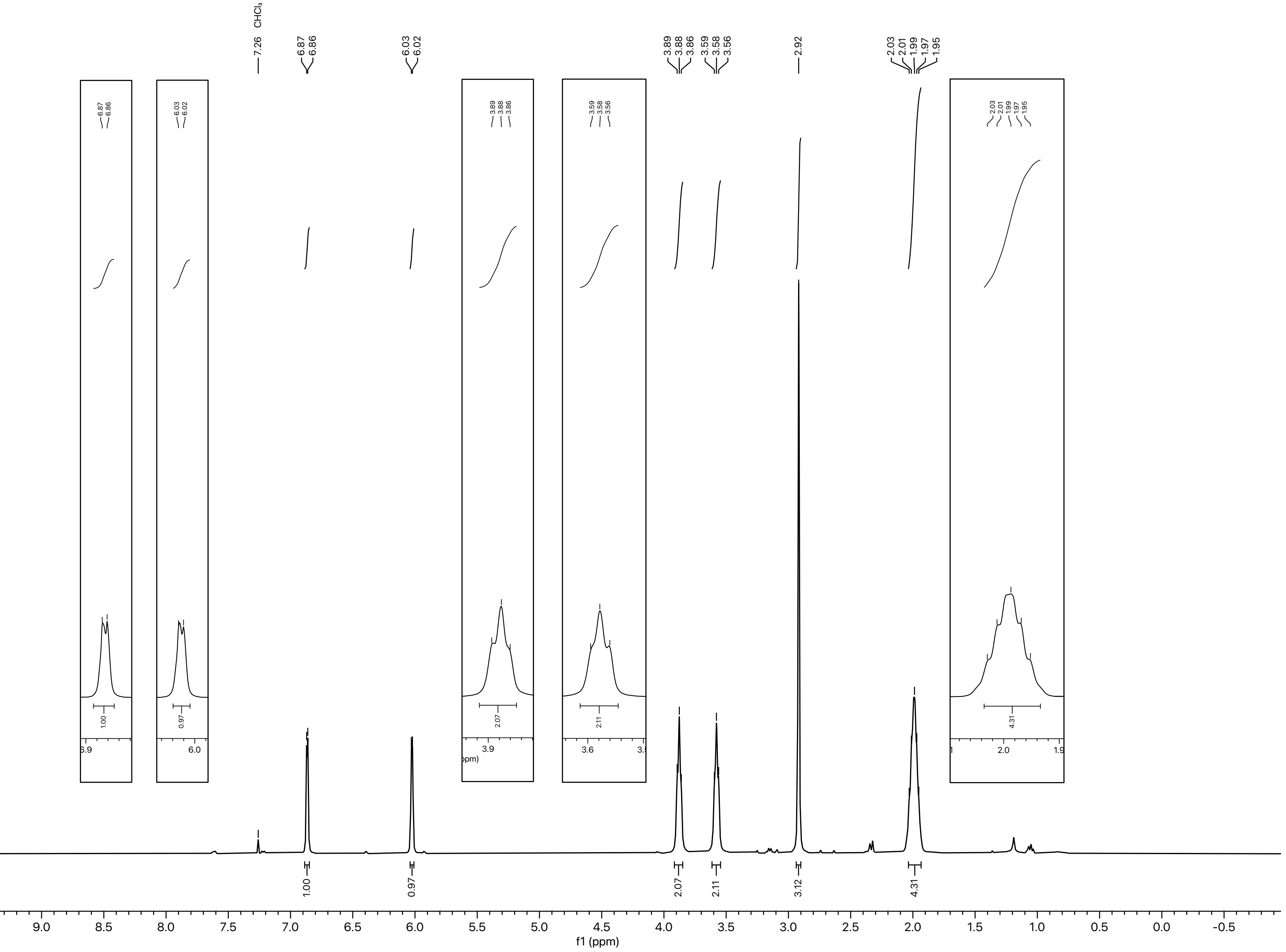

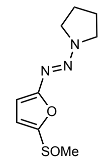

2-triazenyl-furan-34
^{13}C NMR
$CDCl_3$, 100 MHz

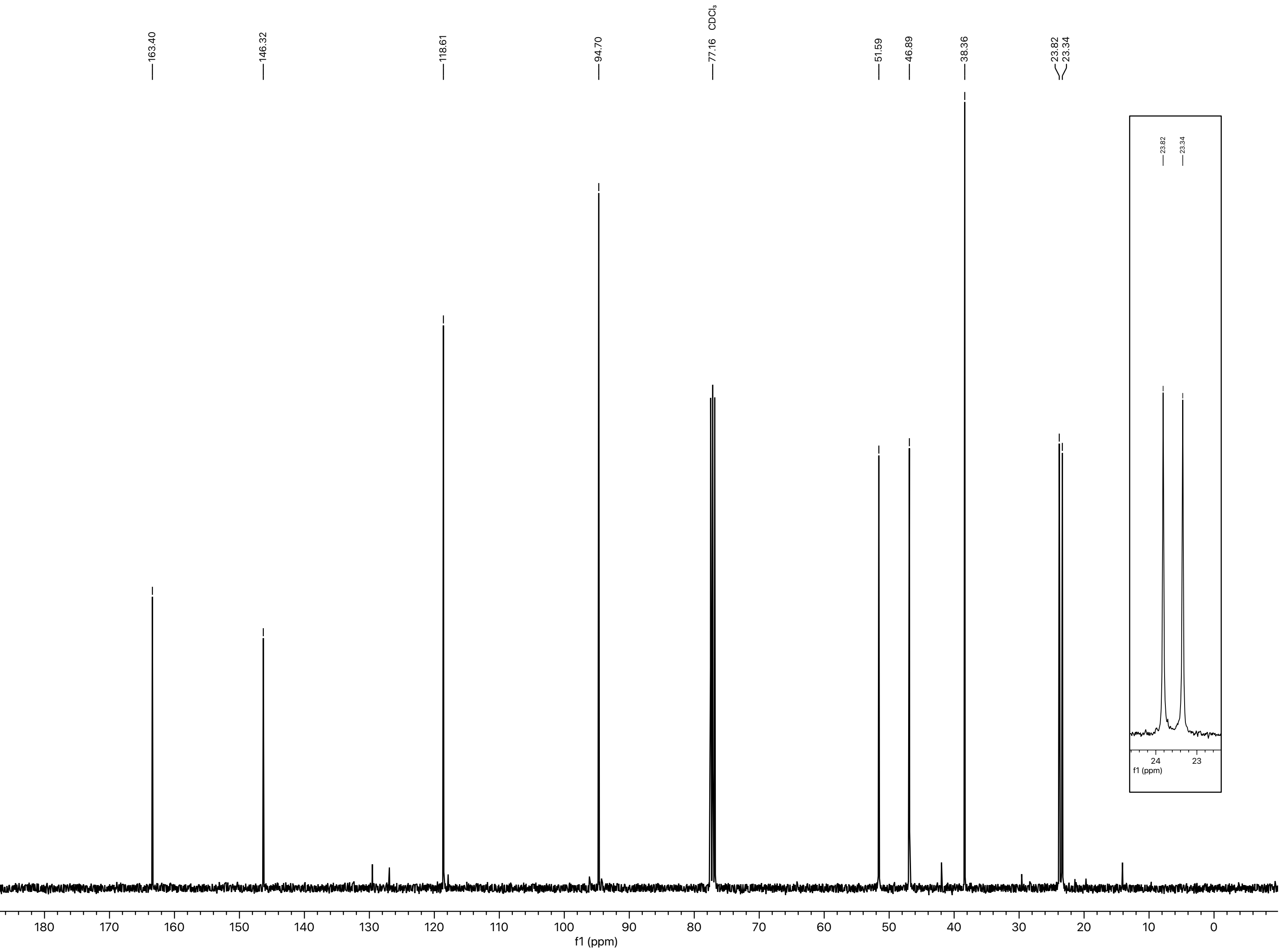

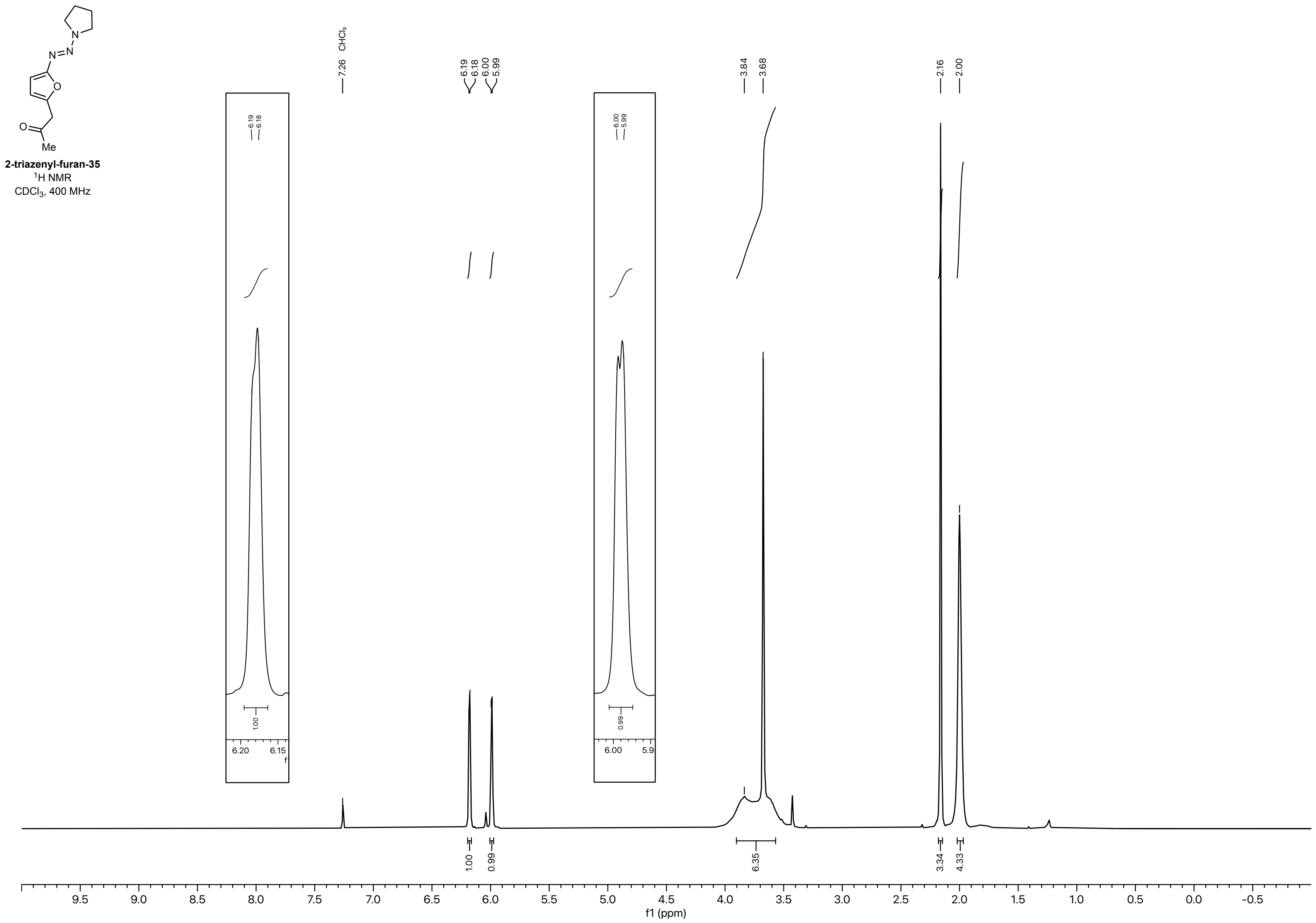

N=N
N
O
O
Me
2-triazenyl-furan-35
¹H NMR
CDCl₃, 400 MHz
7.26 CHCl₃
6.19
6.18
6.00
5.99
3.84
3.68
2.16
2.00
1.00
0.99
6.35
3.34
4.33
6.20
6.15
6.00
5.9
9.5
9.0
8.5
8.0
7.5
7.0
6.5
6.0
5.5
5.0
4.5
4.0
3.5
3.0
2.5
2.0
1.5
1.0
0.5
0.0
-0.5
f1 (ppm)

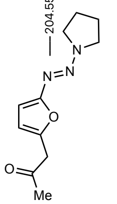

2-triazenyl-furan-35
^{13}C NMR
$CDCl_3$, 100 MHz

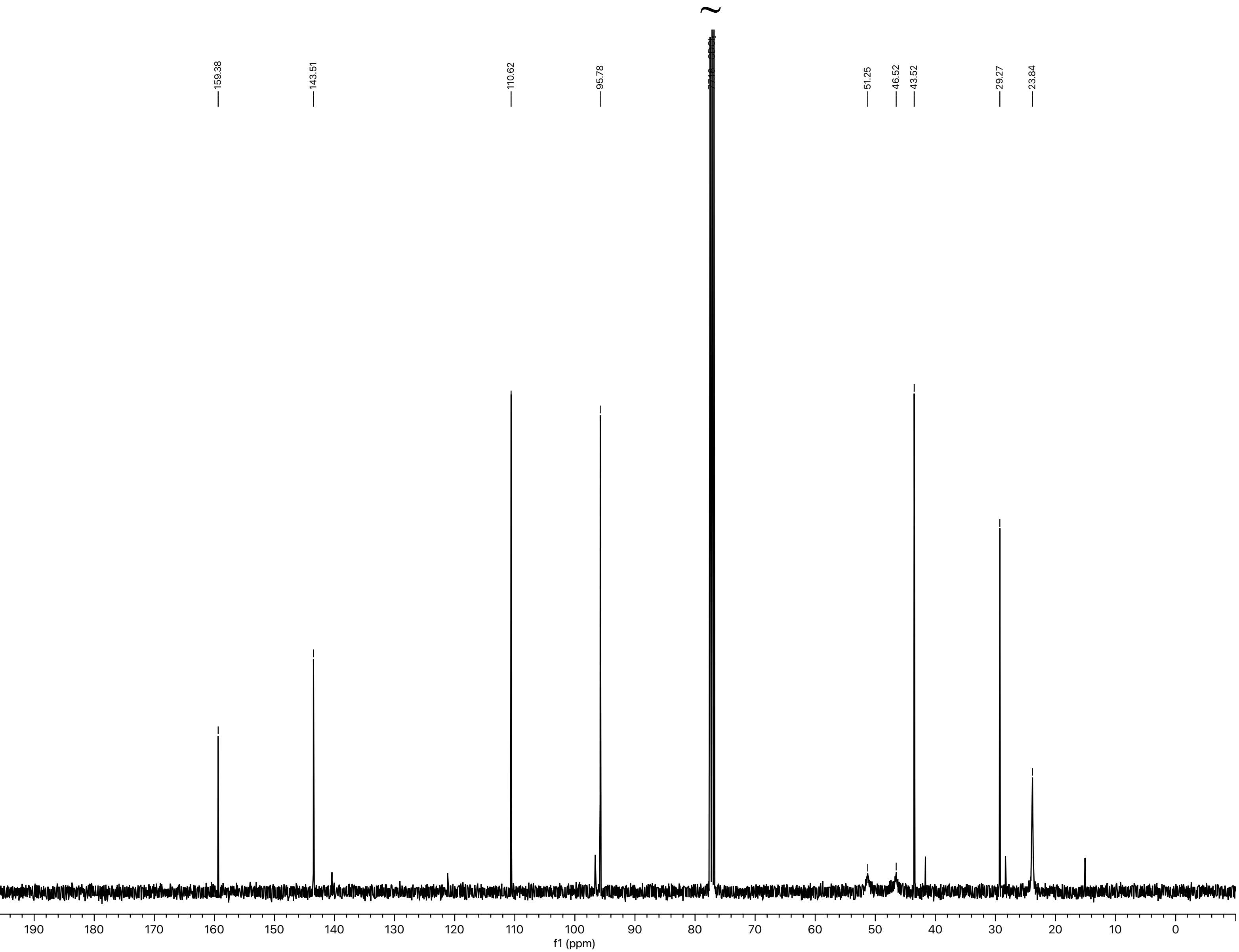

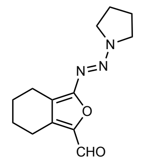

2-triazenyl-furan-36
^{1}H NMR
$CDCl_3$, 400 MHz

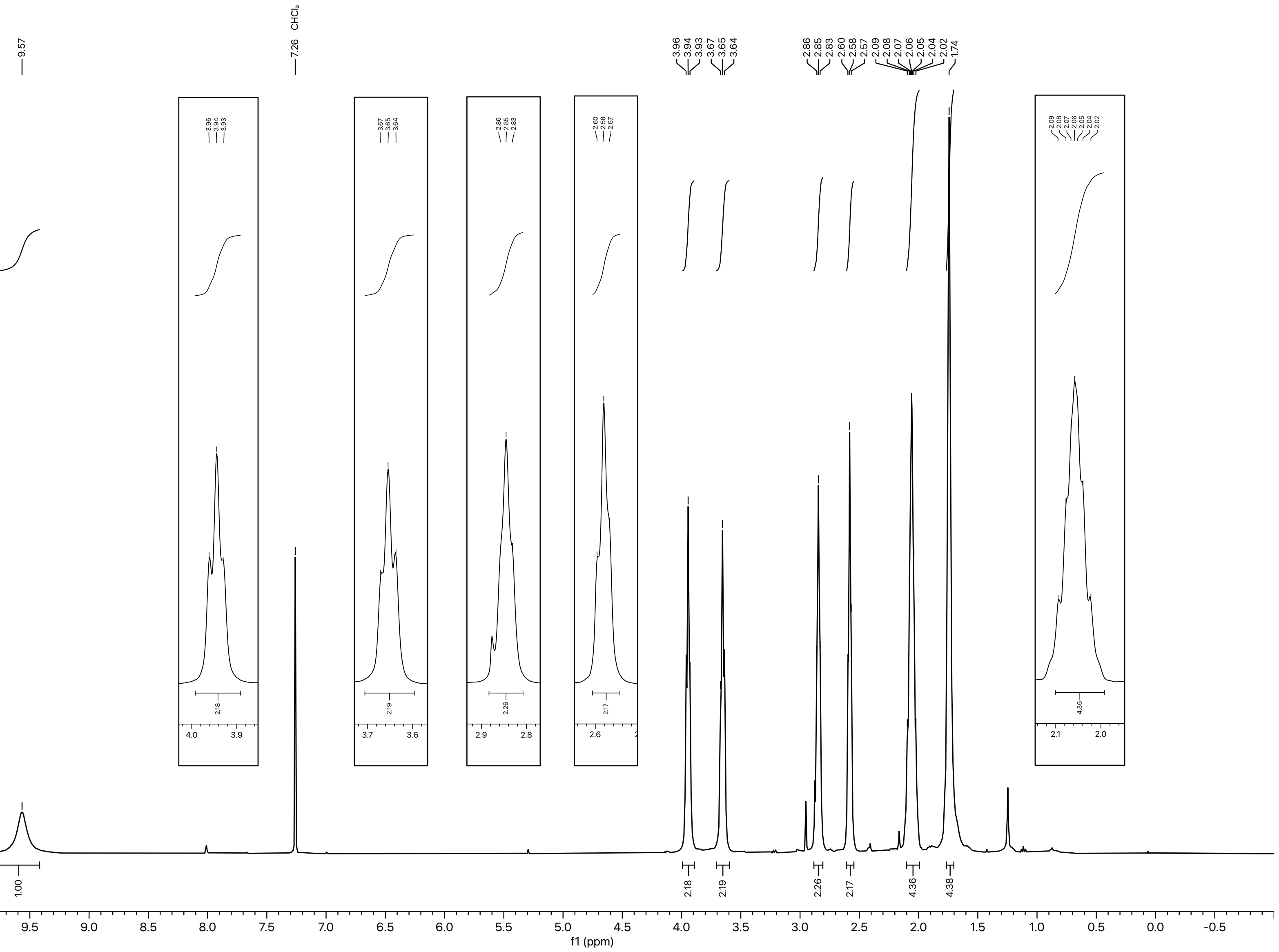

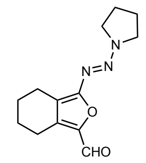

2-triazenyl-furan-36
^{13}C NMR
$CDCl_3$, 100 MHz

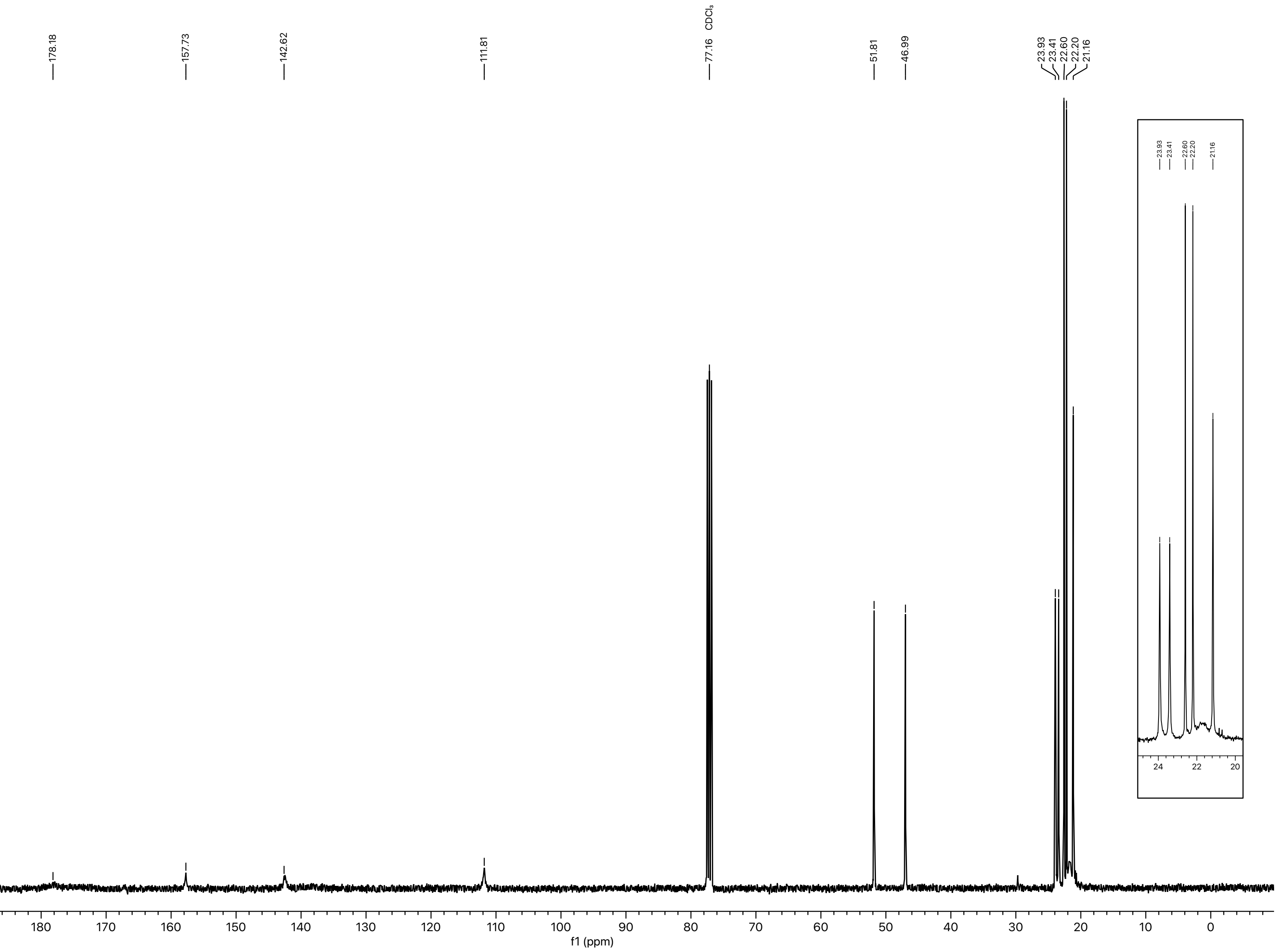

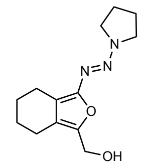

2-triazenyl-furan-37
^{1}H NMR
$CDCl_3$, 400 MHz

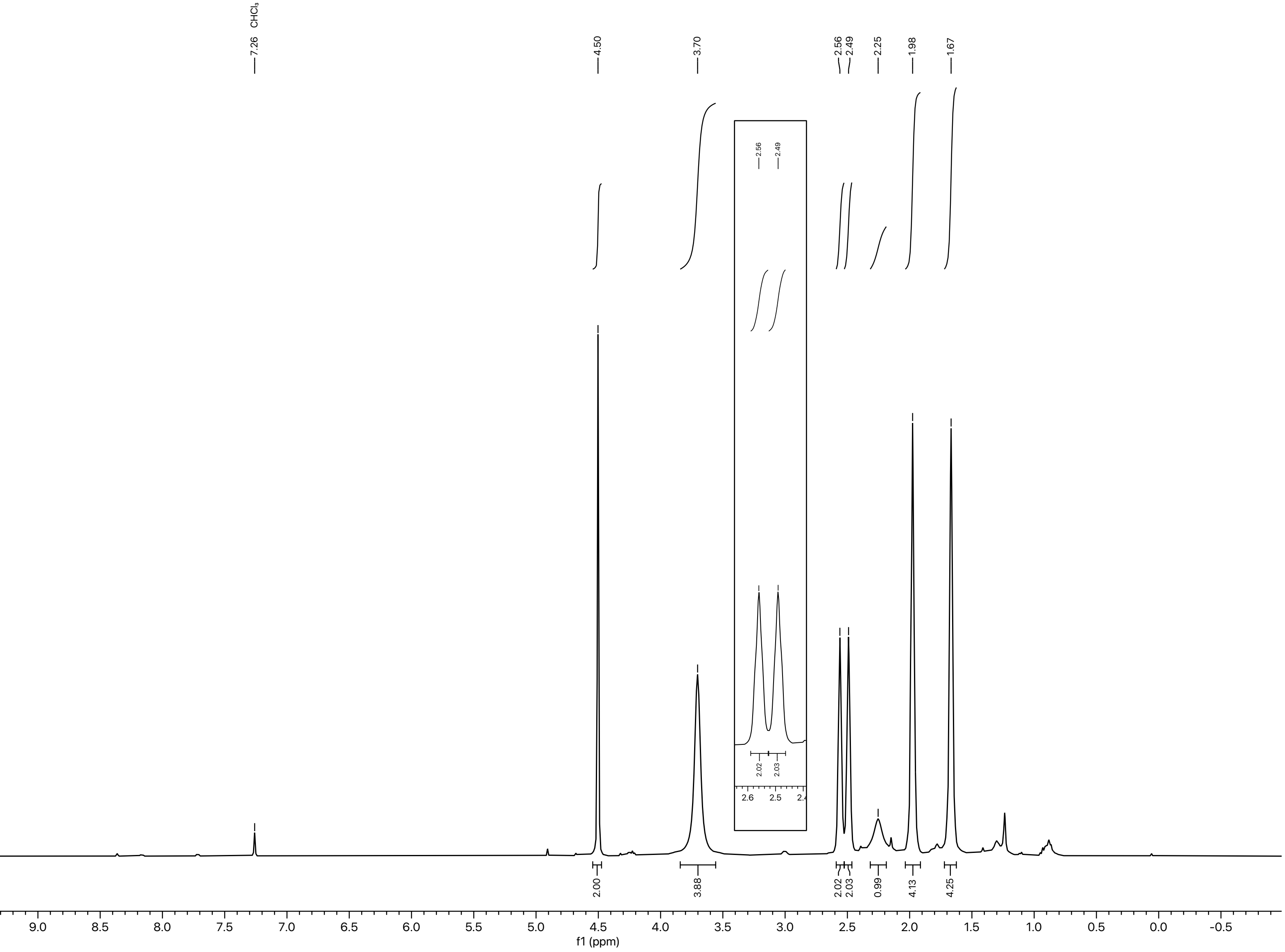

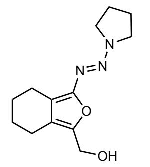

2-triazenyl-furan-37
^{13}C NMR
$CDCl_3$, 100 MHz

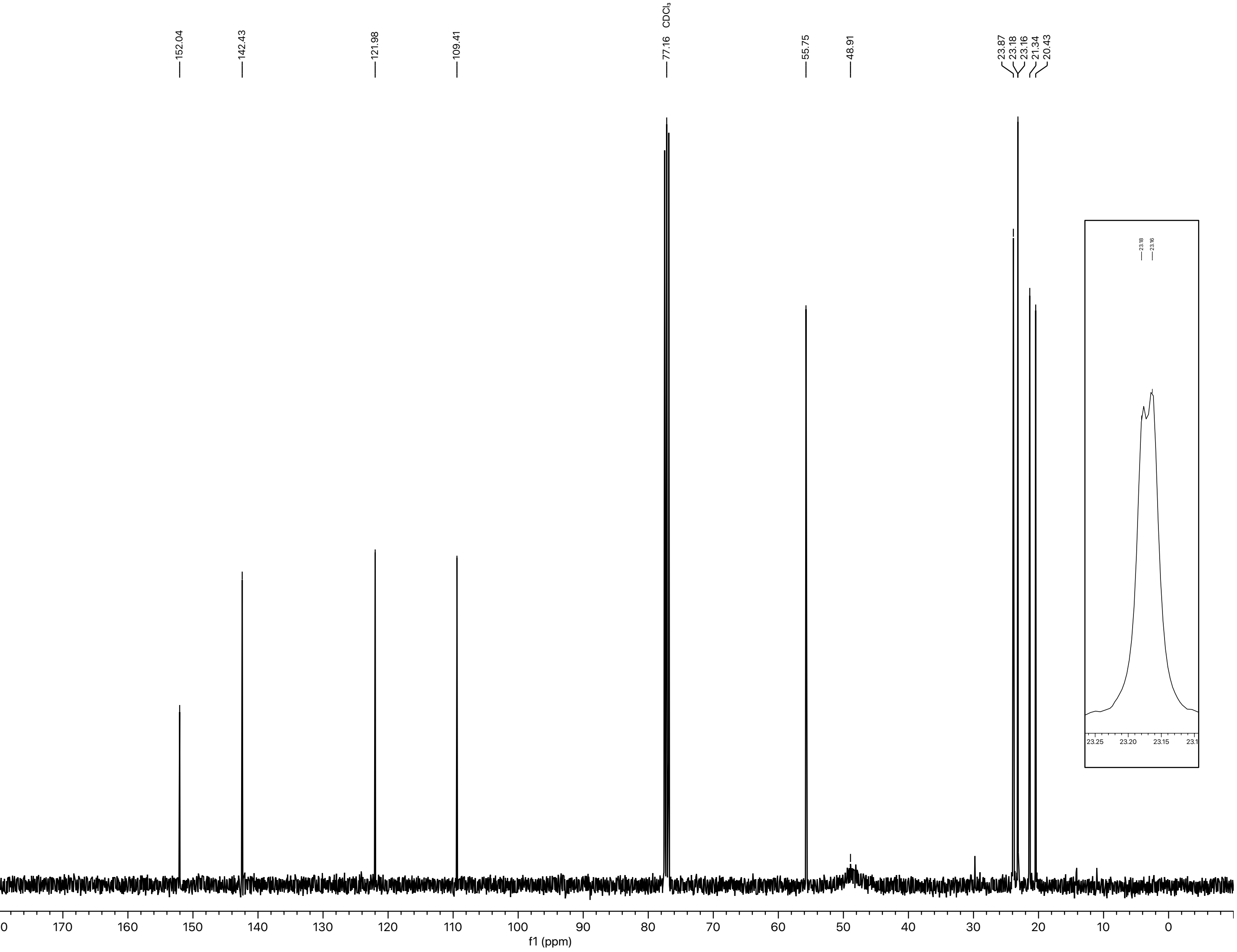

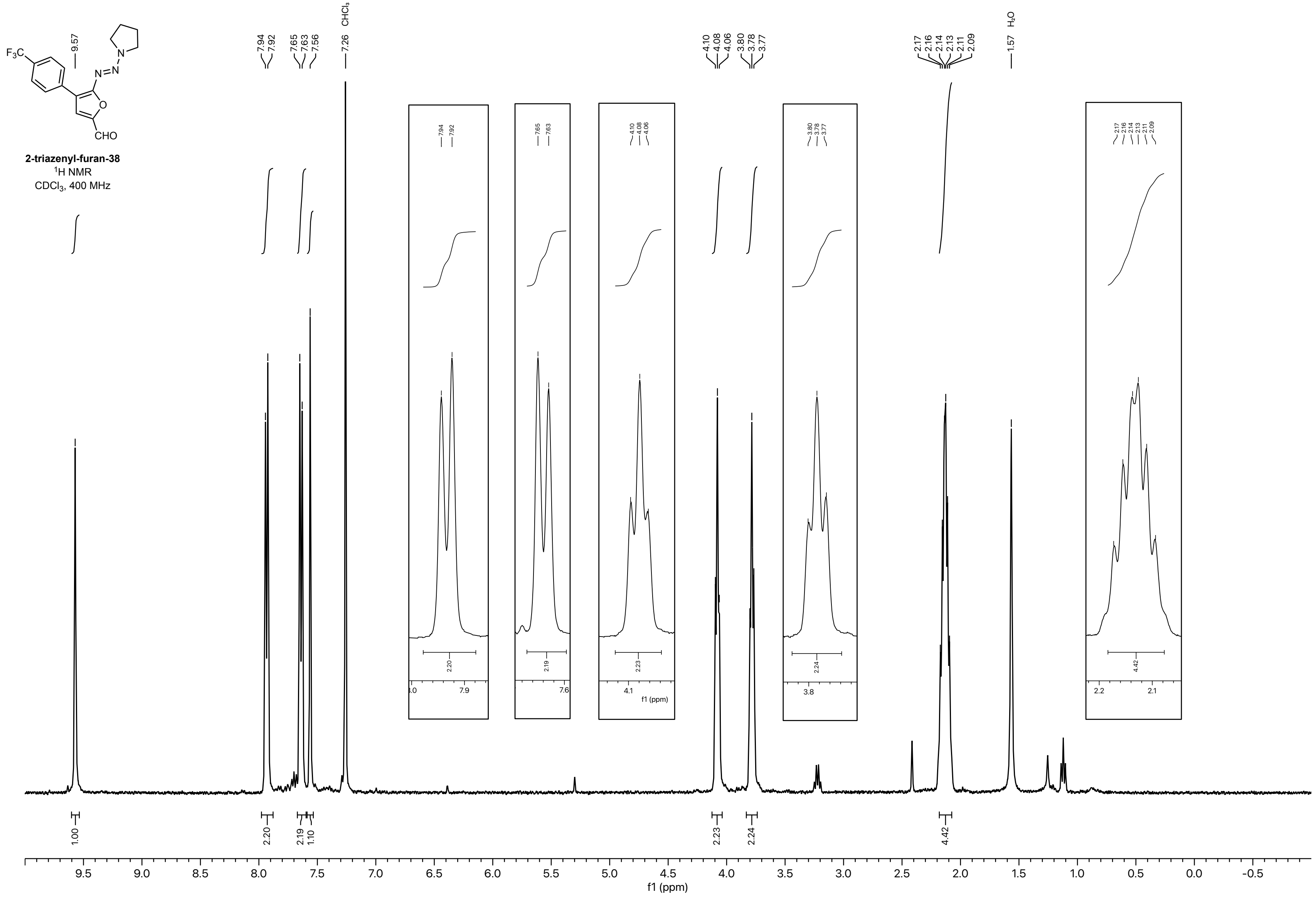

2-triazenyl-furan-38
^1H NMR
$CDCl_3$, 400 MHz
9.57
7.94
7.92
7.65
7.63
7.56
7.26 $CHCl_3$
4.10
4.08
4.06
3.80
3.78
3.77
2.17
2.16
2.14
2.13
2.11
2.09
1.57 H_2O
1.00
2.20
2.19
1.10
2.23
2.24
4.42
f1 (ppm)

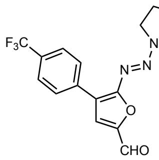

2-triazenyl-furan-38

^{13}C NMR

$CDCl_3$, 100 MHz

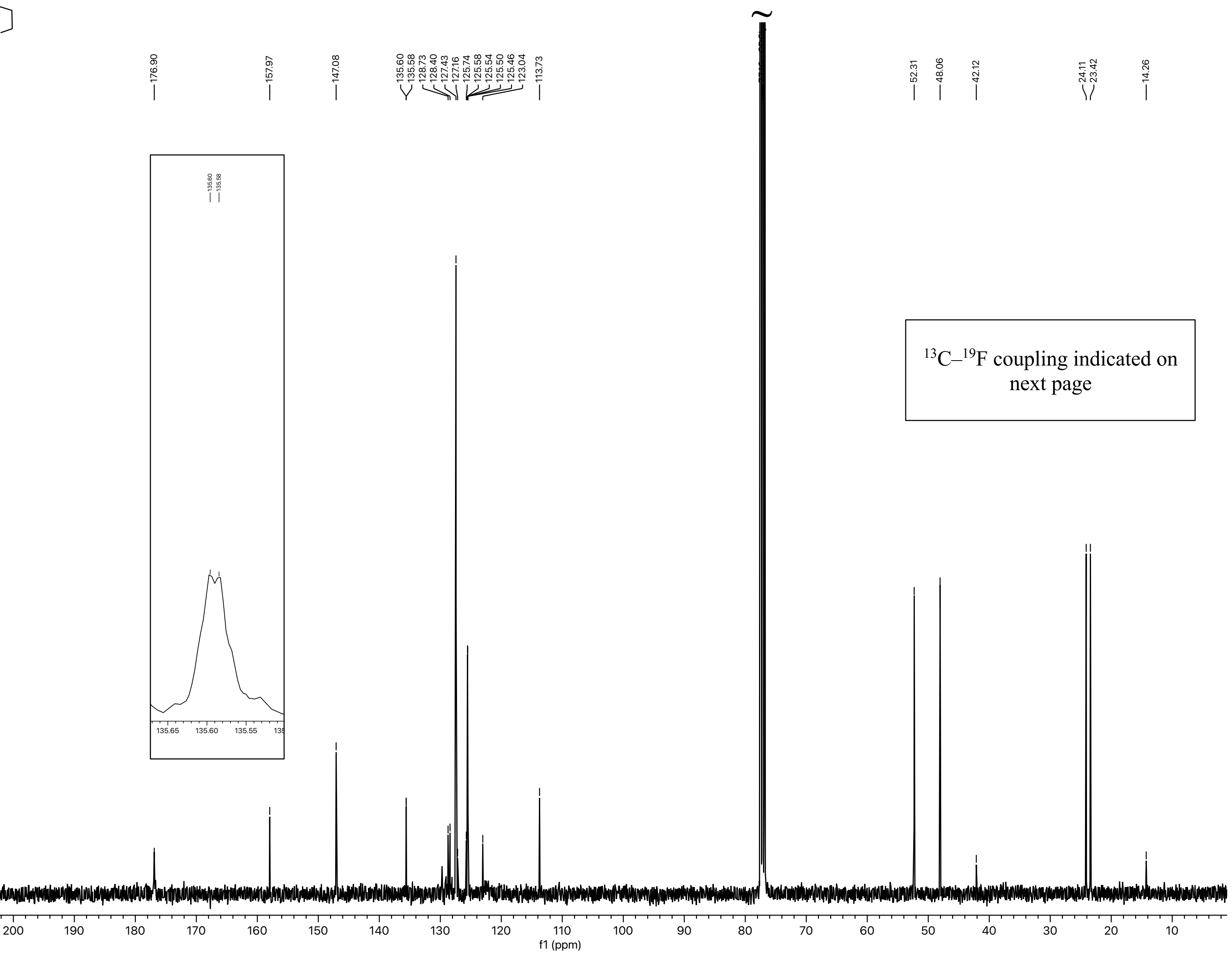

^{13}C–^{19}F coupling indicated on next page

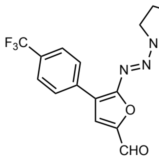

2-triazenyl-furan-38
^{13}C NMR
$CDCl_3$, 100 MHz

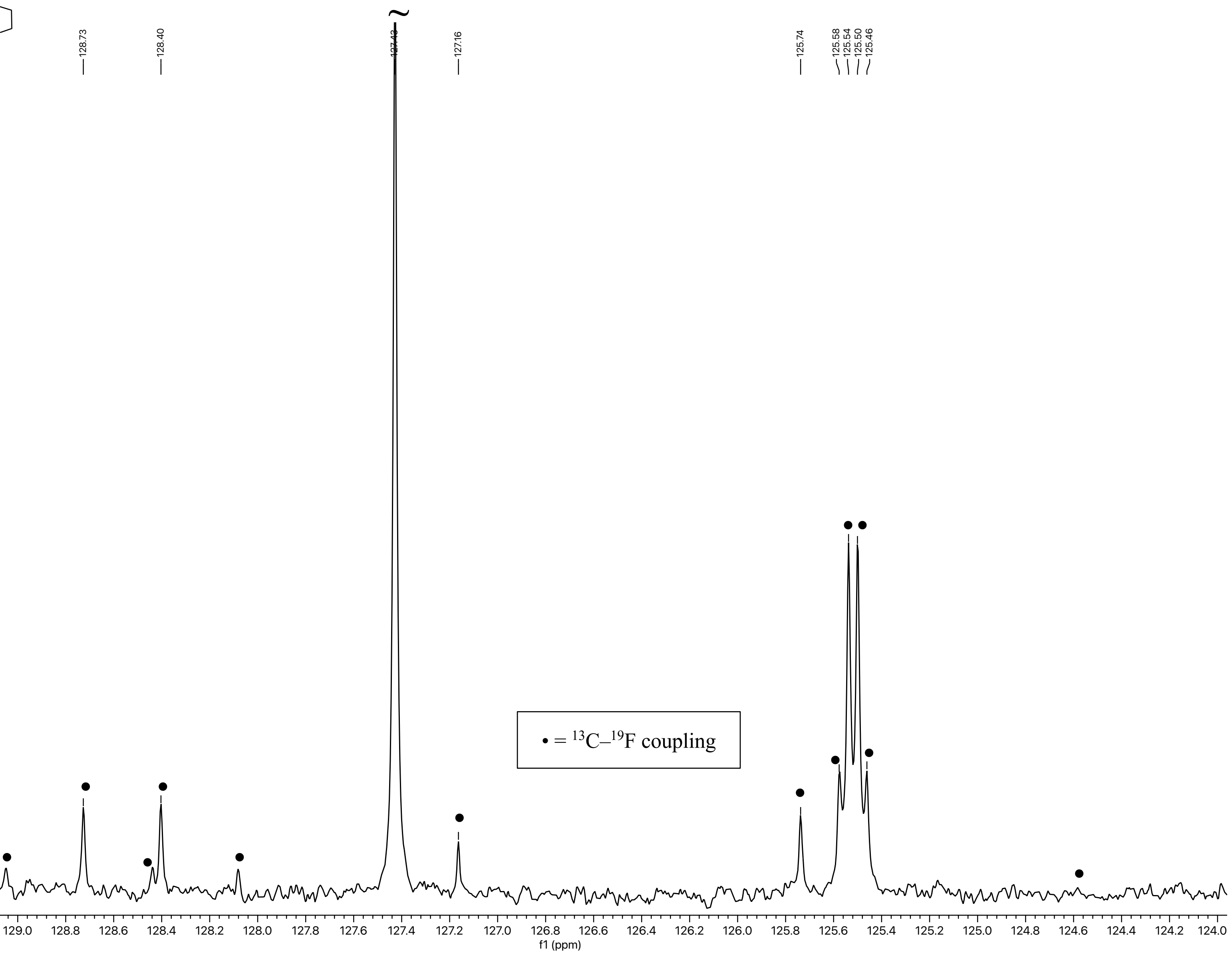

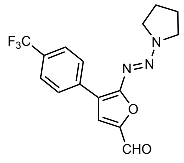

2-triazenyl-furan-38
^{19}F NMR
$CDCl_3$, 377 MHz

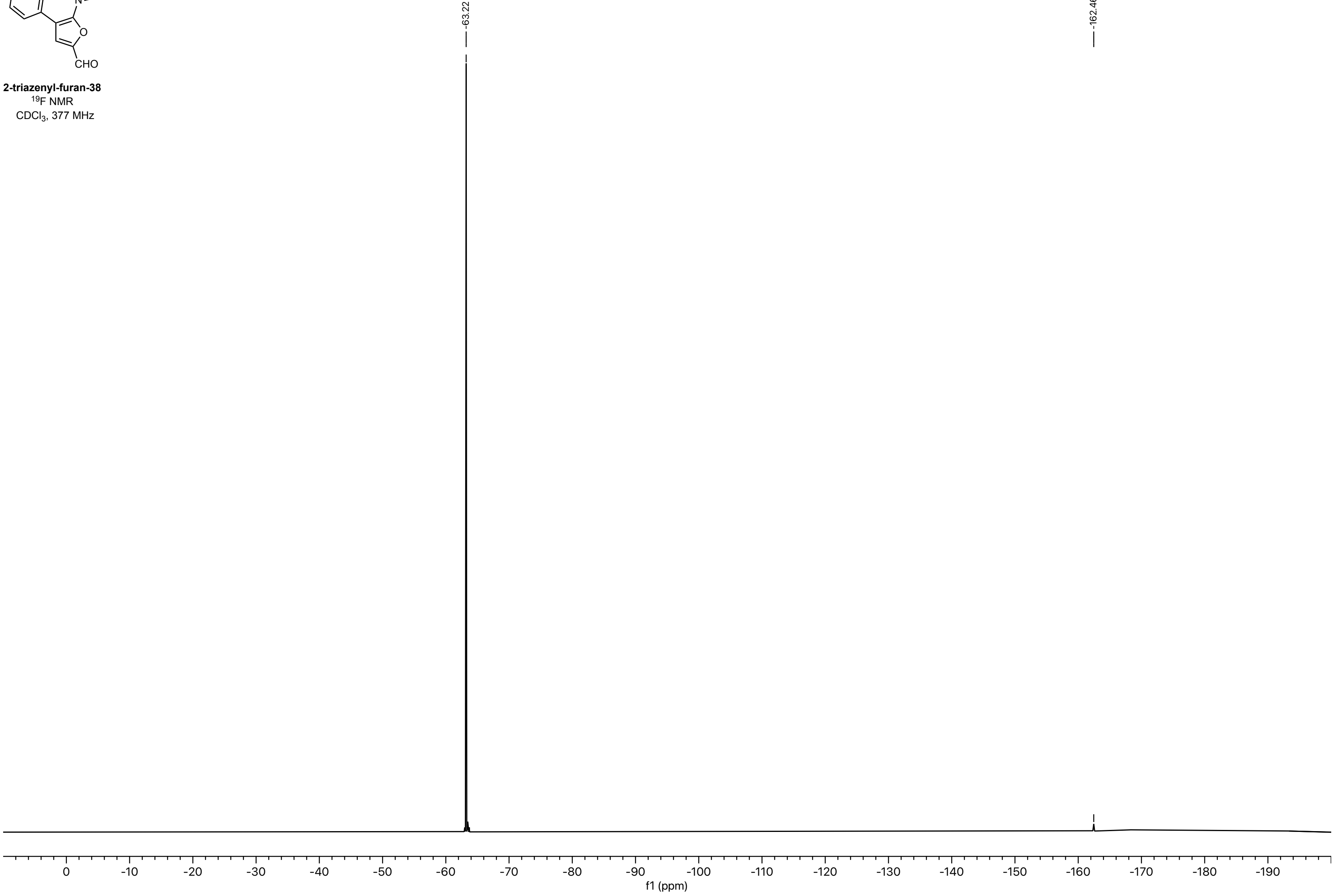

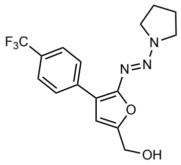

2-triazenyl-furan-39
^{1}H NMR
$CDCl_3$, 400 MHz

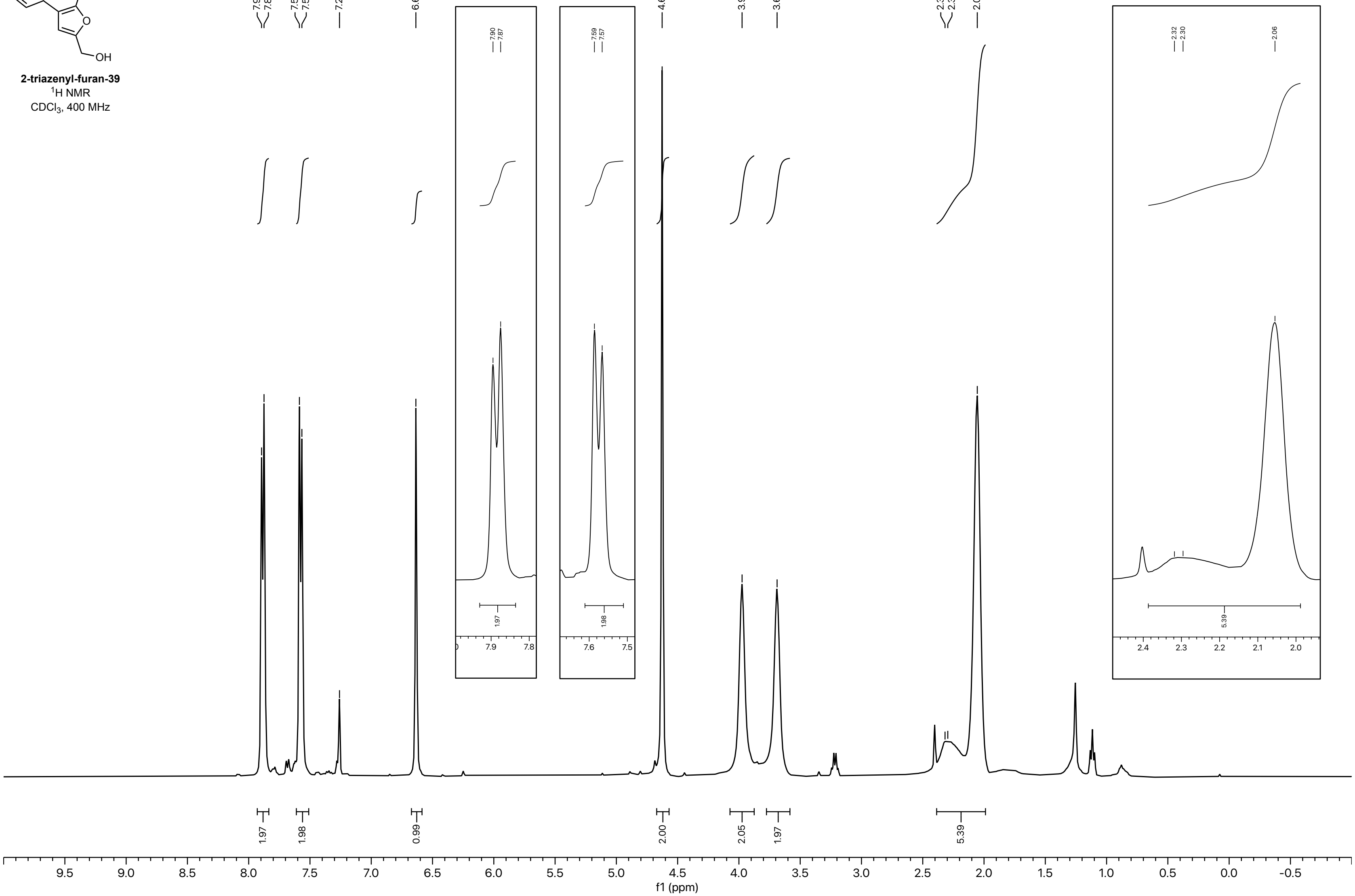

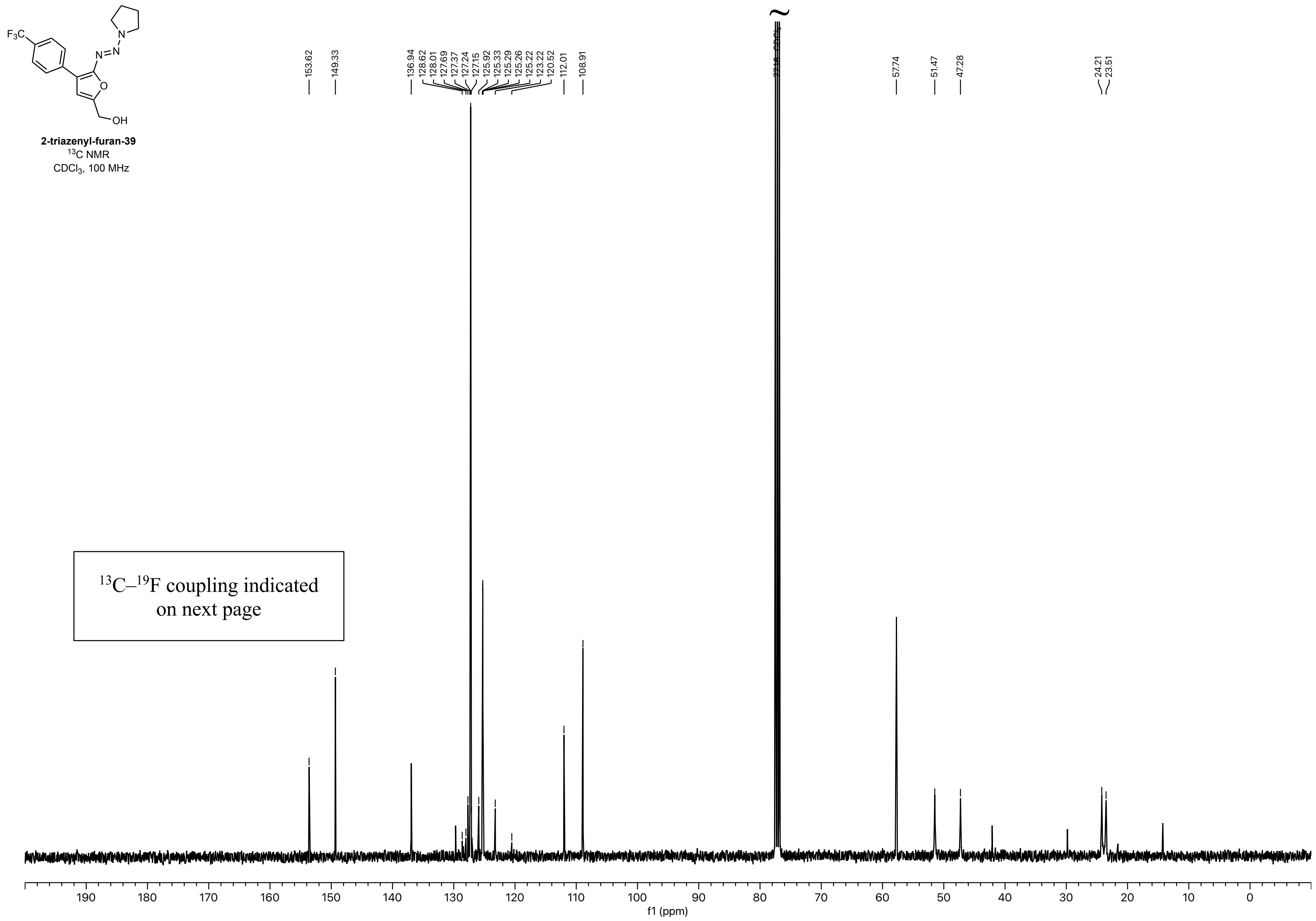
2-triazenyl-furan-39
^{13}C NMR
$CDCl_3$, 100 MHz
153.62
149.33
136.94
128.62
128.01
127.69
127.37
127.24
127.15
125.92
125.33
125.29
125.26
125.22
123.22
120.52
112.01
108.91
57.74
51.47
47.28
24.21
23.51
$^{13}C–^{19}F$ coupling indicated on next page
f1 (ppm)

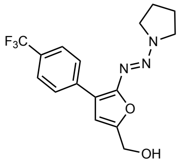

2-triazenyl-furan-39
^{13}C NMR
$CDCl_3$, 100 MHz

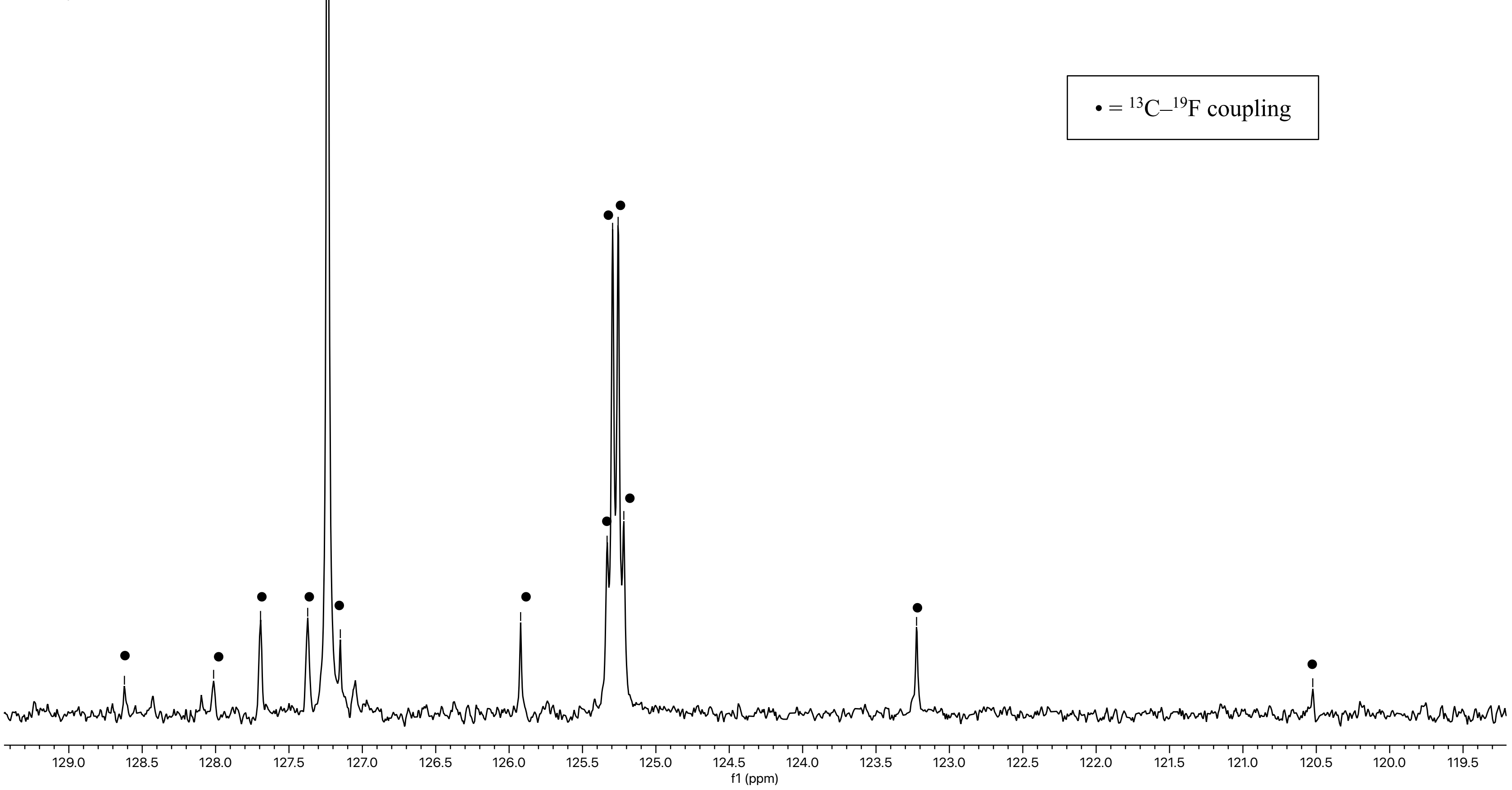

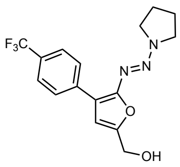

2-triazenyl-furan-39
^{19}F NMR
$CDCl_3$, 377 MHz

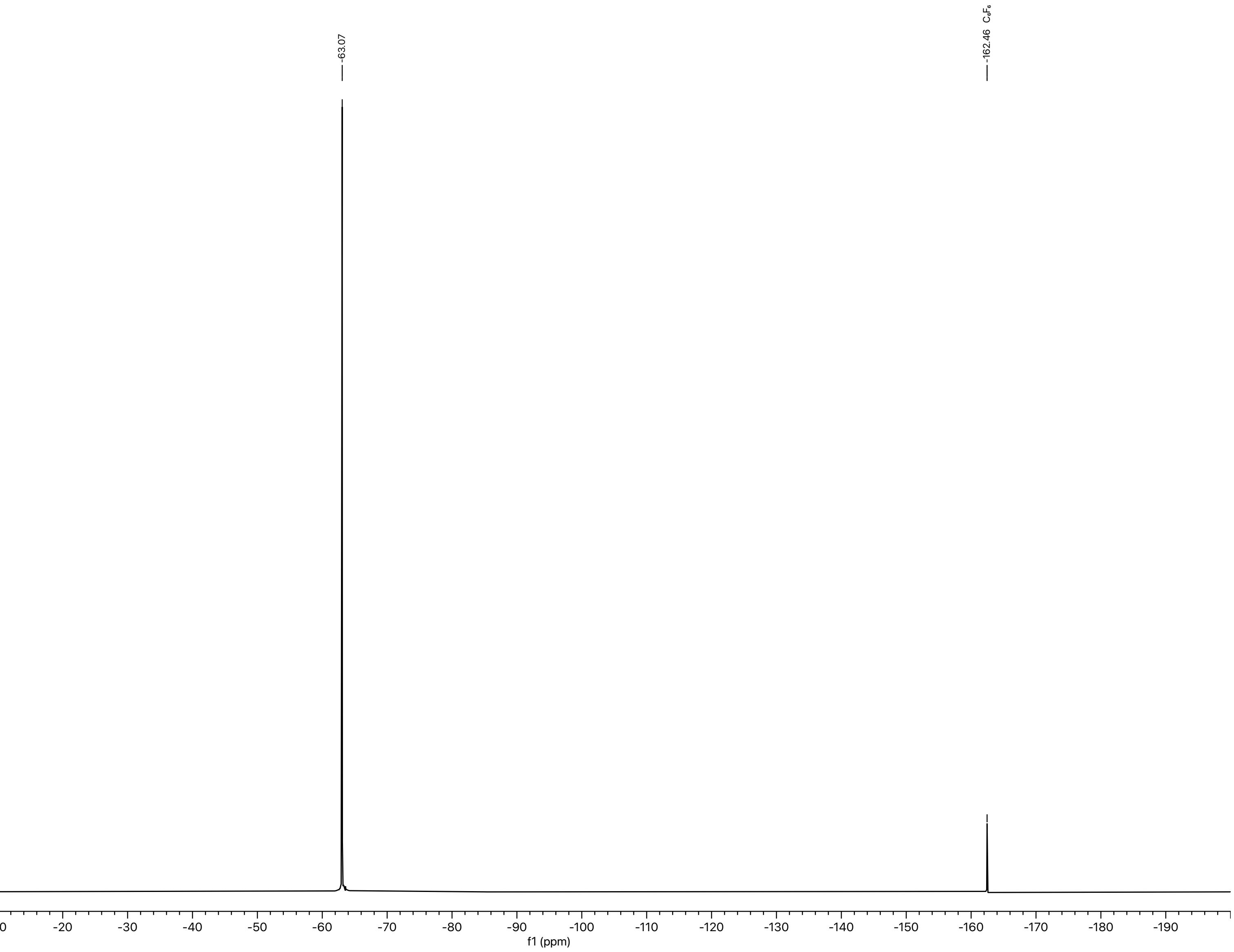

Synthesis of a 3-Triazenyl Furan

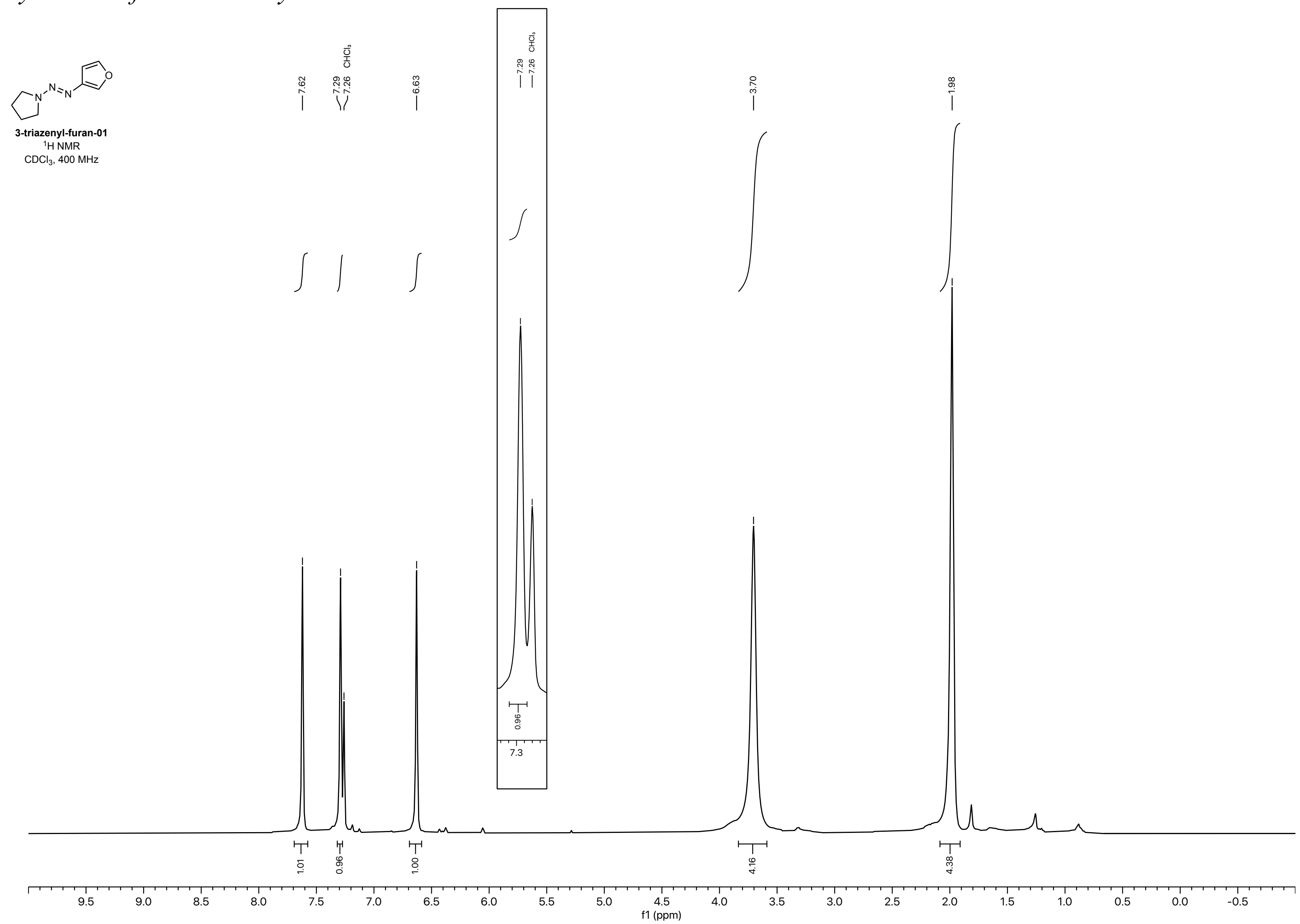

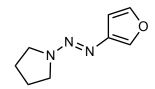

3-triazenyl-furan-01
^{13}C NMR
$CDCl_3$, 100 MHz

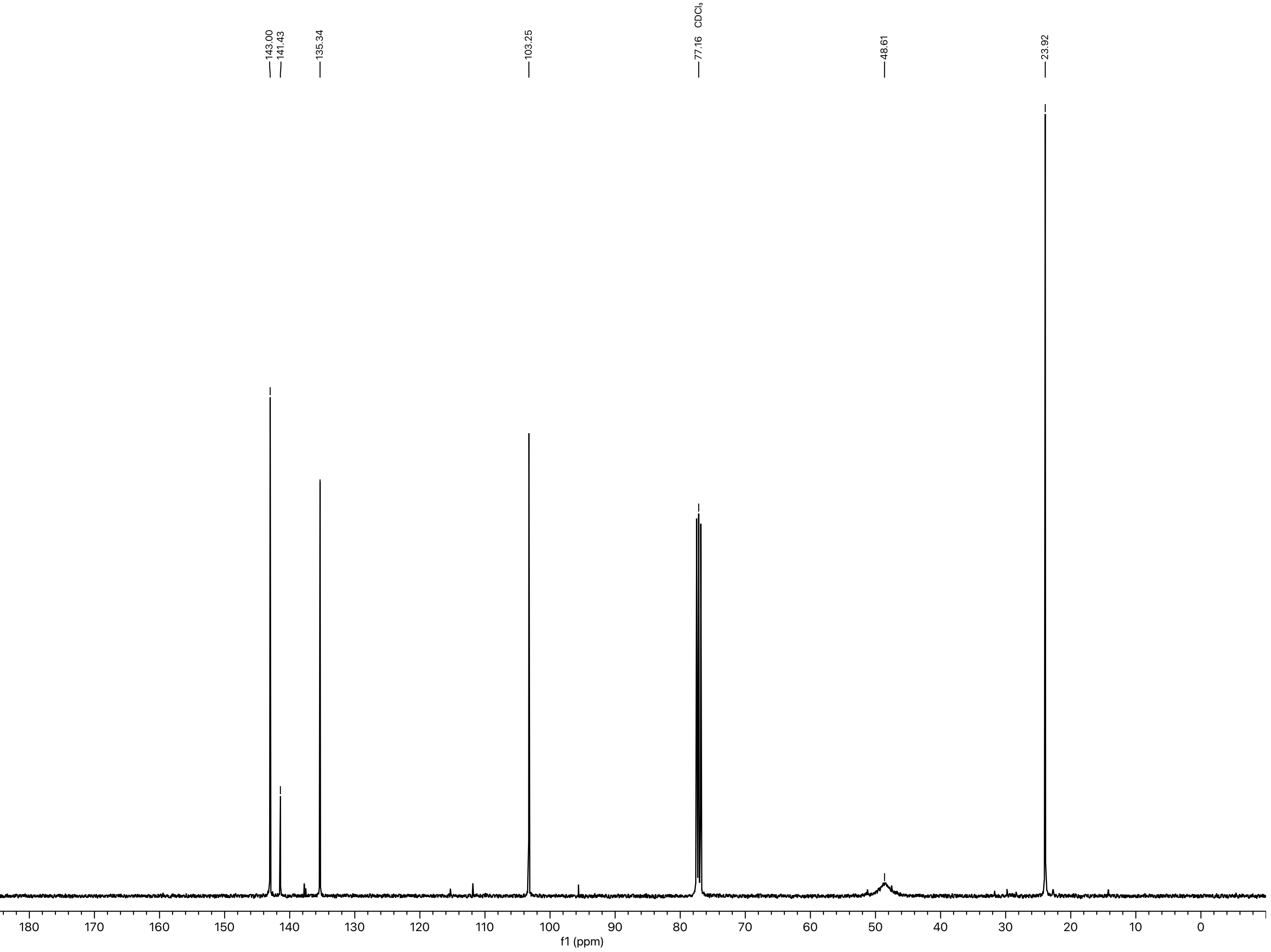

Mechanistic Experiment

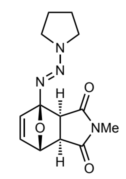

***exo*-int-01**
^{1}H NMR
CD_2Cl_2, 400 MHz

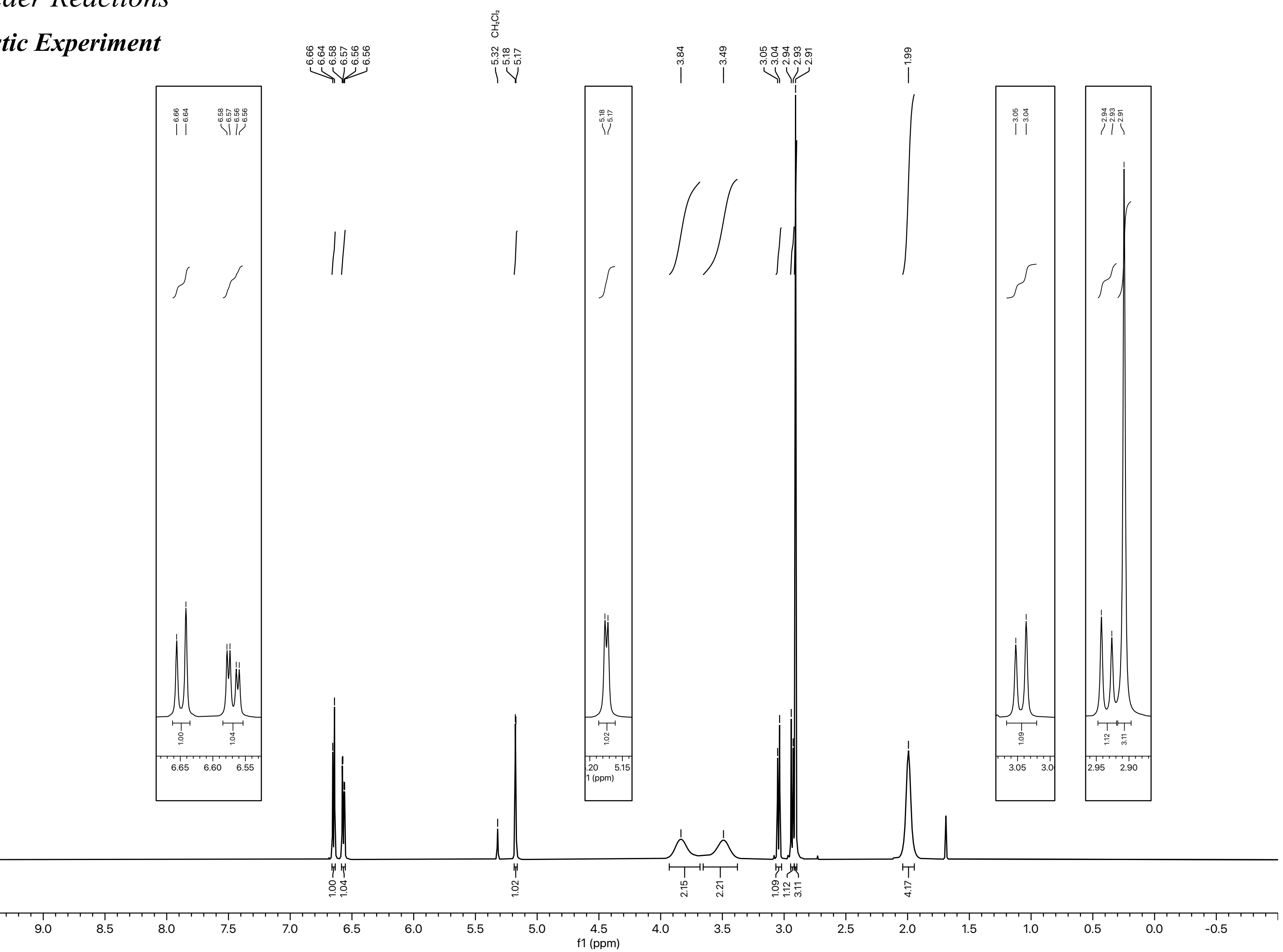

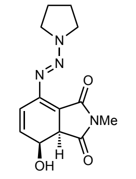

***exo*-int-02**
^{1}H NMR
$CDCl_3$, 400 MHz

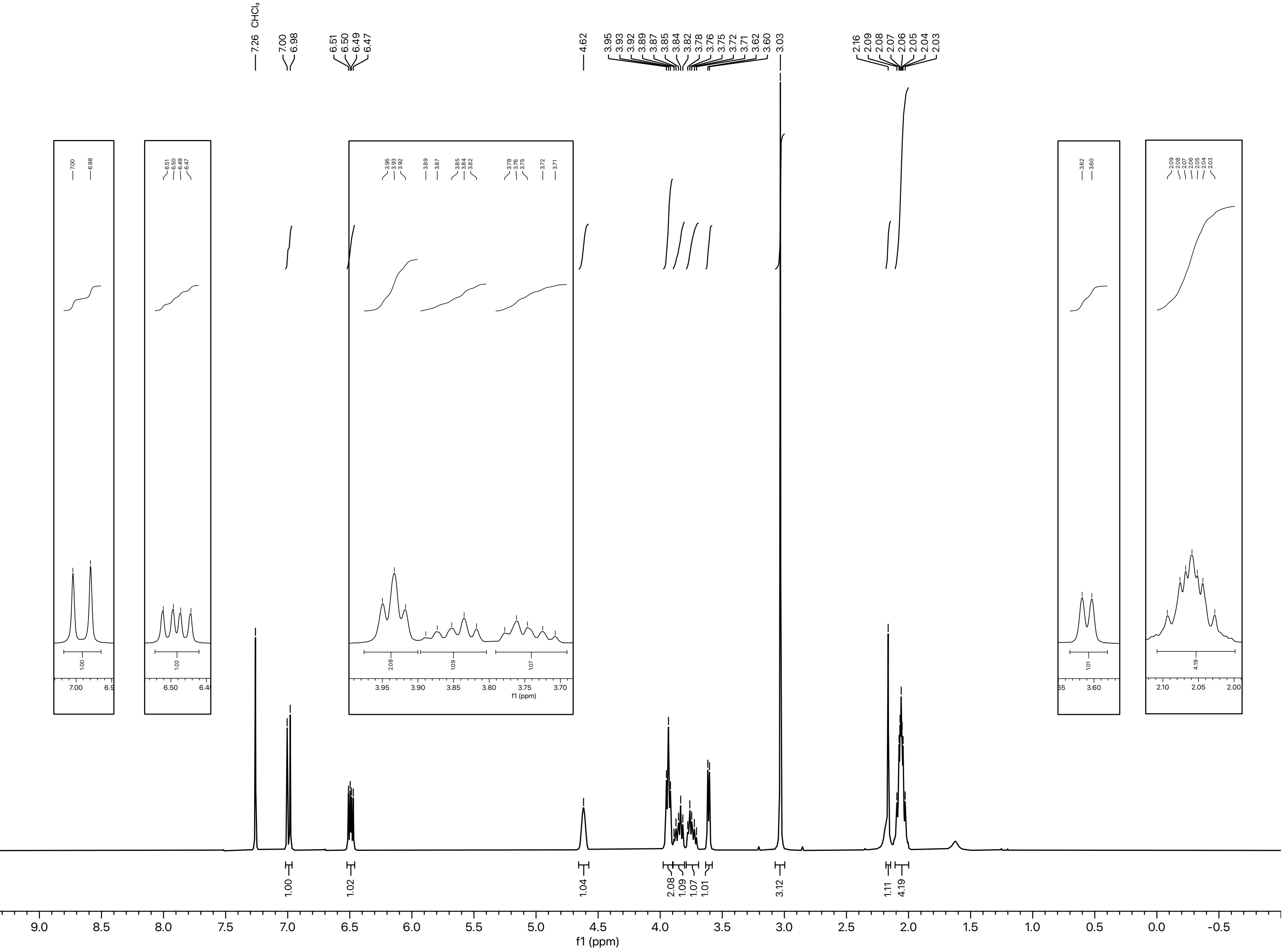

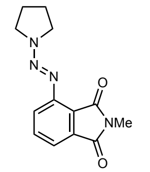

7
^{1}H NMR
$CDCl_3$, 400 MHz

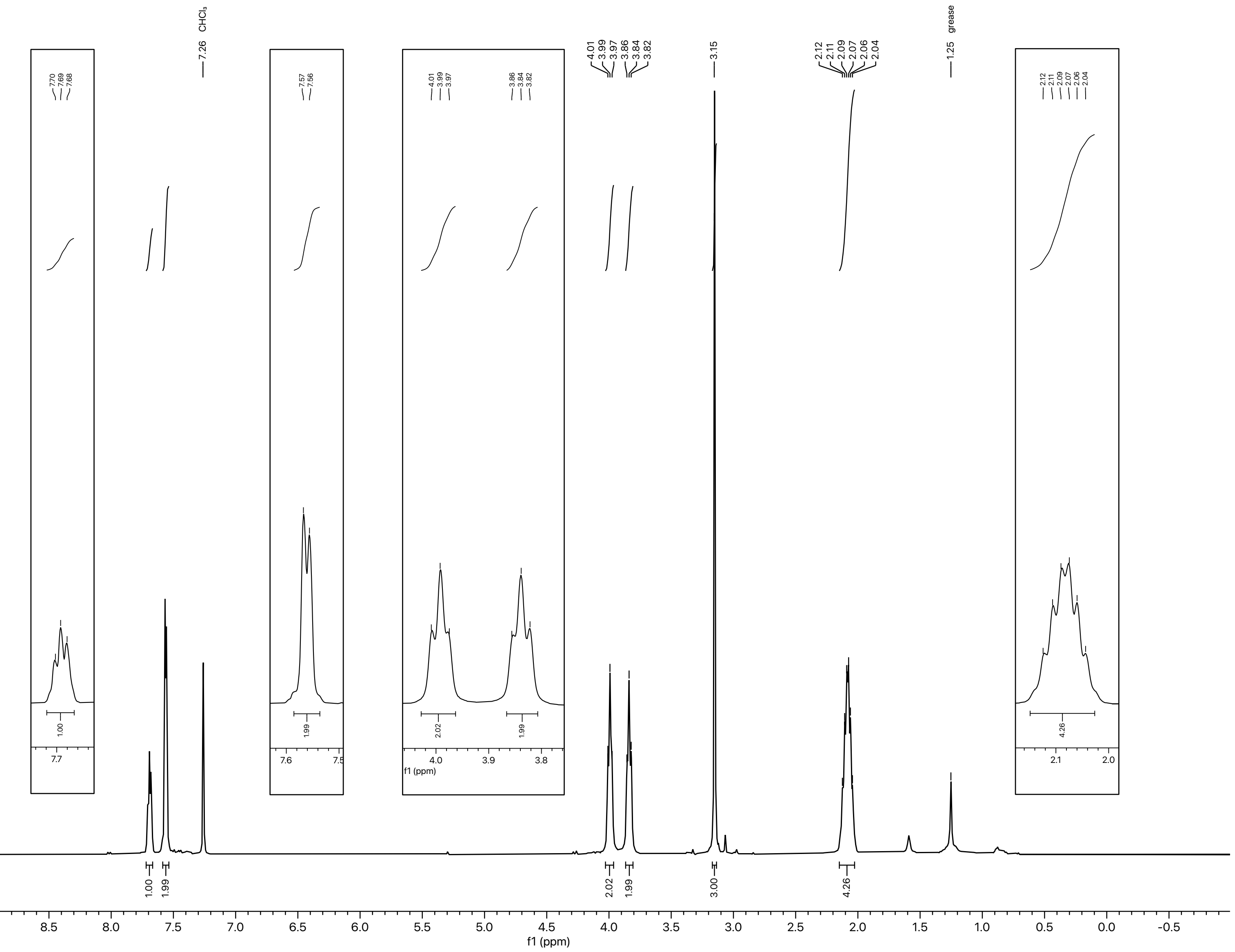

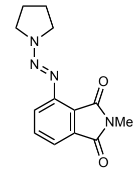

7
^{13}C NMR
$CDCl_3$, 150 MHz

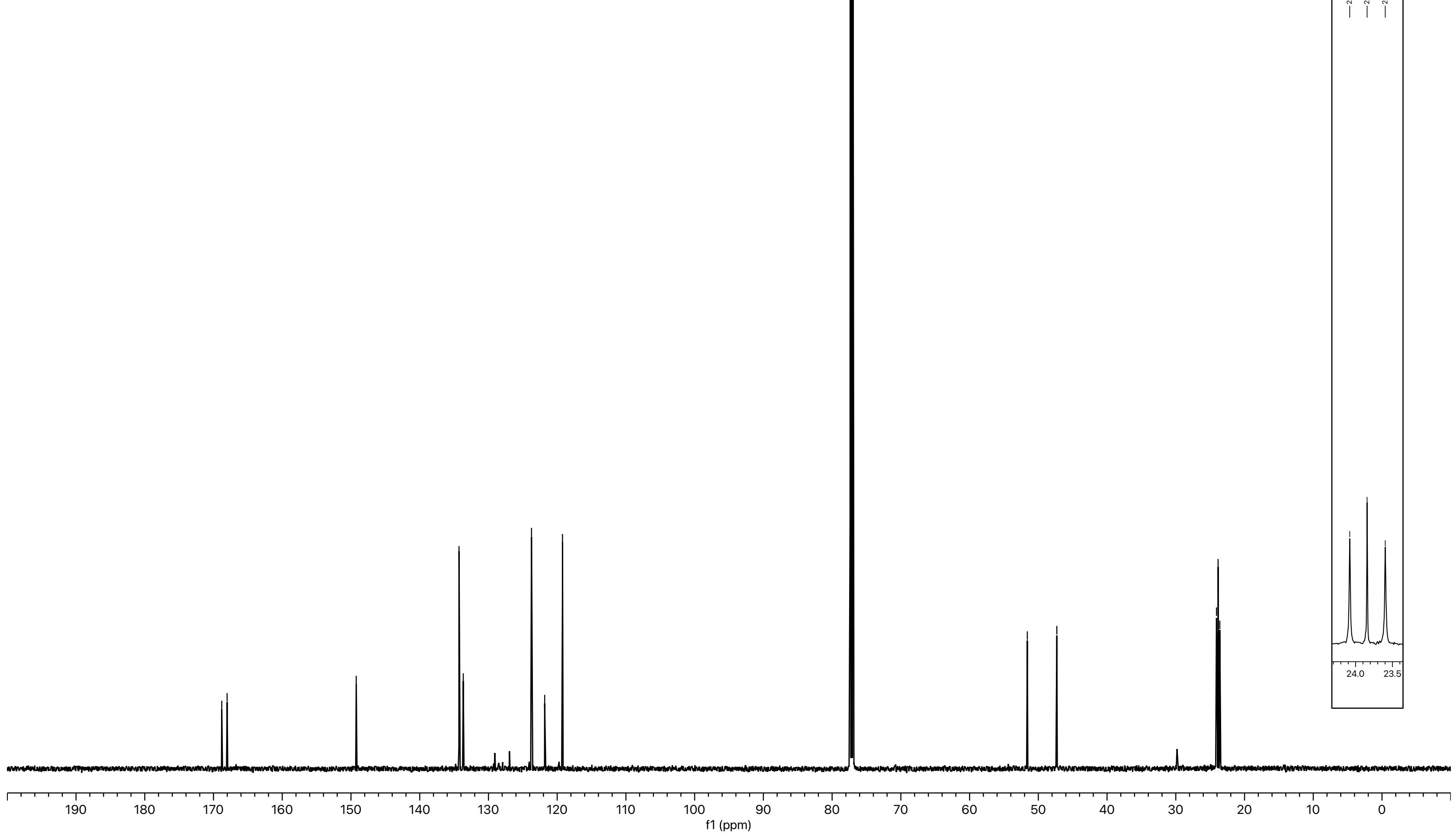

N-Phenylmaleimide as Dienophile

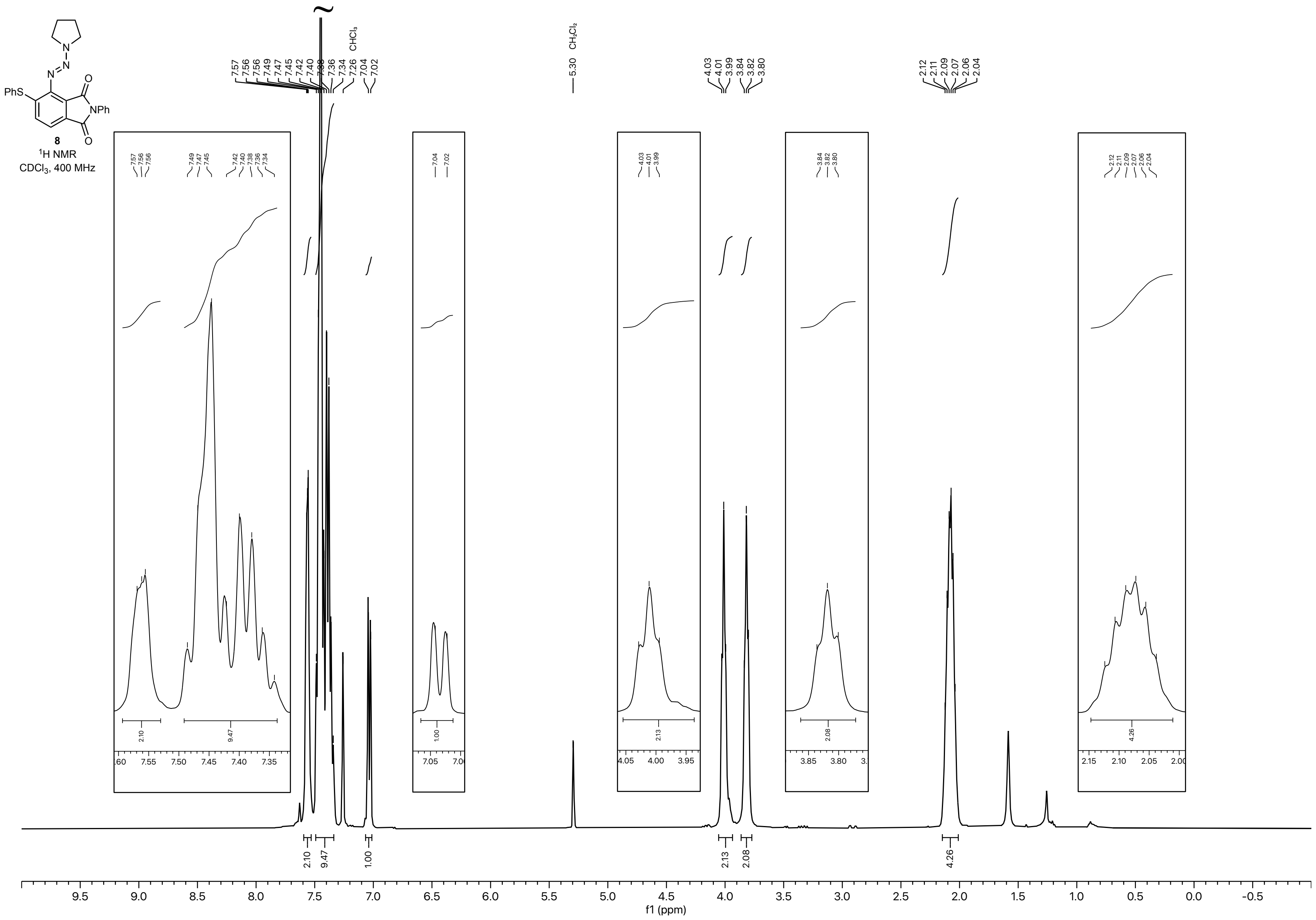

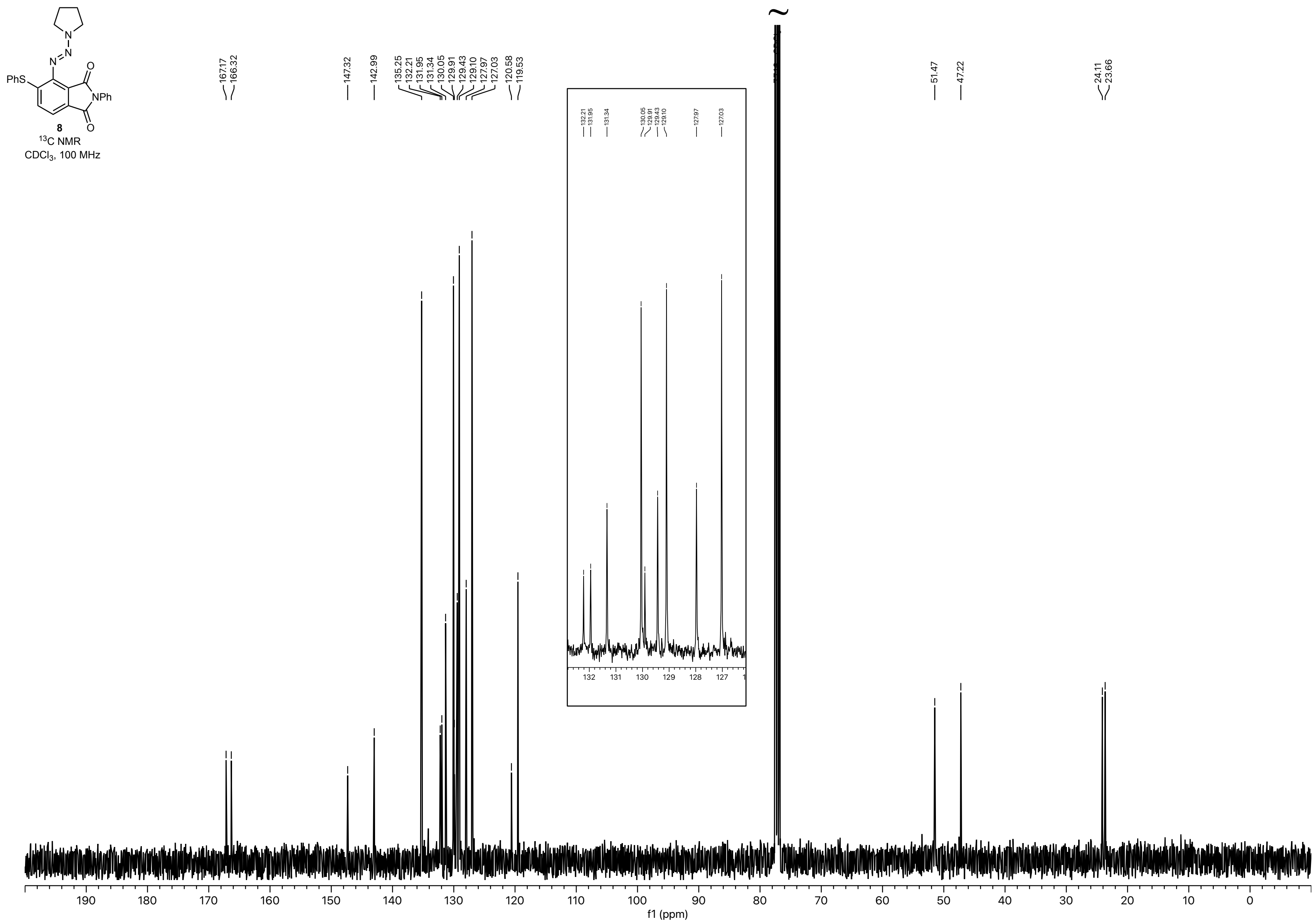
PhS
NPh
8
^{13}C NMR
$CDCl_3$, 100 MHz
167.17
166.32
147.32
142.99
135.25
132.21
131.95
131.34
130.05
129.91
129.43
129.10
127.97
127.03
120.58
119.53
51.47
47.22
24.11
23.66
f1 (ppm)

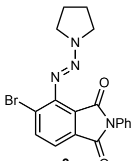

9

^{1}H NMR

$CDCl_3$, 400 MHz

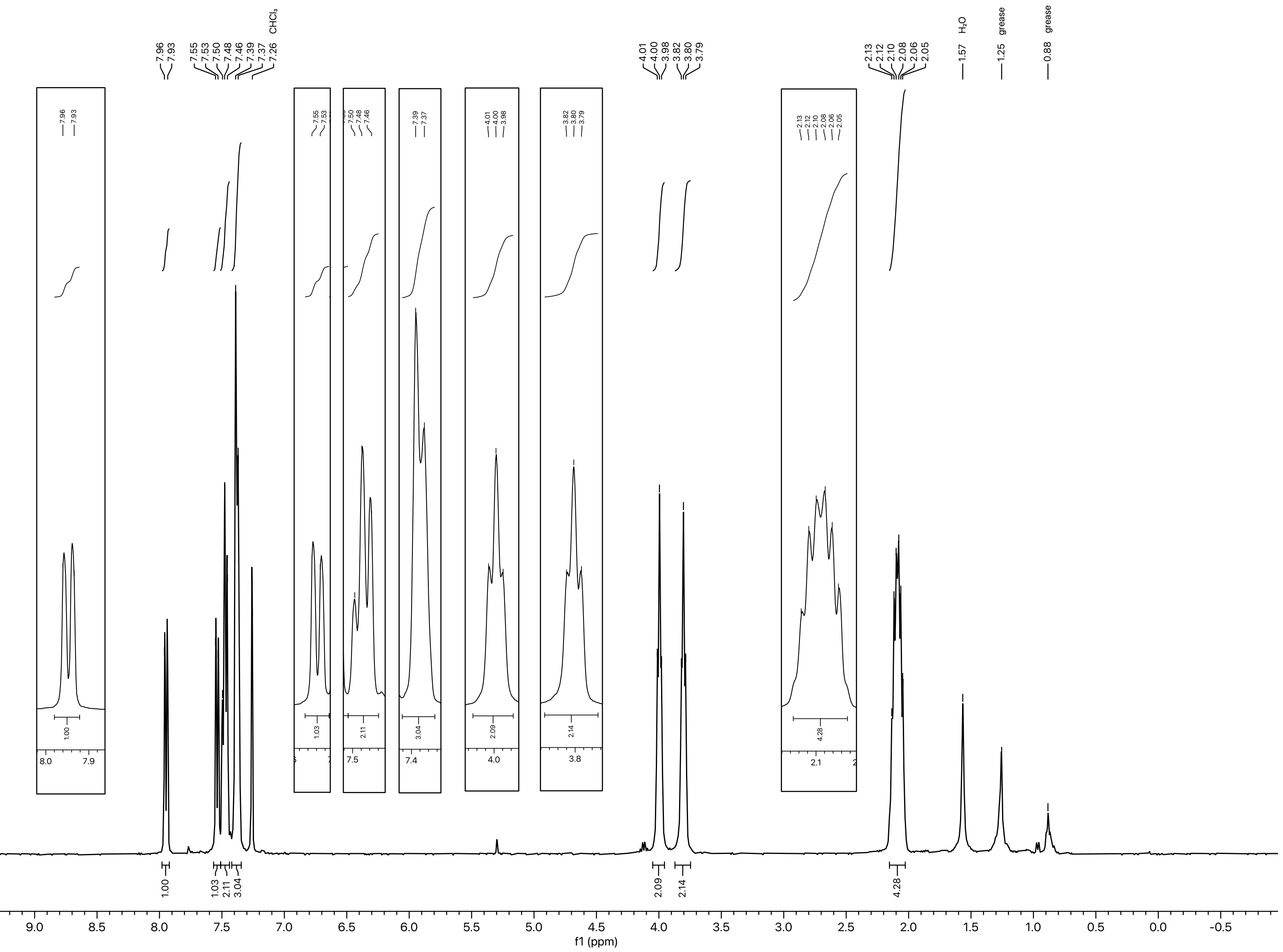

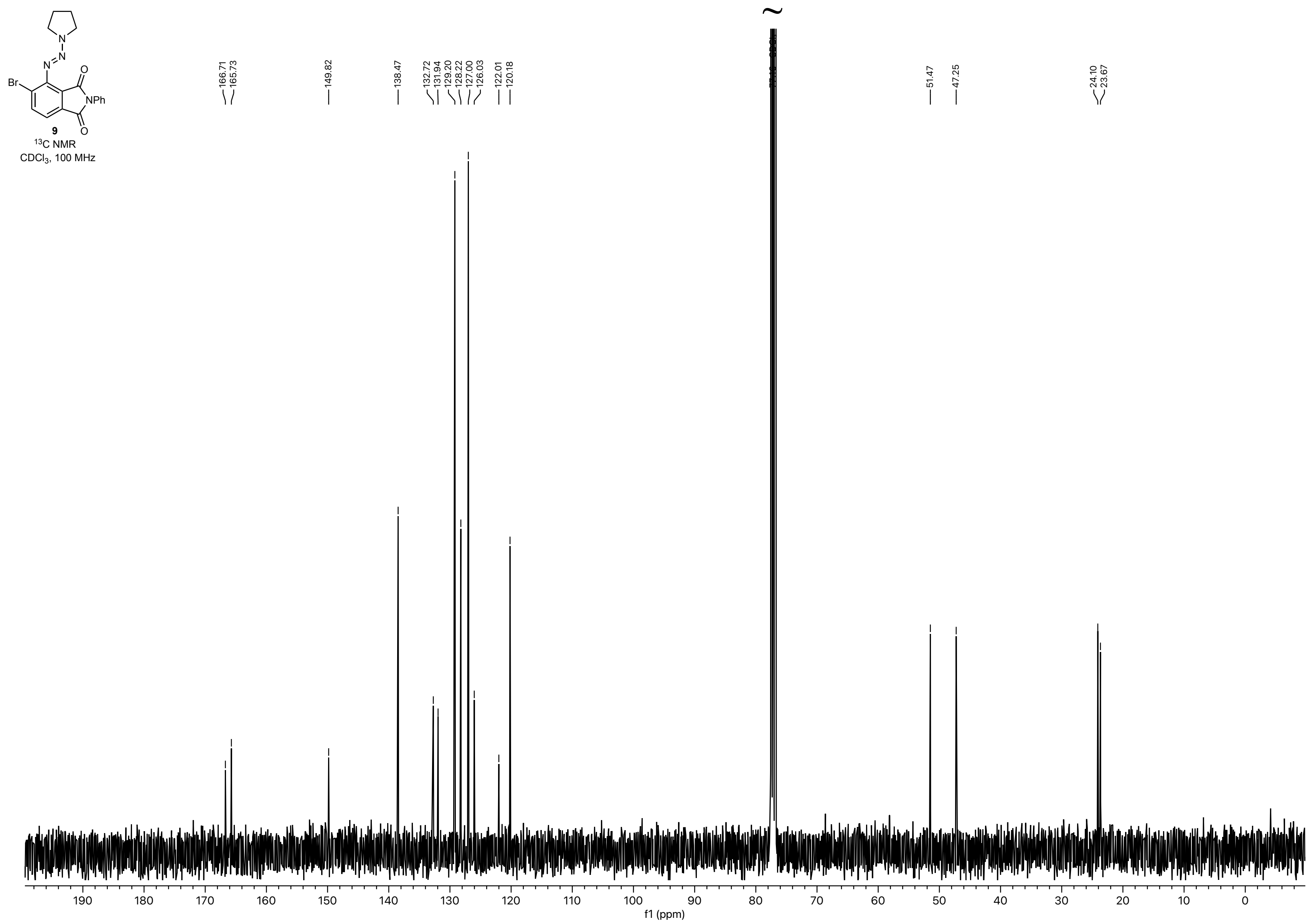

Br
N
N
N
O
O
NPh
9
^{13}C NMR
$CDCl_3$, 100 MHz
166.71
165.73
149.82
138.47
132.72
131.94
129.20
128.22
127.00
126.03
122.01
120.18
51.47
47.25
24.10
23.67
190 180 170 160 150 140 130 120 110 100 90 80 70 60 50 40 30 20 10 0
f1 (ppm)

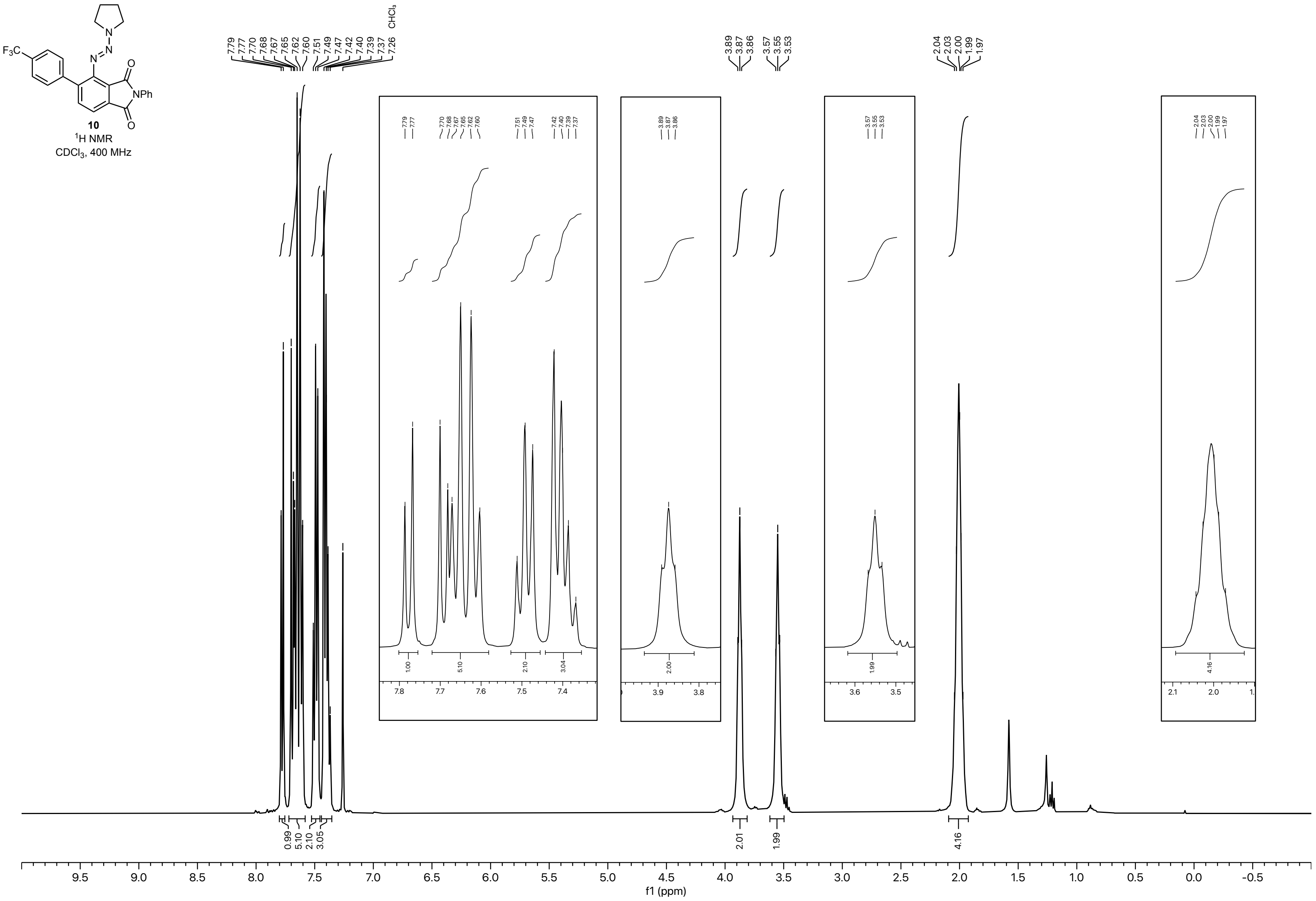

F$_3$C
NPh
10
^{1}H NMR
$CDCl_3$, 400 MHz
7.79 7.77 7.70 7.68 7.67 7.65 7.62 7.60 7.51 7.49 7.47 7.42 7.40 7.39 7.37 7.26 CHCl$_3$
3.89 3.87 3.86 3.55 3.53
2.04 2.03 2.00 1.99 1.97
0.99 5.10 2.10 3.05
2.01
1.99
4.16
f1 (ppm)

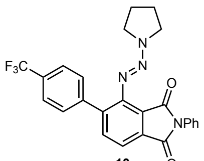

10

^{13}C NMR

$CDCl_3$, 100 MHz

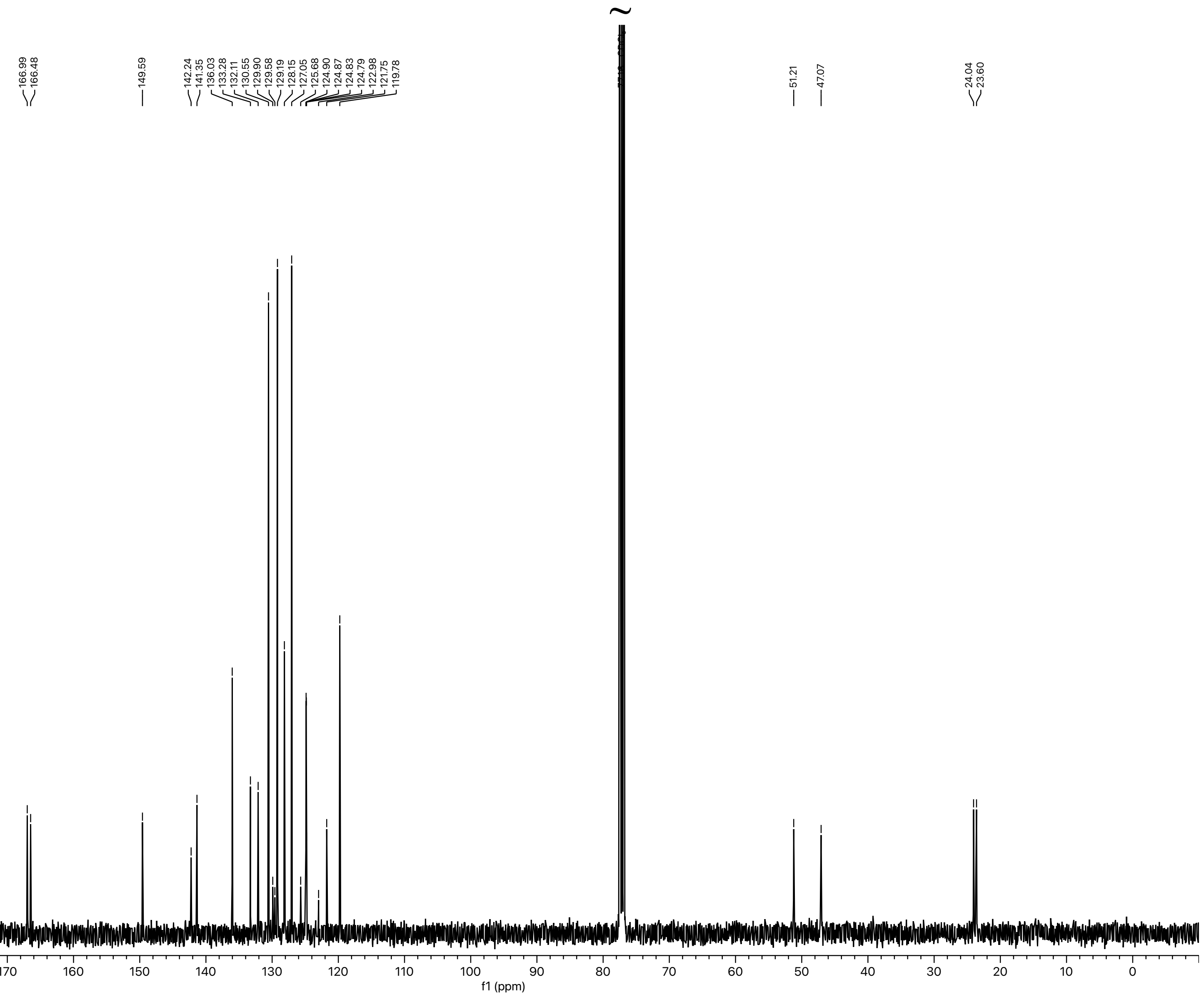

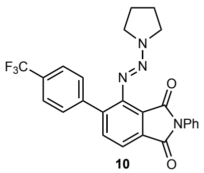

10

^{13}C NMR

$CDCl_3$, 100 MHz

• = ^{13}C–^{19}F coupling

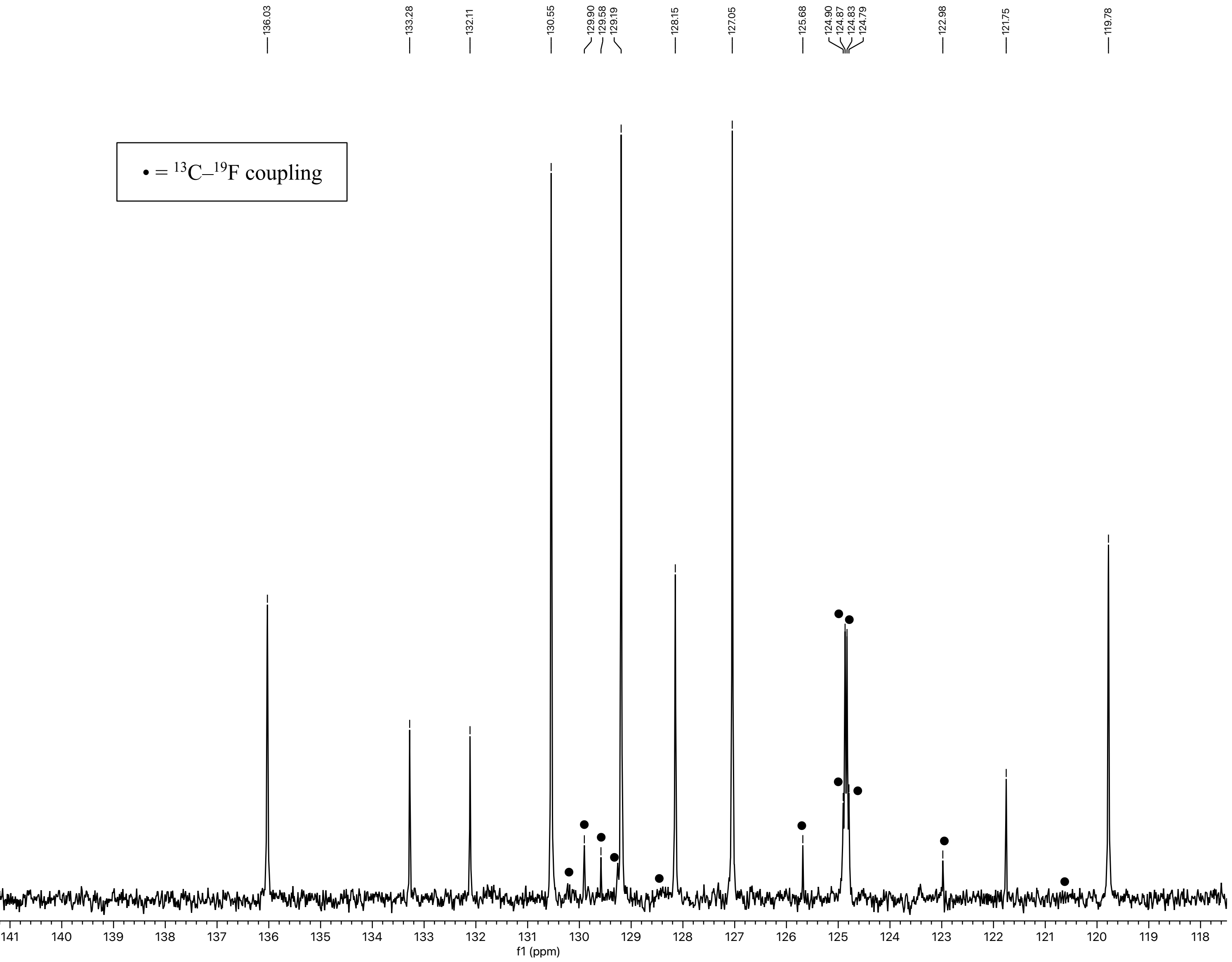

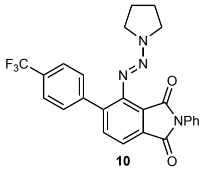

10
^{19}F NMR
$CDCl_3$, 377 MHz

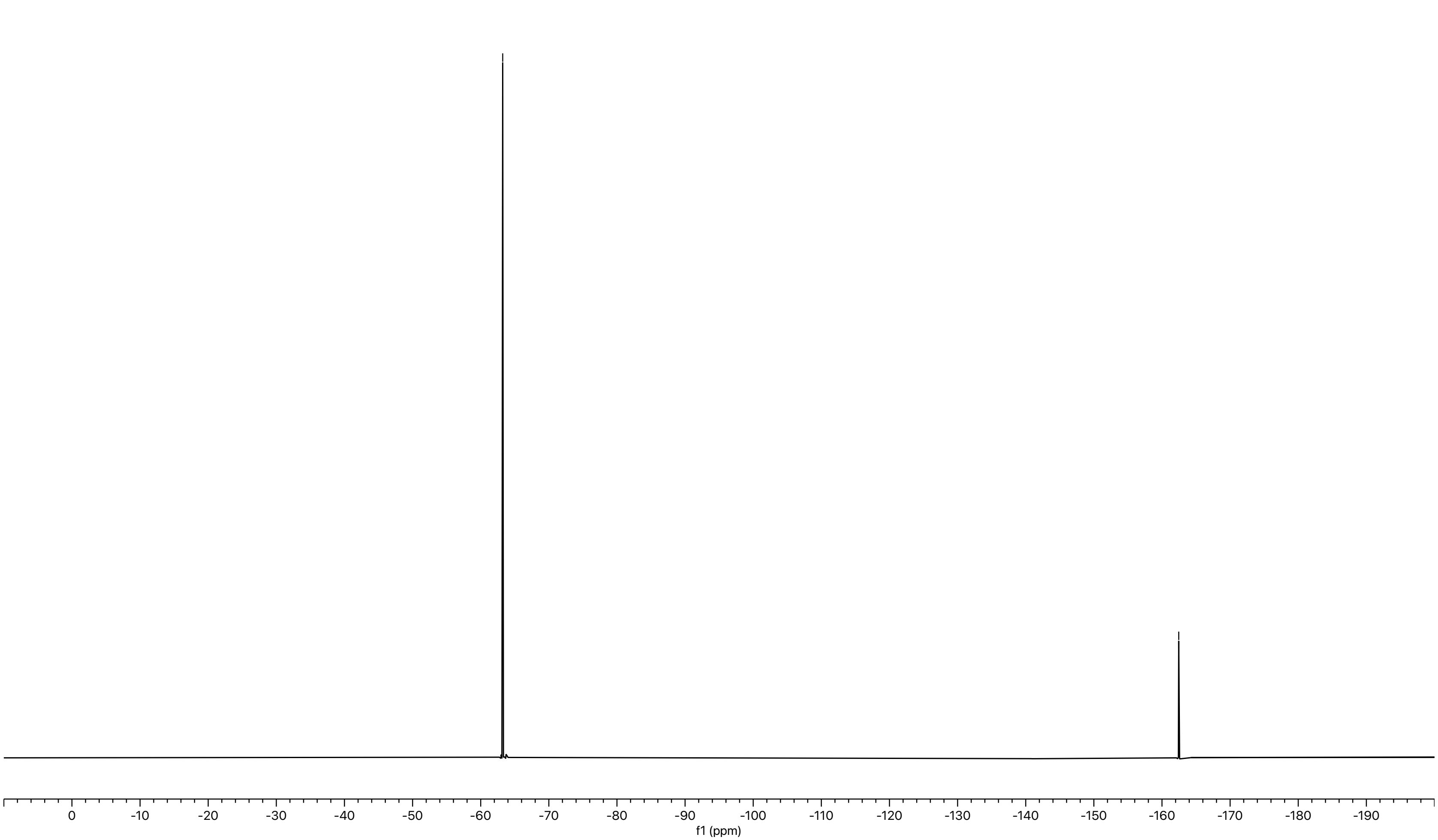

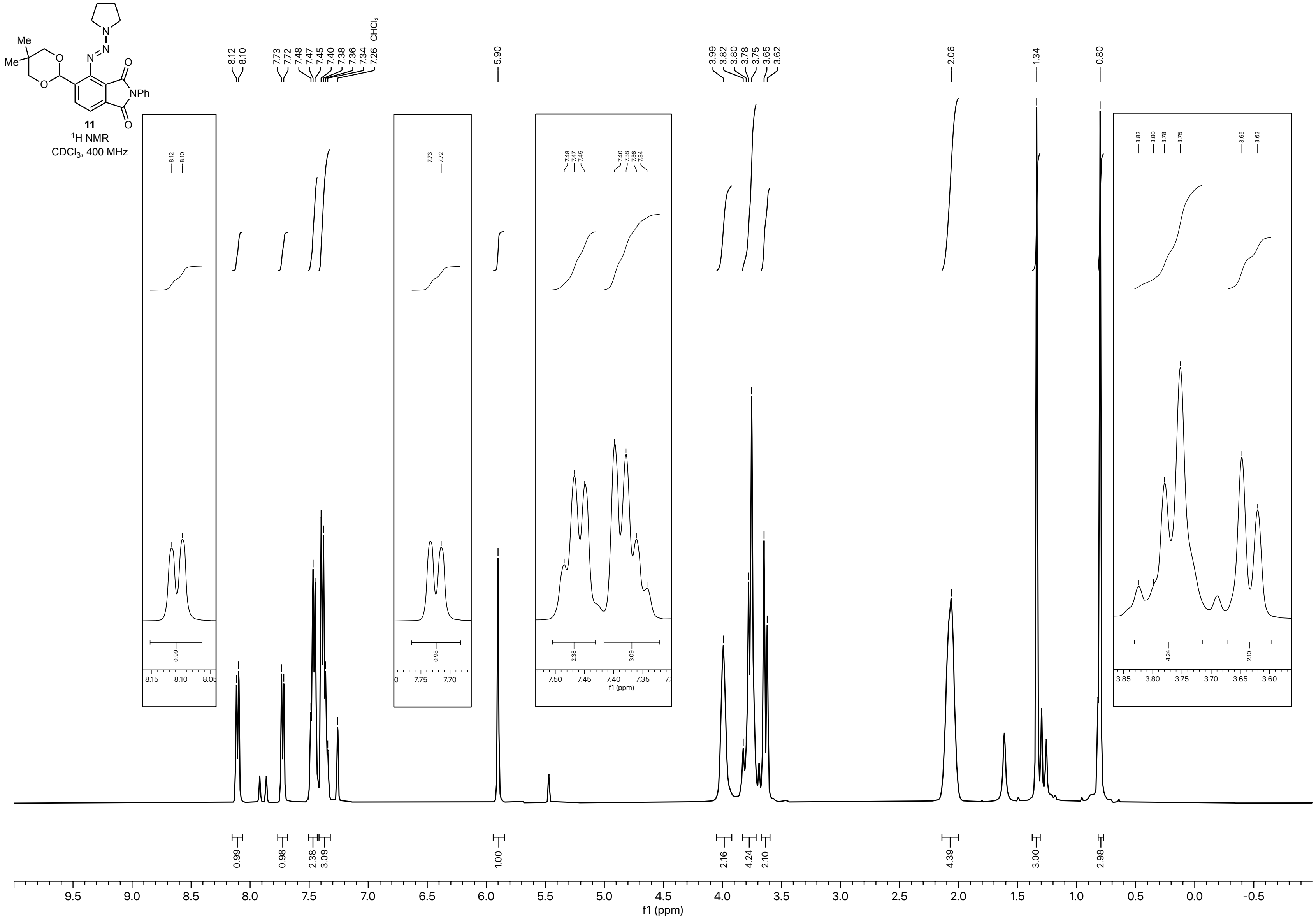

11
^{1}H NMR
$CDCl_3$, 400 MHz

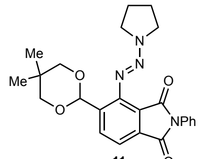

11

^{13}C NMR

$CDCl_3$, 100 MHz

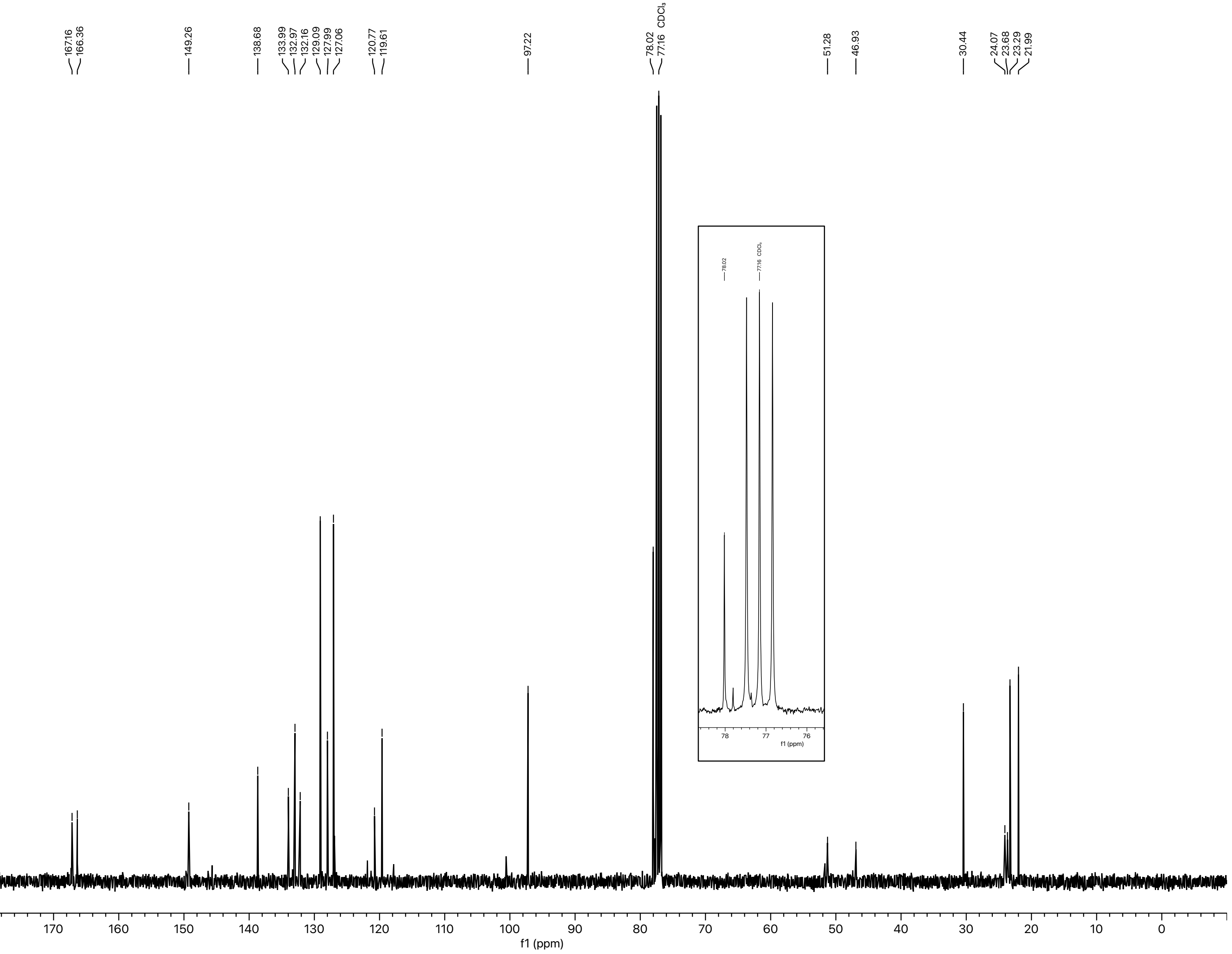

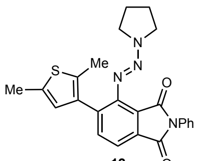

12

^{1}H NMR

$CDCl_3$, 900 MHz

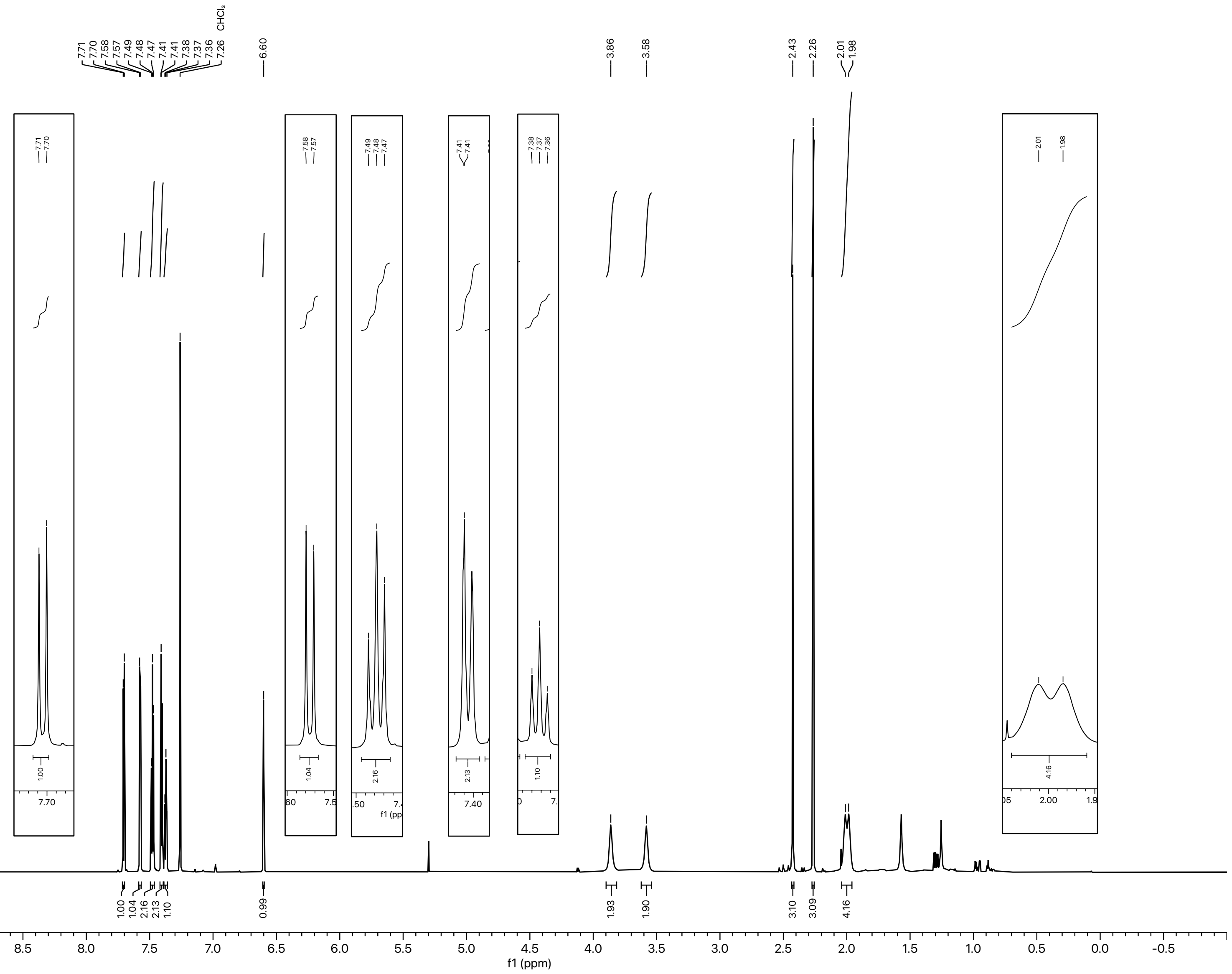

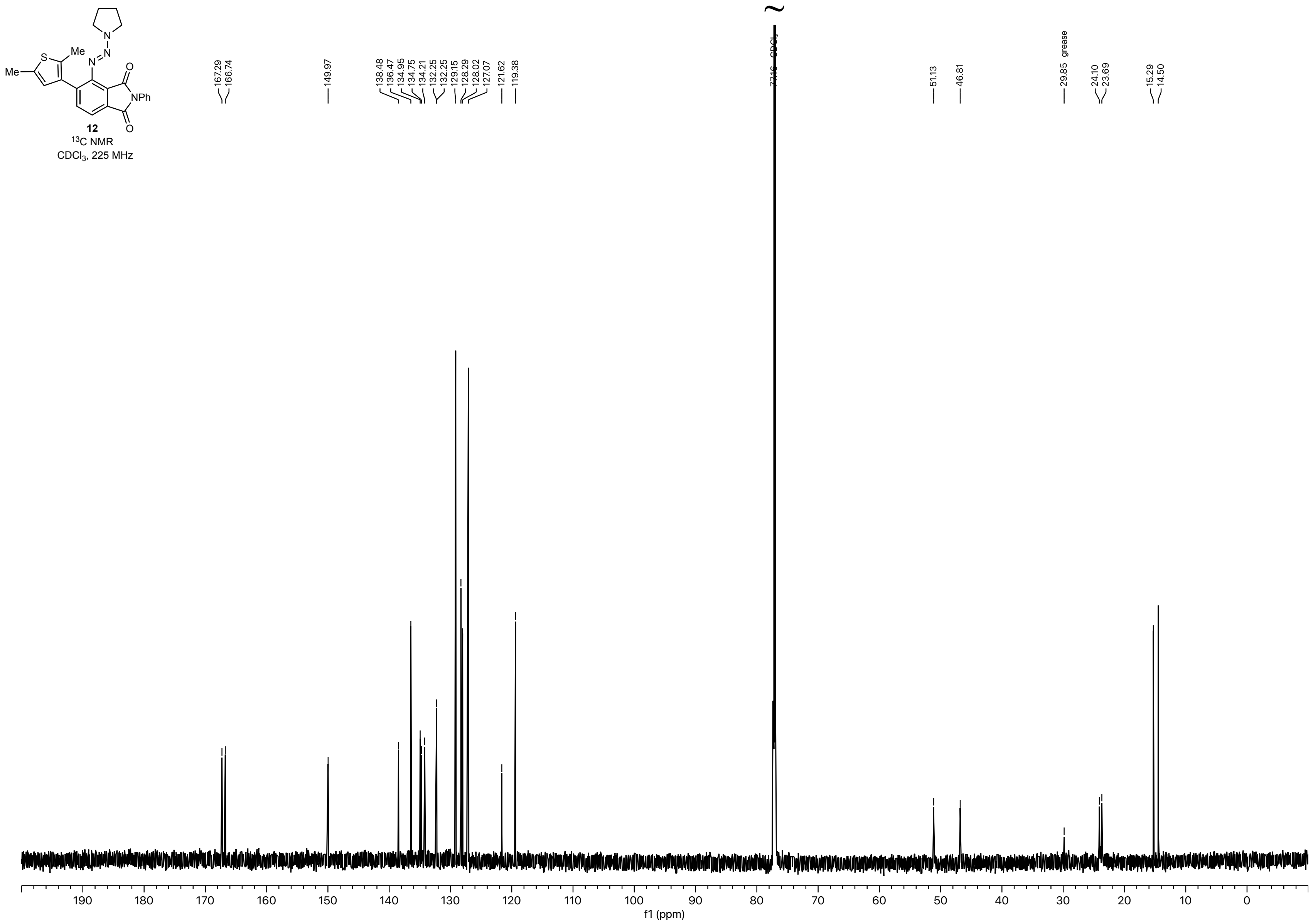
12
^{13}C NMR
$CDCl_3$, 225 MHz
167.29
166.74
149.97
138.48
136.47
134.95
134.75
134.21
132.25
132.25
129.15
128.29
128.02
127.07
121.62
119.38
77.16 $CDCl_3$
51.13
46.81
29.85 grease
24.10
23.69
15.29
14.50
f1 (ppm)

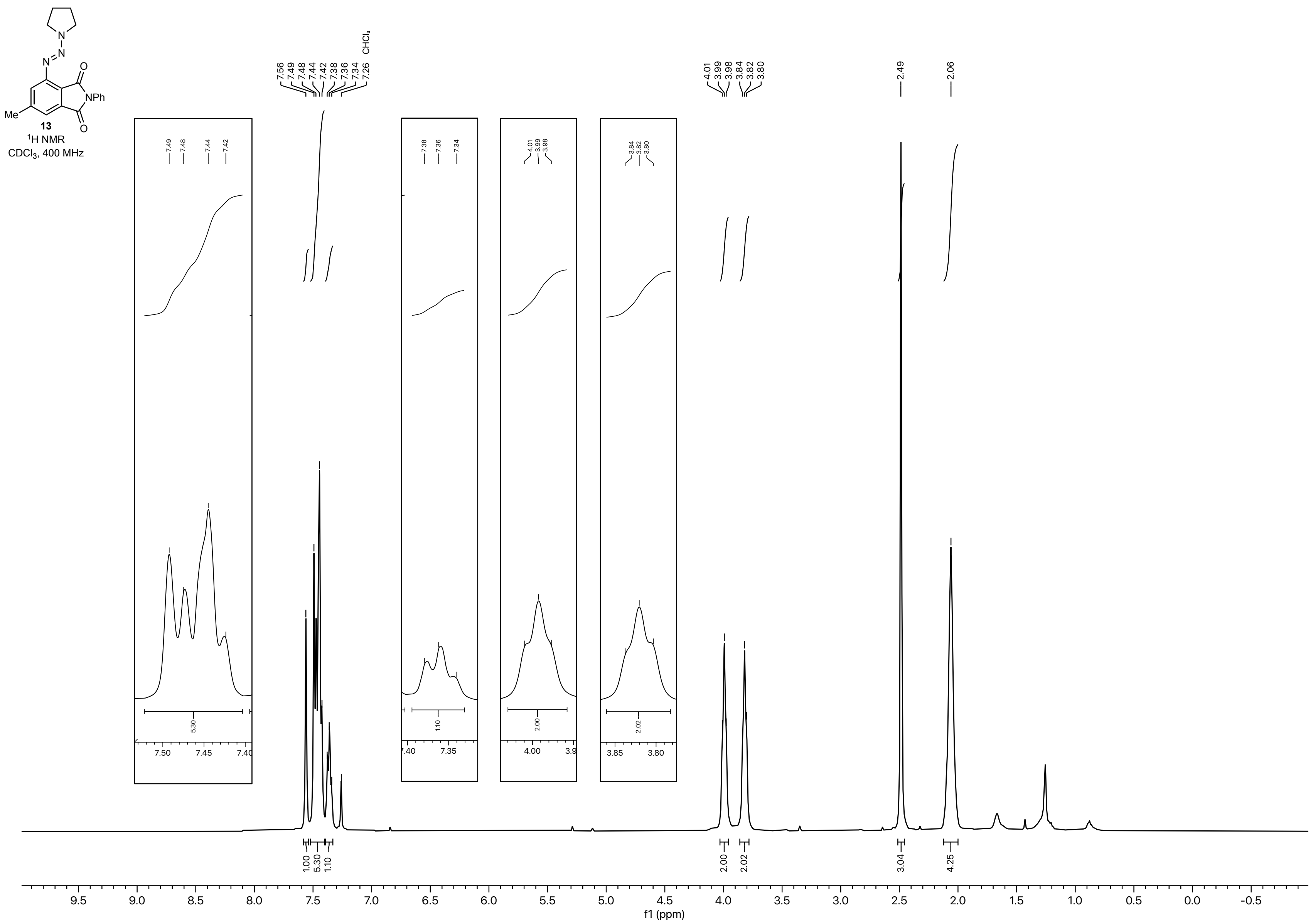

13
^{1}H NMR
$CDCl_3$, 400 MHz
7.56 7.49 7.48 7.44 7.42 7.38 7.36 7.34 7.26
$CHCl_3$
4.01 3.99 3.98 3.84 3.82 3.80
2.49
2.06
1.00 5.30 1.10
2.00 2.02
3.04
4.25
f1 (ppm)

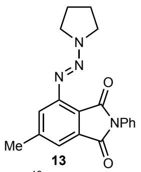

13

^{13}C NMR

$CDCl_3$, 100 MHz

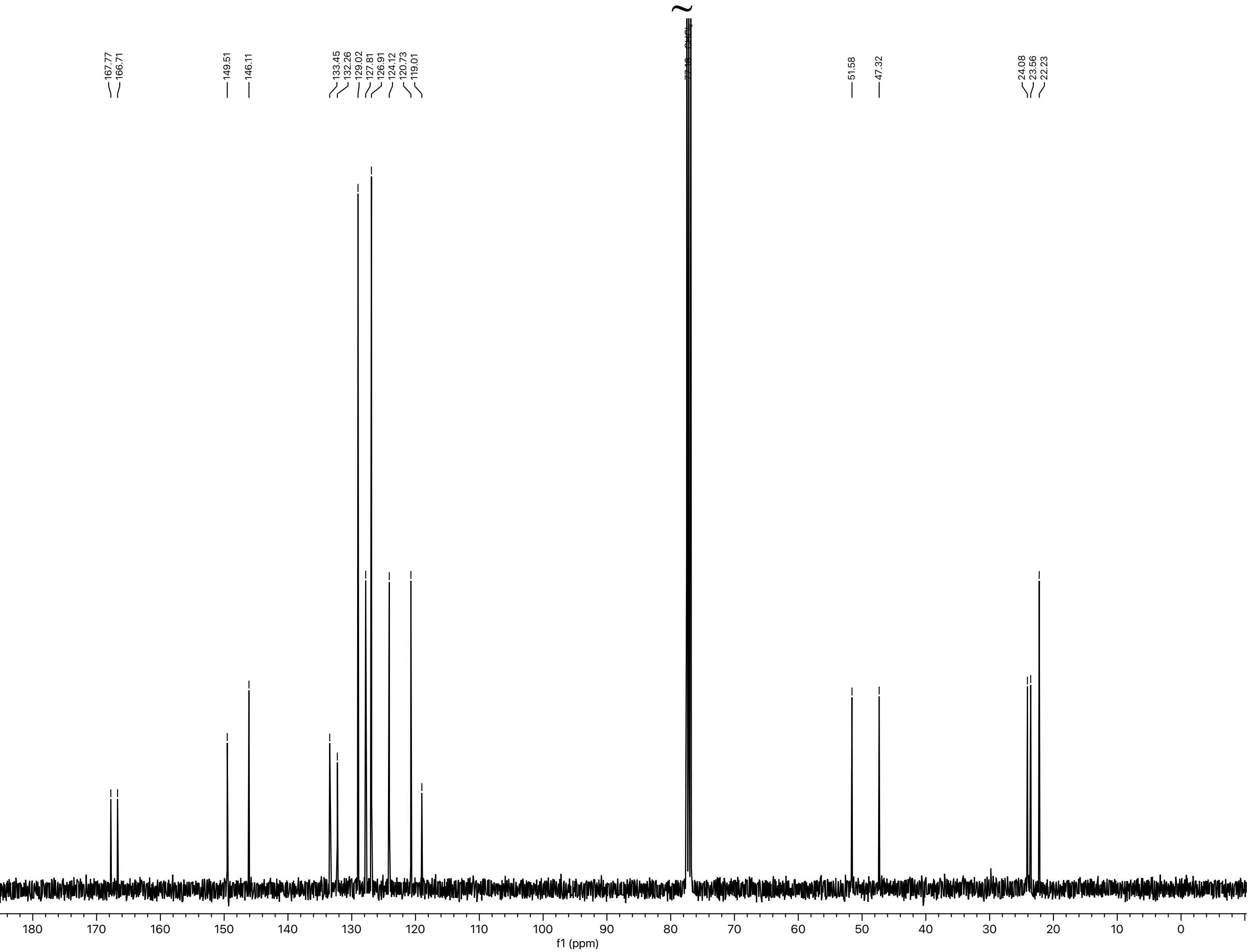

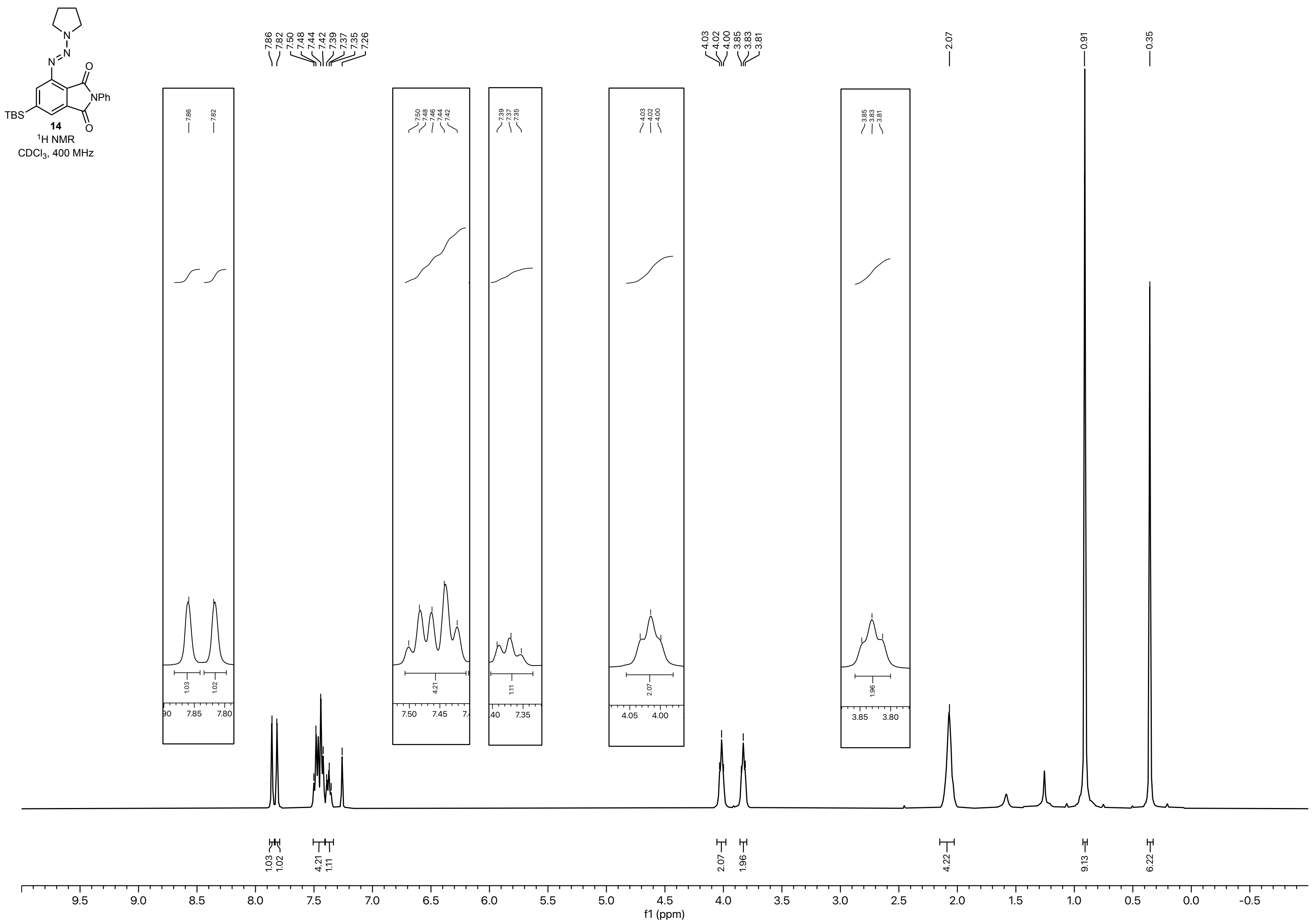
TBS
NPh
14
^{1}H NMR
$CDCl_3$, 400 MHz
7.86 7.82 7.50 7.48 7.44 7.42 7.39 7.37 7.35 7.26
4.03 4.02 4.00 3.85 3.83 3.81
2.07
0.91
0.35
1.03 1.02 4.21 1.11 2.07 1.96 4.22 9.13 6.22
9.5 9.0 8.5 8.0 7.5 7.0 6.5 6.0 5.5 5.0 4.5 4.0 3.5 3.0 2.5 2.0 1.5 1.0 0.5 0.0 -0.5
f1 (ppm)

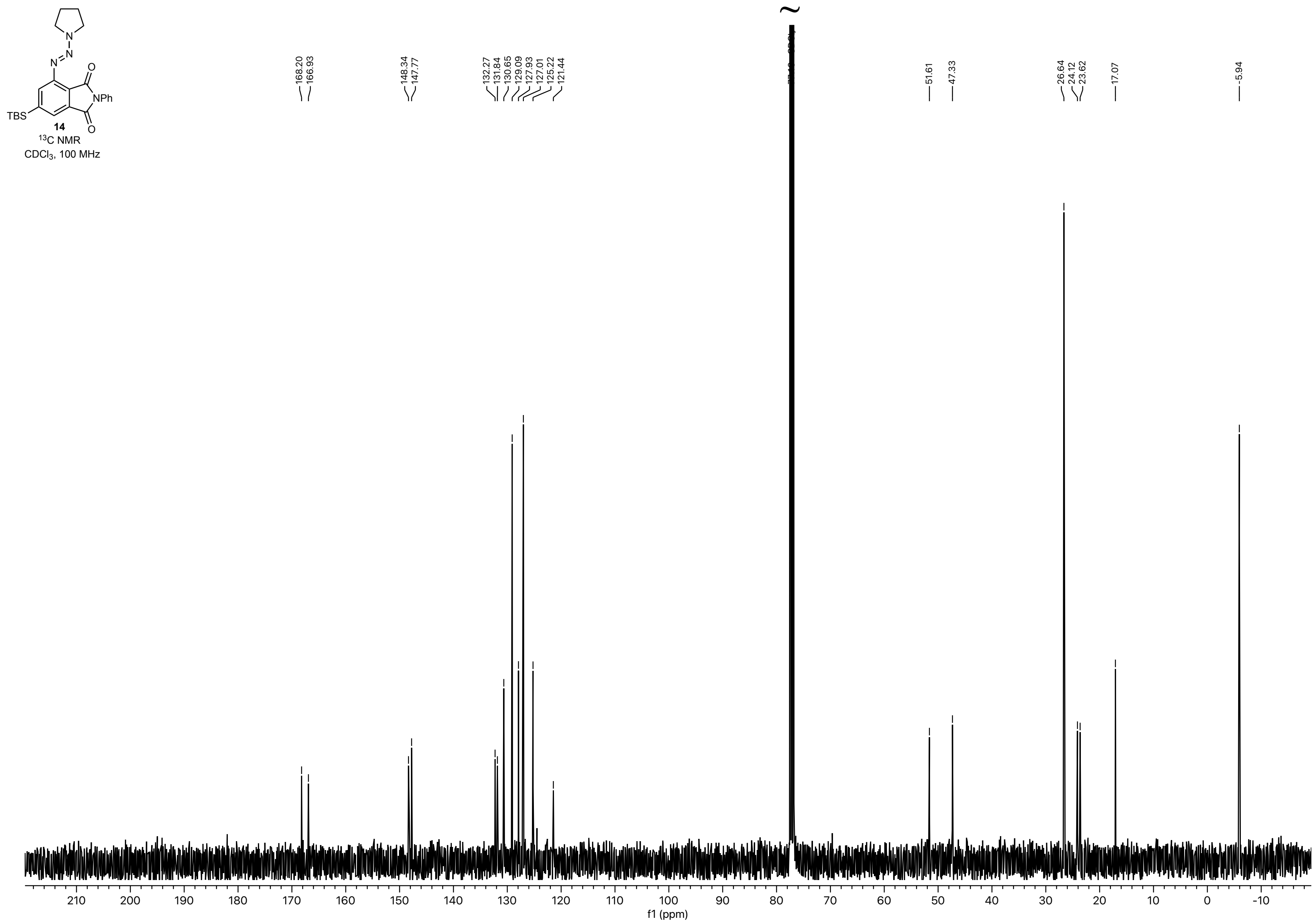

TBS
NPh
14
13C NMR
CDCl3, 100 MHz
168.20
166.93
148.34
147.77
132.27
131.84
130.65
129.09
127.93
127.01
125.22
121.44
51.61
47.33
26.64
24.12
23.62
17.07
-5.94
210 200 190 180 170 160 150 140 130 120 110 100 90 80 70 60 50 40 30 20 10 0 -10
f1 (ppm)

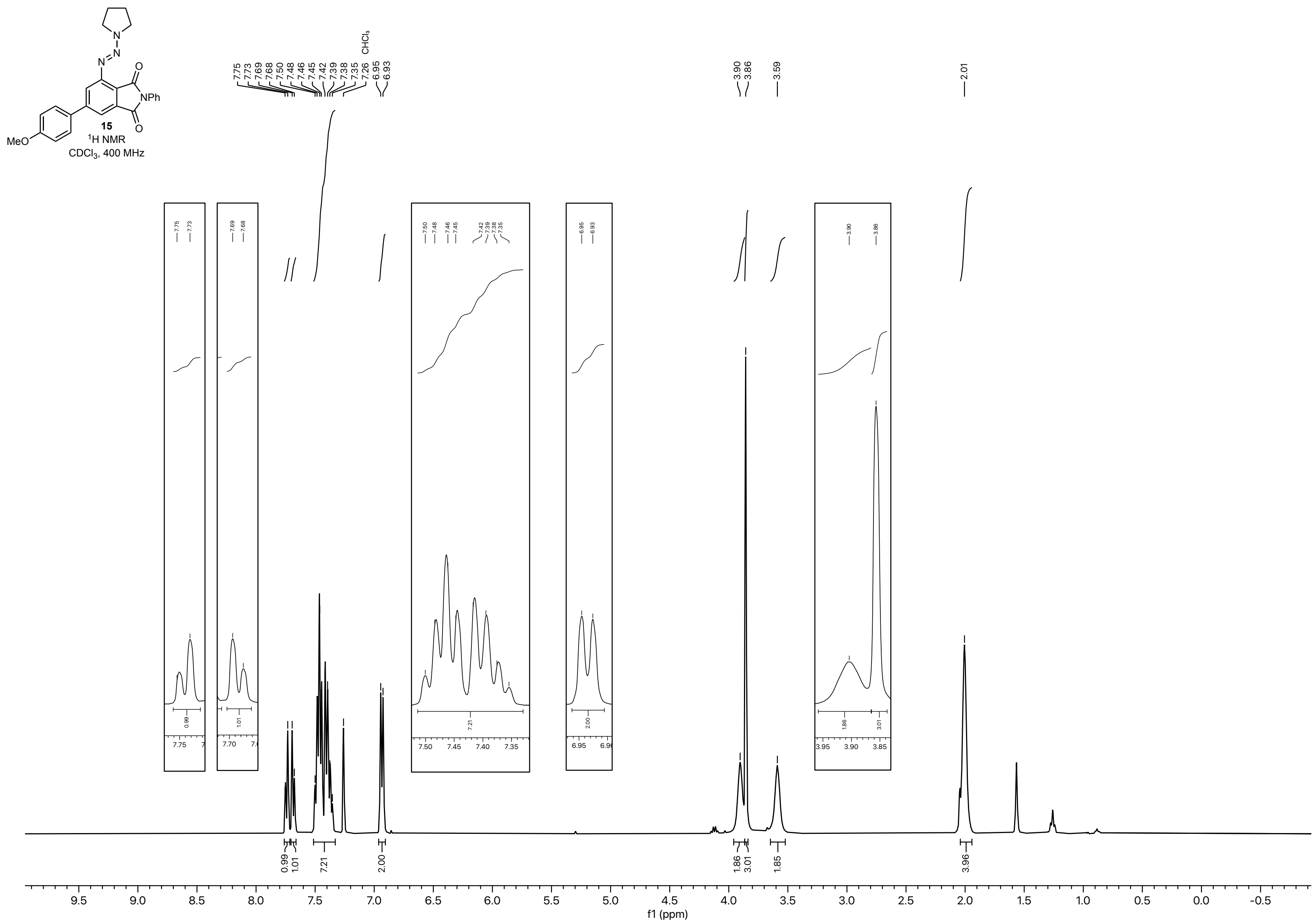

15
^{1}H NMR
CDCl$_3$, 400 MHz
MeO
NPh
CHCl$_3$
7.75 7.73 7.69 7.68 7.50 7.48 7.46 7.45 7.42 7.39 7.38 7.35 7.26 6.95 6.93
3.90 3.86 3.59 2.01
0.99 1.01 7.21 2.00 1.86 3.01 1.85 3.96
f1 (ppm)

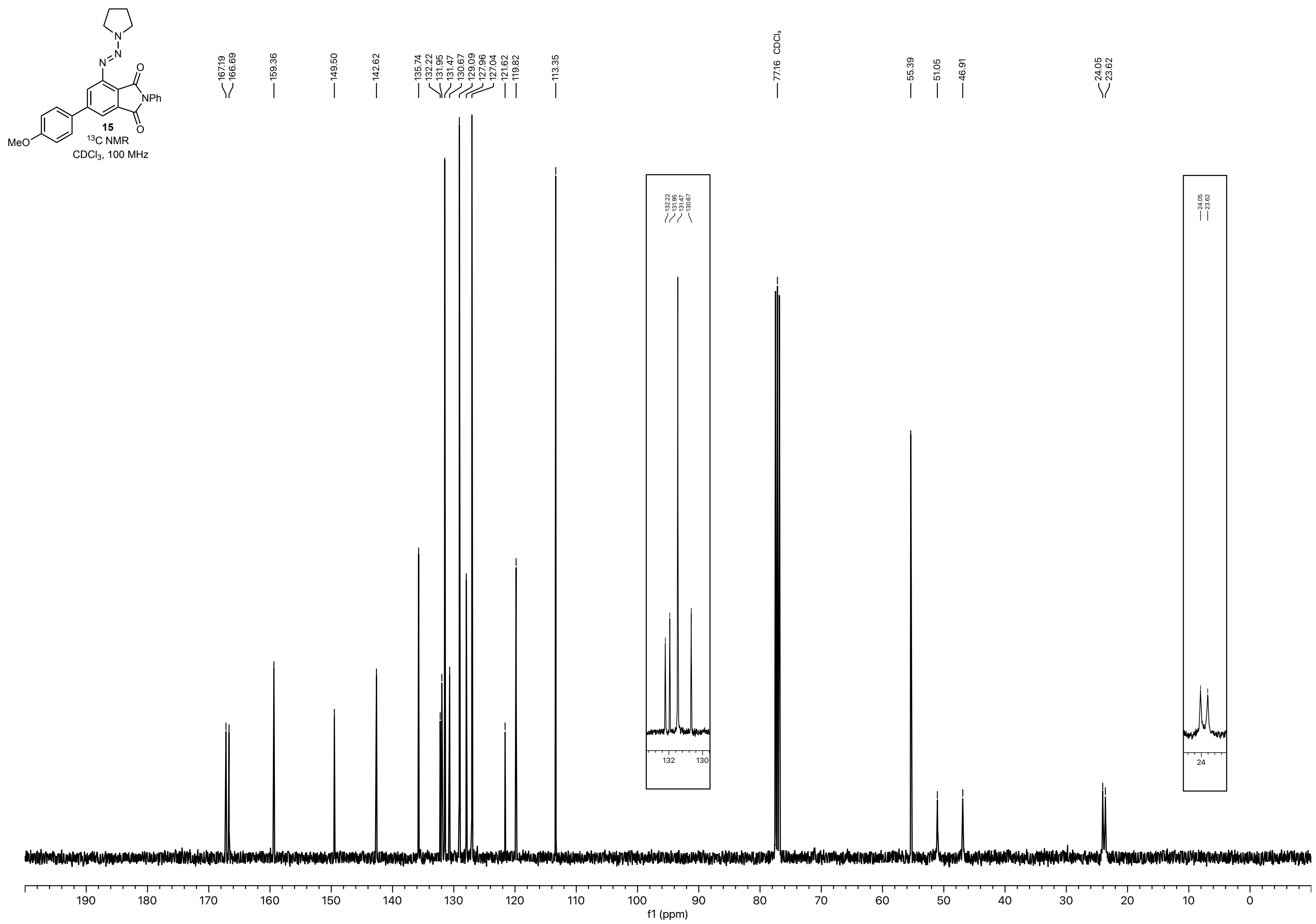
N
N
N
O
NPh
O
MeO
15
^{13}C NMR
CDCl$_3$, 100 MHz
167.19
166.69
159.36
149.50
142.62
135.74
132.22
131.95
131.47
130.67
129.09
127.96
127.04
121.62
119.82
113.35
77.16 CDCl$_3$
55.39
51.05
46.91
24.05
23.62
190
180
170
160
150
140
130
120
110
100
90
80
70
60
50
40
30
20
10
0
f1 (ppm)

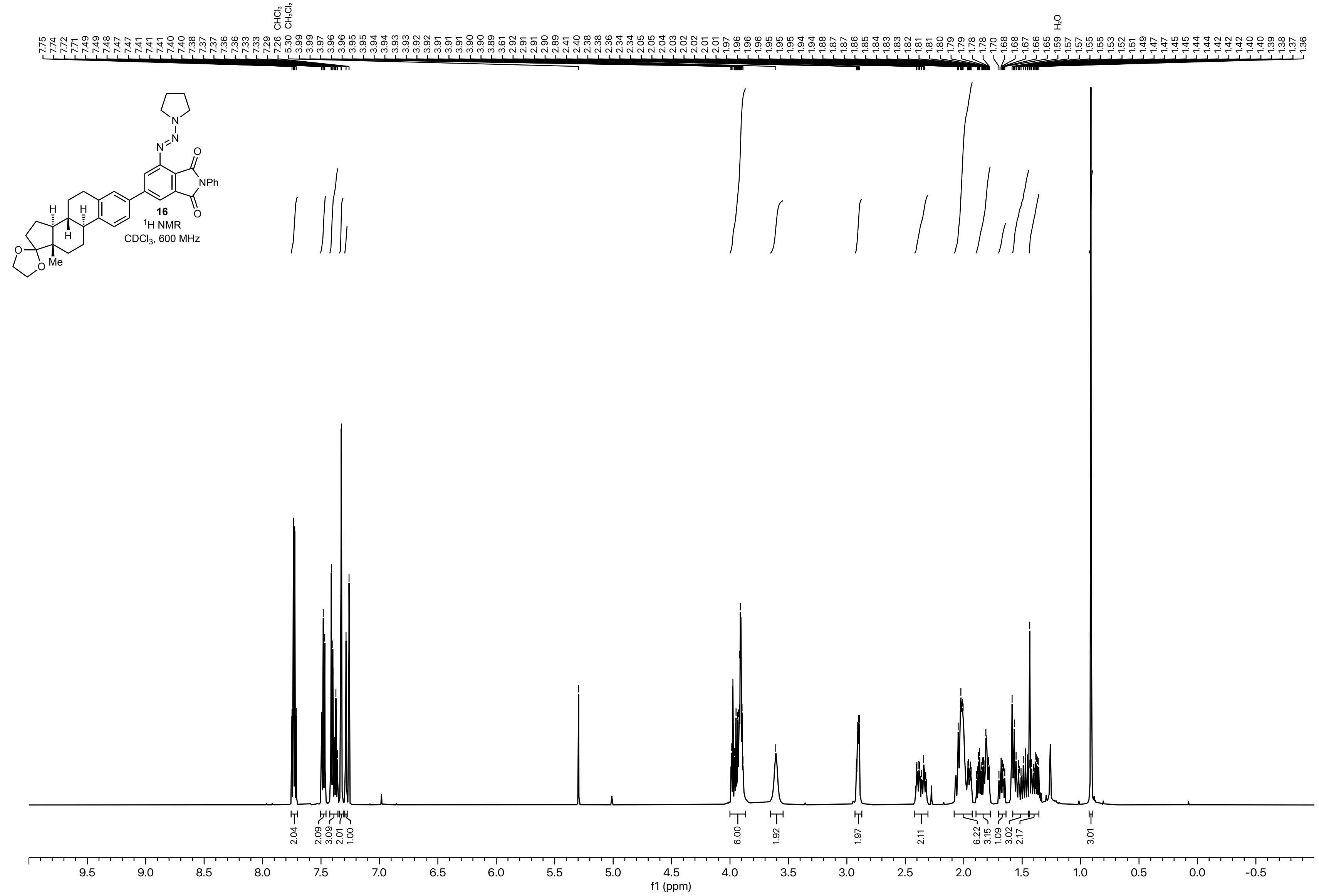
16
1H NMR
CDCl3, 600 MHz
NPh
Me
CHCl3
CH2Cl2
H2O
f1 (ppm)

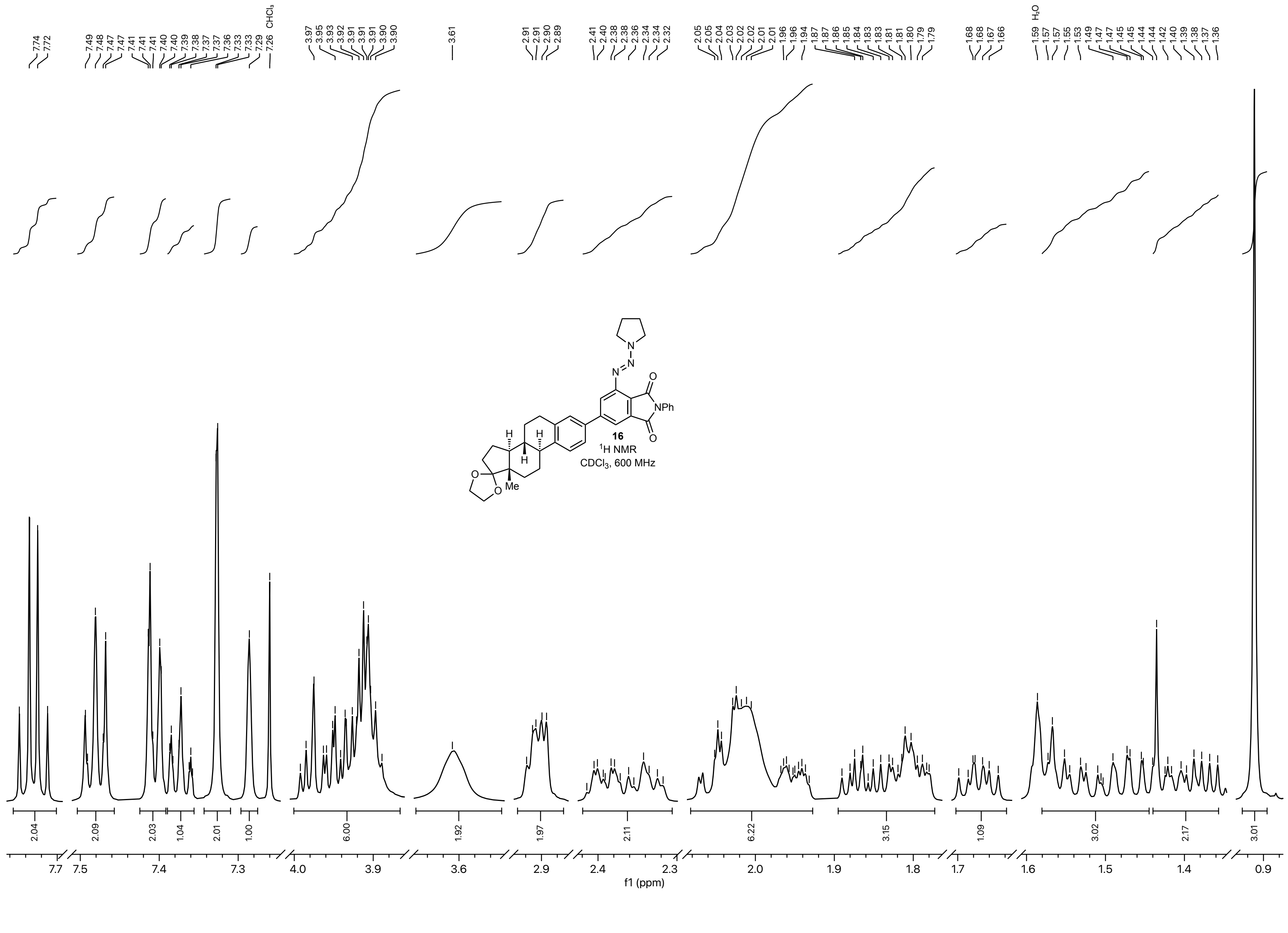
16
¹H NMR
CDCl₃, 600 MHz
f1 (ppm)

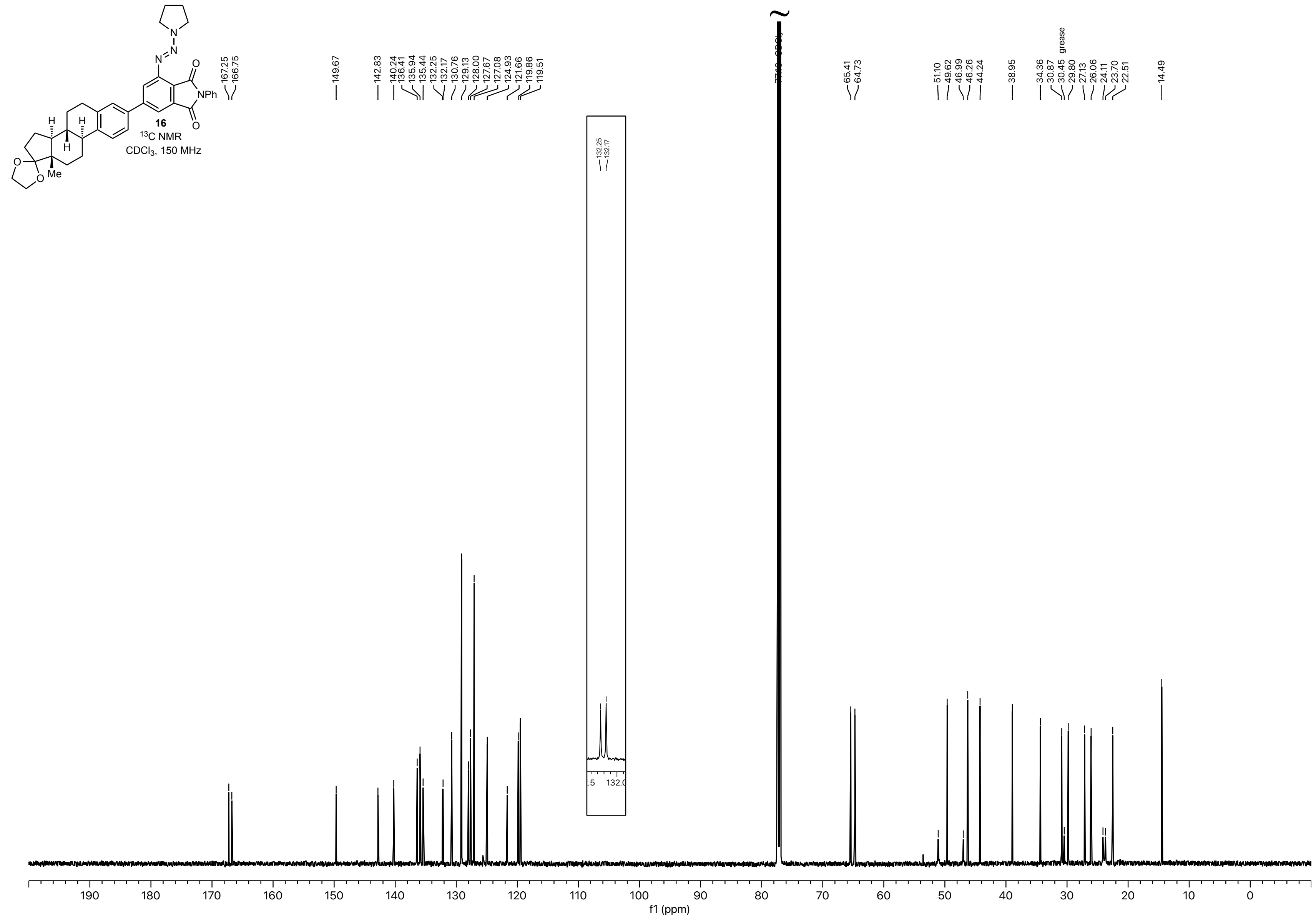
16
^{13}C NMR
$CDCl_3$, 150 MHz
NPh
Me
167.25
166.75
149.67
142.83
140.24
136.41
135.94
135.44
132.25
132.17
130.76
129.13
128.00
127.67
127.08
124.93
121.66
119.86
119.51
65.41
64.73
51.10
49.62
46.99
46.26
44.24
38.95
34.36
30.87
30.45
grease
29.80
27.13
26.06
24.11
23.70
22.51
14.49
132.25
132.17
190 180 170 160 150 140 130 120 110 100 90 80 70 60 50 40 30 20 10 0
f1 (ppm)

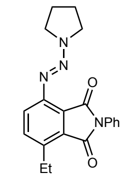

17
^1H NMR
$CDCl_3$, 400 MHz

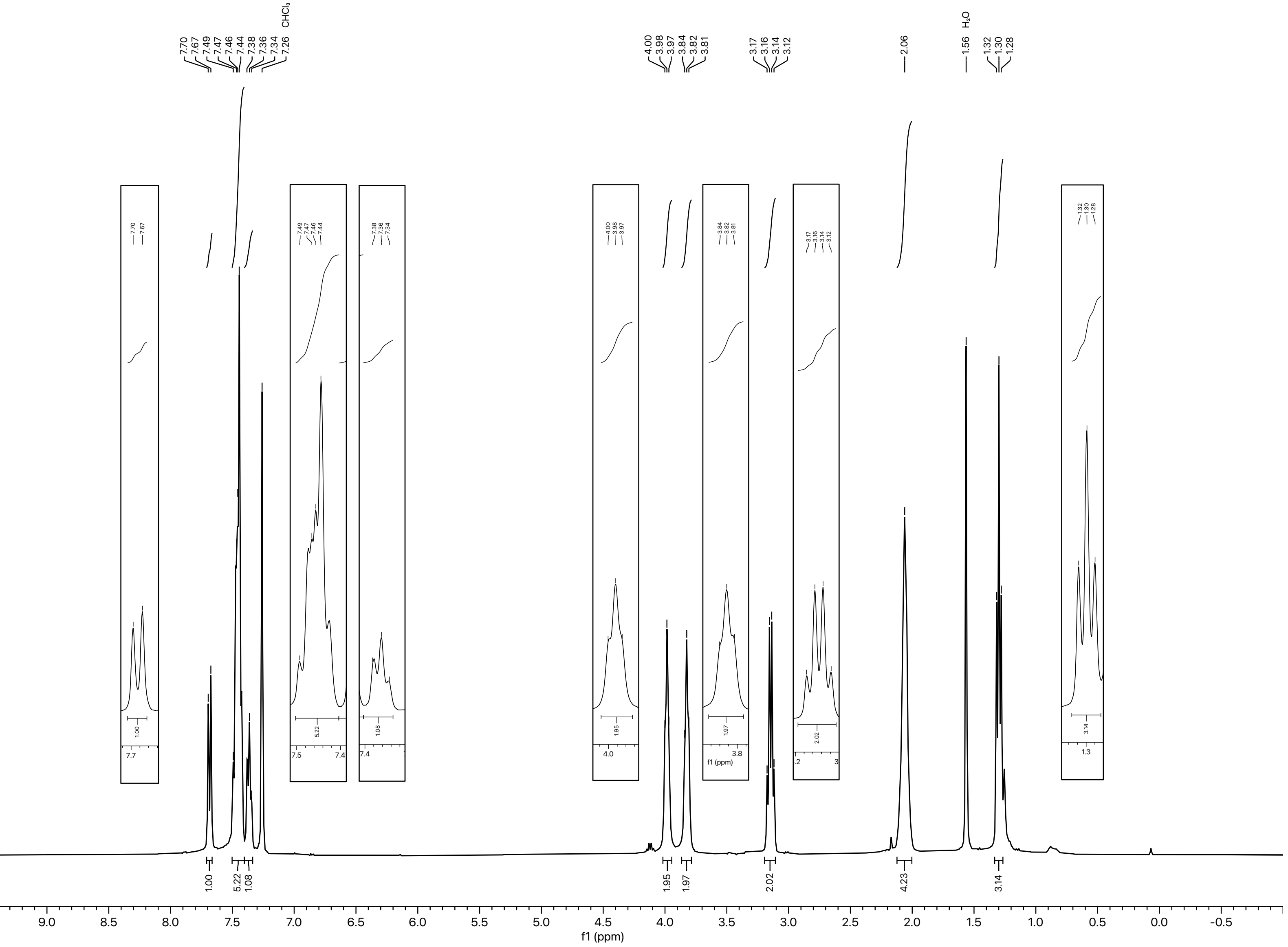

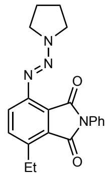

17
^{13}C NMR
$CDCl_3$, 100 MHz

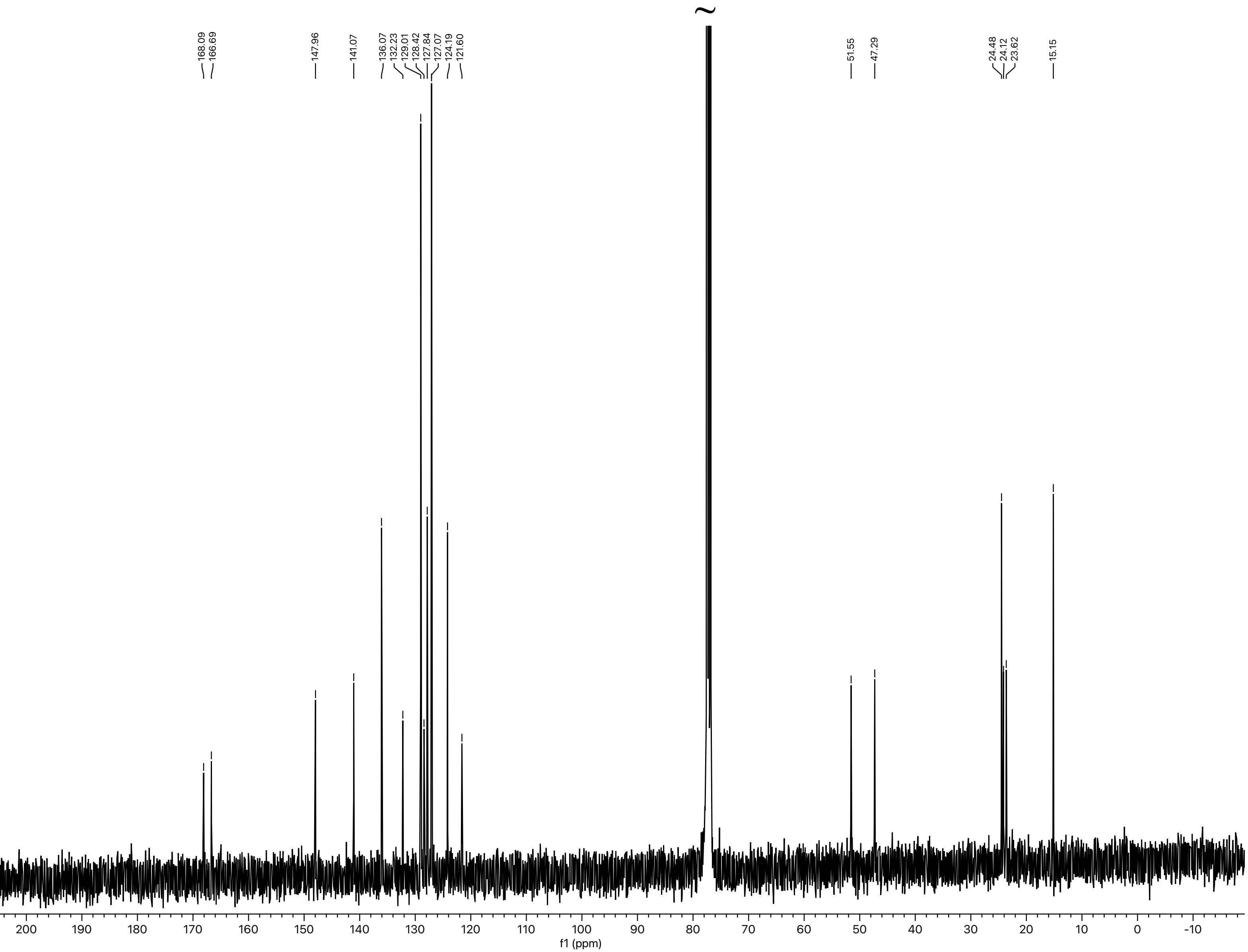

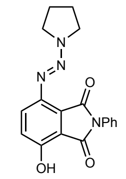

18
^1H NMR
$CDCl_3$, 900 MHz

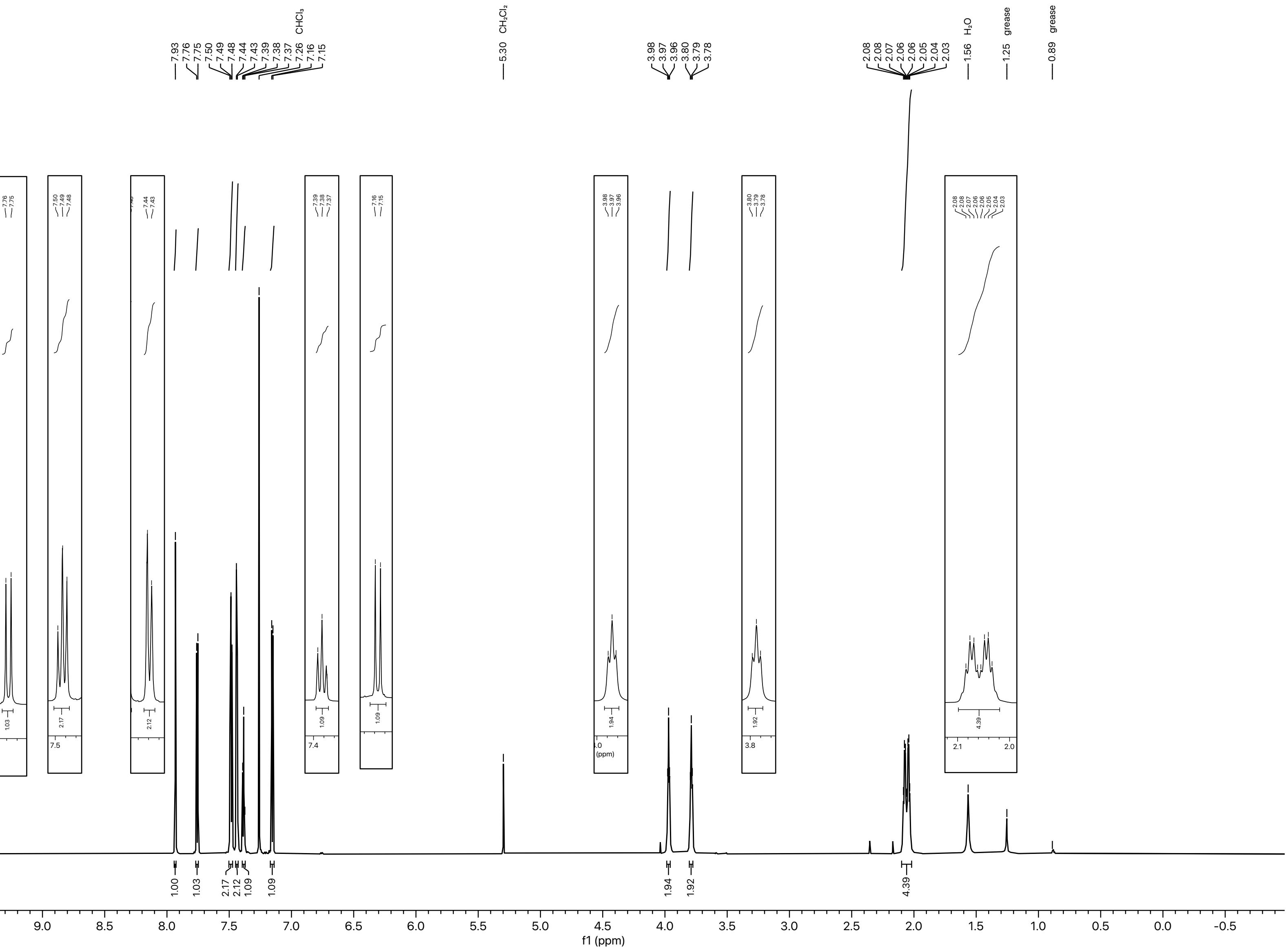

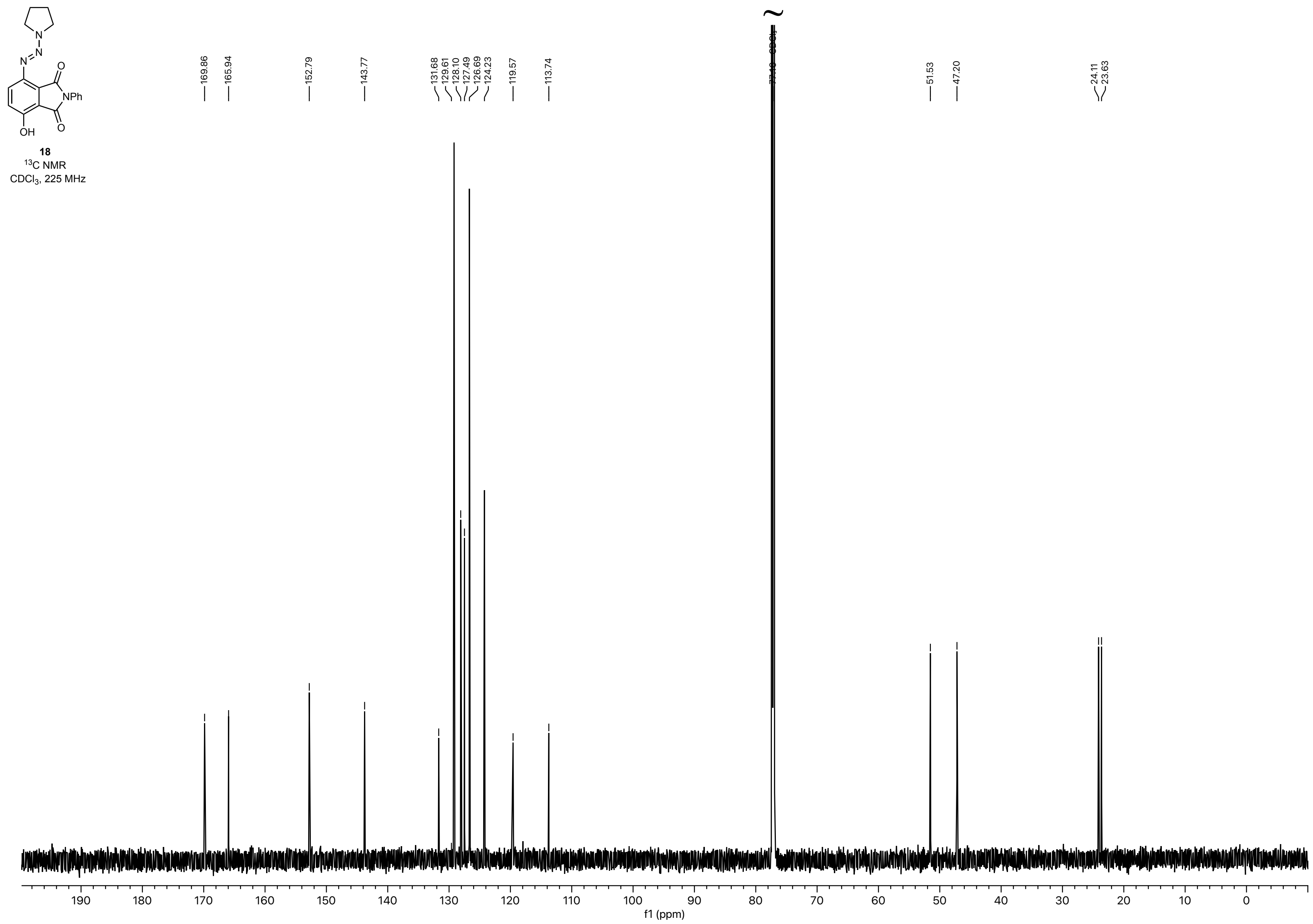

N
N
N
O
NPh
O
OH
18
^{13}C NMR
CDCl$_3$, 225 MHz
169.86
165.94
152.79
143.77
131.68
129.61
128.10
127.49
126.69
124.23
119.57
113.74
CDCl$_3$
51.53
47.20
24.11
23.63
190
180
170
160
150
140
130
120
110
100
90
80
70
60
50
40
30
20
10
0
f1 (ppm)

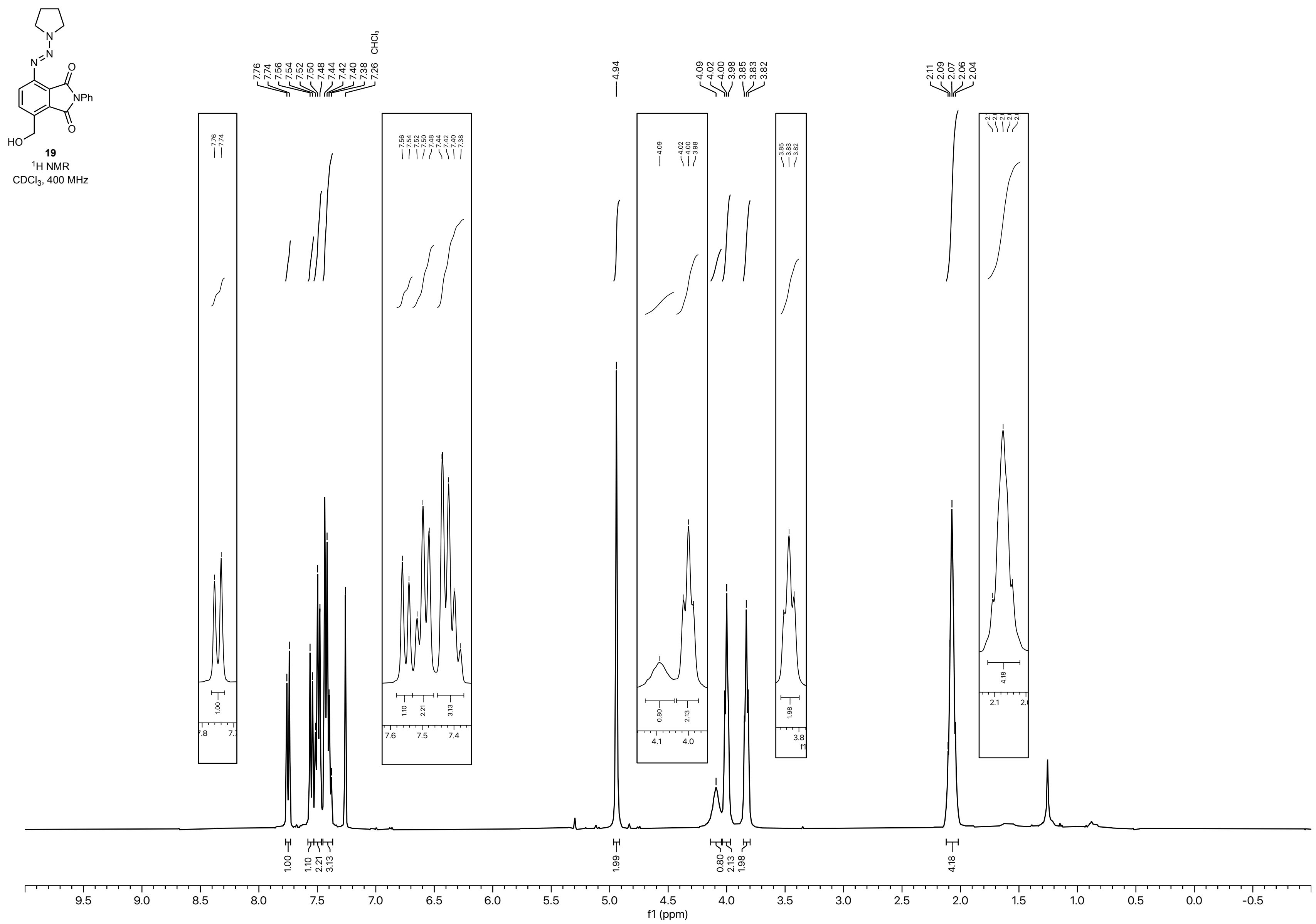

NPh
HO
19
¹H NMR
CDCl₃, 400 MHz
7.76 7.74 7.56 7.54 7.52 7.50 7.48 7.44 7.42 7.40 7.38 7.26 CHCl₃
4.94
4.09 4.02 4.00 3.98 3.85 3.83 3.82
2.11 2.09 2.07 2.06 2.04
1.00 1.10 2.21 3.13 1.99 0.80 2.13 1.98 4.18
f1 (ppm)

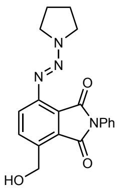

19

^{13}C NMR

$CDCl_3$, 100 MHz

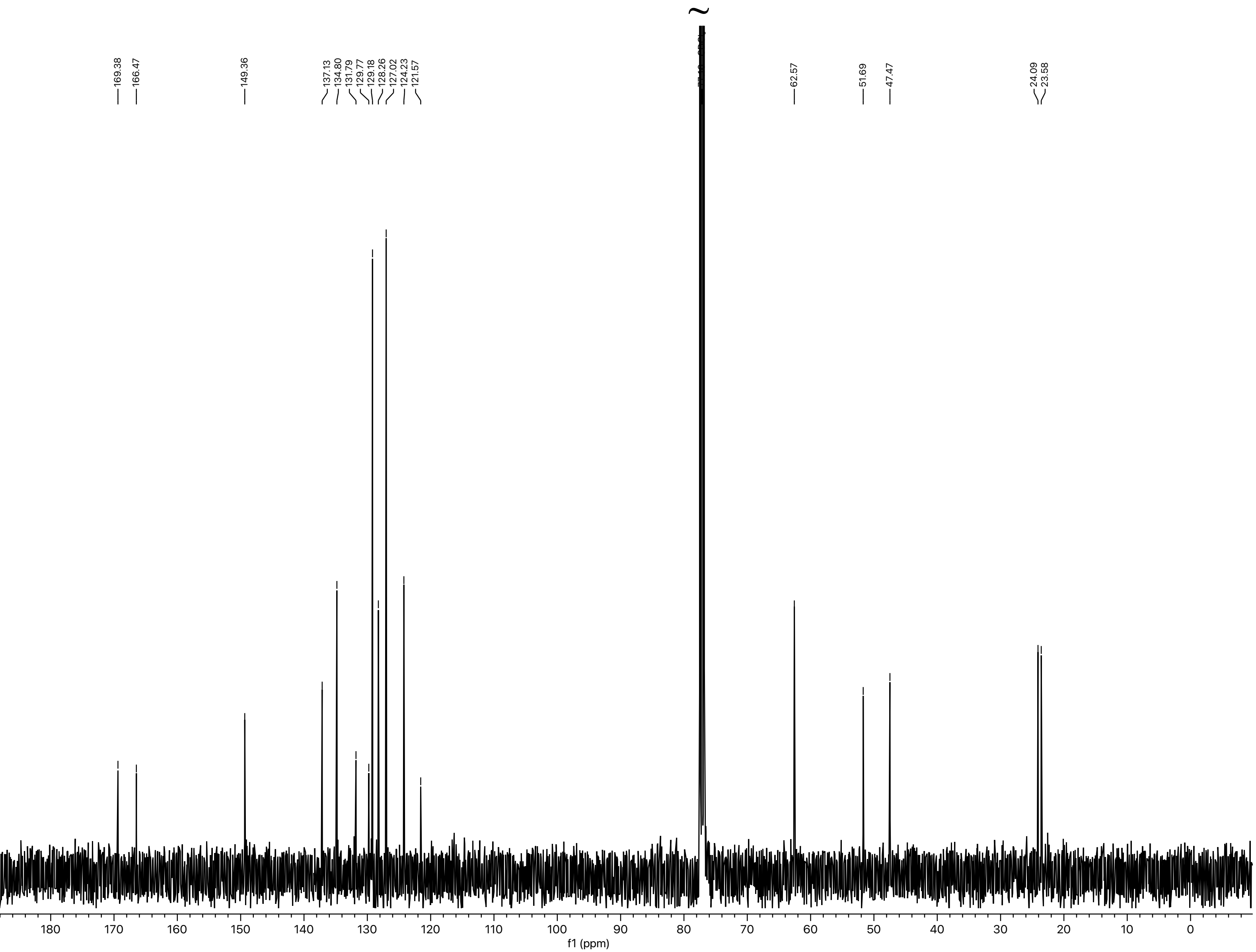

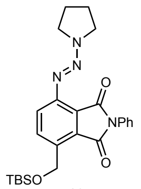

20
^{1}H NMR
$CDCl_3$, 900 MHz

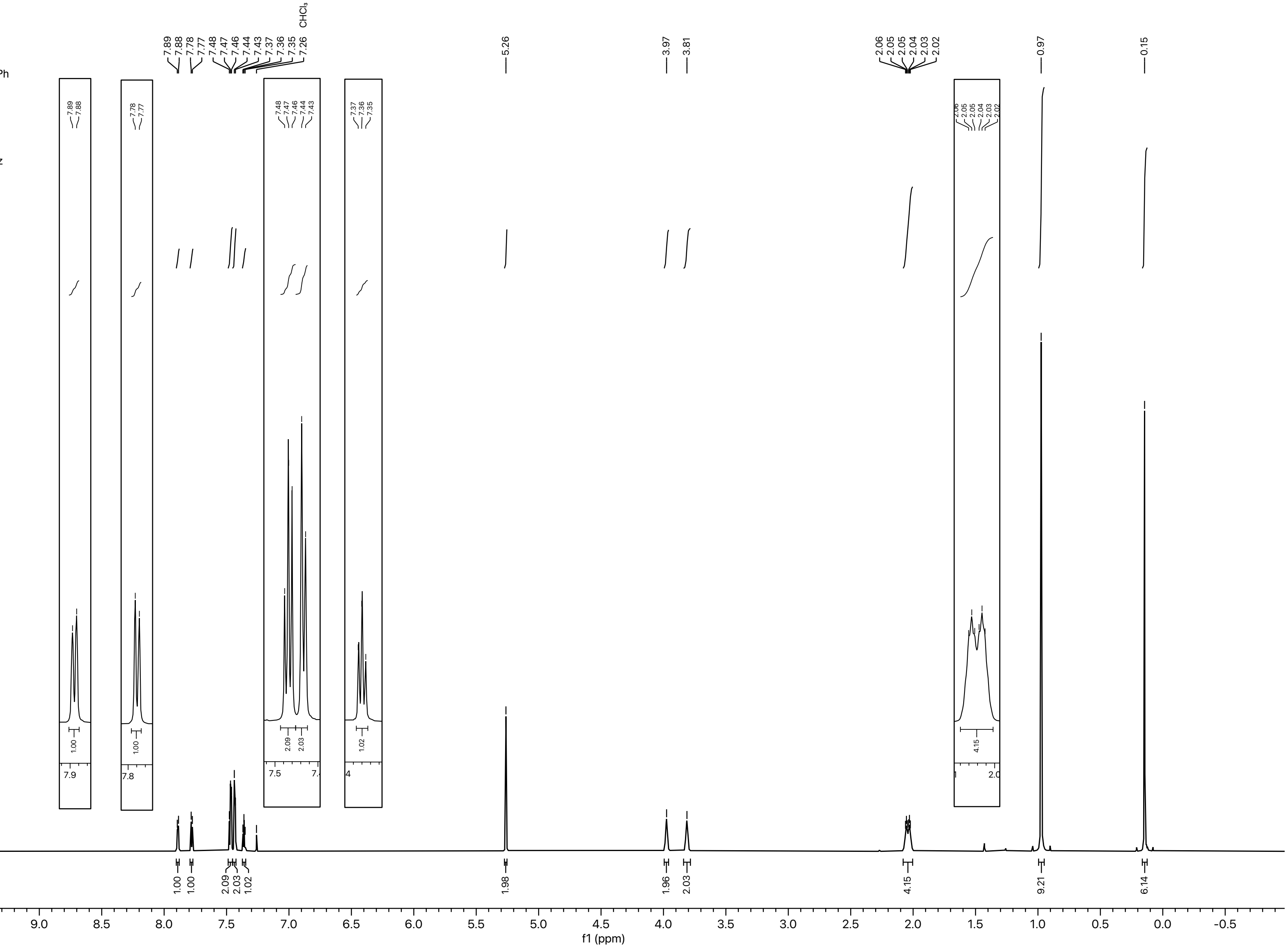

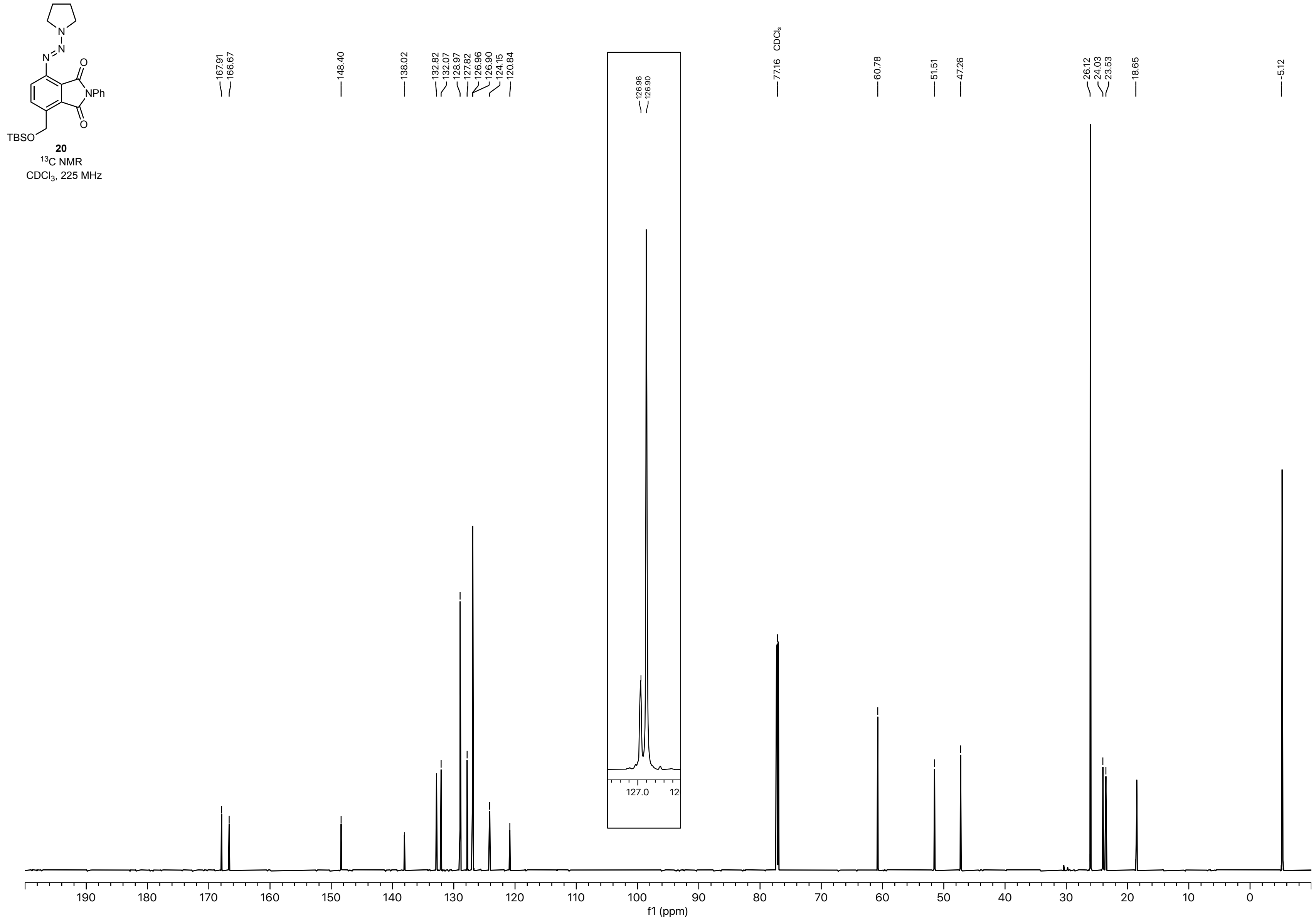
N
N
N
O
NPh
O
TBSO
20
13C NMR
CDCl3, 225 MHz
167.91
166.67
148.40
138.02
132.82
132.07
128.97
127.82
126.96
126.90
124.15
120.84
126.96
126.90
127.0
77.16 CDCl3
60.78
51.51
47.26
26.12
24.03
23.53
18.65
-5.12
190
180
170
160
150
140
130
120
110
100
90
80
70
60
50
40
30
20
10
0
f1 (ppm)

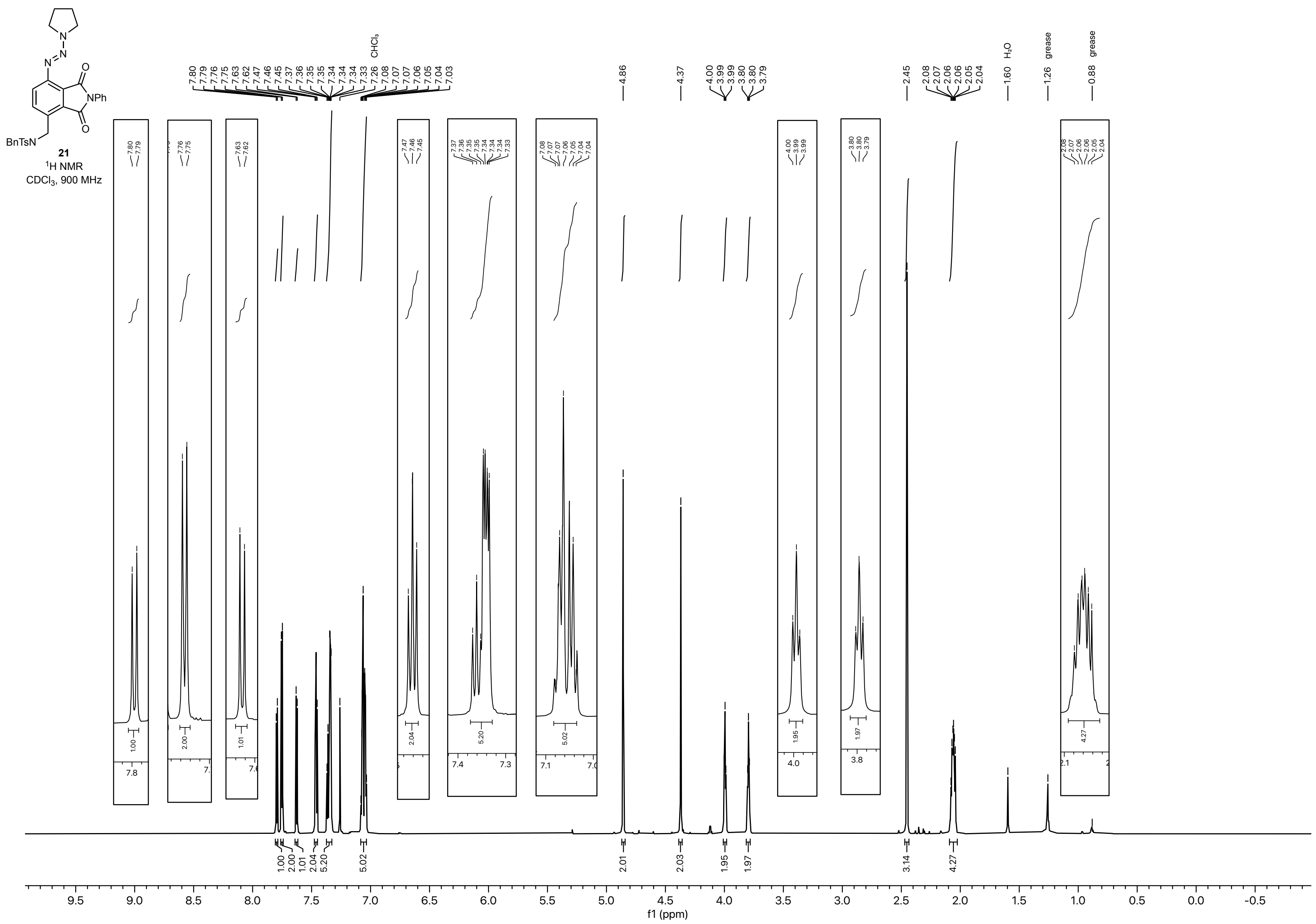

21
^{1}H NMR
$CDCl_3$, 900 MHz
BnTsN
NPh
CHCl3
H2O
grease
grease
f1 (ppm)

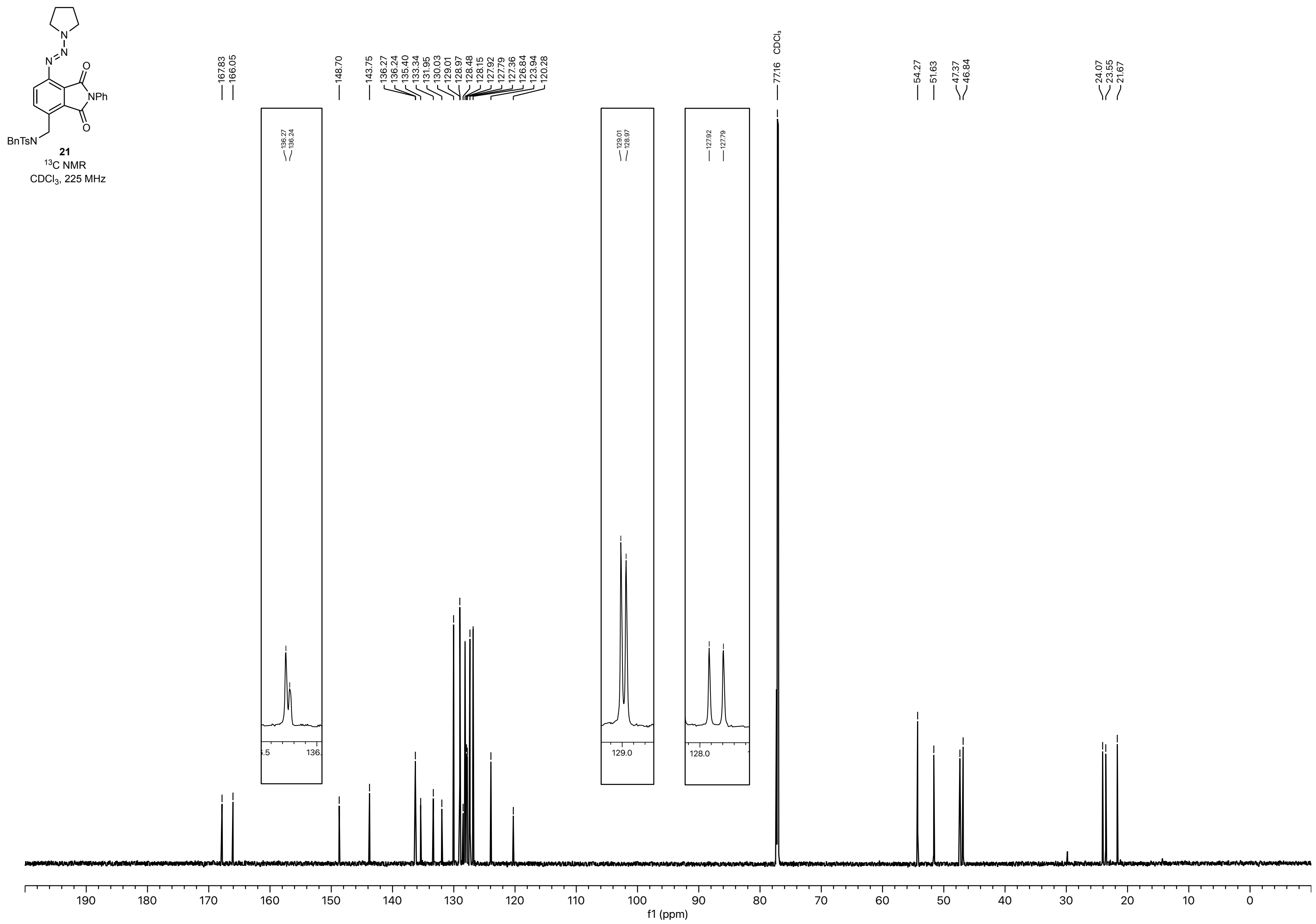

NPh
BnTsN
21
^{13}C NMR
$CDCl_3$, 225 MHz
167.83
166.05
148.70
143.75
136.27
136.24
135.40
133.34
131.95
130.03
129.01
128.97
128.48
128.15
127.92
127.79
127.36
126.84
123.94
120.28
$CDCl_3$
77.16
54.27
51.63
47.37
46.84
24.07
23.55
21.67
f1 (ppm)

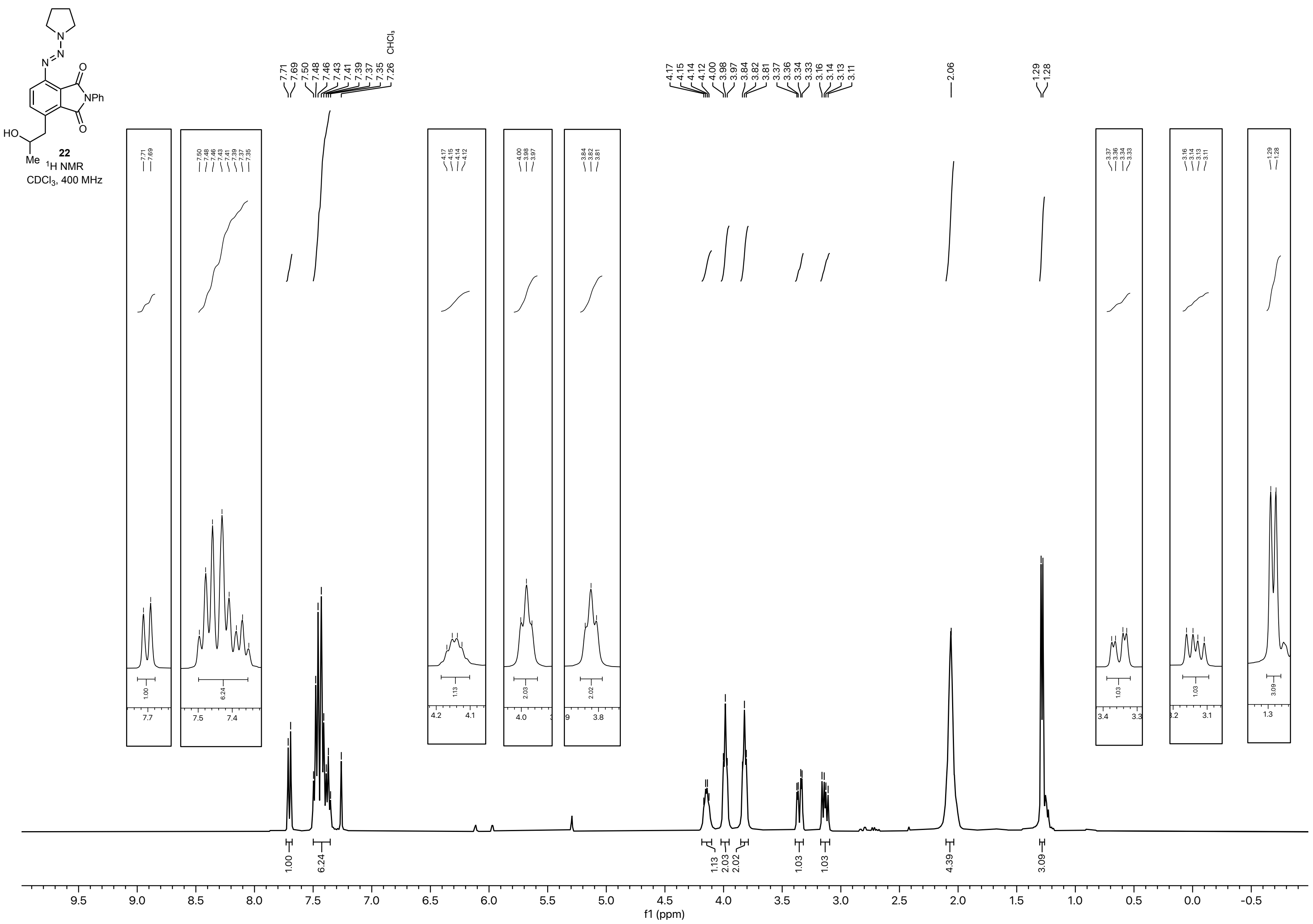

22
1H NMR
CDCl3, 400 MHz
CHCl3
f1 (ppm)

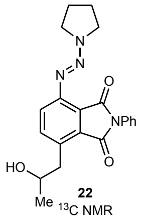

22

^{13}C NMR

$CDCl_3$, 100 MHz

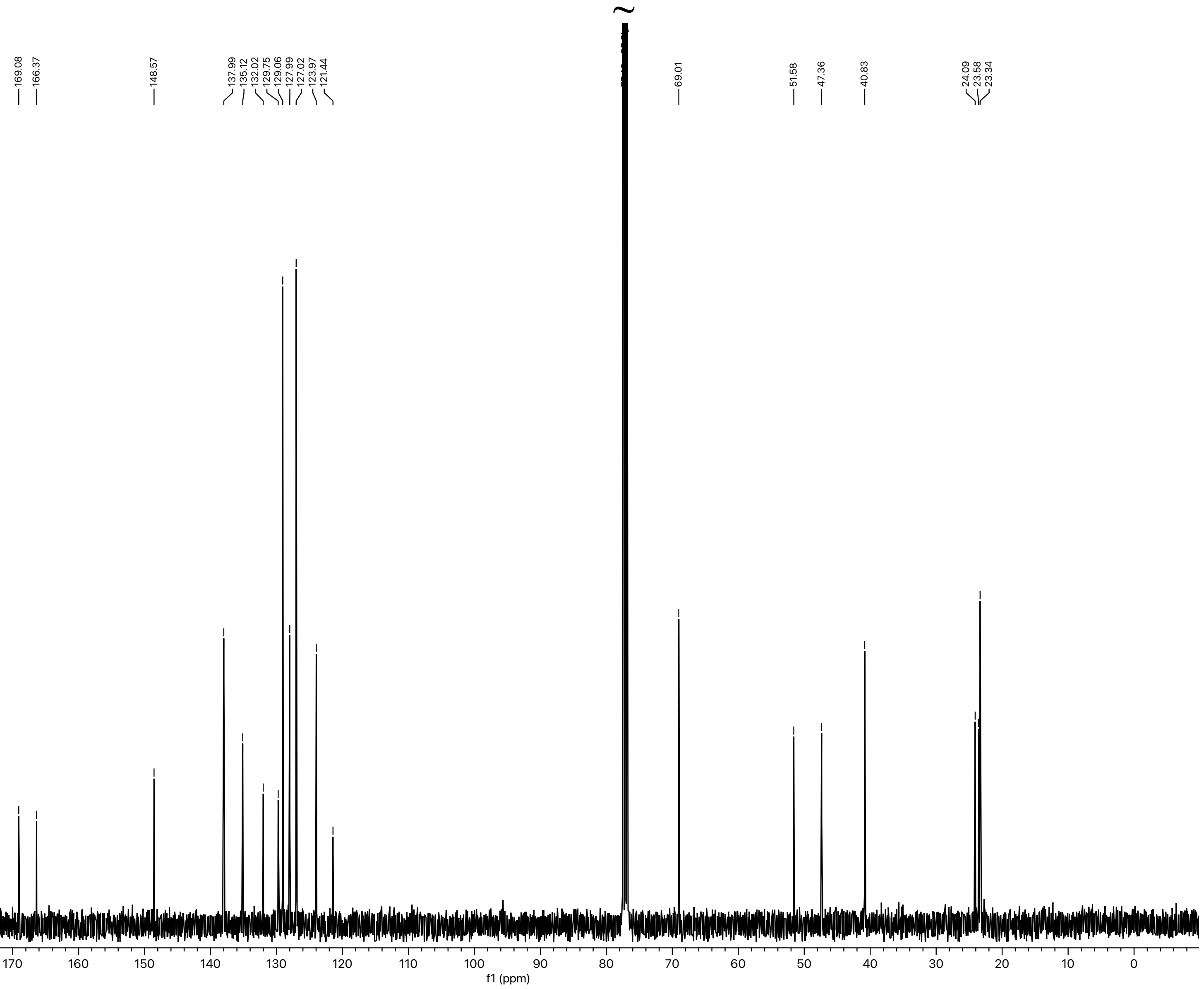

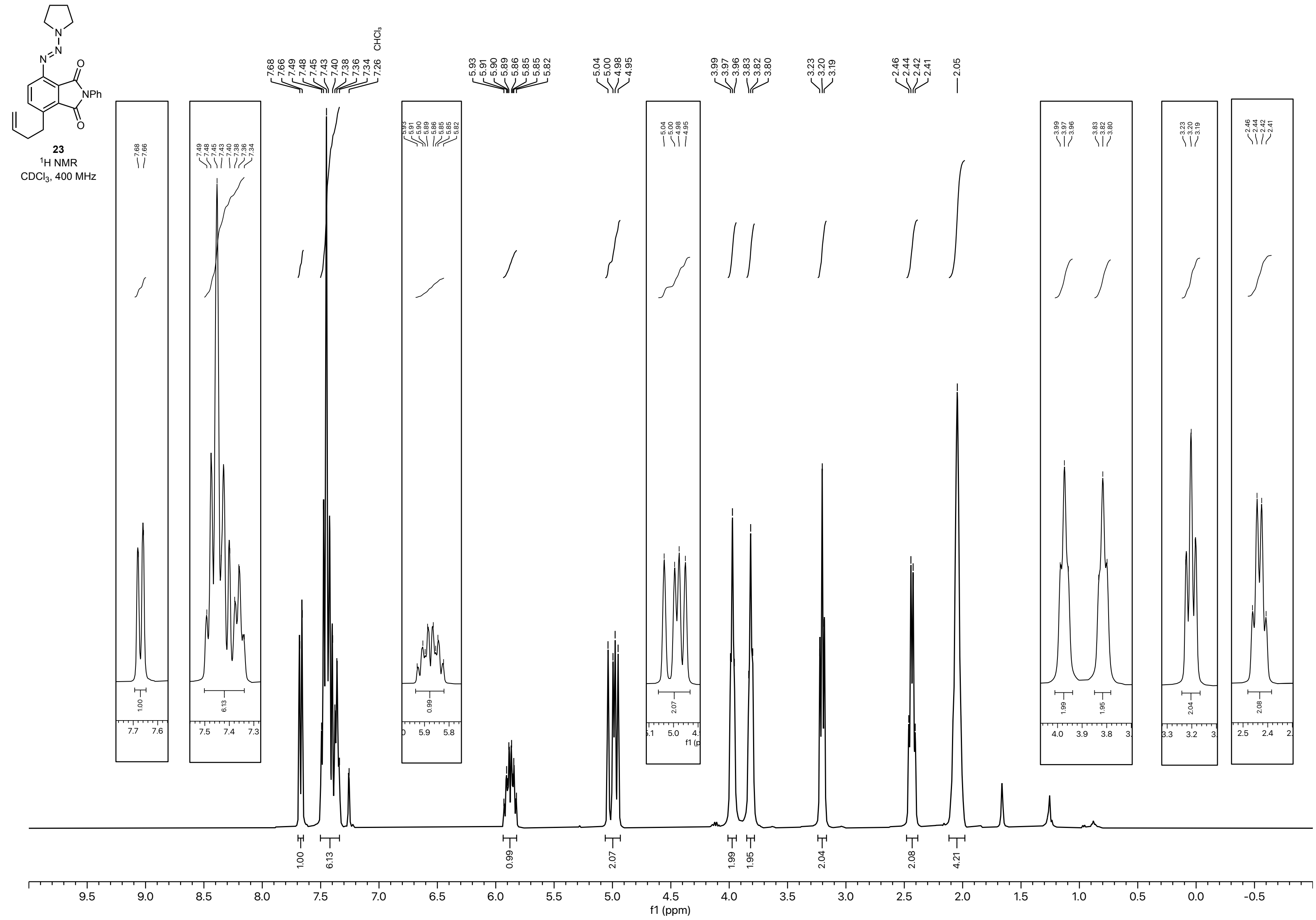

23

^{1}H NMR

$CDCl_3$, 400 MHz

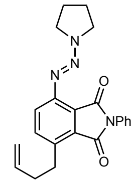

23
^{13}C NMR
$CDCl_3$, 100 MHz

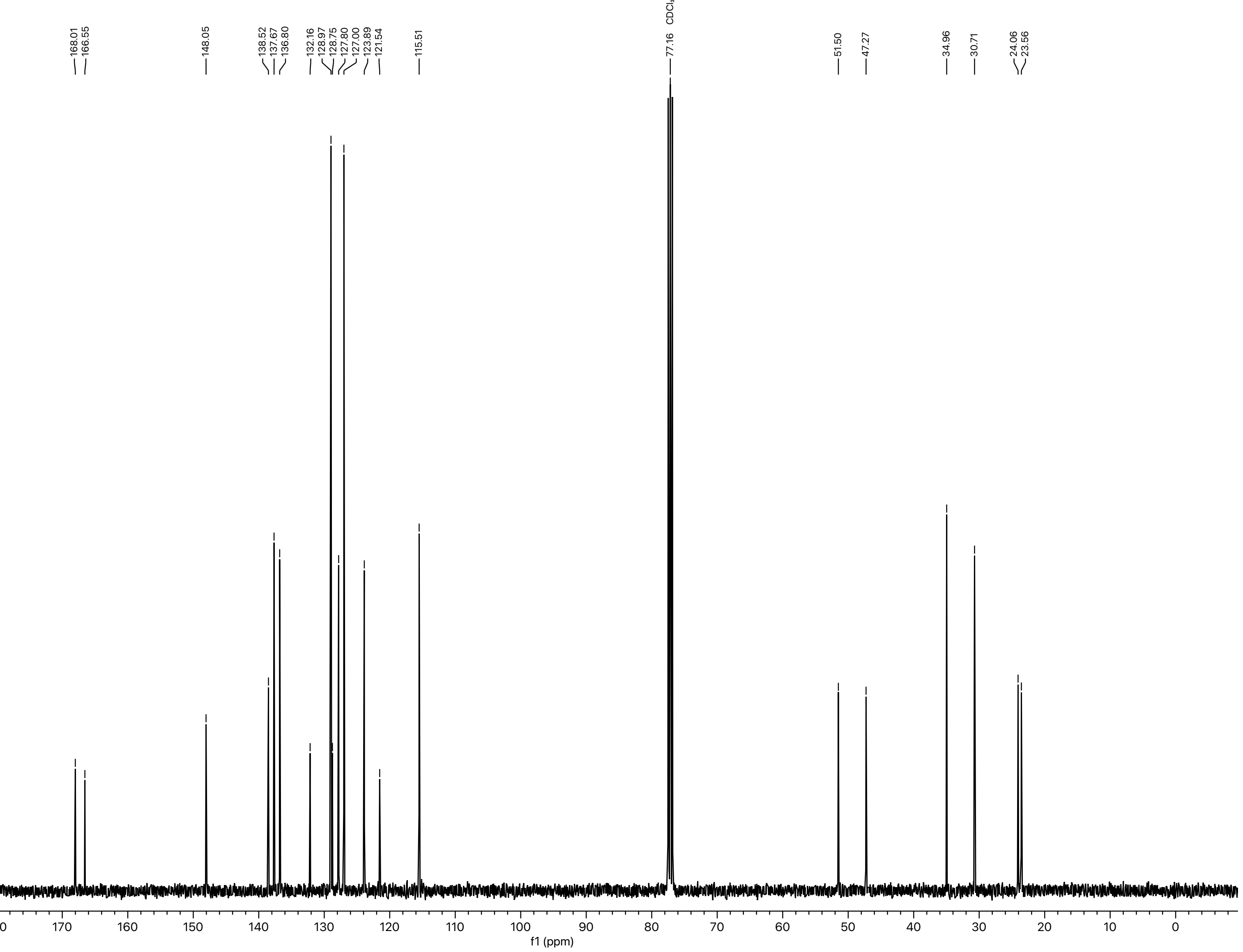

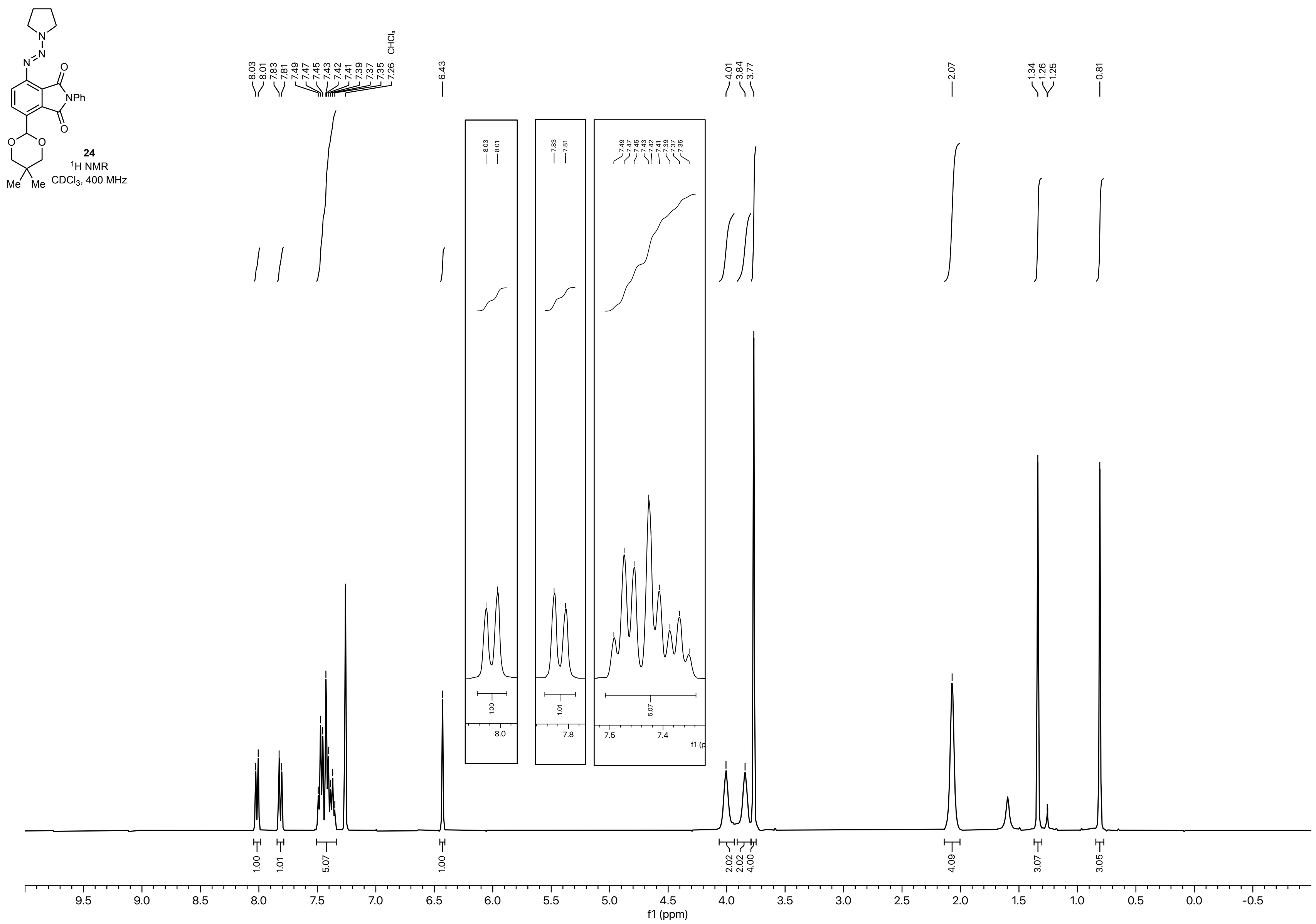

NPh
24
^{1}H NMR
$CDCl_3$, 400 MHz
Me Me
8.03 8.01 7.83 7.81 7.49 7.47 7.45 7.43 7.42 7.41 7.39 7.37 7.35 7.26 $CHCl_3$
6.43
4.01 3.84 3.77
2.07
1.34 1.26 1.25
0.81
1.00 1.01 5.07 1.00 2.02 2.02 4.00 4.09 3.07 3.05
f1 (ppm)

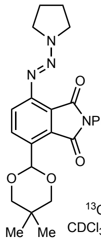

24
^{13}C NMR
$CDCl_3$, 100 MHz

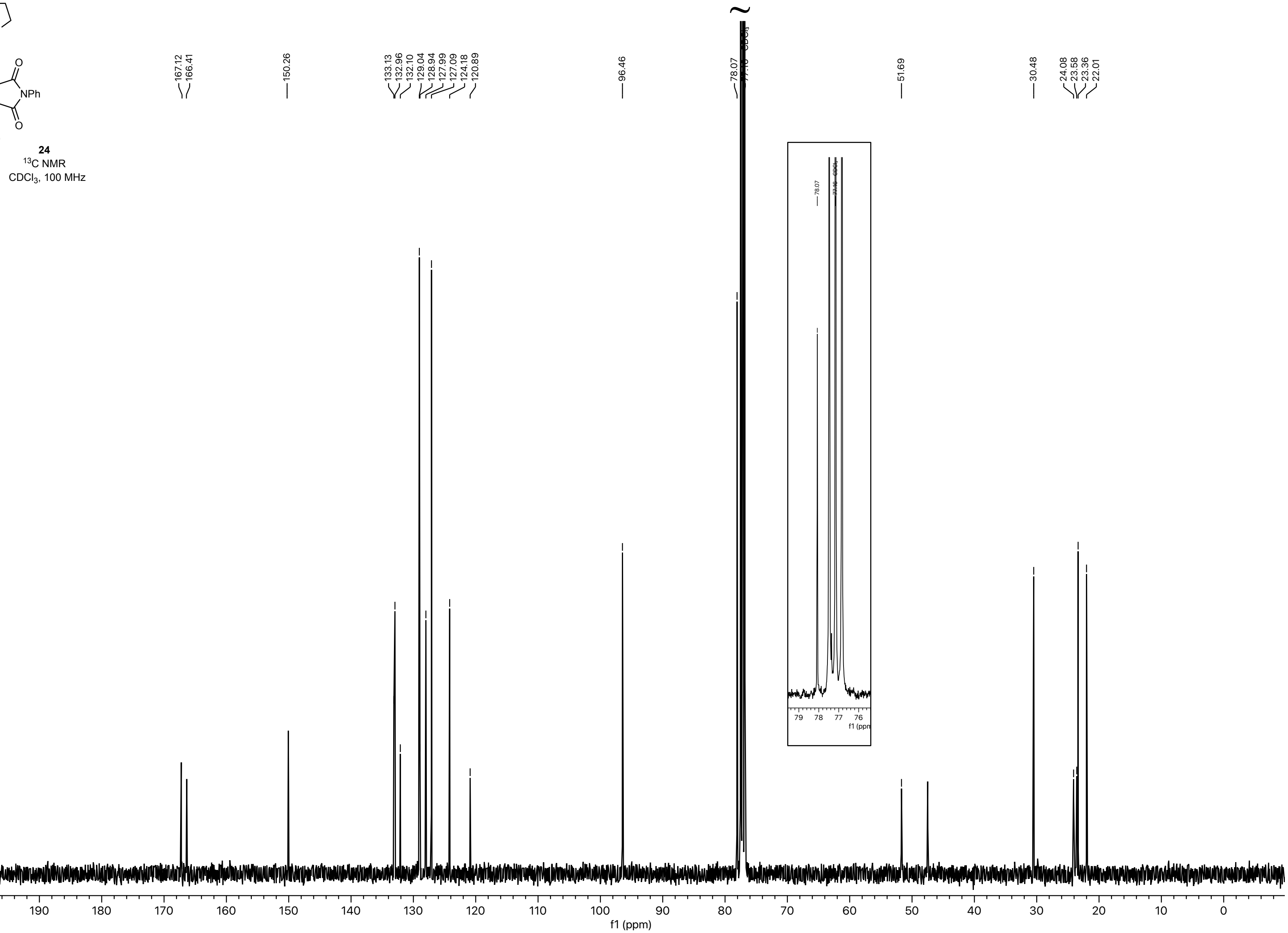

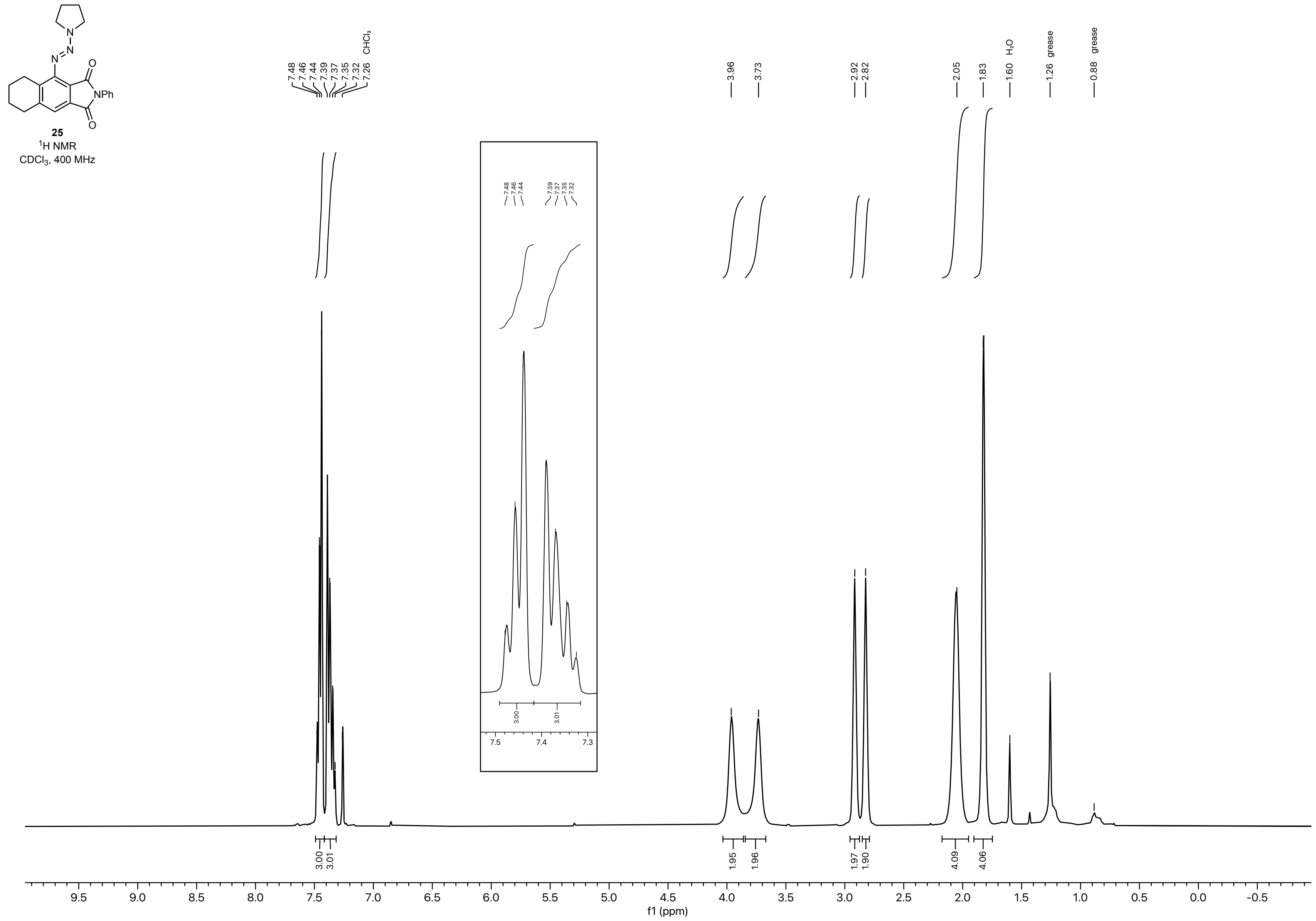

25
^{1}H NMR
$CDCl_3$, 400 MHz

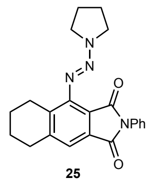

25
^{13}C NMR
$CDCl_3$, 100 MHz

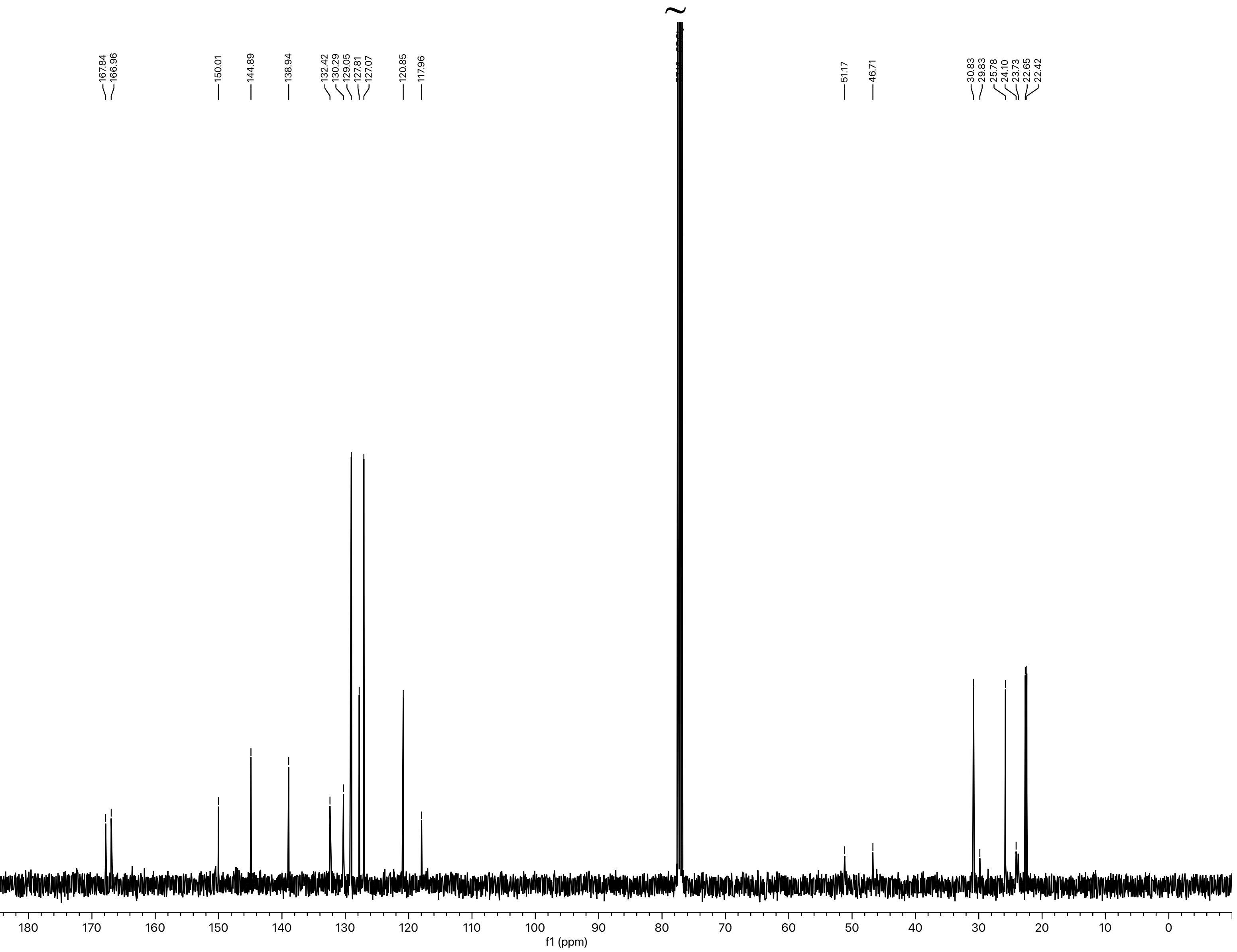

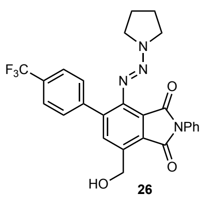

26

^{1}H NMR

$CDCl_3$, 400 MHz

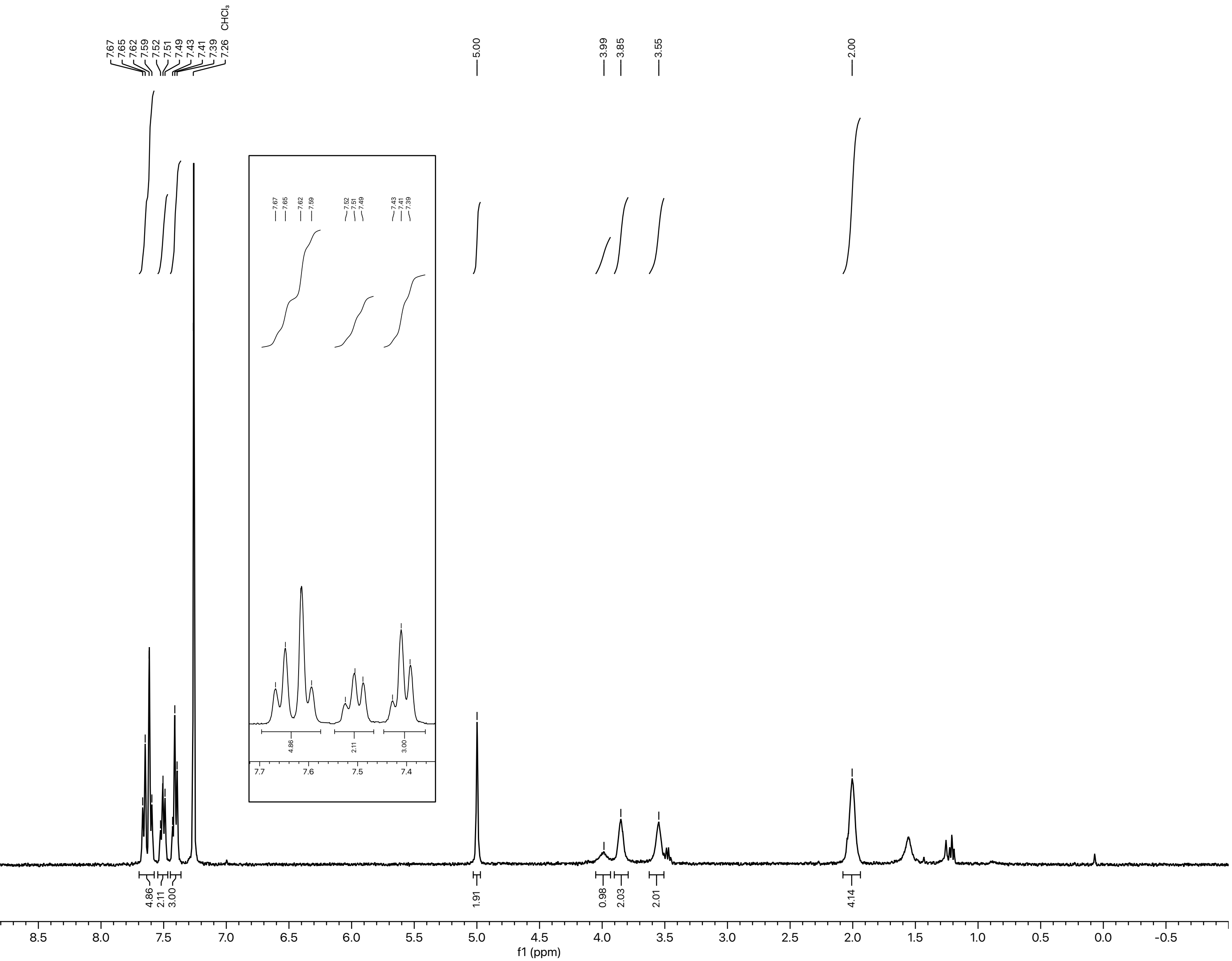

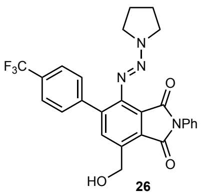

26

^{13}C NMR

$CDCl_3$, 100 MHz

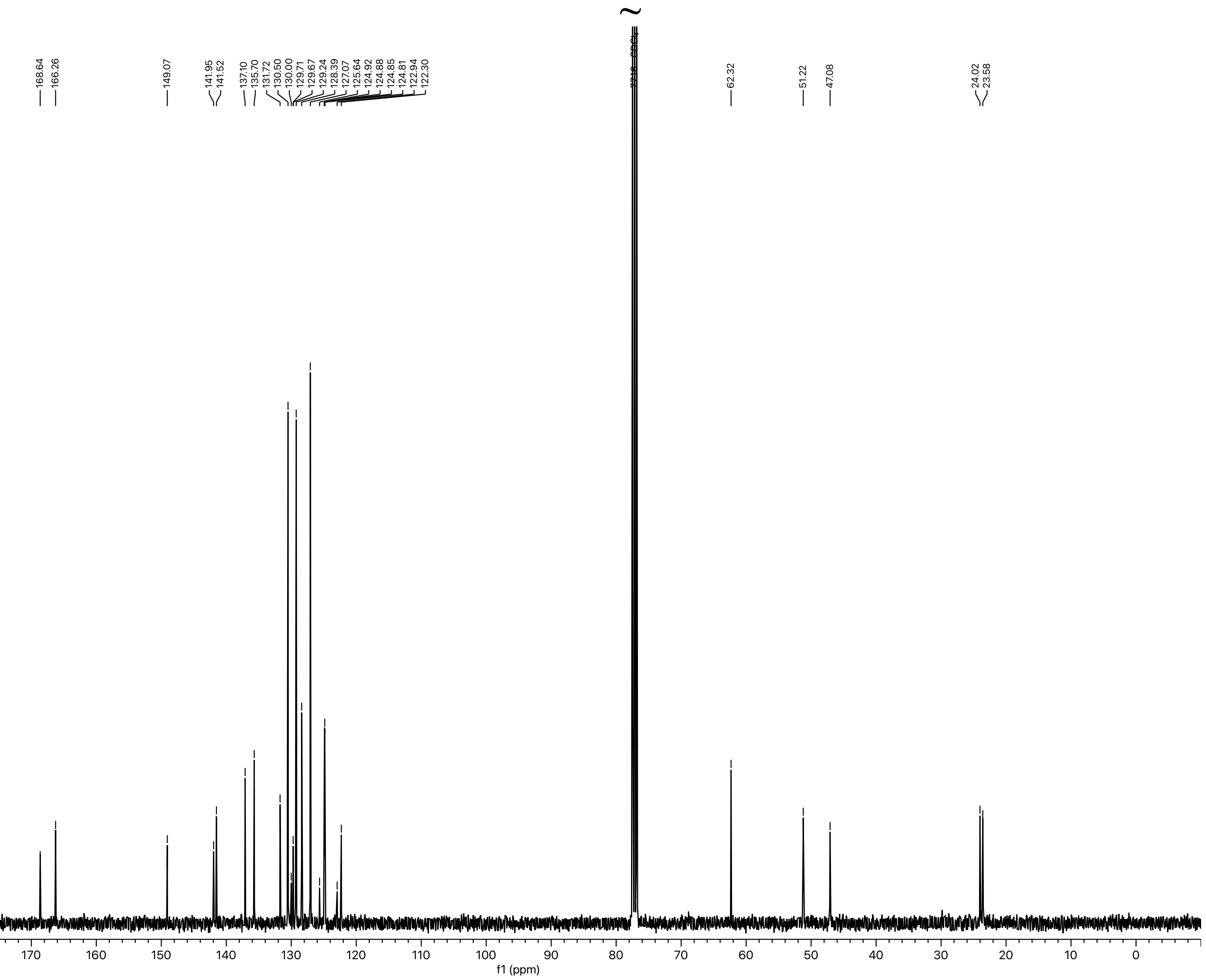

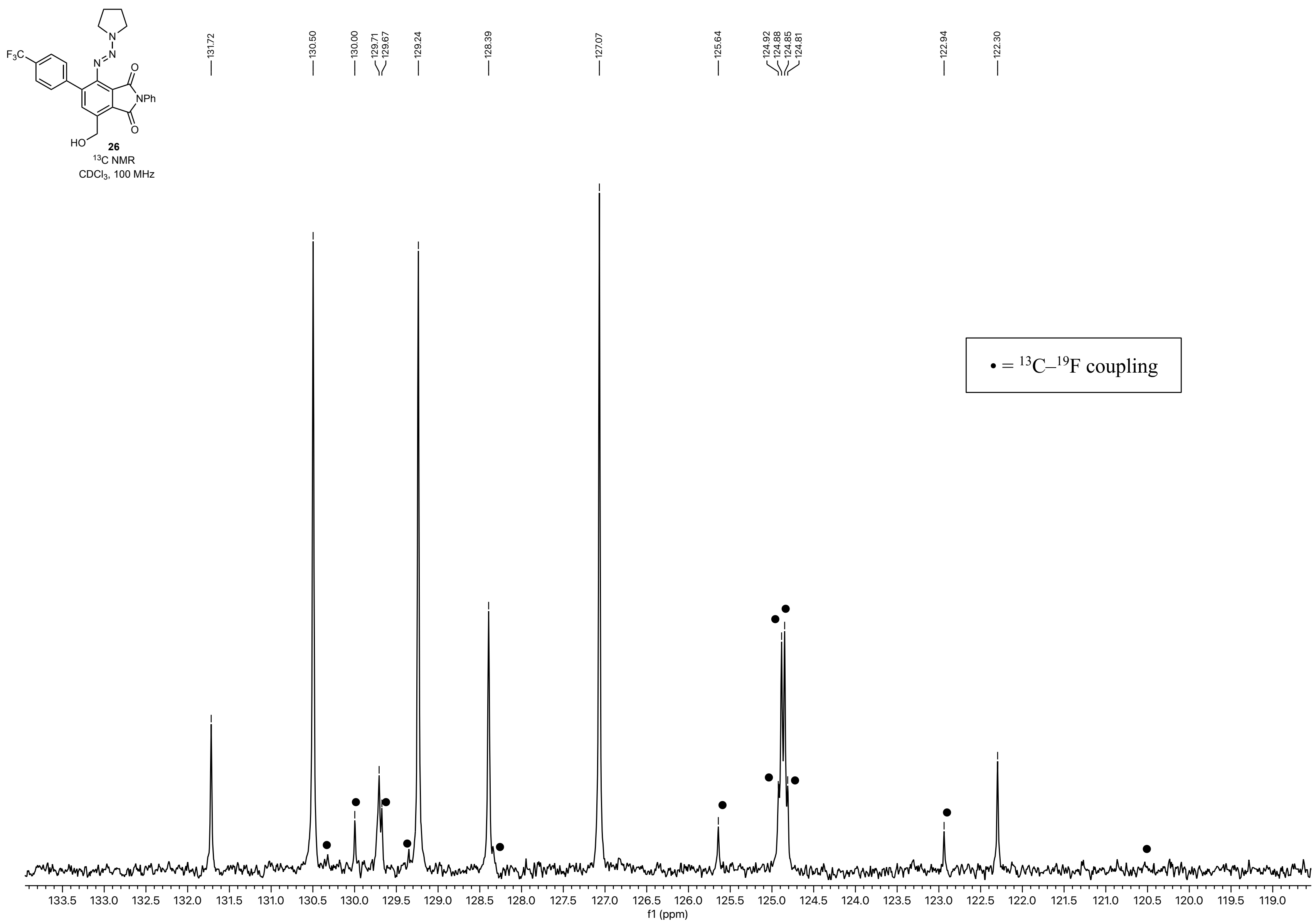
26
^{13}C NMR
$CDCl_3$, 100 MHz
131.72
130.50
130.00
129.71
129.67
129.24
128.39
127.07
125.64
124.92
124.88
124.85
124.81
122.94
122.30
• = ^{13}C–^{19}F coupling
f1 (ppm)

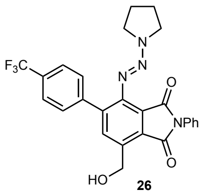

26
^{19}F NMR
$CDCl_3$, 377 MHz

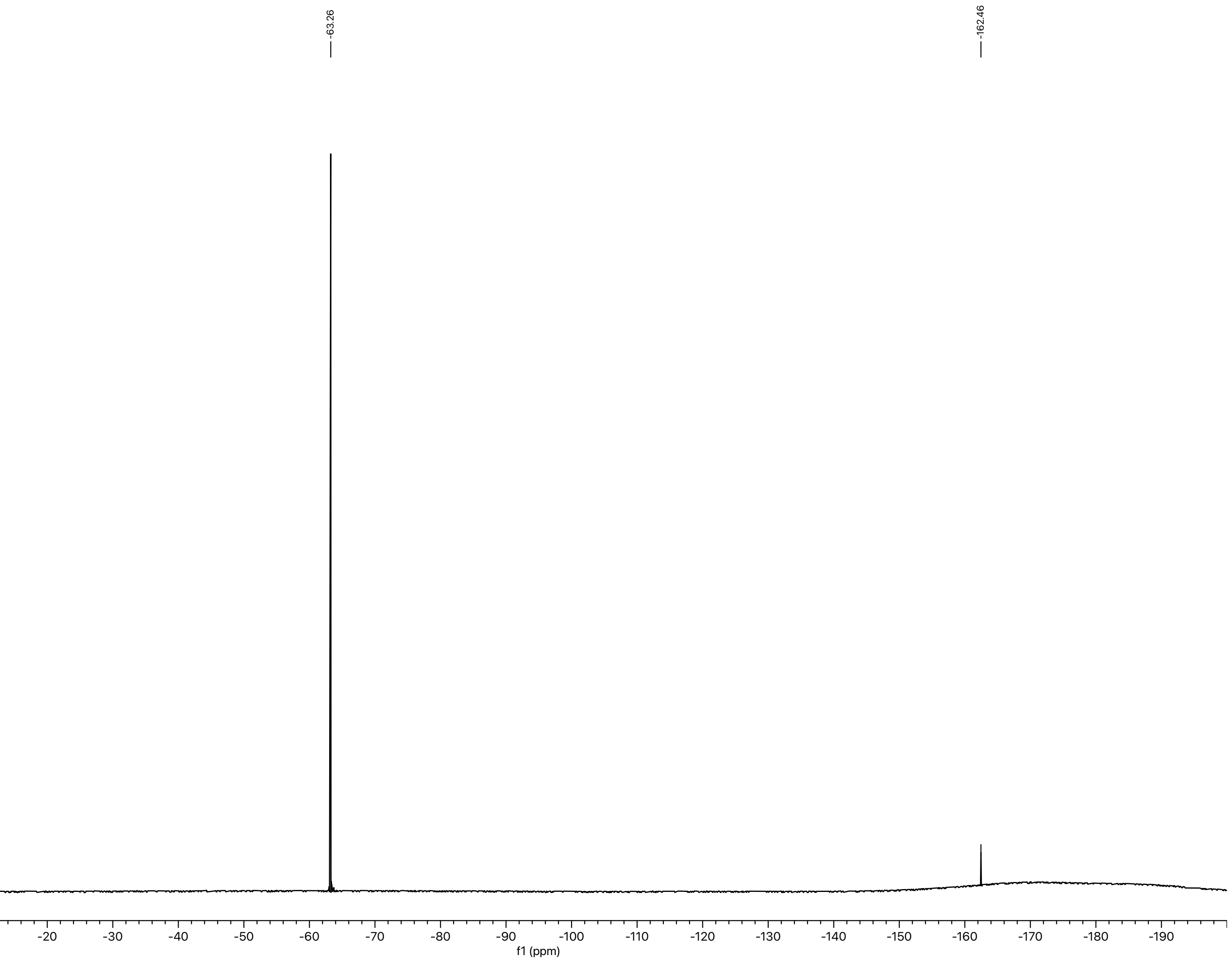

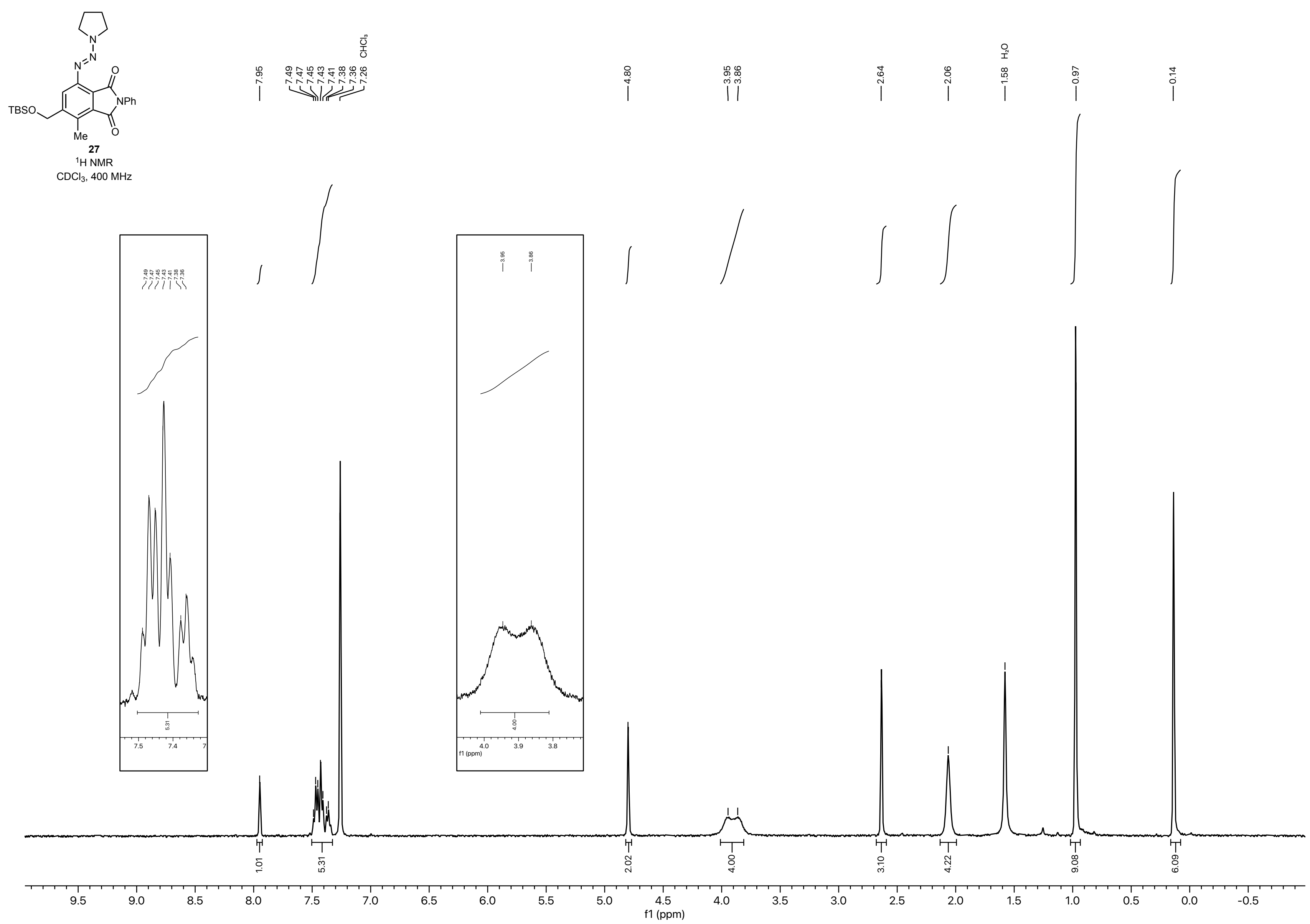

TBSO
NPh
Me
27
¹H NMR
CDCl3, 400 MHz
7.95
7.49
7.47
7.45
7.43
7.41
7.38
7.36
7.26
CHCl3
4.80
3.95
3.86
2.64
2.06
1.58
H2O
0.97
0.14
1.01
5.31
2.02
4.00
3.10
4.22
9.08
6.09
f1 (ppm)

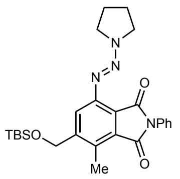

27
^{13}C NMR
$CDCl_3$, 100 MHz

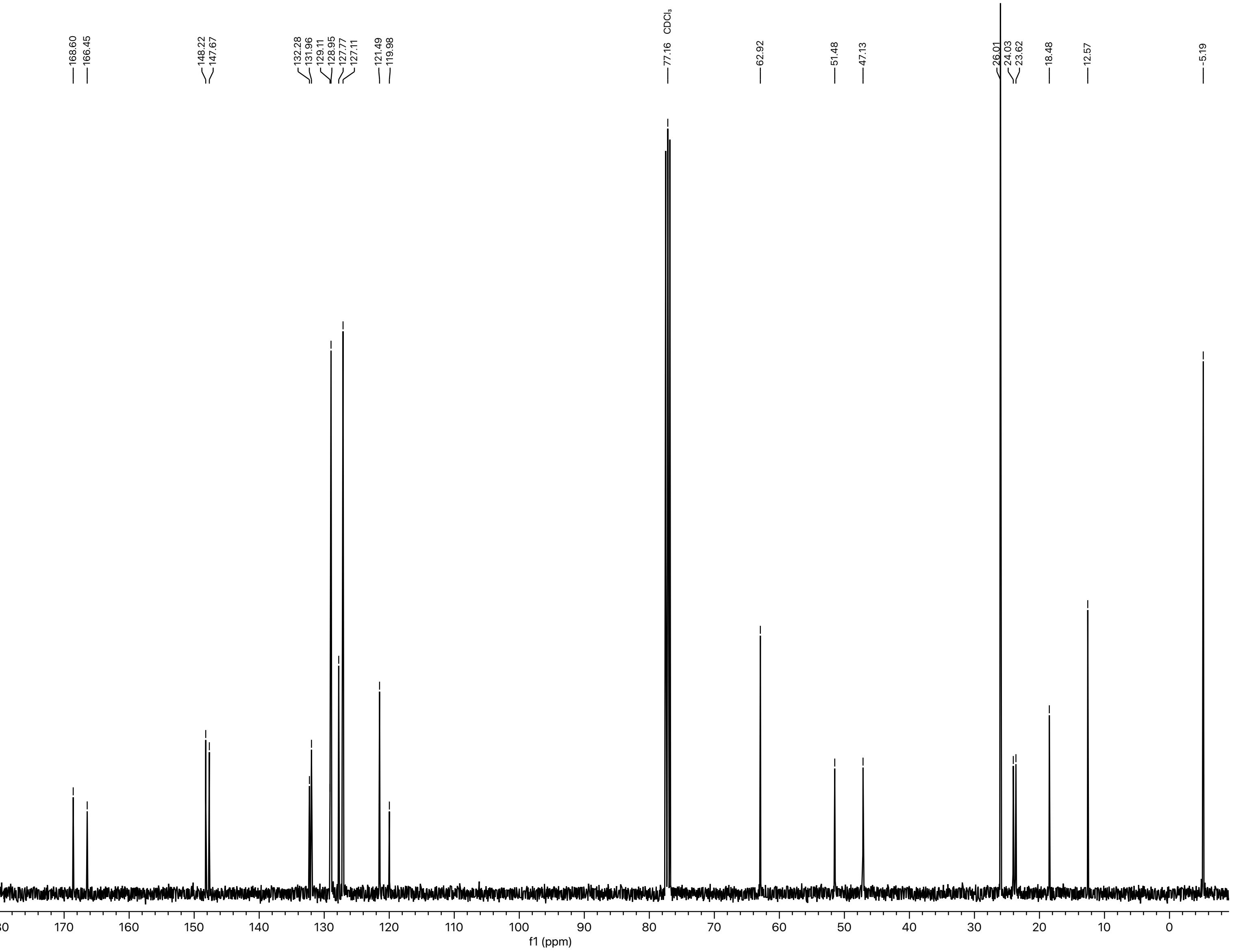

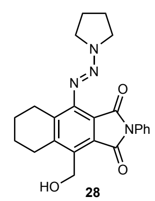

28

^{1}H NMR

$CDCl_3$, 400 MHz

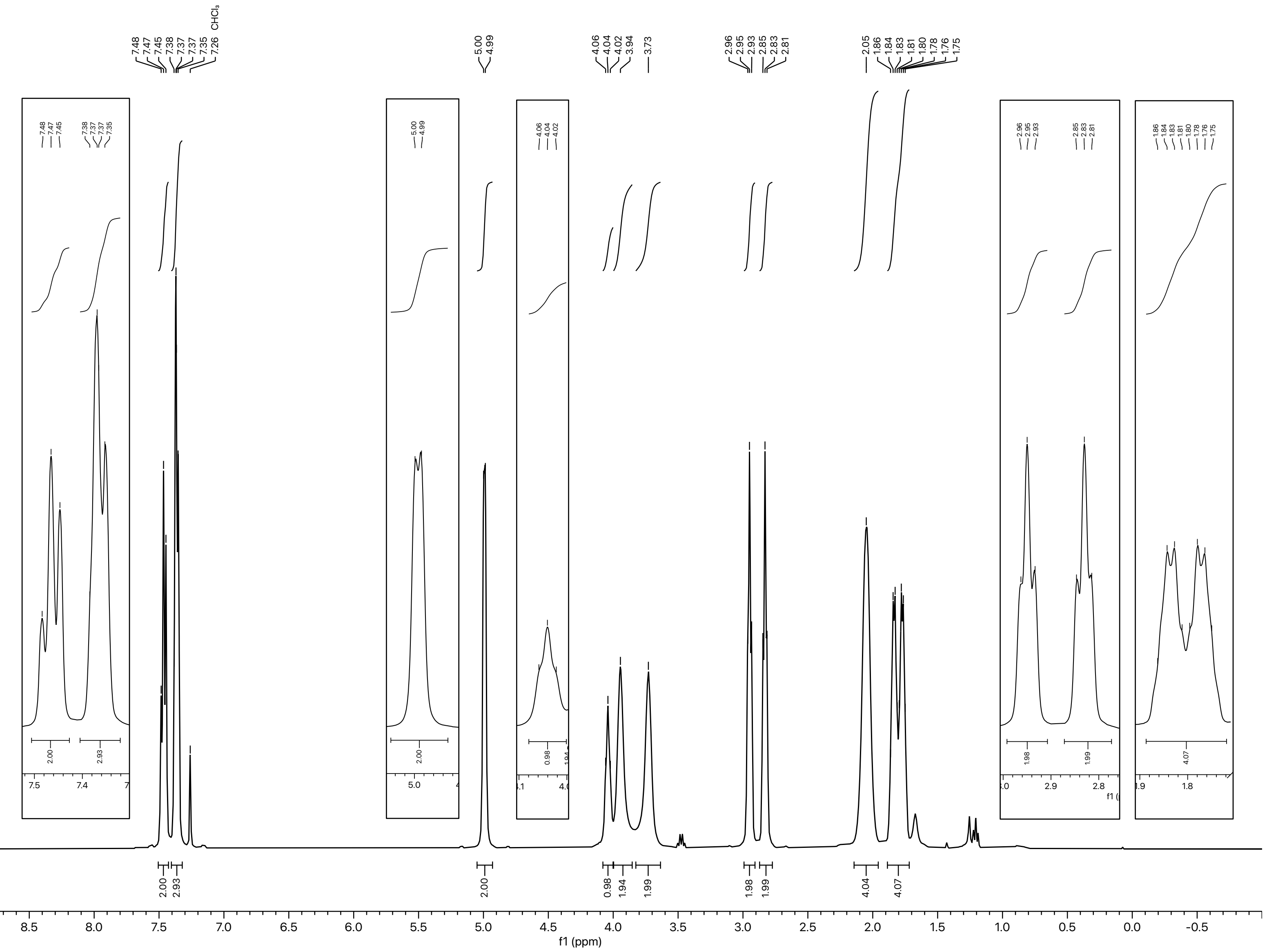

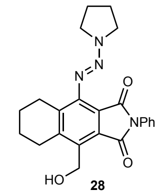

28
^{13}C NMR
$CDCl_3$, 100 MHz

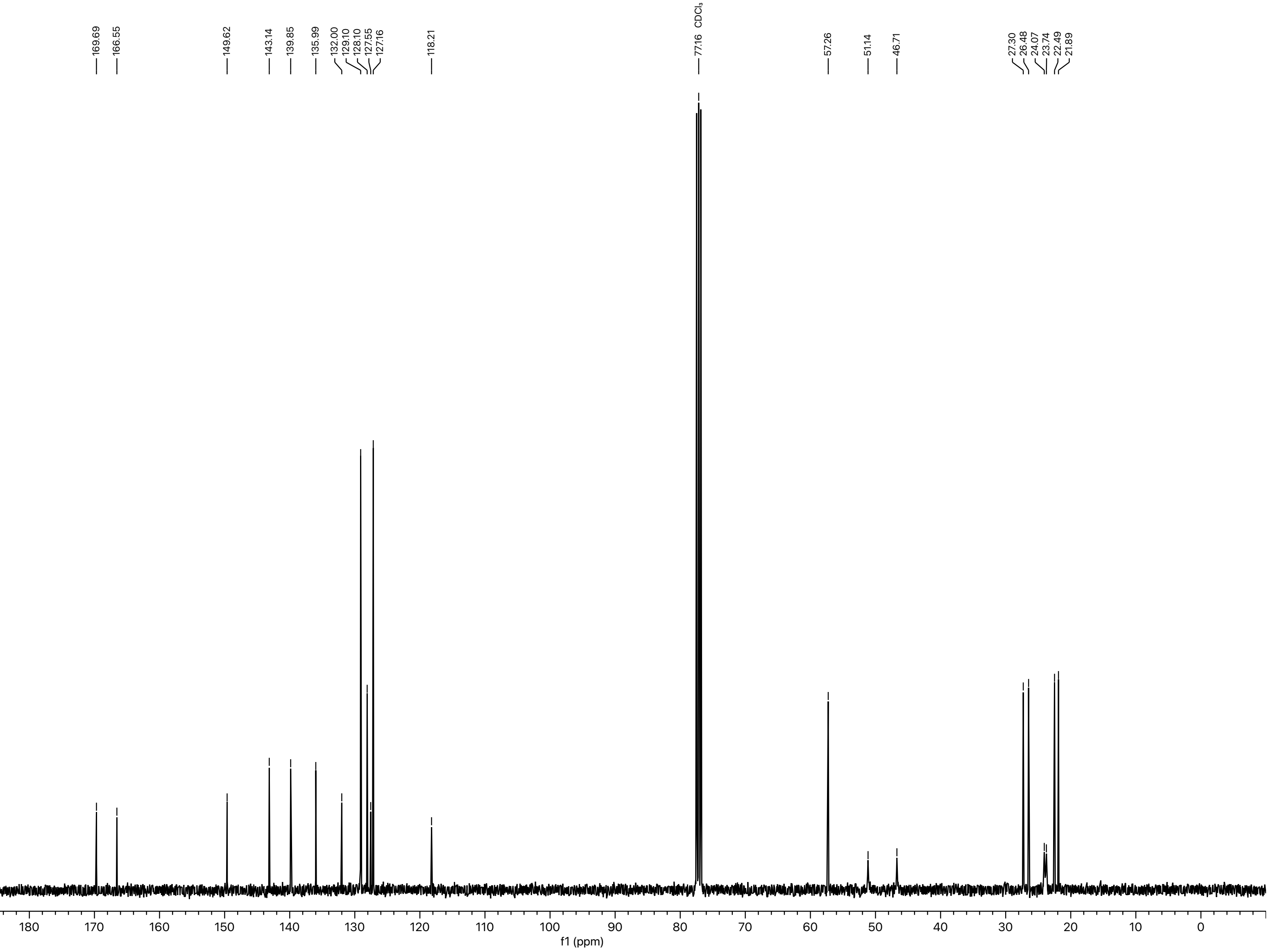

Dimethyl Acetylenedicarboxylate as Dienophile

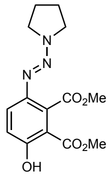

29
^{1}H NMR
$CDCl_3$, 400 MHz

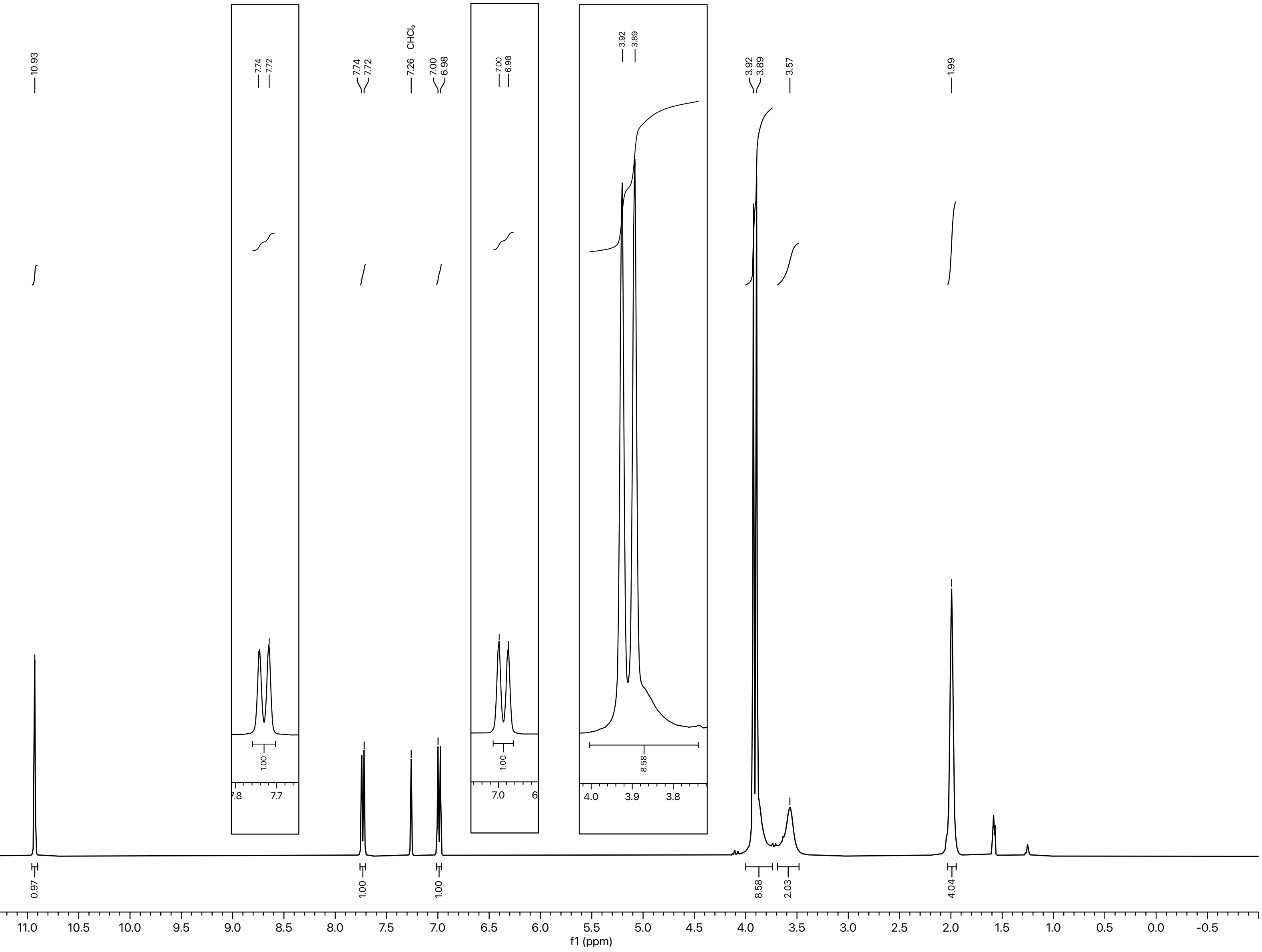

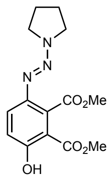

29
^{13}C NMR
$CDCl_3$, 100 MHz

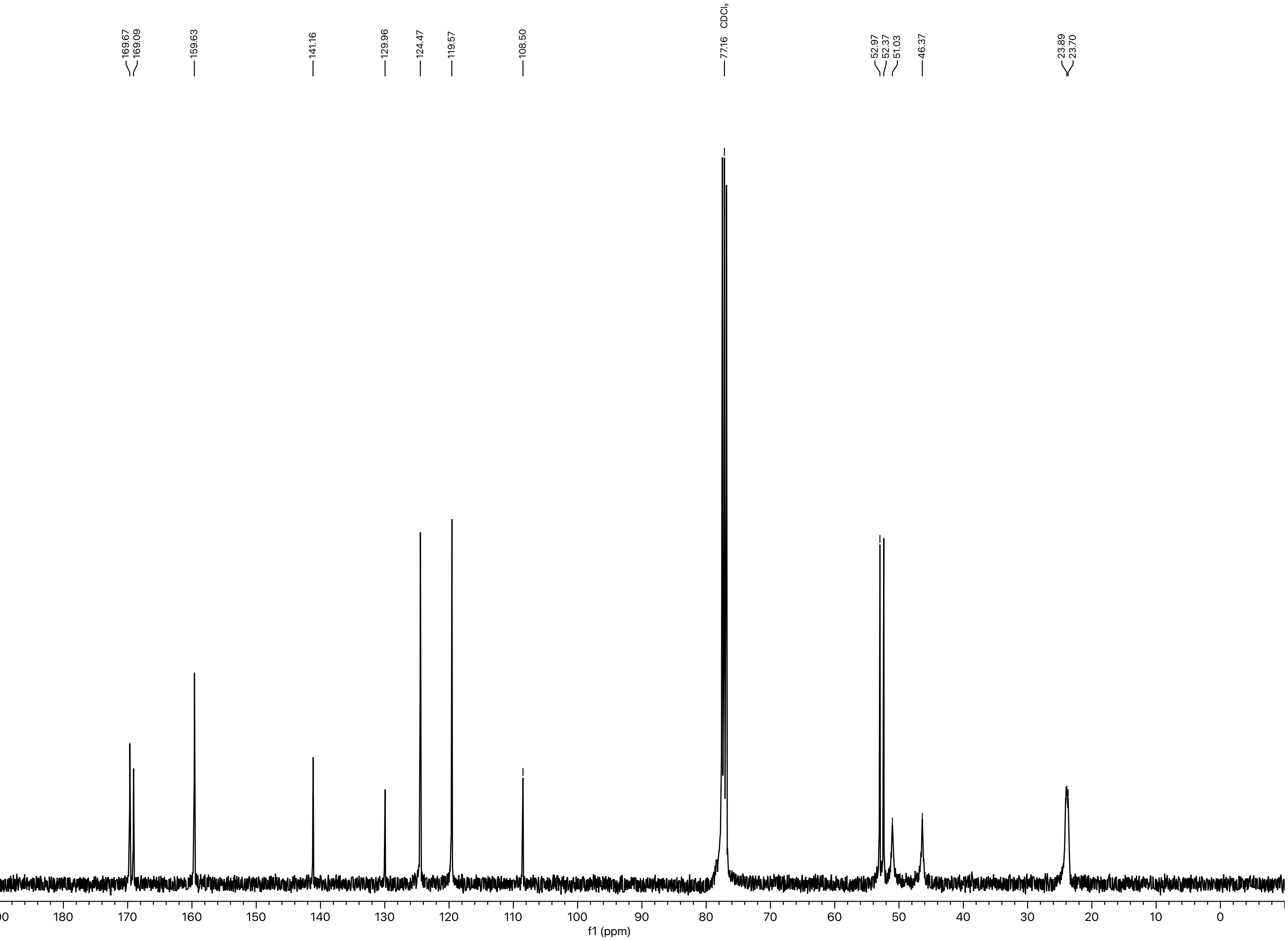

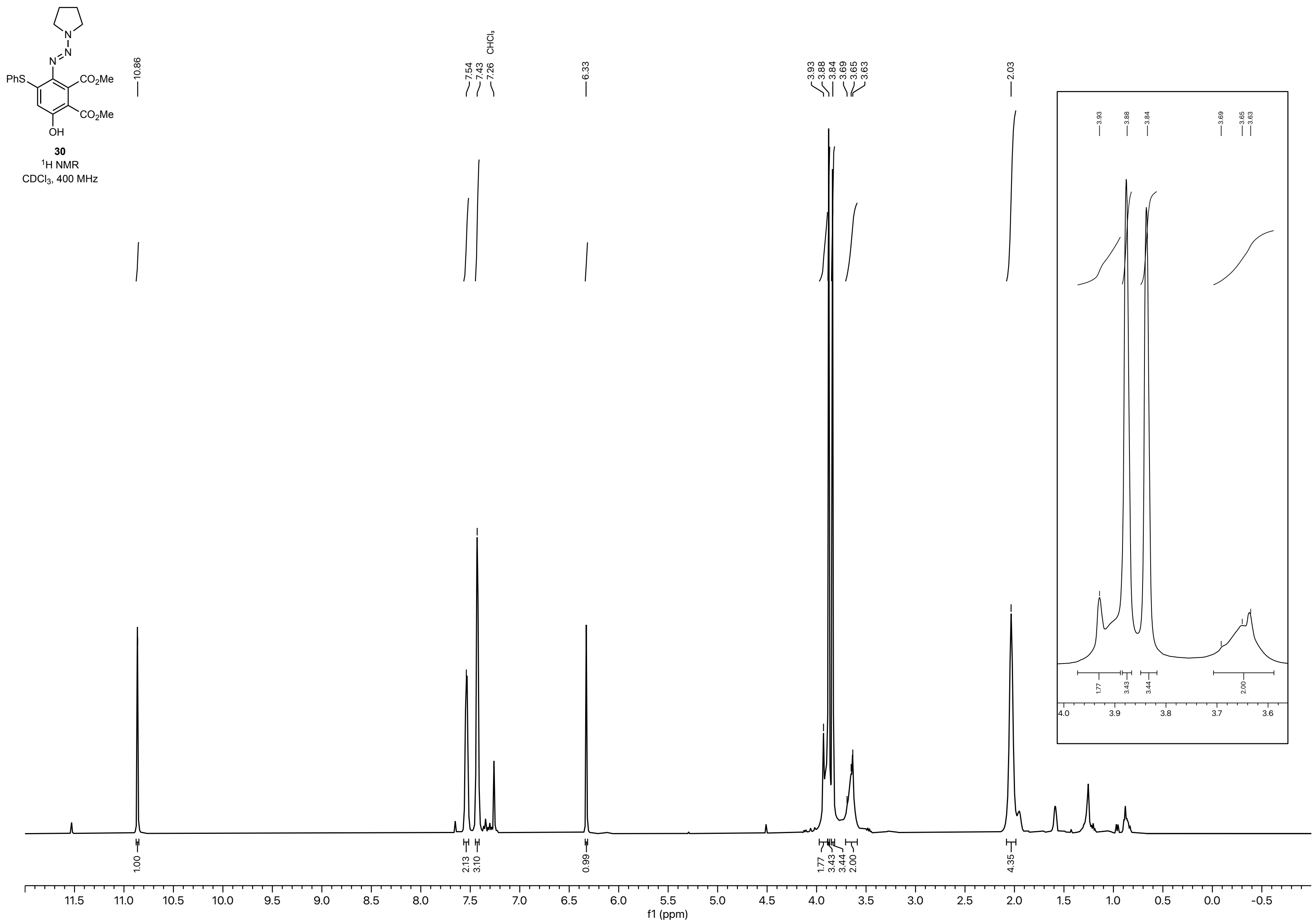

PhS
CO2Me
CO2Me
OH
N
N
N
30
1H NMR
CDCl3, 400 MHz
10.86
7.54
7.43
7.26 CHCl3
6.33
3.93
3.88
3.84
3.69
3.65
3.63
2.03
1.00
2.13
3.10
0.99
1.77
3.43
3.44
2.00
4.35
f1 (ppm)

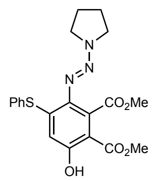

30
^{13}C NMR
$CDCl_3$, 100 MHz

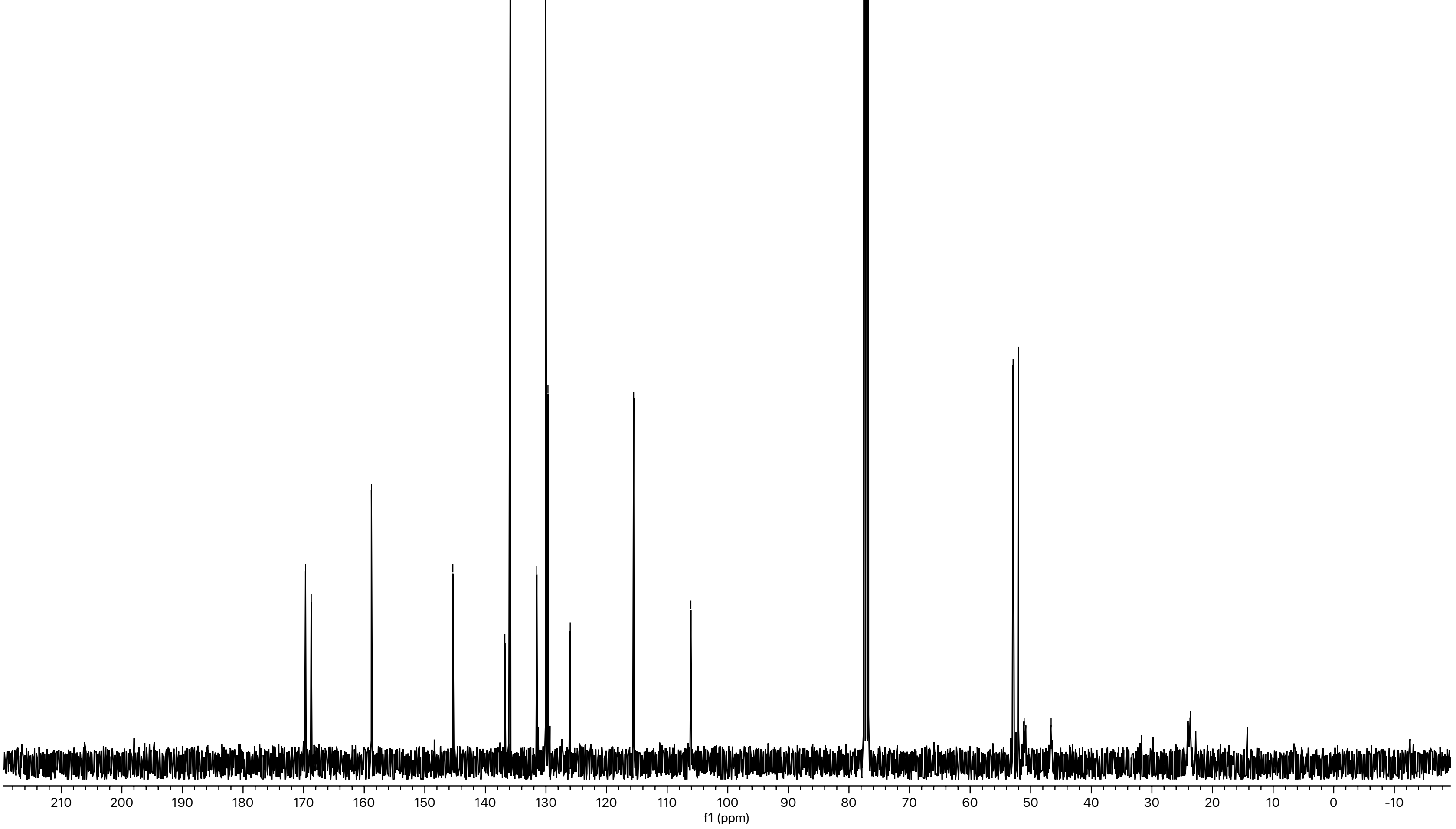

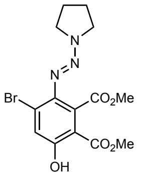

31
^1H NMR
$CDCl_3$, 900 MHz

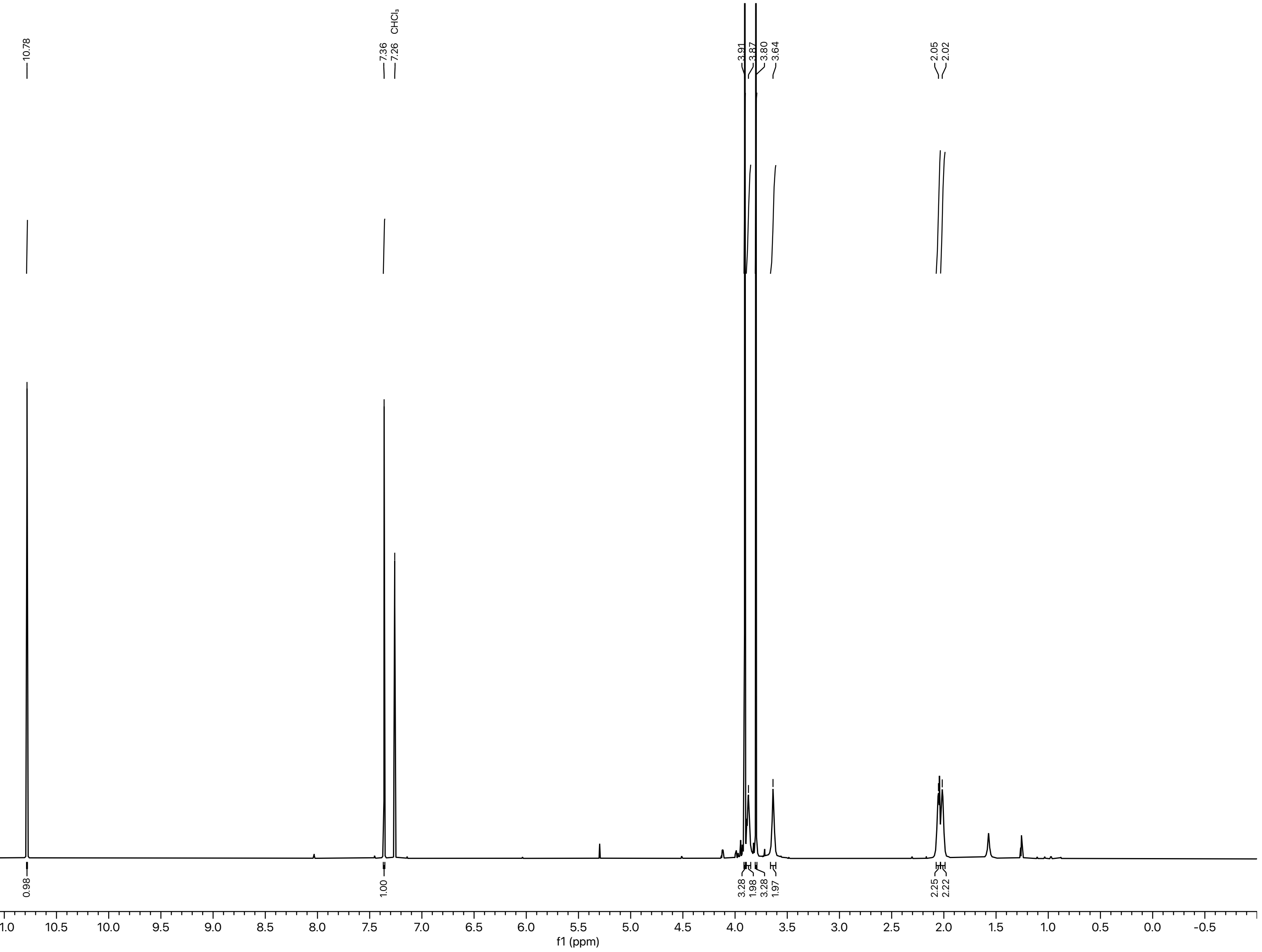

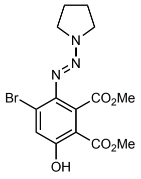

31
^{13}C NMR
$CDCl_3$, 225 MHz

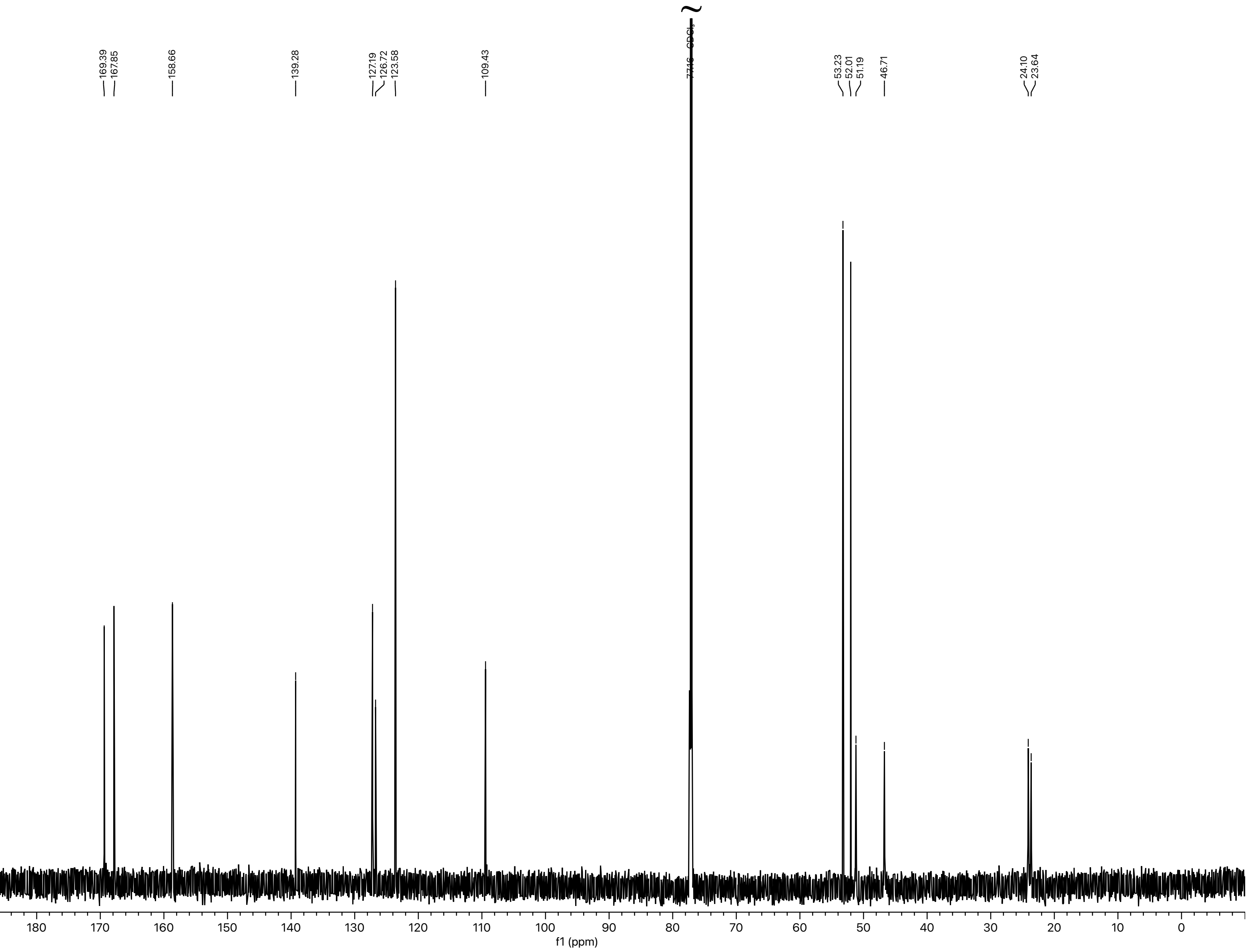

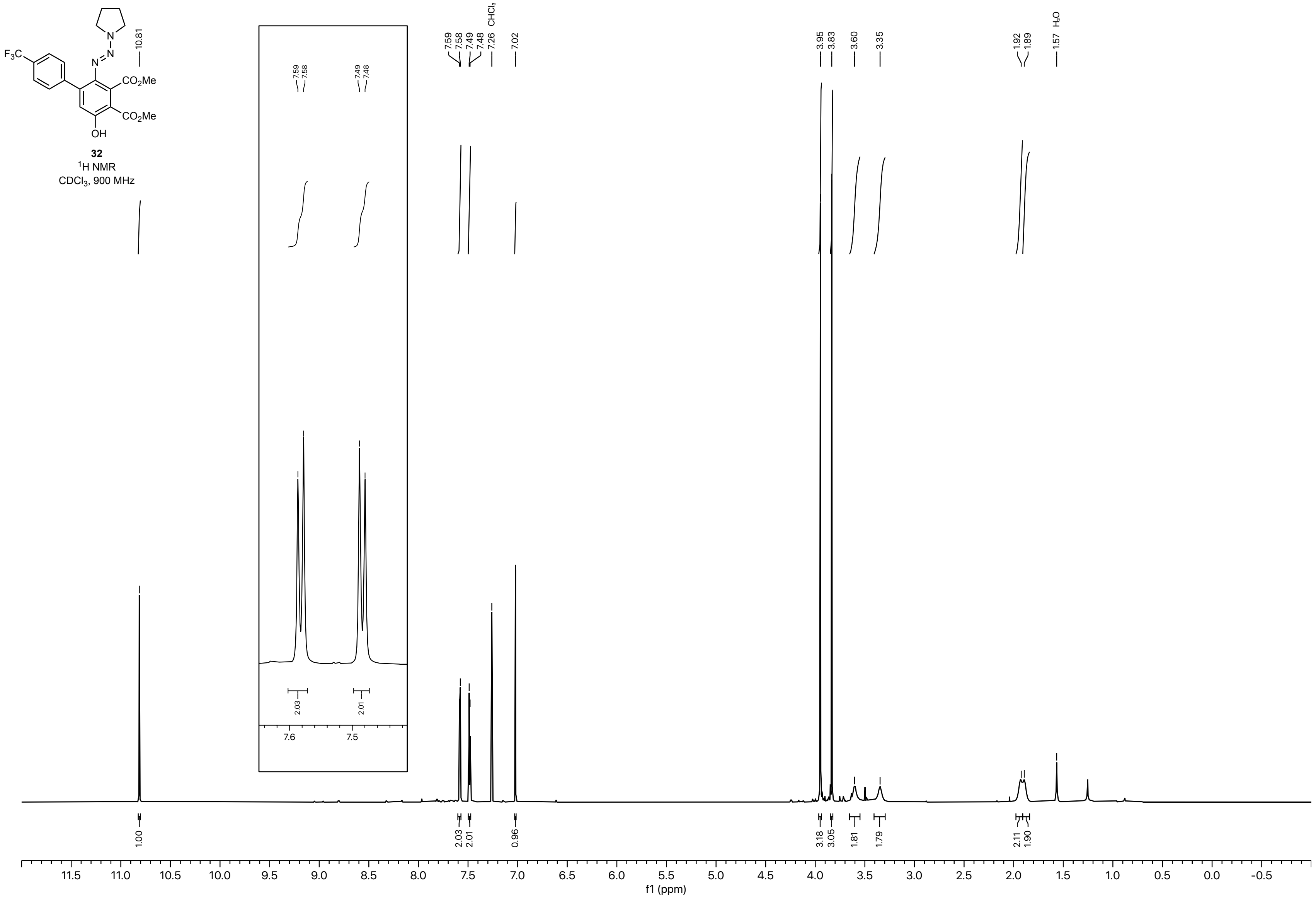

32
^{1}H NMR
$CDCl_3$, 900 MHz
CO2Me
CO2Me
OH
F3C
10.81
7.59
7.58
7.49
7.48
7.26 CHCl3
7.02
3.95
3.83
3.60
3.35
1.92
1.89
1.57 H2O
1.00
2.03
2.01
0.96
3.18
3.05
1.81
1.79
2.11
1.90
f1 (ppm)

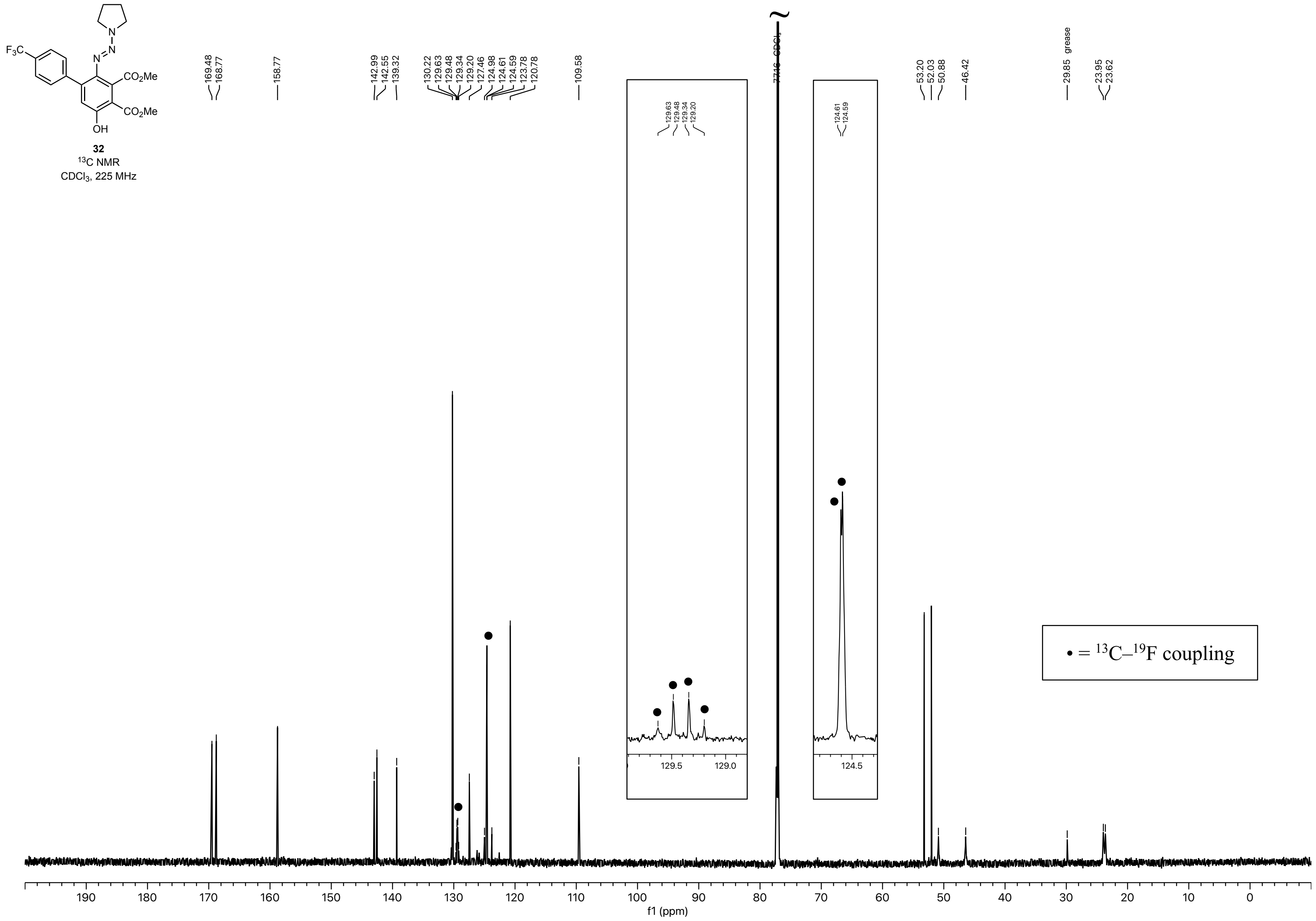
F3C
CO2Me
CO2Me
OH
N
N
N
32
13C NMR
CDCl3, 225 MHz
169.48
168.77
158.77
142.99
142.55
139.32
130.22
129.63
129.48
129.34
129.20
127.46
124.98
124.61
124.59
123.78
120.78
109.58
77.16 CDCl3
53.20
52.03
50.88
46.42
29.85 grease
23.95
23.62
• = 13C–19F coupling
f1 (ppm)

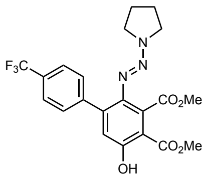

32
^{19}F NMR
$CDCl_3$, 377 MHz

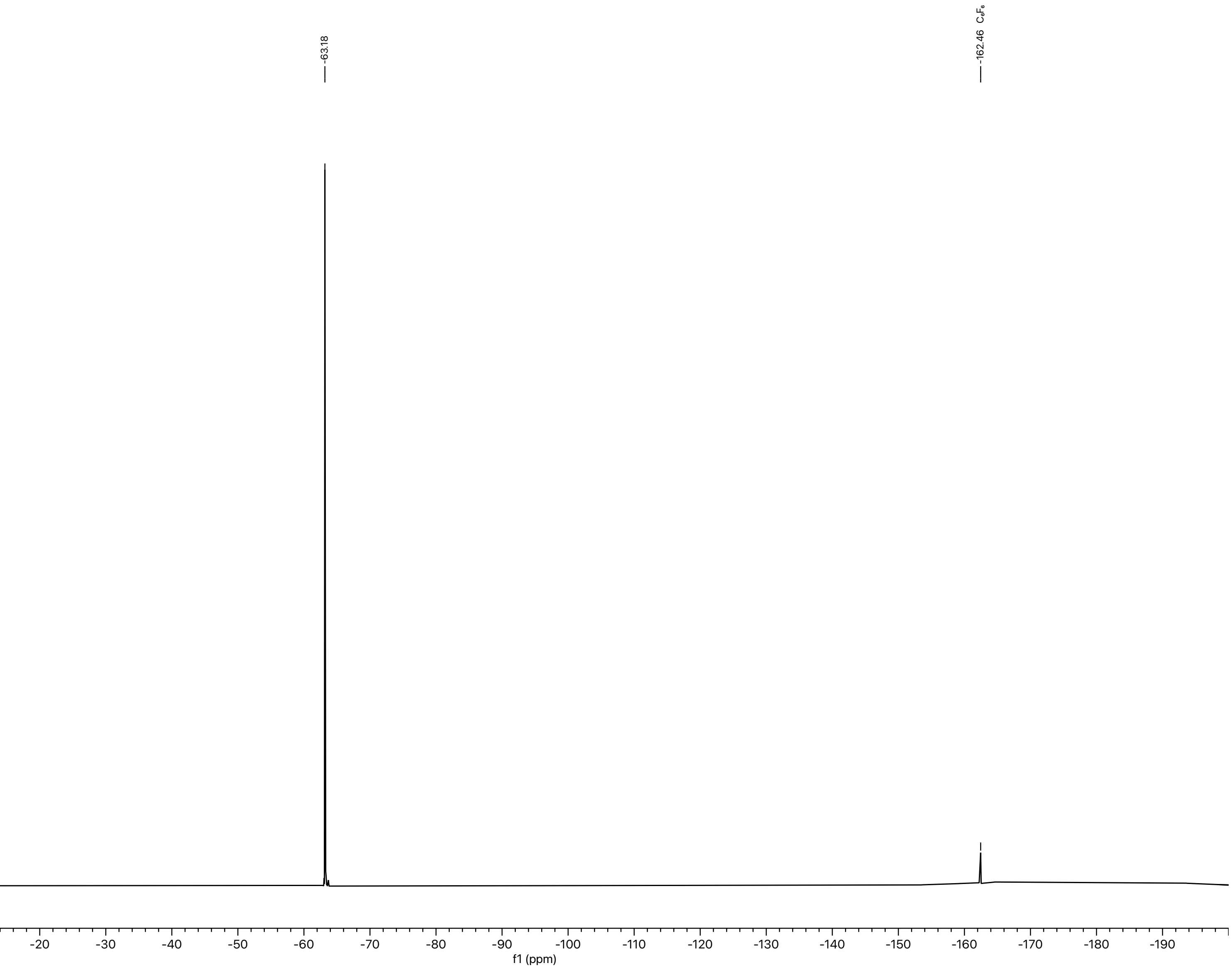

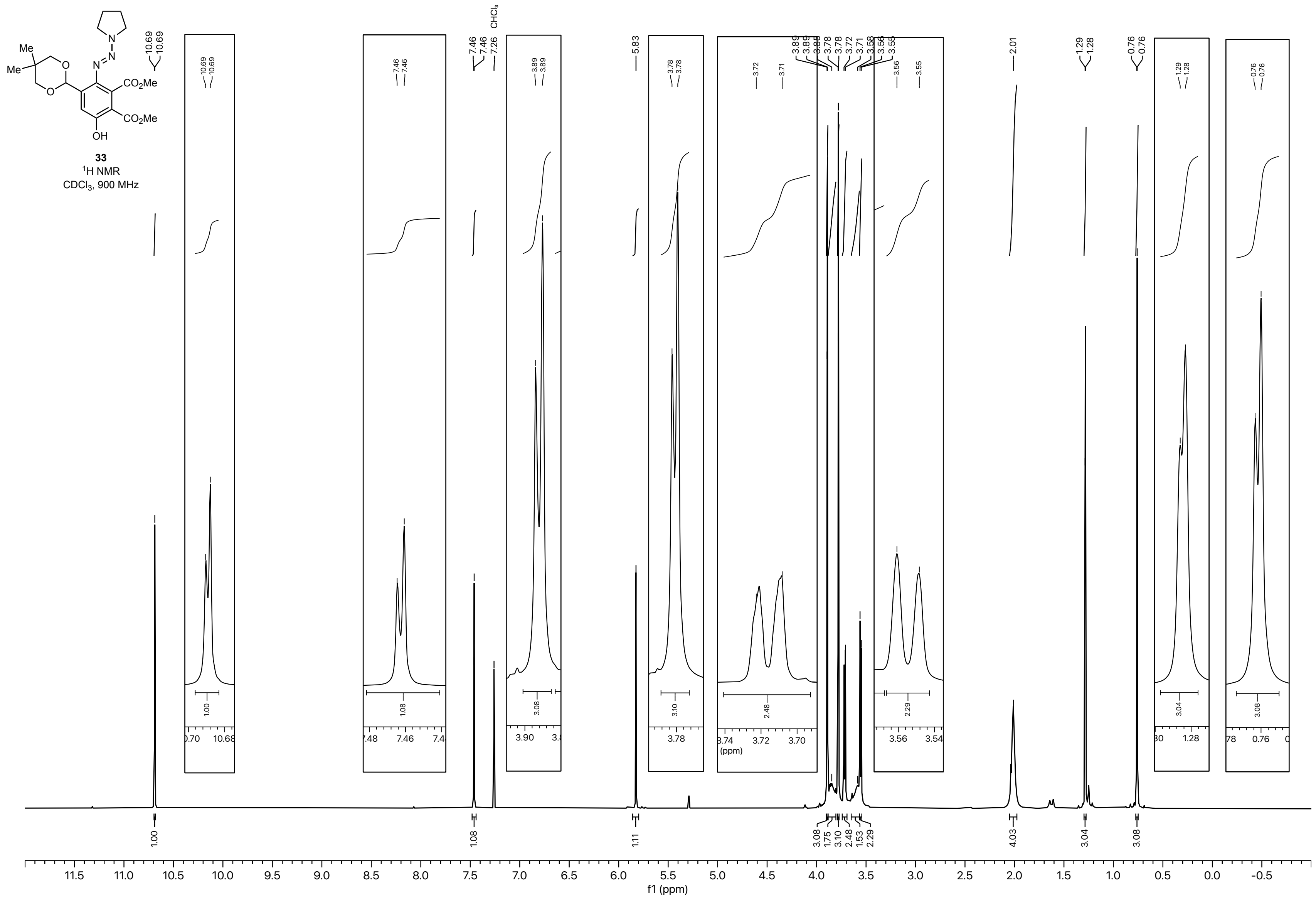

33
^{1}H NMR
$CDCl_3$, 900 MHz
CHCl₃
f1 (ppm)

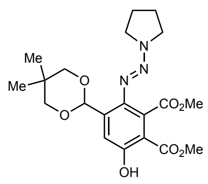

33
^{13}C NMR
$CDCl_3$, 225 MHz

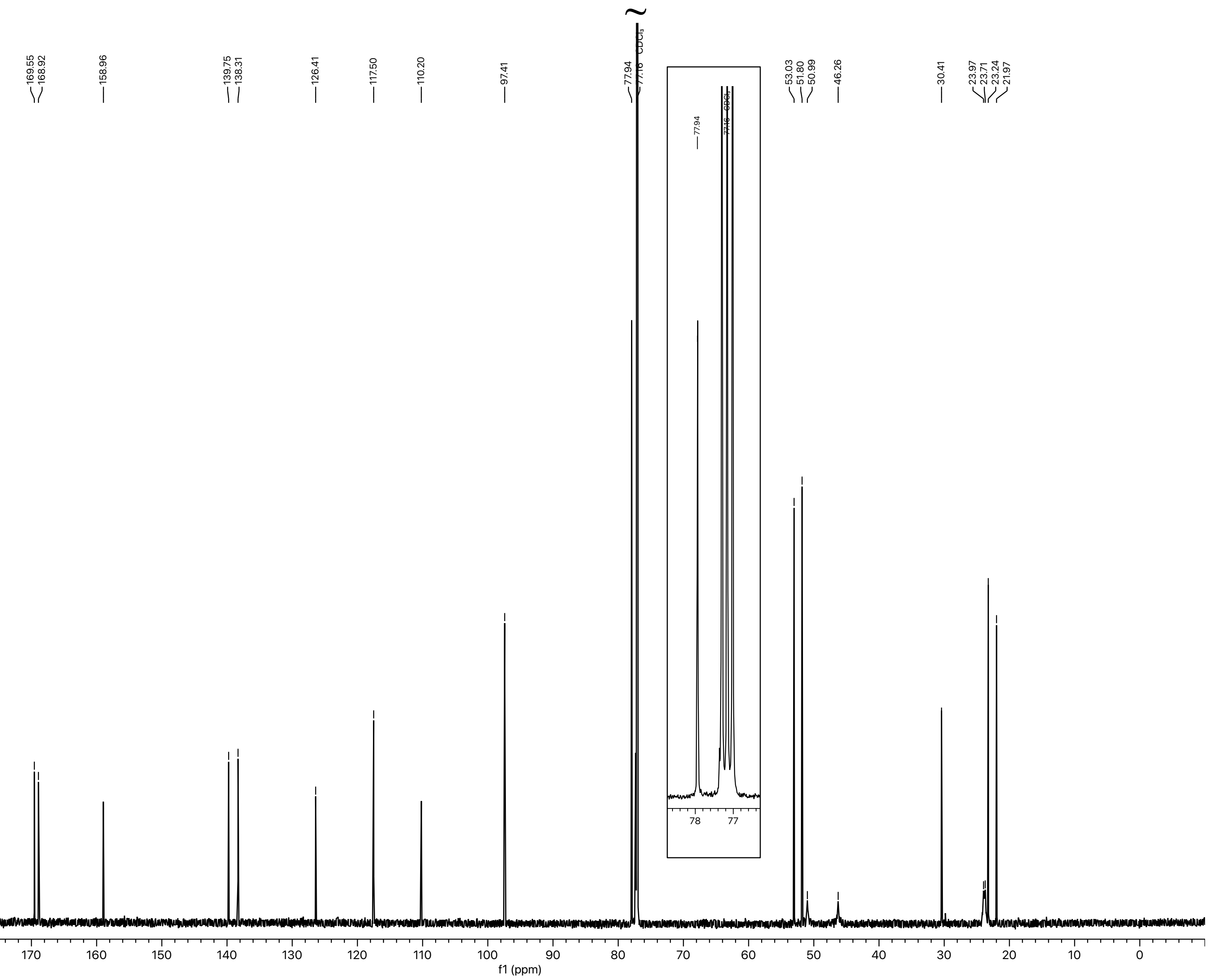

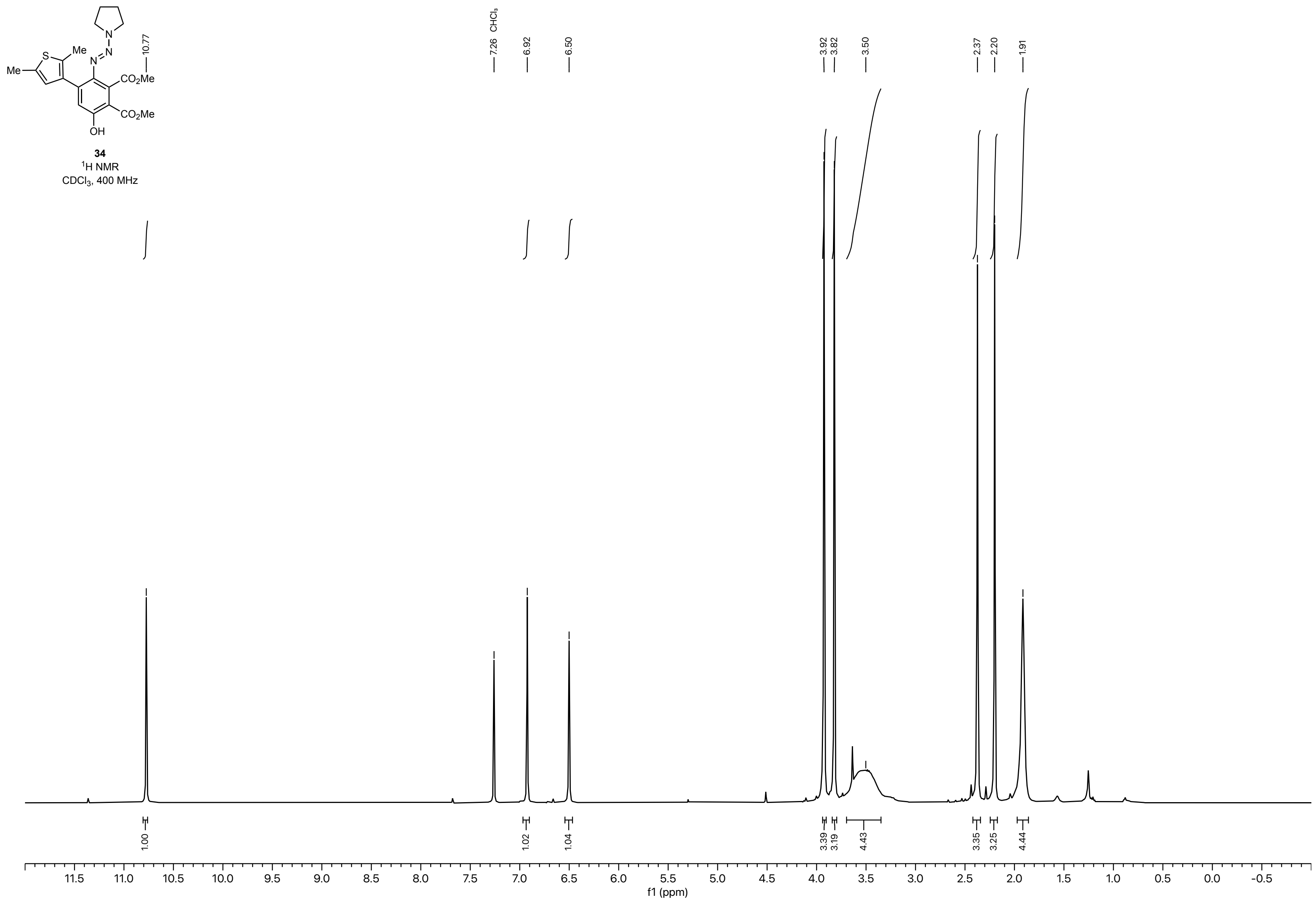
34
1H NMR
CDCl3, 400 MHz
10.77
7.26 CHCl3
6.92
6.50
3.92
3.82
3.50
2.37
2.20
1.91
1.00
1.02
1.04
3.39
3.19
4.43
3.35
3.25
4.44
f1 (ppm)

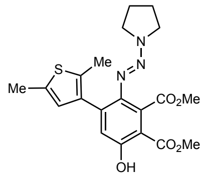

34
^{13}C NMR
$CDCl_3$, 100 MHz

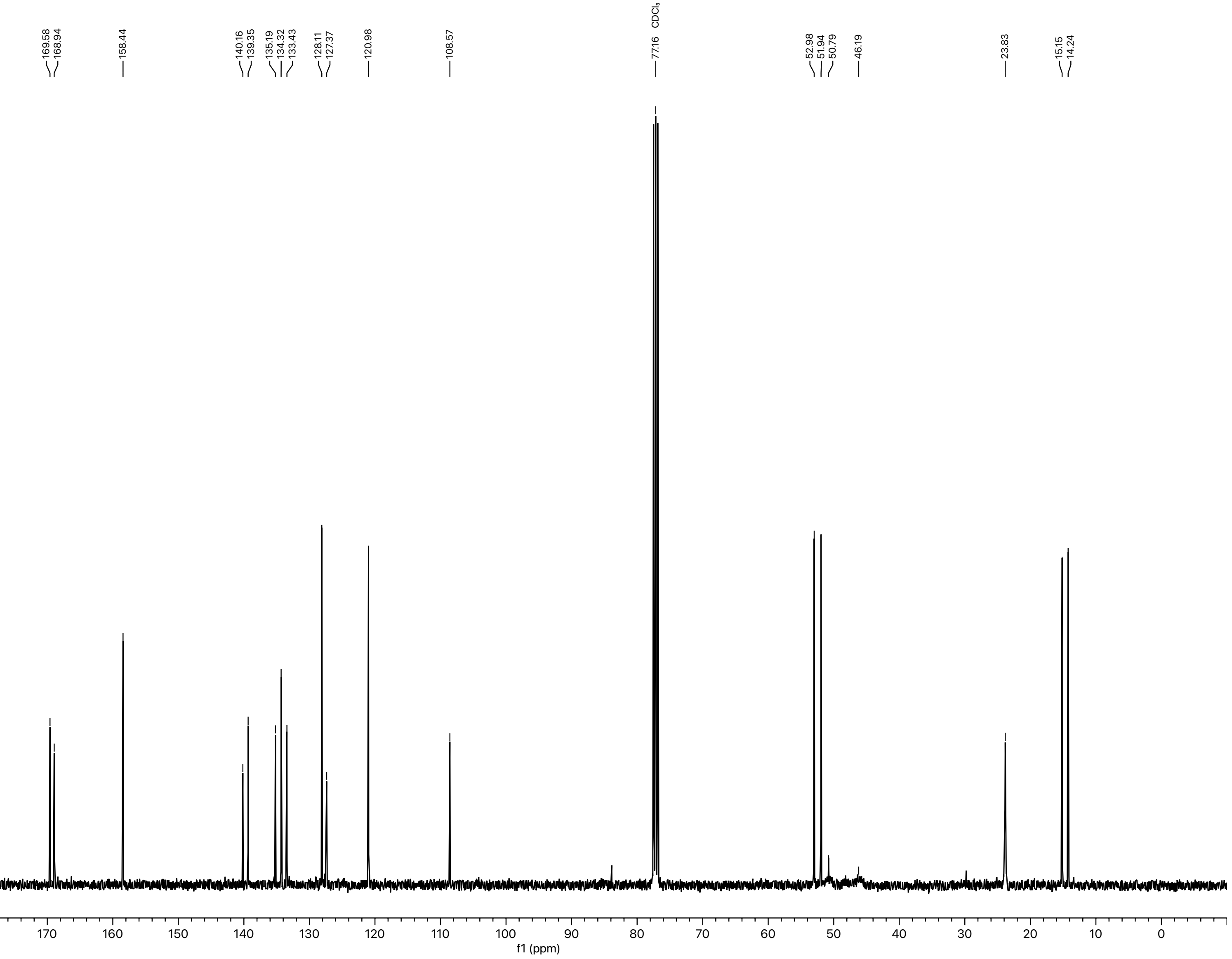

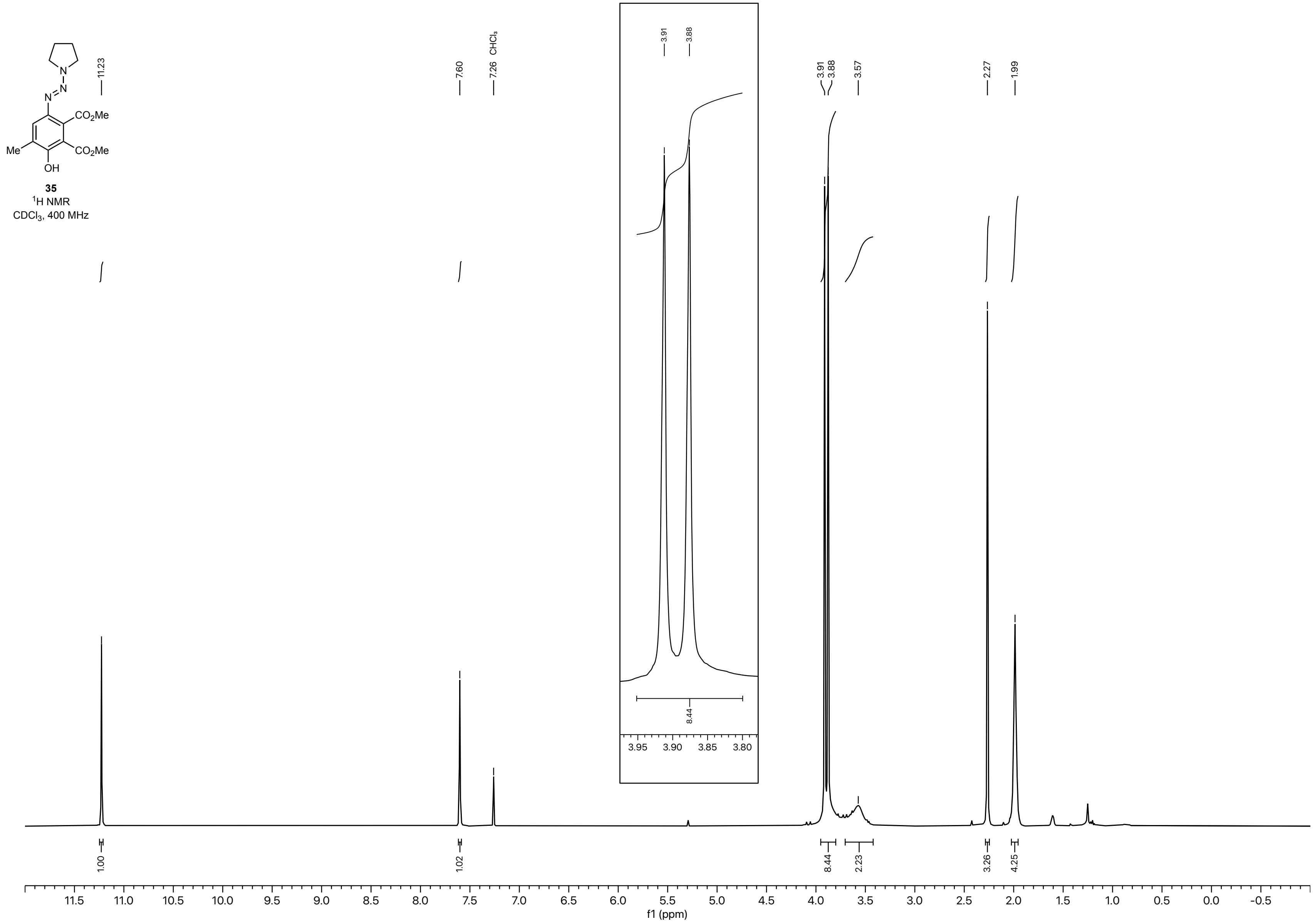
35
^{1}H NMR
CDCl$_3$, 400 MHz
11.23
7.60
7.26 CHCl$_3$
3.91
3.88
3.57
2.27
1.99
1.00
1.02
8.44
2.23
3.26
4.25
f1 (ppm)

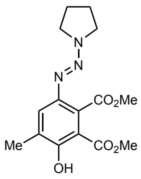

35
^{13}C NMR
$CDCl_3$, 100 MHz

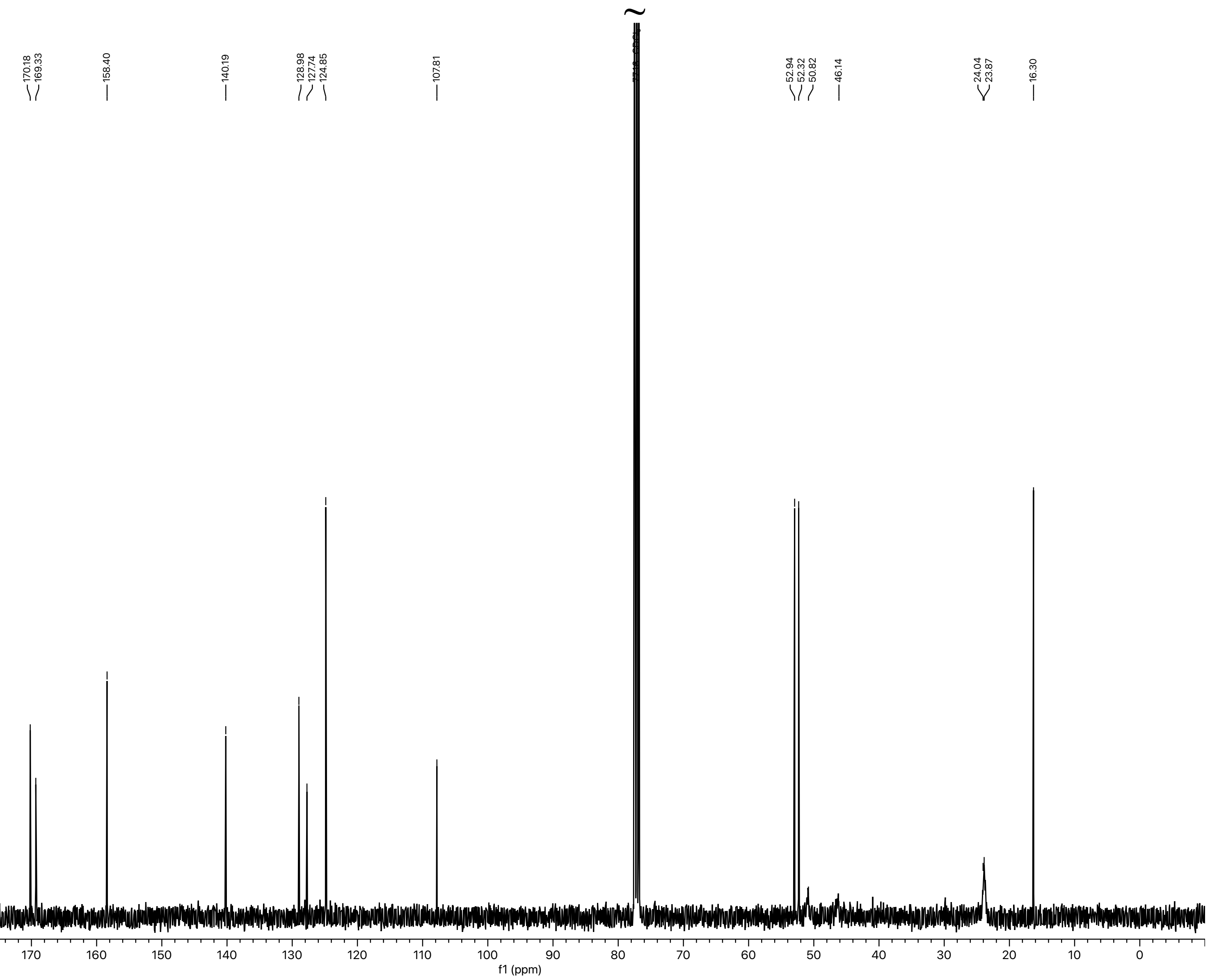

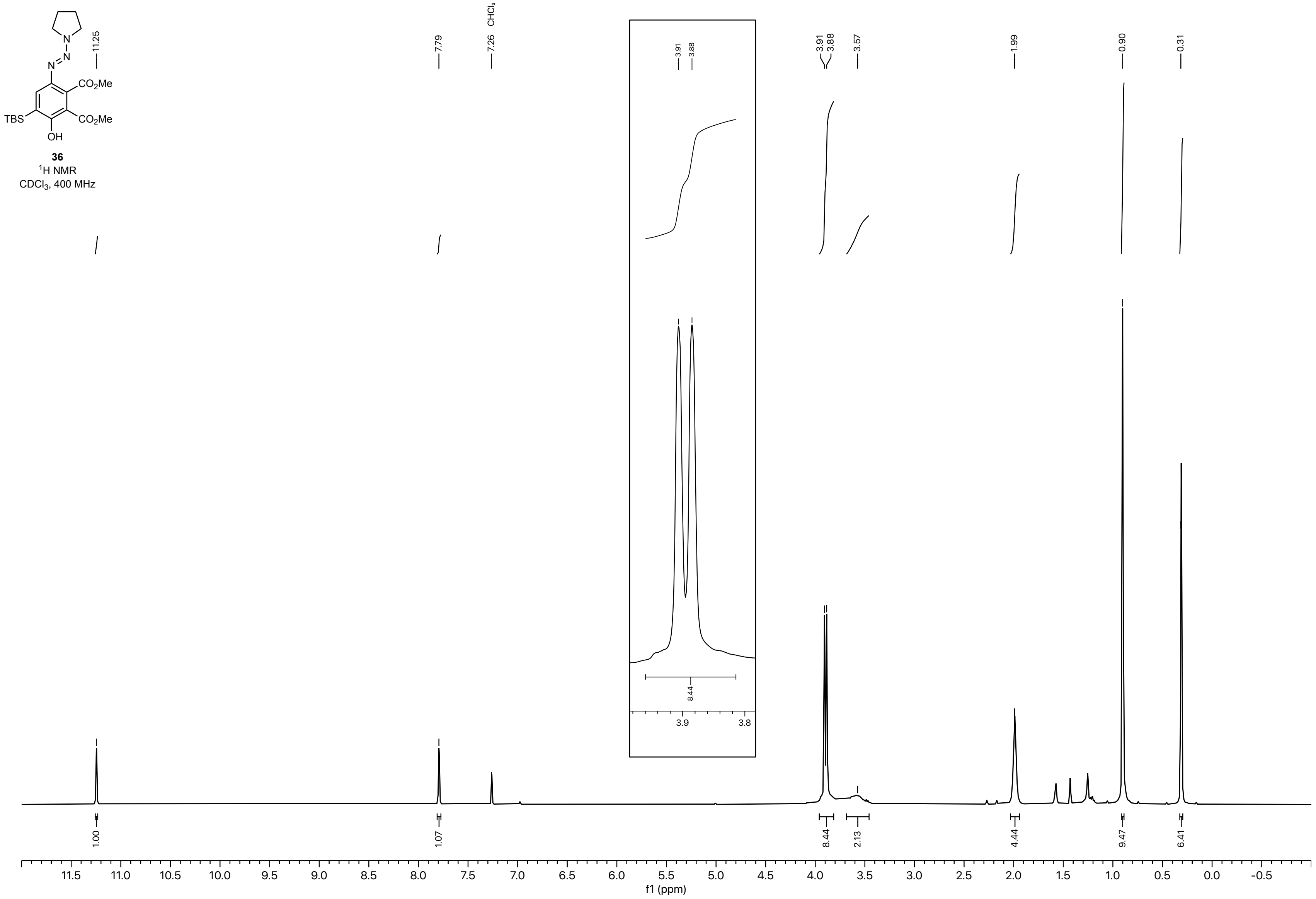
TBS
OH
N
N
N
CO_2Me
CO_2Me
36
^{1}H NMR
$CDCl_3$, 400 MHz
11.25
7.79
7.26 $CHCl_3$
3.91
3.88
3.57
1.99
0.90
0.31
8.44
1.00
1.07
2.13
4.44
9.47
6.41
3.9
3.8
f1 (ppm)

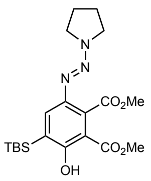

36

^{13}C NMR

$CDCl_3$, 100 MHz

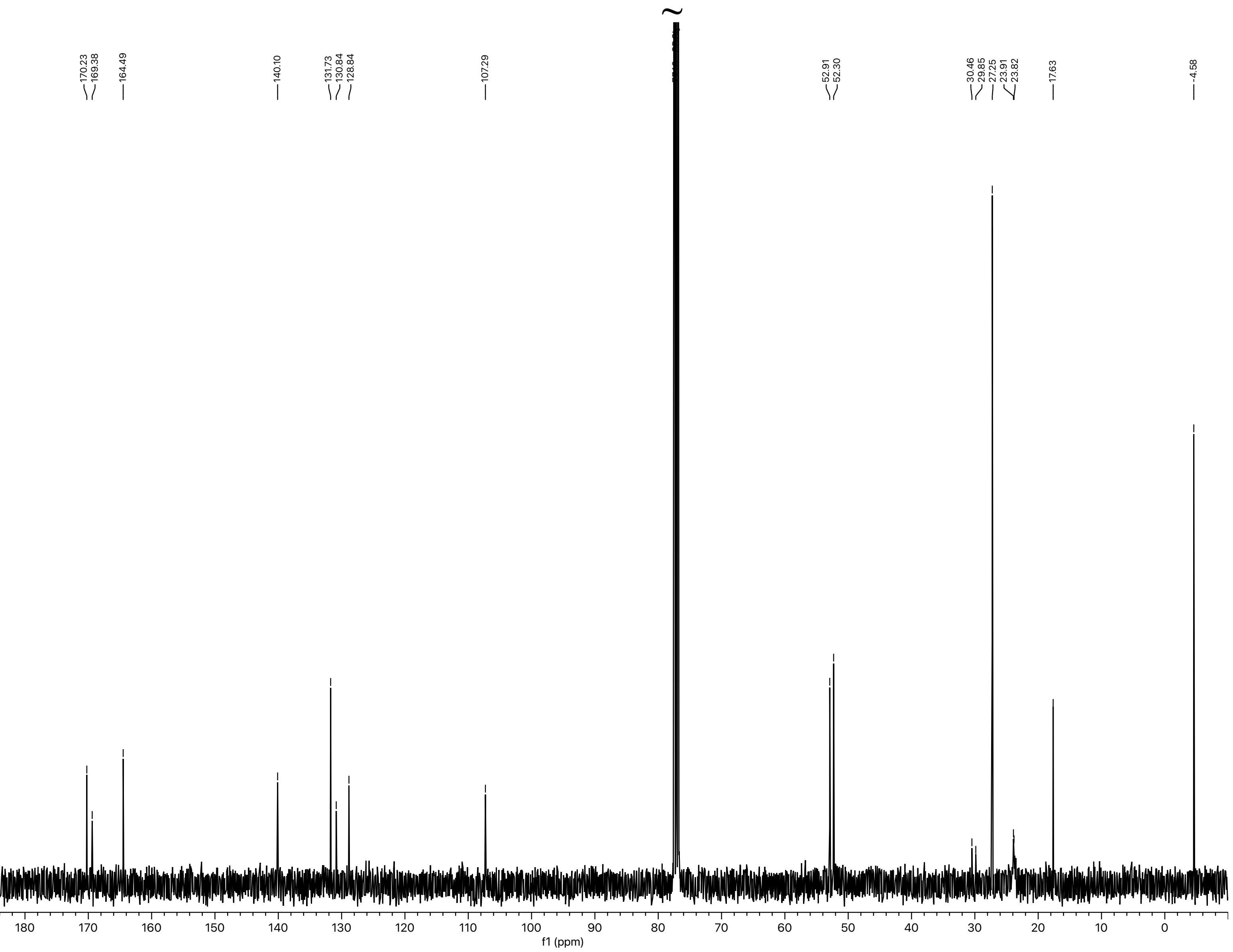

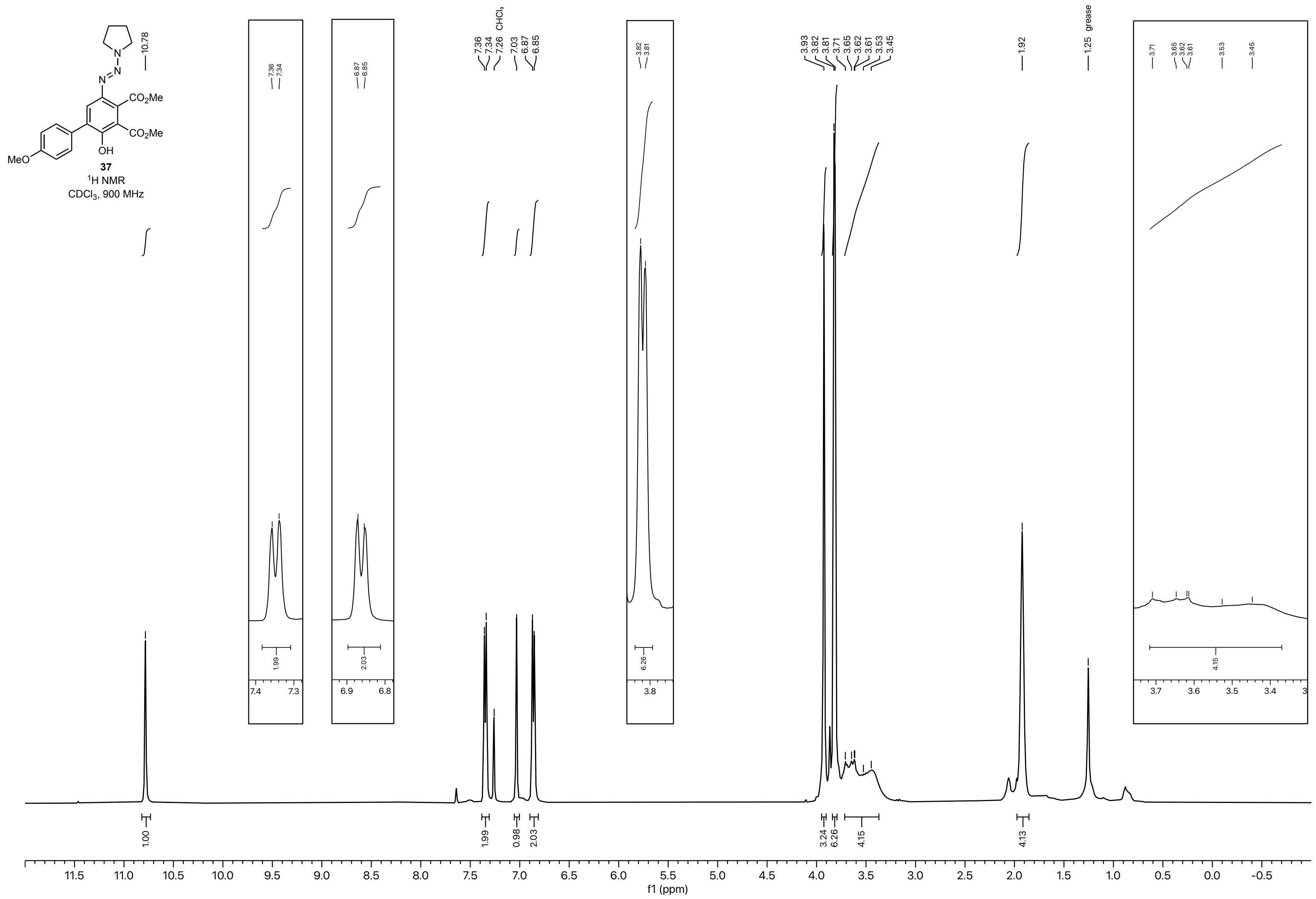

CO2Me
CO2Me
OH
MeO
37
1H NMR
CDCl3, 900 MHz
10.78
7.36 7.34 7.26 CHCl3 7.03 6.87 6.85
3.93 3.82 3.81 3.71 3.65 3.62 3.61 3.53 3.45
1.92
1.25 grease
1.00
1.99
0.98
2.03
3.24
6.26
4.15
4.13
f1 (ppm)

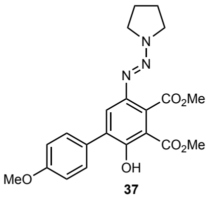

37
^{13}C NMR
$CDCl_3$, 100 MHz

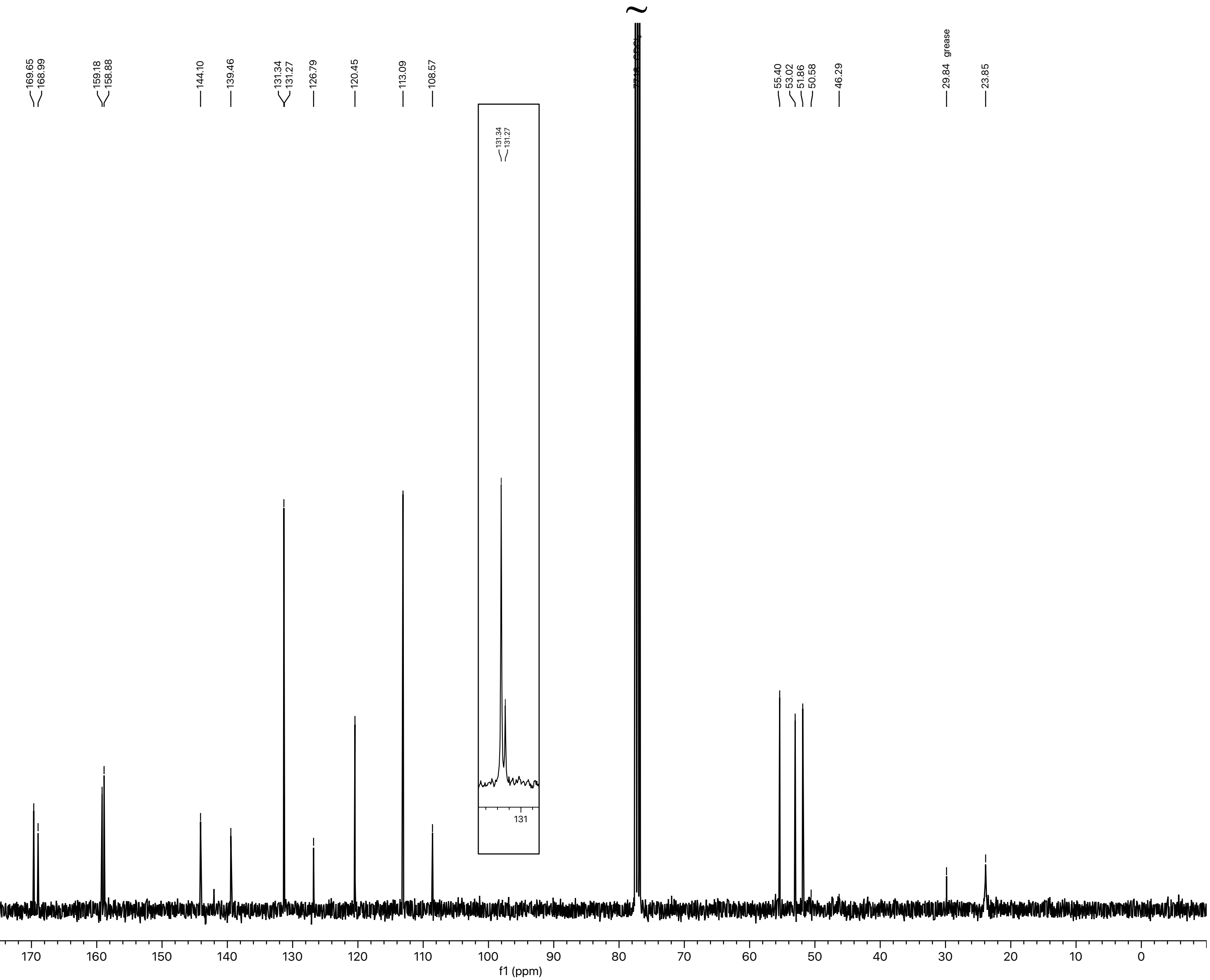

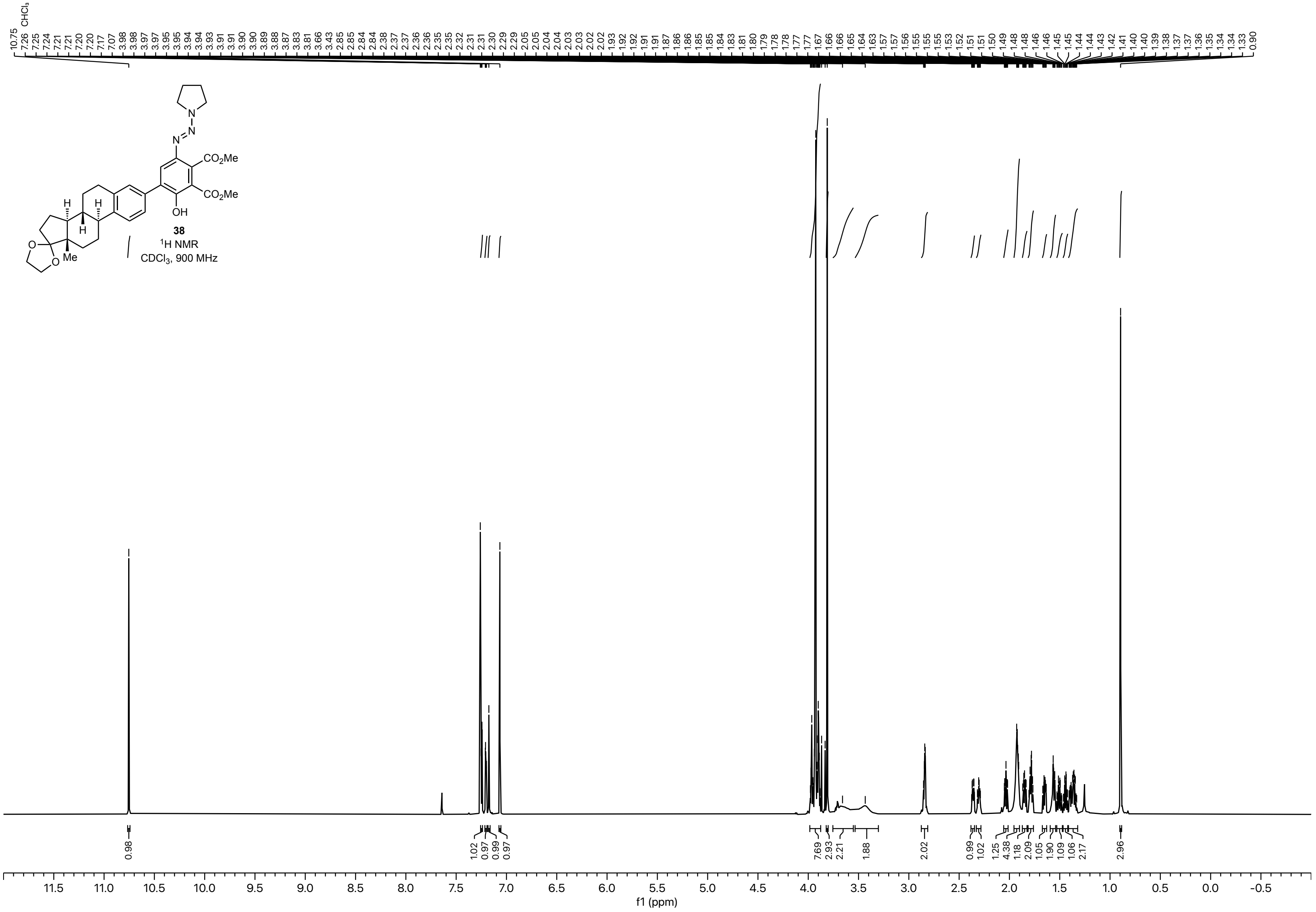

N
N=N
CO2Me
CO2Me
OH
H
H
H
O
O
Me
38
1H NMR
CDCl3, 900 MHz
f1 (ppm)

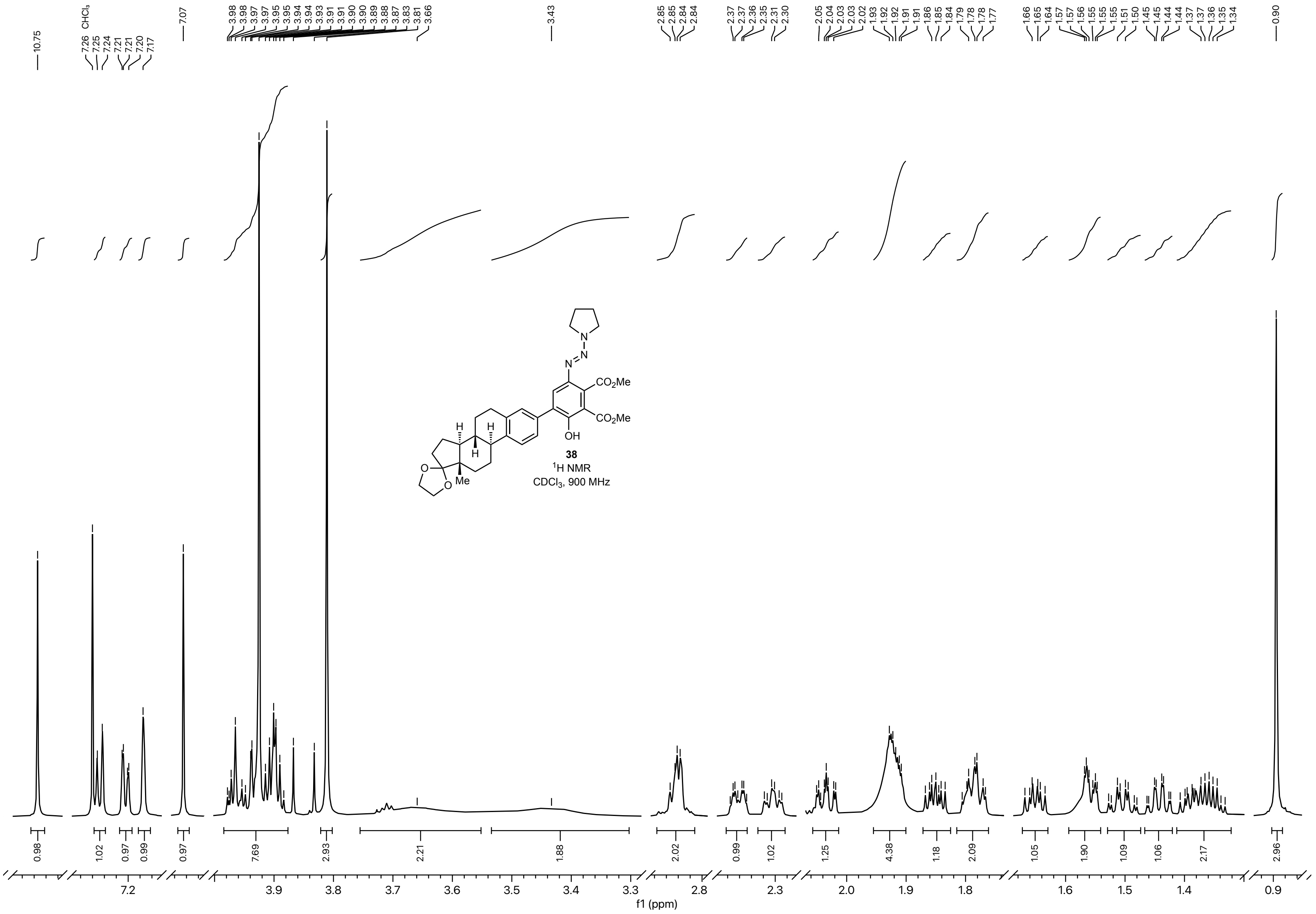
38
^{1}H NMR
$CDCl_3$, 900 MHz
f1 (ppm)

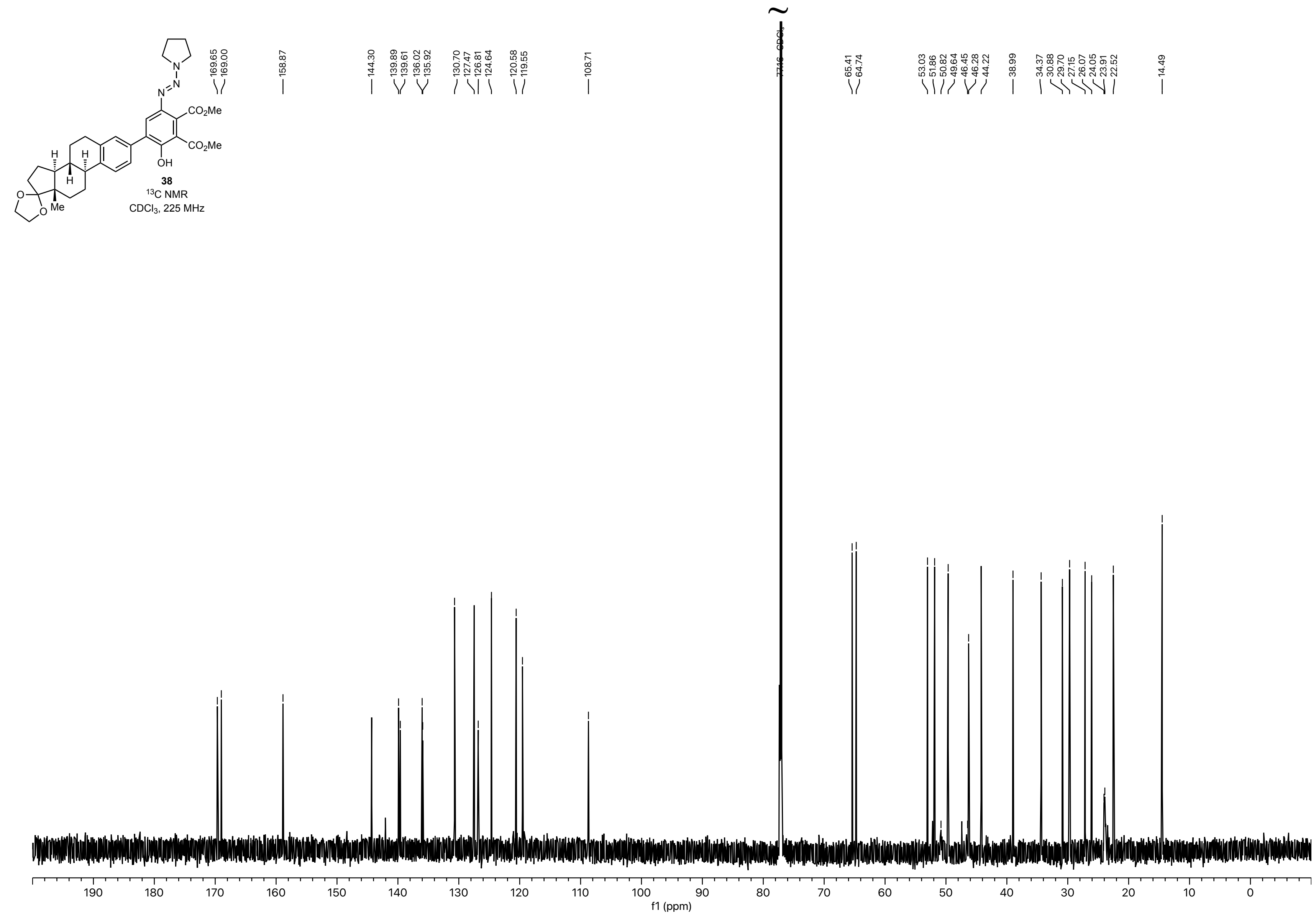
38
^{13}C NMR
$CDCl_3$, 225 MHz
CO_2Me
CO_2Me
OH
Me
169.65
169.00
158.87
144.30
139.89
139.61
136.02
135.92
130.70
127.47
126.81
124.64
120.58
119.55
108.71
77.16 $CDCl_3$
65.41
64.74
53.03
51.86
50.82
49.64
46.45
46.28
44.22
38.99
34.37
30.88
29.70
27.15
26.07
24.05
23.91
22.52
14.49
f1 (ppm)

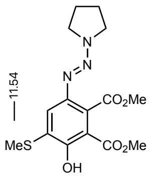

39
^{1}H NMR
$CDCl_3$, 900 MHz

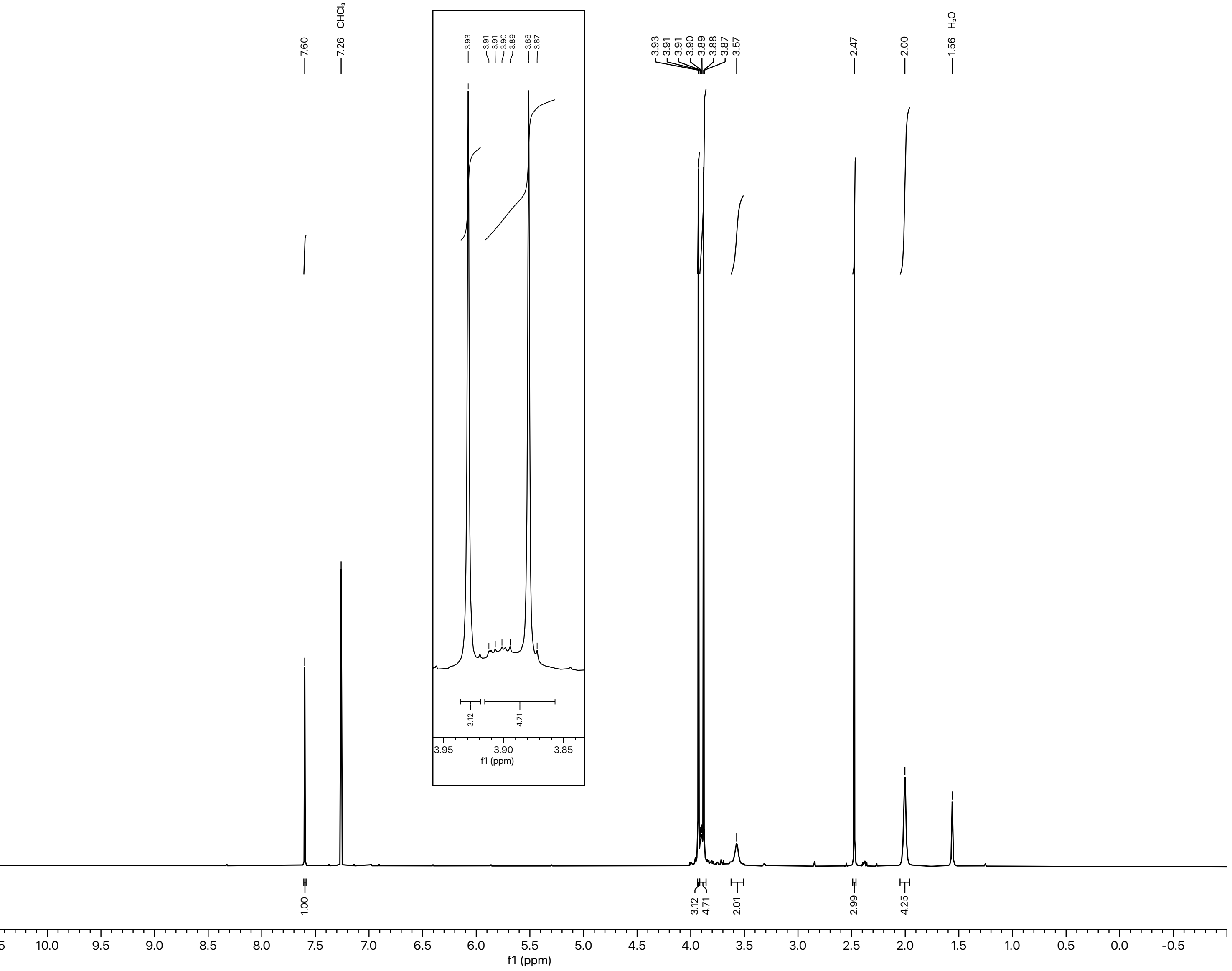

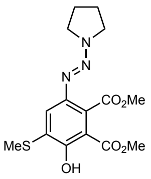

39
^{13}C NMR
$CDCl_3$, 225 MHz

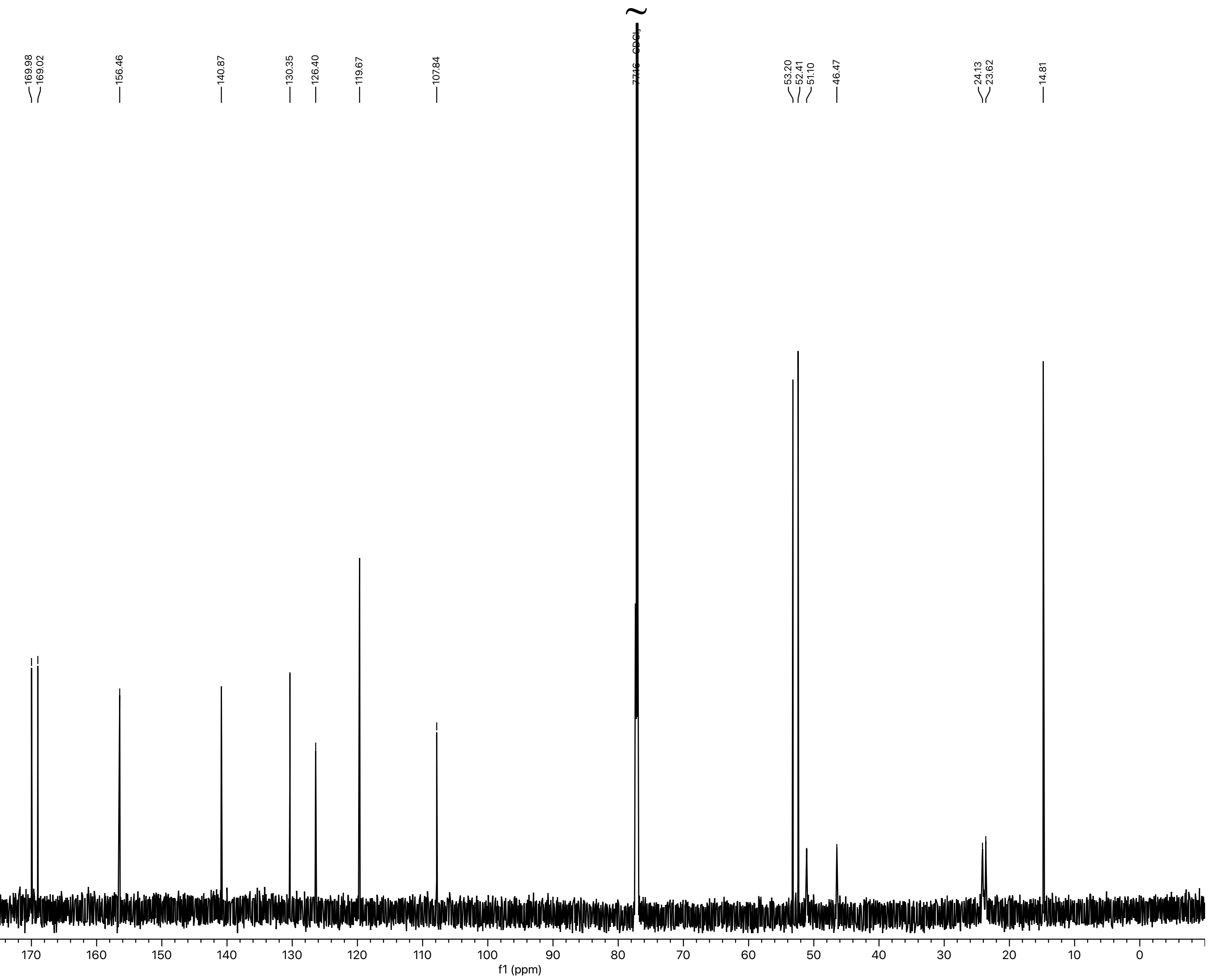

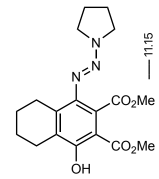

40
^1H NMR
$CDCl_3$, 400 MHz

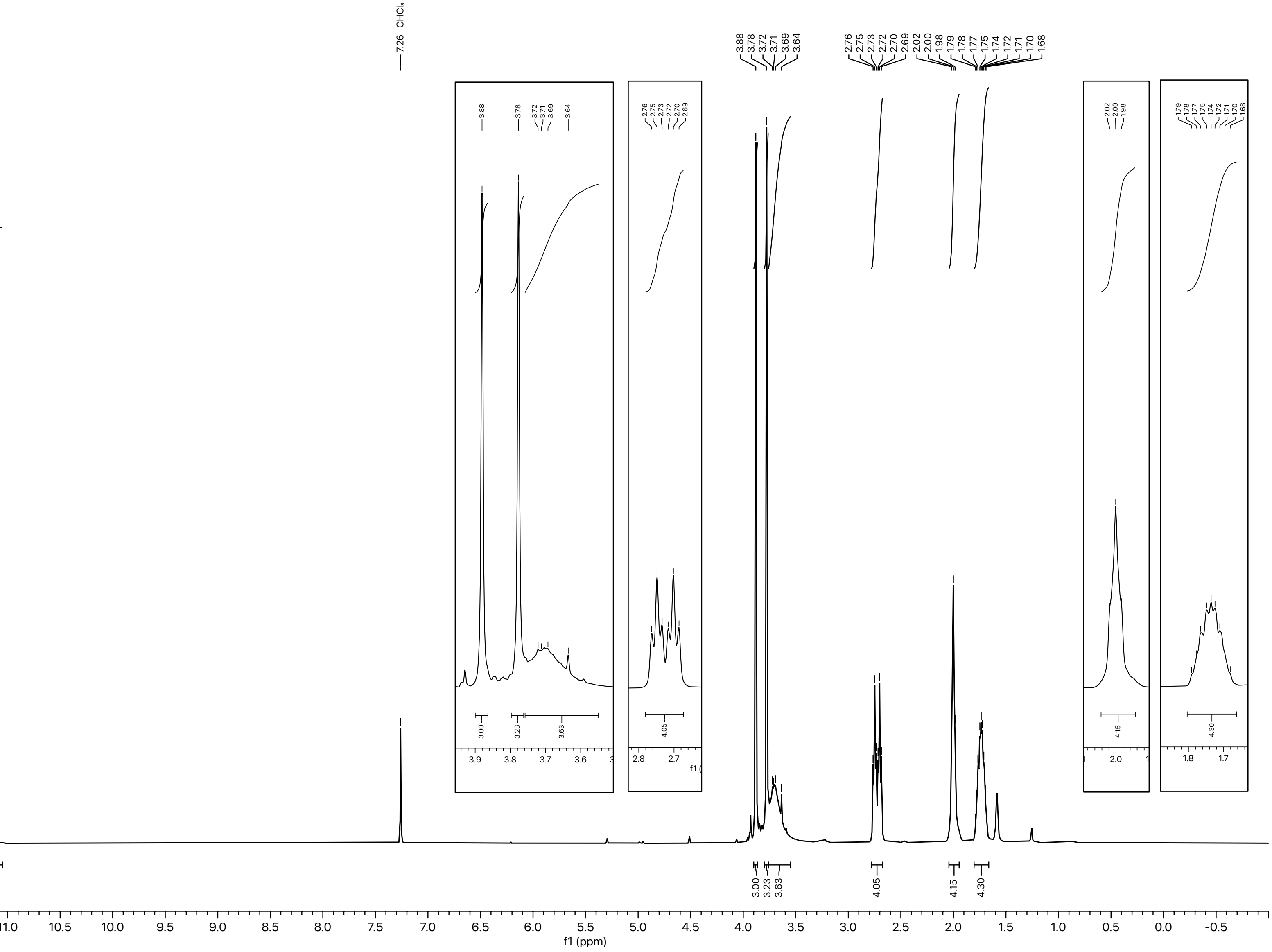

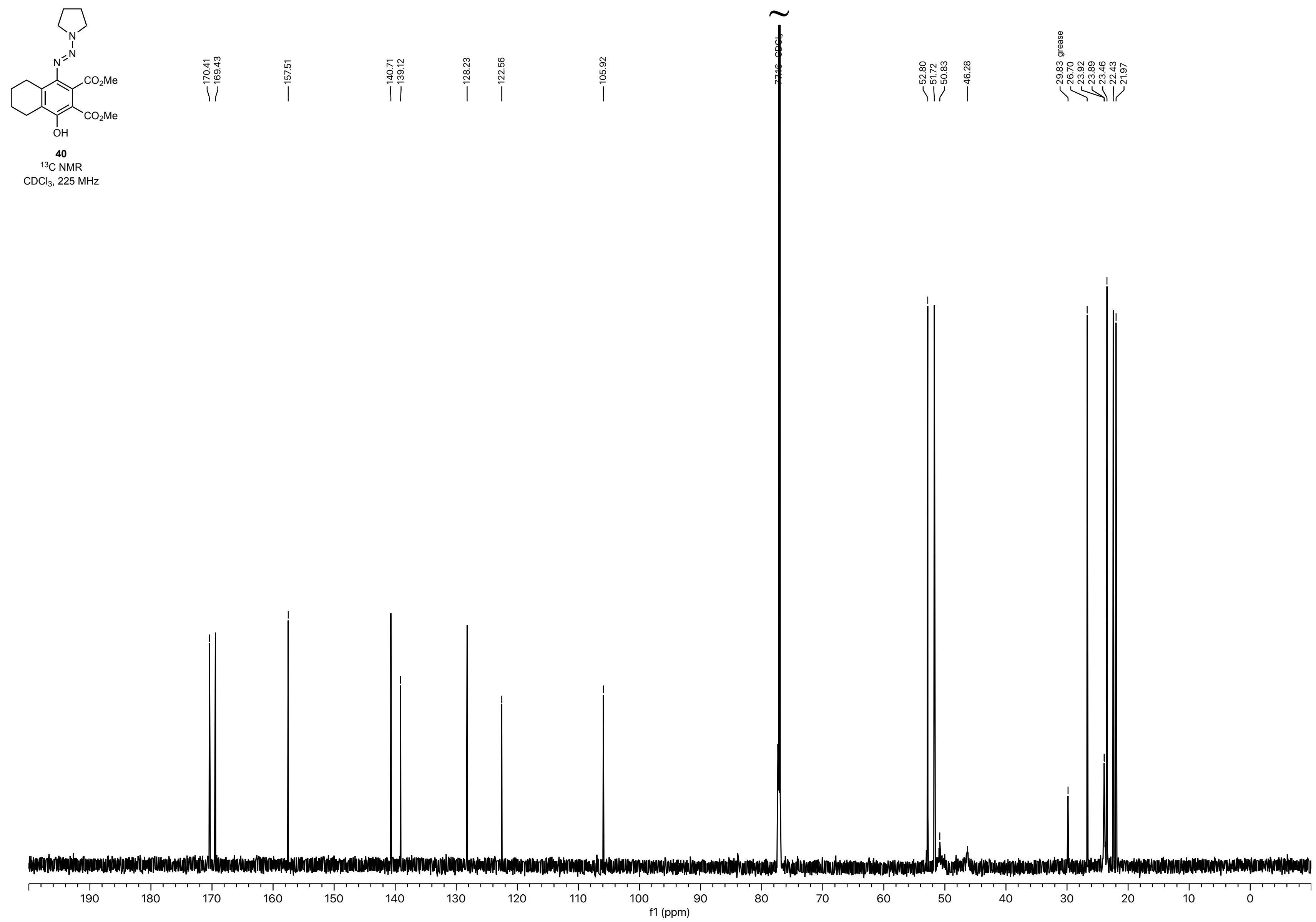

40
^{13}C NMR
$CDCl_3$, 225 MHz

Allenes as Dienophiles

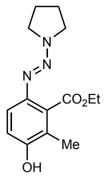

42
^{1}H NMR
$CDCl_3$, 400 MHz

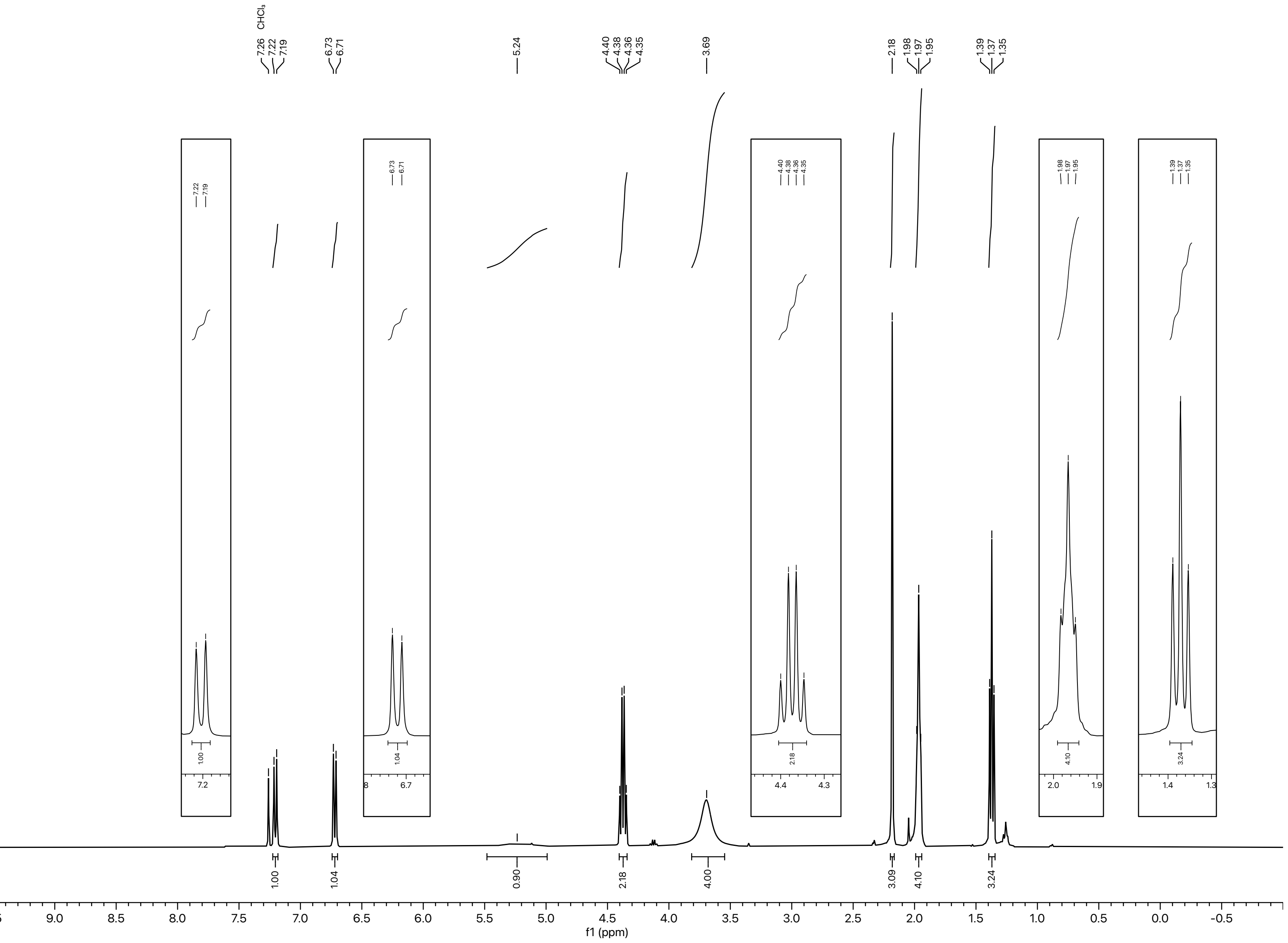

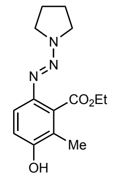

42
^{13}C NMR
$CDCl_3$, 100 MHz

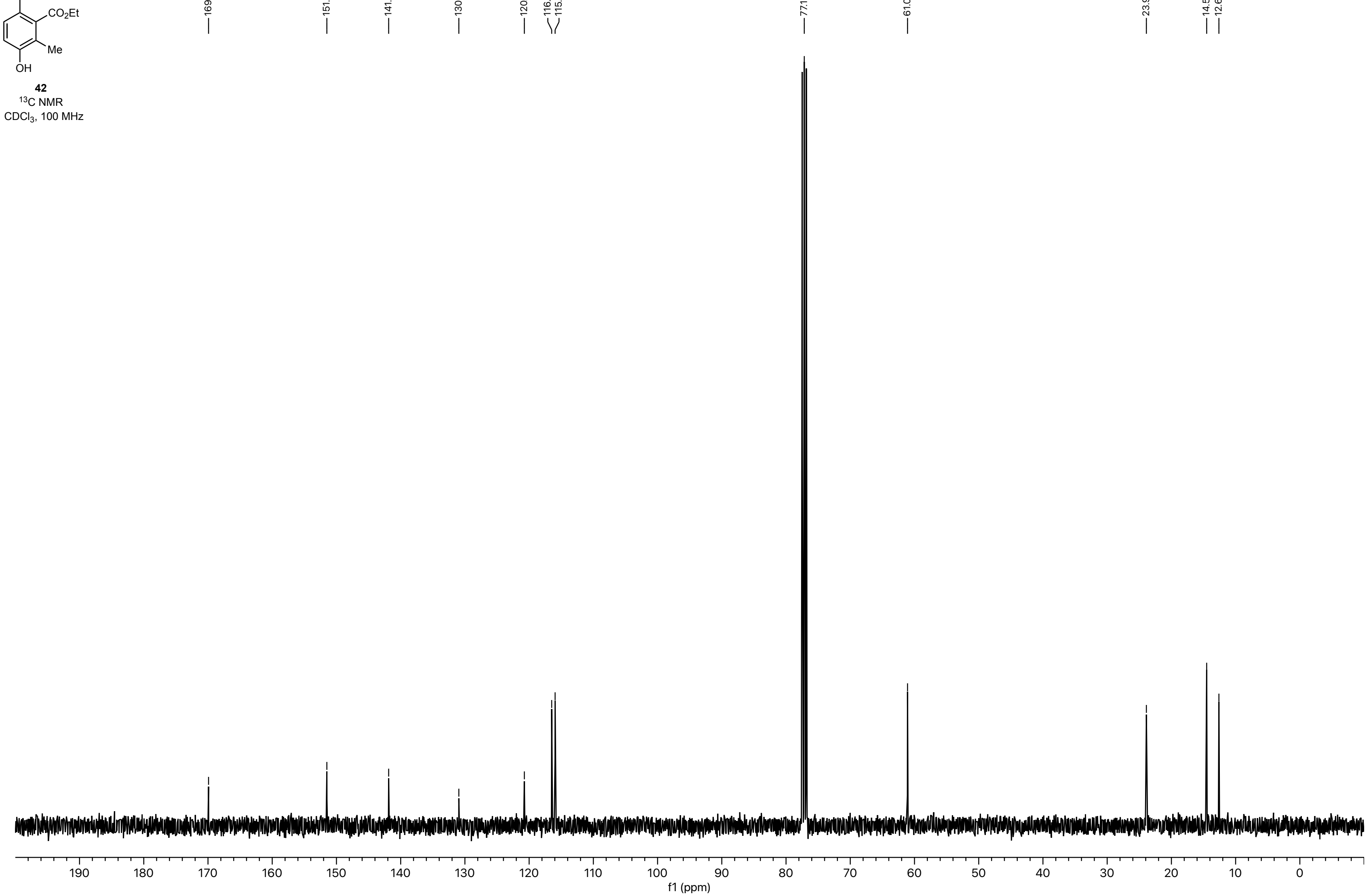

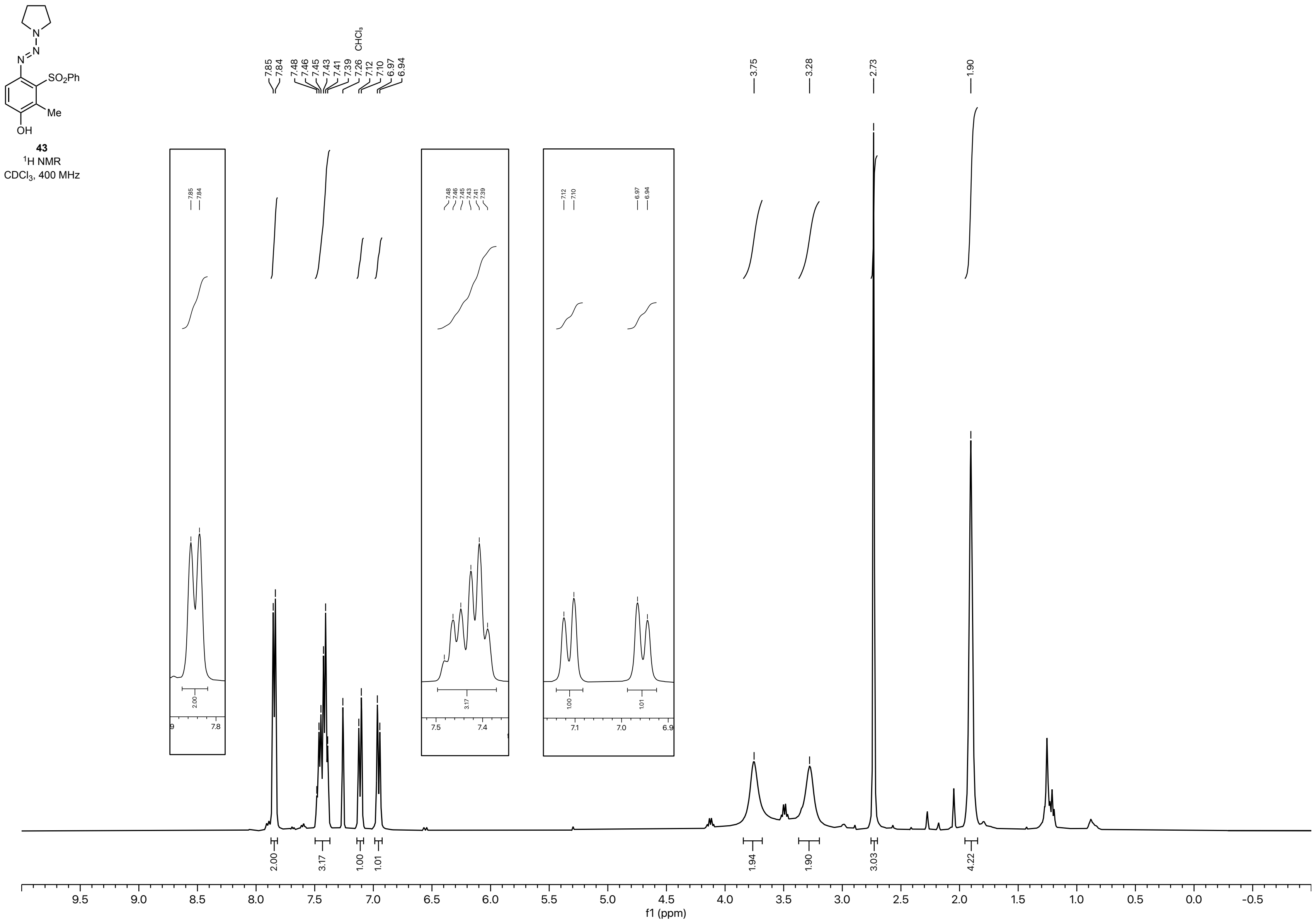
SO2Ph
Me
OH
43
1H NMR
CDCl3, 400 MHz
7.85
7.84
7.48
7.46
7.45
7.43
7.41
7.39
CHCl3
7.26
7.12
7.10
6.97
6.94
3.75
3.28
2.73
1.90
2.00
3.17
1.00
1.01
1.94
1.90
3.03
4.22
f1 (ppm)

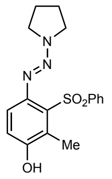

43
^{13}C NMR
$CDCl_3$, 100 MHz

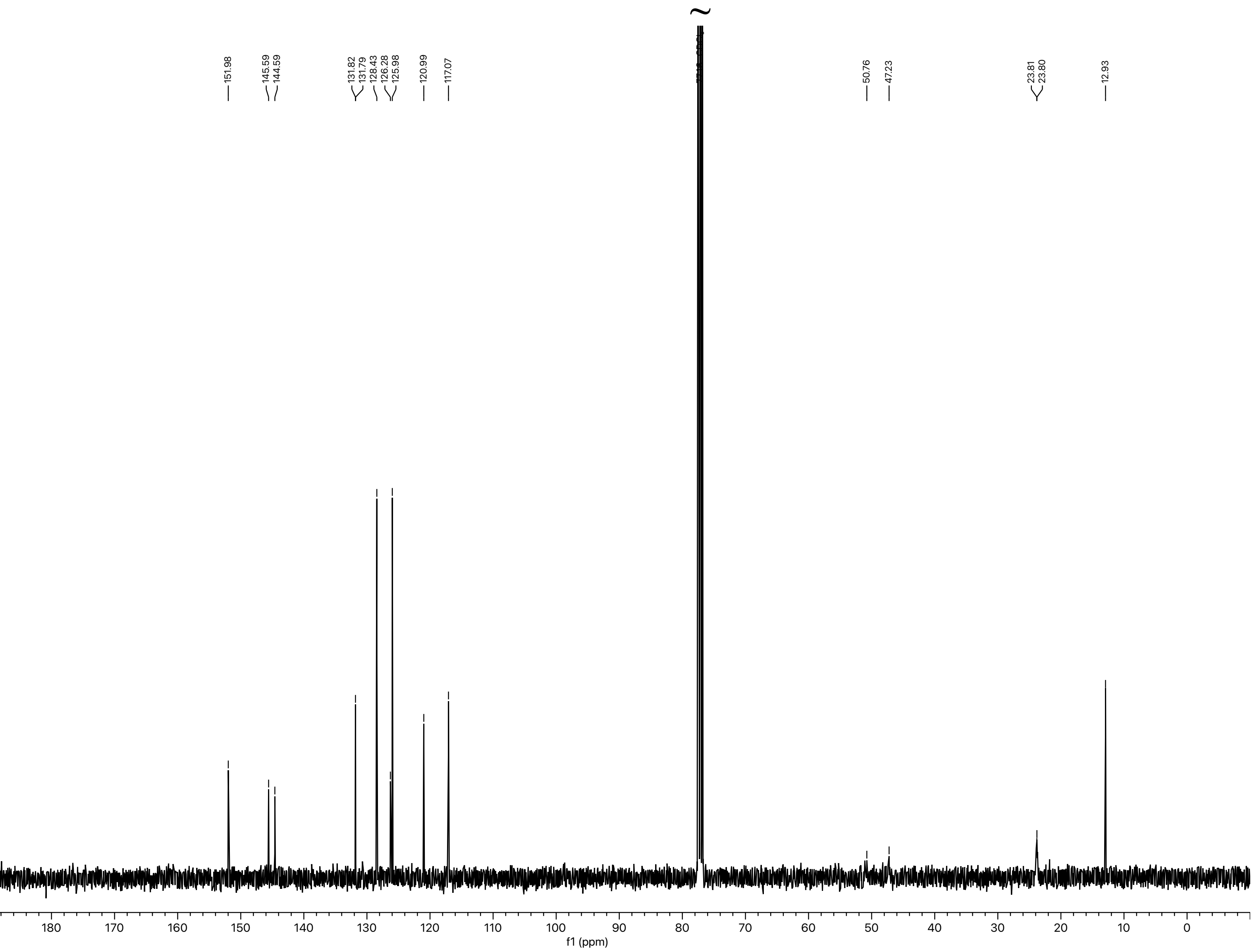

Tethered Dienophiles

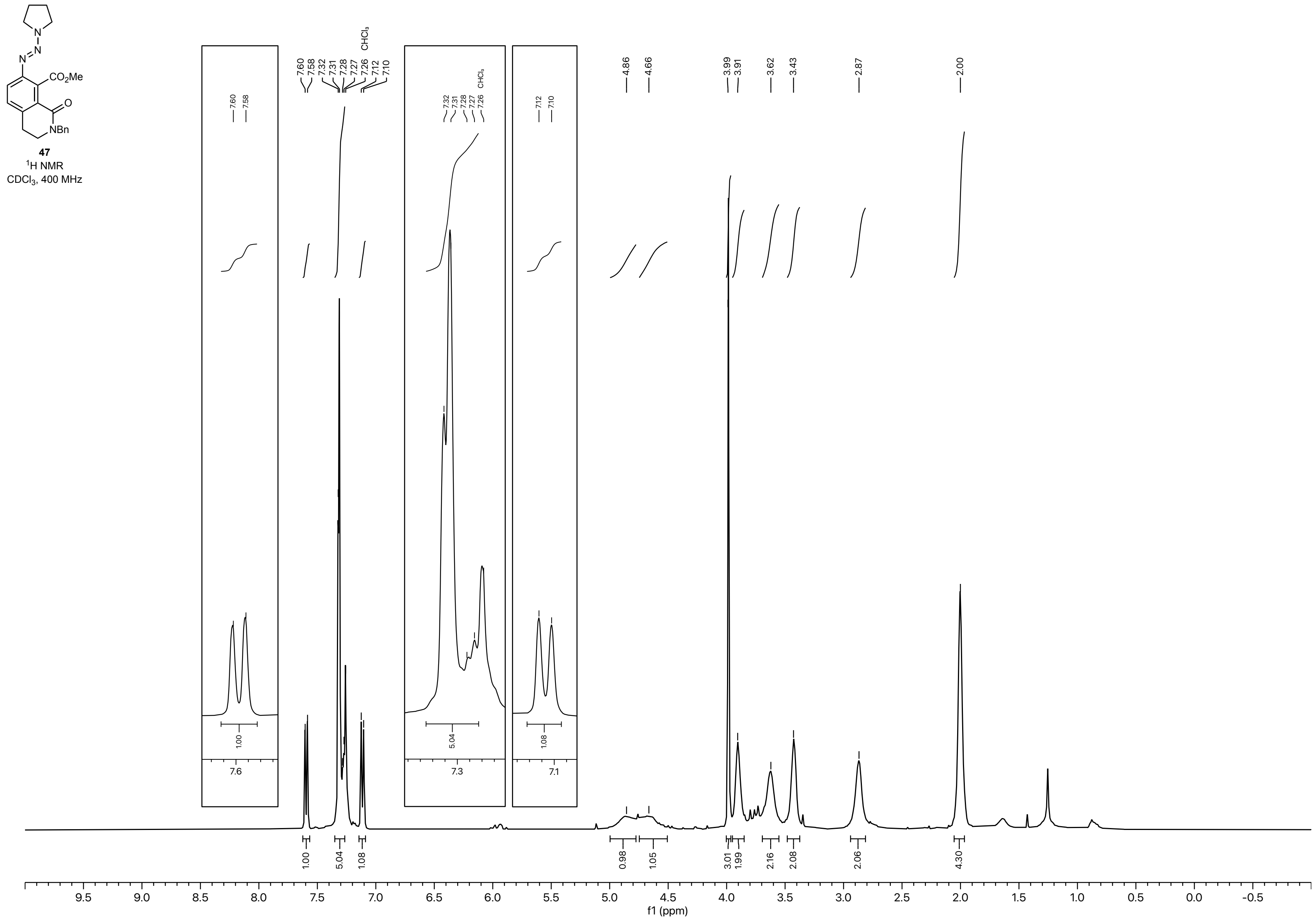

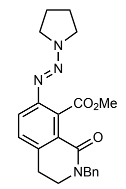

47
^{13}C NMR
$CDCl_3$, 100 MHz

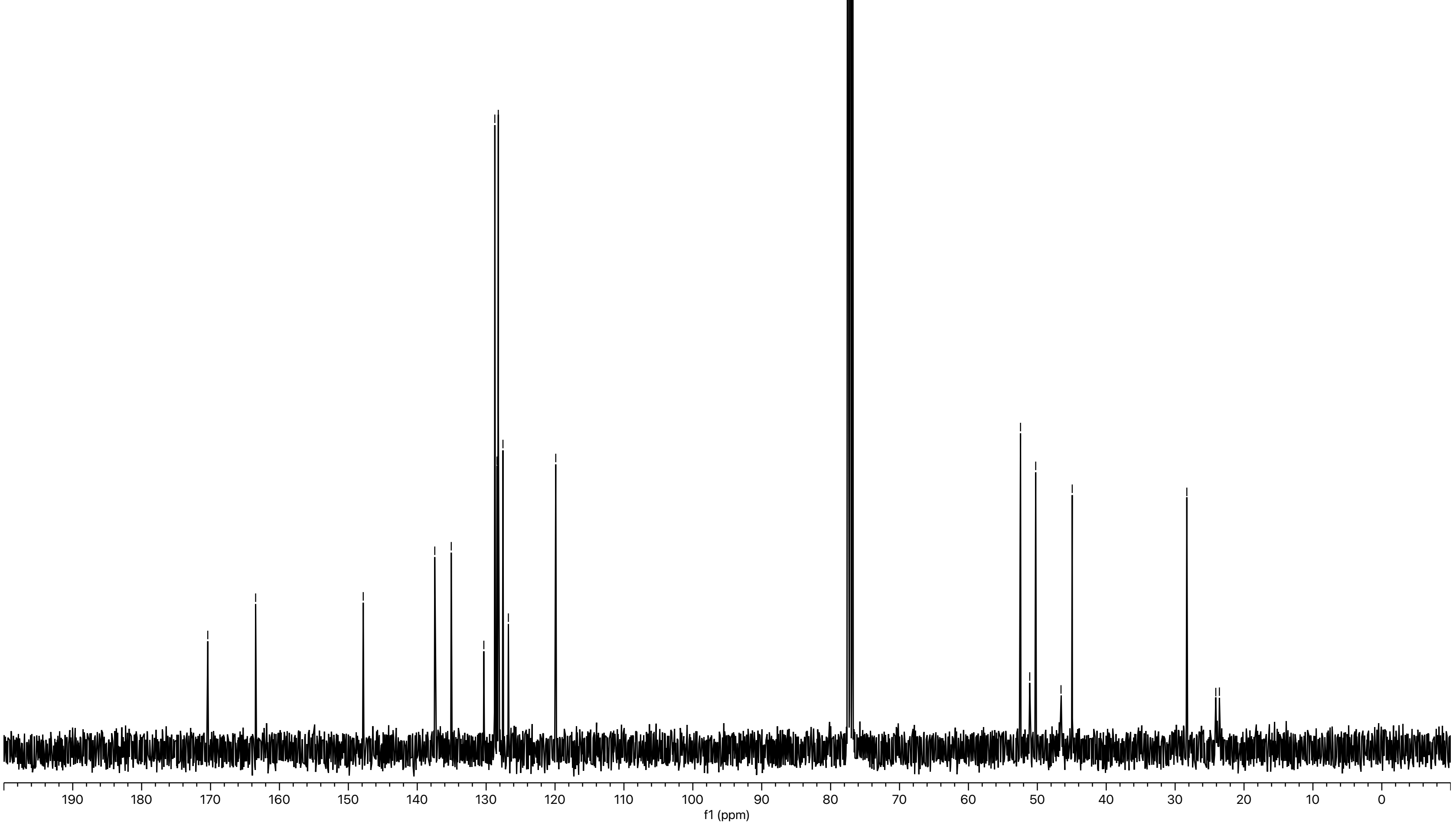

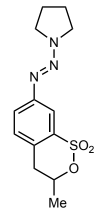

51
^{1}H NMR
$CDCl_3$, 600 MHz

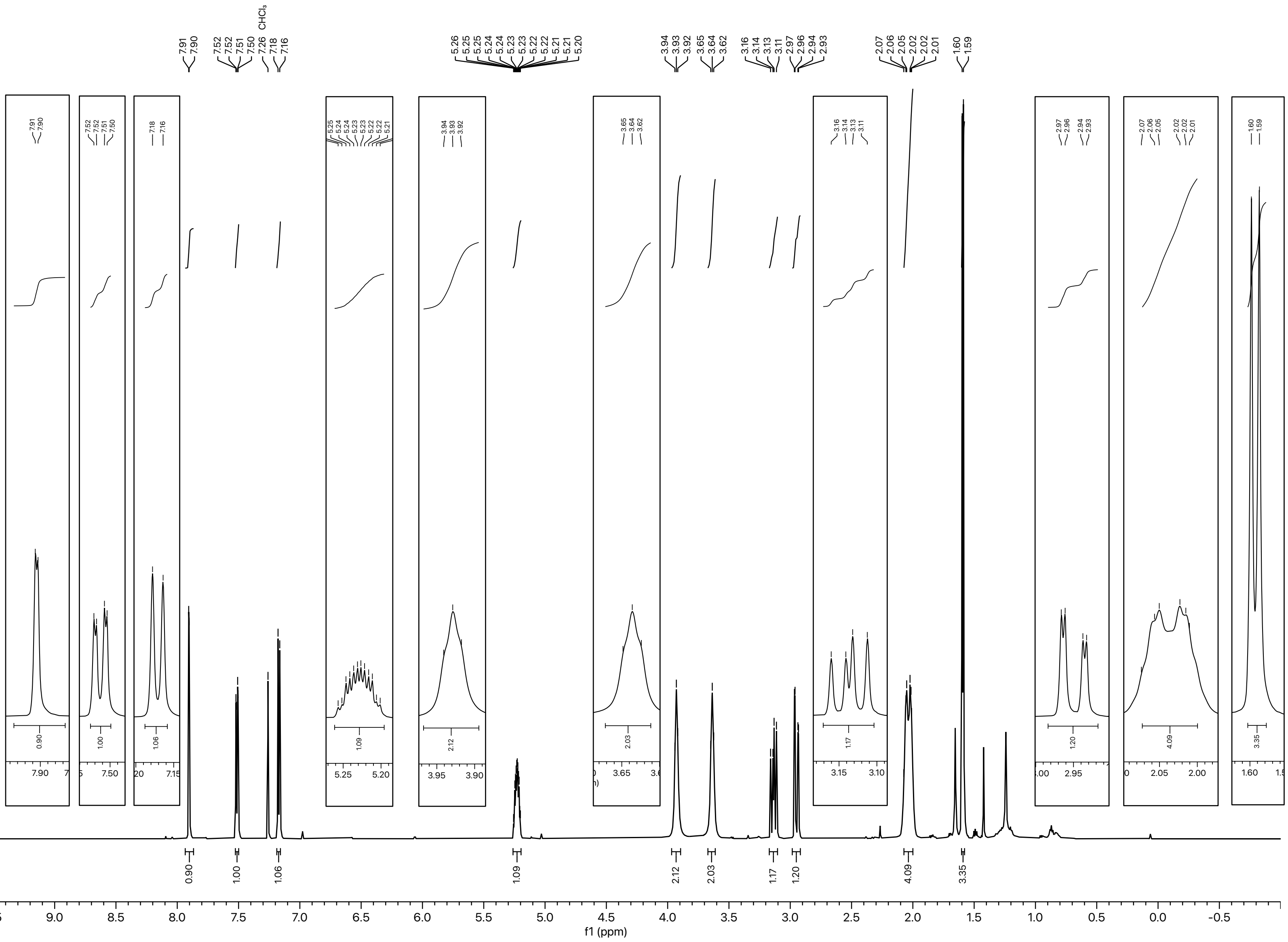

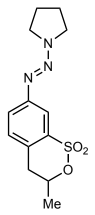

51
^{13}C NMR
$CDCl_3$, 100 MHz

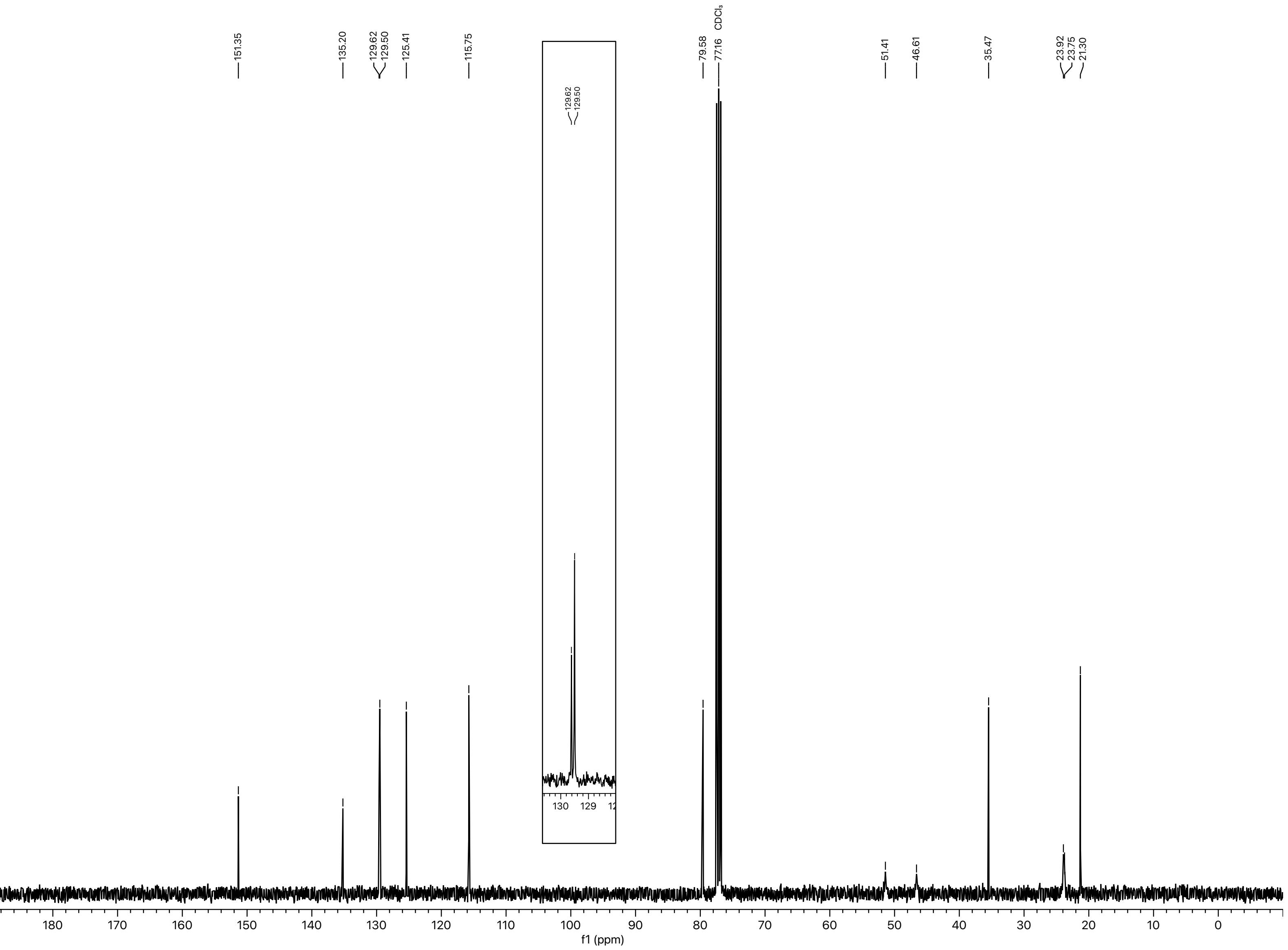

Benzyne as Dienophile

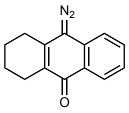

53
^{1}H NMR
$CDCl_3$, 600 MHz

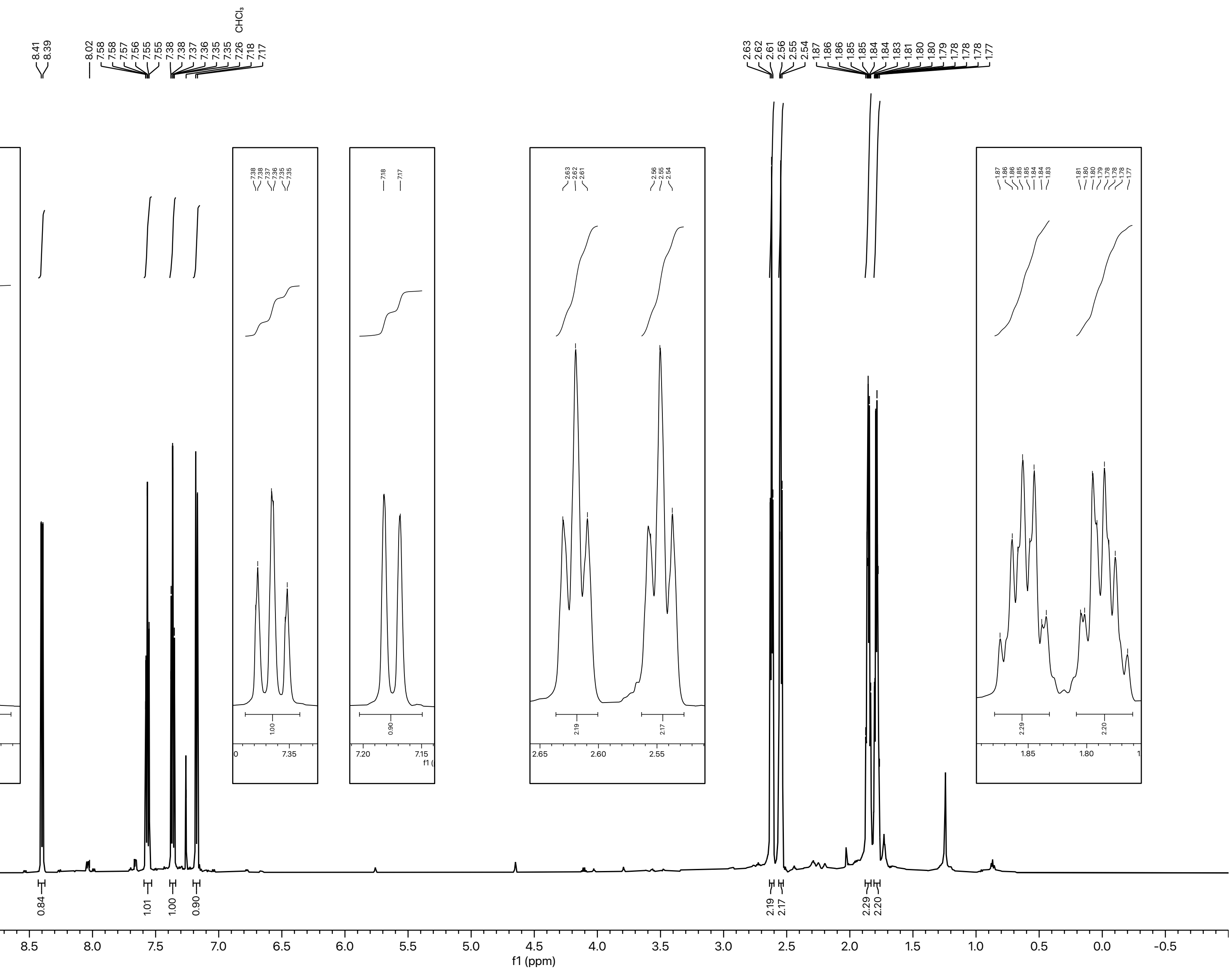

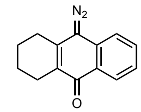

53

^{13}C NMR

$CDCl_3$, 150 MHz

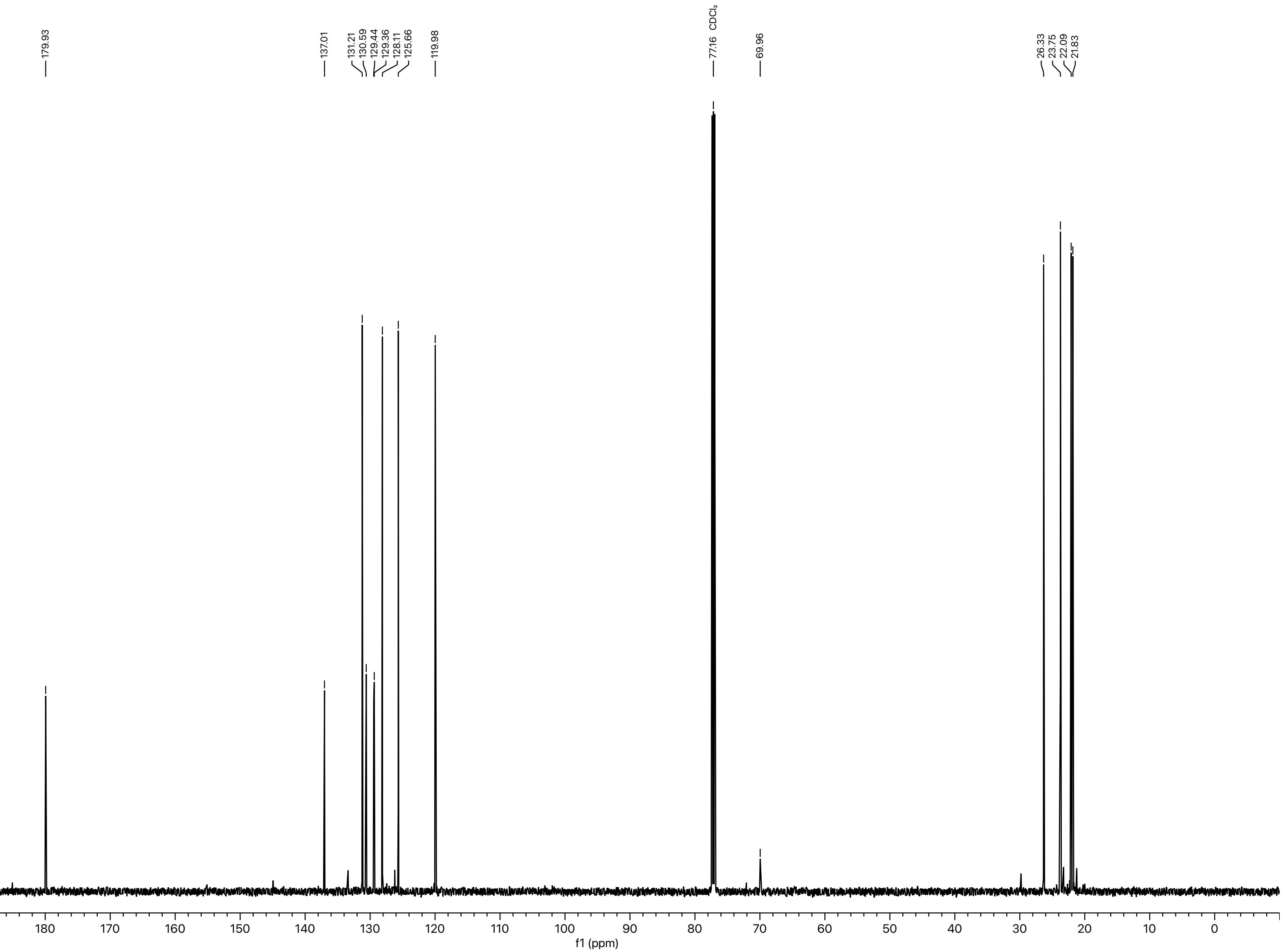

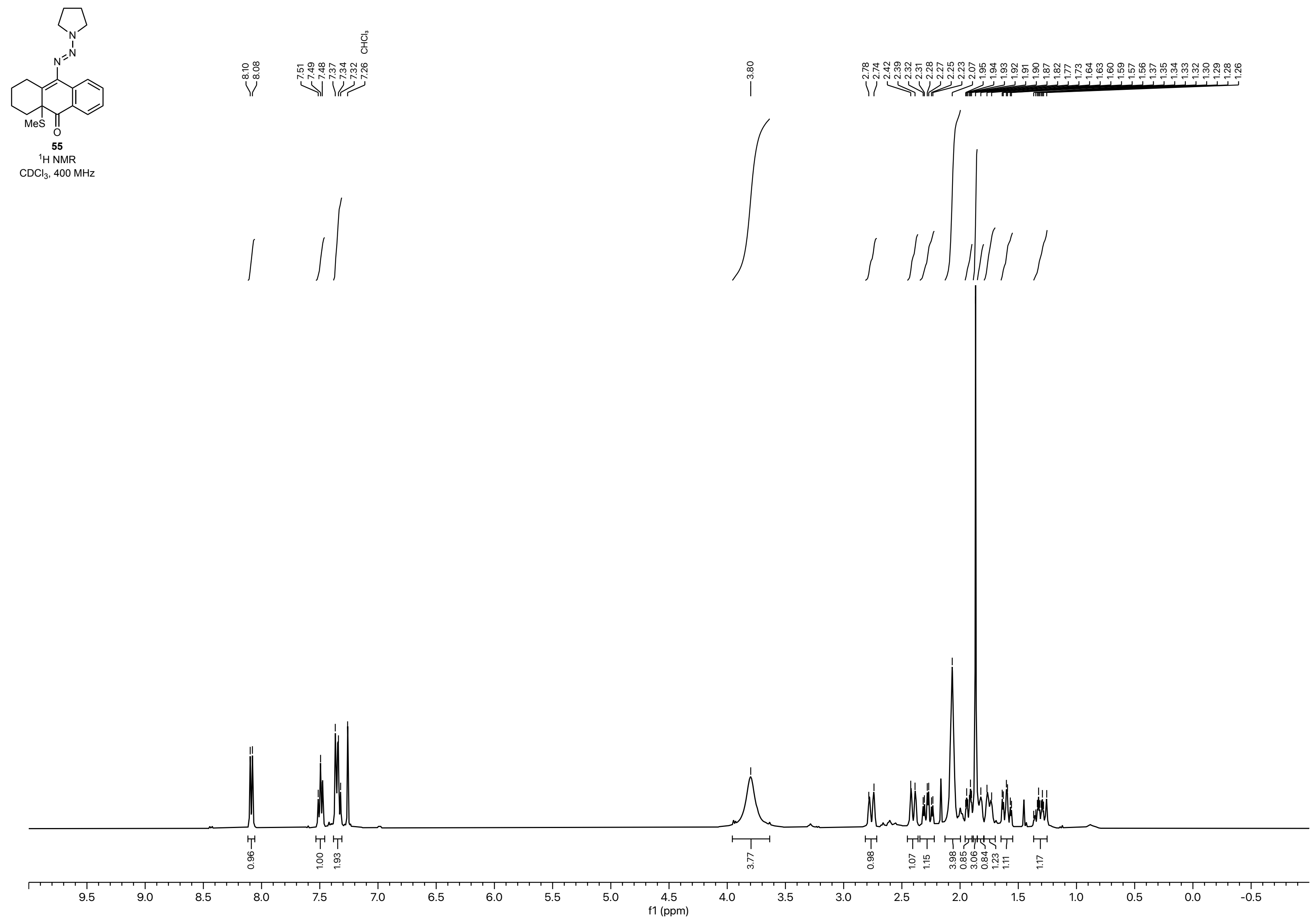

N
N
N
MeS
O
55
^{1}H NMR
$CDCl_3$, 400 MHz
f1 (ppm)

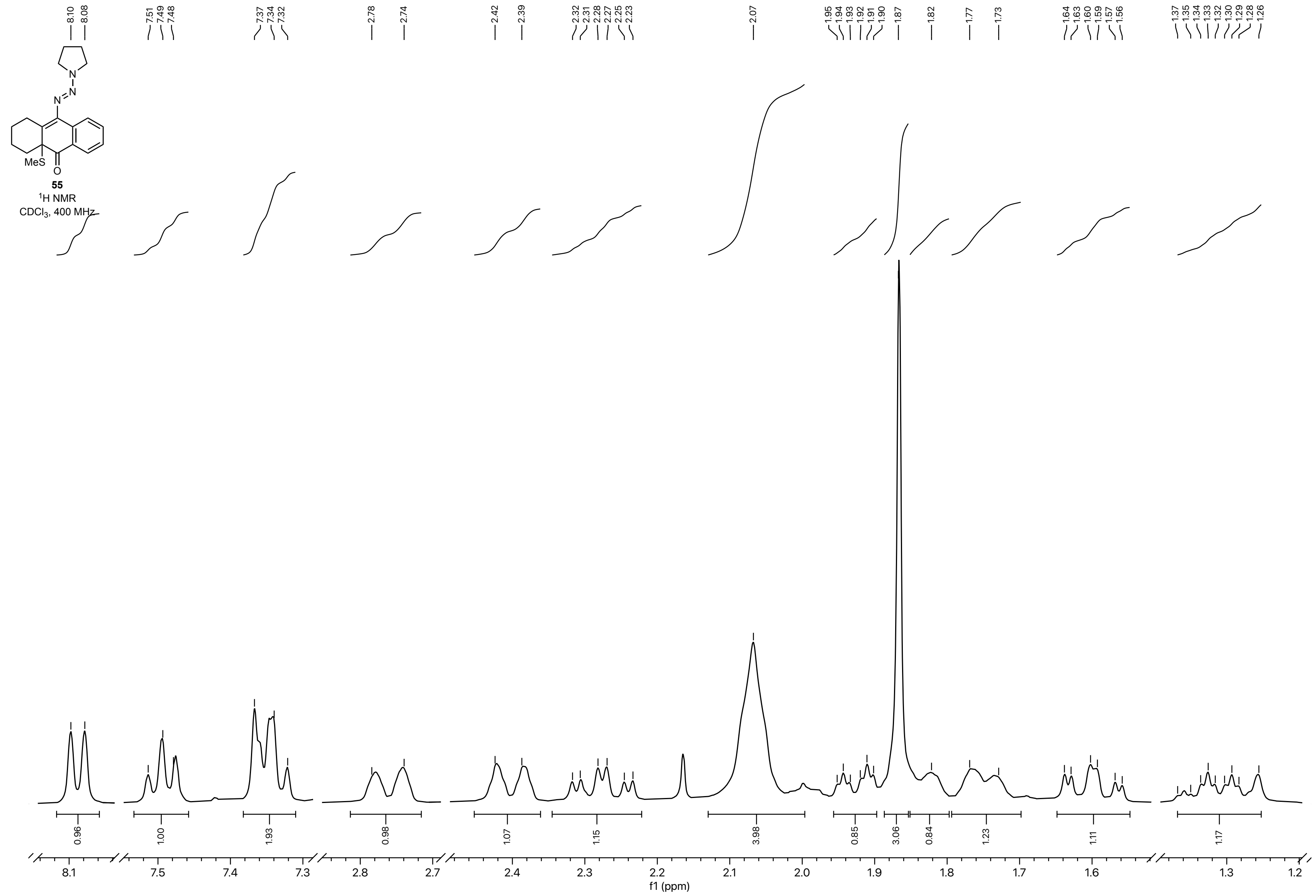

MeS
O
N
N
N
55
^{1}H NMR
$CDCl_3$, 400 MHz
8.10
8.08
7.51
7.49
7.48
7.37
7.34
7.32
2.78
2.74
2.42
2.39
2.32
2.31
2.28
2.27
2.25
2.23
2.07
1.95
1.94
1.93
1.92
1.91
1.90
1.87
1.82
1.77
1.73
1.64
1.63
1.60
1.59
1.57
1.56
1.37
1.35
1.34
1.33
1.32
1.30
1.29
1.28
1.26
0.96
1.00
1.93
0.98
1.07
1.15
3.98
0.85
3.06
0.84
1.23
1.11
1.17
8.1
7.5
7.4
7.3
2.8
2.7
2.4
2.3
2.2
2.1
2.0
1.9
1.8
1.7
1.6
1.3
1.2
f1 (ppm)

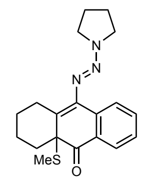

55

^{13}C NMR

$CDCl_3$, 100 MHz

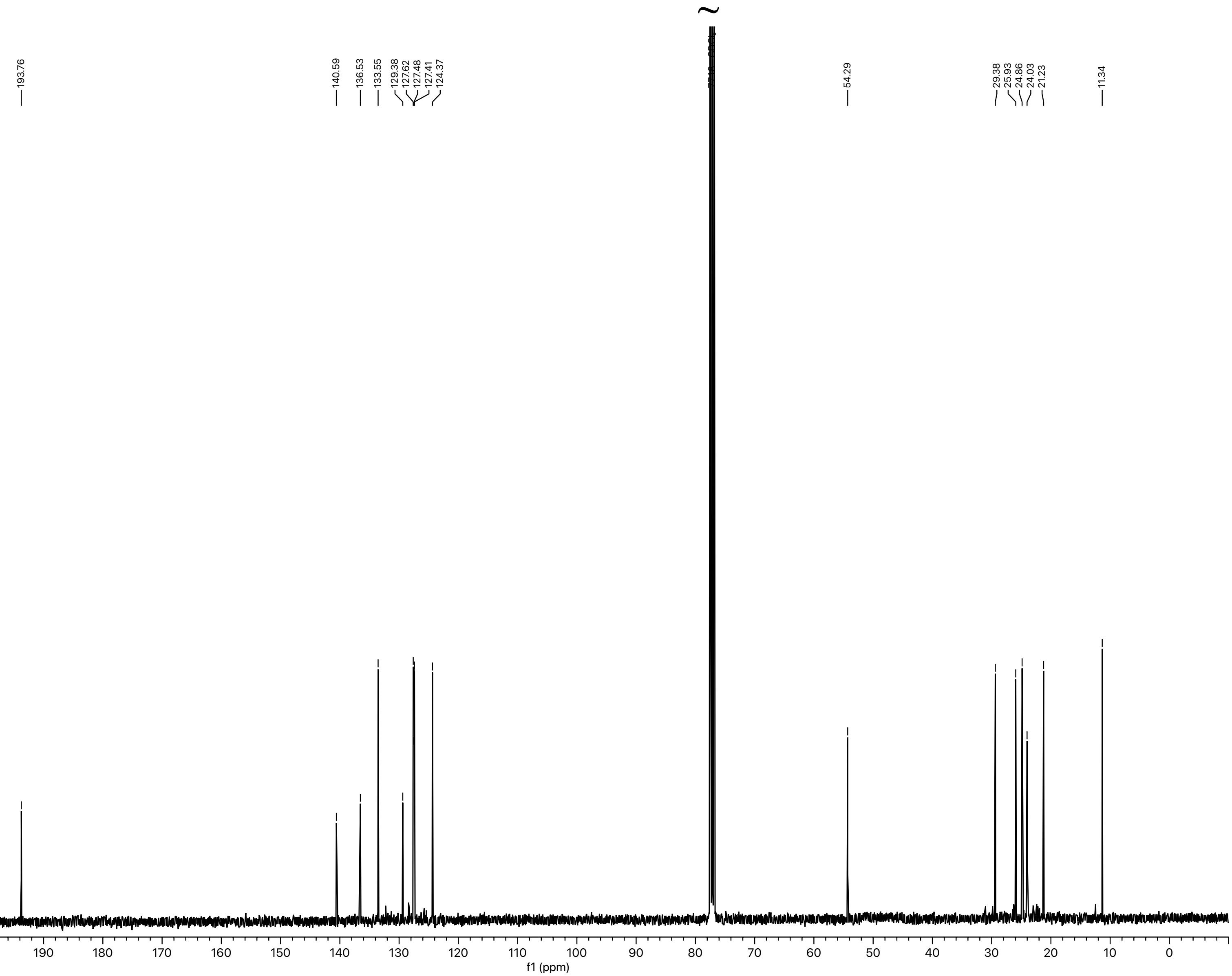

Derivatization Experiments

From Cycloadduct 29

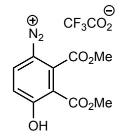

56
^{1}H NMR
$(CD_3)_2SO$, 400 MHz

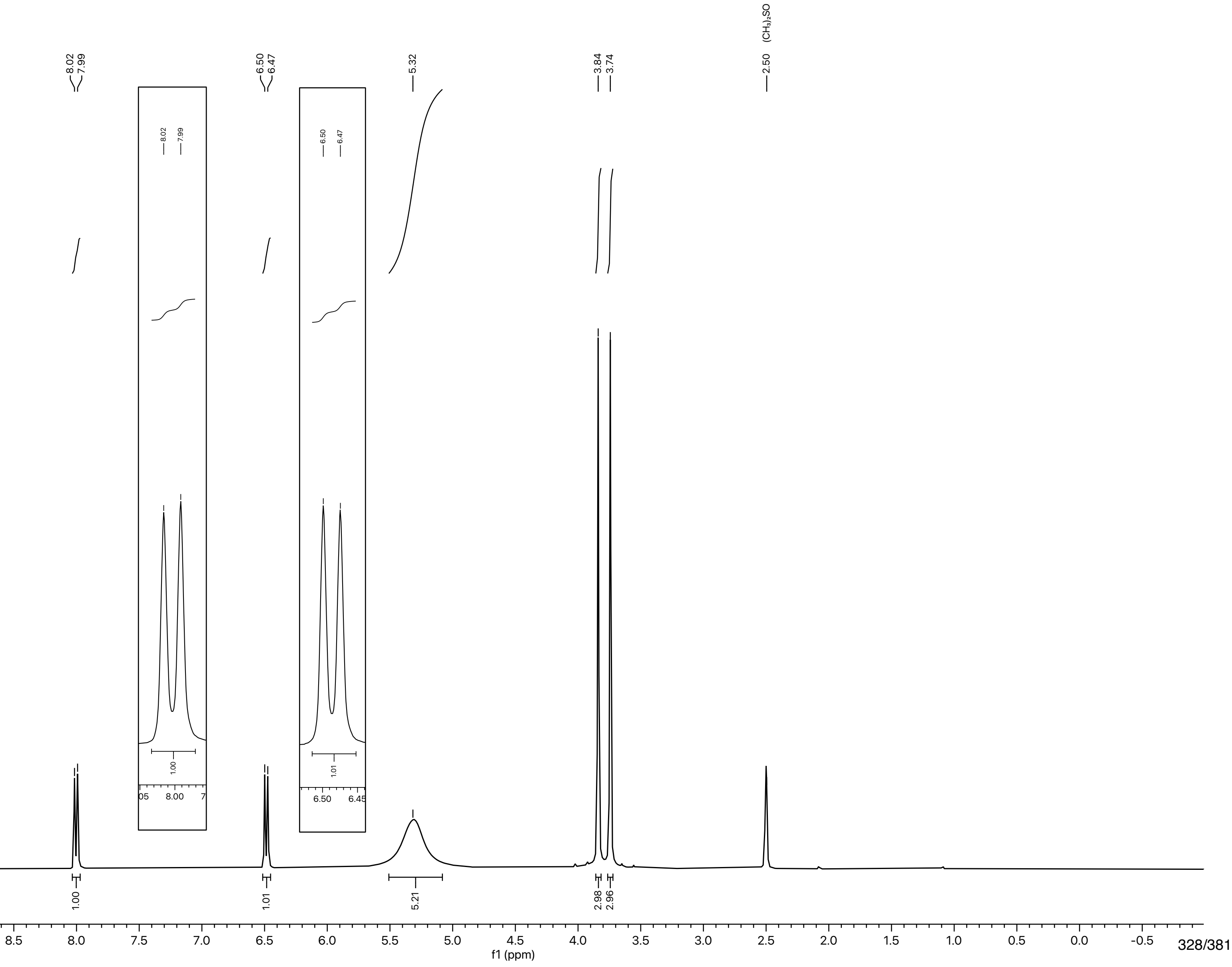

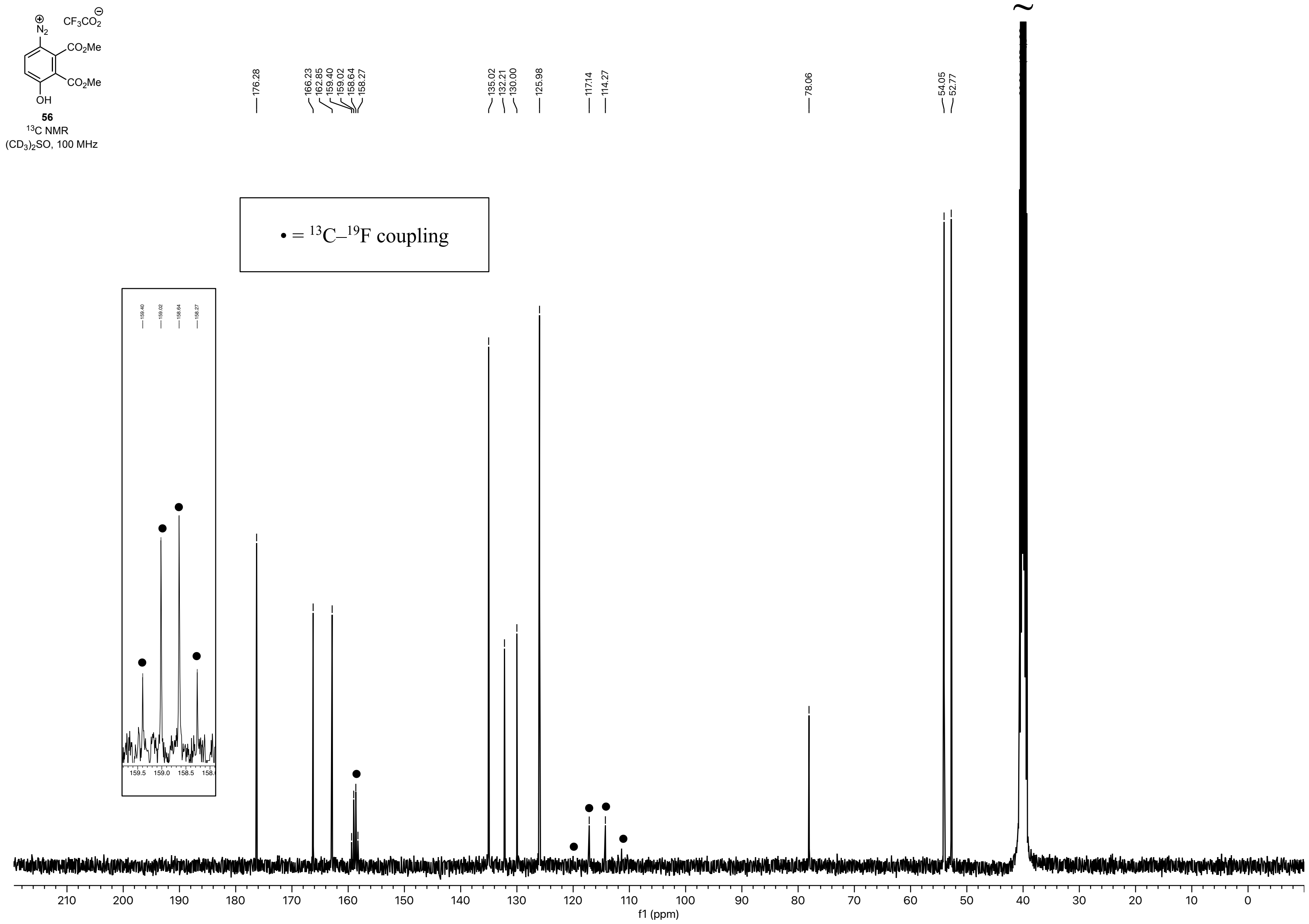
56
^{13}C NMR
$(CD_3)_2SO$, 100 MHz
• = ^{13}C–^{19}F coupling
176.28
166.23
162.85
159.40
159.02
158.64
158.27
135.02
132.21
130.00
125.98
117.14
114.27
78.06
54.05
52.77
f1 (ppm)

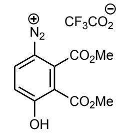

56
^{19}F NMR
$CDCl_3$, 377 MHz

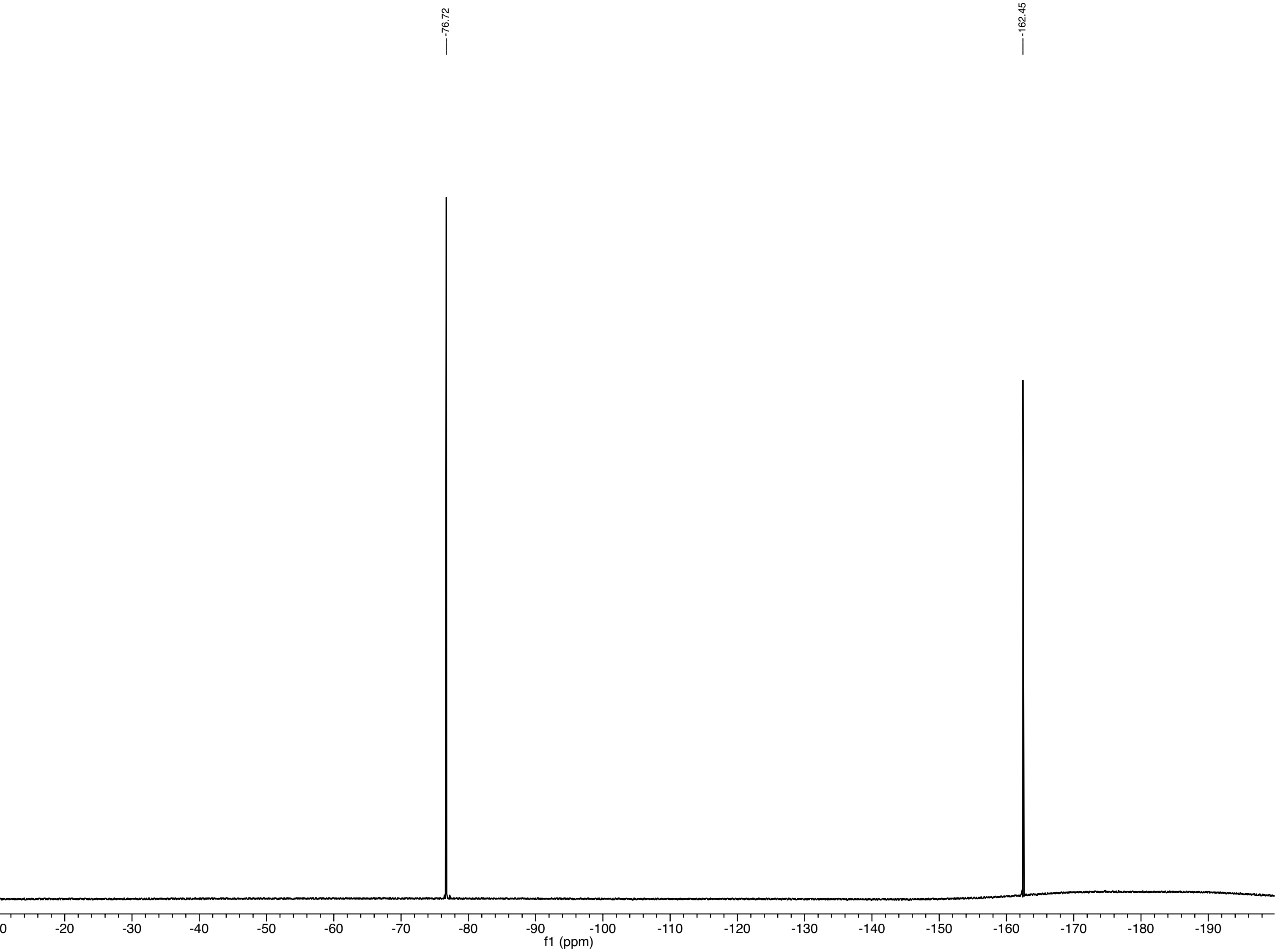

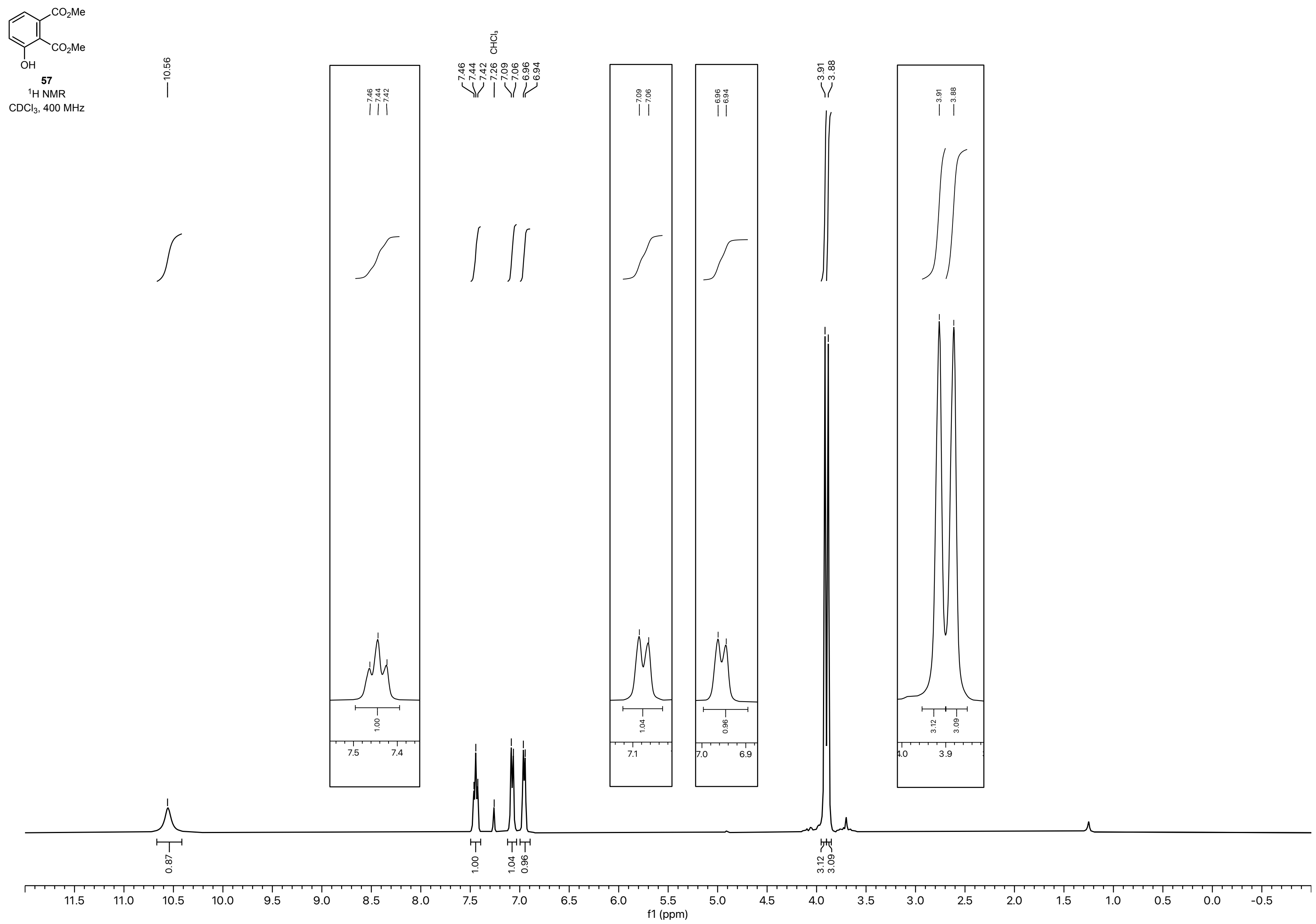

CO2Me
CO2Me
OH
57
1H NMR
CDCl3, 400 MHz
10.56
7.46
7.44
7.42
7.26
CHCl3
7.09
7.06
6.96
6.94
3.91
3.88
0.87
1.00
1.04
0.96
3.12
3.09
f1 (ppm)

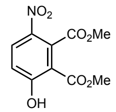

58
^{1}H NMR
$CDCl_3$, 400 MHz

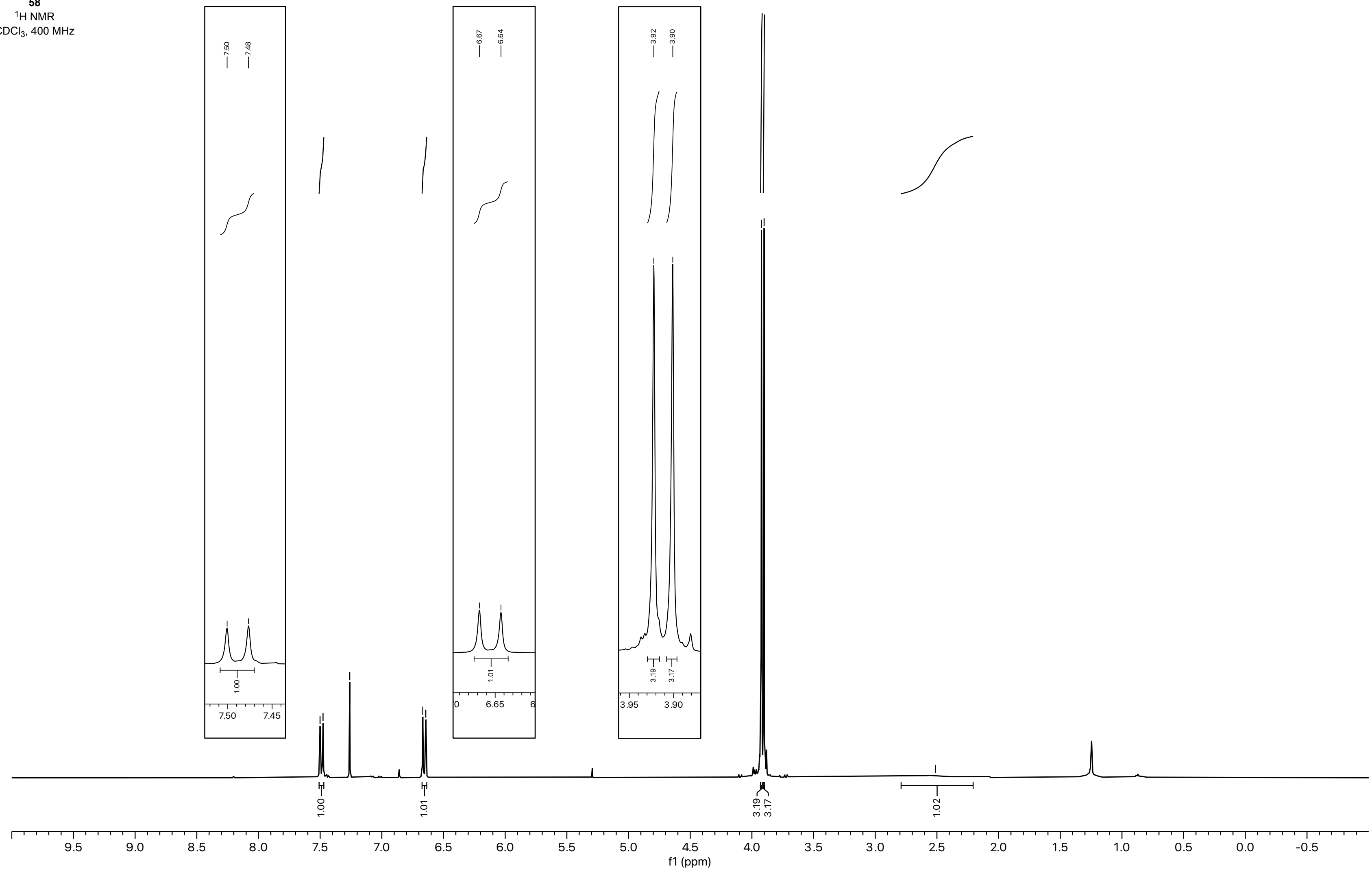

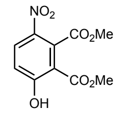

58

^{13}C NMR

$CDCl_3$, 100 MHz

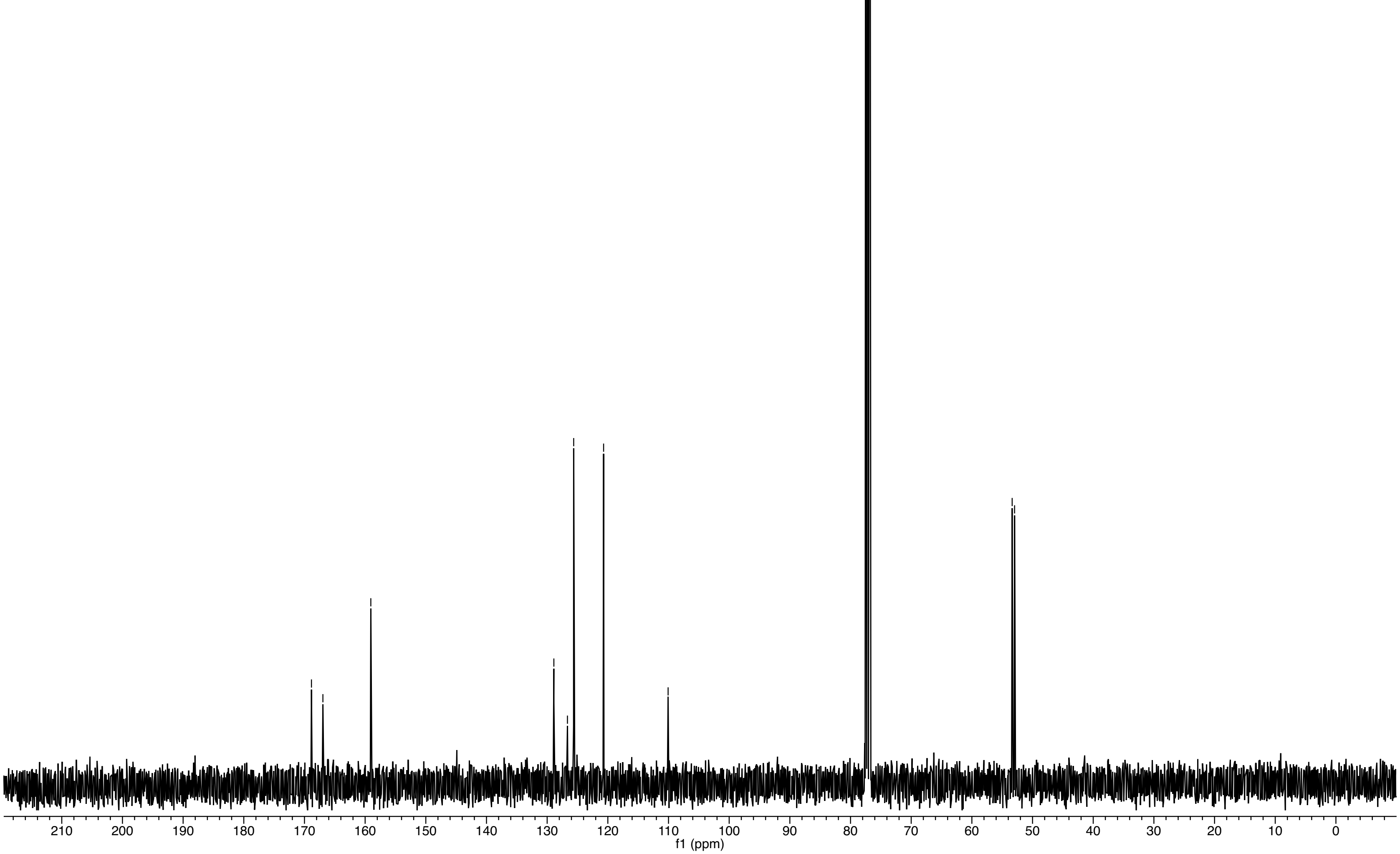

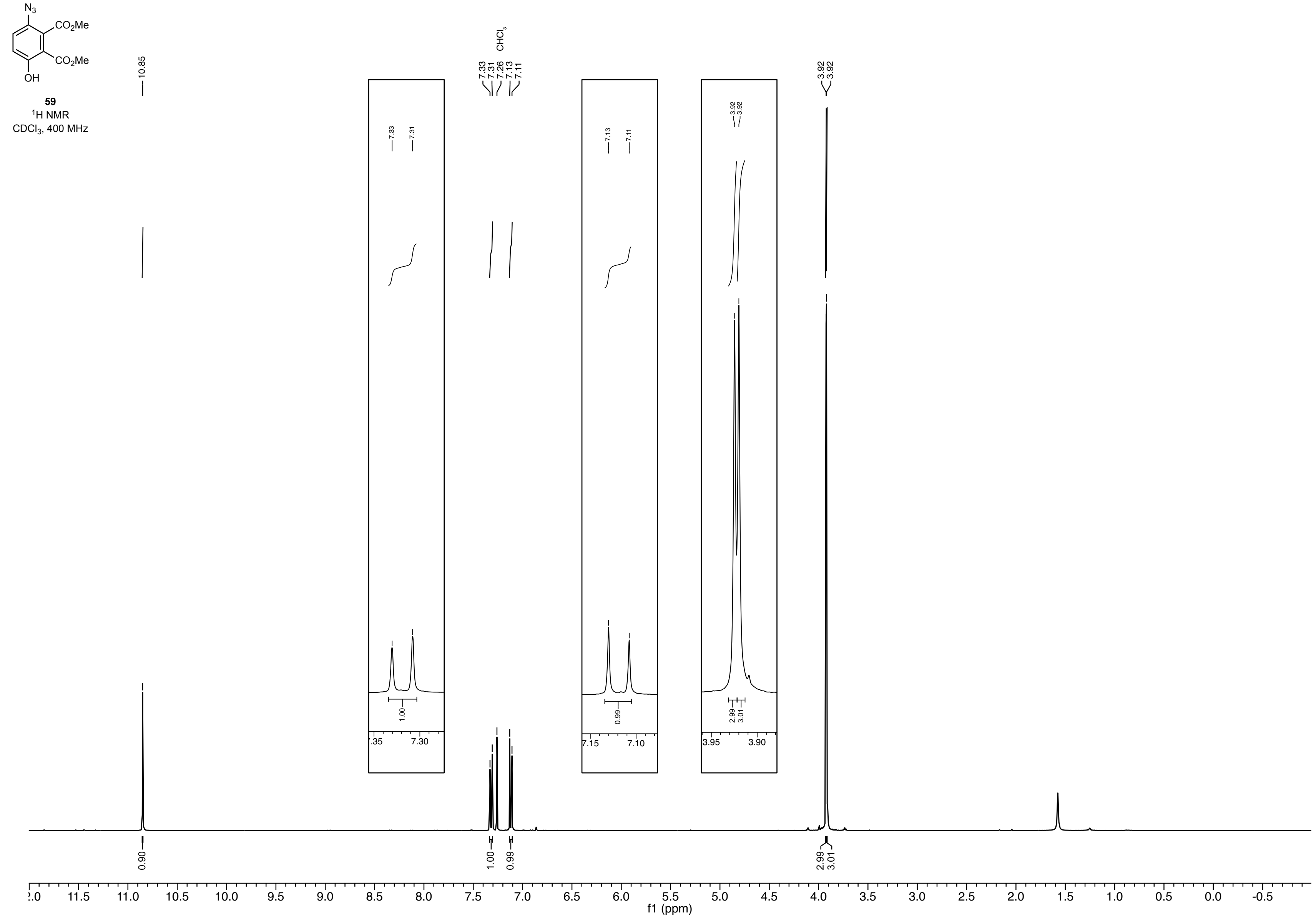

59
^{1}H NMR
$CDCl_3$, 400 MHz

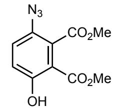

59

^{13}C NMR

$CDCl_3$, 100 MHz

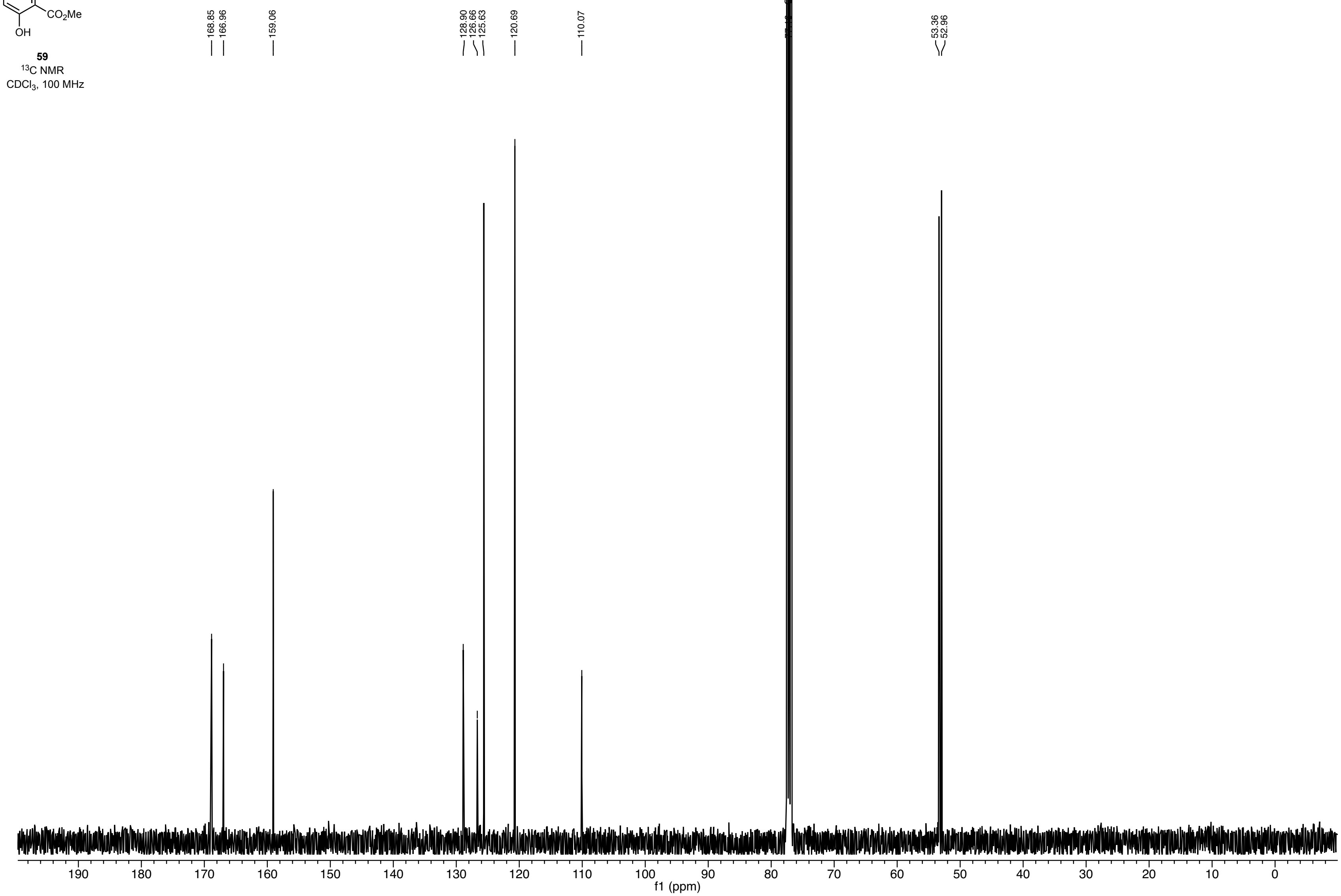

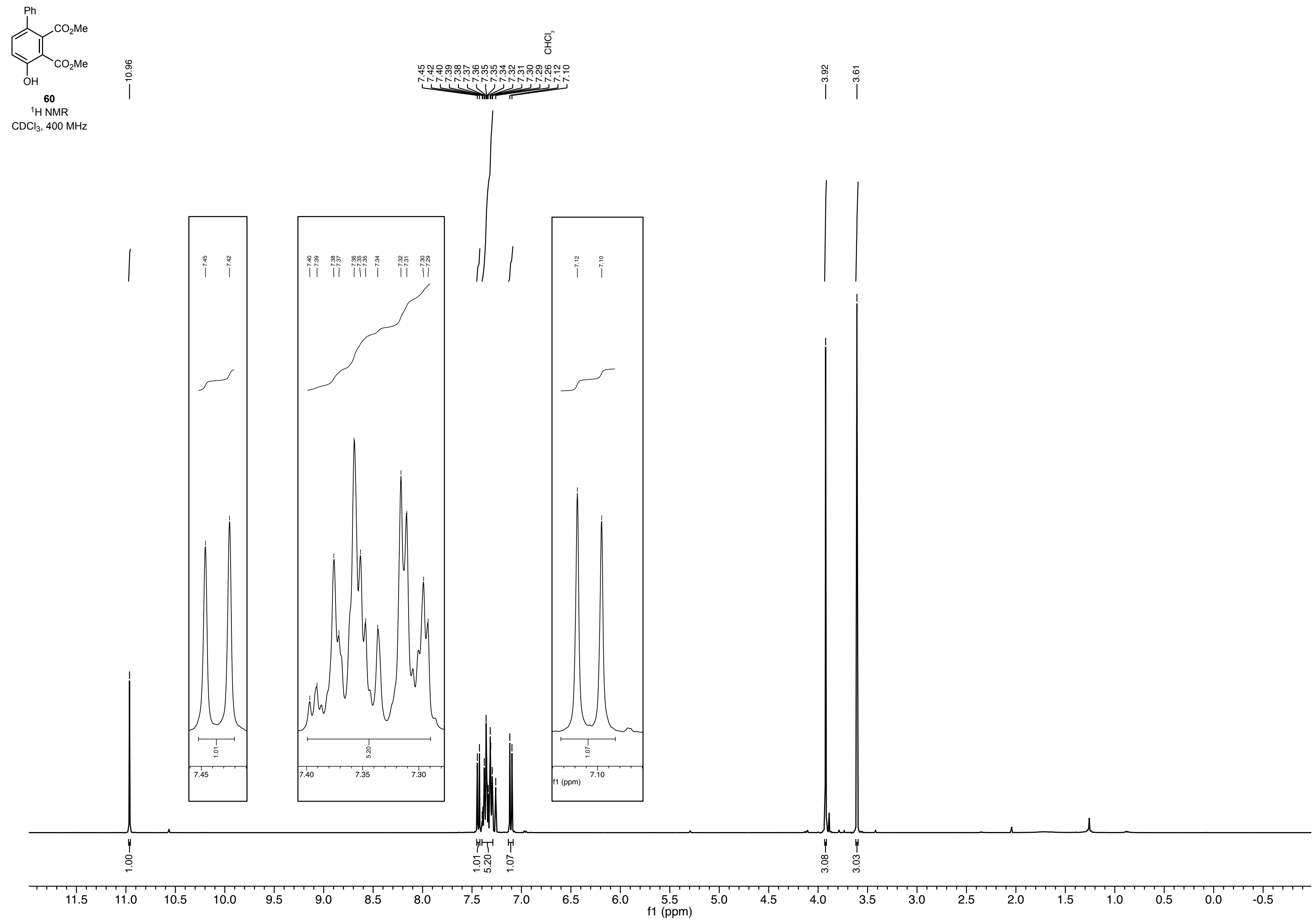
Ph
CO2Me
CO2Me
OH
60
1H NMR
CDCl3, 400 MHz
10.96
7.45 7.42 7.40 7.39 7.38 7.37 7.36 7.35 7.35 7.34 7.32 7.31 7.30 7.29 7.29 7.26 7.12 7.10
CHCl3
3.92
3.61
1.00
1.01
5.20
1.07
3.08
3.03
f1 (ppm)

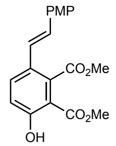

61
^{1}H NMR
$CDCl_3$, 400 MHz

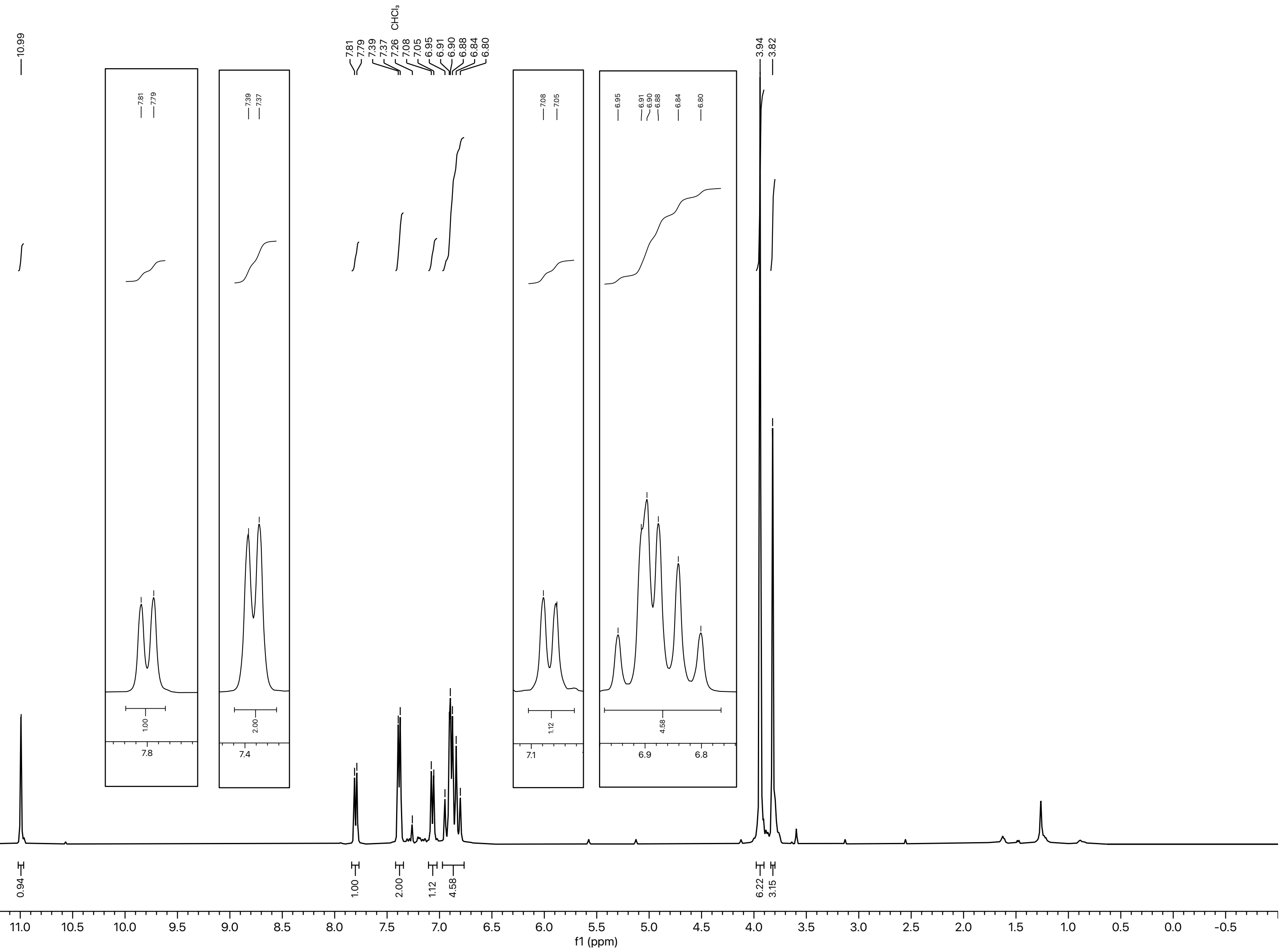

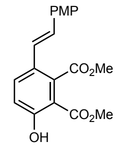

61
^{13}C NMR
$CDCl_3$, 100 MHz

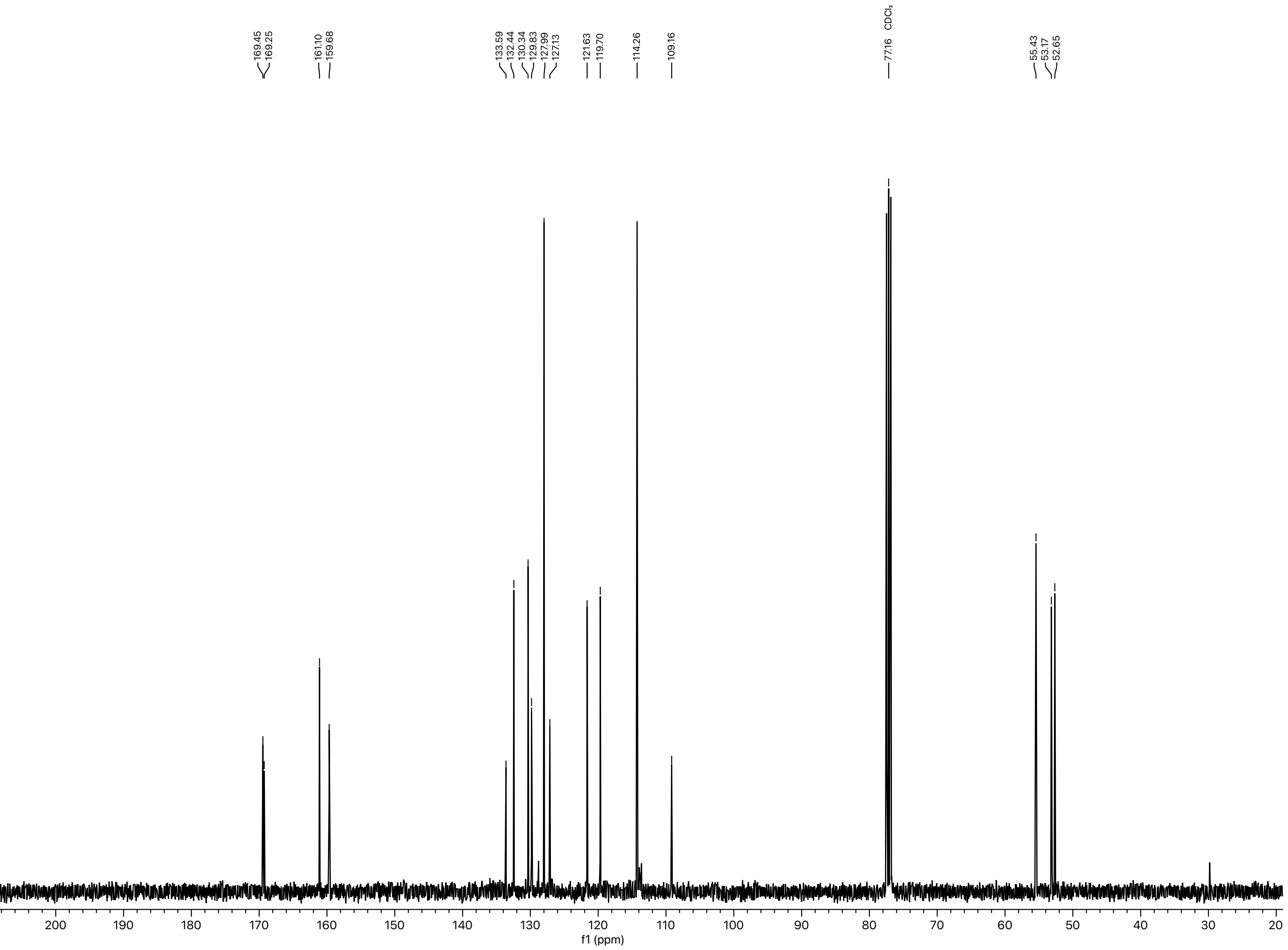

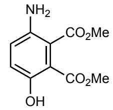

62
^{1}H NMR
$CDCl_3$, 400 MHz

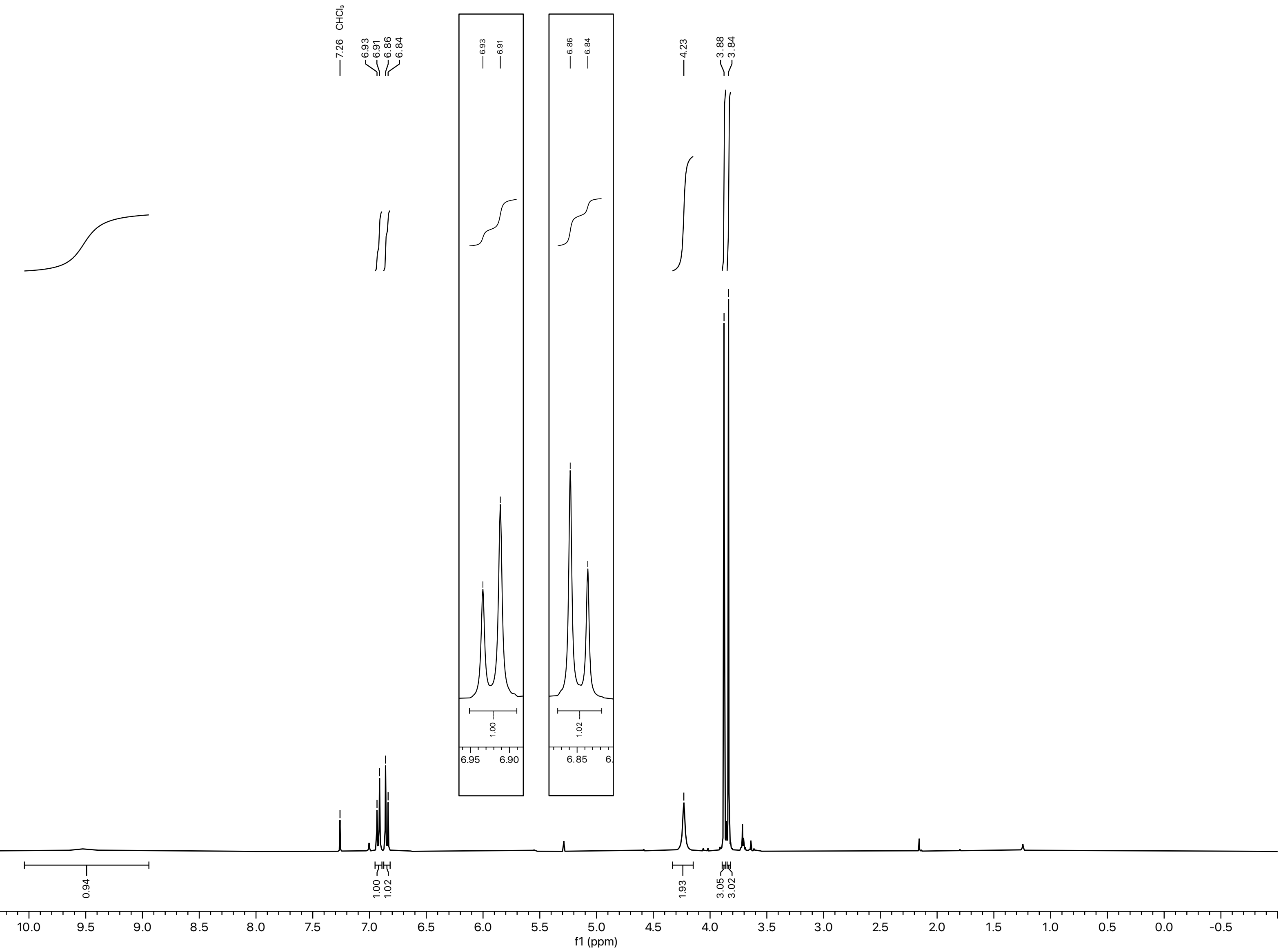

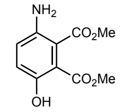

62
^{13}C NMR
$CDCl_3$, 100 MHz

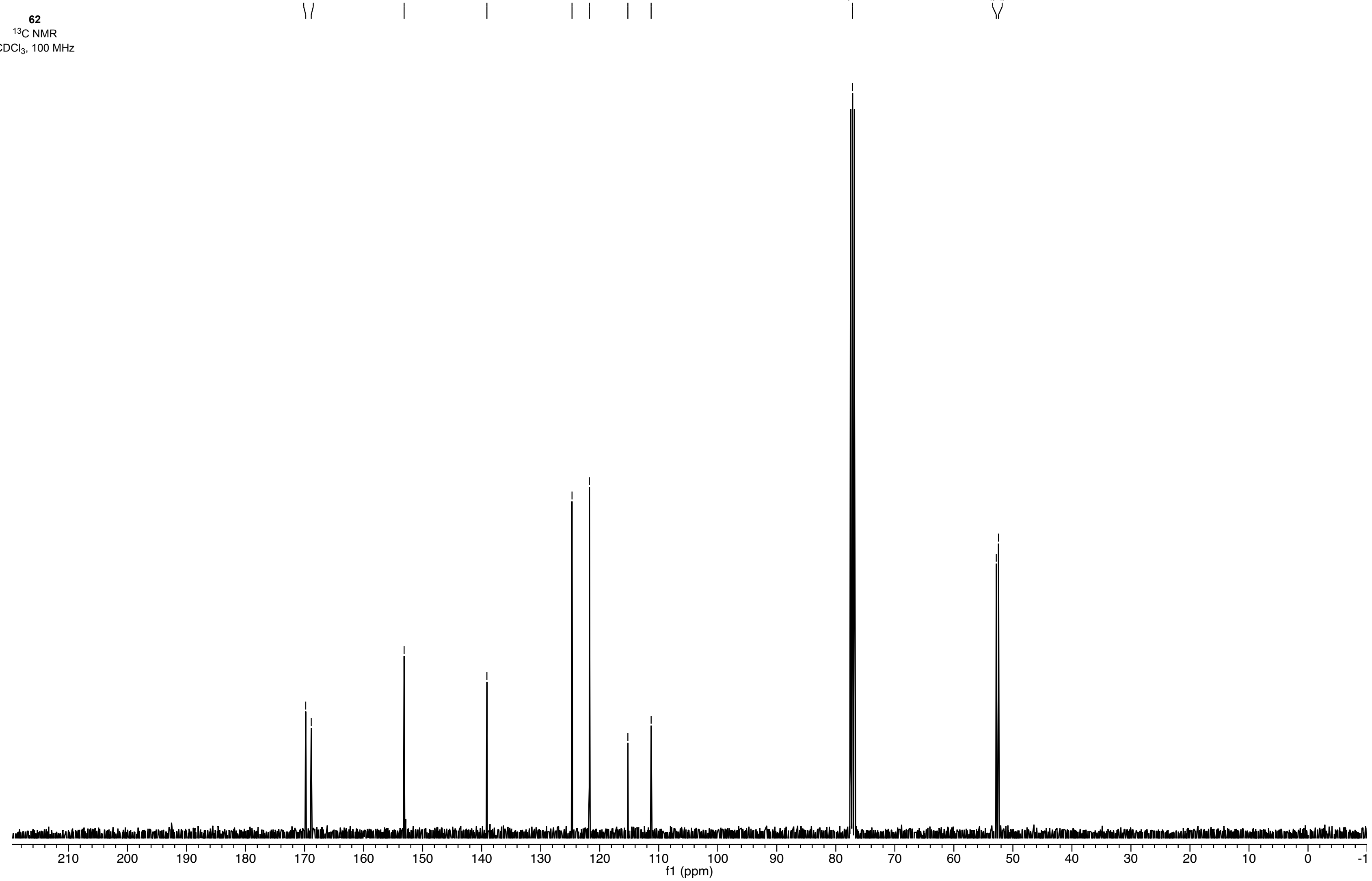

From Phthalic Anhydride 64

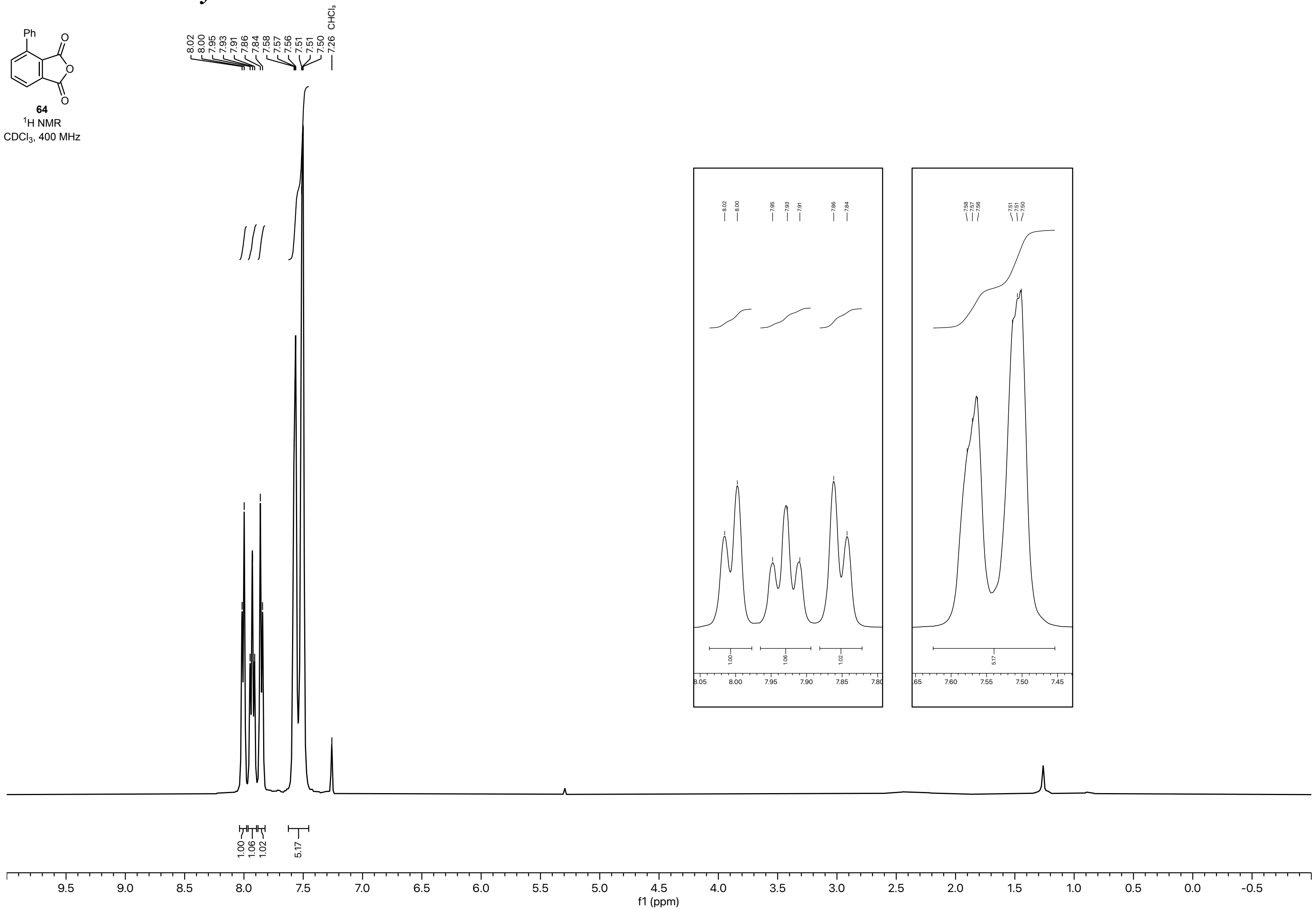

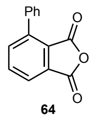

64

^{13}C NMR

$CDCl_3$, 100 MHz

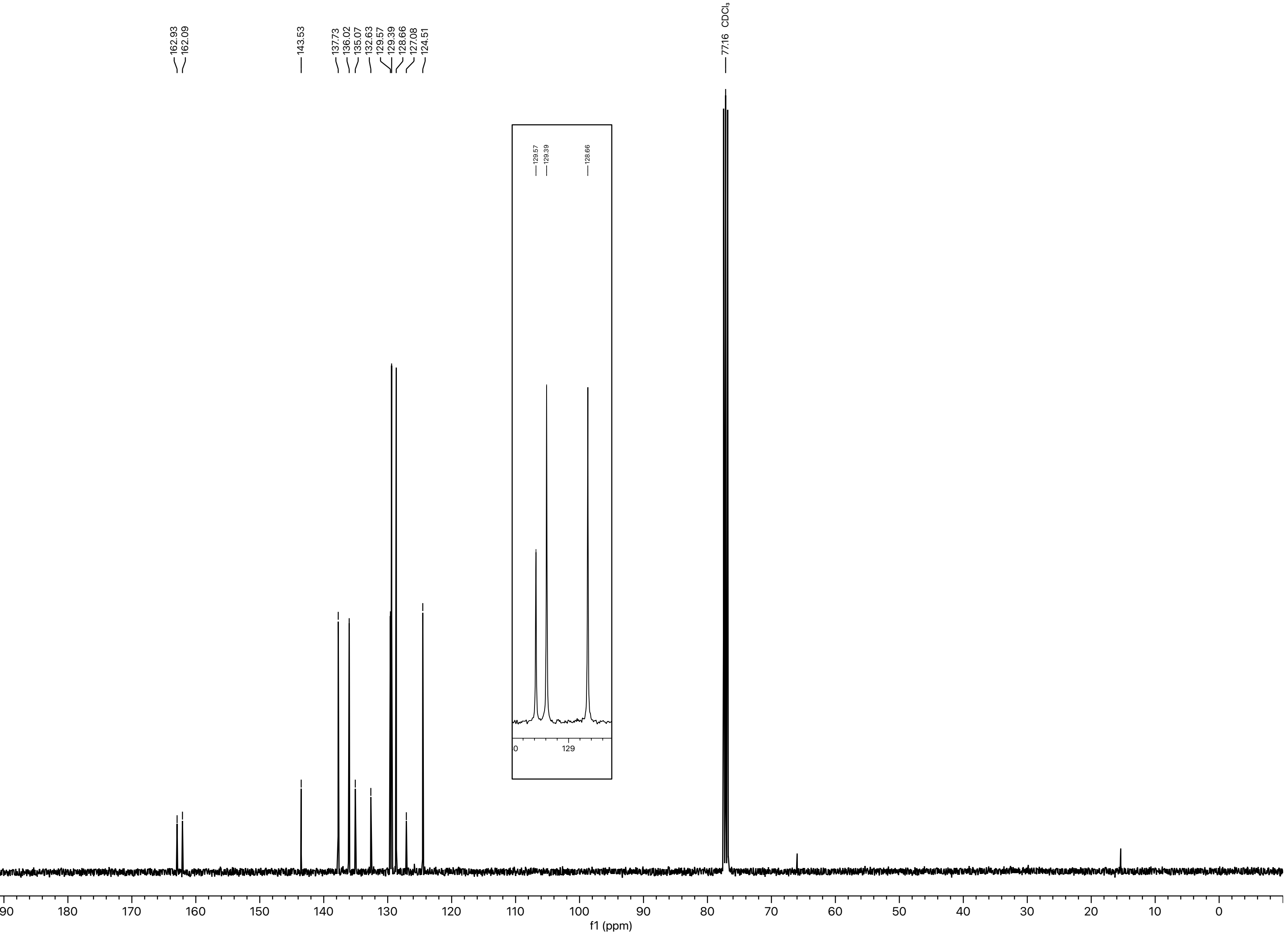

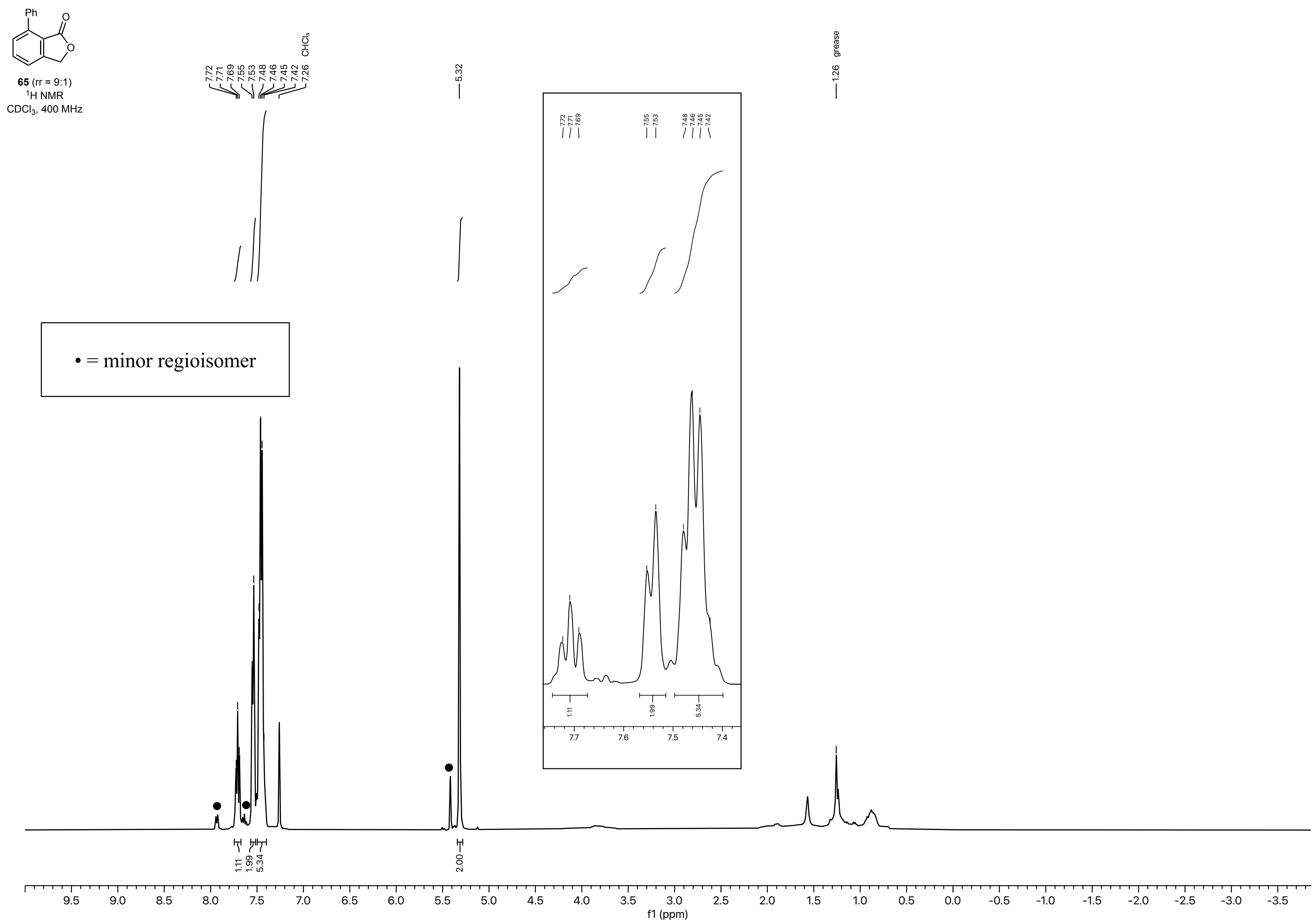
Ph
O
O
65 (rr = 9:1)
1H NMR
CDCl3, 400 MHz
7.72 7.71 7.69 7.55 7.53 7.48 7.46 7.45 7.42 7.26 CHCl3
5.32
1.26 grease
• = minor regioisomer
1.11 1.99 5.34
2.00
f1 (ppm)

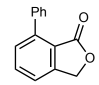

65 (rr = 9:1)
^{13}C NMR
$CDCl_3$, 100 MHz

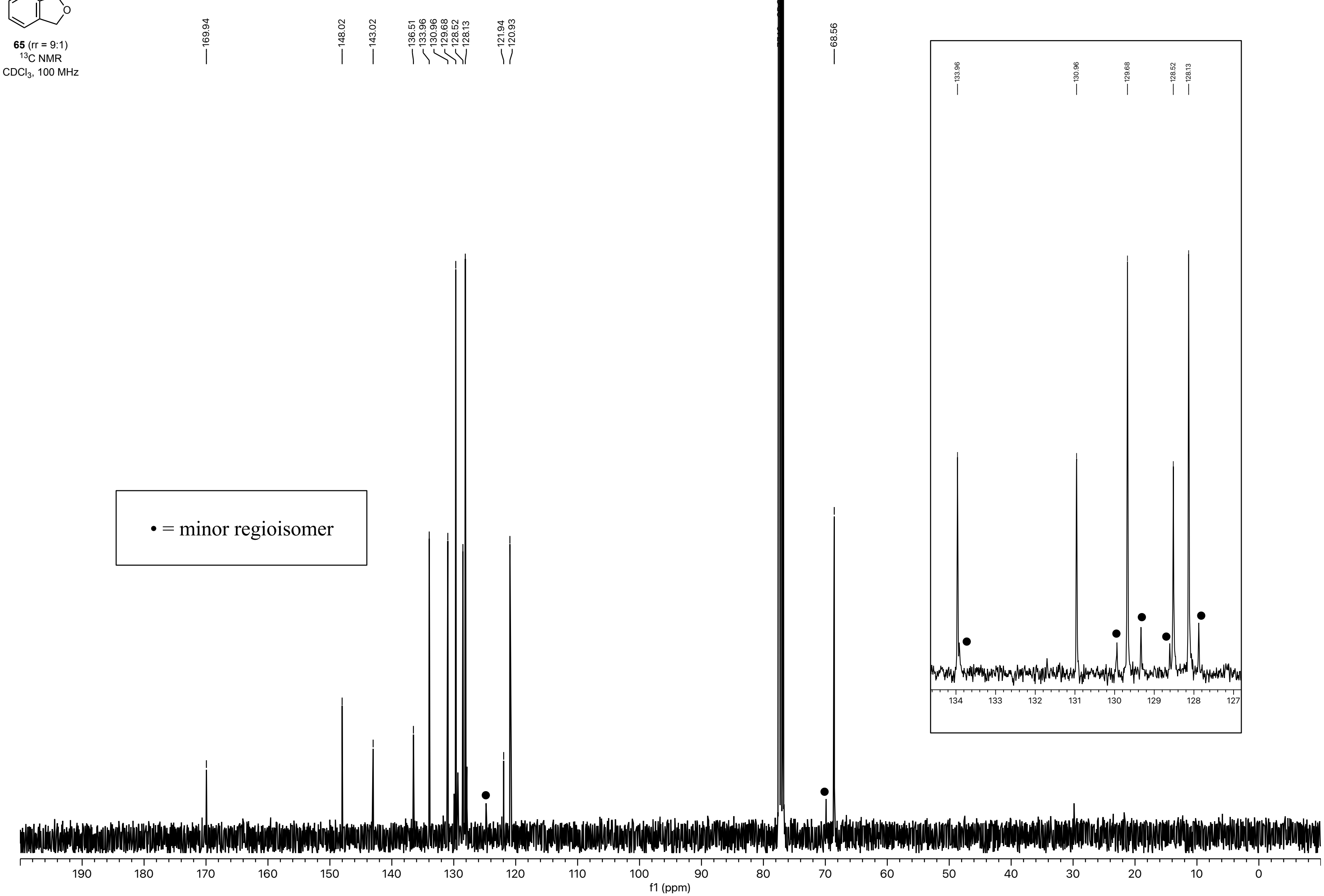

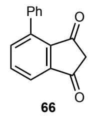

66
^{1}H NMR
$CDCl_3$, 400 MHz

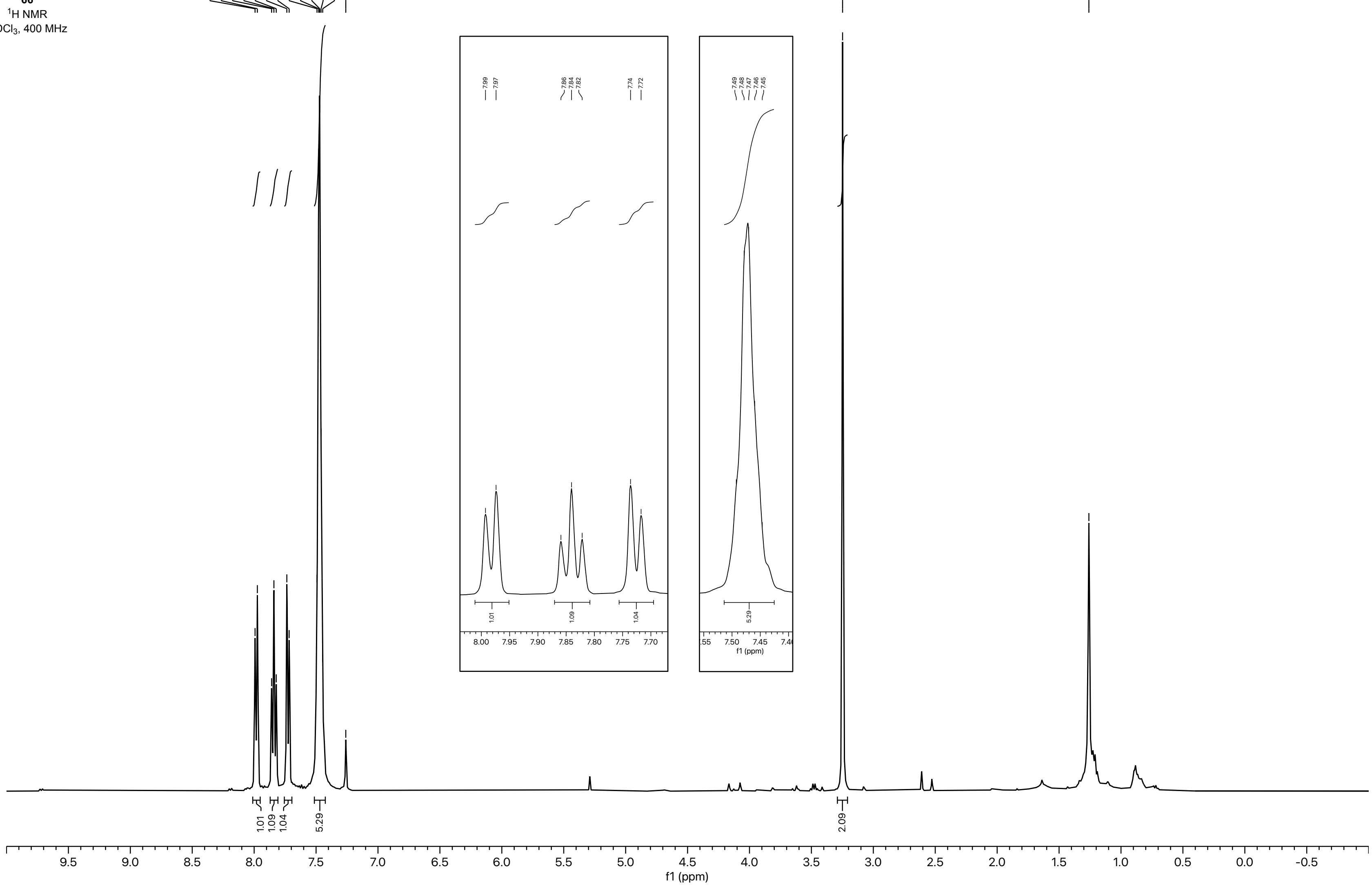

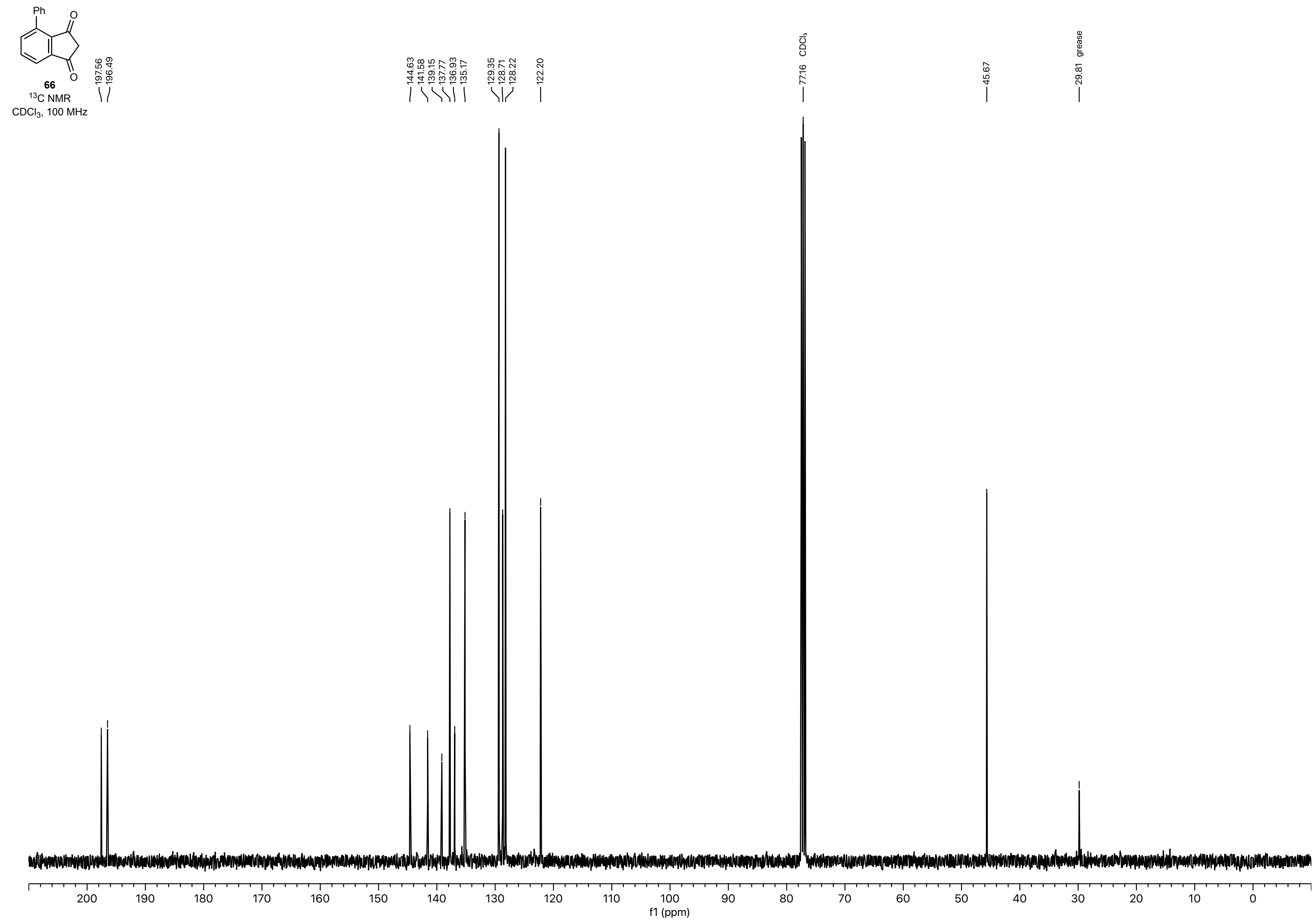
Ph
O
O
66
^{13}C NMR
$CDCl_3$, 100 MHz
197.56
196.49
144.63
141.58
139.15
137.77
136.93
135.17
129.35
128.71
128.22
122.20
77.16 $CDCl_3$
45.67
29.81 grease
200 190 180 170 160 150 140 130 120 110 100 90 80 70 60 50 40 30 20 10 0
f1 (ppm)

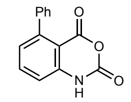

67

^1H NMR

$CDCl_3$, 400 MHz

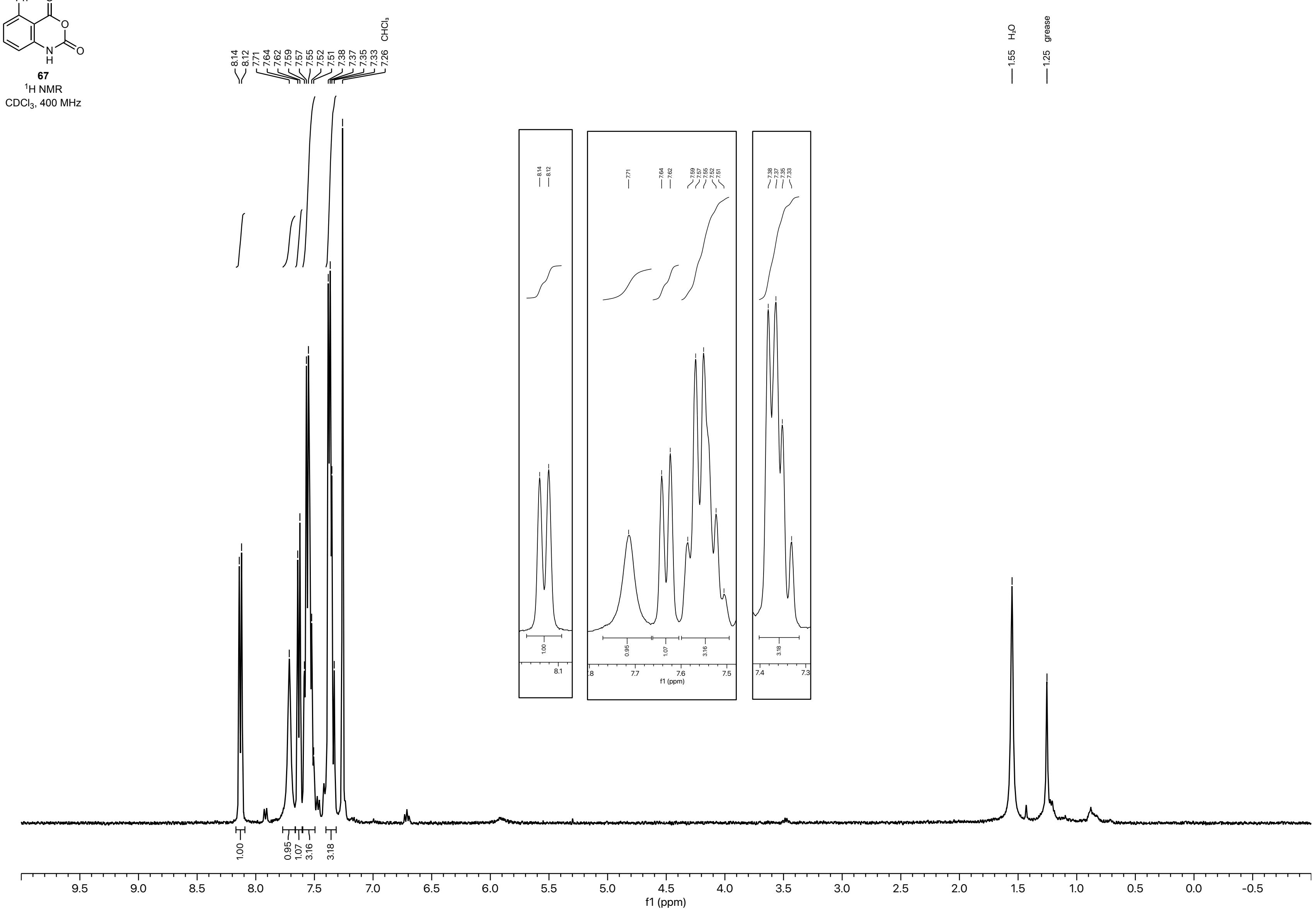

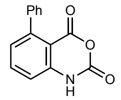

67
^{13}C NMR
$CDCl_3$, 150 MHz

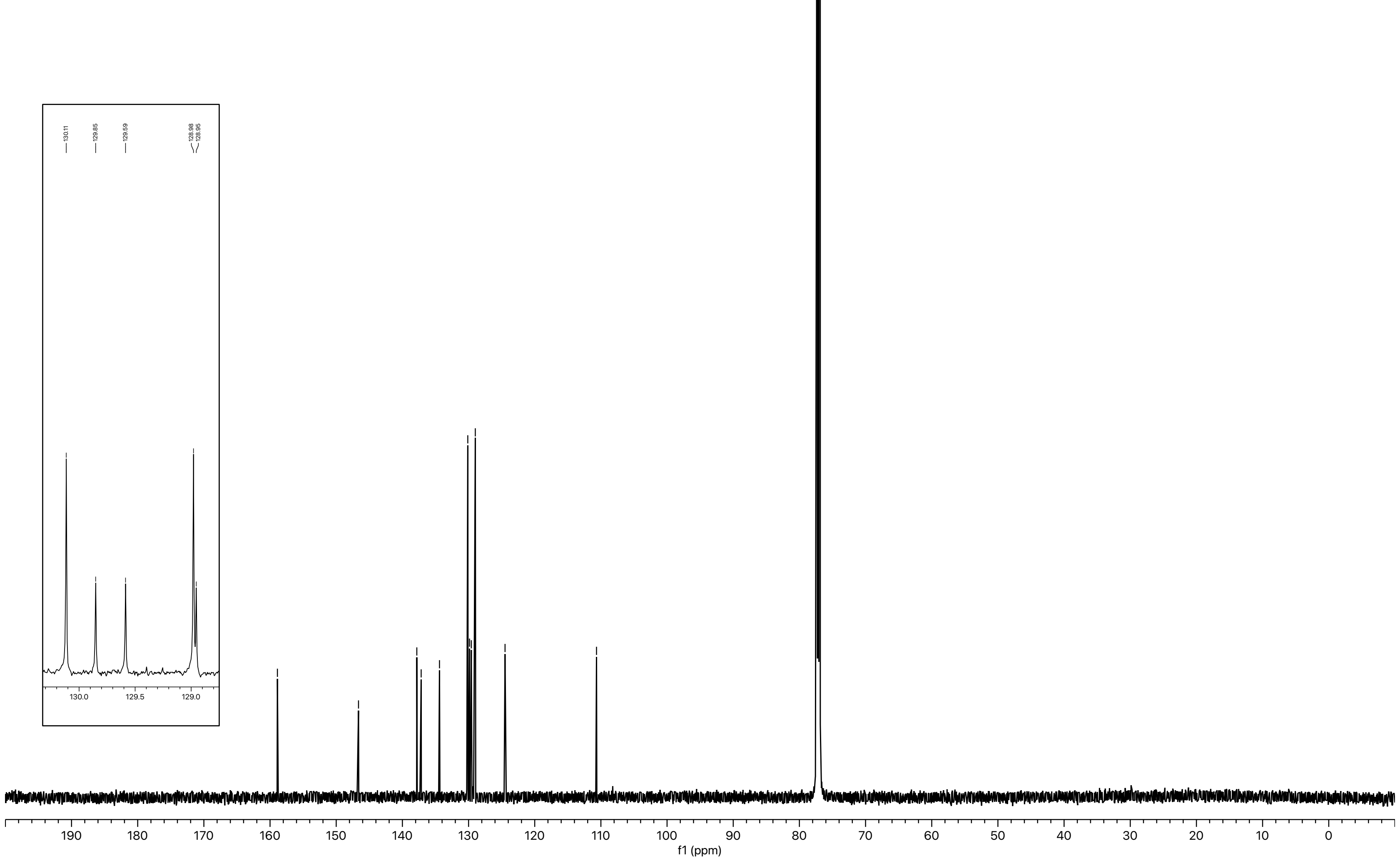

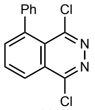

68
^{1}H NMR
$(CD_3)_2SO$, 400 MHz

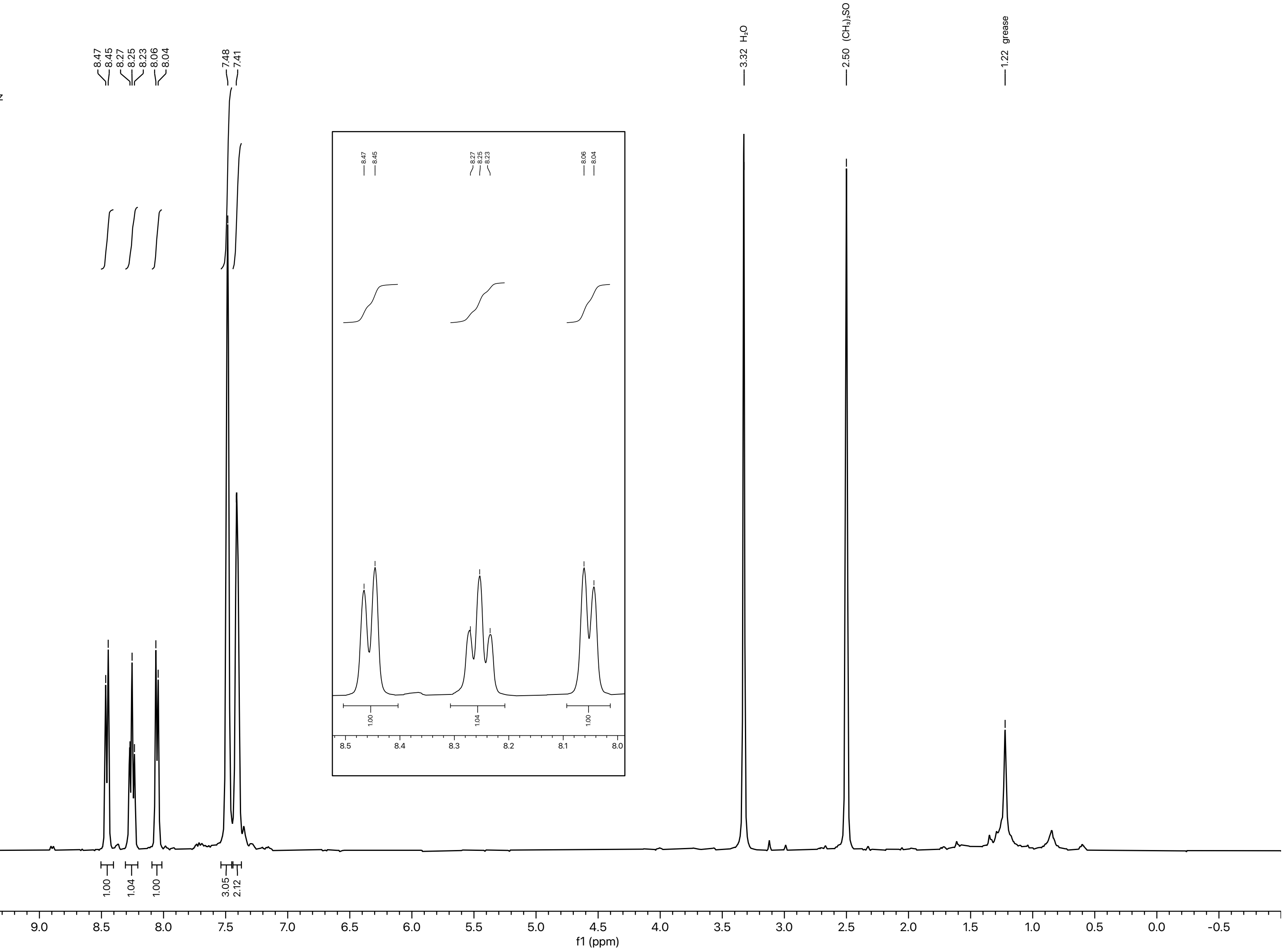

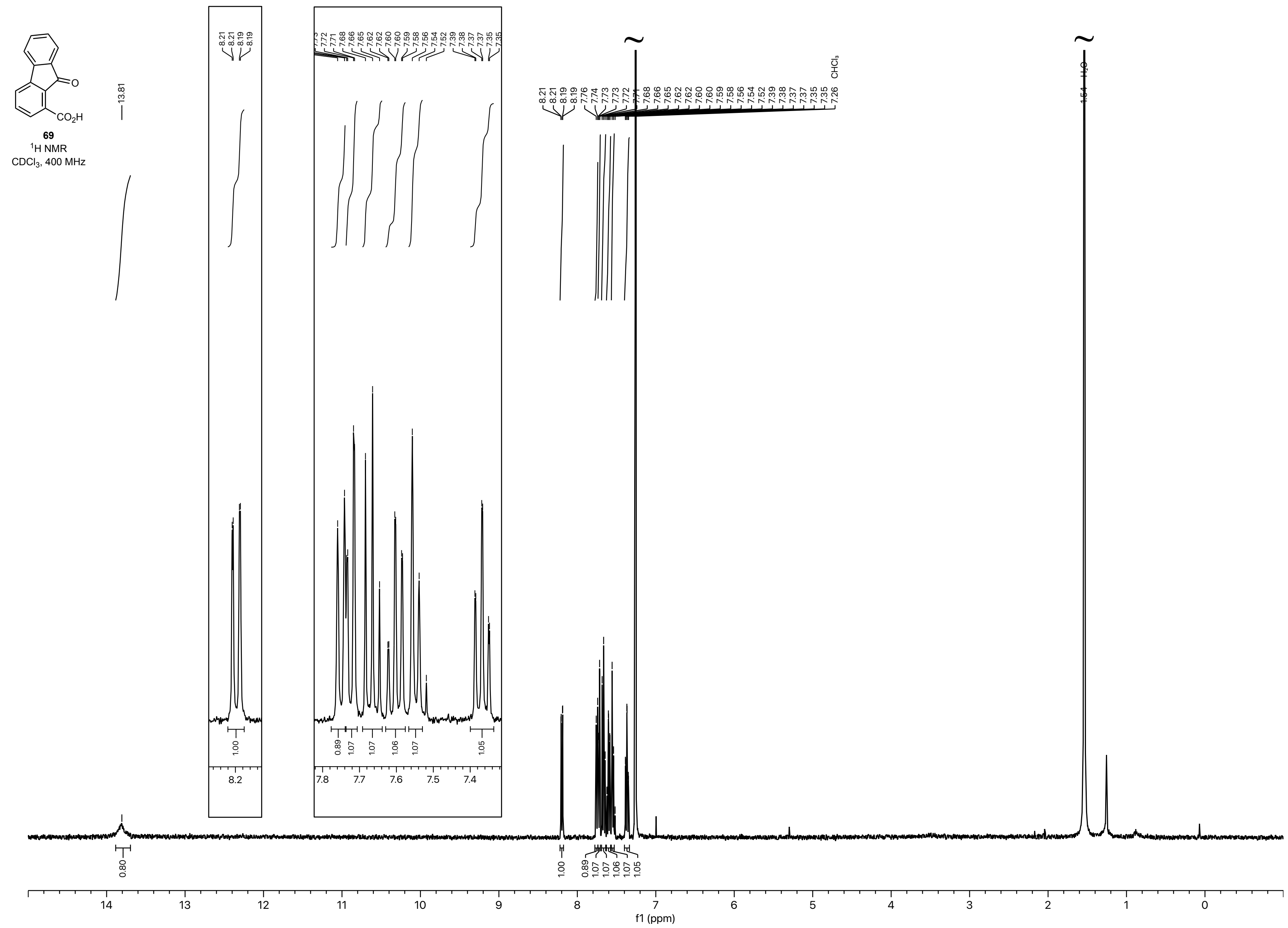
CO2H
O
69
1H NMR
CDCl3, 400 MHz
13.81
CHCl3
H2O
f1 (ppm)

From Quinone Diazide 53

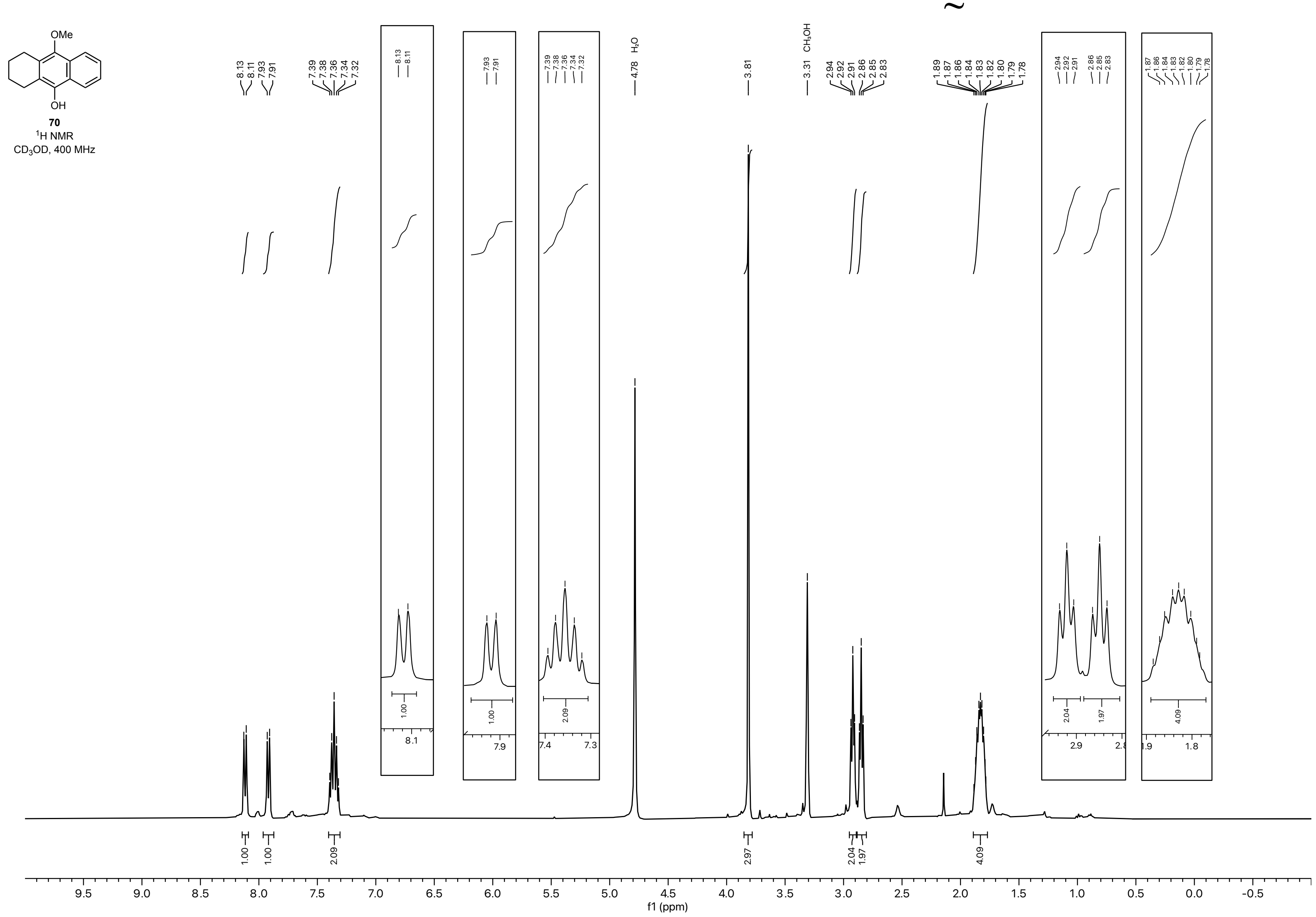

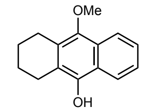

70
^{13}C NMR
CD_3OD, 100 MHz

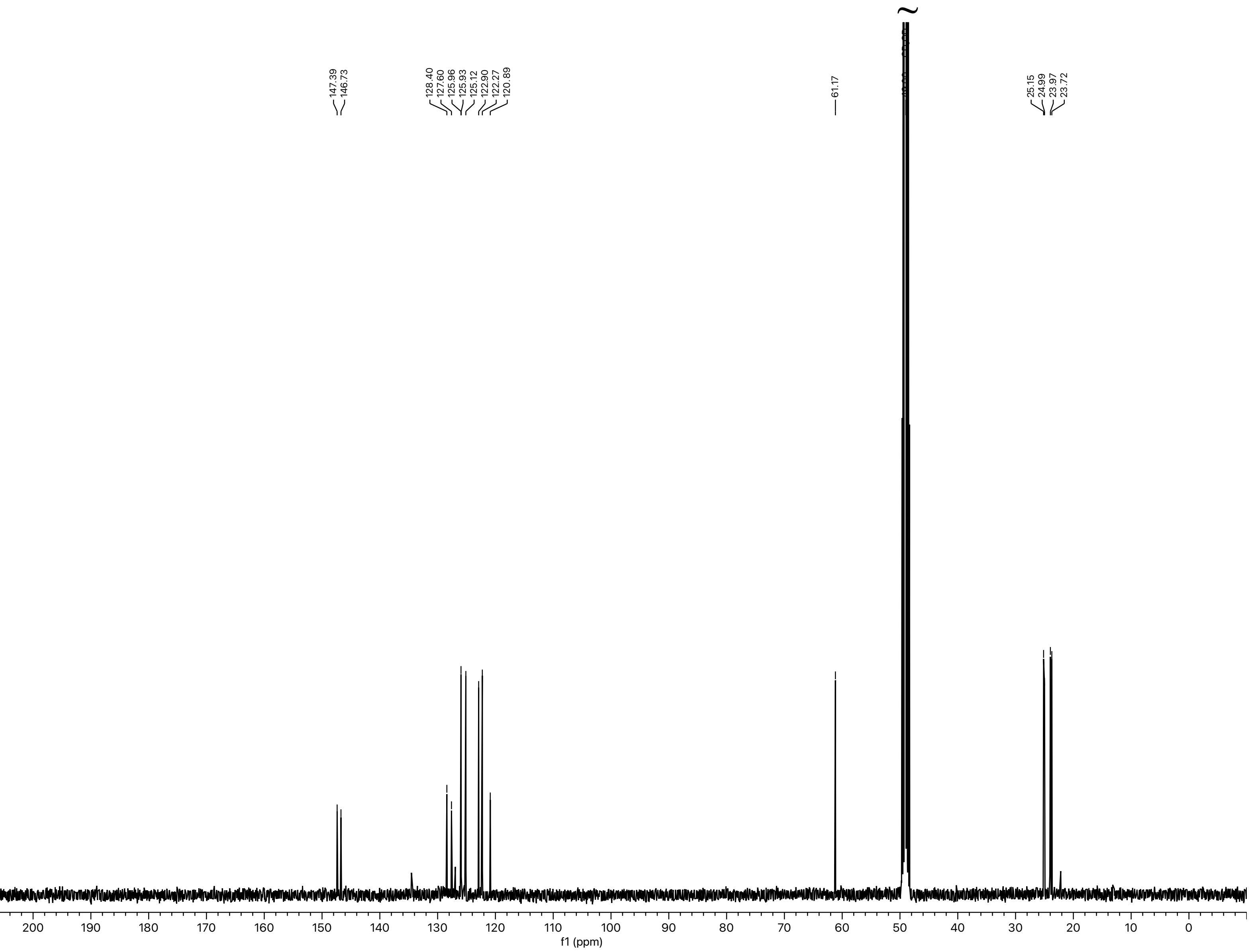

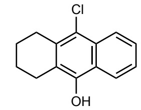

71
^1H NMR
$CDCl_3$, 400 MHz

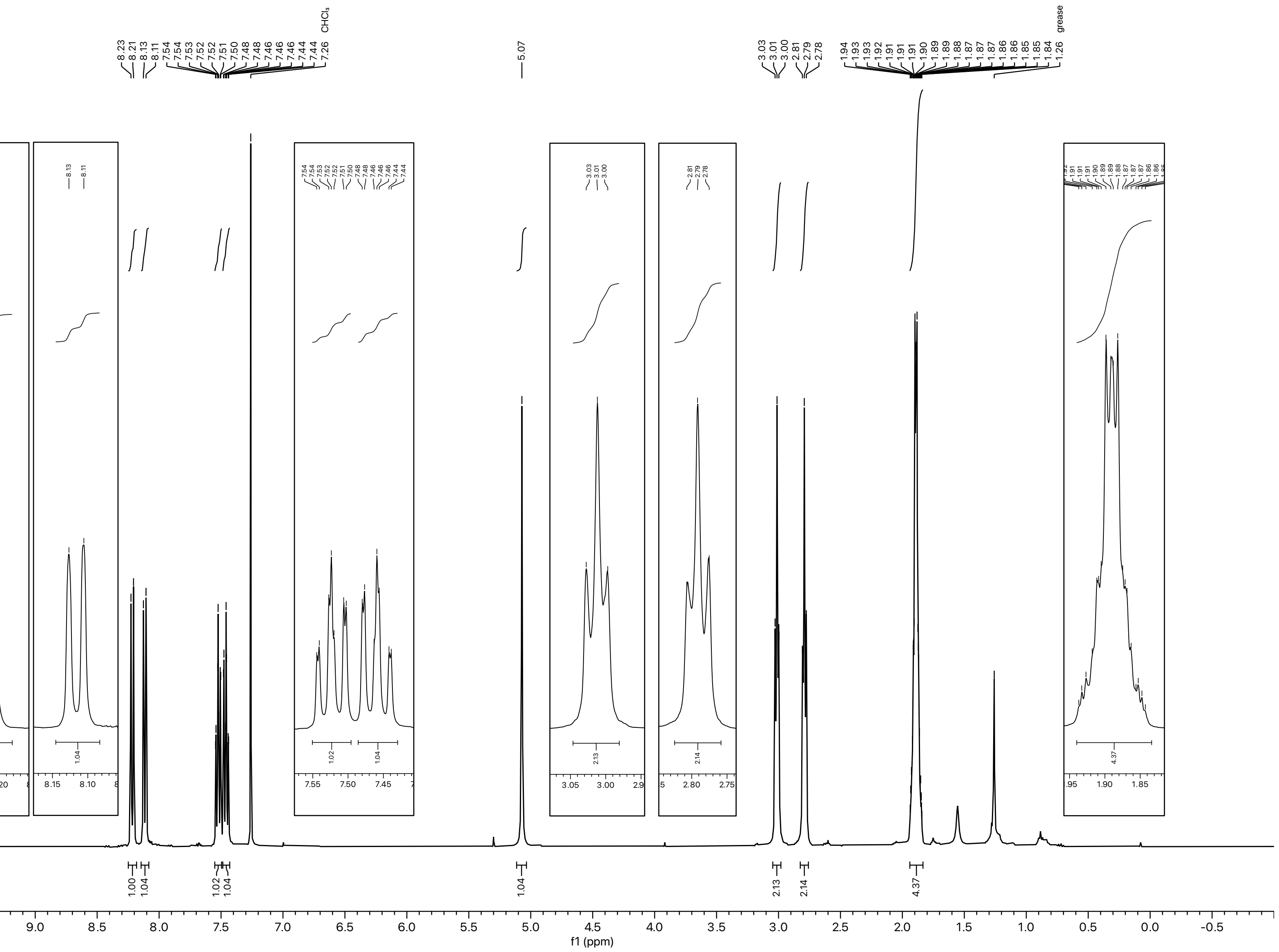

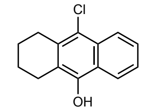

71
^{13}C NMR
$CDCl_3$, 100 MHz

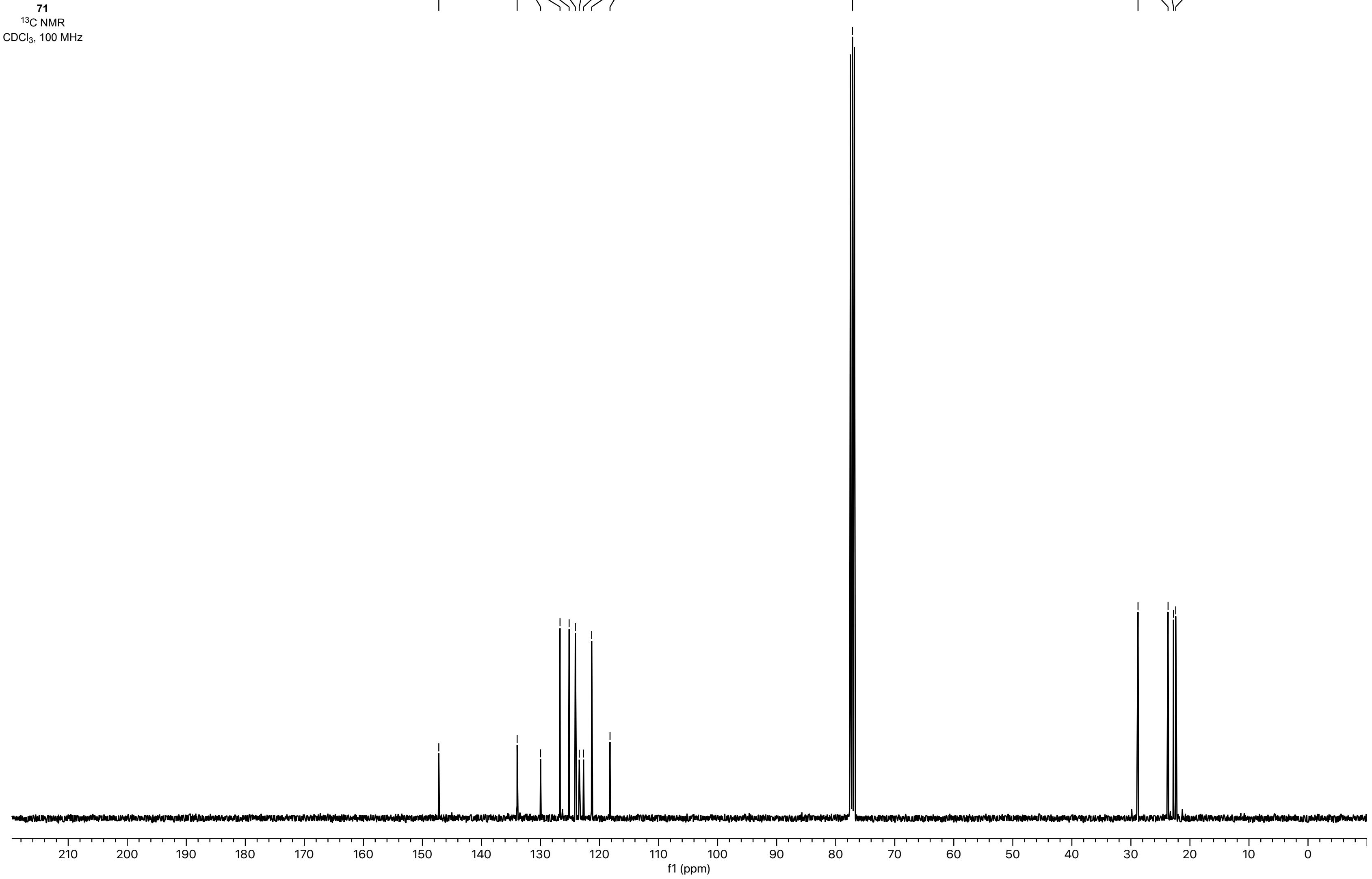

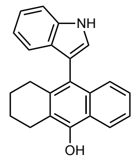

72

^{1}H NMR

CD_3OD, 400 MHz

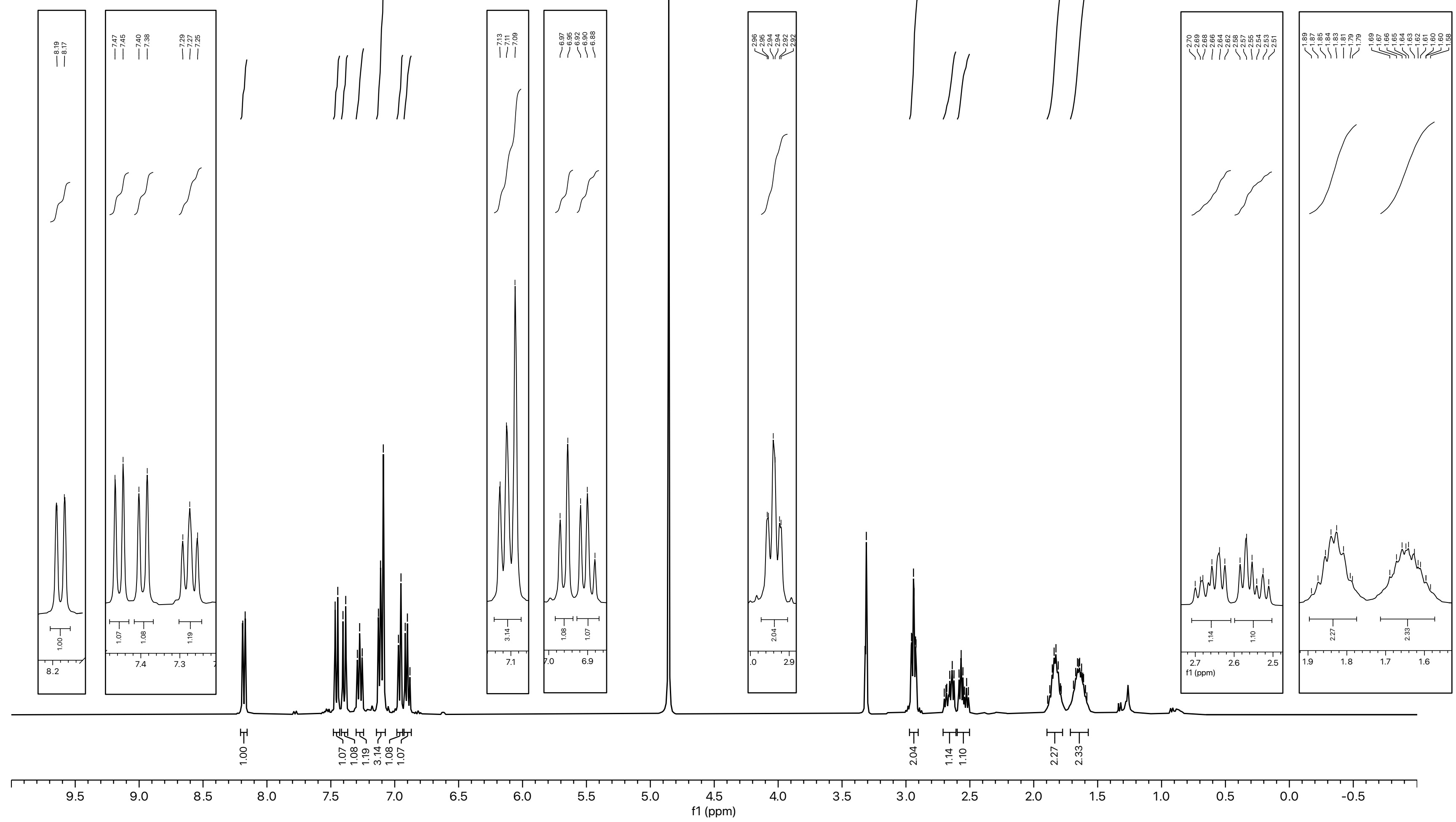

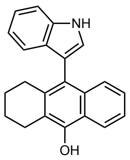

72
^{13}C NMR
CD_3OD, 100 MHz

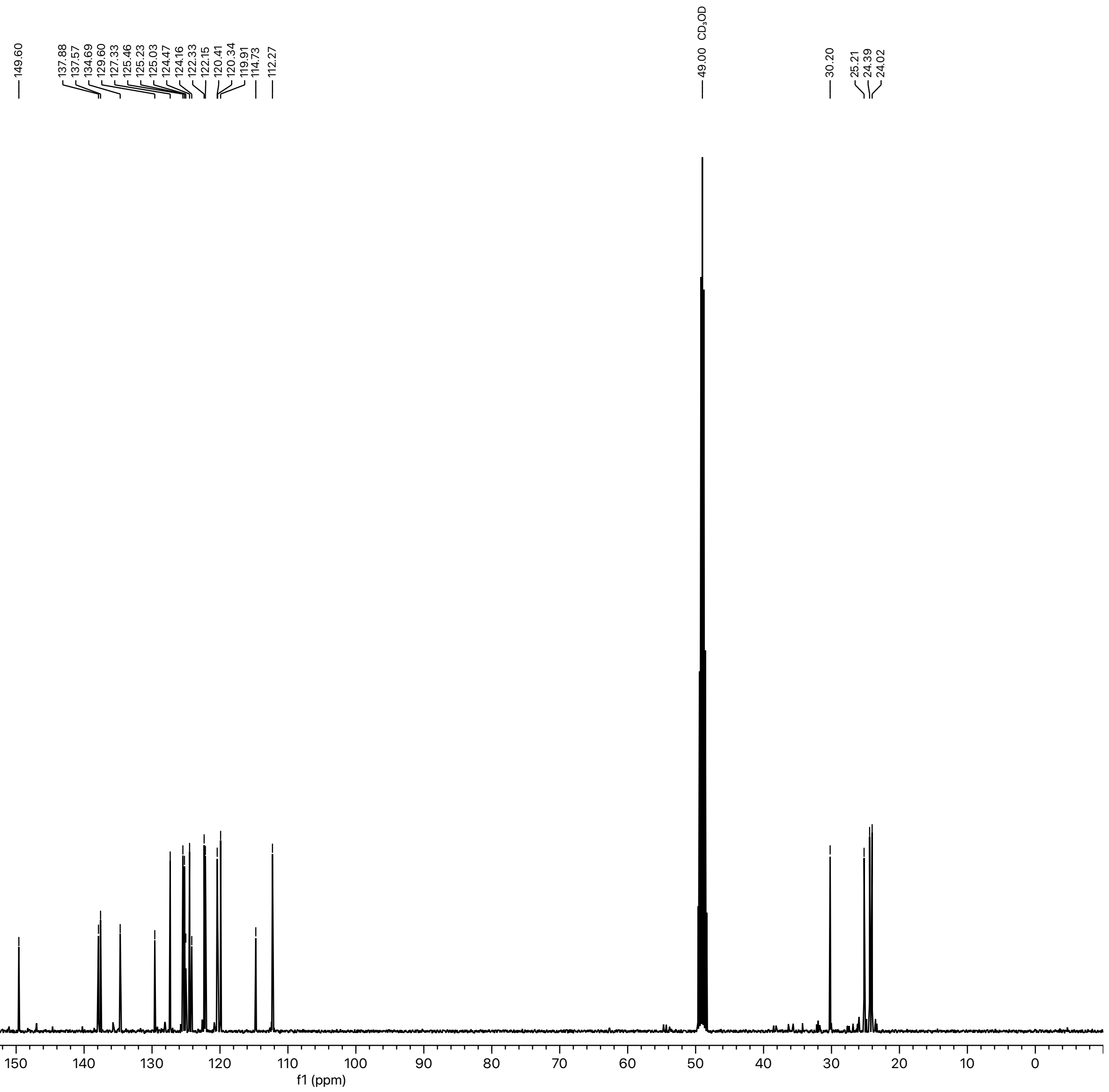

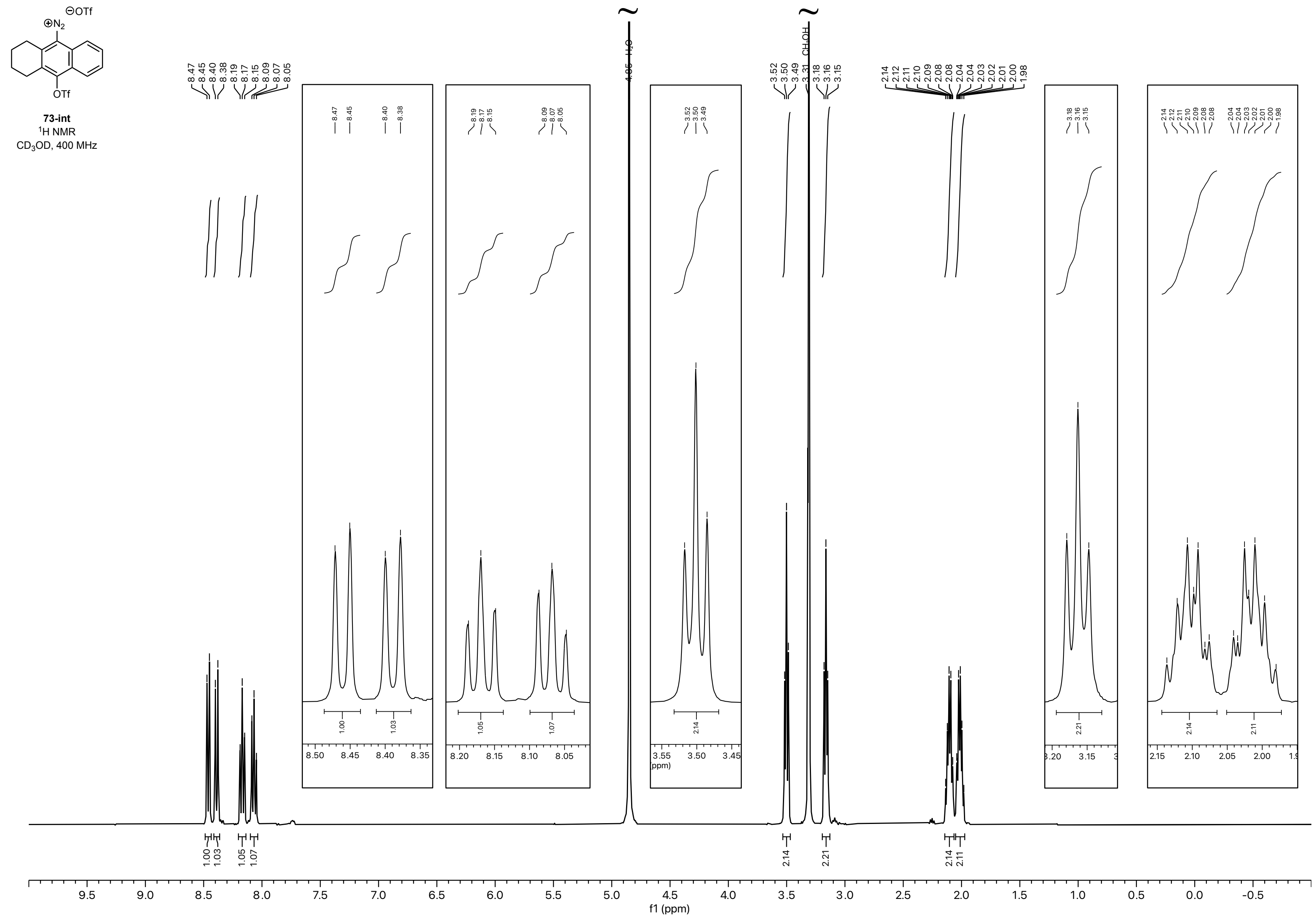

73-int
^1H NMR
CD_3OD, 400 MHz
H_2O
CH_3OH
f1 (ppm)

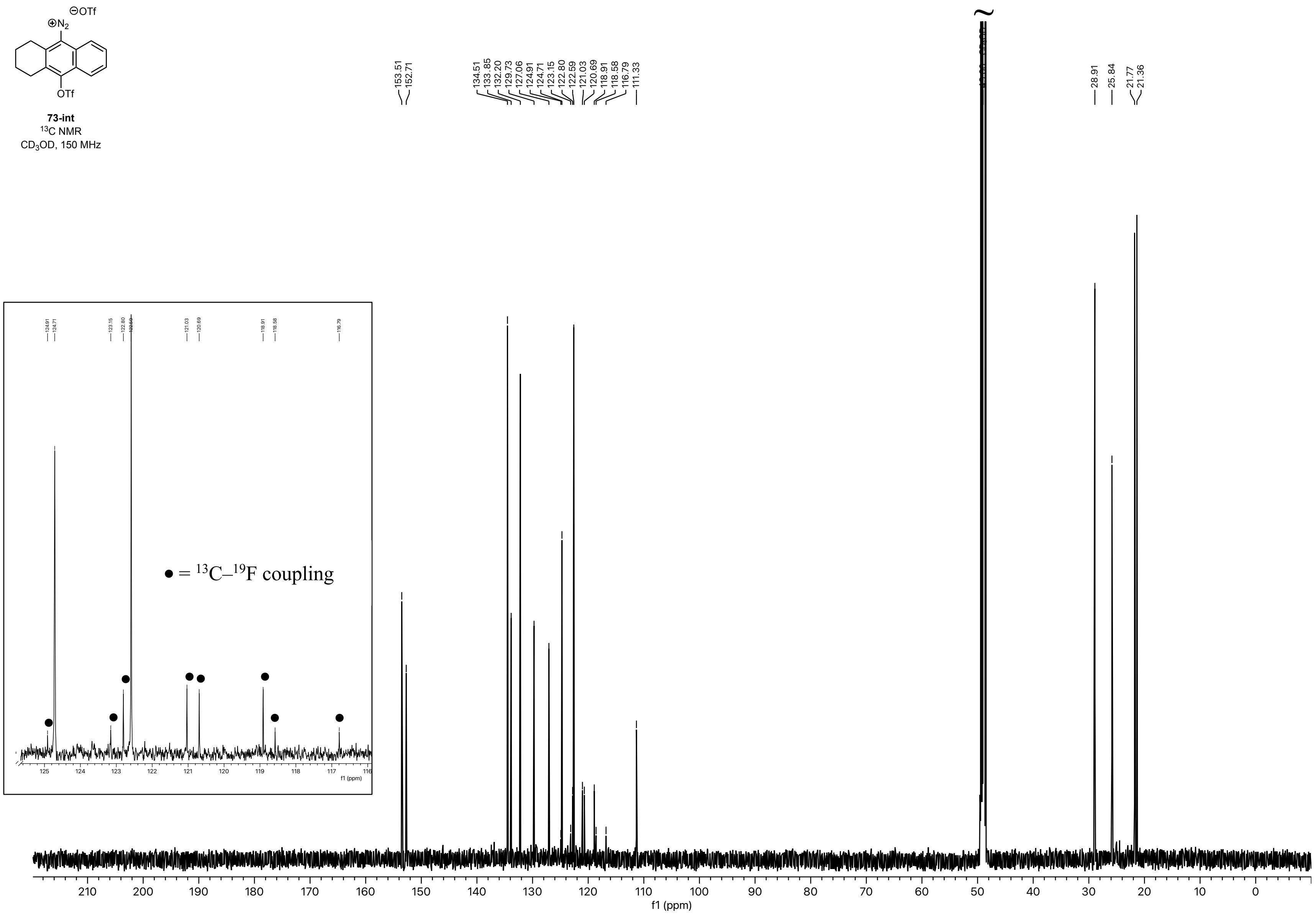

⊖OTf
⊕N2
OTf
73-int
13C NMR
CD3OD, 150 MHz
153.51
152.71
134.51
133.85
132.20
129.73
127.06
124.91
124.71
123.15
122.80
122.59
121.03
120.69
118.91
118.58
116.79
111.33
28.91
25.84
21.77
21.36
● = 13C–19F coupling
f1 (ppm)

73-int
^{19}F NMR
CD_3OD, 377 MHz

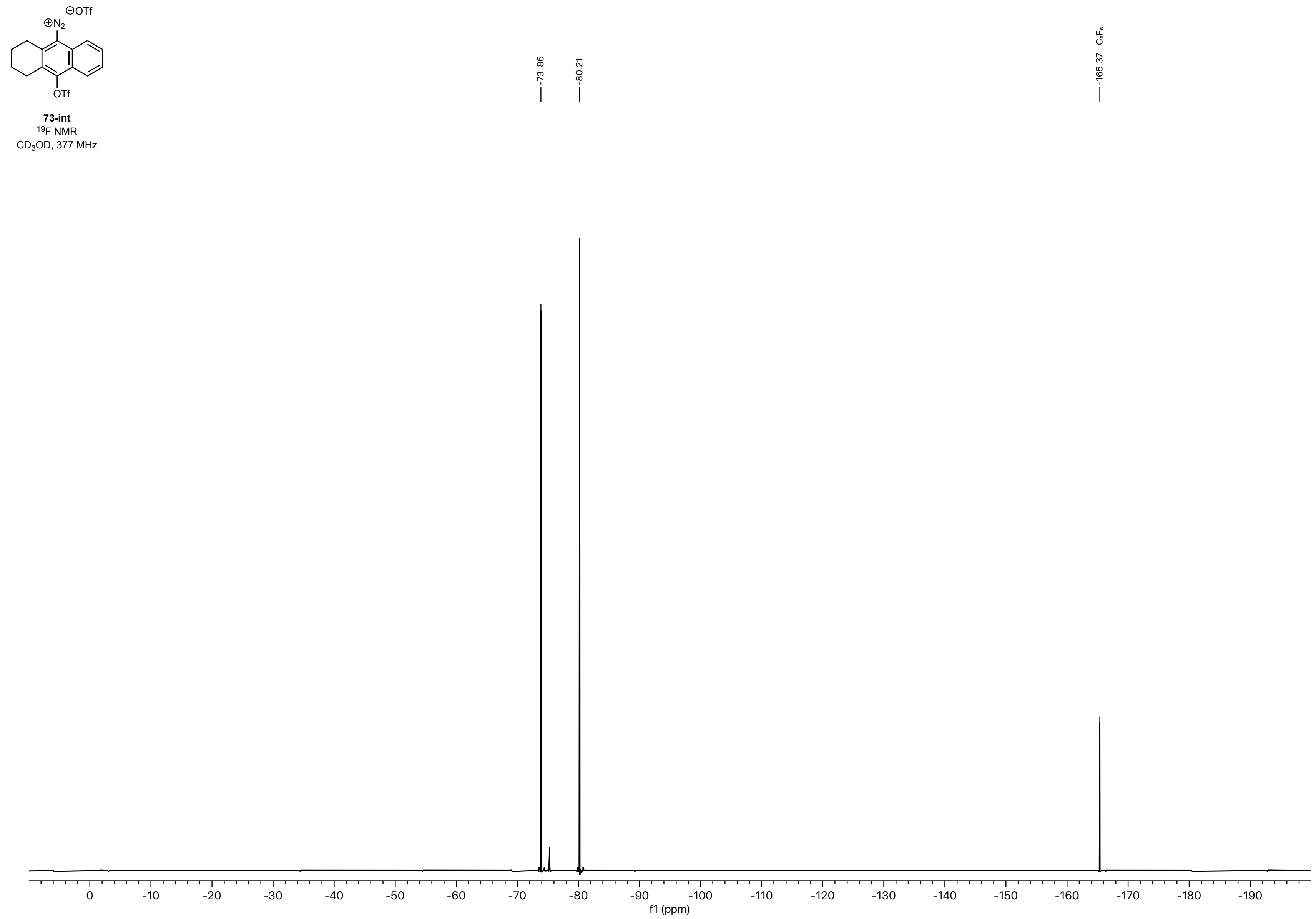

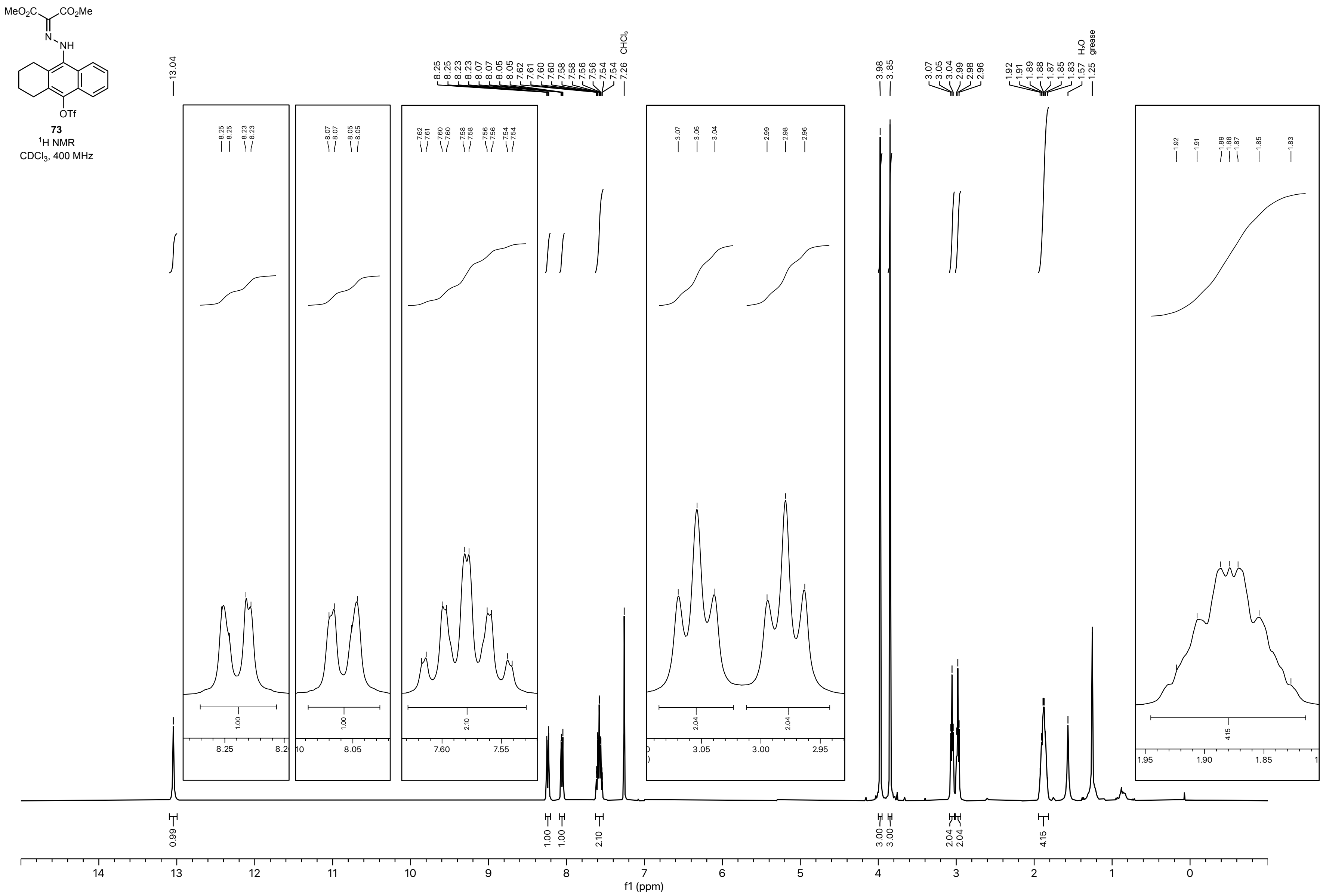

MeO2C CO2Me
N NH
OTf
73
1H NMR
CDCl3, 400 MHz
13.04
8.25 8.25 8.23 8.23 8.07 8.07 8.05 8.05 7.62 7.61 7.60 7.60 7.58 7.58 7.56 7.56 7.54 7.54 7.26
CHCl3
3.98 3.85
3.07 3.05 3.04 2.99 2.98 2.96
1.92 1.91 1.89 1.88 1.87 1.85 1.83 1.57 1.25
H2O
grease
0.99 1.00 1.00 2.10 3.00 3.00 2.04 2.04 4.15
f1 (ppm)

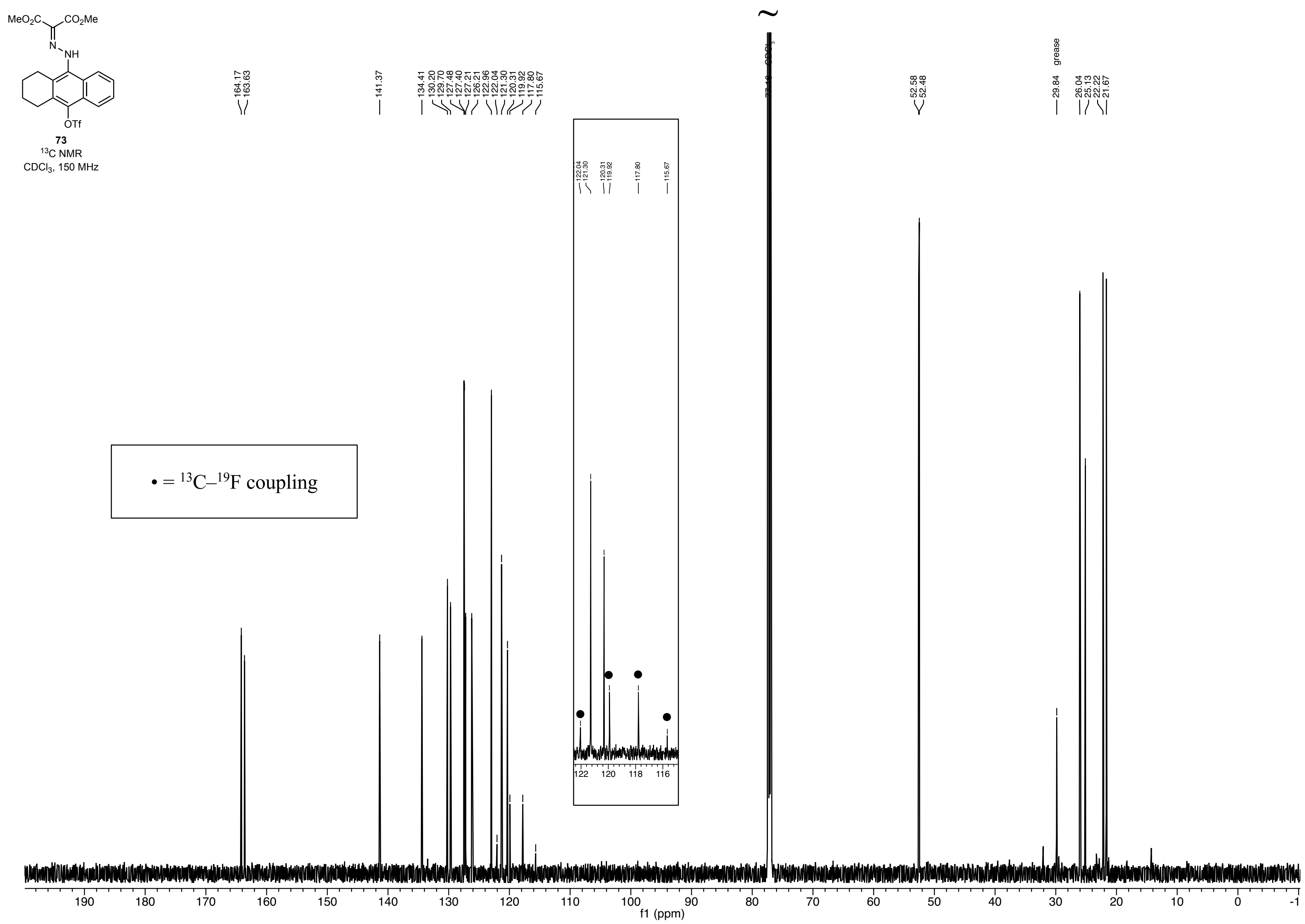

73
^{13}C NMR
$CDCl_3$, 150 MHz
164.17
163.63
141.37
134.41
130.20
129.70
127.48
127.40
127.21
126.21
122.96
122.04
121.30
120.31
119.92
117.80
115.67
$CDCl_3$
52.58
52.48
grease
29.84
26.04
25.13
22.22
21.67
• = ^{13}C–^{19}F coupling
f1 (ppm)

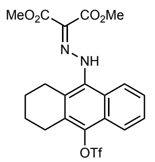

73
^{19}F NMR
$CDCl_3$, 377 MHz

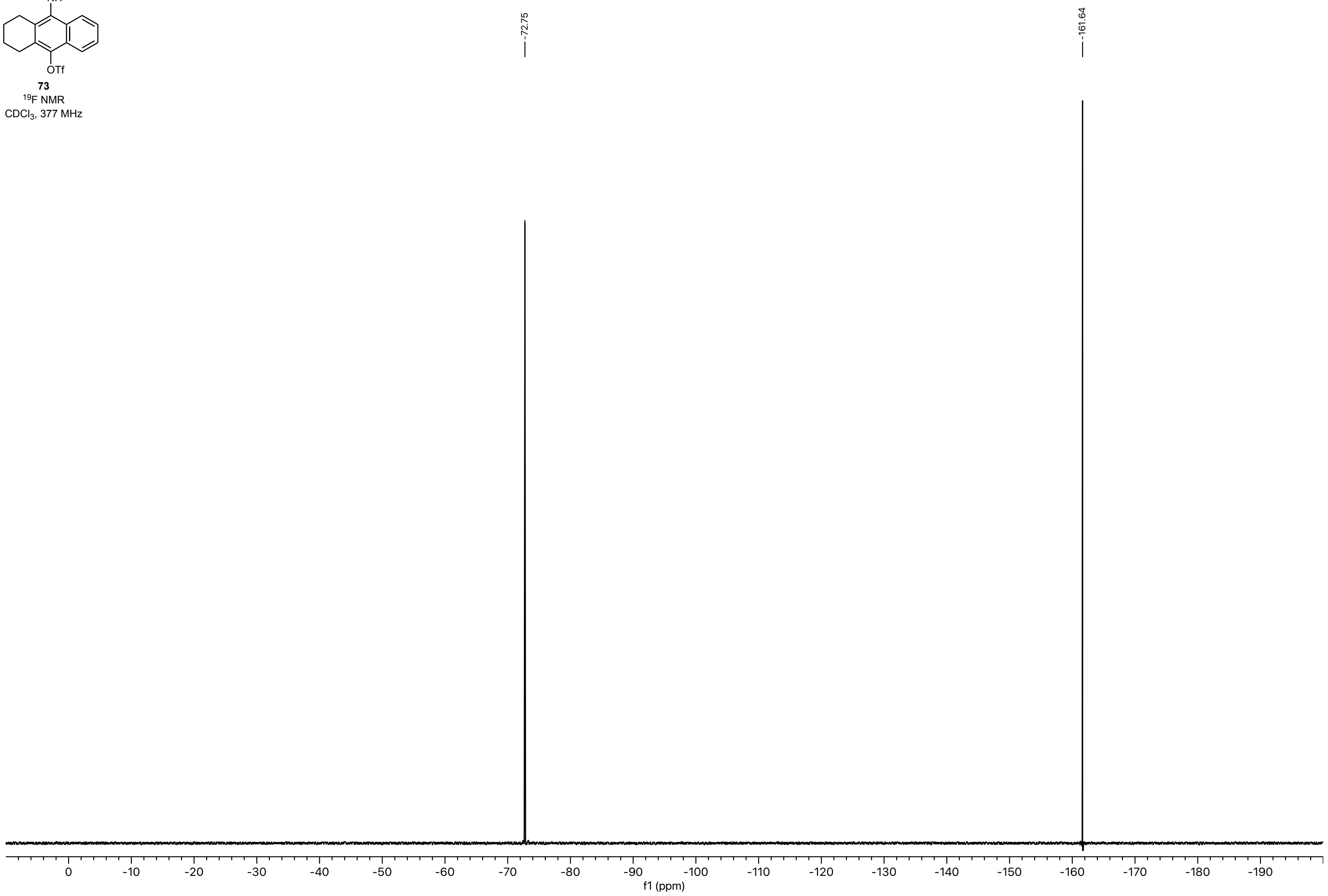

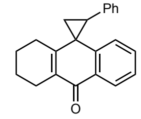

74
^{1}H NMR
CD_2Cl_2, 400 MHz

characterized as a 1:09
mixture of diastereoisomers

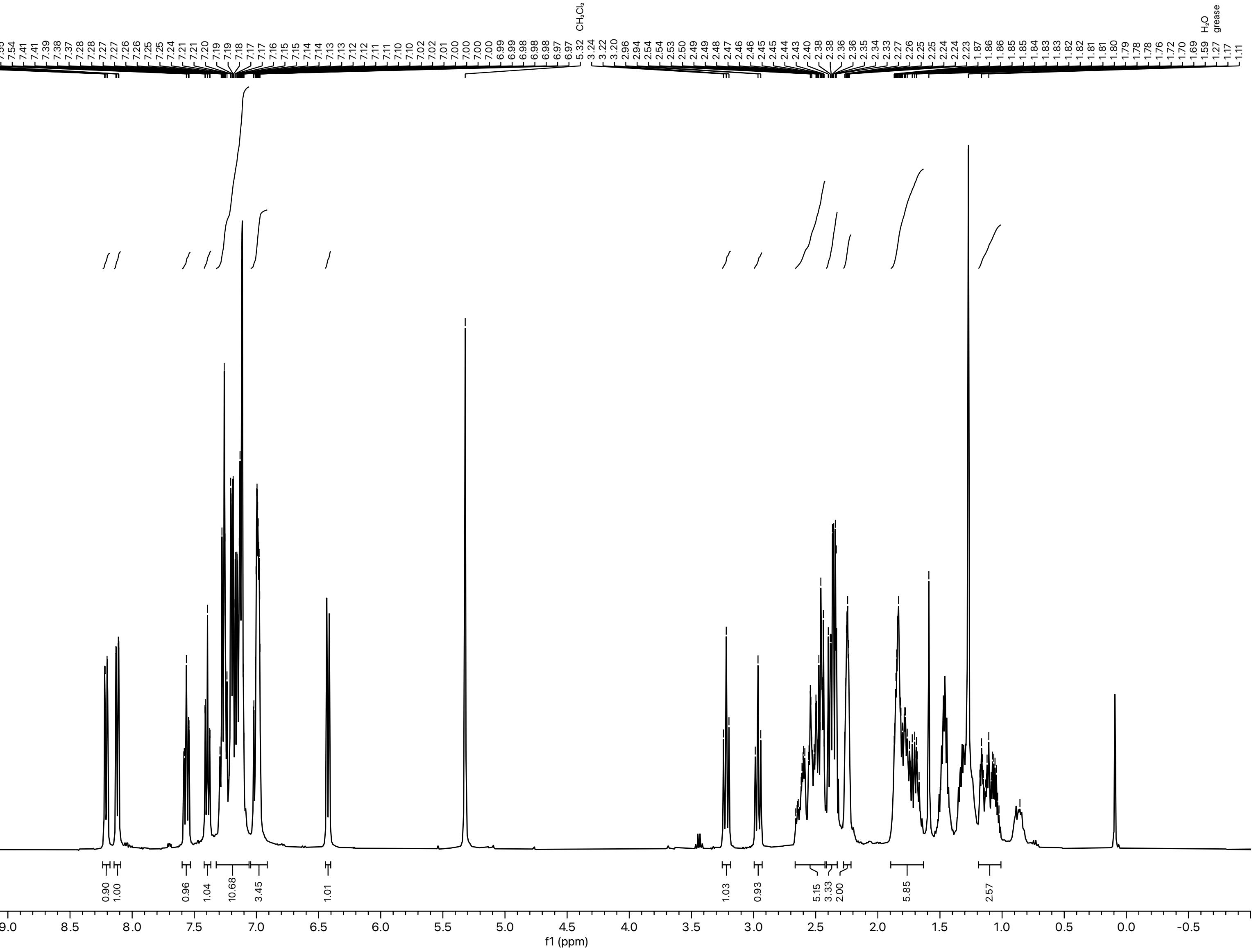

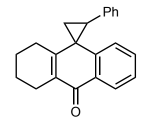

74
^{1}H NMR
CD_2Cl_2, 400 MHz

characterized as a 1:09 mixture of diastereoisomers

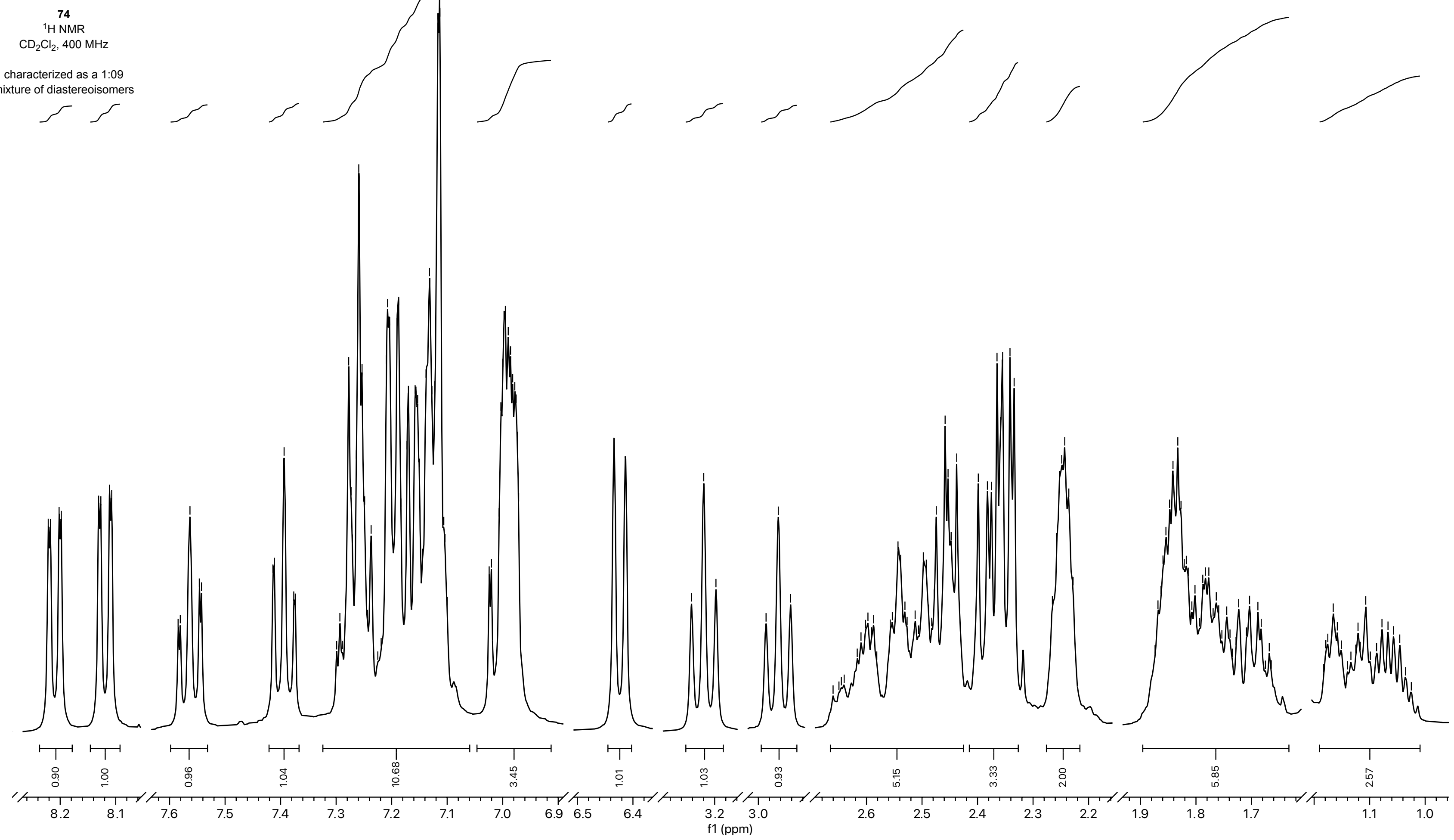

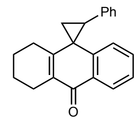

74

^{13}C NMR

CD_2Cl_2, 100 MHz

characterized as a 1:09 mixture of diastereoisomers

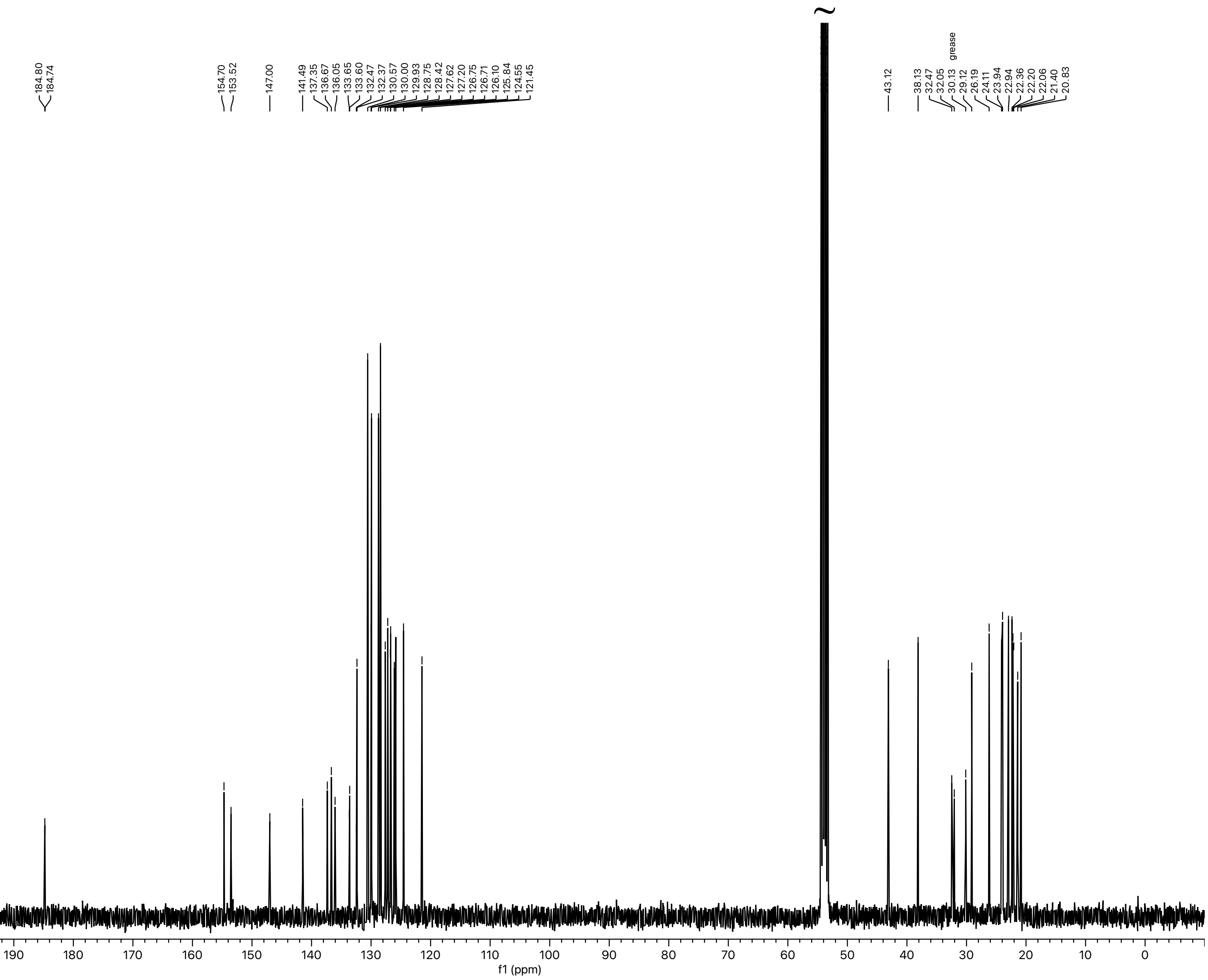

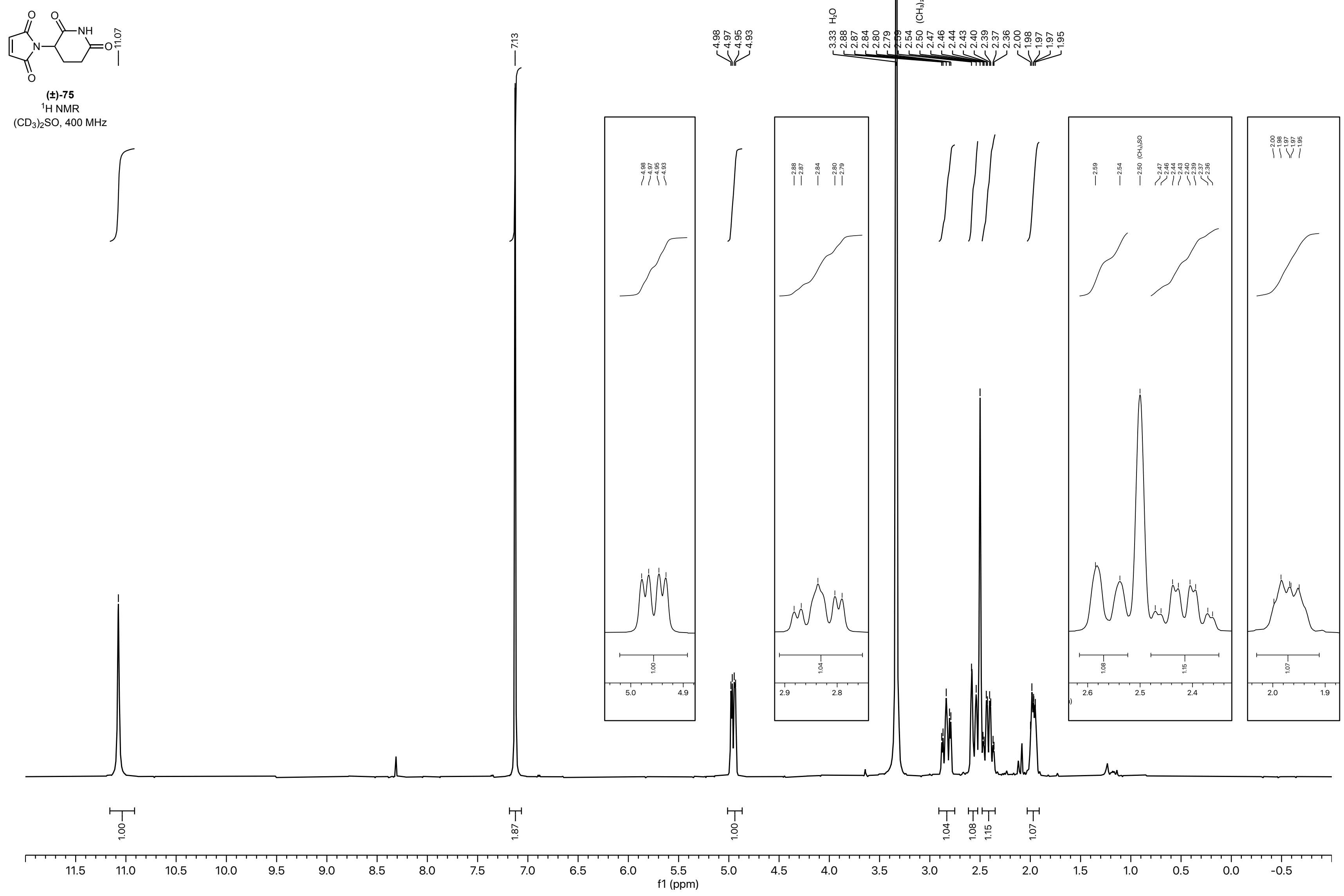

(±)-75
¹H NMR
(CD₃)₂SO, 400 MHz
11.07
7.13
4.98 4.97 4.95 4.93
3.33 H₂O
2.88 2.87 2.84 2.80 2.79 2.59 2.54 2.50 (CH₃)₂SO 2.47 2.46 2.44 2.43 2.40 2.39 2.37 2.36
2.00 1.98 1.97 1.97 1.95
1.00
1.87
1.00
1.04
1.08
1.15
1.07
f1 (ppm)

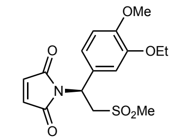

(*S*)-76
^{1}H NMR
$CDCl_3$, 400 MHz

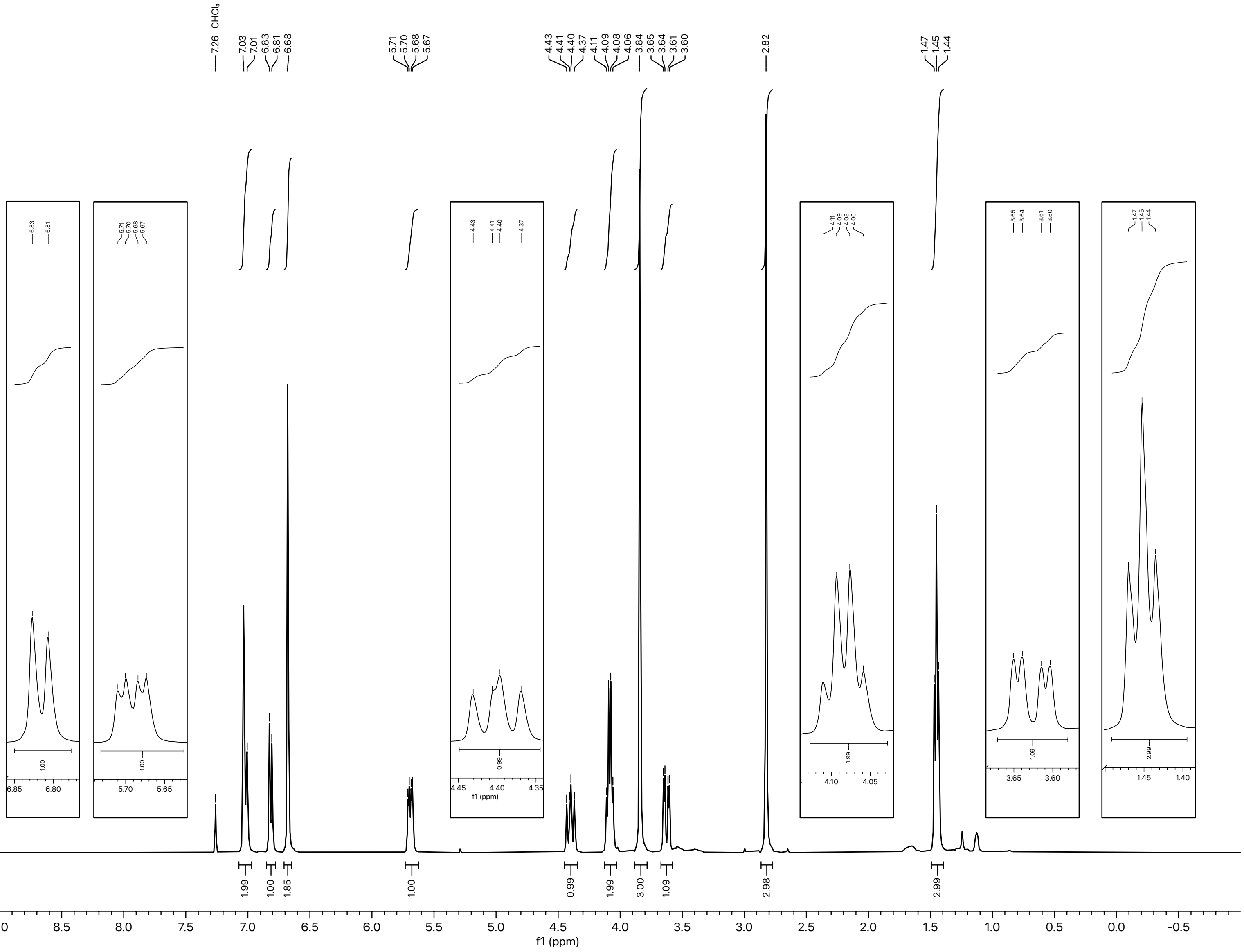

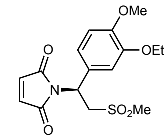

(*S*)-76
^{13}C NMR
$CDCl_3$, 100 MHz

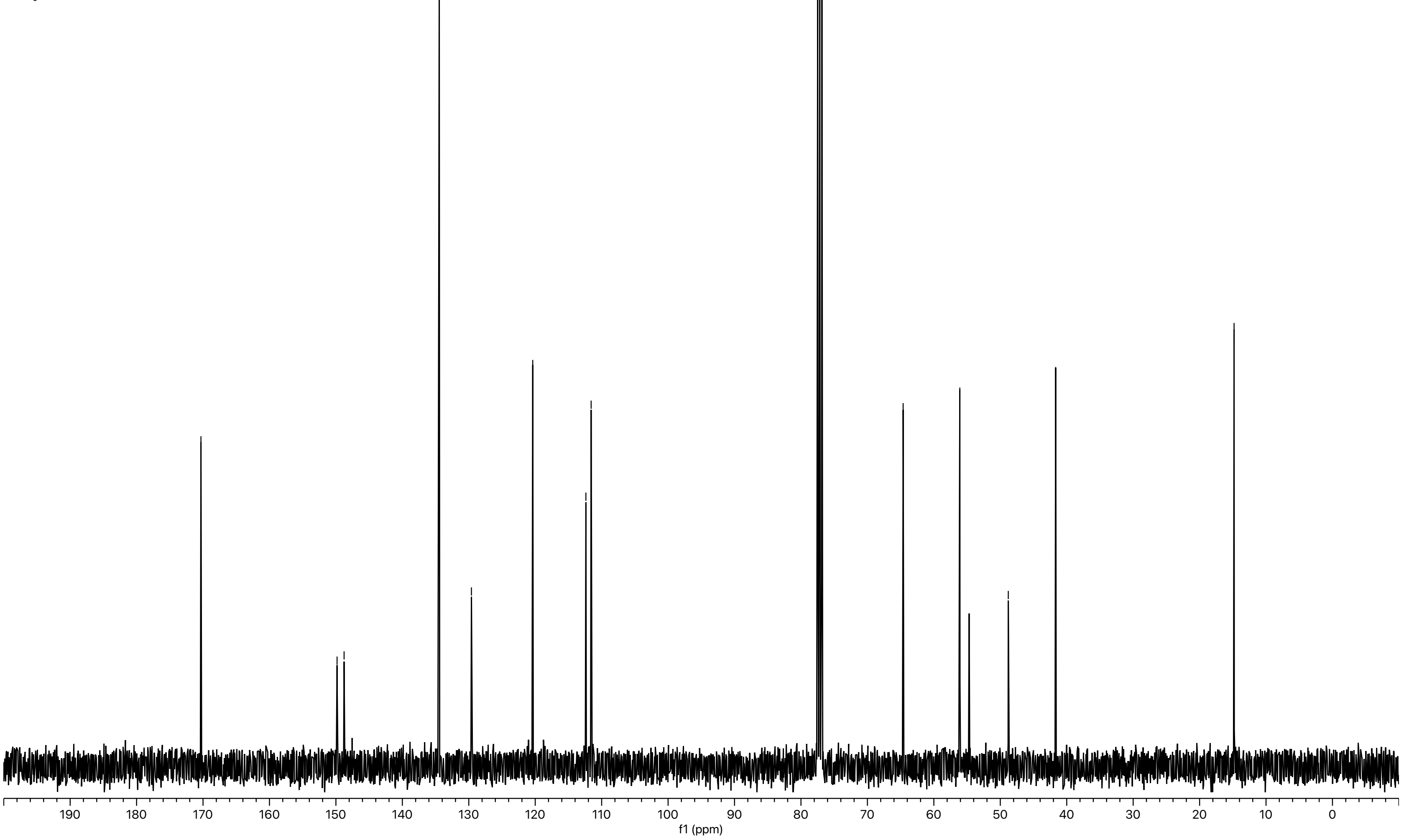

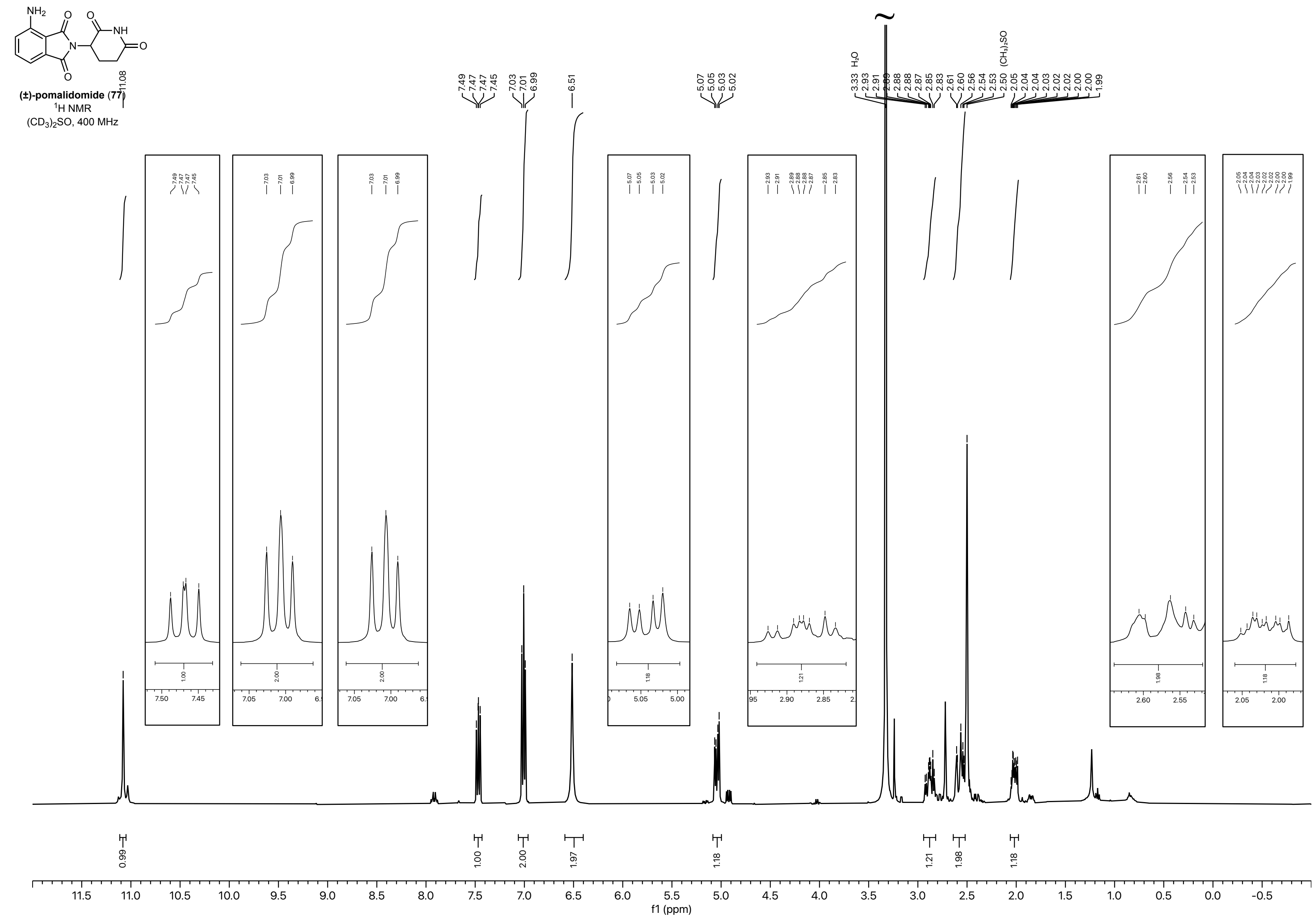

(±)-pomalidomide (77)
1H NMR
(CD3)2SO, 400 MHz
H2O
(CH3)2SO
f1 (ppm)

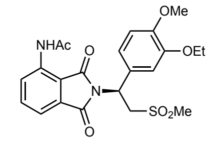

(*S*)-apremilast (78)
^{1}H NMR
$CDCl_3$, 400 MHz

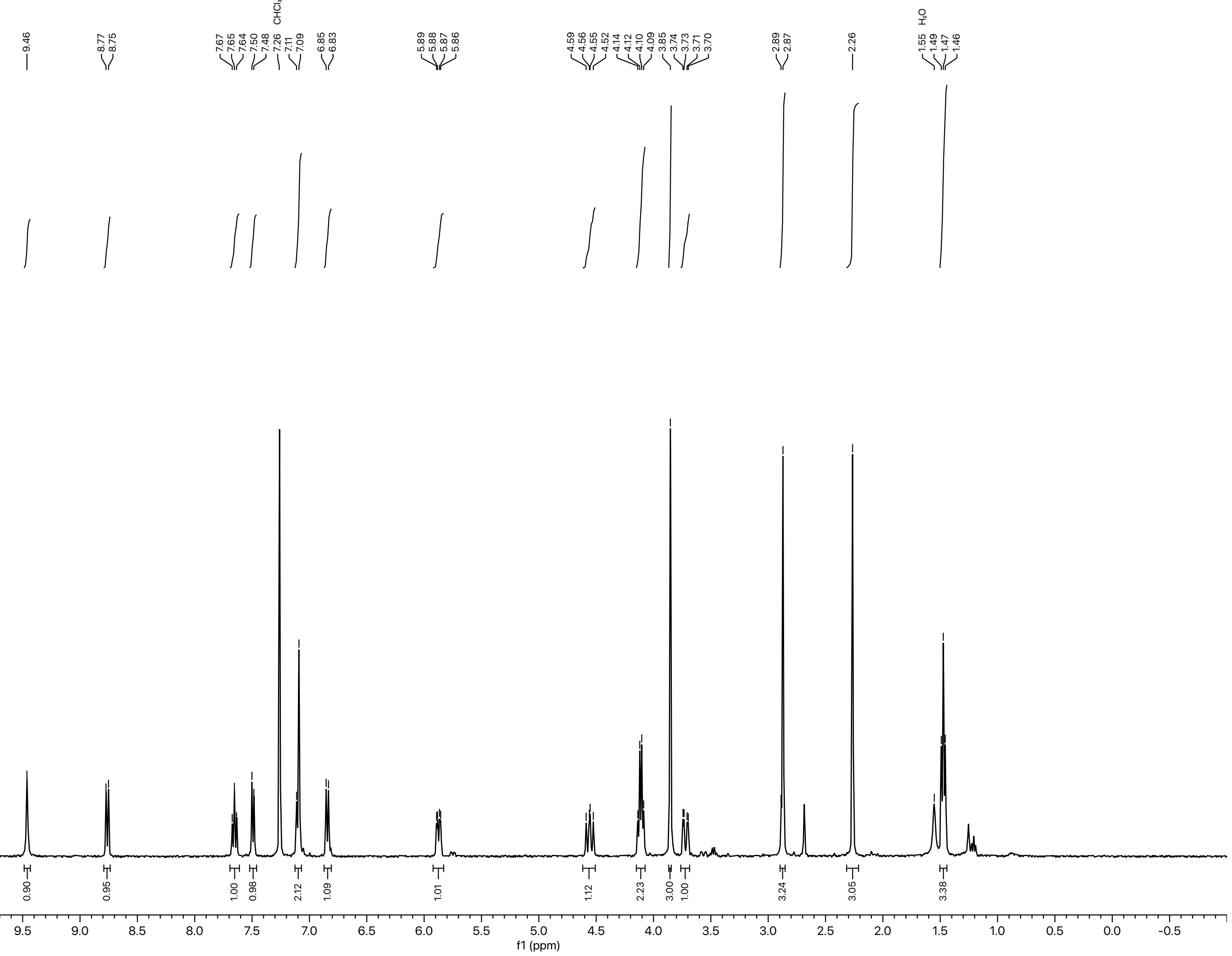

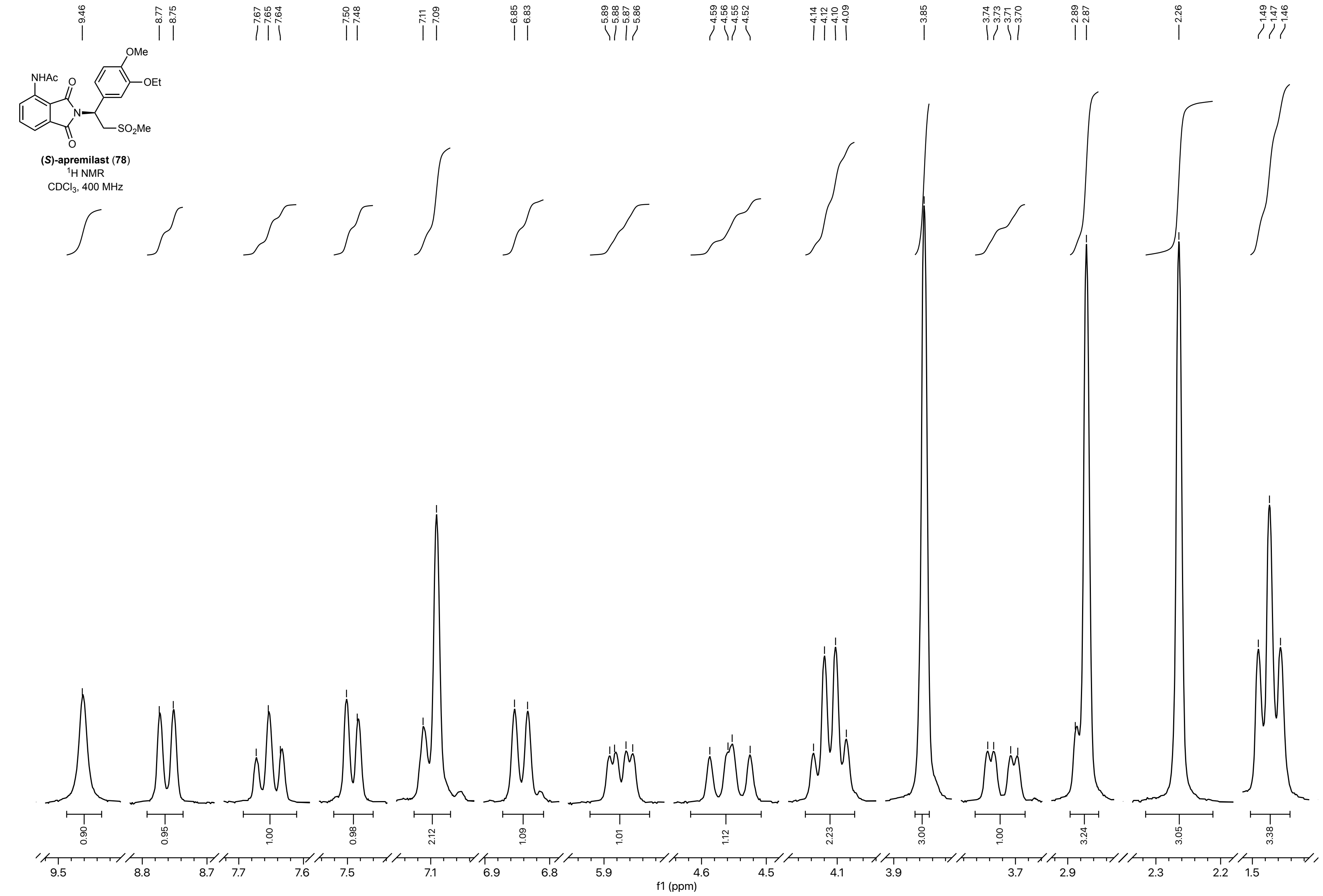

NHAc
OMe
OEt
N
SO2Me
O
O
(S)-apremilast (78)
1H NMR
CDCl3, 400 MHz
9.46
8.77
8.75
7.67
7.65
7.64
7.50
7.48
7.11
7.09
6.85
6.83
5.89
5.88
5.87
5.86
4.59
4.56
4.55
4.52
4.14
4.12
4.10
4.09
3.85
3.74
3.73
3.71
3.70
2.89
2.87
2.26
1.49
1.47
1.46
0.90
0.95
1.00
0.98
2.12
1.09
1.01
1.12
2.23
3.00
1.00
3.24
3.05
3.38
9.5
8.8
8.7
7.7
7.6
7.5
7.1
6.9
6.8
5.9
4.6
4.5
4.1
3.9
3.7
2.9
2.3
2.2
1.5
f1 (ppm)

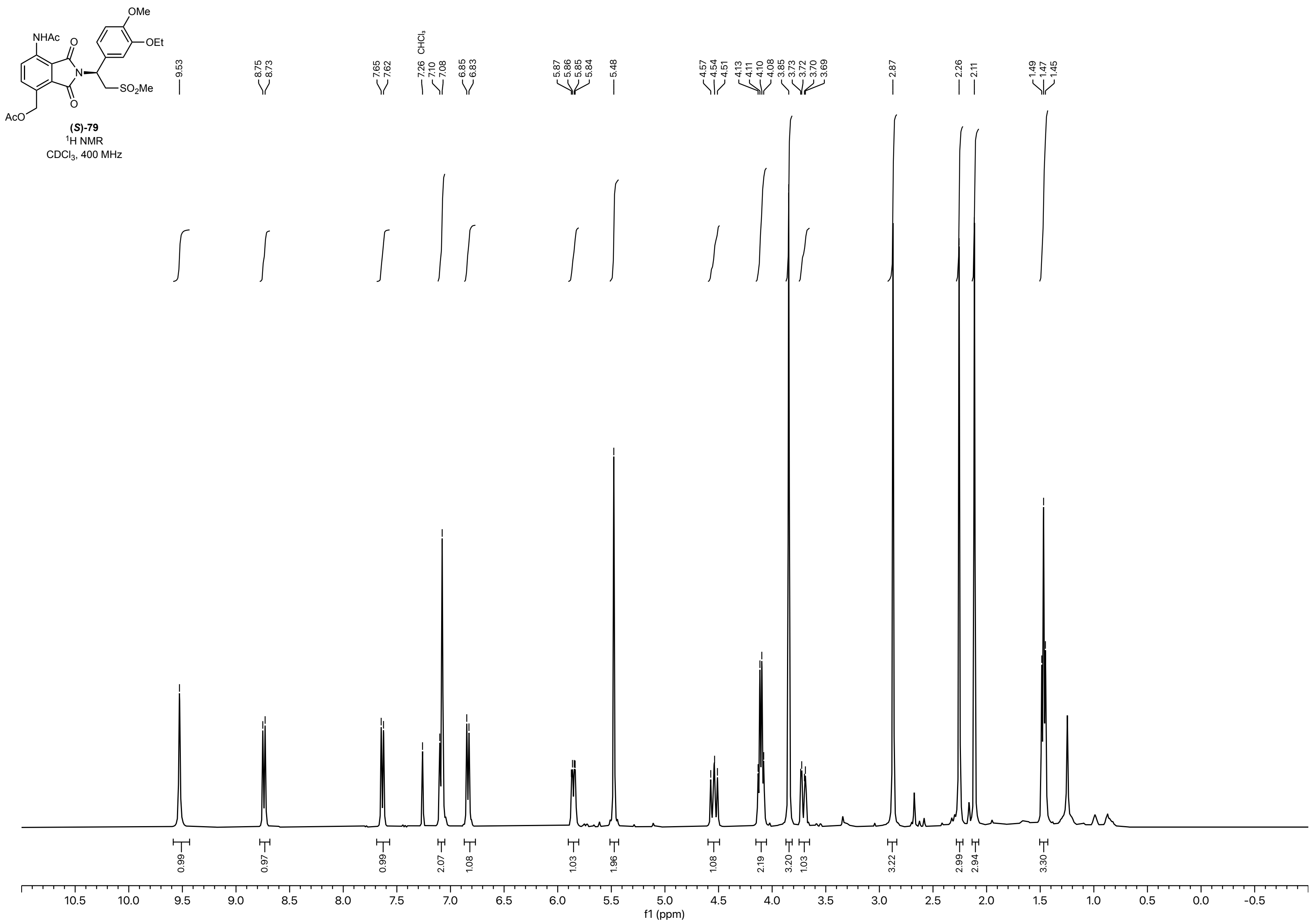
(S)-79
1H NMR
CDCl3, 400 MHz
9.53
8.75
8.73
7.65
7.62
7.26 CHCl3
7.10
7.08
6.85
6.83
5.87
5.86
5.85
5.84
5.48
4.57
4.54
4.51
4.13
4.11
4.10
4.08
3.85
3.73
3.72
3.70
3.69
2.87
2.26
2.11
1.49
1.47
1.45
0.99
0.97
0.99
2.07
1.08
1.03
1.96
1.08
2.19
3.20
1.03
3.22
2.99
2.94
3.30
10.5
10.0
9.5
9.0
8.5
8.0
7.5
7.0
6.5
6.0
5.5
5.0
4.5
4.0
3.5
3.0
2.5
2.0
1.5
1.0
0.5
0.0
-0.5
f1 (ppm)

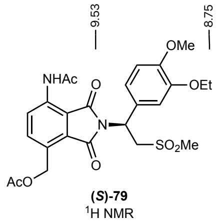

(*S*)-79
^{1}H NMR
$CDCl_3$, 400 MHz

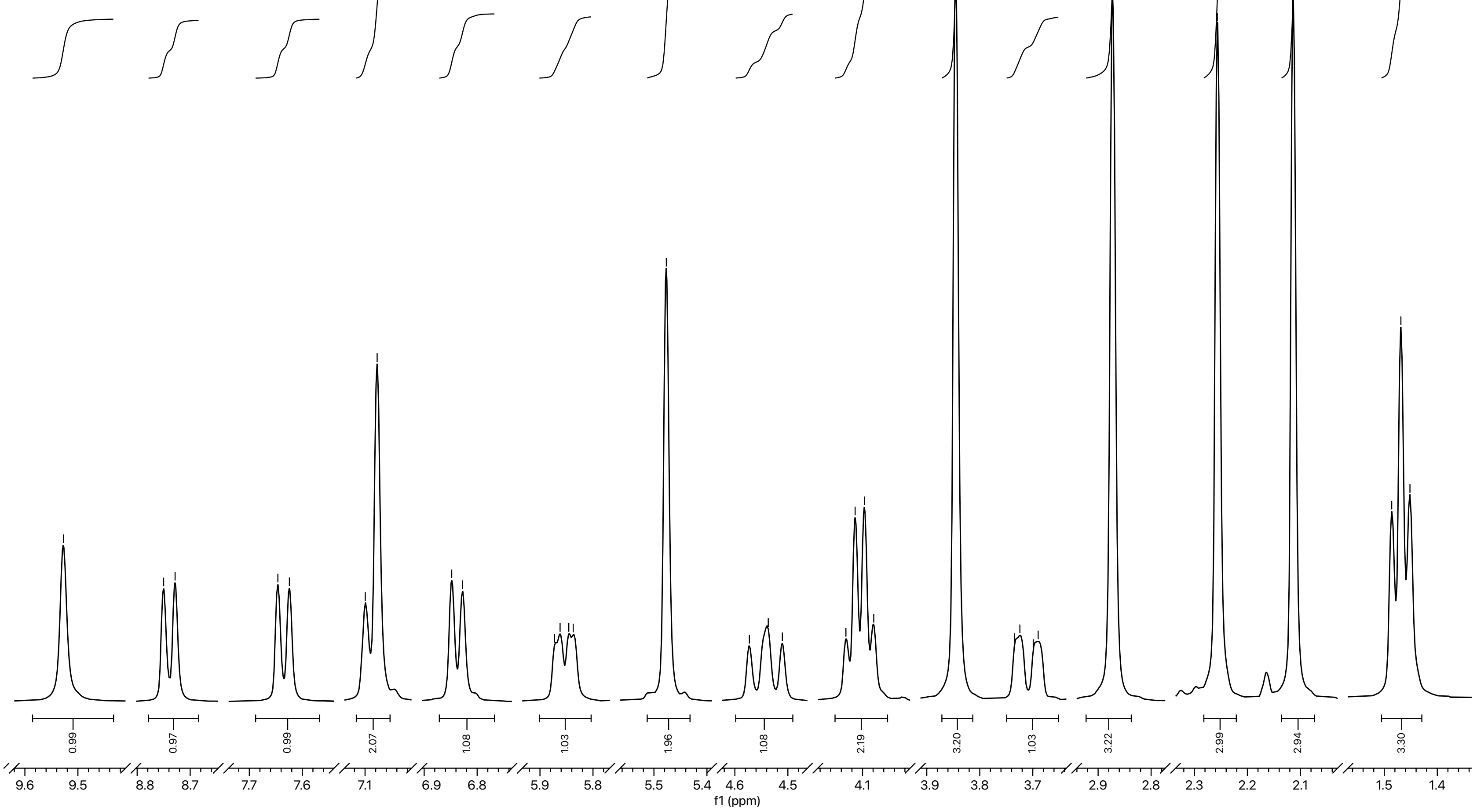

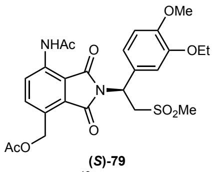

(*S*)-79
^{13}C NMR
$CDCl_3$, 100 MHz

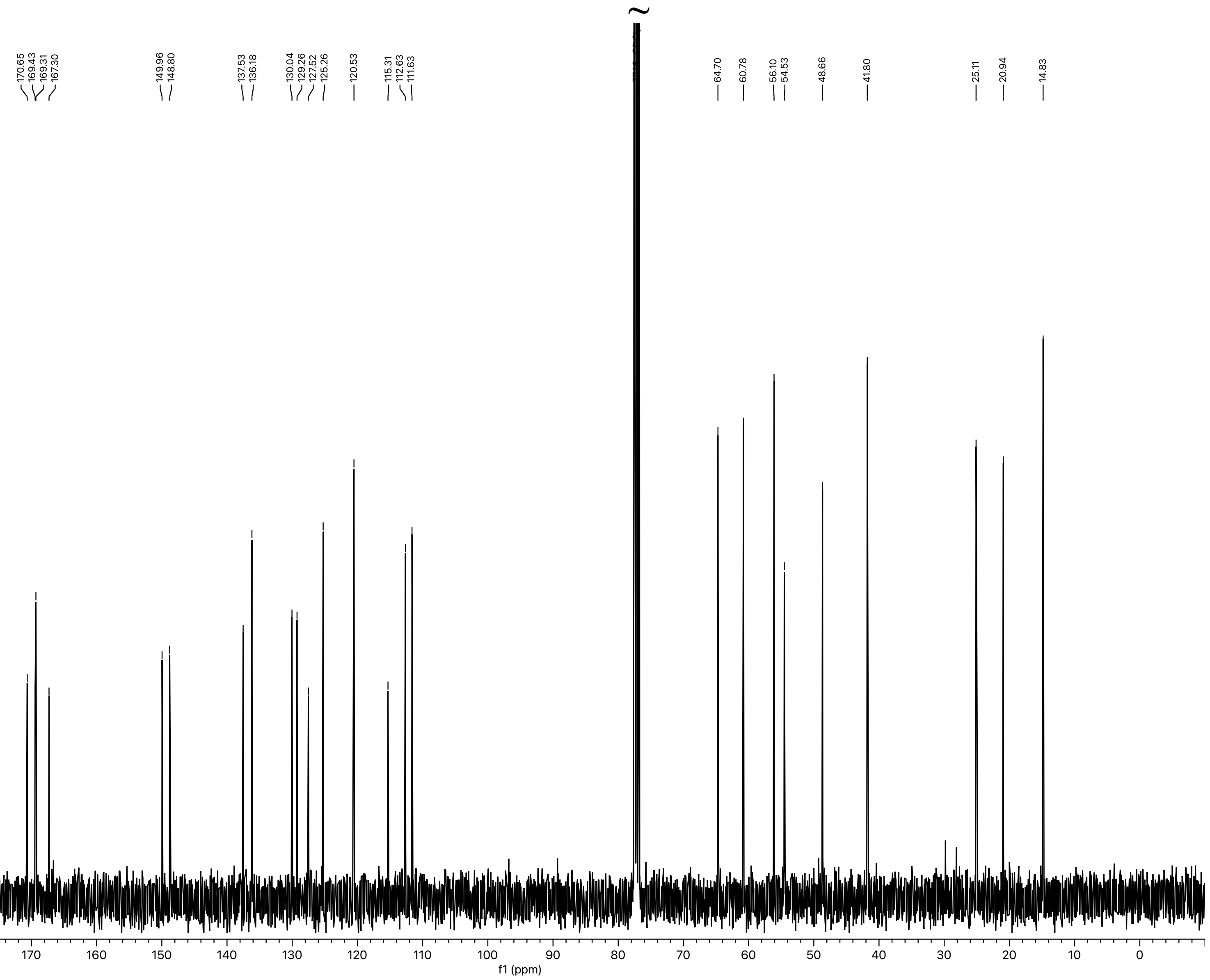

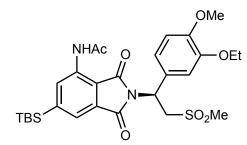

(*S*)-80
^{1}H NMR
$CDCl_3$, 400 MHz

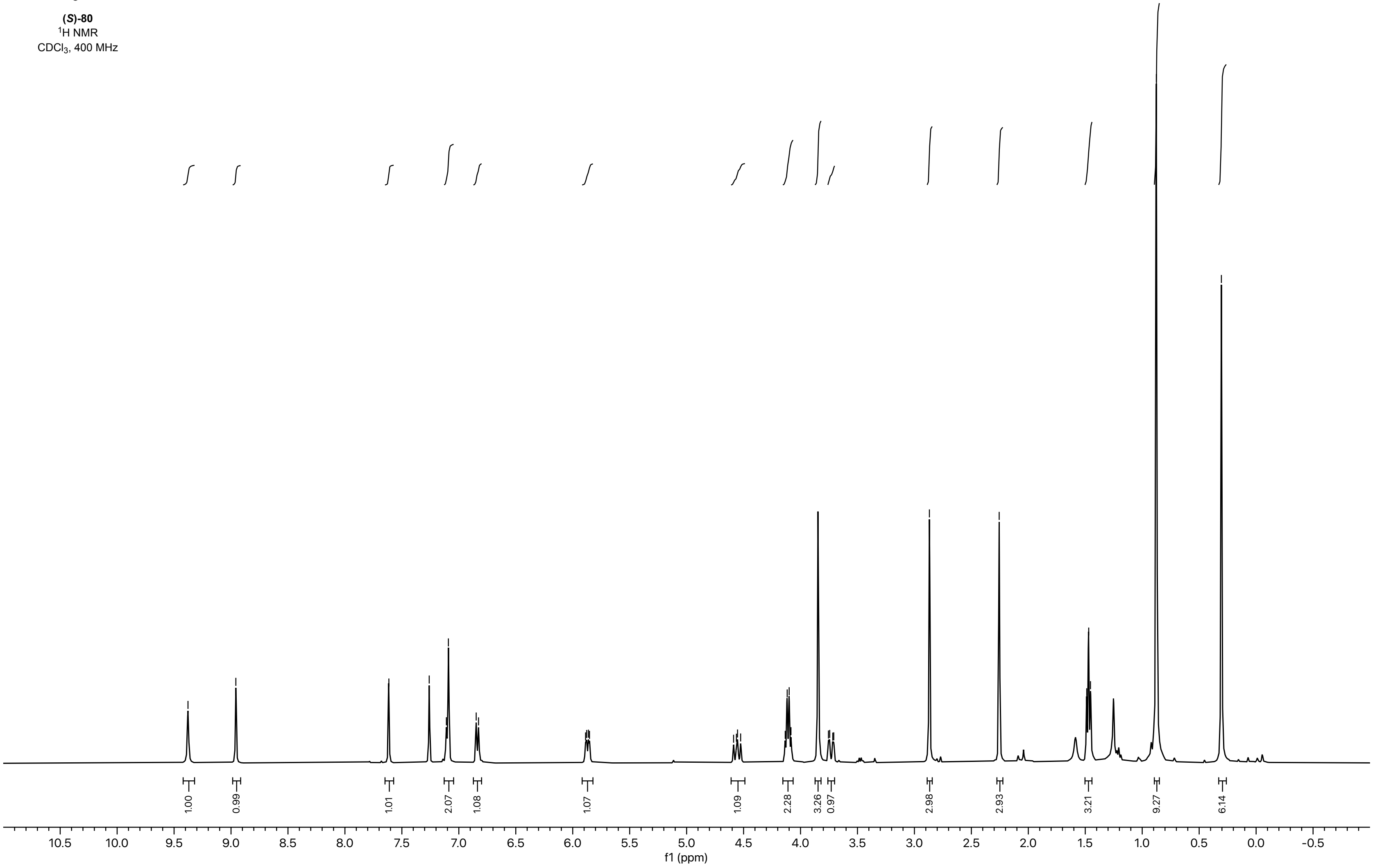

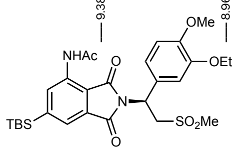

(*S*)-80
^{1}H NMR
$CDCl_3$, 400 MHz

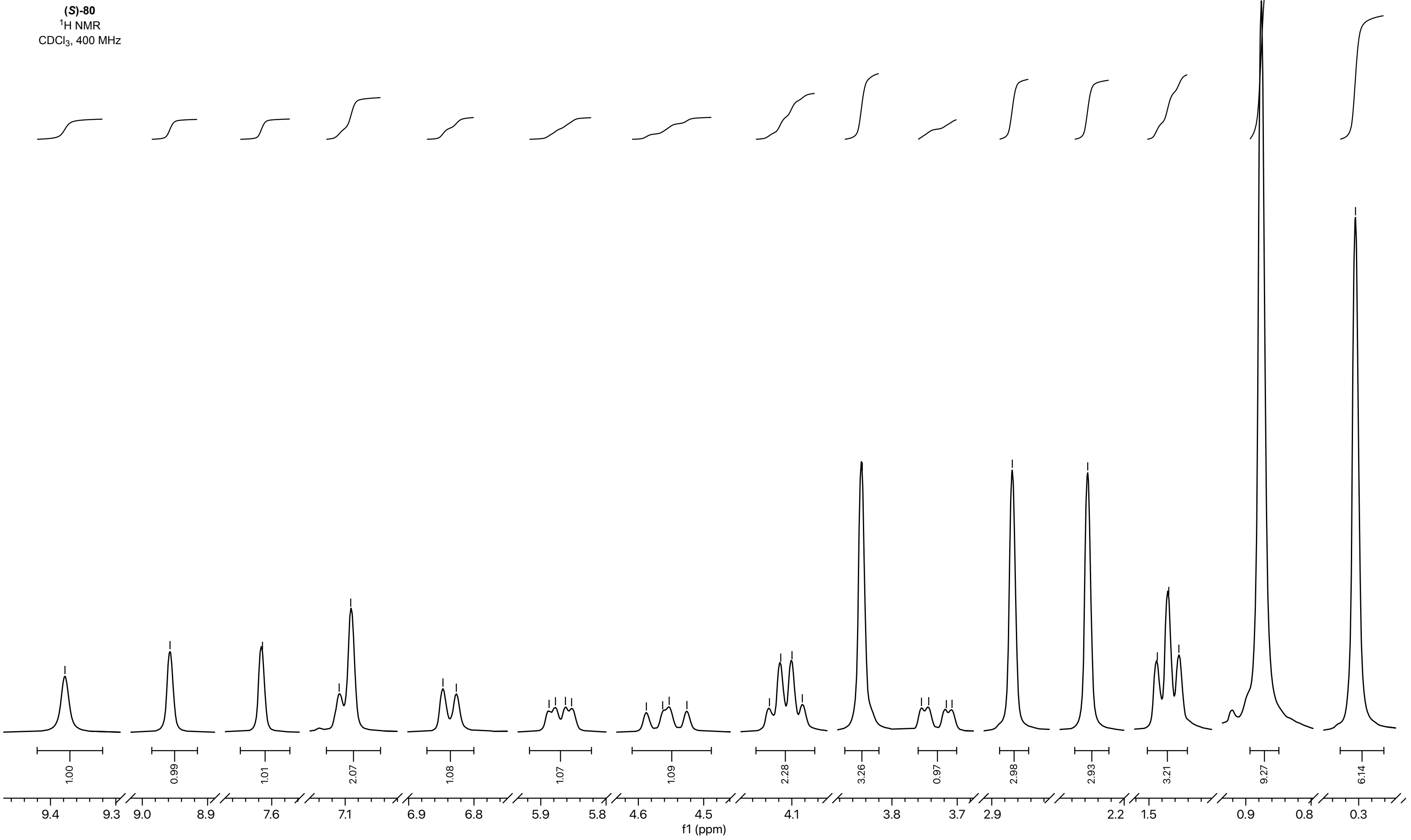

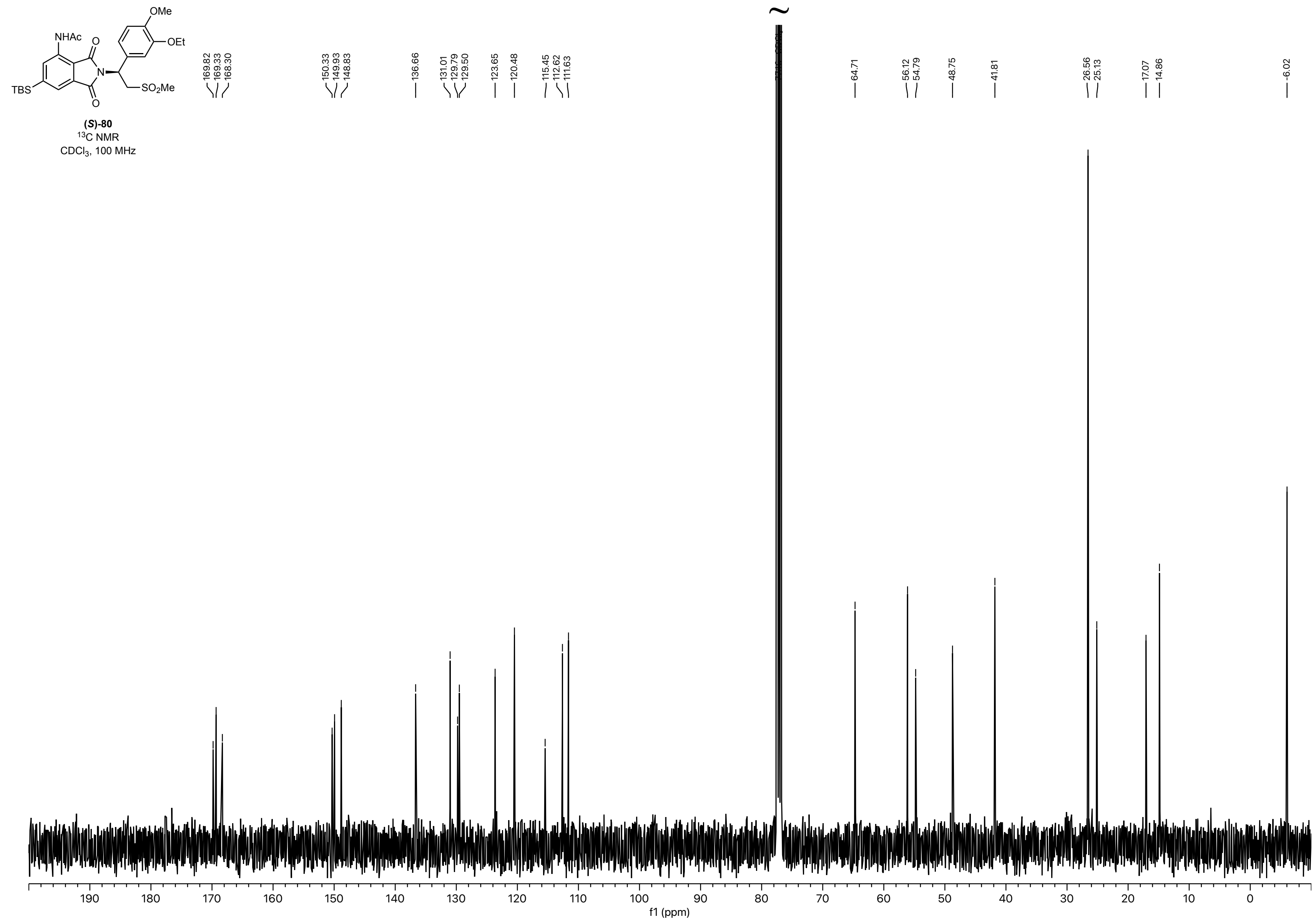

(*S*)-80
^{13}C NMR
$CDCl_3$, 100 MHz

References

(1) (a) Tertov, B. A.; Nazarova, Z. N.; Gararaeva, Y. A.; Shibaeva, N. V. *Zh. Org. Khim.* **1971**, *7*, 1062. (b) Nakhai, A.; Stensland, B.; Svensson, P. H.; Bergman, J. *Eur. J. Org. Chem.* **2010**, 6588. DOI: 10.1002/ejoc.201000328. (c) Anchi, A.; Malunavar, S. S.; Naik, R. S.; Kalkhambkar, R. G.; Laali, K. K. *Eur. J. Org. Chem.* **2022**, e202201210. DOI: 10.1002/ejoc.202201210. (d) Anchi, A.; Kalkhambkar, R. G.; Kumar P, P.; Naik, R. S.; Refat, M. S.; Adam, A. M. A.; Alsuhaibani, A. M.; Bakare, S. B. *ChemistrySelect* **2023**, *8*, e202300871. DOI: 10.1002/slct.202300871.

(2) (a) Lythgoe, D. J.; McClenaghan, I.; Ramsden, C. A. *J. Heterocycl. Chem.* **1993**, *30*, 113. DOI: 10.1002/jhet.5570300120. (b) Hooper, M. W.; Utsunomiya, M.; Hartwig, J. F. *J. Org. Chem.* **2003**, *68*. DOI: 10.1021/jo0266339.

(3) (a) Campbell, M. M.; Kaye, A. D.; Sainsbury, M. *Tetrahedron* **1982**, *38*, 2783. DOI: 10.1016/0040-4020(82)85004-7. (b) Lingham, A. R.; Rook, T. J.; Hügel, H. M. *Aust. J. Chem.* **2003**, *55*, 795. DOI: 10.1071/ch02178.

(4) (a) Bobošíková, M.; Clegg, W.; Coles, S. J.; Dandárová, M.; Hursthouse, M. B.; Kiss, T.; Krutošíková, A.; Liptaj, T.; Prónayová, N. a.; Ramsden, C. A. *J. Chem. Soc., Perkin Trans. 1* **2001**, 680. DOI: 10.1039/b010010m. (b) Padwa, A.; Dimitroff, M.; Waterson, A. G.; Wu, T. *J. Org. Chem.* **1997**, *62*, 4088. DOI: 10.1021/jo9702599.

(5) Rosenau, C. P.; Jelier, B. J.; Gossert, A. D.; Togni, A. *Angew. Chem., Int. Ed.* **2018**, *57*, 9528. DOI: 10.1002/anie.201802620.

(6) Budwitz, J. E.; Newton, C. G. *Org. Synth.* **2023**, *100*, 159. DOI: 10.15227/orgsyn.100.0159.

(7) (a) Bruker AXS Inc. APEX3 Crystallography Software Suite: 2016, 5465 East Cheryl Parkway, Madison, WI 53711, USA, . (b) Sheldrick, G. M. *Acta Crystallogr. A* **2007**, *64*, 112. DOI: 10.1107/S0108767307043930. (c) Sheldrick, G. M. *Acta Crystallogr. C* **2015**, *71*, 3. DOI: 10.1107/S2053229614024218.

(8) Alcarranza, M.; Villegas, I.; Muñoz-García, R.; Recio, R.; Fernández, I.; Alarcón-de-la-Lastra, C. *Pharmaceuticals* **2022**, *15*, 966. DOI: 10.3390/ph15080966.

(9) Suleymanov, A. A.; Scopelliti, R.; Fadaei Tirani, F.; Severin, K. *Org. Lett.* **2018**, *20*, 3323. DOI: 10.1021/acs.orglett.8b01214.

(10) Yao, L.; Smith, B. T.; Aubé, J. *J. Org. Chem.* **2004**, *69*, 1720. DOI: 10.1021/jo0356098.

(11) Chang, J. W.; Montgomery, J. E.; Lee, G.; Moellering, R. E. *Angew. Chem., Int. Ed.* **2018**, *57*, 15712. DOI: 10.1002/anie.201809059.

(12) Yamamoto, K.; Bruun, T.; Kim, J. Y.; Zhang, L.; Lautens, M. *Org. Lett.* **2016**, *18*, 2644. DOI: 10.1021/acs.orglett.6b00975.

(13) Peng, L.; Zhao, Y.; Okuda, Y.; Le, L.; Tang, Z.; Yin, S.-F.; Qiu, R.; Orita, A. *J. Org. Chem.* **2023**, *88*, 3089. DOI: 10.1021/acs.joc.2c02876.

(14) Li, Y. S.; Liang, C. F. *J. Chin. Chem. Soc.* **2022**, *69*, 849. DOI: 10.1002/jccs.202200018.

(15) Smith, J. R. H.; Michejda, C. J. *Synthesis* **1983**, 476. DOI: 10.1055/s-1983-30389.

(16) Kofron, W. G.; Baclawski, L. M. *J. Org. Chem.* **1976**, *41*, 1879. DOI: 10.1021/jo00872a047.

(17) Vedejs, E.; Larsen, S. *Org. Synth.* **1986**, *64*, 127. DOI: 10.15227/orgsyn.064.0127.

(18) Jiao, P.; Kawasaki, M.; Yamamoto, H. *Angew. Chem., Int. Ed.* **2009**, *48*, 3333. DOI: 10.1002/anie.200900682.

(19) Love, B. E.; Jones, E. G. *J. Org. Chem.* **1999**, *64*, 3755. DOI: 10.1021/jo982433e.

(20) Kiefer, G.; Riedel, T.; Dyson, P. J.; Scopelliti, R.; Severin, K. *Angew. Chem., Int. Ed.* **2014**, *54*, 302. DOI: 10.1002/anie.201408597.

(21) Dota, K.; Shimizu, T.; Hasegawa, S.; Miyashita, M.; Tanino, K. *Tetrahedron Lett.* **2011**, *52*, 910. DOI: 10.1016/j.tetlet.2010.12.063.
(22) Platon, M.; Wijaya, N.; Rampazzi, V.; Cui, L.; Rousselin, Y.; Saeys, M.; Hierso, J. C. *Chem. Eur. J.* **2014**, *20*, 12584. DOI: 10.1002/chem.201403337.
(23) Glorius, F.; Ernst, J.; Rakers, L. *Synthesis* **2016**, *49*, 260. DOI: 10.1055/s-0036-1588609.
(24) Hadfield, M. S.; Lee, A.-L. *Chem. Commun.* **2011**, *47*, 1333. DOI: 10.1039/c0cc04217j.
(25) Ando, N.; Fukazawa, A.; Kushida, T.; Shiota, Y.; Itoyama, S.; Yoshizawa, K.; Matsui, Y.; Kuramoto, Y.; Ikeda, H.; Yamaguchi, S. *Angew. Chem., Int. Ed.* **2017**, *56*, 12210. DOI: 10.1002/anie.201706929.
(26) Guo, R.; Zhai, H.; Li, Y. *Chin. Chem. Lett.* **2021**, *32*, 1400. DOI: 10.1016/j.cclet.2020.09.023.
(27) Khatua, H.; Das, S.; Patra, S.; Das, S. K.; Roy, S.; Chattopadhyay, B. *J. Am. Chem. Soc.* **2022**, *144*, 21858. DOI: 10.1021/jacs.2c10719.
(28) Straathof, N. J. W.; Cramer, S. E.; Hessel, V.; Noël, T. *Angew. Chem., Int. Ed.* **2016**, *55*, 15549. DOI: 10.1002/anie.201608297.
(29) Wright, A. C.; Du, Y. E.; Stoltz, B. M. *J. Org. Chem.* **2019**, *84*, 11258. DOI: 10.1021/acs.joc.9b01541.
(30) Lam, K.; Markó, I. E. *Chem. Commun.* **2009**, 95. DOI: 10.1039/b813545b.
(31) Kalaitzakis, D.; Kouridaki, A.; Noutsias, D.; Montagnon, T.; Vassilikogiannakis, G. *Angew. Chem., Int. Ed.* **2015**, *54*, 6283. DOI: 10.1002/anie.201500744.
(32) Huffman, B. J.; Chen, S.; Schwarz, J. L.; Plata, R. E.; Chin, E. N.; Lairson, L. L.; Houk, K. N.; Shenvi, R. A. *Nat. Chem.* **2020**, *12*, 310. DOI: 10.1038/s41557-019-0413-8.
(33) Buu Hue, B. T.; Dijkink, J.; Kuiper, S.; van Schaik, S.; van Maarseveen, J. H.; Hiemstra, H. *Eur. J. Org. Chem.* **2006**, 127. DOI: 10.1002/ejoc.200500609.
(34) Lam, S.; Lo, B.; Wong, W. T.; Chiu, P. *Asian J. Org. Chem.* **2012**, *1*, 30. DOI: 10.1002/ajoc.201200038.
(35) Takadoi, M.; Katoh, T.; Ishiwata, A.; Terashima, S. *Tetrahedron* **2002**, *58*, 9903. DOI: 10.1016/s0040-4020(02)01358-3.
(36) Nazef, N.; Davies, R. D. M.; Greaney, M. F. *Org. Lett.* **2012**, *14*, 3720. DOI: 10.1021/ol301513h.
(37) Sutton, J.; Clark, D. E.; Higgs, C.; de Groot, M. J.; Harris, N. V.; Taylor, A.; Lockey, P. M.; Maubach, K.; Woodrooffe, A.; Davis, R. J.; Coleman, R. A.; Clark, K. L. *Bioorg. Med. Chem. Lett.* **2014**, *24*, 2212. DOI: 10.1016/j.bmcl.2014.02.068.
(38) Jung, M. E.; Nichols, C. J. *J. Org. Chem.* **1998**, *63*, 347. DOI: 10.1021/jo971890c.
(39) Holland, H. L.; Turner, C. D.; Andreana, P. R.; Nguyen, D. *Can. J. Chem.* **1999**, *77*, 463. DOI: 10.1139/v99-068.
(40) Semple, J. E.; Wang, P. C.; Lysenko, Z.; Joullie, M. M. *J. Am. Chem. Soc.* **1980**, *102*, 7505. DOI: 10.1021/ja00545a018.
(41) Murali, R.; Surya Prakash Rao, H.; Scheeren, H. W. *Tetrahedron* **2001**, *57*, 3165. DOI: 10.1016/s0040-4020(01)00175-2.
(42) Warnica, J. M.; Gleason, J. L. *Chem. Commun.* **2023**, *59*, 10496. DOI: 10.1039/d3cc03351a.
(43) Cabri, W.; Candiani, I.; Bedeschi, A.; Santi, R. *J. Org. Chem.* **1993**, *58*, 7421. DOI: 10.1021/jo00078a021.
(44) Satyam, K.; Harish, B.; Nanubolu, J. B.; Suresh, S. *Chem. Commun.* **2020**, *56*, 2803. DOI: 10.1039/d0cc00321b.

(45) Zhang, X.-Q.; Ma, Y.-R.; Liu, Y.-K. *Org. Lett.* **2023**, *25*, 8220. DOI: 10.1021/acs.orglett.3c03182.
(46) Firth, J. D.; Craven, P. G. E.; Lilburn, M.; Pahl, A.; Marsden, S. P.; Nelson, A. *Chem. Commun.* **2016**, *52*, 9837. DOI: 10.1039/c6cc04662b.
(47) Burde, A. S.; Chemler, S. R. *ACS Catal.* **2022**, *12*, 7559. DOI: 10.1021/acscatal.2c02214.
(48) Yang, J.; Liu, S.; Zheng, J. F.; Zhou, J. *Eur. J. Org. Chem.* **2012**, *2012*, 6248. DOI: 10.1002/ejoc.201200918.
(49) Eberhart, A. J.; Shrives, H. J.; Álvarez, E.; Carrër, A.; Zhang, Y.; Procter, D. J. *Chem. Eur. J.* **2015**, *21*, 7428. DOI: 10.1002/chem.201406424.
(50) Nicolaou, K. C.; Daines, R. A.; Uenishi, J.; Li, W. S.; Papahatjis, D. P.; Chakraborty, T. K. *J. Am. Chem. Soc.* **1988**, *110*, 4672. DOI: 10.1021/ja00222a028.
(51) Horvath, K. L.; Magann, N. L.; Sowden, M. J.; Gardiner, M. G.; Sherburn, M. S. *J. Am. Chem. Soc.* **2019**, *141*, 19746. DOI: 10.1021/jacs.9b08885.
(52) (a) DeAngelis, C. J.; Goyal, G.; Liss, M. J.; Budwitz, J. E.; Herlihy, M. S.; Conner, A. V.; Wheeler, S. E.; Ma, P.; Li, M.; Houk, K. N.; Newton, C. G. *J. Am. Chem. Soc.* **2025**, *147*, 6087. DOI: 10.1021/jacs.4c17174. (b) Dissanayake, I.; Hart, J. D.; Becroft, E. C.; Sumby, C. J.; Newton, C. G. *J. Am. Chem. Soc.* **2020**, *142*, 13328. DOI: 10.1021/jacs.0c06306. (c) Budwitz, J. E.; Newton, C. G. *Synlett* **2026**, in press. DOI: 10.1055/a-2779-2027.
(53) Seong, C. M.; Kargbo, S. S.; Yu, C.-L.; Gibney, D.; Boyn, J.-N.; Roberts, C. C. *Nature* **2025**, *649*, 91. DOI: 10.1038/s41586-025-09830-1.
(54) (a) Obermüller, R.; Tobisch, H.; Stockhammer, L.; Waser, M. *Org. Process Res. Dev.* **2024**, *28*, 3735. DOI: 10.1021/acs.oprd.4c00296. (b) Sperry, J. B.; Azuma, M.; Stone, S. *Org. Process Res. Dev.* **2021**, *25*, 212. DOI: 10.1021/acs.oprd.0c00467.
(55) Margelefsky, E. L.; Dobson, B. C.; Chen, T.; Afanador, N. L. *Org. Process Res. Dev.* **2025**, *29*, 418. DOI: 10.1021/acs.oprd.4c00439.
(56) Dallaston, M. A.; Houston, S. D.; Williams, C. M. *Chem. Eur. J.* **2020**, *26*, 11966. DOI: 10.1002/chem.202001658.
(57) Lik, A.; Fritze, L.; Müller, L.; Helten, H. *J. Am. Chem. Soc.* **2017**, *139*, 5692. DOI: 10.1021/jacs.7b01835.
(58) Raheem, M.-A.; Nagireddy, J. R.; Durham, R.; Tam, W. *Synth. Commun.* **2010**, *40*, 2138. DOI: 10.1080/00397910903219534.
(59) Curti, C.; Battistini, L.; Zanardi, F.; Rassu, G.; Zambrano, V.; Pinna, L.; Casiraghi, G. *J. Org. Chem.* **2010**, *75*, 8681. DOI: 10.1021/jo101799e.
(60) (a) Martzel, T.; Lohier, J. F.; Gaumont, A. C.; Brière, J. F.; Perrio, S. *Eur. J. Org. Chem.* **2018**, 5069. DOI: 10.1002/ejoc.201800749. (b) Ramirez, N. P.; Lana-Villarreal, T.; Gonzalez-Gomez, J. C. *Eur. J. Org. Chem.* **2020**, 1539. DOI: 10.1002/ejoc.201900888.
(61) Grandane, A.; Longwitz, L.; Roolf, C.; Spannenberg, A.; Murua Escobar, H.; Junghanss, C.; Suna, E.; Werner, T. *J. Org. Chem.* **2019**, *84*, 1320. DOI: 10.1021/acs.joc.8b02789.
(62) Papatzimas, J. W.; Gorobets, E.; Maity, R.; Muniyat, M. I.; MacCallum, J. L.; Neri, P.; Bahlis, N. J.; Derksen, D. J. *J. Med. Chem.* **2019**, *62*, 5522. DOI: 10.1021/acs.jmedchem.9b00455.
(63) Kosynkin, D. V.; Tour, J. M. *Org. Lett.* **2001**, *3*, 993. DOI: 10.1021/ol007036g.
(64) Shinohara, H.; Sonoda, M.; Atobe, S.; Masuno, H.; Ogawa, A. *Tetrahedron Lett.* **2011**, *52*, 6238. DOI: 10.1016/j.tetlet.2011.09.068.
(65) Mao, S.; Yuan, B.; Wang, X.; Zhao, Y.; Wang, L.; Yang, X.-Y.; Chen, Y.-M.; Zhang, S.-Q.; Li, P. *Org. Lett.* **2022**, *24*, 3594. DOI: 10.1021/acs.orglett.2c00994.

(66) Wang, C.; Chen, H.; Wang, Z.; Chen, J.; Huang, Y. *Angew. Chem., Int. Ed.* **2012**, *51*, 7242. DOI: 10.1002/anie.201203230.
(67) Soucy, C.; Favreau, D.; Kayser, M. M. *J. Org. Chem.* **1987**, *52*, 129. DOI: 10.1021/jo00377a023.
(68) Padwa, A.; Weingarten, M. D. *J. Org. Chem.* **2000**, *65*, 3722. DOI: 10.1021/jo991938h.
(69) Liao, W.; Liao, Q.; Xu, C.; Wu, X.; Xiong, Y.; Li, Z.; Tang, H. *ACS Appl. Polym. Mater.* **2022**, *4*, 6466. DOI: 10.1021/acsapm.2c00885.
(70) López, H. S.; Enciso, J. E.; Ochoa-Terán, A.; Velazquez, J. I.; Sarmiento, J. I. *Mendeleev Commun.* **2016**, *26*, 69. DOI: 10.1016/j.mencom.2016.01.027.
(71) Basistyi, V. S.; Frederich, J. H. *Org. Lett.* **2022**, *24*, 1907. DOI: 10.1021/acs.orglett.2c00227.
(72) Plangger, I.; Wurst, K.; Magauer, T. *Org. Lett.* **2022**, *24*, 7151. DOI: 10.1021/acs.orglett.2c02843.
(73) Finlay, H.; Adisechan, A. K.; Gunaga, P.; Lloyd, J.; Srinivasu, P. Preparation of Phthalazine Derivatives for Use as Potassium Ion Channel Inhibitors. WO2014143608 A1, 2014.
(74) Woon, E. C. Y.; Zervosen, A.; Sauvage, E.; Simmons, K. J.; Živec, M.; Inglis, S. R.; Fishwick, C. W. G.; Gobec, S.; Charlier, P.; Luxen, A.; Schofield, C. J. *ACS Med. Chem. Lett.* **2011**, *2*, 219. DOI: 10.1021/ml100260x.
(75) Bronstein, H. E.; Choi, N.; Scott, L. T. *J. Am. Chem. Soc.* **2002**, *124*, 8870. DOI: 10.1021/ja0123148.
(76) Kitamura, M.; Kisanuki, M.; Sakata, R.; Okauchi, T. *Chem. Lett.* **2011**, *40*, 1129. DOI: 10.1246/cl.2011.1129.
(77) Jin, J.; León Rojas, A. F.; Zhao, Y.; Helen Kyne, S.; Xia, B.; Zhong, B.; Chan, P. W. H. *Org. Chem. Front.* **2024**, *11*, 290. DOI: 10.1039/d3qo01676e.
(78) Maas, G.; Tretter, A. *Liebigs Ann.* **1985**, 1866. DOI: 10.1002/jlac.198519850913.
(79) Ferguson, G. N.; Valant, C.; Horne, J.; Figler, H.; Flynn, B. L.; Linden, J.; Chalmers, D. K.; Sexton, P. M.; Christopoulos, A.; Scammells, P. J. *J. Med. Chem.* **2008**, *51*, 6165. DOI: 10.1021/jm800557d.
(80) Dao, H. T.; Baran, P. S. *Angew. Chem., Int. Ed.* **2014**, *53*, 14382. DOI: 10.1002/anie.201408022.
(81) Ha, S.; Lee, Y.; Kwak, Y.; Mishra, A.; Yu, E.; Ryou, B.; Park, C.-M. *Nat. Commun.* **2020**, *11*, 2509. DOI: 10.1038/s41467-020-16283-9.
(82) Liashuk, O. S.; Grygorenko, O. O.; Volovenko, Y. M.; Waser, J. *Chem. Eur. J.* **2023**, *29*, e202301650. DOI: 10.1002/chem.202301650.
(83) Vázquez, M. E.; Blanco, J. B.; Imperiali, B. *J. Am. Chem. Soc.* **2005**, *127*, 1300. DOI: 10.1021/ja0449168.
(84) Steinebach, C.; Lindner, S.; Udeshi, N. D.; Mani, D. C.; Kehm, H.; Köpff, S.; Carr, S. A.; Gütschow, M.; Krönke, J. *ACS Chem. Biol.* **2018**, *13*, 2771. DOI: 10.1021/acschembio.8b00693.
(85) Syu, J.-F.; Gopula, B.; Jian, J.-H.; Li, W.-S.; Kuo, T.-S.; Wu, P.-Y.; Henschke, J. P.; Hsieh, M.-C.; Tsai, M.-K.; Wu, H.-L. *Org. Lett.* **2019**, *21*, 4614. DOI: 10.1021/acs.orglett.9b01513.
